# Catalysis by a Metastable Photoswitchable Cage

**DOI:** 10.1002/anie.8106106

**Published:** 2026-09-23

**Authors:** Simona S. Capomolla, Christopher M. Leonhardt, Samina Rasheed, Jake P. Violi, William A. Donald, Paul J. Lusby, Jonathon E. Beves

**Affiliations:** ^1^ School of Chemistry UNSW Sydney Sydney New South Wales Australia; ^2^ Institute of Organic Chemistry Justus Liebig University Giessen Giessen Germany; ^3^ EaStCHEM School of Chemistry University of Edinburgh Edinburgh Scotland UK

**Keywords:** catalysis, non‐equilibrium, photoswitch, self‐assembly, supramolecular

## Abstract

The introduction of *ortho*‐methyl substituents on aryl‐substituted *ortho*‐tetrafluoroazobenzene photoswitches breaks the π‐conjugation and improves photoswitching selectivity and increases the thermal half‐life of the metastable isomer. Using this approach, a visible‐light‐responsive ligand was assembled with a non‐switchable ligand and palladium(II) ions into a mixture of molecular cages. When irradiated with 530 nm light, the system converges to form a single product, a metastable, mixed‐ligand molecular cage. This cage can be reversibly disassembled using 424 nm light, an example of integrative self‐sorting controlled by light. The metastable assembly can catalyze both Michael addition and nitroaldol reactions. Importantly, visible light accesses a metastable catalytic state inaccessible under thermodynamic control. We demonstrate that the active assembled species can be switched on and off at will using visible irradiation and the metastable species is the most catalytically active assembly.

## Graphical abstract

A self‐assembled heteroleptic cage is formed by isomerizing a photoswitchable ligand to its metastable *Z*‐isomeric form (***Z*‐1**) using green light and combining with a non‐switchable ligand (**2**) and palladium(II) ions. The metastable [Pd_2_(***Z‐*1**)_2_(**2**)_2_]^4+^ cage is an out‐of‐equilibrium self‐assembled catalyst, and can be reversibly assembled and disassembled with visible light.

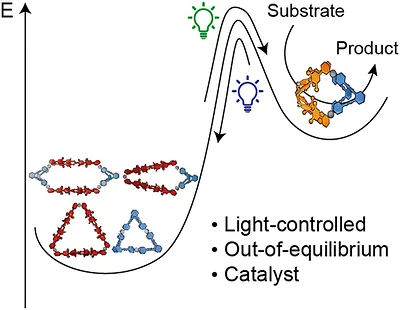

## 1 Introduction

The dynamic properties of self‐assembled systems allow them to be responsive to parameters such as changes in pH [1], concentration [2], solvent [3, 4], temperature [5], redox potential [6], anions [7, 8], or guests [9, 10, 11, 12]. The properties of different assembled states can be used for sensing, transport, or catalysis, for example [13, 14]. In the majority of examples, the change in conditions drives rearrangements towards a new thermodynamic minimum. However, accessing transient assemblies could allow behavior beyond simple switching, and for this to be programmed over time.

Using light to access non‐equilibrium systems [15, 16] is simpler than using chemical fuels where kinetic asymmetry is inherently harder to achieve [17, 18]. There are examples of functional non‐equilibrium systems driven by light such as those that show differences in anion binding [19, 20, 21] and transport [22, 23], as well as molecular pumps to control assembly [24, 25], macrocycle hosts for disequilibrating photoisomerization by selective host‐guest binding [26], or molecular motors that control reaction outcomes [27] or direct the synthesis of complex topologies [25]. Nanoparticles with photoresponsive surfactants have also facilitated switchable catalysis [28], for example by forming an active metastable isomer [29] or using aggregation of nanoparticles [30, 31, 32] to inactivate catalysis [33]. Other examples of nanoparticles with photoswitches attached to the surface have allowed catalysis to be switched off in the metastable form [34, 35]. Small molecule photoswitchable catalysts are also well known [27, 36, 37, 38], however, the most active species is usually the thermodynamically stable form.

Coordination cages [6, 39, 40, 41, 42, 43, 44, 45, 46, 47, 48] have emerged as a useful platform to develop catalysts that take their inspiration from Nature [49, 50, 51, 52, 53, 54, 55, 56, 57]. Among these structures, the Lusby group has shown that the [Pd_2_L_4_]^4+^ class of structures can be highly proficient catalysts for several different transformations [58, 59, 60]. These cages owe much of their catalytic performance to their electro‐positive interior, coupled with the multiple *ortho*‐pyridyl hydrogen‐bond donor atoms that project into the cavity, providing an effective environment for stabilizing high‐energy intermediates (such as deprotonated nucleophiles or leaving groups) or polarized transition states. Nonetheless, there are significant challenges that are specific to coupling catalysis with switching, namely that the use of apolar solvents to maximize host–guest interactions are at odds with the need for coordinating solvents to promote cage rearrangement (and *vice versa*). The structure–activity relationships of cages are also often not predictable, which in the context of interconversion between different structures, means that it is not obvious whether catalysis will be switched on or off in response to a particular stimulus.

Photoresponsive self‐assembled cages [61, 62, 63, 64, 65, 66, 67, 68, 69, 70, 71, 72, 73, 74, 75, 76, 77, 78, 79, 80, 81, 82] have shown properties such as switchable guest binding [61, 62, 63, 65, 69, 83], and more recently catalysis [76, 78, 79]. We have established catalysis within photoswitchable cages [78], showing the cavity of a mixed‐ligand heteroleptic cage was a suitable space for a Michael addition reaction. In that example, the active cage contained the thermodynamically stable isomer of the photoswitchable ligand, which disassembled with light, as the metastable ligand formed only products that were not catalytically active. While metastable cages have been shown to encapsulate guests [83], to the best of our knowledge there are no examples of a catalytically active metastable molecular cage. Herein, we report a system consisting of photoswitchable ligand **1**, non‐switchable ligand **2** [84], and palladium(II) ions (Figure 1).

**FIGURE 1:**
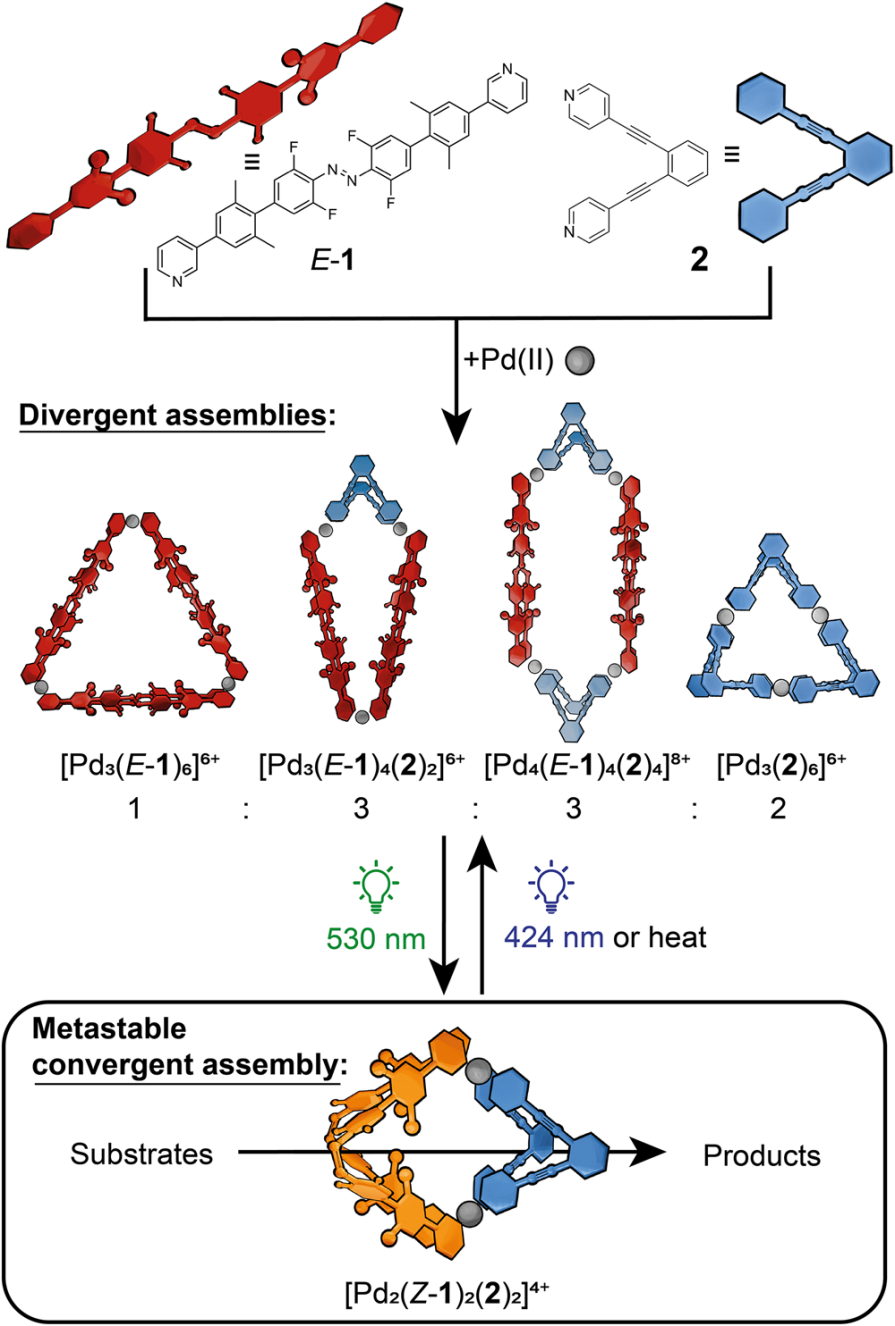
The assembly of heteroleptic species from photoswitchable ligand **1** and non‐switchable ligand **2**.

## 2 Results and Discussion

### 2.1 Optimizing Ligand Photoswitching

*ortho*‐Fluoroazobenzene [85, 86] photoswitches can be switched from stable (*E*) to metastable (*Z*) isomers using only visible light and have exceptionally long thermal half‐lifetimes. These switches have been included into macrocycles [87], and bicycles [88], and we have built them into ligands [67, 68, 78]. The addition of conjugated aromatic rings onto the 4‐position of the azobenzene results in greater overlap in the absorption bands of the *E* and *Z* isomers, and therefore poorer selectivity of switching compared to the parent compound. Here, we improved the selectivity of switching by systematically twisting the appended phenyl ring out of plane using *ortho*‐methyl substituents. As proof of principle, model photoswitches **3–5** (Figure 2) were prepared by Suzuki coupling. Starting with the readily accessible 4,4’‐dibromo‐*ortho*‐tetrafluroazobenzene [67, 86], we prepared the 1,1,2,2‐tetraethylethylene glycol boronic ester B(Epin) [89], which is stable to column chromatography on silica, unlike the pinacol ester (BPin) analogue [67]. From that boronic ester, reaction with the appropriate aryl halide gave good, isolated yields of switches **3** (79%) and **4** (74%), while the more sterically demanding dimethyl derivative **5** (34%) had a lower but still acceptable yield. Each of these photoswitches, including the B(Epin) ester [90], was crystallized in their *E* isomers, and their single crystal X‐ray structures were refined (Figure 2a). This data shows the angle between the planes of the biphenyl rings increases from unsubstituted *E*‐**3** (28° and 31°) to dimethyl *E*‐**4** (43° and 71°) to tetramethyl *E*‐**5** (85° and 90°) [91]. Significantly, these structural changes are accompanied by improved photostationary state (PSS) selectivity (530 nm, %Z: **3**, 78%; **4**, 88%; **5**, 93%) and thermal half‐lives (t_½_) of the metastable isomers (t_½_: **3**, 11 days; **4**, 19 days; **5**, 209 days) due to breaking the biphenyl π orbital overlap.

**FIGURE 2:**
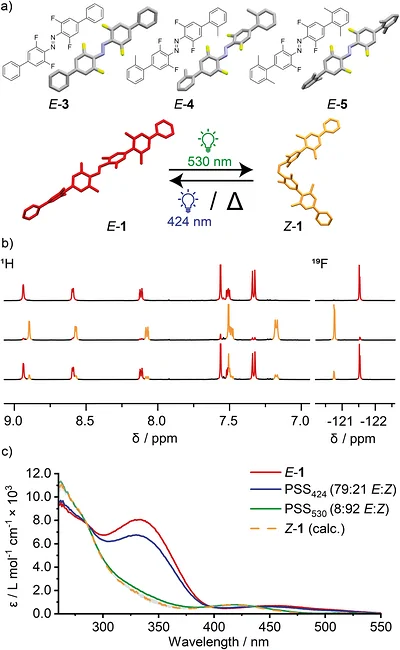
Improving the photoswitching selectivity of substituted *ortho*‐fluoroazobenzene by breaking conjugation. a) Single‐crystal X‐ray structures of switches *E*‐**3**, *E*‐**4**, *E*‐**5**, and photoswitchable ligand in both *E*‐**1** and *Z*‐**1** forms [91] (CCDC: 2552326–2552330). b) ^1^H and ^19^F NMR spectra of photoswitchable ligand **1** in pure *E* form, after irradiation with 530 nm light to give 92% of the *Z*‐isomer, and irradiation with 424 nm light to give 79% of the *E*‐isomer. c) UV–vis absorption spectra of pure *E*‐**1**, after irradiation with 424 nm or 530 nm light, and the calculated spectrum of pure *Z*‐**1**.

We then prepared tetramethyl ligand *E*‐**1** and crystallized both the *E* and *Z* isomers (Figure 2a). In *E*‐**1**, the equivalent angle between the planes of the phenyl rings (79° and 87°) are similar to compound *E*‐**5**, and upon irradiation with 530 nm light a PSS is reached with 92% *Z*‐**1** (Figure 2b,c), with a thermal half‐life of 97 days. This represents a significant improvement on previously reported azobenzene ligands (e.g. di(3‐pyridyl)‐4,4’‐*ortho*‐tetrafluoroazobenzene had a maximum %Z = 80%; *t*_½_ = 25 days) [67]. The crystal structure of *Z*‐**1** shows the expected shape of the ligand with a separation between the two pyridine units of 16 Å (pyridine centroid‐centroid distance, Supporting Information‐13.6).

### 2.2 Self‐Assembly of *E*‐1 With Palladium(II)

With photoswitchable ligand **1** in hand, we turned to its self‐assembly with palladium(II) ions. Reaction of *E*‐**1** with [Pd(CH_3_CN)_4_](BF_4_)_2_ in DMSO‐*d*_6_ gave a single, high symmetry species as evidenced by the ^1^H and ^19^F NMR spectra. Electrospray ionization mass spectrometry (ESI‐MS) revealed a trinuclear species [Pd_3_(*E*‐**1**)_6_](BF_4_)_6_, which we assign as the double‐walled triangle based on the number of ^1^H NMR signals. The analogous reaction in acetonitrile‐*d*_3_ gave a mixture of two major species: one with a single ligand environment in the ^1^H and ^19^F NMR, and one with two different ligand environments in equal abundance. Using ESI‐MS data, and by analogy with previous examples [3, 67, 92, 93, 94], these were assigned to the [Pd_3_(*E*‐**1**)_6_](BF_4_)_6_ trimer, and the [Pd_4_(*E*‐**1**)_8_](BF_4_)_8_ distorted tetrahedron (Supporting Information‐5.2). We next investigated the self‐assembly using [Pd(py*)_4_](BAr_F_)_2_ (where py* = 3‐chloropyrdine; BAr_F_ = tetrakis[3,5‐bis(trifluoromethyl)phenyl]borate), as this would potentially produce an anion‐free cavity that is suitable for catalysis. We have found that the trimer [Pd_3_(*E*‐**1**)_6_]^6+^ is again formed preferentially. Titration experiments in acetonitrile‐*d*_3_ showed the distribution between species [Pd_4_(*E*‐**1**)_8_]^8+^ and [Pd_3_(*E*‐**1**)_6_]^6+^ shifts towards the tetramer when BF_4_^−^ is present. (Supporting Information‐5.3). We explored the photoswitching conditions in either DMSO or acetonitrile. When the solutions of [Pd_3_(*E*‐**1**)_6_](BF_4_)_6_ in DMSO‐*d*_6_, [Pd_3_(*E*‐**1**)_6_](BAr_F_)_6_ in acetonitrile‐*d*_3_, or a mixture of [Pd_3_(*E*‐**1**)_6_](BF_4_)_6_ and [Pd_4_(*E*‐**1**)_8_](BF_4_)_8_ in acetonitrile‐*d*_3_ were irradiated with visible light (530 nm, for 10 min, Supporting Information‐5.4) to isomerize *E*‐**1** to *Z*‐**1**, a complex mixture of ill‐defined species resulted in each case. This indicates that the shape of *Z*‐**1** is not ideal for forming well‐defined discrete species with cavities suitable for catalysis on its own.

### 2.3 Self‐assembly of a Heteroleptic Metastable Cage

Ligand *E*‐**1** has divergent pyridyl lone‐pairs (N···N separation 24.6 Å), which leads to a relatively flat energy landscape of palladium(II) containing self‐assembled structures (e.g. double‐walled triangles, tetrahedra). When *E*‐**1** is combined with the non‐switchable ligand **2** and [Pd(py*)_4_](BAr_F_)_2_ in acetonitrile‐*d*_3_, an even wider range of new species are formed. A selection of analytical techniques indicates that this mixture includes both mixed‐ligand [Pd_3_(*E*‐**1**)_4_(**2**)_2_](BAr_F_)_6_ and [Pd_4_(*E*‐**1**)_4_(**2**)_4_](BAr_F_)_8_ species, in addition to the homoleptic trimers [Pd_3_(*E*‐**1**)_6_](BAr_F_)_6_ and [Pd_3_(**2**)_6_](BAr_F_)_6_. We will subsequently refer to this mixture as “divergent assemblies.” The ^1^H NMR spectrum of “divergent assemblies” (Figure 3c) is consistent with a 3:3:1:2 mixture of these structures, assigned from the symmetry of the species in the NMR spectrum (Supporting Information‐6.1) and ESI‐MS data (Supporting Information‐6.4). Simplistic molecular mechanics models of these cages also suggest that they are feasible structures (Supporting Information‐12).

**FIGURE 3:**
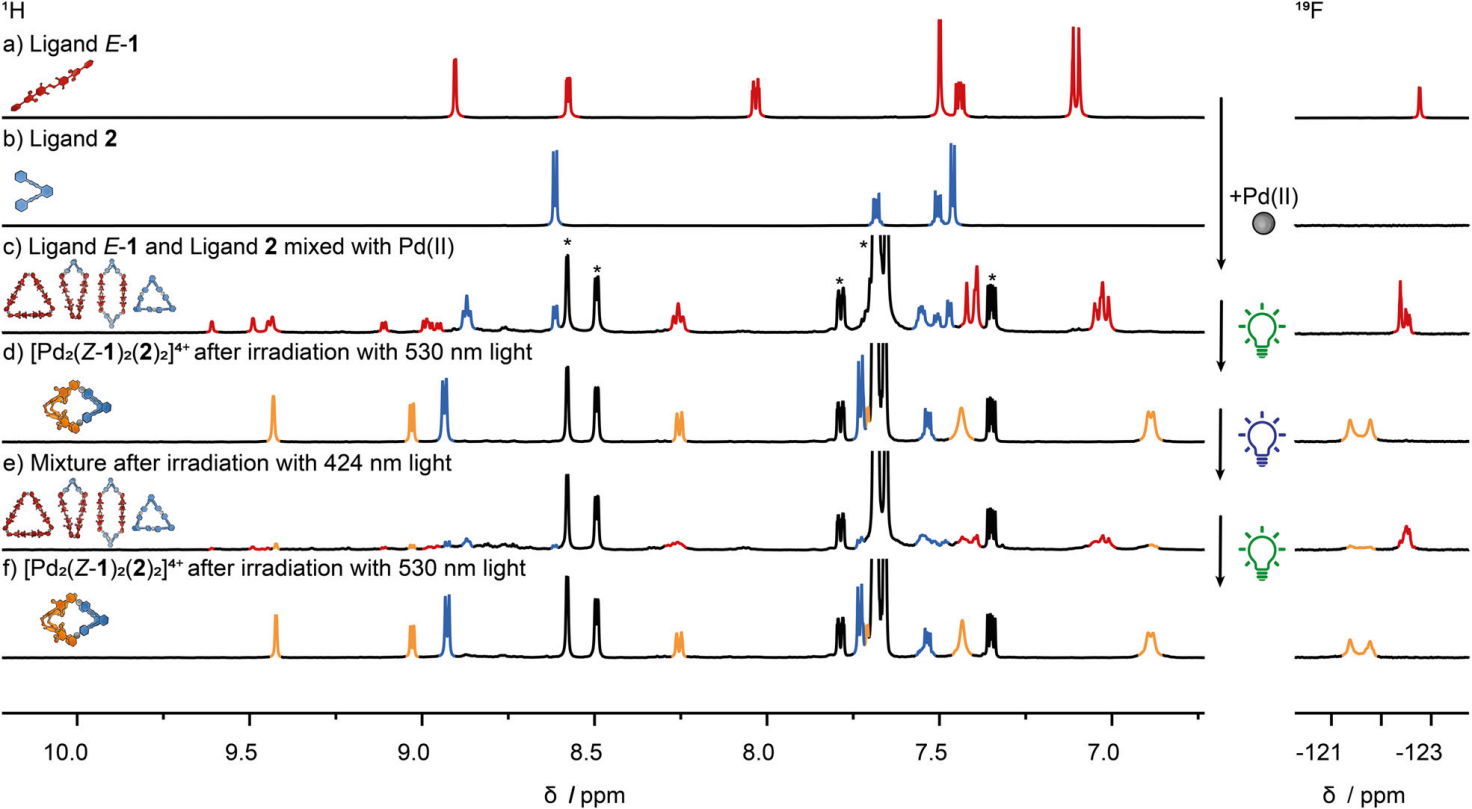
Assembly of photoswitchable ligand **1** and non‐switchable ligand **2** with palladium(II) ions, and response to visible light. ^1^H (600 MHz) and ^19^F (565 MHz) NMR spectra in acetonitrile‐*d*_3_ of a) photoswitchable ligand *E*‐**1**, b) non‐switchable ligand **2**, c) a 1:1:1 mixture of *E*‐**1**: **2**: [Pd(py*)_4_](BAr_F_)_2_ 30 min after mixing and sequential irradiation with d) 530 nm light for 10 min forming [Pd_2_(*Z*‐**1**)_2_(**2**)_2_]^4+^, e) 424 nm light for 10 min, and f) 530 nm light for 10 min again. Peaks denoted with * correspond to py* and BAr_F_.

While the “divergent assembly” mixture indicates a poor geometric complementary between the *E*‐**1** and **2**, the X‐ray structure of *Z*‐**1** suggested a good match with the non‐switchable ligand **2**. When the 1:1:1 mixture of *E*‐**1**, **2** and [Pd(py*)_4_](BAr_F_)_2_ in acetonitrile‐*d*_3_ was irradiated with green (530 nm) light for 10 min, the sample quantitatively assembles into a single new species (Figure 3d), assigned as the dinuclear, heteroleptic cage [Pd_2_(*Z*‐**1**)_2_(**2**)_2_]^4+^. ESI‐MS confirmed the formula of this metastable species, with the major signal corresponding to [Pd_2_(*Z*‐**1**)_2_(**2**)_2_+2(BAr_F_)]^2+^ (Supporting Information‐6.3). A ^1^H ROESY NMR spectrum further showed through‐space contacts between the two different ligands, establishing they are coordinated to the same palladium(II) center (Supporting Information‐6.2). Diffusion NMR data (Supporting Information‐11) confirmed the species has a shorter hydrodynamic radius (5.7 ± 0.3 Å) than that determined for [Pd_3_(*E*‐**1**)_6_]^6+^ (7.9 ± 0.1 Å) and [Pd_3_(**2**)_6_]^6+^ (9.5 ± 0.1 Å). Finally, a single crystal X‐ray structure of the BF_4_^−^ salt unambiguously established the structure (Figure 4). The structure confirms the *Z* configuration of ligand **1**, and as expected from their shape complementarity [39, 40, 41, 82, 95, 96, 97, 98, 99], that the ligands adopt the *cis*‐configuration around the palladium(II) centers, similar to that observed for a related cage formed using a bent *E*‐isomer [78]. The geometries of the two *Z*‐**1** ligands in the cage are similar to that of the free ligand, forming the self‐assembled structure with ligand bend angles of 61° and 64°, compared to 73° in uncoordinated *Z*‐**1**. The intra‐ligand pyridine‐pyridine distances of *Z*‐**1** in the cage (13 Å) are slightly shorter than in the free ligand (16 Å), indicating some compression occurs to match the size of **2**, but that the switchable ligand is largely preorganized for selective heteroleptic complexation as the *Z* isomer. The metastable cage [Pd_2_(*Z*‐**1**)_2_(**2**)_2_](BAr_F_)_4_ can also be obtained by combining equal amounts of the two pre‐formed homoleptic species [Pd_3_(*E*‐**1**)_6_](BAr_F_)_6_ and [Pd_3_(**2**)_6_](BAr_F_)_6_ in acetonitrile‐*d*_3_ and irradiating the mixture with green light (Supporting Information‐6.1). This observation suggests that the product is thermodynamically preferred under green light irradiation. We added 4,4‐dimethylaminopyridine (DMAP) to coordinate all the palladium(II) ions [101] and leave the free ligands readily observable by ^1^H and ^19^F NMR (Supporting Information 6.5). This confirms ligand **1** is quantitatively in the *Z*‐**1** form when irradiated with green light together with palladium(II), compared to 92% when the ligand is irradiated by the same light source in the absence of palladium(II). This is a significant improvement in selectivity, which cannot be achieved by irradiation of the ligand alone with any wavelength of light. The additional stabilization of a metastable isomer when coordinated to palladium(II) was reported for a monomeric complex [68], but to the best of our knowledge has not been reported for a higher order structure.

**FIGURE 4:**
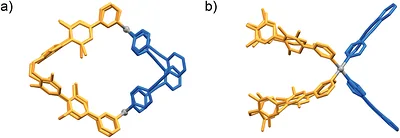
Single crystal X‐ray structure of metastable cage [Pd_2_(*Z*‐**1**)_2_(**2**)_2_]^4+^ in [Pd_2_(*Z*‐**1**)_2_(**2**)_2_](BF_4_)_4_ **·**6CH_3_CN (CCDC: 2580400). Key structural information: C‐N‐N‐C torsion angles 24(3) ° and 7(1) ° confirm *Z* configuration of *Z*‐**1**. Pd···Pd distance 9.982(1) Å [100].

Irradiation of an acetonitrile‐*d*_3_ solution of the metastable cage [Pd_2_(*Z*‐**1**)_2_(**2**)_2_](BAr_F_)_4_ with blue light (424 nm) regenerates *E*‐**1** and the system re‐arranges to form the mixture of “divergent assemblies,” mostly [Pd_3_(*E*‐**1**)_4_(**2**)_2_](BAr_F_)_6_, [Pd_4_(*E*‐**1**)_4_(**2**)_4_](BAr_F_)_8_ and homoleptic [Pd_3_(*E*‐**1**)_6_](BAr_F_)_6_ and [Pd_3_(**2**)_6_](BAr_F_)_6_, in addition to minor species that presumably contain mixed *E*‐**1** and *Z*‐**1** ligands (Figure 3e). Irradiation with 530 nm light completely reassembles [Pd_2_(*Z*‐**1**)_2_(**2**)_2_](BAr_F_)_4_ (Figure 3f), indicating the process is fully reversible.

The thermal half‐life of the metastable cage [Pd_2_(*Z*‐**1**)_2_(**2**)_2_](BAr_F_)_4_ in acetonitrile‐*d*_3_ was too long to measure accurately below the boiling point of the solvent. Therefore, the half‐life was measured in DMSO‐*d*_6_ and compared directly with the free ligand. The room temperature thermal half‐life of ligand *Z*‐**1** in DMSO‐*d*_6_ was estimated to be 97 days (Supporting Information‐4.4), while that of [Pd_2_(*Z*‐**1**)_2_(**2**)_2_](BAr_F_)_4_ is at least several years (Supporting Information‐6.5). To show that [Pd_2_(*Z*‐**1**)_2_(**2**)_2_](BAr_F_)_4_ is metastable and not the thermodynamically favored structure, we heated [Pd_2_(*Z*‐**1**)_2_(**2**)_2_](BAr_F_)_4_ in DMSO‐*d*_6_ for 10 h at 100°C without irradiation. This process reforms the “divergent assemblies” (Supporting Information‐6.5), confirming that these are the thermodynamic products in the dark.

### 2.4 Self‐Sorting Between Different Cages

We have previously reported a thermodynamically stable cage formed between ligand *E*‐**6** (Figure 5a) and non‐switchable ligand **2** [78]. Upon irradiation with green light, this [Pd_2_(*E*‐**6**)_2_(**2**)_2_](BAr_F_)_4_ cage disassembled to give a complex mixture of products, including [Pd(*Z*‐**6**)_2_](BAr_F_)_2_ and [Pd_3_(**2**)_6_](BAr_F_)_6_. We investigated whether this photoswitching behavior could complement that of our new cage (Figure 5b). First, to establish the relative stability of the two heteroleptic cages, we irradiated an acetonitrile‐*d*_3_ solution of **1** with 530 nm light to enrich the sample in *Z*‐**1**. This sample of *Z*‐**1** was added to an acetonitrile‐*d*_3_ solution of [Pd_2_(*E*‐**6**)_2_(**2**)_2_](BAr_F_)_4_. This led to the formation of [Pd_2_(*Z*‐**1**)_2_(**2**)_2_](BAr_F_)_4_ and free ligand *E*‐**6** as major products, showing that [Pd_2_(*Z*‐**1**)_2_(**2**)_2_](BAr_F_)_4_ is the preferred species when ligands *Z*‐**1**, *E*‐**6** and **2** are mixed with palladiumd(II) (Supporting Information‐7.3). We then tested the light‐driven self‐sorting by preparing a 2:2:2 mixture of *E*‐**1,** *E*‐**6** and **2** with three equivalents of [Pd(py*)_4_](BAr_F_)_2_ in acetonitrile‐*d*_3_ (Figure 5c i). Initially, in the dark, the system selectively assembles [Pd_2_(*E*‐**6**)_2_(**2**)_2_](BAr_F_)_4_ due to the shape complementarity of the two ligands, together with homoleptic [Pd_3_(*E*‐**1**)_6_](BAr_F_)_6_ (Figure 5c ii). When irradiated with green light, both ligands **1** and **6** isomerize to their *Z* isomers, and the system rearranges to form [Pd_2_(*Z*‐**1**)_2_(**2**)_2_](BAr_F_)_4_, as now the *Z*‐isomer of **1** is shape complementary to ligand **2,** and the monomeric [Pd(*Z*‐**6**)_2_](BAr_F_)_2_. This effectively allows the non‐switchable ligand **2** to be selectively paired with either ligand **1** or **6** depending on the irradiation conditions (Supporting Information‐7.2).

**FIGURE 5:**
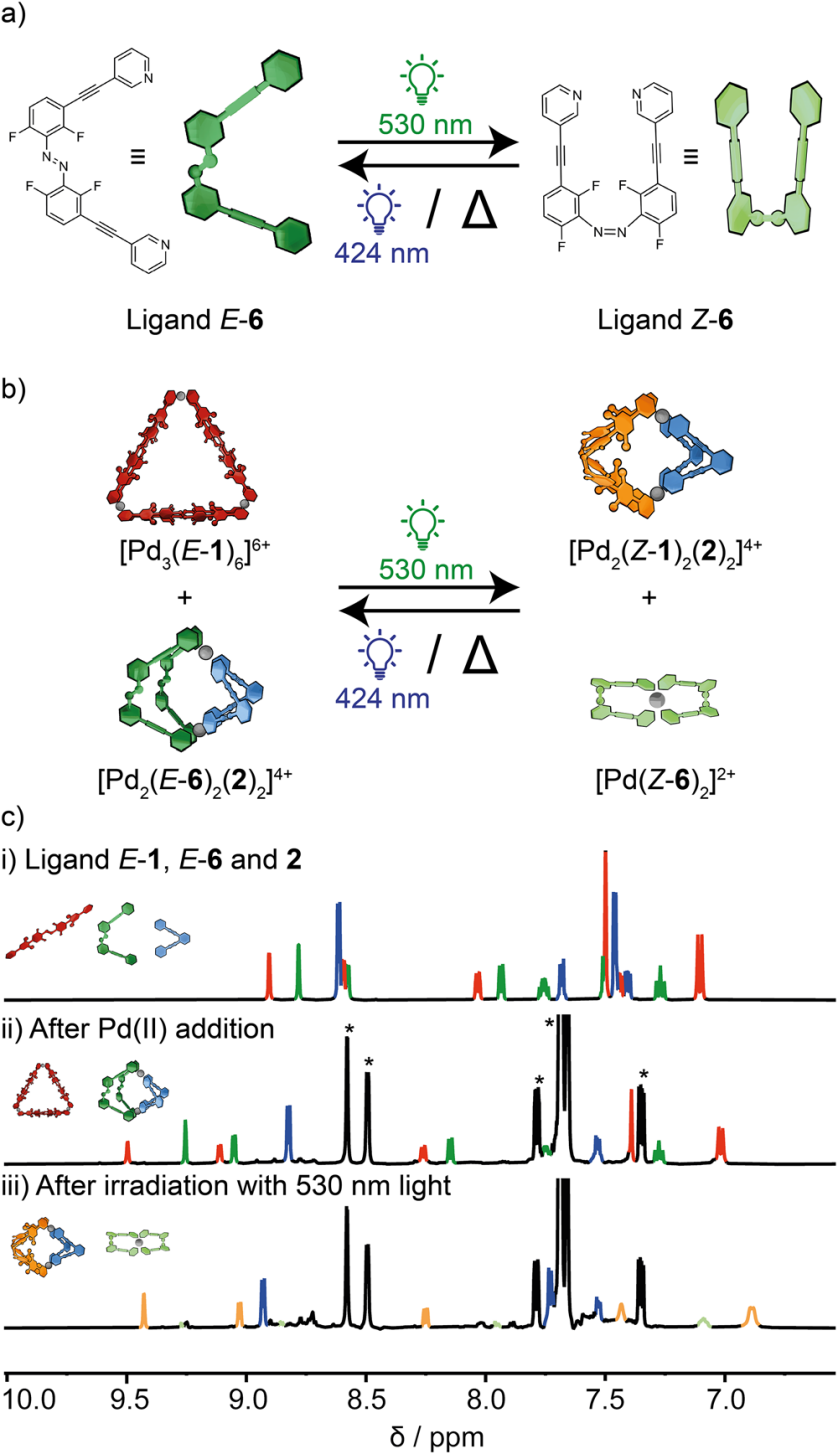
Self‐sorting between self‐assembled cages with light. a) Structure of ligand **6**, b) self‐assembled species present before and after light irradiation, and c) ^1^H (600 MHz, 298 K) NMR spectra in acetonitrile‐*d*_3_ of i) a 1:1:1 mixture of ligand *E*‐**1**, *E*‐**6**, and **2**, ii) after [Pd(py*)_4_](BAr_F_)_2_ addition forming [Pd_2_(*E*‐**6**)_2_(**2**)_2_](BAr_F_)_4_ and [Pd_3_(*E*‐**1**)_6_](BAr_F_)_6_, and iii) after irradiation with 530 nm light forming [Pd_2_(*Z*‐**1**)_2_(**2**)_2_](BAr_F_)_4_ and [Pd(*Z*‐**6**)_2_](BAr_F_)_2_. Peaks denoted with * correspond to py* and BAr_F_.

### 2.5 Catalysis by a Metastable Photoswitchable Cage

We initially chose to test catalysis with the Michael addition between methyl vinyl ketone and benzoyl nitromethane, a transformation which we have shown is catalyzed by our previous photoswitchable cage [78]. Benzoyl nitromethane is bound inside the cage via CH···O hydrogen bonds between the inwardly directed *ortho*‐pyridyl CH of ligands **1** and **2**, and the carbonyl‐O of the substrate. This reaction also uses 18‐crown‐6 as a co‐catalyst to facilitate deprotonation [59], while a mixture of DCM‐*d*_2_:MeCN‐*d*_3_ 11:1 is used to balance catalysis with switching. An assessment of the homoleptic species [Pd_3_(*E*‐**1**)_6_](BAr_F_)_6_ and [Pd_3_(**2**)_6_](BAr_F_)_6_ revealed that these are ineffective catalysts (Supporting Information‐9.4 and 9.6). Moreover, the complete mixture of “divergent assemblies” ([Pd_3_(*E*‐**1**)_4_(**2**)_2_](BAr_F_)_6_, [Pd_4_(*E*‐**1**)_4_(**2**)_4_](BAr_F_)_8_ plus the two homoleptic trimers, Supporting Information‐9.5), only gave 4% product formed over 10 h. In contrast, the metastable assembly [Pd_2_(*Z*‐**1**)_2_(**2**)_2_](BAr_F_)_4_ produced almost 5 times more product over the same time period (Figure 6).

**FIGURE 6:**
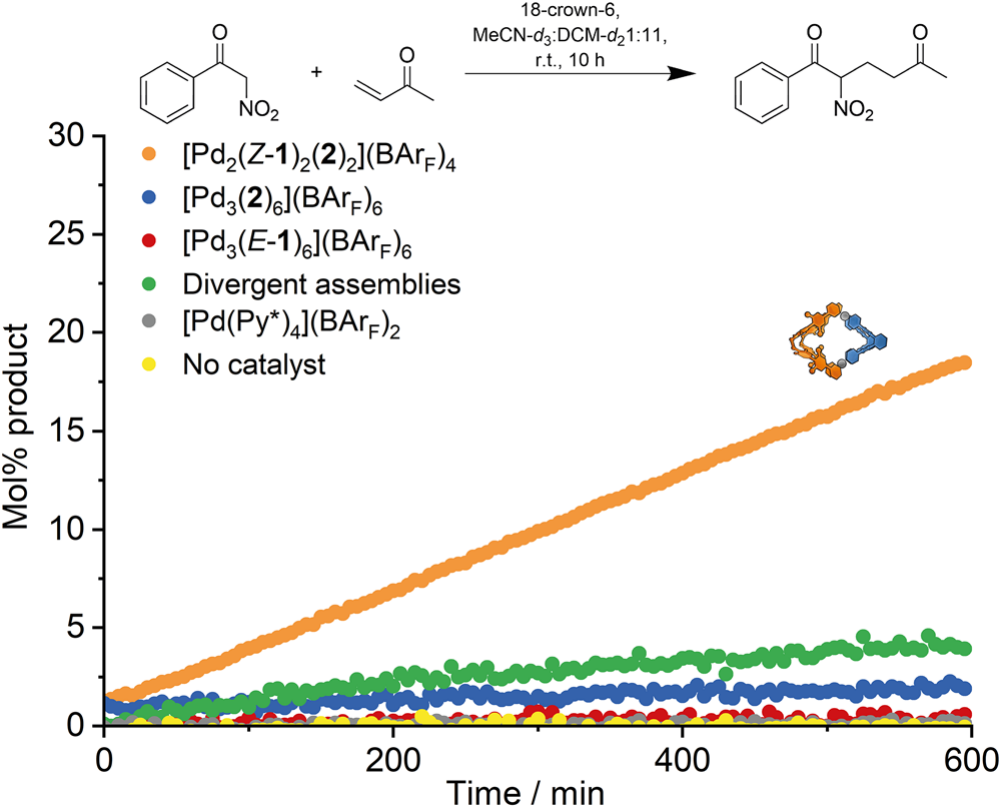
Michael addition in different self‐assembled species. Conditions: benzoyl nitromethane (17.5 mM, 9.8 equiv.), methyl vinyl ketone (31.3 mM, 17.5 equiv.), 18‐crown‐6 (12.5 mM, 7.0 equiv.) and different self‐assembled species ([Pd] = 1.8 mM, 1.0 equiv.) in MeCN‐*d*_3_:DCM‐*d*_2_ 1:11.

To expand the scope of photoswitching catalysis, we next studied the reversible nitroaldol reaction between *para*‐nitrobenzaldehyde and nitromethane‐*d*_3_. We have very recently studied this reaction using a wide range of coordination cages; this investigation revealed that several non‐switchable heteroleptic cages of a similar shape and size to the metastable [Pd_2_(*Z*‐**1**)_2_(**2**)_2_](BAr_F_)_4_ were excellent catalysts [102]. The same investigation also probed the activity of [Pd_3_(**2**)_6_](BAr_F_)_6_, which was observed to be a poorer catalyst. In this current investigation, it was found that the homoleptic [Pd_3_(*E*‐**1**)_6_](BAr_F_)_6_ did not catalyze the reaction at all, but the “divergent assemblies” (the mixture of [Pd_3_(*E*‐**1**)_4_(**2**)_2_](BAr_F_)_6_, [Pd_4_(*E*‐**1**)_4_(**2**)_4_](BAr_F_)_8_, [Pd_3_(*E*‐**1**)_6_](BAr_F_)_6,_ and [Pd_3_(**2**)_6_](BAr_F_)_6_) and metastable cage [Pd_2_(*Z*‐**1**)_2_(**2**)_2_](BAr_F_)_4_ are all effective catalysts (Figure 7a). We estimated the initial rates of reaction from the first 50 min of the reaction and found the metastable cage has the fastest rate with 1.3 × 10^−3^ M·s^−1^, followed by homoleptic [Pd_3_(**2**)_6_]^6+^ with 2.4 × 10^−4^ M·s^−1^, and last the combined “divergent assemblies” with 5.6 × 10^−5^ M·s^−1^ (Supporting Information‐10.2). This order‐of‐magnitude difference between in the activity of the metastable cage compared to the “divergent assemblies” serves as an excellent basis for catalytic photoswitching.

**FIGURE 7:**
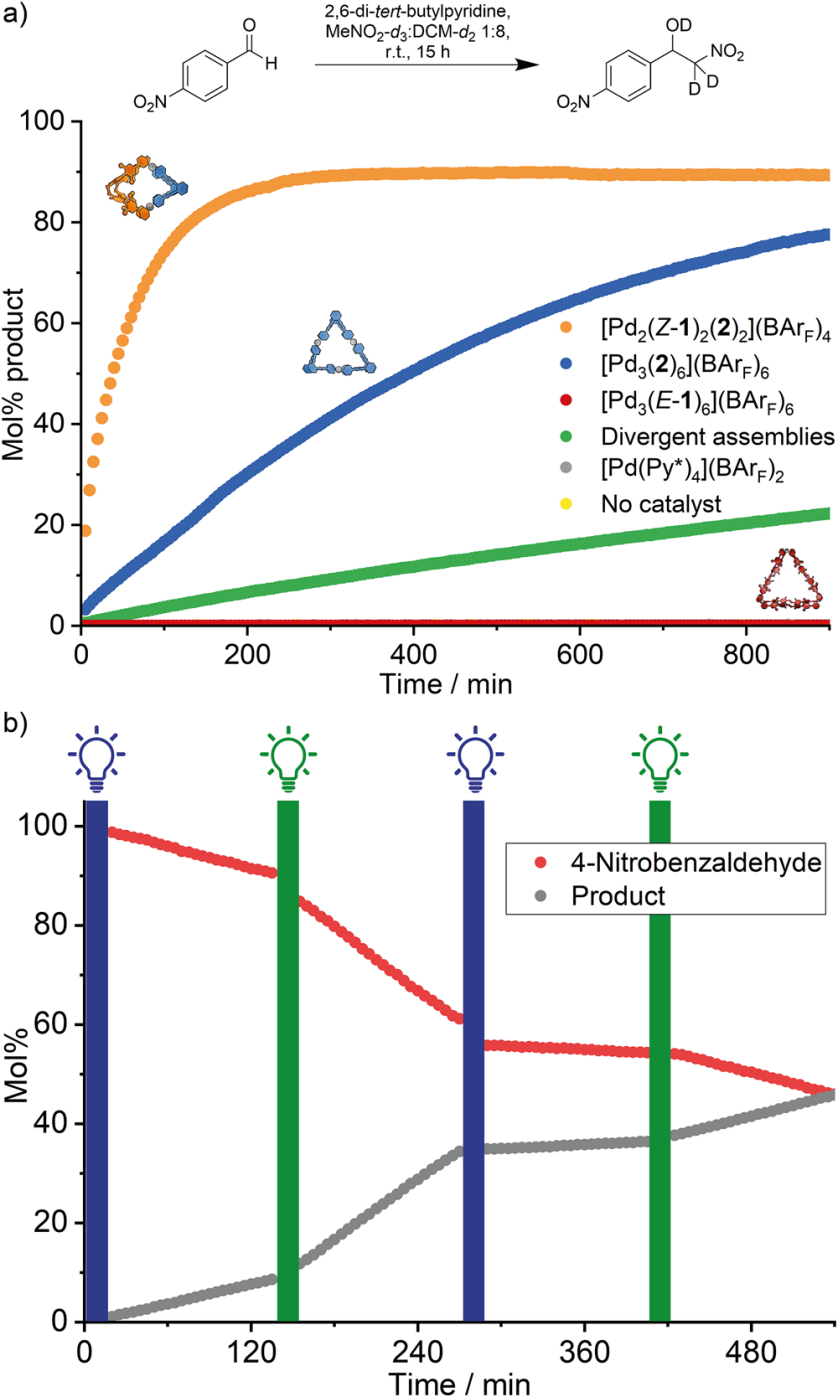
Catalysis for the nitroaldol reaction between *para*‐nitrobenzaldehyde and nitromethane‐*d*_3_, a) catalyzed by homoleptic assemblies [Pd_3_(*E*‐**1**)_6_]^6+^, [Pd_3_(**2**)_6_]^6+^, divergent assemblies such as [Pd_3_(*E*‐**1**)_4_(**2**)_2_]^6+^, [Pd_4_(*E*‐**1**)_4_(**2**)_4_]^8+^ and metastable cage [Pd_2_(*Z*‐**1**)_2_(**2**)_2_]^4+^ (in DCM‐*d*_2_:MeNO_2_‐*d*_3_ 8:1) and b) switchable catalysis with irradiation with alternating 424 nm or 530 nm light every 120 min (in DCM‐*d*_2_:MeCN‐*d*_3_:MeNO_2_‐*d*_3_ 9:1:1.4). Recovery delay d1 = 25 sec to ensure accurate integration. Reaction conditions: 96 mM *para*‐nitrobenzaldehyde, 2.3 mM 2,6‐di‐*tert*‐butylpyridine, all cases with [Pd] = 1.6 mM.

To demonstrate that this catalysis is a cavity‐based process, we carried out inhibition studies using triflate as a strong binding guest [78]. Similar to previous studies, adding tetrabutylammonium triflate to [Pd_2_(*Z*‐**1**)_2_(**2**)_2_](BAr_F_)_4_ (in DCM‐*d*_2_:MeNO_2_‐*d*_3_ 8:1) shows shifts in the ^1^H NMR resonances—characteristic and pronounced deshielding (Δδ ≈1 ppm) of the inward facing *ortho*‐pyridyl signals (Figure S132)—that are consistent with selective encapsulation [78, 103]. In contrast, the remaining cage signals remain largely unchanged. Significantly, adding triflate halts the catalysis by [Pd_2_(*Z*‐**1**)_2_(**2**)_2_](BAr_F_)_4_ and no product is formed in the reaction of *para*‐nitrobenzaldehyde and nitromethane (Supporting Information‐10.9).

Finally, to demonstrate that the catalytic activity can be manipulated using only visible light, we repeated the reaction in a solvent mixture that allows both catalysis and the more rapid interconversion of structures (DCM‐*d*_2_:MeCN‐*d*_3_:MeNO_2_‐*d*_3_ 9:1:1.4). Under these conditions (Figure 7b), the metastable cage can be assembled using green light to initiate the catalysis, then disassembled using violet light. The assembly process shows no signs of fatigue over multiple switching cycles (Supporting Information‐10.11). Together, this data is consistent with the reaction being selective for inside the metastable structure, showing how light can be used to assemble a thermodynamically unstable but functional structure.

## 3 Conclusion

Using light to form non‐equilibrium assemblies offers a pathway to time‐dependent functions. Here, we have used an example with a long thermal half‐life, but the same principle could apply with shorter lived assemblies, where light intensity and duration would lead to different evolving functions over time. Such behavior allows the energy of absorbed light to be used for catalyzing a chemical bond forming reaction, where no photo process is involved in the catalysis. In the case we present such photo reactions can be eliminated as the catalysis is observed to continue for hours after the light is switched off. We anticipate that catalytically active non‐equilibrium assemblies could be used for more complex evolving behaviors, including for information processing [104], and multistep reactions.

## Author Contributions

**Simona S. Capomolla**: conceptualization, investigation, synthesis, NMR and XRD experiments, data analysis, manuscript writing and visualization. **Christopher M. Leonhardt**: synthesis, methodology. **Samina Rasheed**: synthesis, conceptualization. **Jake P. Violi**: ESI‐MS experiments. **William A. Donald**: ESI‐MS experiments. **Paul J. Lusby**: conceptualization, methodology, data analysis, manuscript writing. **Jonathon E. Beves**: conceptualization, methodology, data analysis, manuscript writing.

## Conflicts of Interest

The authors declare no conflict of interest.

## Supporting information



Experimental details, spectroscopic data and analysis are supplied as Supporting Information. A preprint of this work, including all experimental data, is available at *ChemRxiv*, DOI:   https://chemrxiv.org/doi/full/10.26434/chemrxiv.15003829/v5, 29 July 2026.
The authors have cited additional references within the Supporting Information [105, 106, 107, 108, 109, 110, 111, 112, 113, 114, 115, 116, 117, 118].
**Supporting File**: anie74709‐sup‐0001‐SuppMat.pdf.

## Data Availability

The data that support the findings of this study are openly available in ChemRxiv at https://chemrxiv.org/doi/full/10.26434/chemrxiv.15003829/v5.
