# Supplementary material for: Catalysis by a Metastable Photoswitchable Cage
Source: Angew Chem Int Ed Engl. 2026 Sep 23;65(46):e8106106. doi: 10.1002/anie.8106106 (PMC13749340; doi:10.1002/anie.8106106)
Supplement: Supplementary file 1 [file ANIE-65-e8106106-s001.pdf]

Supporting Information

Catalysis by a metastable photoswitchable cage

Simona S. Capomolla[a], Christopher M. Leonhardt[b], Samina Rasheed[a], Jake P. Violi[a], William A. Donald[a], Paul J. Lusby[c]* and Jonathon E. Beves[a]*

[a]School of Chemistry, UNSW Sydney, Sydney, New South Wales 2052, Australia

[b]Institute of Organic Chemistry, Justus Liebig University Giessen, 35392 Giessen, Germany

[c]EaStCHEM, School of Chemistry, University of Edinburgh, Joseph Black Building, David Brewster Road, Edinburgh, Scotland, EH9 3FJ, U.K

Table of contents

1 General Experimental 6
1.1 General Experimental 6

2 Synthesis of photoswitches 3, 4 and 5 7
2.1 Synthetic overview 7
2.2 Synthesis of 1,2-bis(4-bromo-2,6-difluorophenyl)diazene (**S1**) 8
2.3 Synthesis and characterization of intermediate photoswitch **S2** 9
2.3.1 Synthesis of intermediate photoswitch **S2** 9
2.3.2 1D and 2D NMR characterization of intermediate photoswitch **S2** 10
2.4 Synthesis and characterization of photoswitch **3** 13
2.4.1 Synthesis of photoswitch **3** 13
2.4.2 1D NMR characterization of photoswitch **3** in DMSO-d_6 14
2.5 Synthesis and characterization of photoswitch **4** 16
2.5.1 Synthesis of photoswitch **4** 16
2.5.2 1D and 2D NMR characterization of photoswitch **4** 17
2.6 Synthesis and characterization of photoswitch **5** 21
2.6.1 Synthesis of photoswitch **5** 21
2.6.2 1D and 2D NMR characterization of photoswitch **5** 22

3 Synthesis of photoswitchable ligand 1 26
3.1 Synthetic overview 26
3.2 Synthesis and characterization of 4-bromo-2,6-dimethylphenol (**S3**) 27
3.2.1 Synthesis of 4-bromo-2,6-dimethylphenol (**S3**) 27
3.2.2 1D NMR of 4-bromo-2,6-dimethylphenol (**S3**) 28
3.3 Synthesis and characterization of 2,6-dimethyl-4-(pyridin-3-yl)phenol (**S4**) ... 29
3.3.1 Synthesis of 2,6-dimethyl-4-(pyridin-3-yl)phenol (**S4**) 29
3.3.2 1D and 2D NMR characterization of 2,6-dimethyl-4-(pyridin-3-yl)phenol (**S4**) 30
3.4 Synthesis and characterization of intermediate **S5** 34
3.4.1 Synthesis of intermediate **S5** 34
3.4.2 1D and 2D NMR of intermediate **S5** 35
3.5 Synthesis and characterization of ligand **1** 40
3.5.1 Synthesis of ligand **1** 40
3.5.2 1D and 2D NMR characterization of ligand *E*-**1** 41
3.5.3 1D NMR characterization of a mixture of ligand *E*-**1** and *Z*-**1** 46

4 Photoswitching properties of photoswitches 3, 4, 5 and ligand 1 48
4.1 Photoswitching properties of **3** 48
4.1.1 Determining the PSS distributions of photoswitch **3** in DMSO-d_6 using ^{1}H and ^{19}F NMR spectroscopy 48
4.1.1 UV-vis spectra of photoswitch **3** in DMSO after irradiation with different wavelengths 48
4.1.2 Determining the thermal half-life of photoswitch *Z*-**3** in DMSO 48
4.2 Photoswitching properties of **4** 52
4.2.1 Determining the PSS distributions of photoswitch **4** in DMSO-d_6 using ^{1}H and ^{19}F NMR spectroscopy 52
4.2.2 UV-vis spectra of photoswitch **4** in DMSO after irradiation with different wavelengths 57
4.2.3 Determining the thermal half-life of photoswitch *Z*-**4** in DMSO 59
4.3 Photoswitching properties of **5** 62
4.3.1 Determining the PSS distributions of photoswitch **5** in DMSO-d_6 using ^{1}H and ^{19}F NMR spectroscopy 62
4.3.2 UV-vis spectra of photoswitch **5** in DMSO after irradiation with different wavelengths 65
4.3.3 Determining the thermal half-life of photoswitch *Z*-**5** in DMSO 67
4.4 Photoswitching properties of ligand **1** 70

4.4.1 Determining the PSS distributions of ligand **1** in DMSO-*d*$_6$ using ^{1}H and ^{19}F NMR spectroscopy ... 70
4.4.2 UV-vis spectra of ligand **1** in DMSO after irradiation with different wavelengths ... 74
4.4.3 Determining the thermal half-life of ligand *Z*-**1** in DMSO ... 76
4.5 Summary photochromic properties of photoswitch **3**, **4**, **5** and ligand **1** ... 79

5 Synthesis and characterization of homoleptic cages $[Pd_3(E\text{-}1)_6]^{6+}$ and $[Pd_4(E\text{-}1)_8]^{8+}$ ... 80
5.1 Self assembly of $[Pd_3(E\text{-}\mathbf{1})_6](BAr_F)_6$ in MeCN-*d*$_3$ ... 80
5.1.1 1D and 2D NMR characterization of $[Pd_3(E\text{-}\mathbf{1})_6](BAr_F)_6$ in MeCN-*d*$_3$ ... 81
5.1.2 ESI-MS of $[Pd_3(E\text{-}\mathbf{1})_6](BAr_F)_6$ in MeCN-*d*$_3$ ... 85
5.1.3 Self-assembly of $[Pd_3(E\text{-}\mathbf{1})_6](BF_4)_6$ in DMSO-*d*$_6$ ... 86
5.1.4 1D NMR characterization of $[Pd_3(E\text{-}\mathbf{1})_6](BF_4)_6$ in DMSO-*d*$_6$ ... 87
5.1.5 ESI-MS of $[Pd_3(E\text{-}\mathbf{1})_6](BF_4)_6$ in DMSO ... 88
5.2 Self-assembly of $[Pd_3(E\text{-}\mathbf{1})_6](BF_4)_6$ and $[Pd_4(E\text{-}\mathbf{1})_8](BF_4)_8$ in MeCN-*d*$_3$ ... 89
5.2.1 1D and 2D NMR characterization of $[Pd_3(E\text{-}\mathbf{1})_6](BF_4)_6$ and $[Pd_4(E\text{-}\mathbf{1})_8](BF_4)_8$ in MeCN-*d*$_3$ ... 90
5.2.2 ESI-MS of the mixture of $[Pd_3(E\text{-}\mathbf{1})_6](BF_4)_6$ and $[Pd_4(E\text{-}\mathbf{1})_8](BF_4)_8$ in MeCN-*d*$_3$ ... 94
5.3 Interconversion of $[Pd_3(E\text{-}\mathbf{1})_6](BF_4)_6$ and $[Pd_4(E\text{-}\mathbf{1})_8](BF_4)_8$ into $[Pd_3(E\text{-}\mathbf{1})_6](BAr_F)_6$ and vice versa by counter anion exchange ... 96
5.3.1 Interconverting $[Pd_3(E\text{-}\mathbf{1})_6](BAr_F)_6$ into $[Pd_3(E\text{-}\mathbf{1})_6](BF_4)_6$ and $[Pd_4(E\text{-}\mathbf{1})_8](BF_4)_8$ by adding $NaBF_4$ ... 96
5.3.2 Interconverting $[Pd_3(E\text{-}\mathbf{1})_6](BF_4)_6$ and $[Pd_4(E\text{-}\mathbf{1})_8](BF_4)_8$ into $[Pd_3(E\text{-}\mathbf{1})_6](BAr_F)_6$ by adding $NaBAr_F$ ... 101
5.4 Photoswitching of homoleptic $[Pd_3(E\text{-}\mathbf{1})_6]^{6+}$ and $[Pd_4(E\text{-}\mathbf{1})_8]^{8+}$ species ... 106
5.4.1 Photoswitching of $[Pd_3(E\text{-}\mathbf{1})_6](BAr_F)_6$ in MeCN-*d*$_3$ ... 106
5.4.2 Photoswitching of $[Pd_3(E\text{-}\mathbf{1})_6](BF_4)_6$ in DMSO-*d*$_6$ ... 107
5.4.3 Photoswitching of the mixture of $[Pd_3(E\text{-}\mathbf{1})_6](BF_4)_6$ and $[Pd_4(E\text{-}\mathbf{1})_8](BF_4)_8$ in MeCN-*d*$_3$ ... 110

6 Synthesis and characterization of heteroleptic metastable $[Pd_2(Z\text{-}1)_2(2)_2](BAr_F)_4$ in MeCN-*d*$_3$ ... 112
6.1 Synthesis of heteroleptic metastable $[Pd_2(Z\text{-}\mathbf{1})_2(\mathbf{2})_2](BAr_F)_4$ in MeCN-*d*$_3$ ... 112
6.2 1D and 2D NMR characterization of $[Pd_2(Z\text{-}\mathbf{1})_2(\mathbf{2})_2](BAr_F)_4$ ... 114
6.3 ESI-MS of $[Pd_2(Z\text{-}\mathbf{1})_2(\mathbf{2})_2](BAr_F)_4$ in MeCN-*d*$_3$ ... 119
6.4 ESI-MS of the mixture of $[Pd_3(E\text{-}\mathbf{1})_4(\mathbf{2})_2](BAr_F)_6$ and $[Pd_4(E\text{-}\mathbf{1})_4(\mathbf{2})_4](BAr_F)_8$ in MeCN-*d*$_3$ ... 120
6.5 Photoswitching and thermal stability of heteroleptic $[Pd_2(Z\text{-}\mathbf{1})_2(\mathbf{2})_2](BAr_F)_4$ ... 121
6.5.1 Irradiating $[Pd_2(Z\text{-}\mathbf{1})_2(\mathbf{2})_2](BAr_F)_4$ with 530 nm and 424 nm light ... 121
6.5.2 Determining the PSS distribution of $[Pd_2(Z\text{-}\mathbf{1})_2(\mathbf{2})_2](BAr_F)_4$ in MeCN-*d*$_3$ after irradiation with 530 nm light ... 123
6.5.3 Thermal half-life of $[Pd_2(Z\text{-}\mathbf{1})_2(\mathbf{2})_2](BAr_F)_4$ in DMSO-*d*$_6$ ... 125

7 Integrative self-sorting of photoswitchable cages ... 126
7.1 General ... 126
7.2 Self-sorting of ligands *E*-**1**, *E*-**6** and **2** ... 128
7.3 Competition experiments ... 131
7.3.1 Adding ligand *E*-**6** to $[Pd_2(Z\text{-}\mathbf{1})_2(\mathbf{2})_2](BAr_F)_4$ ... 131
7.3.2 Adding ligand *Z*-**1** to $[Pd_2(E\text{-}\mathbf{6})_2(\mathbf{2})_2](BAr_F)_4$ ... 133

8 Catalysis experiments - general ... 135
8.1 Preparation of self-assembled species for catalysis ... 135
8.2 Preparation of a $[Pd_3(E\text{-}\mathbf{1})_6](BAr_F)_6$ sample ... 135
8.3 Preparation of a $[Pd_3(\mathbf{2})_6](BAr_F)_6$ sample ... 136
8.4 Preparation of a mixture of $[Pd_3(E\text{-}\mathbf{1})_6](BAr_F)_6$, $[Pd_3(\mathbf{2})_6](BAr_F)_6$, $[Pd_3(E\text{-}\mathbf{1})_4(\mathbf{2})_2](BAr_F)_6$ and $[Pd_4(E\text{-}\mathbf{1})_4(\mathbf{2})_4](BAr_F)_8$ sample ... 136
8.5 Preparation of a $[Pd_2(Z\text{-}\mathbf{1})_2(\mathbf{2})_2](BAr_F)_4$ sample ... 137

9 Catalysis: Michael addition between benzoyl nitromethane and methyl vinyl ketone catalysed by different self-assembled species 138
9.1 Preparation of stock solutions used for Michael addition catalysis samples 138
9.2 Monitoring the Michael addition reaction between benzoyl nitromethane and methyl vinyl ketone using ^{1}H NMR spectroscopy 139
9.3 Using $[Pd_2(Z\text{-}\mathbf{1})_2(\mathbf{2})_2](BAr_F)_4$ to catalyze the Michael addition reaction between benzoyl nitromethane and methyl vinyl ketone... 140
9.4 Using $[Pd_3(\mathbf{2})_6](BAr_F)_6$ to catalyze the Michael addition reaction between benzoyl nitromethane and methyl vinyl ketone.. 141
9.5 Using a mixture of $[Pd_3(E\text{-}\mathbf{1})_6](BAr_F)_6$, $[Pd_3(\mathbf{2})_6](BAr_F)_6$, $[Pd_3(E\text{-}\mathbf{1})_4(\mathbf{2})_2](BAr_F)_6$ and $[Pd_4(E\text{-}\mathbf{1})_4(\mathbf{2})_4](BAr_F)_8$ to catalyze the Michael addition reaction between benzoyl nitromethane and methyl vinyl ketone.. 142
9.6 Using $[Pd_3(E\text{-}\mathbf{1})_6](BArF)_6$ / $[Pd_4(E\text{-}\mathbf{1})_8](BArF)_8$ to catalyze the Michael addition reaction between benzoyl nitromethane and methyl vinyl ketone 143
9.7 Monitoring the Michael addition reaction between benzoyl nitromethane and methyl vinyl ketone using $[Pd(Py^*)_4](BAr_F)_2$ 145
9.8 Monitoring the Michael addition reaction between benzoyl nitromethane and methyl vinyl ketone using no catalyst 146

10 Catalysis: Nitroaldol reaction between nitromethane and 4-nitrobenzaldehyde 147
10.1 Preparation of stock solutions used for the nitroaldol reaction 147
10.2 Monitoring the nitroaldol reaction between 4-nitrobenzaldehyde and nitromethane in self-assembled species using ^{1}H NMR spectroscopy......... 147
10.2.1 General procedure for the nitroaldol reaction between 4-nitrobenzaldehyde and nitromethane 147
10.2.2 Calculating the conversion of the nitroaldol reaction between 4-nitrobenzaldehyde and nitromethane 148
10.3 Using $[Pd_2(Z\text{-}\mathbf{1})_2(\mathbf{2})_2](BAr_F)_4$ to catalyze the nitroaldol reaction between 4-nitrobenzaldehyde and nitromethane.. 150
10.4 Using $[Pd_3(\mathbf{2})_6](BAr_F)_6$ to catalyze the nitroaldol reaction between 4-nitrobenzaldehyde and nitromethane.. 151
10.5 Using $[Pd_3(E\text{-}\mathbf{1})_6](BAr_F)_6$ and $[Pd_4(E\text{-}\mathbf{1})_8](BAr_F)_8$ to catalyze the nitroaldol reaction between 4-nitrobenzaldehyde and nitromethane........................... 152
10.6 Using a mixture of $[Pd_3(E\text{-}\mathbf{1})_6](BAr_F)_6$, $[Pd_3(\mathbf{2})_6](BAr_F)_6$, $[Pd_3(E\text{-}\mathbf{1})_4(\mathbf{2})_2](BAr_F)_6$ and $[Pd_4(E\text{-}1)_4(2)_4](BAr_F)_8$ to catalyze the nitroaldol reaction between 4-nitrobenzaldehyde and nitromethane.. 153
10.7 Monitoring the nitroaldol reaction between 4-nitrobenzaldehyde and nitromethane using $[Pd(Py^*)_4](BAr_F)_2$... 154
10.8 Monitoring the nitroaldol reaction between 4-nitrobenzaldehyde and nitromethane using no catalyst... 155
10.9 Competitive binding: Preventing the nitroaldol reaction by adding triflate to the metastable cage.. 156
10.10 Binding of triflate (TBAOTf) by $[Pd_2(Z\text{-}\mathbf{1})_2(\mathbf{2})_2](BAr_F)_4$ in:$MeNO_2$-d_3:DCM-d_2 8:1 157
10.11 Manipulating the nitroaldol catalysis rates using visible light.................. 160
10.11.1 Switching nitroaldol catalysis in DCM-d_2:$MeNO_2$-d_3 8:1 160
10.11.2 Switching nitroaldol catalysis in DCM-d_2:MeCN-d_3:MeNO2-d_3 9:1:1.4 162

11 Diffusion NMR measurements 165
11.1 Gradient calibration 165
11.2 Diffusion measurements for $[Pd_3(E\text{-}\mathbf{1})_6](BAr_F)_6$ 166
11.3 Diffusion measurements of the mixture of $[Pd_3(E\text{-}\mathbf{1})_6](BF_4)_6$ and $[Pd_4(E\text{-}\mathbf{1})_8](BF_4)_8$ 167
11.4 Diffusion measurements for $[Pd_2(Z\text{-}\mathbf{1})_2(\mathbf{2})_2](BAr_F)_4$ 168

11.5 Diffusion measurements for [Pd_3(**2**)$_6$](BAr_F)$_6$ 169
11.6 Summary Diffusion measurements 170

12 Spartan models of [Pd_3(*E*-1)$_6$]$^{6+}$, [Pd_4(*E*-1)$_8$]$^{8+}$, [Pd_3(*E*-1)$_4$(2)$_2$]$^{6+}$, [Pd_4(*E*-1)$_4$(2)$_4$]$^{8+}$ and [Pd_2(*Z*-1)$_2$(2)$_2$]$^{4+}$ 172

13 Single crystal X-ray structures 175
13.1 Single crystal X-ray structure of **S2** (CCDC: 2552325) 175
13.2 Single crystal X-ray structure of photoswitch **3** (CCDC: 2552326) 177
13.3 Single crystal X-ray structure of photoswitch **4** (CCDC: 2552327) 179
13.4 Single crystal X-ray structure of photoswitch **5** (CCDC: 2552328) 181
13.5 Single crystal X-ray structure of ligand *E*-**1** (CCDC: 2552329) 183
13.6 Single crystal X-ray structure of ligand *Z*-**1** (CCDC: 2552330) 185
13.7 Single crystal X-ray structure of [Pd_2(*Z*-**1**)$_2$(**2**)$_2$](BF_4)$_4$ (CCDC: 2580400) 187

14 References 189

1 General Experimental

1.1 General Experimental

Reagents and solvents were purchased from Sigma-Aldrich, Merck, Chem Supply, Combi-Blocks, Ambeed or Alfa Aeser, and were used without purification unless stated otherwise.

The palladium complex $[Pd(Py^*)_4](BArF)_2$[1-2], ligand **2**[2-3] and ligand **6**[2] were prepared following previously reported procedures.

Anhydrous solvents were dispensed from an Inertcorp solvent purification system (model number: PS-MD-7).

Microwave reactions were carried out in a Biotage® Initiator+ microwave synthesizer.

Flash chromatography was performed on the Biotage® Selekt System using Biotage® Sfär high-capacity silica columns or self-packed columns (SiliaFlash® P60, 40-63 µm). Ethyl acetate in hexane or methanol in dichloromethane were used as eluents.

NMR spectroscopy was performed using a Bruker Avance III 400 with a Prodigy CryoProbe, a Bruker Avance III 500, a Bruker Avance III 600 or a Bruker Avance III HD 600 with a TCI CryoProbe. All chemical shifts were calibrated against residual solvent signals. All coupling constants (*J*) are reported in Hertz. Signals in the NMR spectra are reported as broad (br), singlet (s), doublets (d), triplets (t), quartets (q), quintets (qu), sextets (sx), septets (sept), or unclear multiplets (m). NMR spectra were processed with MestReNova 14.2.1 software. All NMR data are assigned unambiguously, except where specified. The broad peak observed in ^{19}F spectra between −150 and −210 ppm corresponds to a Teflon matrix, which is a standard component of all Bruker probes.

UV-vis experiments were performed on an Agilent Cary 60 Bio UV-visible Spectrophotometer equipped with a customized Cary Single Cell Peltier Accessory, keeping the samples at 25 °C unless stated otherwise. The cell holder was modified to allow for irradiation perpendicular to the direction of measurement. A Luxeon Rebel LED was mounted on a heat sink 4 cm from the cell and driven using a 1000 mA LuxDrive PowerPuck. The sample was analyzed in a quartz cuvette with a path length of 1 cm. All samples were stirred to ensure homogeneity Recording UV-vis absorption spectra of cage samples proved difficult as high concentrations (mM) are required for cage assembly in coordinating solvents such as DMSO or acetonitrile. Such conditions are not compatible with even short (2 mm) pathlengths cuvettes used for UV-vis as the absorption is too high to accurately measure, and if the sample is diluted the cage composition can no longer be verified by NMR spectroscopy.

High-resolution mass spectrometry (HR-MS) experiments were performed on a hybrid linear quadrupole ion trap mass spectrometer (Thermo LTQ Orbitrap XL) equipped with a nanospray ionisation (NSI) source.

Micheal addition substrates were purified as follows before use: methyl vinyl ketone was dried over K_2CO_3 and $CaCl_2$ and distilled at 30 °C under reduced pressure; benzoyl nitromethane was recrystallized from isopropanol.

Table S1 Specifications for the LEDs used in this work.

LED Part Number (supplier)	Dominant wavelength / nm
J035-L1C1VLT (Luxeon)	424
J035-L1C1ROY (Luxeon)	445
LXML-PM01-0100 Rebel LED (SinkPAD-II)	530

2 Synthesis of photoswitches 3, 4 and 5

2.1 Synthetic overview

The photoswitches **3**, **4** and **5** were synthesized in three steps from the commercially available 4-bromo-2,6-difluoroaniline using modified literature procedures.[4-5] Baeyer-Mills coupling of two equivalents of 4-bromo-2,6-difluoroaniline afforded the intermediate 1,2-bis(4-bromo-2,6-difluorophenyl)diazene (**S1**) in 52% yield. Miyaura borylation conditions were applied to intermediate **S1** using 4,4,5,5-tetraethyl-2-(4,4,5,5-tetraethyl-1,3,2-dioxaborolan-2-yl)-1,3,2-dioxaborolane ($B_2(Epin)_2$) yielding the $B_2(Epin)_2$ coupled precursor **S2**. Successive Suzuki-Miyaura coupling with an excess of either iodobenzene, 1-bromo-2-methylbenzene, or 2-bromo-1,3-dimethylbenzene yielded the photoswitches **3**, **4** and **5** as a mixture of *E*/*Z* isomers in 79%, 74% and 34% yield, respectively.

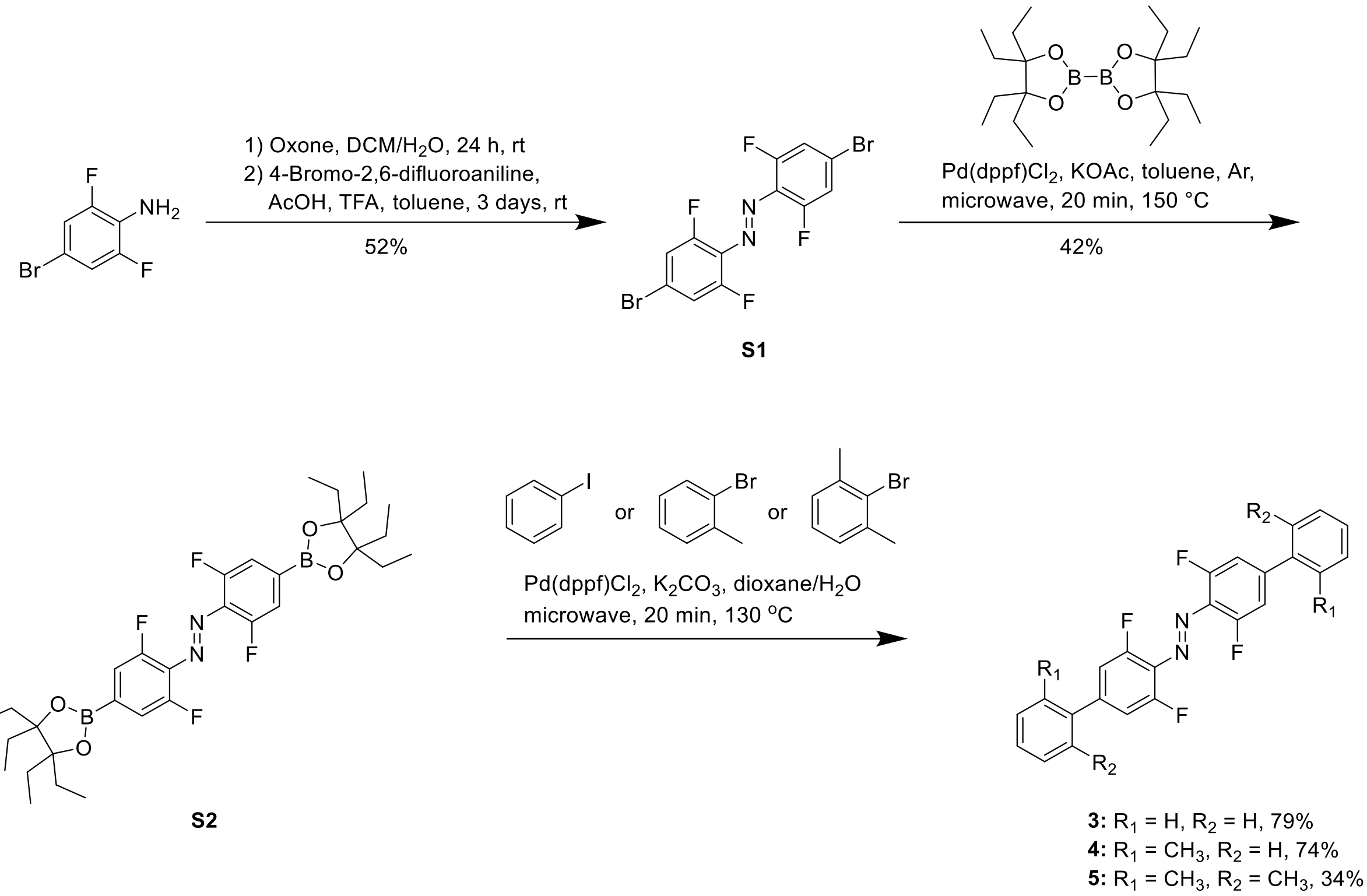

Scheme S1. Synthetic pathway for the photoswitches **3**, **4** and **5.**

2.2 Synthesis of 1,2-bis(4-bromo-2,6-difluorophenyl)diazene (S1)

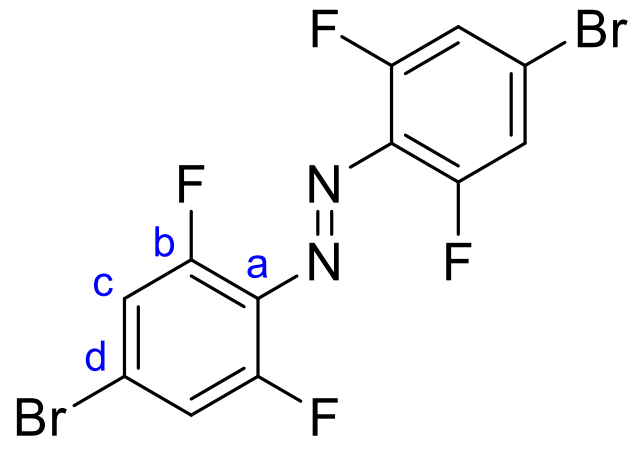

The title compound was synthesized following a literature procedure.[4] Spectral data was identical to that previously reported.[4]

^{1}H NMR (400 MHz, DMSO-d_6) δ 7.78 (d, *J* = 8.9 Hz, 4H, H^c).

2.3 Synthesis and characterization of intermediate photoswitch S2

2.3.1 Synthesis of intermediate photoswitch S2

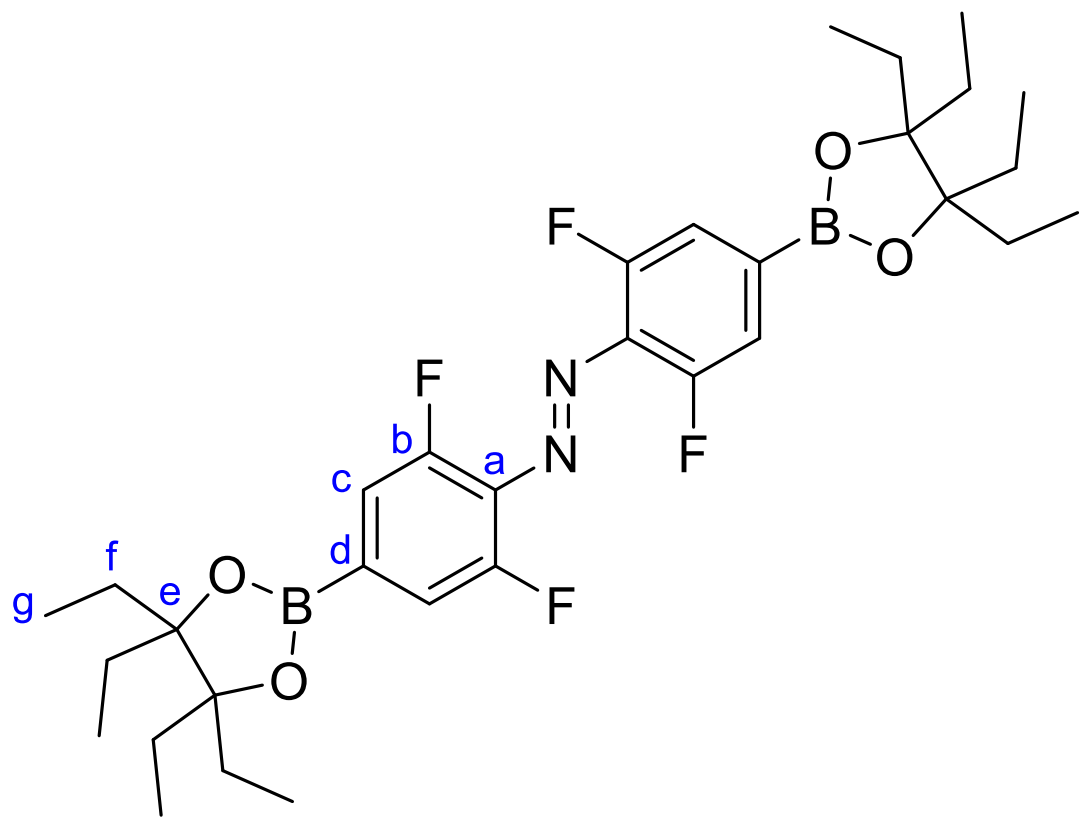

The title compound was synthesized following modified literature procedures.[4-5]

1,2-Bis(4-bromo-2,6-difluorophenyl)diazene (**S1**, 260 mg, 631 µmol, 1.00 equiv.), $B_2(Epin)_2$ (647 mg, 1.77 mmol, 2.80 equiv.) and potassium acetate (372 mg, 3.79 mmol, 6.00 equiv.) were mixed with dry toluene (4.5 mL) in a 2-5 mL microwave vial. The red mixture was degassed with argon for 20 minutes before $Pd(dppf)Cl_2$ (46 mg, 63 µmol, 0.10 equiv.) was added. The mixture was again degassed with argon for 5 minutes and then placed in the microwave reactor at 150 °C for 20 minutes. The reaction mixture was cooled to room temperature, filtered over Celite and washed with EtOAc (30 mL). The filtrate was washed with water (30 mL) and brine (30 mL). The organic layer was dried over $MgSO_4$, and the solvent was removed *in vacuo.* The residue was purified by column chromatography (120 g self-packed silica gel column) using EtOAc in hexane (0-1% gradient) to give the product as a red oil, which crystallized when left to stand at room temperature. The product was dissolved in DCM:MeCN (1:5) and was left to crystallize overnight. The crystals were collected by filtration and dried *in vacuo* to give the title compound as red crystals (165 mg, 267 µmol, 42%).

***E* isomer**

^{1}H NMR (600 MHz, $CDCl_3$) δ 7.46 (d, *J* = 8.9 Hz, 2H, H^c), 1.76 (m, 8H, H^f), 0.97 (t, *J* = 7.5 Hz, 12H, H^g).

^{19}F NMR (565 MHz, $CDCl_3$) δ -122.47 (d, *J* = 9.6 Hz, F^b).

^{13}C NMR (151 MHz, $CDCl_3$) δ 155.1 (dd, *J* = 262, 3 Hz, C^b), 133.4 (t, *J* = 9.8 Hz, C^a), 118.3 (dd, *J* = 18.4, 3.4 Hz, C^k), 89.9 (t, *J* = 5.98 Hz, C^d), 89.7 (C^e), 26.6 (C^f), 8.9 (C^g).

ESI-MS (m/z): 619.3453 $[M+H]^+$ (calculated for $C_{32}H_{45}B_2F_4N_2O_4^+$ 619.3496)

2.3.2 1D and 2D NMR characterization of intermediate photoswitch S2

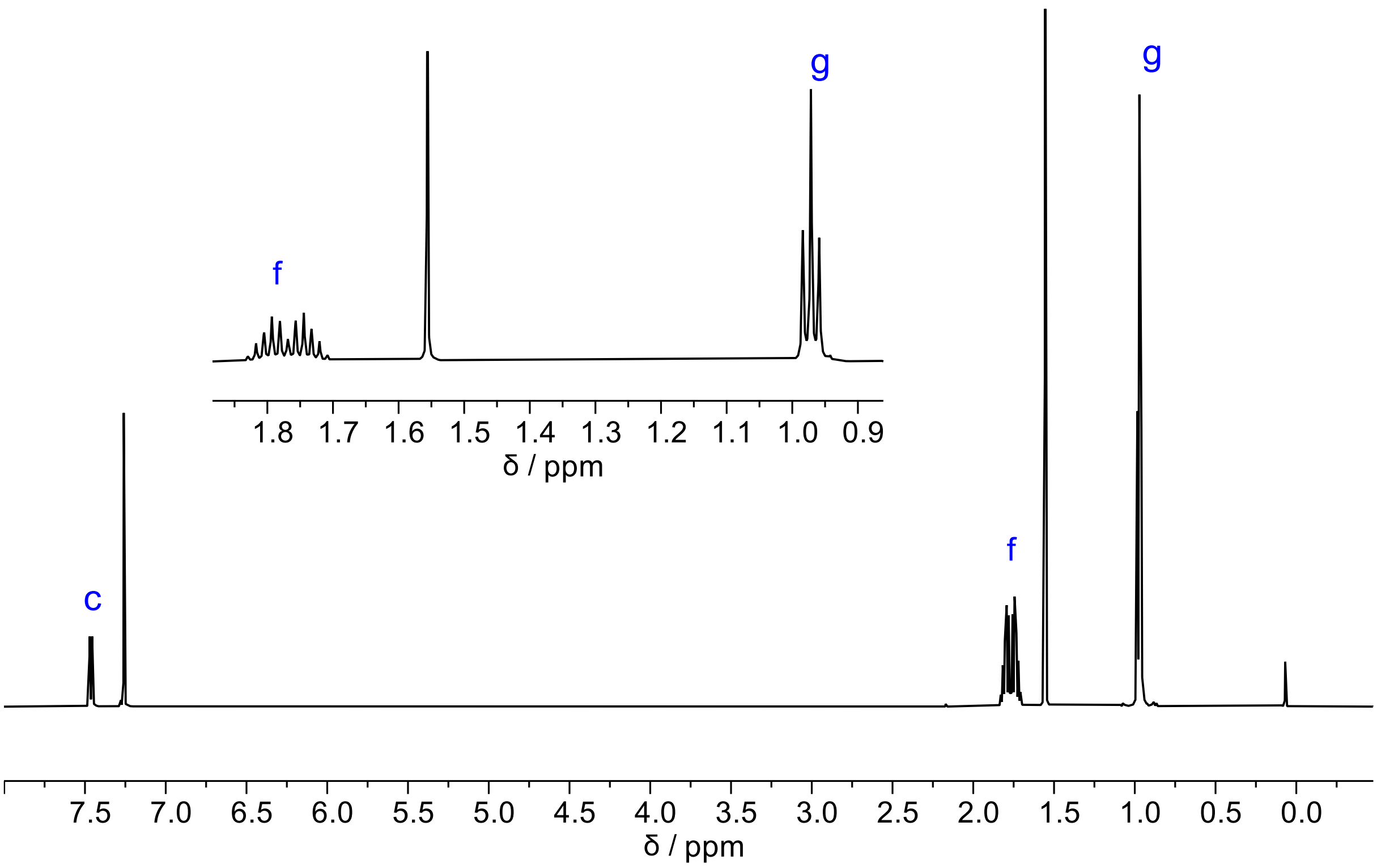

Figure S1. ^{1}H NMR (400 MHz, 298 K, $CDCl_3$) spectrum of intermediate **S2**.

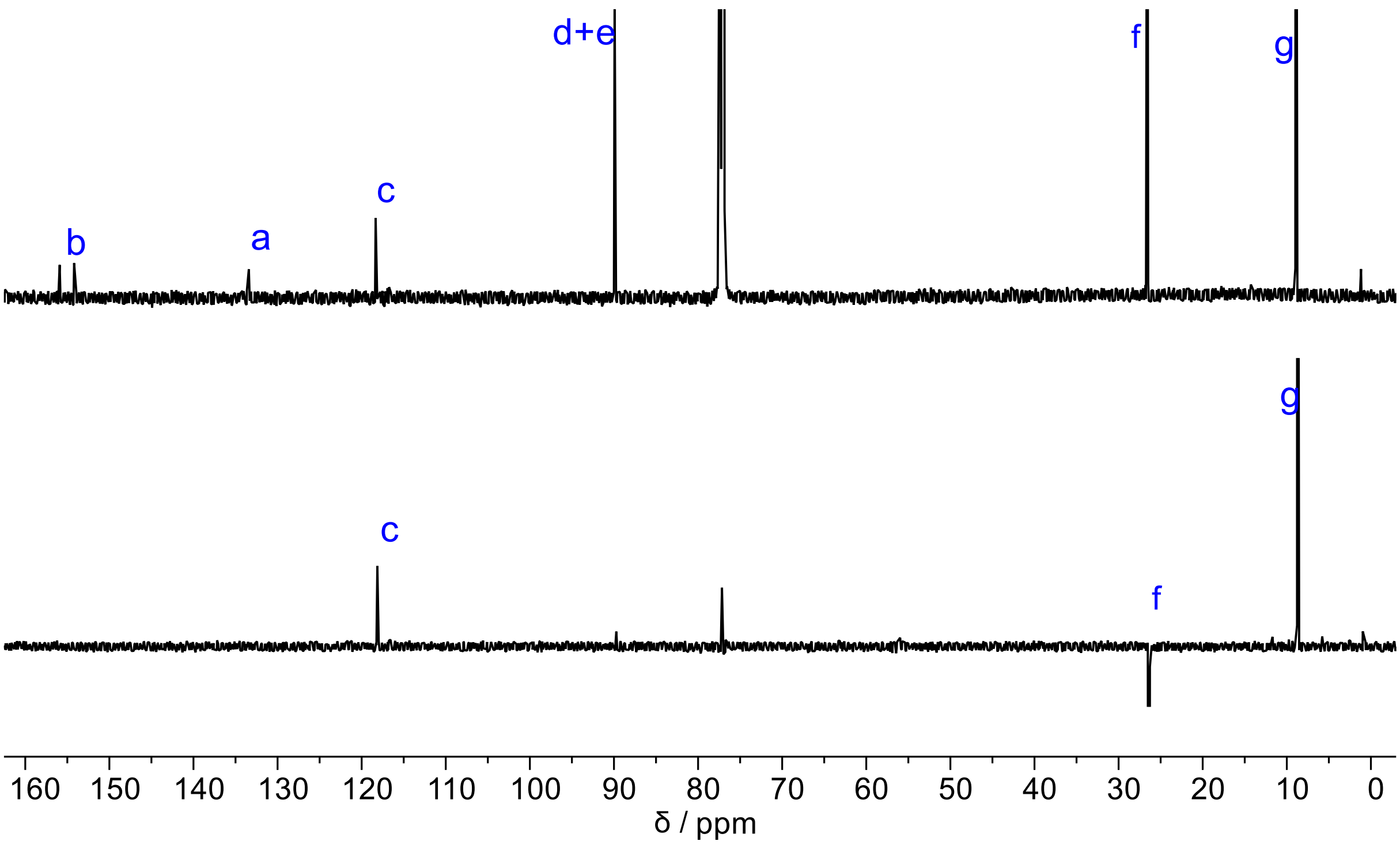

Figure S2 ^{13}C{^{1}H} and DEPT135 NMR (151 MHz, 298 K, $CDCl_3$) spectrum of intermediate **S2**.

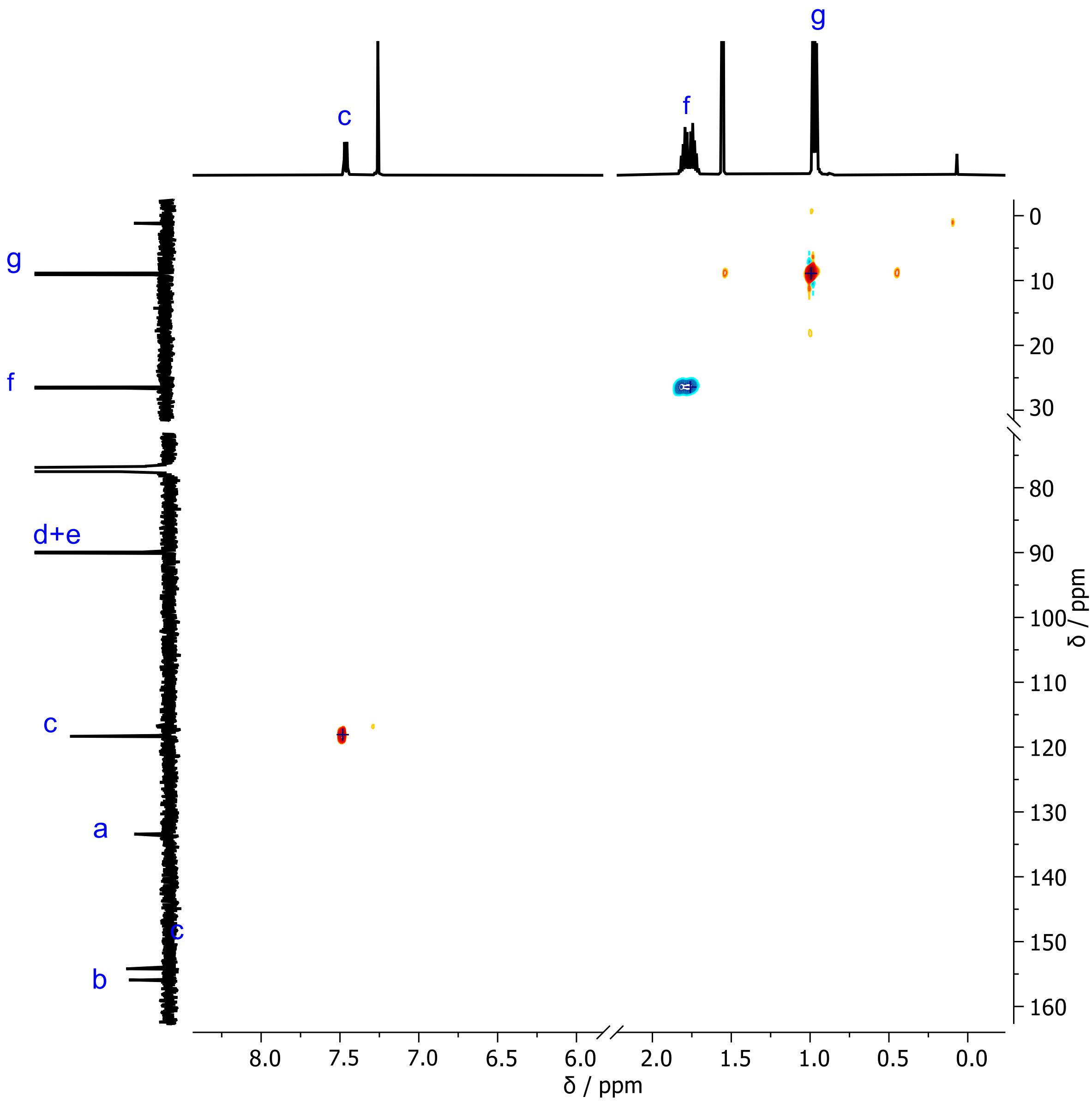

Figure S3 ^{1}H-^{13}C HSQC NMR (500 MHz, 126 MHz, 298 K, $CDCl_3$) spectrum of intermediate **S2**.

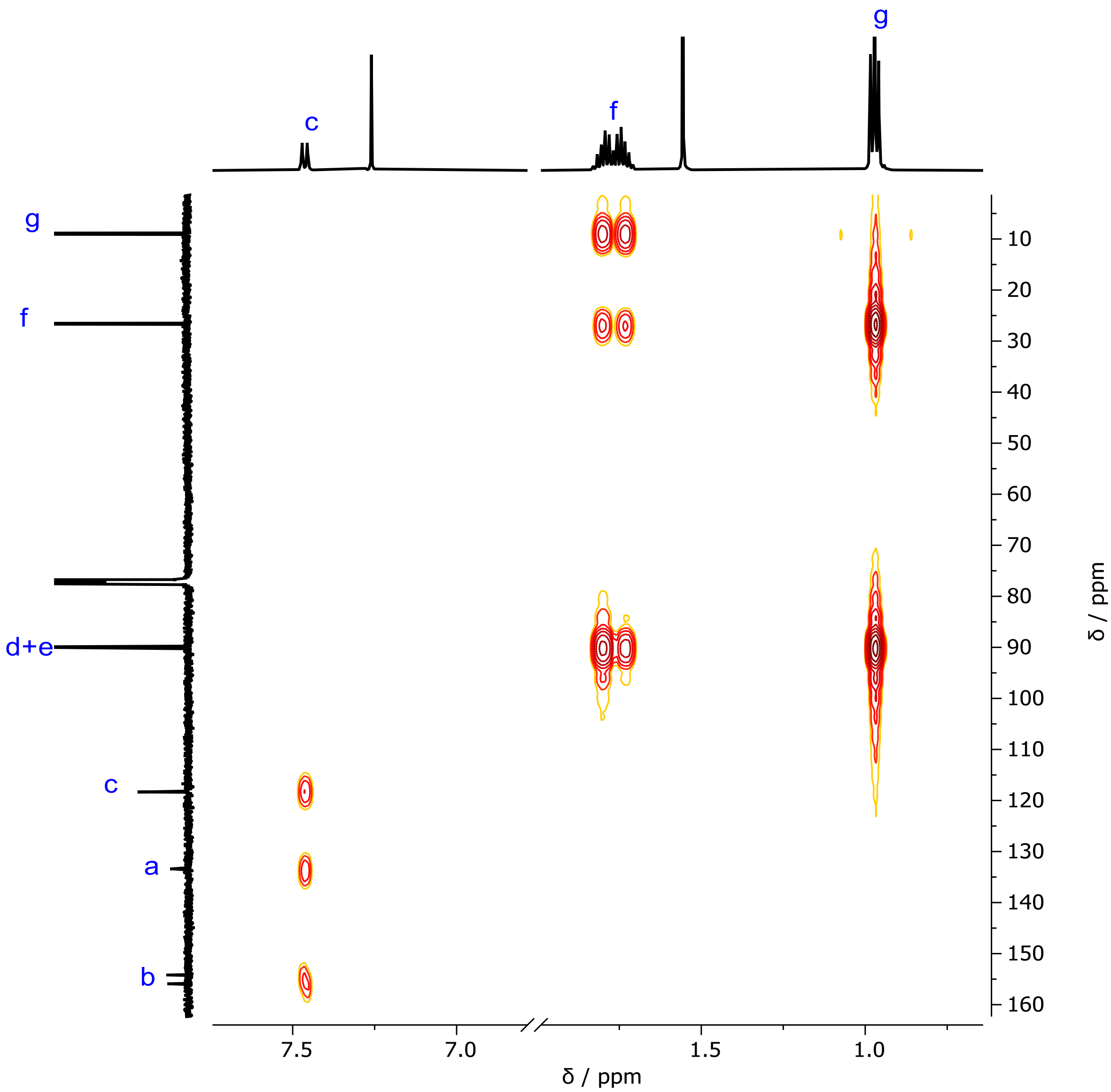

Figure S4 ^{1}H-^{13}C HMBC NMR (600 MHz, 151 MHz, 298 K, $CDCl_3$) spectrum of intermediate **S2**.

2.4 Synthesis and characterization of photoswitch 3

2.4.1 Synthesis of photoswitch 3

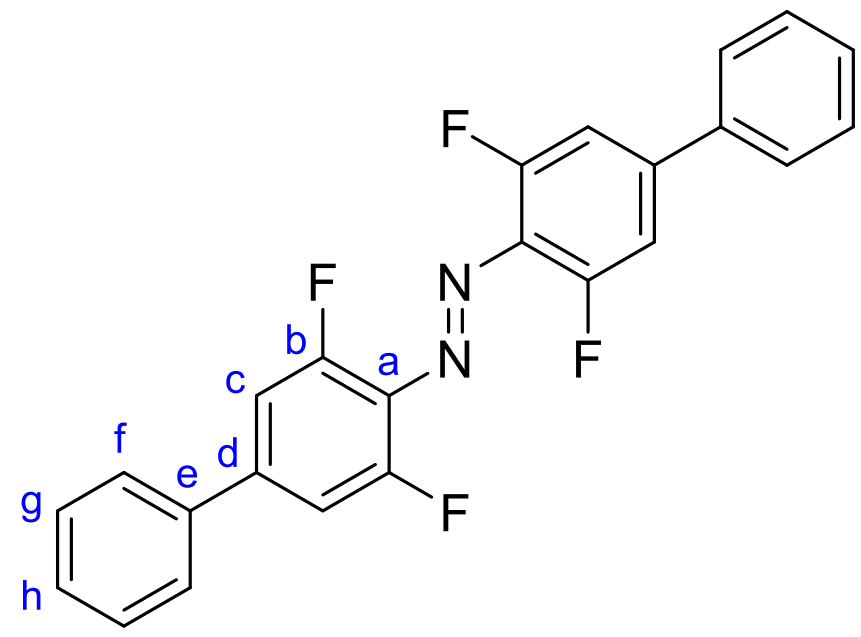

Photoswitch **3** was synthesized following a modified literature procedure.[4]

Intermediate **S2** (100 mg, 162 µmol, 1.00 equiv.) and iodobenzene (110 µL, 970 µmol, 6.00 equiv.) were mixed at room temperature in a 2-5 mL microwave vial with dioxane (3.0 mL). Then K_2CO_3 (55.9 mg, 404 µmol, 2.50 equiv.) in H_2O (0.6 mL) was added. The red mixture was degassed with argon for 20 minutes before Pd(dppf)Cl_2 (12 mg, 16 µmol, 0.10 equiv.) was added. The mixture was degassed for a further 5 minutes and then placed in the microwave at 130 °C for 20 minutes. The reaction mixture was allowed to cool to room temperature, diluted with DCM (20 mL), and filtered over Celite. The organic phase was washed with water (2 × 25 mL) and brine (25 mL). The organic layer was dried over $MgSO_4$, and the solvent was removed *in vacuo*. The residue was purified by column chromatography (40 g, self-packed silica gel column) using EtOAc in hexane (0-10% gradient) to yield the title compound as red crystals (51.7 mg, 127 µmol, 79%).

Spectral data were identical to that previously reported.[4]

^{1}H NMR (400 MHz, DMSO-d_6) δ 7.91 – 7.85 (m, 4H, H^f), 7.76 (dd, *J* = 11.0, 1.3 Hz, 4H, H^c), 7.57 – 7.46 (m, 6H, H^{g+h}).

^{19}F NMR (376 MHz, DMSO-d_6) δ -120.31 (d, *J* = 11.7 Hz, F^b).

^{13}C NMR (101 MHz, DMSO-d_6) δ 155.3 (dd, *J* = 259.1, 5.3 Hz, C^b), 144.8 (t, *J* = 10.3 Hz, C^d), 136.6 (C^e), 129.6 (C^{a+h}), 129.2 (C^g), 127.1 (C^f), 111.0 (dd, *J* = 21.5, 2.3 Hz, C^c).

ESI-MS (m/z): 407.1139 $[M+H]^+$ (calculated for $C_{24}H_{15}F_4N_2^+$ 407.1166)

2.4.2 1D NMR characterization of photoswitch 3 in DMSO-d_6

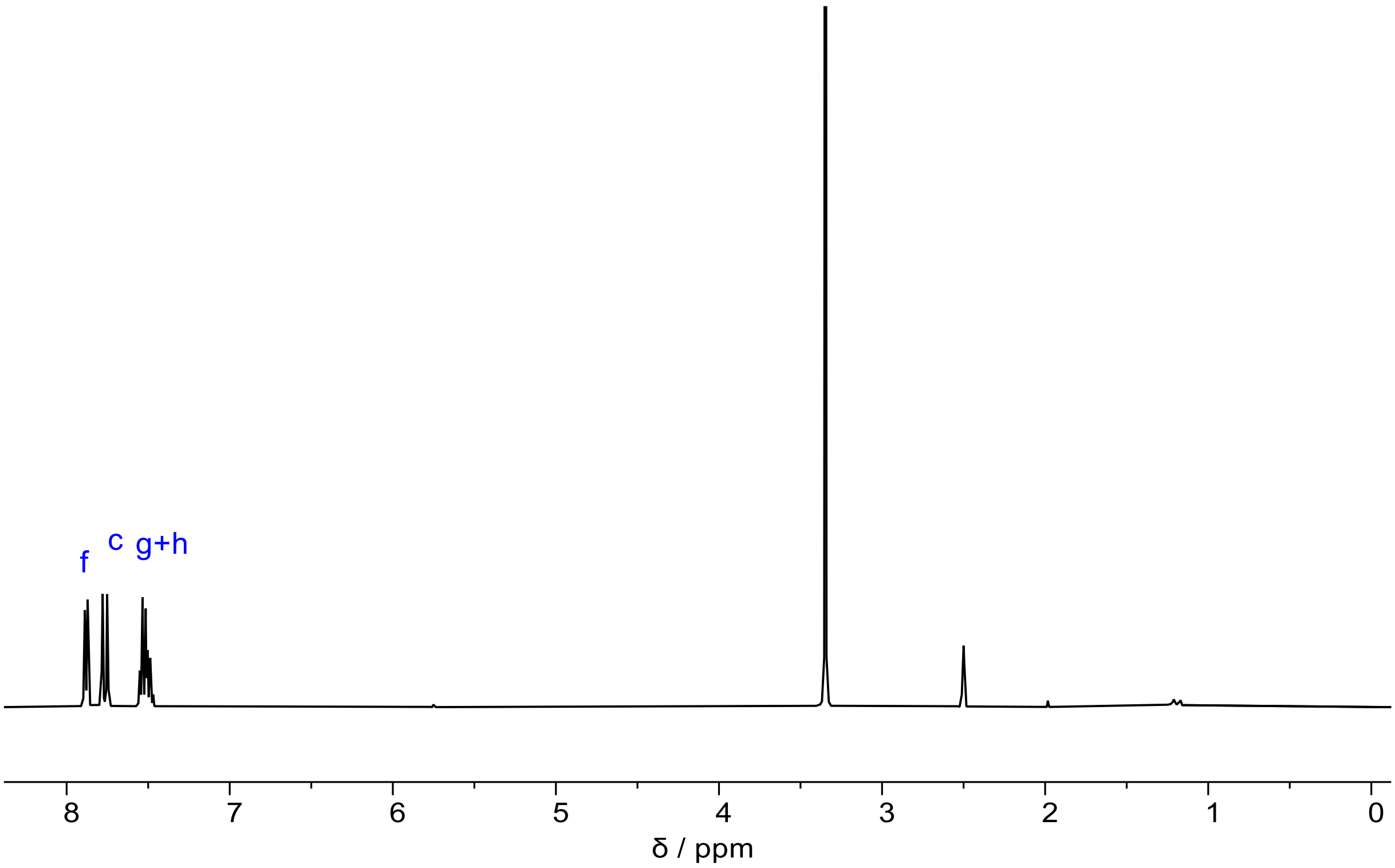

Figure S5. ^{1}H NMR (400 MHz, 298 K, DMSO-d_6) spectrum of photoswitch **3**.

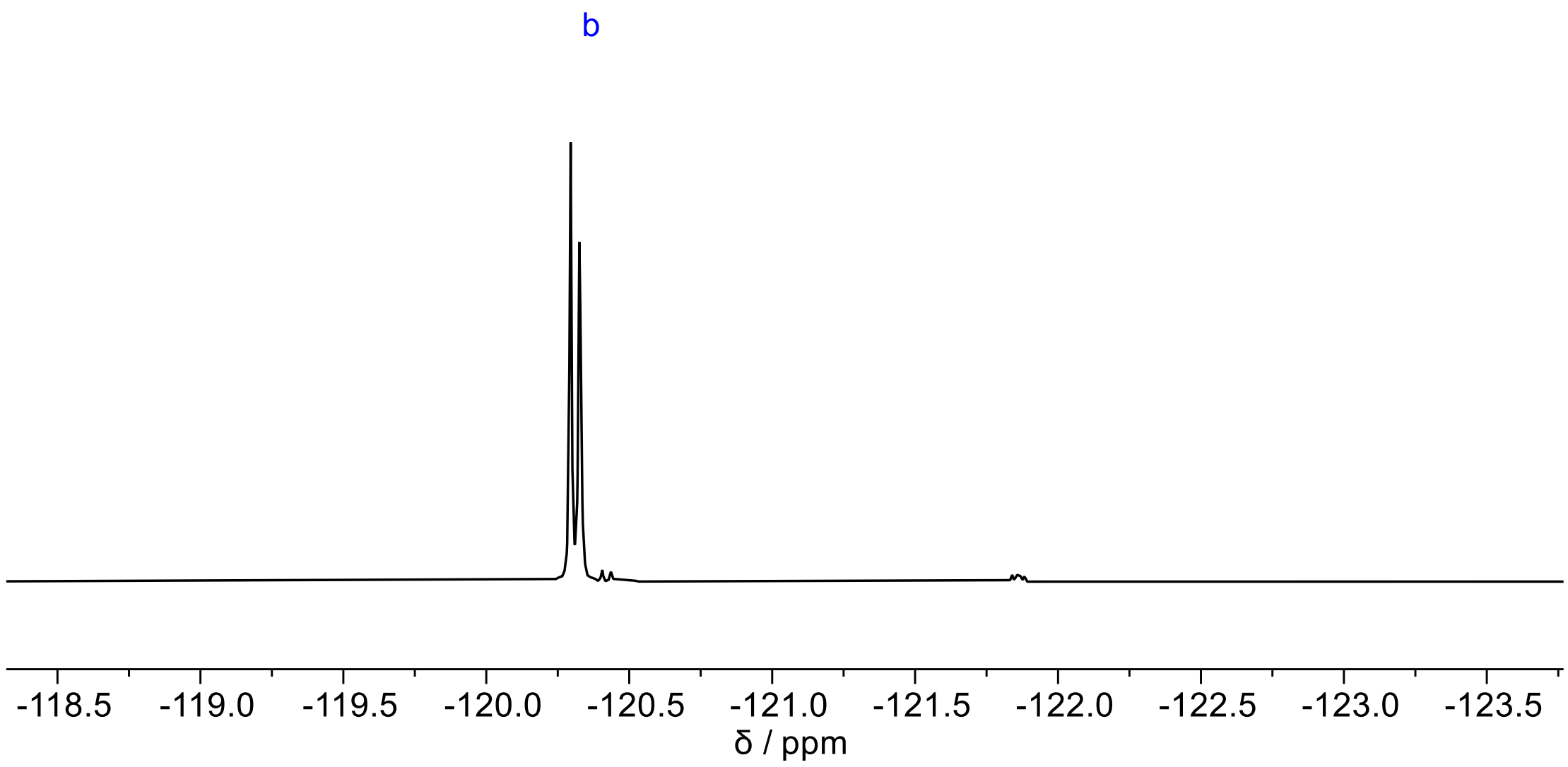

Figure S6. ^{19}F NMR (376 MHz, 298 K, DMSO-d_6) spectrum of photoswitch **3**.

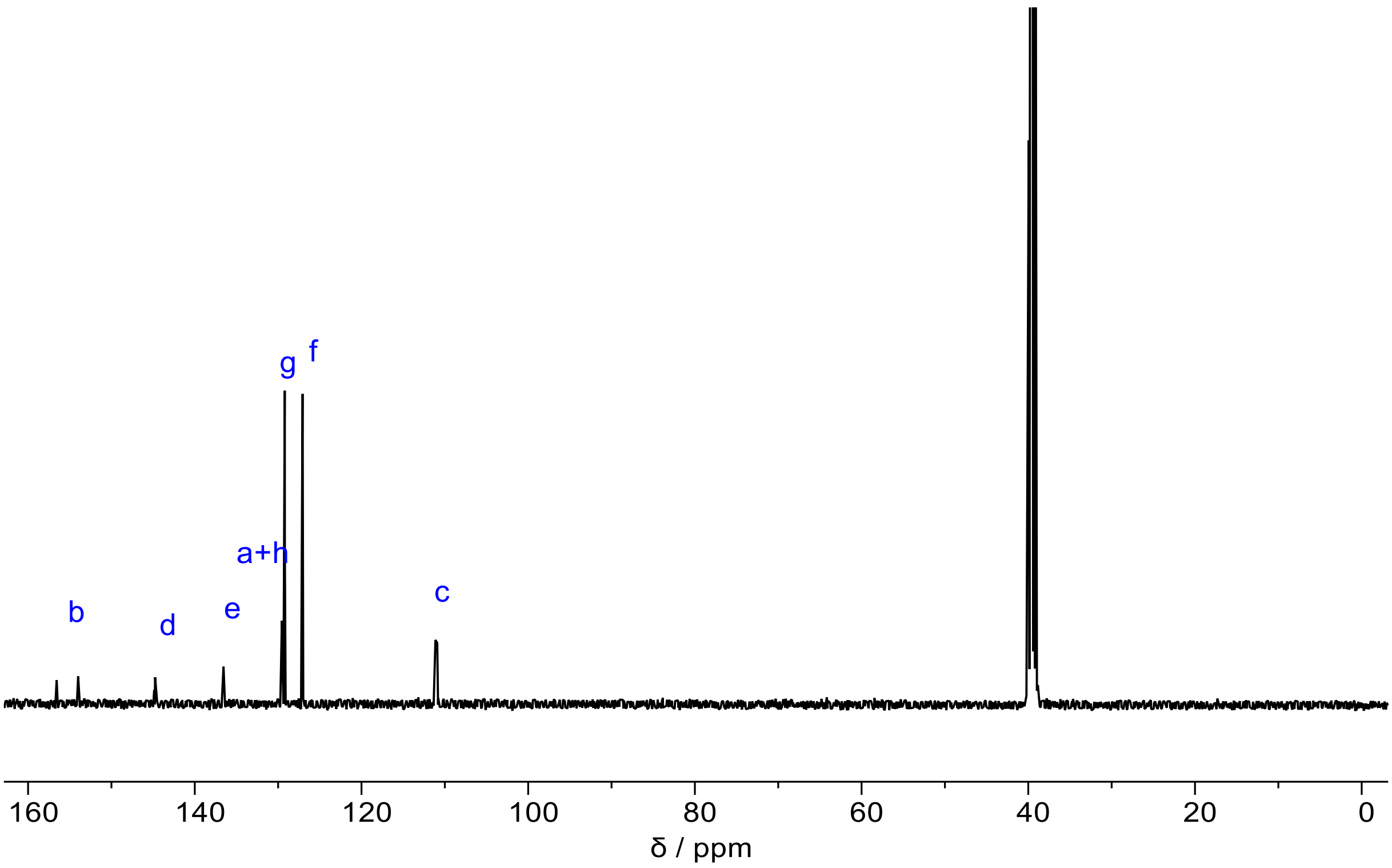

Figure S7. $^{13}C\{^{1}H\}$ NMR (101 MHz, 298 K, DMSO-d_6) spectrum of photoswitch **3**.

2.5 Synthesis and characterization of photoswitch 4

2.5.1 Synthesis of photoswitch 4

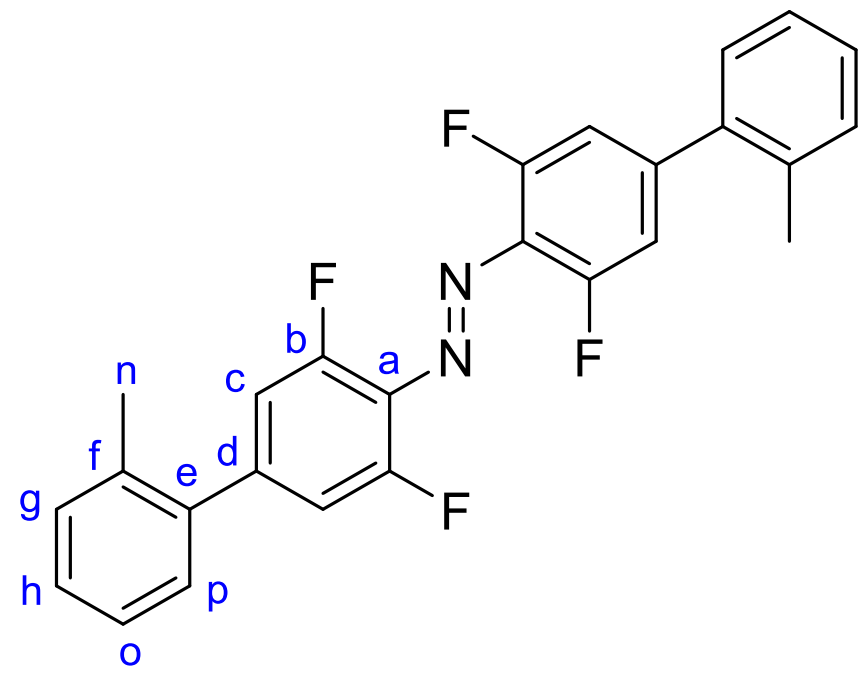

Intermediate **S2** (80.0 mg, 129 µmol, 1.00 equiv.) and 1-bromo-2-methylbenzene (133 mg, 776 µmol, 6.00 equiv.) were mixed with dioxane (3 mL). A solution of potassium carbonate (44.7 mg, 323 µmol, 2.50 equiv.) in water (0.6 mL) was added. The red suspension was degassed with argon for 20 minutes. Pd(dppf)Cl_2 (3.1 mg, 4.3 µmol, 0.03 equiv.) was added, and the reaction mixture was degassed again with argon for 20 minutes. The reaction mixture was then placed in the microwave reactor at 130 °C for 20 minutes to give a dark mixture. After cooling to room temperature, the reaction mixture was filtered through Celite and washed with DCM (50 mL). The filtrate was washed with water (2 × 40 mL) and brine (40 mL). The organic phase was dried over $MgSO_4$ and the solvent was removed *in vacuo*. The residue was purified by column chromatography (25 g, pre-packed silica gel column) using EtOAc in hexane (0-5% gradient) to give the product as a red oil, which crystallized when left to stand at room temperature. The product was dissolved in DCM:MeCN (1:5) and was left to crystallize overnight. The crystals were collected and dried *in vacuo* to give the title compound as red crystals (41.6 mg, 95.8 µmol, 74%).

^{1}H NMR (400 MHz, DMSO-d_6) δ 7.46 – 7.37 (m, 4H, H^{c}), 7.38 – 7.29 (m, 8H, $H^{g+h+o+p}$), 2.34 (s, 6H, H^{n}).

^{19}F NMR (565 MHz, DMSO-d_6) δ -121.60 (d, *J* = 11.0 Hz, F^{b}).

^{13}C NMR (101 MHz, DMSO-d_6) δ 154.4 (dd, *J* = 259.6, 5.3 Hz, C^{b}), 146.4 (t, *J* = 10.3 Hz, C^{d}), 138.3 (C^{f}), 135.0 (C^{g}), 130.7 (C^{h}), 129.43 (t, *J* = 10.0 Hz, C^{a}), 129.39 (C^{p}), 128.7 (C^{e}), 126.2 (C^{o}), 113.9 (dd, *J* = 20.6, 3.0 Hz, C^{c}), 20.0 (C^{n}).

ESI-MS (m/z): 435.1456 $[M+H]^+$ (calculated for $C_{26}H_{19}F_4N_2^+$ 435.1479)

2.5.2 1D and 2D NMR characterization of photoswitch 4

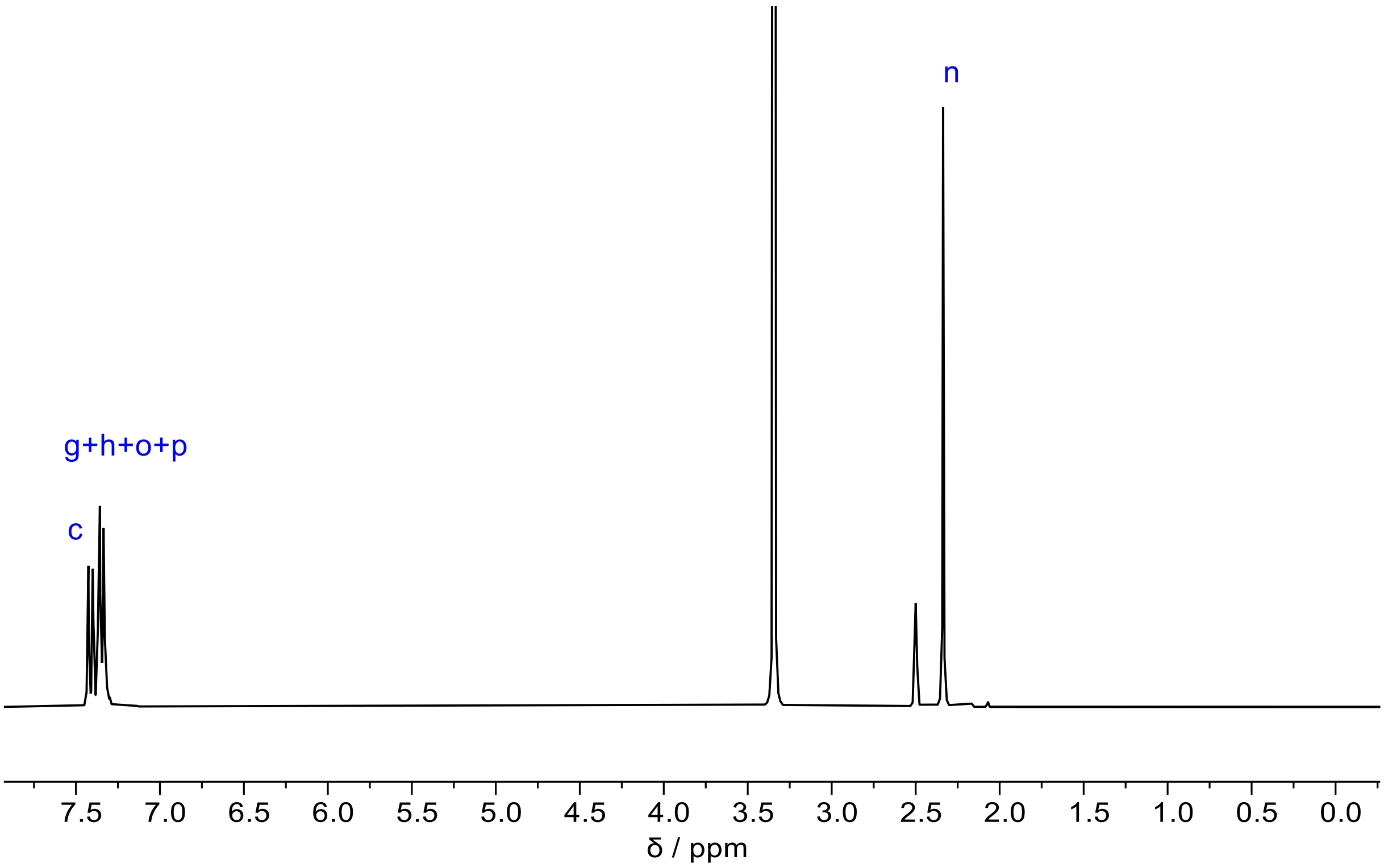

Figure S8. ^{1}H NMR (400 MHz, 298 K, DMSO-d_6) spectrum of photoswitch **4**.

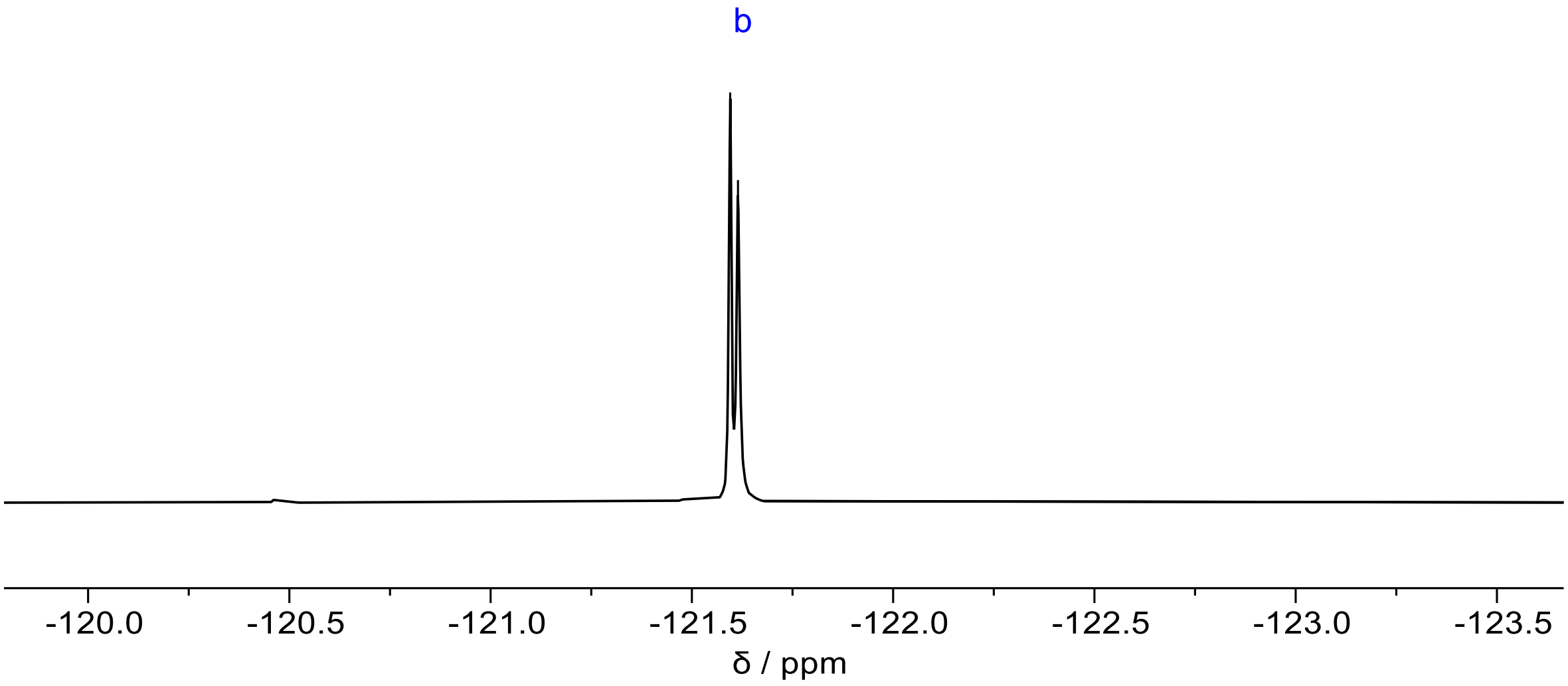

Figure S9. ^{19}F NMR (565 MHz, 298 K, DMSO-d_6) spectrum of photoswitch **4**.

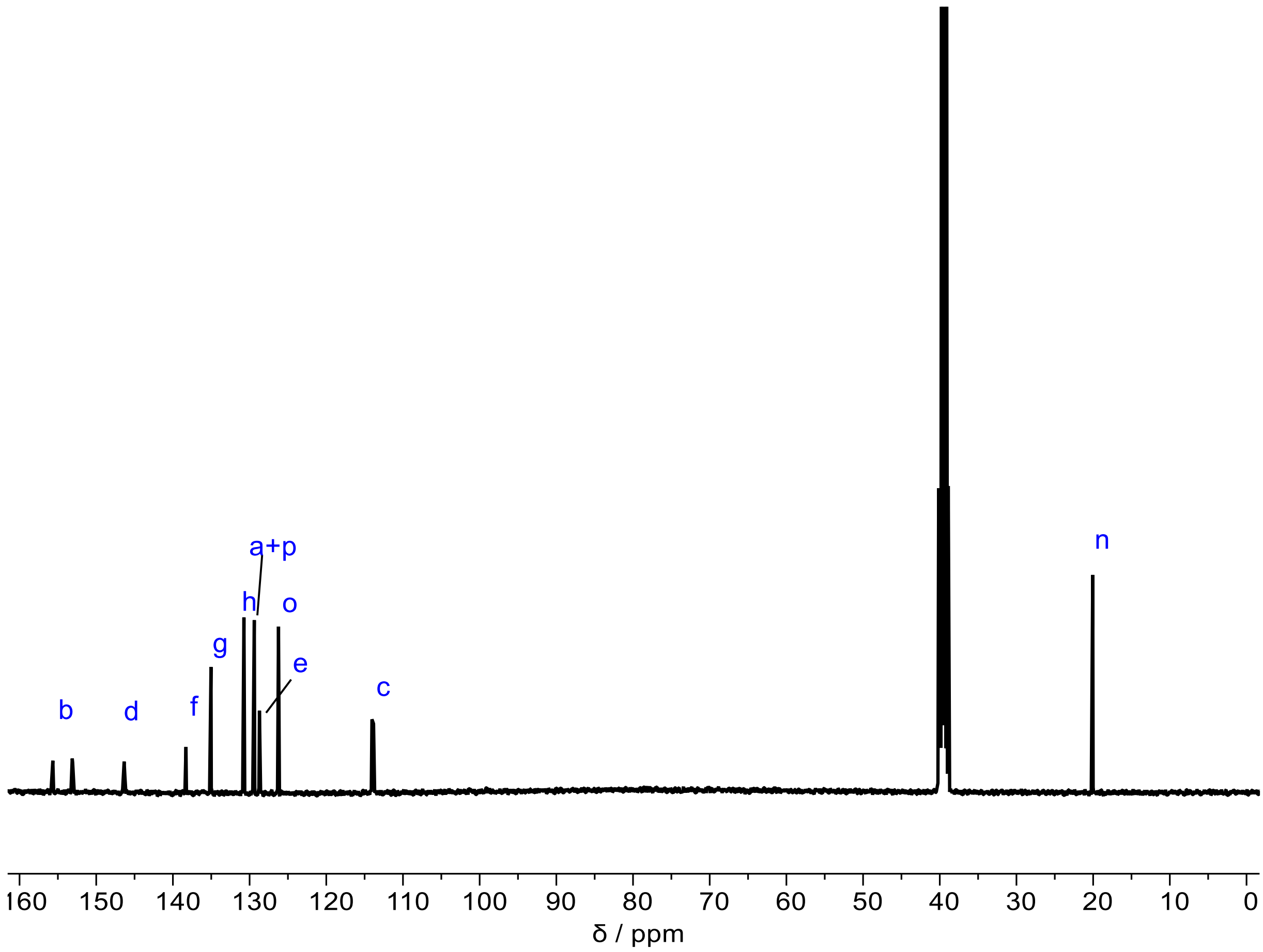

Figure S10. $^{13}C\{^{1}H\}$ NMR (101 MHz, 298 K, DMSO-d_6) spectrum of photoswitch **4**.

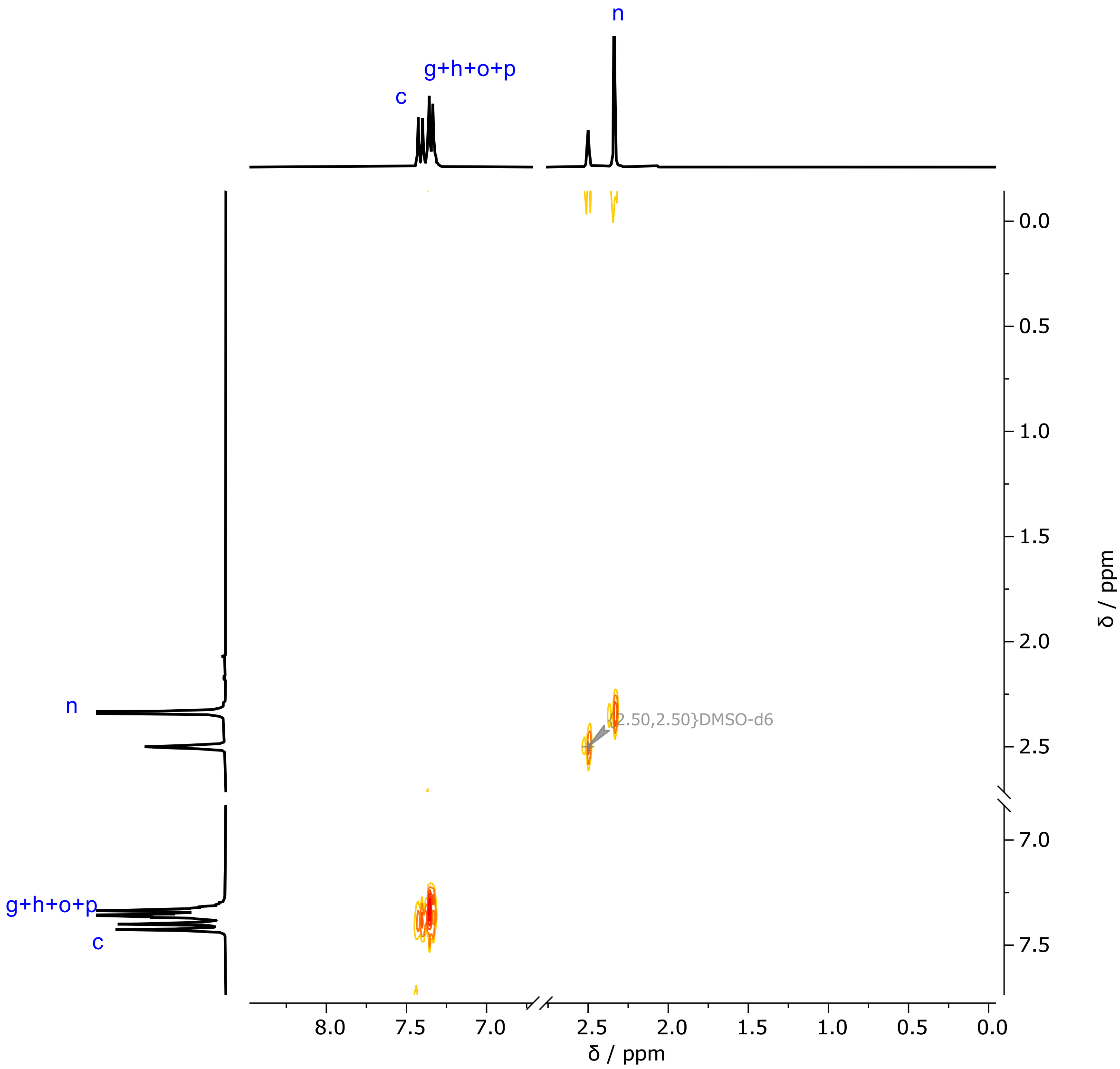

Figure S11. ^{1}H-^{1}H COSY NMR (400 MHz, 101 MHz, 298 K, DMSO-*d*$_6$) spectrum of photoswitch **4**.

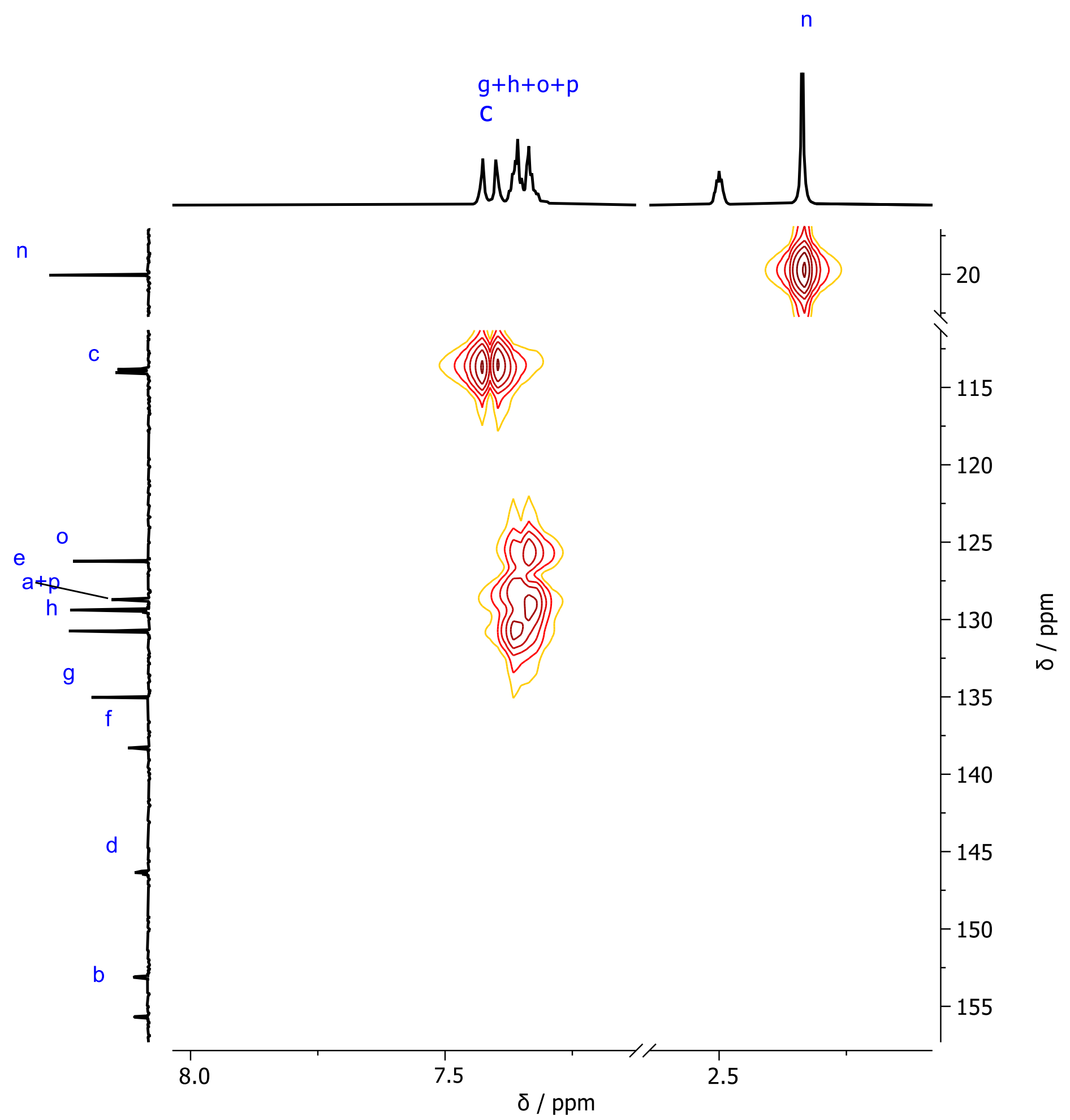

Figure S12. ^{1}H-^{13}C HSQC NMR (400 MHz, 101 MHz, 298 K, DMSO-d_6) spectrum of photoswitch **4**.

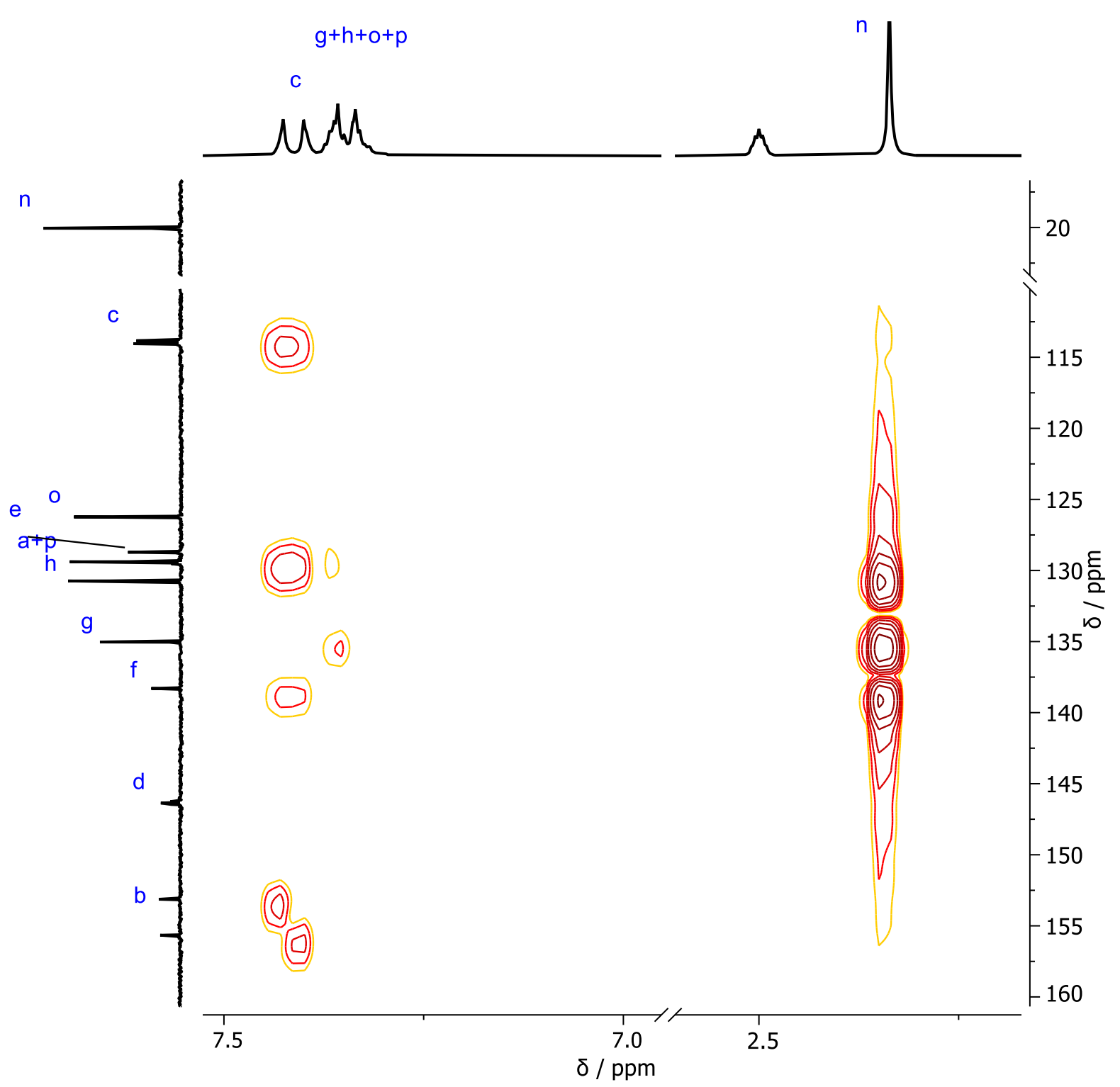

Figure S13. ^{1}H-^{13}C HMBC NMR (400 MHz, 101 MHz, 298 K, DMSO-d_6) spectrum of photoswitch **4**.

2.6 Synthesis and characterization of photoswitch 5

2.6.1 Synthesis of photoswitch 5

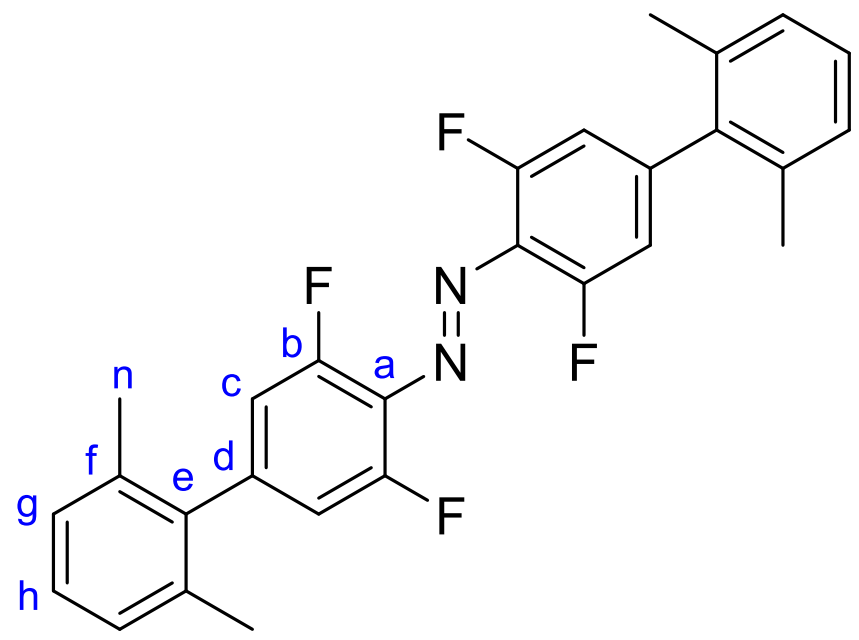

Intermediate **S2** (120 mg, 194 µmol, 1.00 equiv.) and 2-bromo-1,3-dimethylbenzene (215 mg, 1.16 mmol, 6.00 equiv.) were placed in dioxane (4 mL). Then a solution of potassium carbonate (67.0 mg, 48.5 µmol, 2.50 equiv.) in water (0.8 mL) was added. The red suspension was degassed with argon for 20 minutes. Pd(dppf)Cl_2 (4.7 mg, 6.4 µmol, 0.03 equiv.) was added, and the suspension was degassed again with argon for 5 minutes. The reaction mixture was placed in the microwave reactor at 130 °C for 20 minutes to give a dark mixture. After cooling to room temperature, the reaction mixture was filtered through Celite and washed with DCM (50 mL). The filtrate was washed with water (2 × 40 mL) and brine (40 mL). The organic phase was dried over $MgSO_4$ and the solvent was removed *in vacuo*. The residue was purified by column chromatography (25 g, pre-packed silica gel column) using EtOAc in hexane (0-1% gradient) to give the product as a red oil, which crystallized when left to stand at room temperature. The product was dissolved in DCM:MeCN (1:5) and was left to crystallize overnight. The crystals were collected and dried *in vacuo* to give the title compound as red crystals (30.2 mg, 65.3 µmol, 34%).

^{1}H NMR (400 MHz, DMSO-d_6) δ 7.29 – 7.21 (m, 6H, H^{c+h}), 7.17 (dq, J = 7.1, 0.8 Hz, 4H, H^{g}), 2.08 (s, 12H, H^{n}).

^{19}F NMR (565 MHz, DMSO-d_6) δ -121.68 (d, J = 10.6 Hz, F^{b}).

^{13}C NMR (101 MHz, DMSO-d_6) δ 154.7 (dd, J = 259.9, 5.0 Hz, C^{b}), 146.0 (t, J = 10.4 Hz, C^{d}), 138.6 (C^{e}), 135.1 (C^{f}), 129.5 (t, J = 9.9 Hz, C^{a}), 128.1 (C^{h}), 127.6 (C^{g}), 114.2 (dd, J = 20.0, 2.9 Hz, C^{c}), 20.3 (C^{n}).

ESI-MS (m/z): 463.1791 $[M+H]^+$ (calculated for $C_{28}H_{23}F_4N_2^+$ 463.1792)

2.6.2 1D and 2D NMR characterization of photoswitch 5

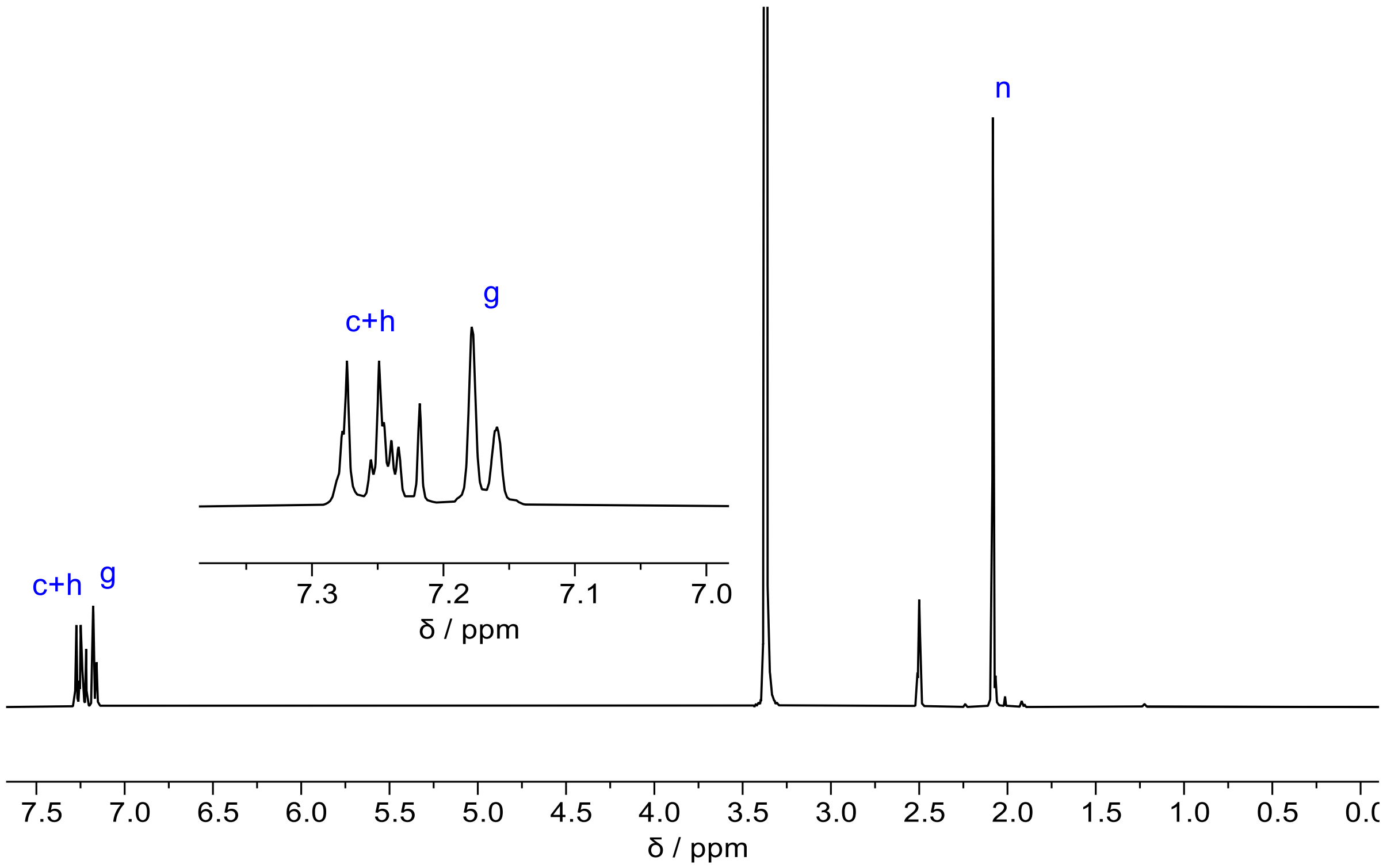

Figure S14. ^{1}H NMR (400 MHz, 298 K, DMSO-d_6) spectrum of photoswitch **5**.

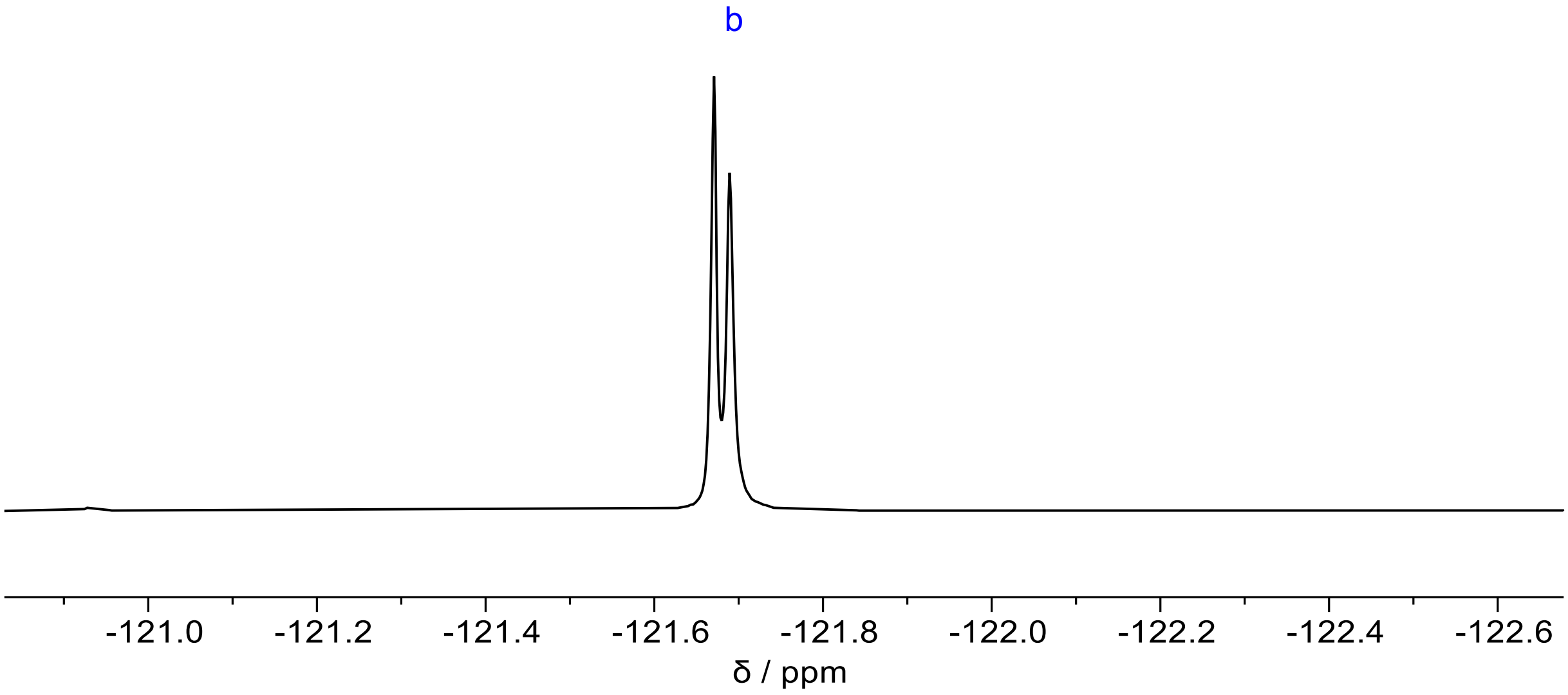

Figure S15. ^{19}F NMR (565 MHz, 298 K, DMSO-d_6) spectrum of photoswitch **5**.

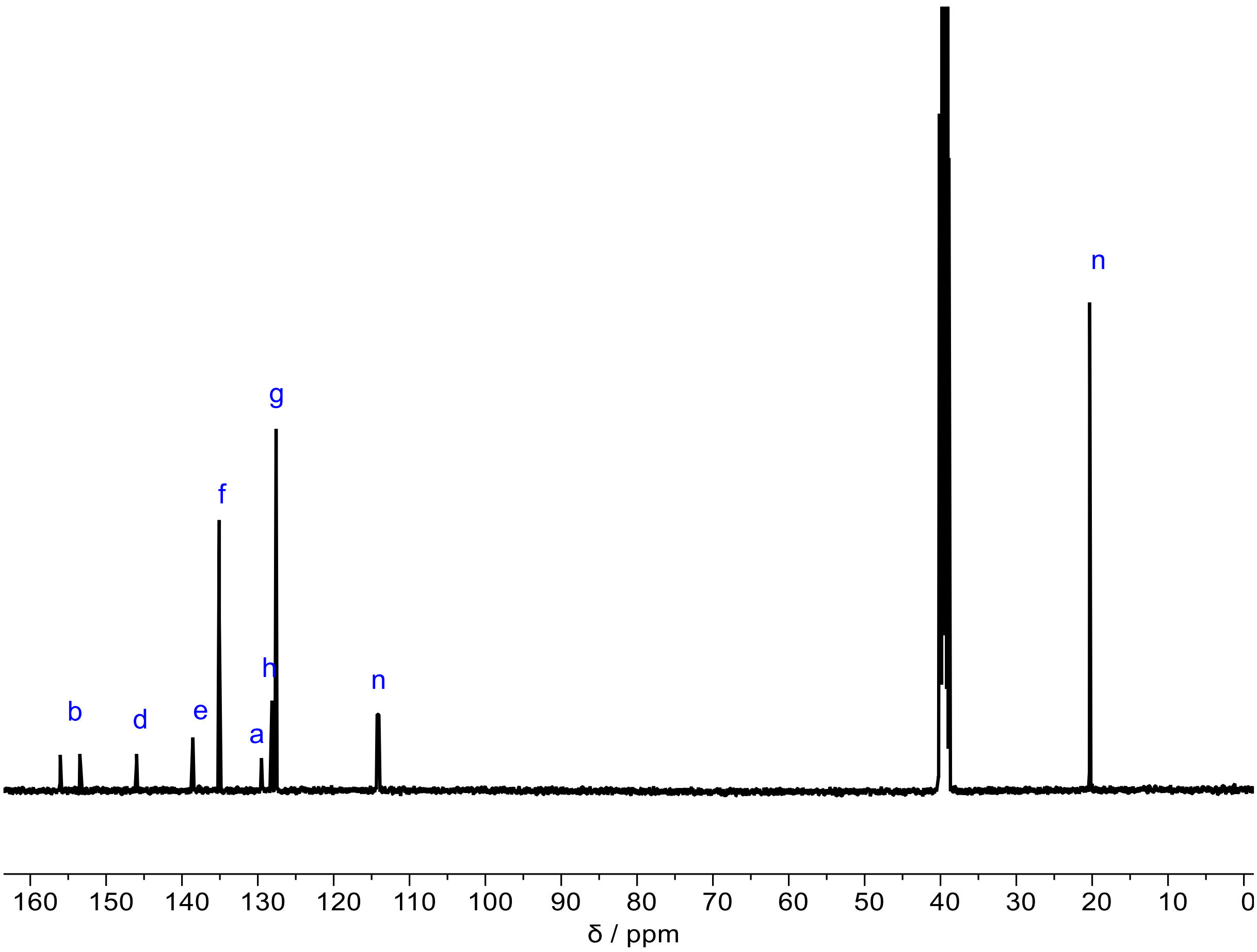

Figure S16. $^{13}C\{^1H\}$ NMR (101 MHz, 298 K, DMSO-d_6) spectrum of photoswitch **5**.

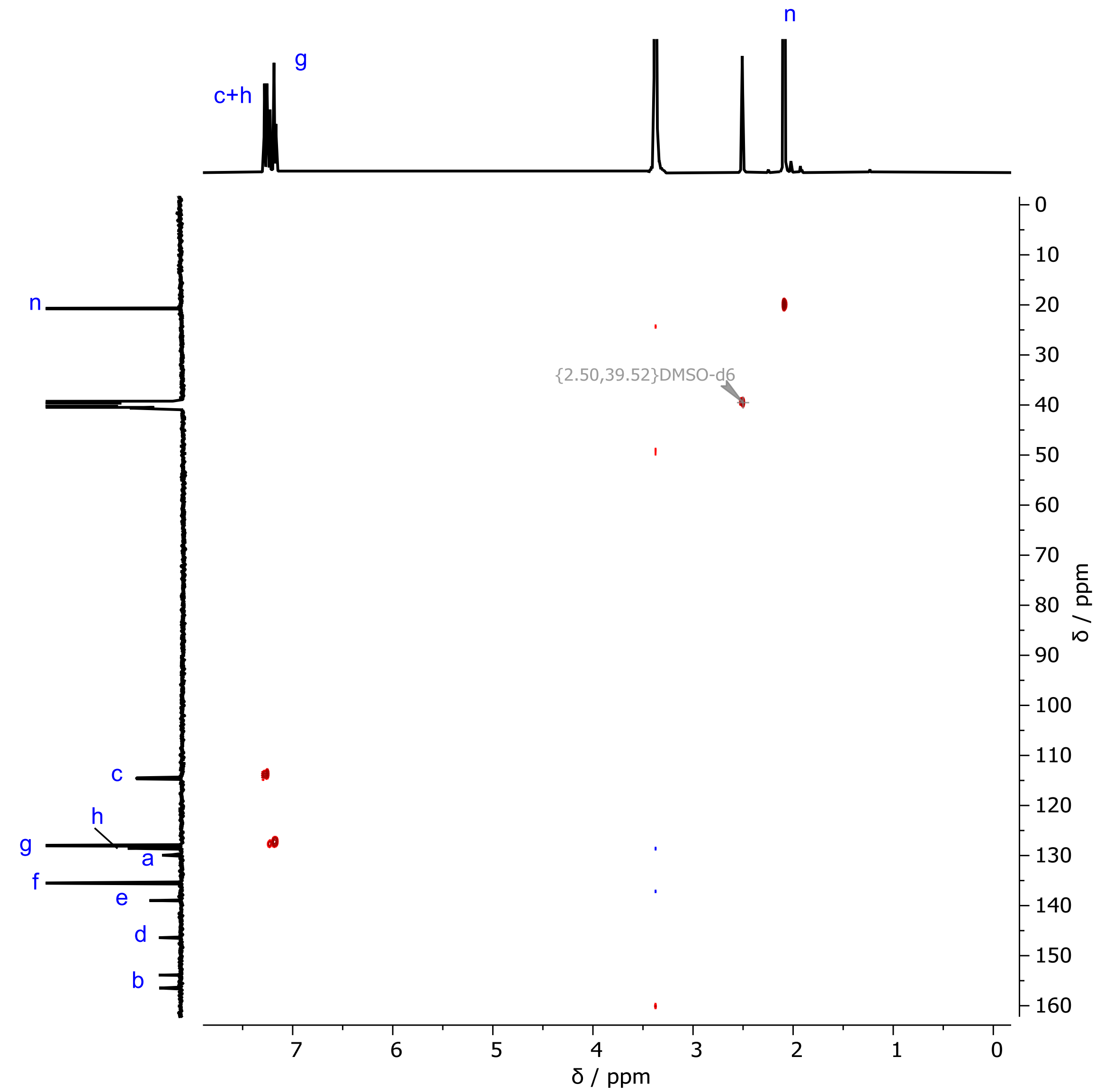

Figure S17. ^{1}H-^{13}C HSQC NMR (400 MHz, 101 MHz, 298 K, DMSO-d_6) spectrum of photoswitch **5**.

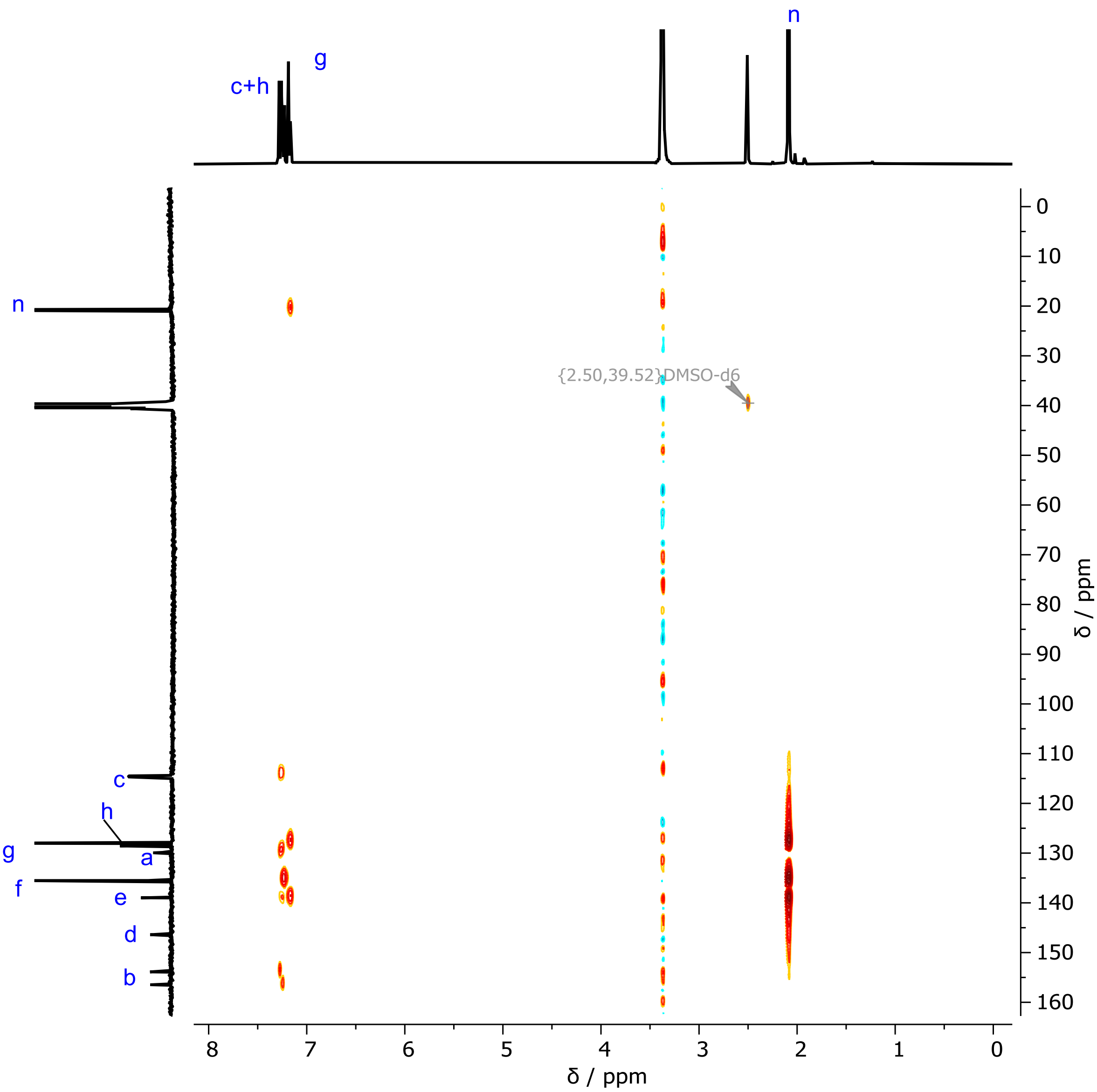

Figure S18. ^{1}H-^{13}C HMBC NMR (400 MHz, 101 MHz, 298 K, DMSO-d_6) spectrum of photoswitch **5.**

3 Synthesis of photoswitchable ligand 1

3.1 Synthetic overview

Ligand **1** was synthesized in four steps using modified literature procedures[6] (Scheme S2). Starting from the commercially available 2,6-dimethylphenol, *N*-bromosuccinimide (NBS) was used to introduce a bromine in the *para*-position of the phenol yielding 4-bromo-2,6-dimethylphenol (**S3**) in 91% yield. Subsequent Suzuki-Miyaura coupling with 3-pyridilboronic acid pinacol ester afforded 2,6-dimethyl-4-(pyridin-3-yl)phenol (**S4**) in 86% yield. Introduction of the triflate leaving group using trifluoromethanesulfonic anhydride led to intermediate **S5** in 72% yield. Finally, Suzuki-Miyaura coupling between intermediate **S5** and the $B_2(Epin)_2$ intermediate **S2** yielded the title compound ligand **1** in 45% yield.

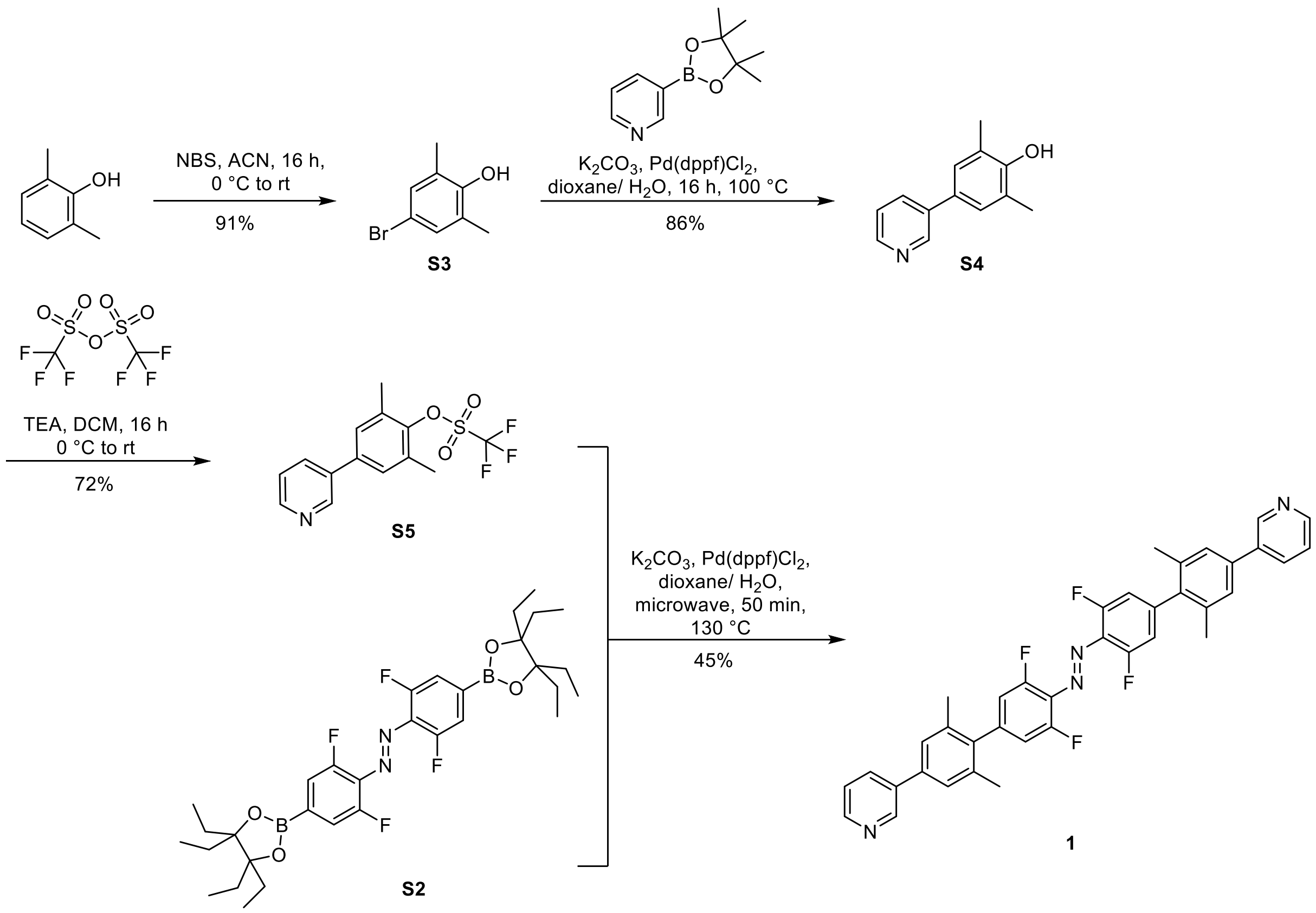

Scheme S2. Synthetic route to the *ortho*-methylated ligand **1**.

3.2 Synthesis and characterization of 4-bromo-2,6-dimethylphenol (S3)

3.2.1 Synthesis of 4-bromo-2,6-dimethylphenol (S3)

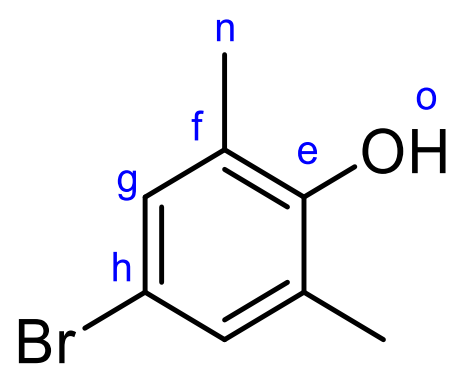

The title compound was synthesized following a literature procedure.[6]

2,6-Dimethylphenol (1.22 g, 10.0 mmol, 1.00 equiv.) was dissolved in acetonitrile (10 mL) and stirred in an ice bath. Then, NBS (1.80 g, 10.0 mmol, 1.00 equiv.) in acetonitrile (10 mL) was added slowly. The mixture was warmed to room temperature and stirred overnight. Water (20 mL) was added to quench the reaction, and the product was extracted into hexanes (6 × 50 mL). The combined organic layers were dried over $MgSO_4$ and filtered. The solvent was removed *in vacuo* to obtain the title compound as off-white crystals (1.82 g 9.05 mmol, 91%).

Spectral data was identical to that previously reported.[6]

^{1}H NMR (400 MHz, $CDCl_3$) δ 7.10 (s, 2H, H^{g}), 4.57 (s, 1H, H^{o}), 2.21 (s, 6H, H^{n}).

^{13}C NMR (101 MHz, $CDCl_3$) δ 151.4 (C^{e}), 131.1 (C^{g}), 125.3 (C^{f}), 112.1 (C^{h}), 15.9 (C^{n}).

3.2.2 1D NMR of 4-bromo-2,6-dimethylphenol (S3)

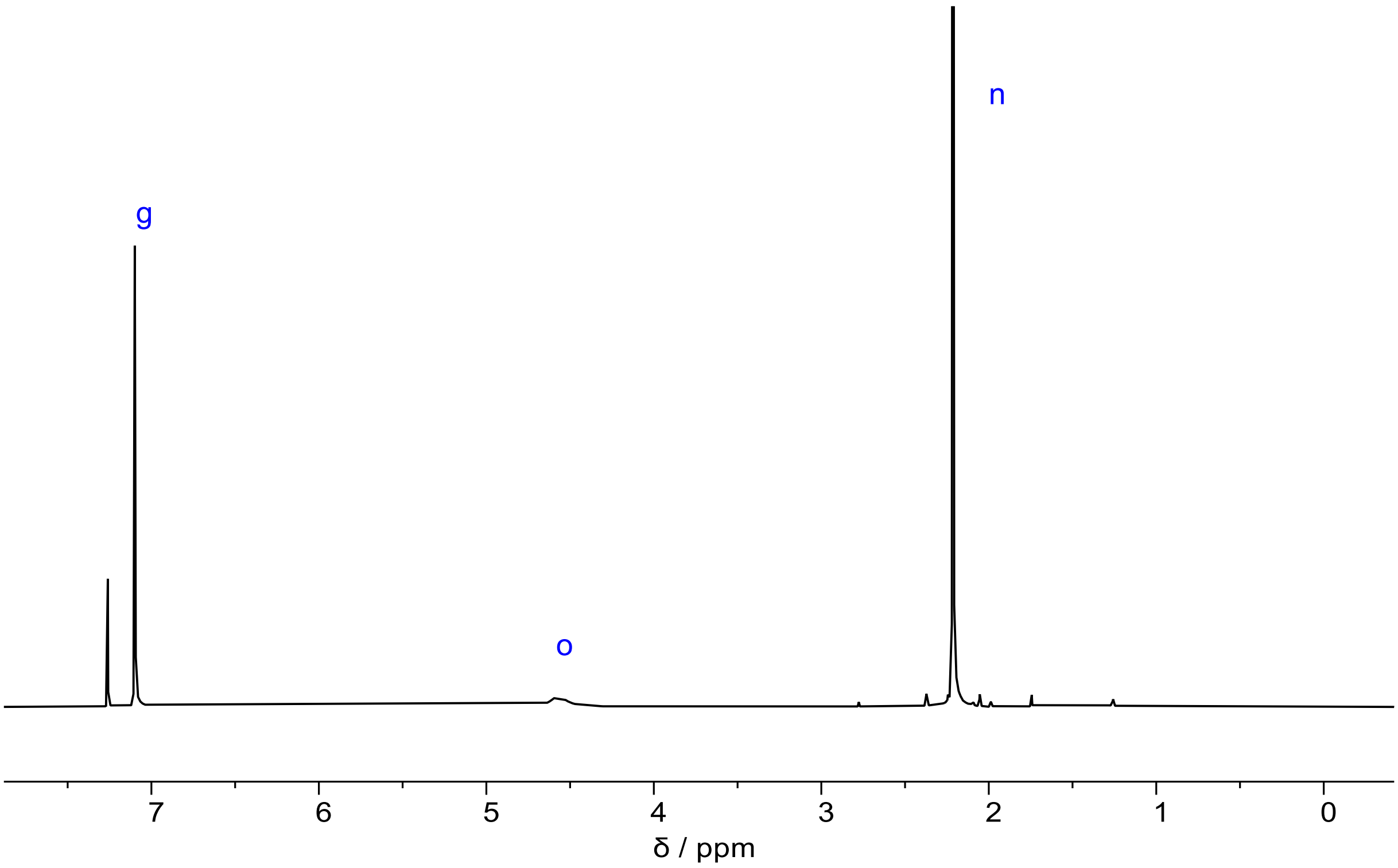

Figure S19. ^{1}H NMR (400 MHz, 298 K, $CDCl_3$) spectrum of intermediate **S3**.

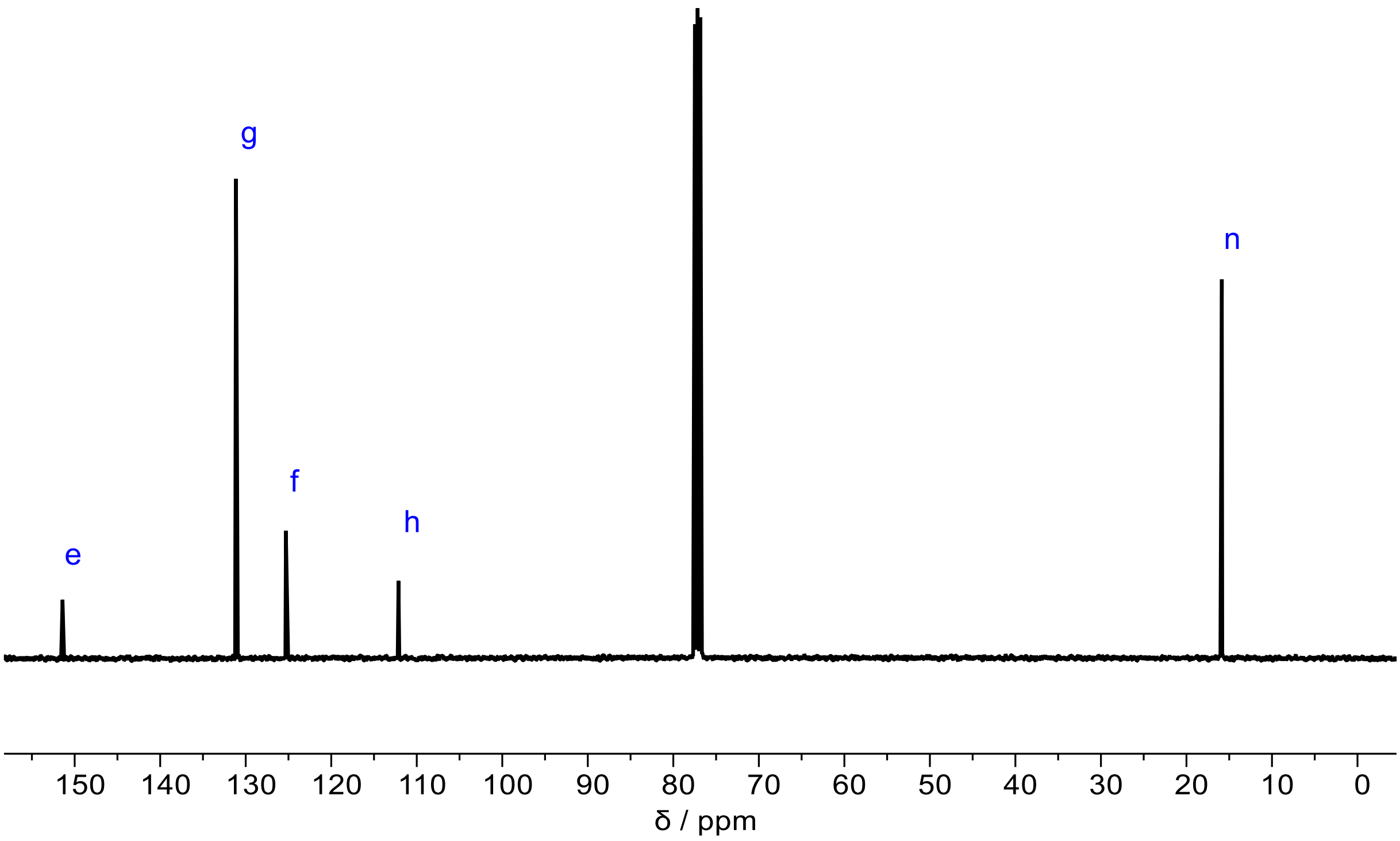

Figure S20. $^{13}C\{^{1}H\}$ NMR (101 MHz, 298 K, $CDCl_3$) spectrum of intermediate **S3**.

3.3 Synthesis and characterization of 2,6-dimethyl-4-(pyridin-3-yl)phenol (S4)

3.3.1 Synthesis of 2,6-dimethyl-4-(pyridin-3-yl)phenol (S4)

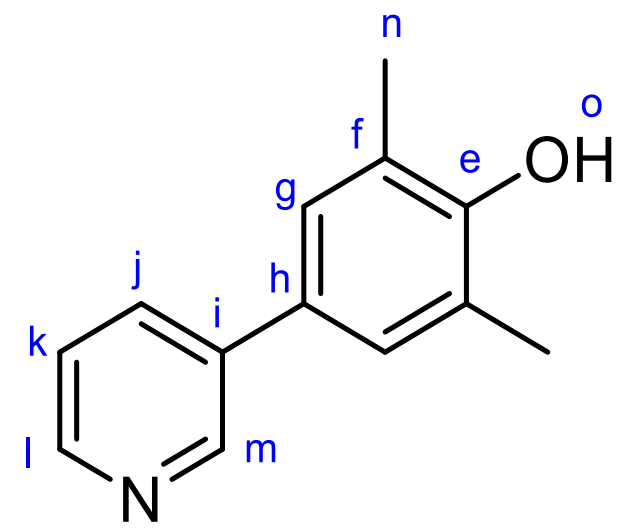

4-Bromo-2,6-dimethylphenol (**S3**, 500 mg, 2.49 mmol. 1.00 equiv.), 3-pyridilboronic acid pinacol ester (816 mg, 3.98 mmol, 1.60 equiv.) and K_2CO_3 (1.75 g, 12.7 mmol, 5.10 equiv.) were placed in a mixture of dioxane/ water (5:1, 18 mL). The mixture was degassed with argon for 30 minutes before Pd(dppf)Cl_2 (44 mg, 60 µmol, 0.02 equiv.) was added. The now red mixture was degassed again with argon for 5 minutes and was heated to 100 °C for 16 h. The reaction mixture was allowed to cool to room temperature, diluted with EtOAc (50 mL), and filtered over Celite. The filtrate was washed with water (50 mL). The aqueous layer was extracted with EtOAc (2 × 50 mL). The organic layers were combined, dried over $MgSO_4$, and the solvent was removed *in vacuo*. The residue was purified by column chromatography (120 g self-packed silica gel column) using EtOAc in hexane (10-50% gradient) to give the product as white solid. The solid was dissolved in diethyl ether (10 mL) and hexane (5 mL) was carefully layered over the diethyl ether phase, leading to immediate crystallization of the product. The crystallization was left overnight, and the off-white crystals were collected by filtration and washed with hexane. The title compound was obtained as off-white crystals (428 mg, 2.15 mmol, 86%).

^{1}H NMR (400 MHz, DMSO-d_6) δ 8.80 (dd, J = 2.4, 0.9 Hz, 1H, H^m), 8.48 (s, 1H, H^o), 8.46 (dd, J = 4.7, 1.6 Hz, 1H, H^l), 7.95 (ddd, J = 8.0, 2.4, 1.6 Hz, 1H, H^j), 7.40 (ddd, J = 7.9, 4.7, 0.9 Hz, 1H, H^k), 7.29 (s, 2H, H^g), 2.23 (s, 6H, H^n).

^{13}C NMR (101 MHz, DMSO-d_6) δ 153.7 (C^e), 147.3 (C^l), 147.1 (C^m), 135.7 (C^i), 133.2 (C^j), 127.7 (C^h), 126.7 (C^g), 124.9 (C^f), 123.7 (C^k), 16.7 (C^n).

ESI-MS (m/z): 200.1053 $[M+H]^+$ (calculated for $C_{13}H_{14}NO^+$ 200.1070)

3.3.2 1D and 2D NMR characterization of 2,6-dimethyl-4-(pyridin-3-yl)phenol (S4)

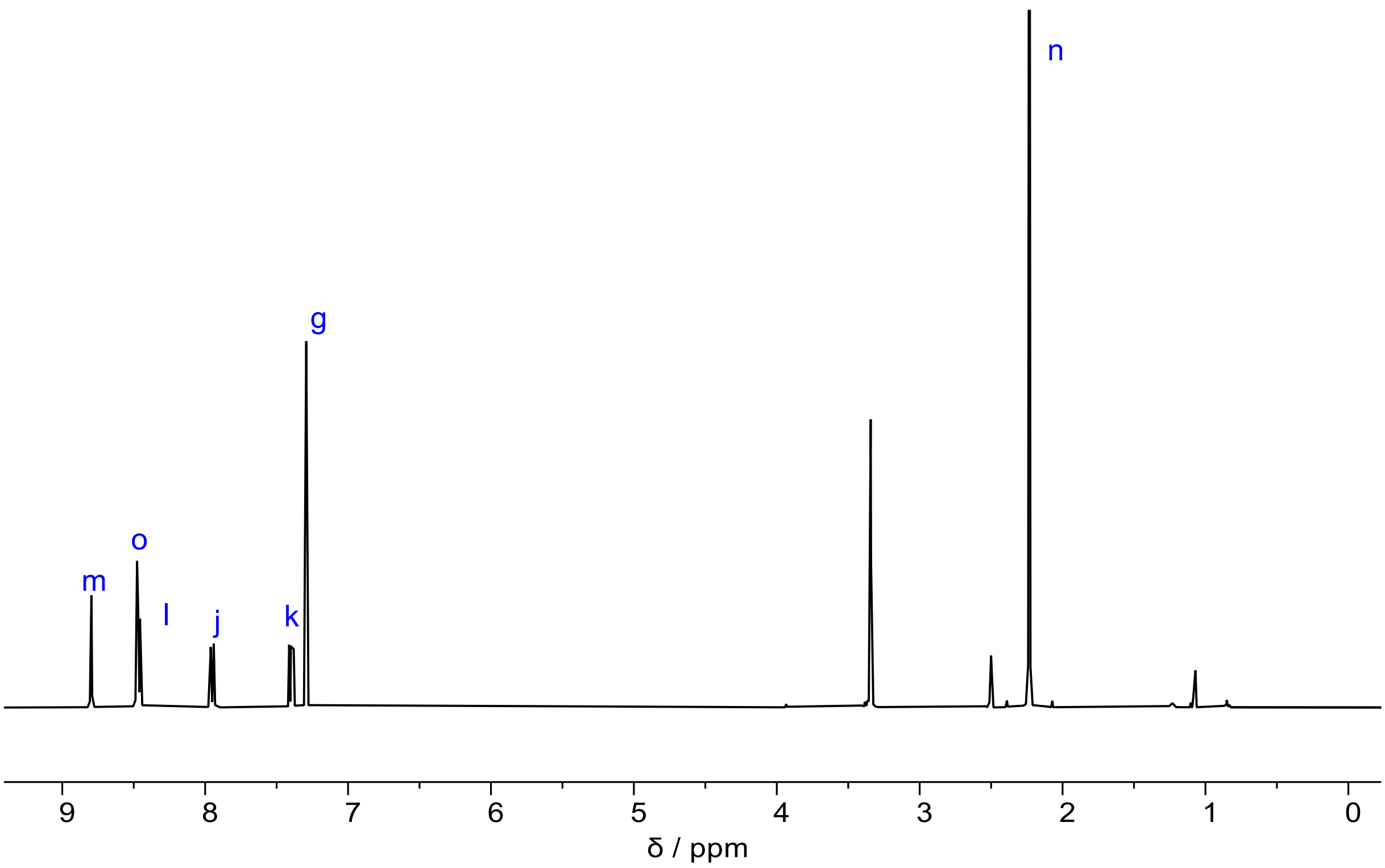

Figure S21. ^{1}H NMR (400 MHz, 298 K, DMSO-d_6) spectrum of intermediate **S4**.

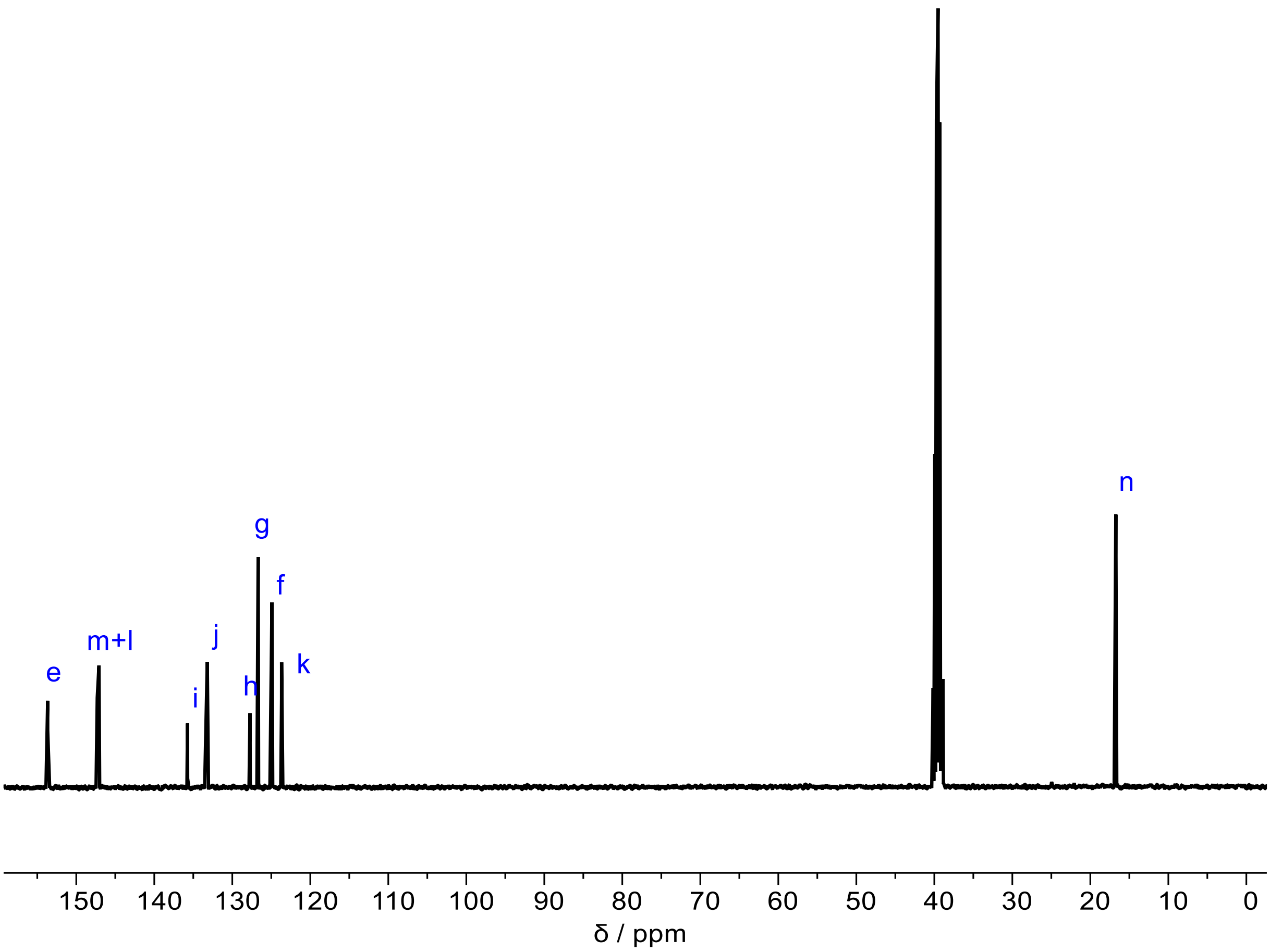

Figure S22. $^{13}C\{^{1}H\}$ NMR (101 MHz, 298 K, DMSO-d_6) spectrum of intermediate **S4**.

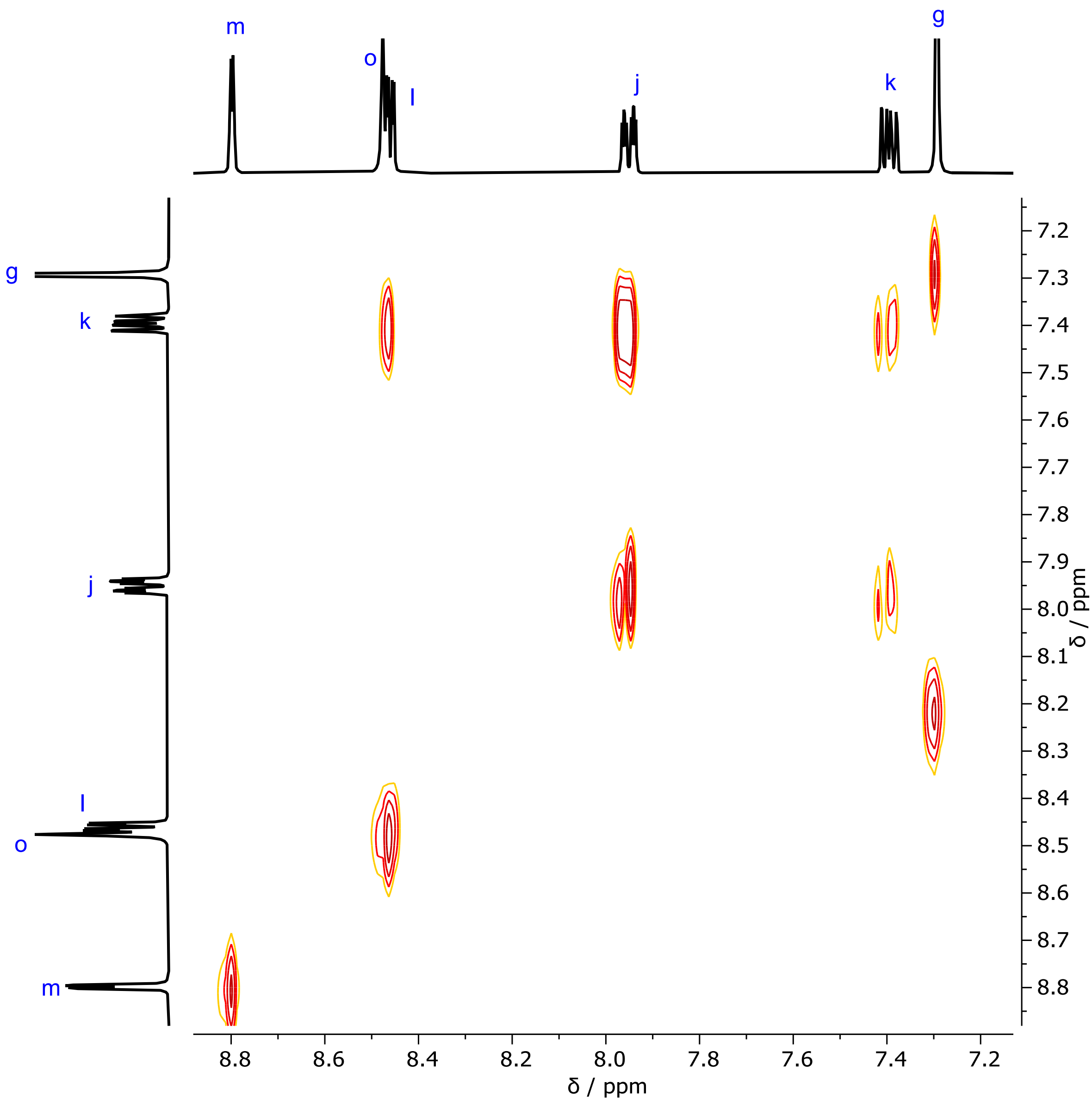

Figure S23. Partial ^{1}H-^{1}H COSY NMR (400 MHz, 298 K, DMSO-d_6) spectrum of intermediate **S4**.

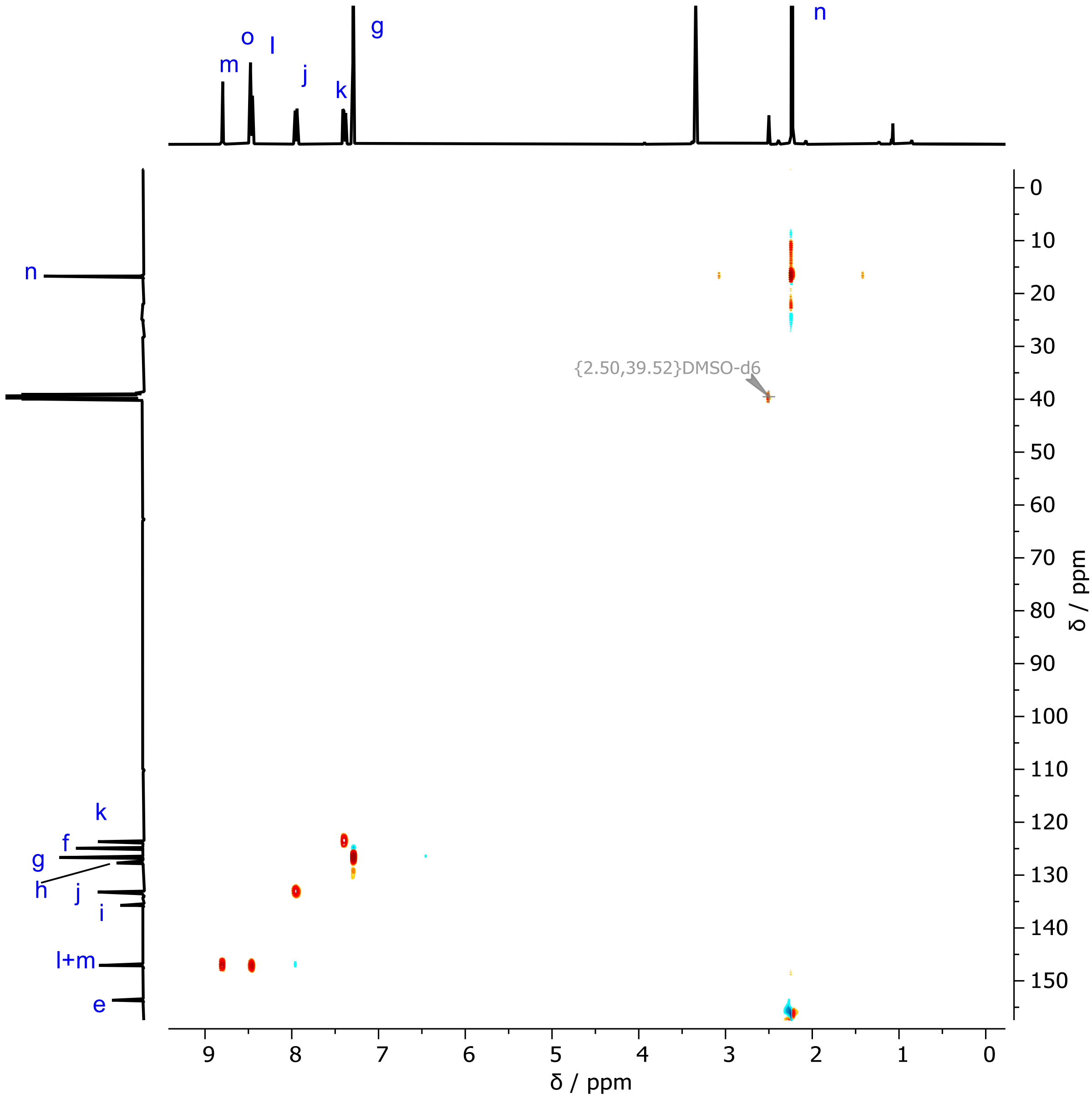

Figure S24. ^{1}H-^{13}C HSQC NMR (400 MHz, 101 MHz, 298 K, DMSO-d_6) spectrum of intermediate **S4**.

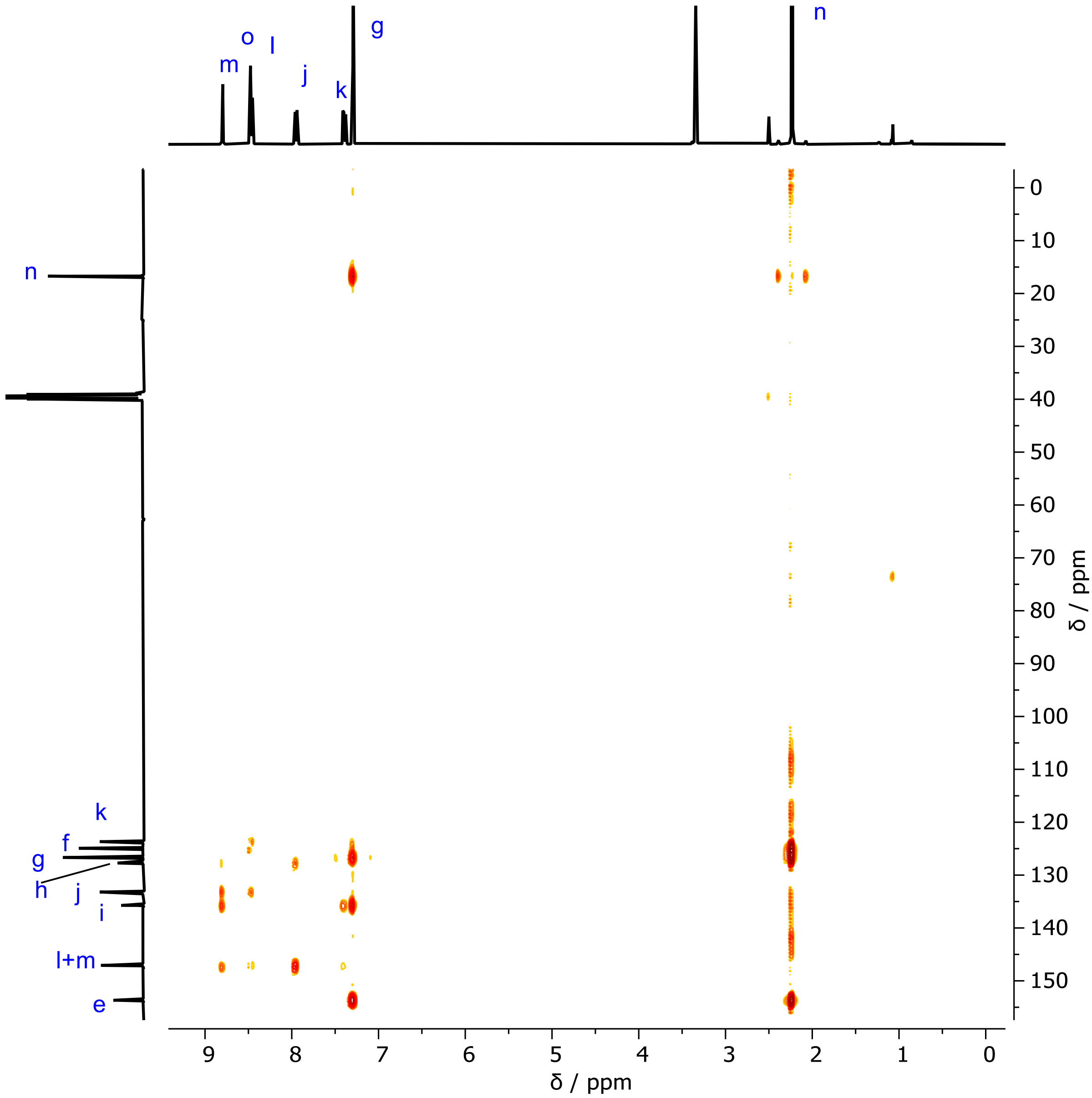

Figure S25. ^{1}H-^{13}C HMBC NMR (400 MHz, 101 MHz, 298 K, DMSO-d_6) spectrum of intermediate **S4**.

3.4 Synthesis and characterization of intermediate S5

3.4.1 Synthesis of intermediate S5

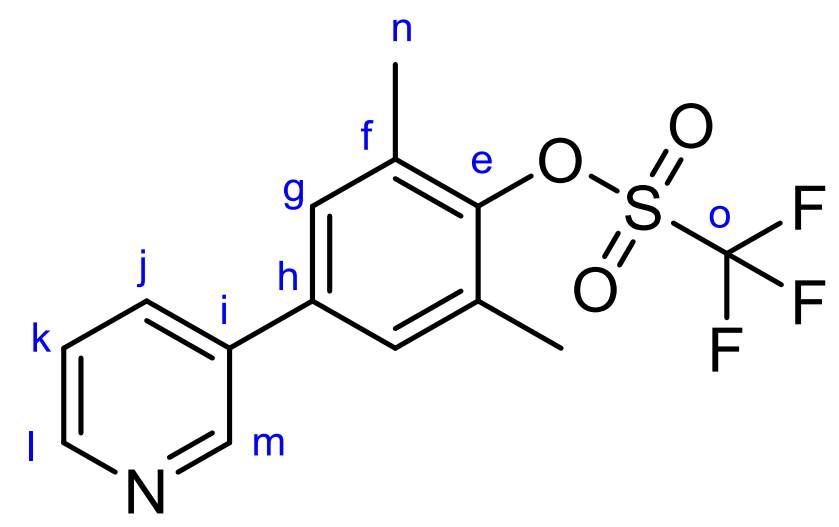

2,6-Dimethyl-4-(pyridin-3-yl)phenol (**S4**, 400 mg, 2.01 mmol. 1.00 equiv.) was dissolved in dry DCM (15 mL) under argon at room temperature. Triethylamine (420 µL, 3.01 mmol, 1.50 equiv.) was added to the slightly yellow solution and the reaction mixture was cooled to 0 °C with an ice bath. Trifluoromethanesulfonic anhydride (370 µL, 2.21 mmol, 1.10 equiv.) was added dropwise, leading to an immediate color change to dark purple. After the addition, the ice bath was removed, allowing the reaction mixture to warm to room temperature, where it was stirred for 16 hours. The reaction mixture was quenched by the addition of saturated aqueous Na_2CO_3 solution (20 mL), and the aqueous phase was extracted three times with DCM (3 × 20 mL). The organic layers were combined, dried over $MgSO_4$, and the solvent was removed *in vacuo*, to obtain the crude material as a dark red oil, which crystallized while standing overnight. The residue was purified by column chromatography (120 g self-packed silica gel column) using EtOAc in hexane (10-40% gradient) to obtain the product as orange crystals (482 mg, 1.45 mmol, 72%).

^{1}H NMR (400 MHz, DMSO-d_6) δ 8.91 (dd, *J* = 2.5, 0.9 Hz, 1H, H^{m}), 8.60 (dd, *J* = 4.8, 1.6 Hz, 1H, H^{l}), 8.10 (ddd, *J* = 7.9, 2.4, 1.6 Hz, 1H, H^{j}), 7.67 (s, 2H, H^{g}), 7.50 (ddd, *J* = 8.0, 4.8, 0.9 Hz, 1H, H^{k}), 2.40 (s, 6H, H^{n}).

^{19}F NMR (376 MHz, DMSO-d_6) δ -73.34 (F^{o}).

^{13}C NMR (101 MHz, DMSO-d_6) δ 149.5 (C^{l}), 148.3 (C^{m}), 147.0 (C^{e}), 137.6 (C^{h}), 134.8 (C^{j}), 134.4 (C^{i}), 132.0 (C^{f}), 129.1 (C^{g}), 124.3 (C^{k}), 117.6 (q, *J* = 319.8 Hz, C^{o}), 17.1 (C^{n}).

ESI-MS (m/z): 332.0535 $[M+H]^+$ (calculated for $C_{14}H_{13}F_3NO_3S^+$ 332.0563)

3.4.2 1D and 2D NMR of intermediate S5

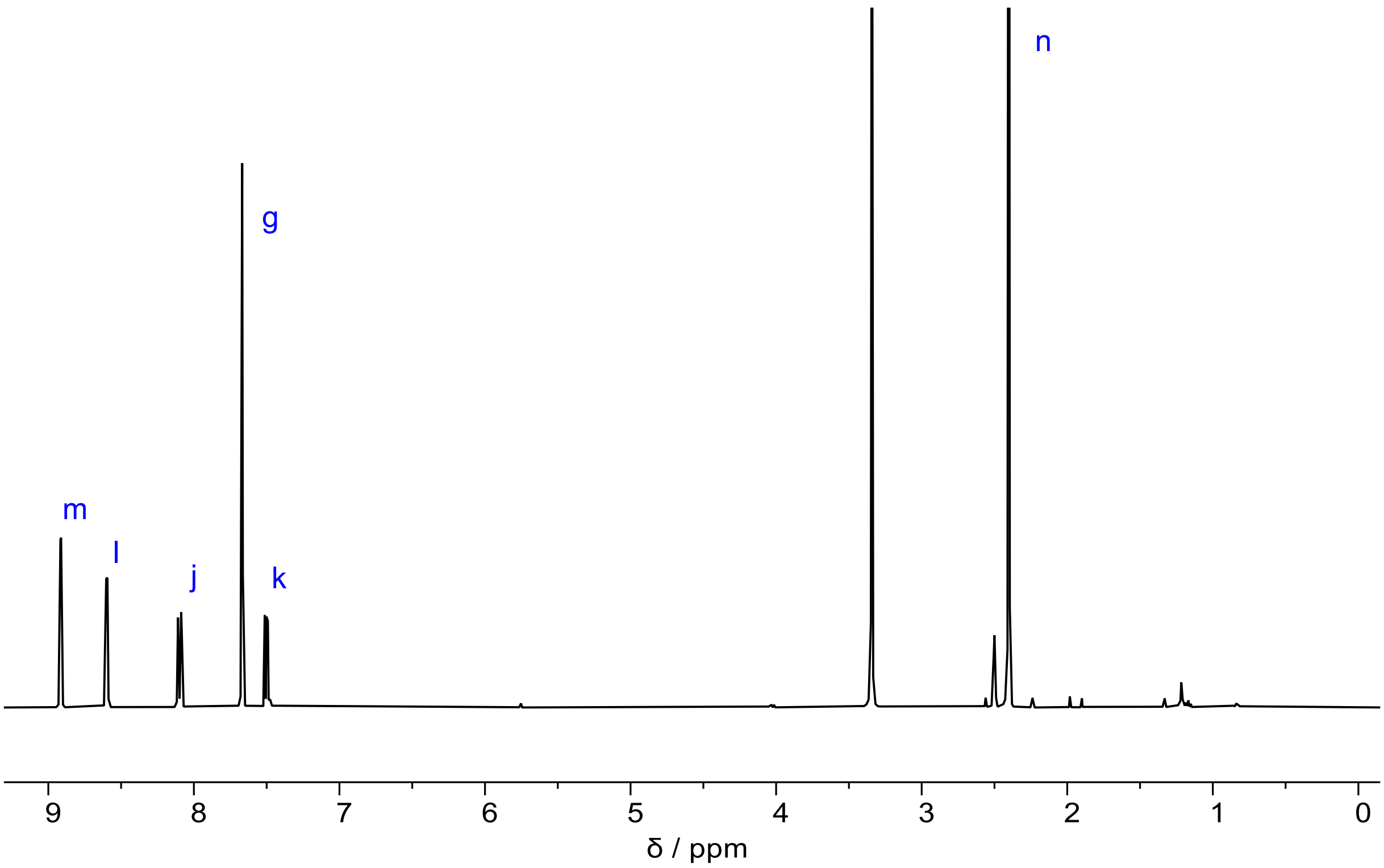

Figure S26. ^{1}H NMR (400 MHz, 298 K, DMSO-d_6) spectrum of intermediate **S5**.

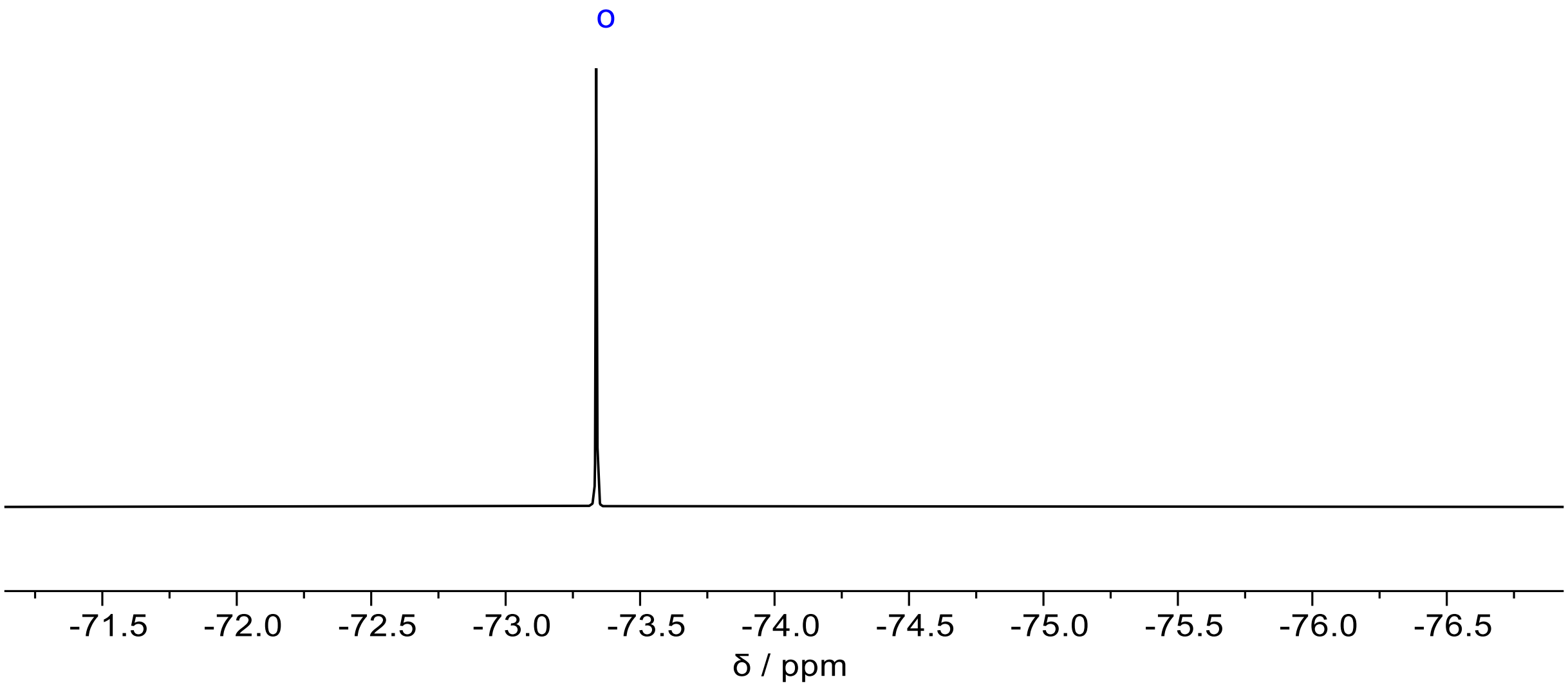

Figure S27. ^{19}F NMR (376 MHz, 298 K, DMSO-d_6) spectrum of intermediate **S5**.

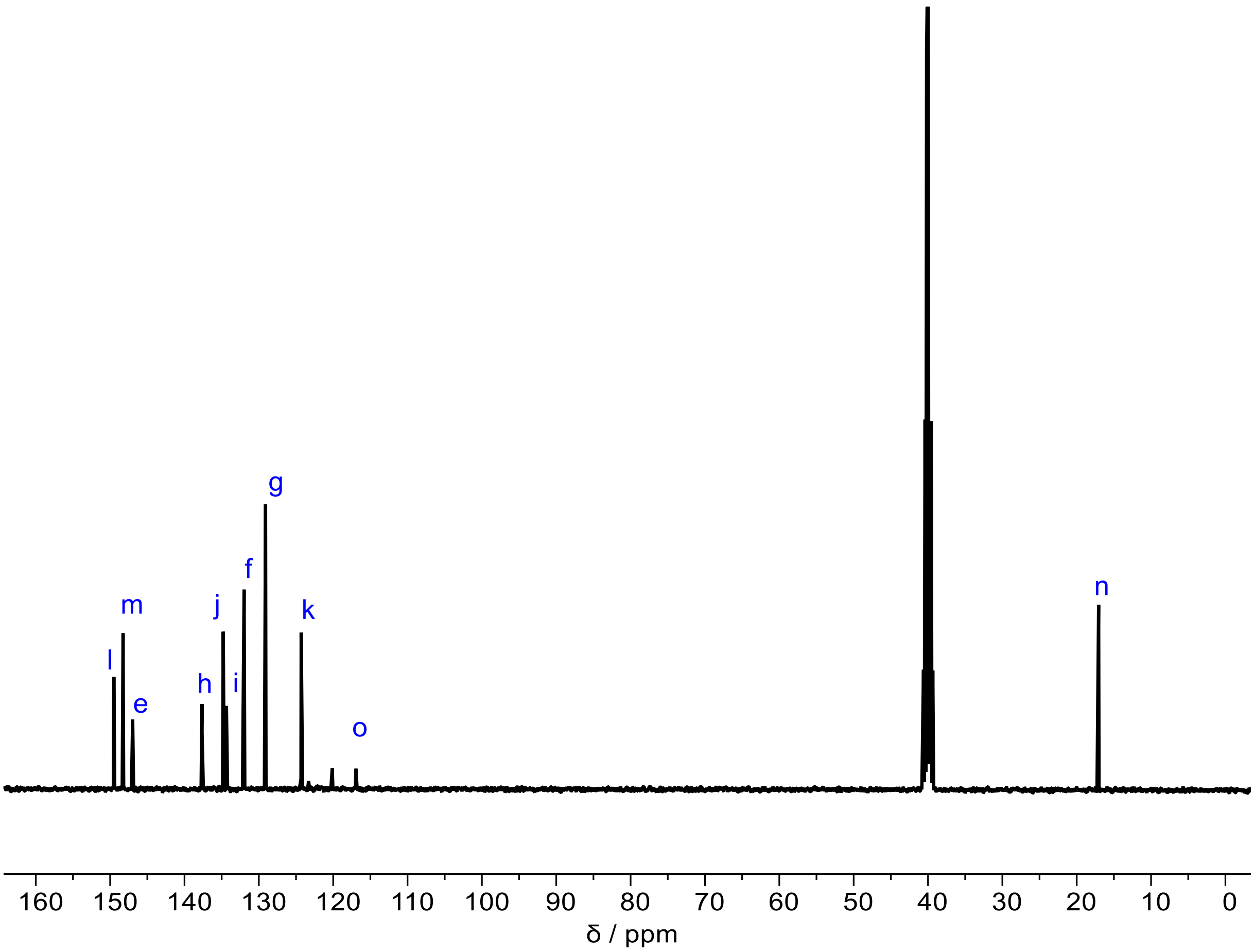

Figure S28. $^{13}C\{^{1}H\}$ NMR (101 MHz, 298 K, DMSO-d_6) spectrum of intermediate **S5**.

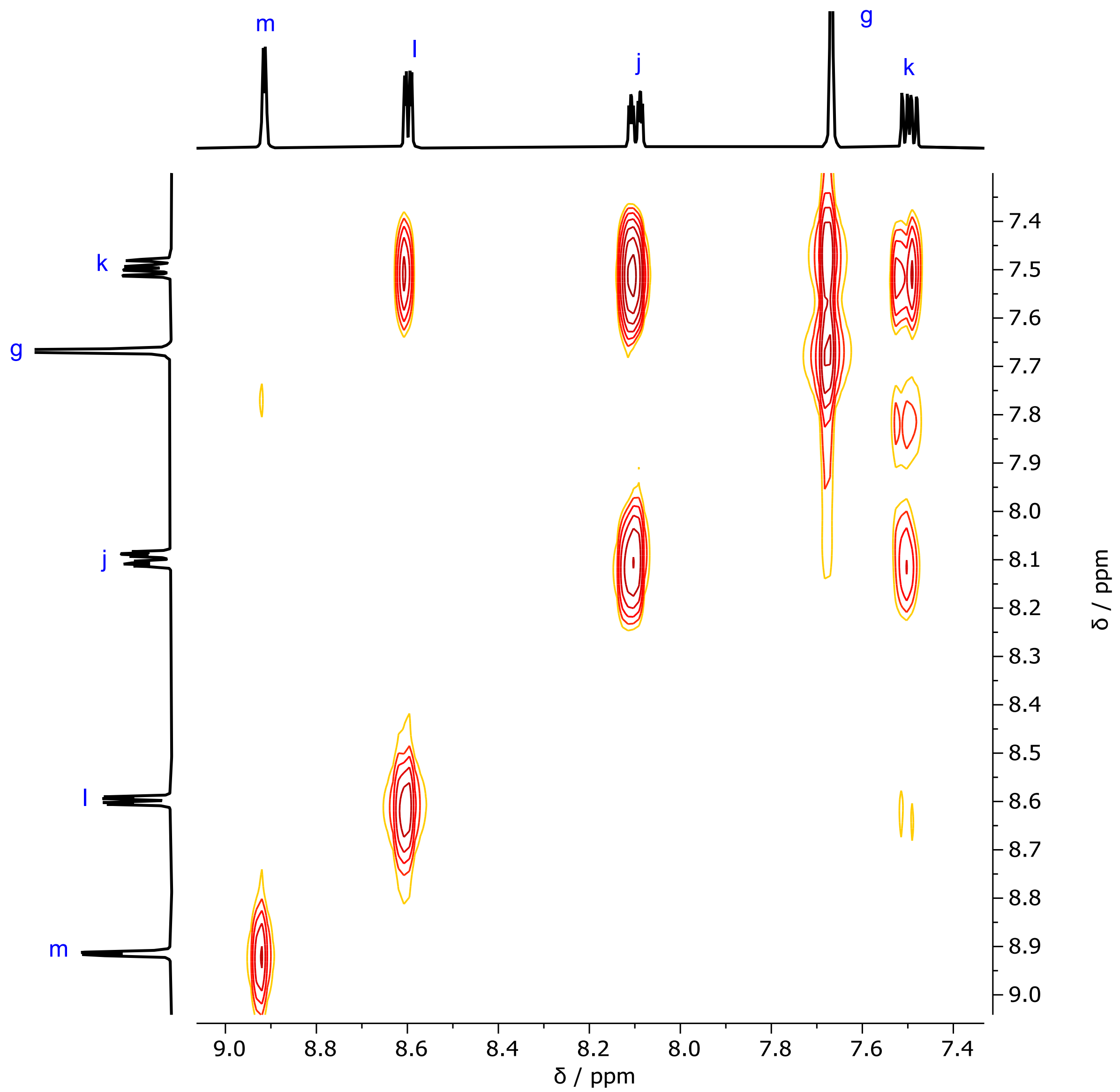

Figure S29. ^{1}H-^{1}H COSY NMR (400 MHz, 298 K, DMSO-d_6) spectrum of intermediate **S5**.

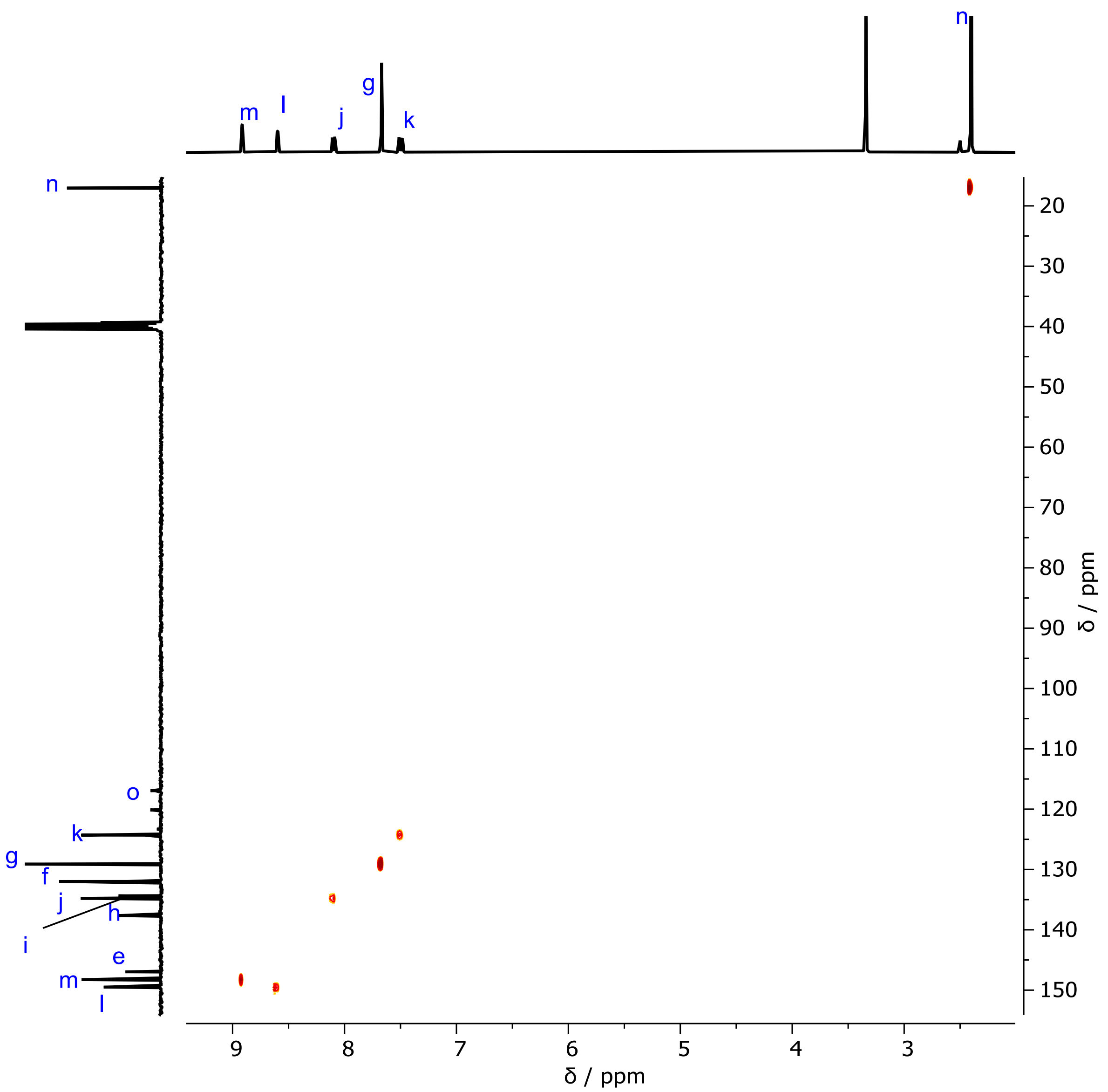

Figure S30. ^{1}H-^{13}C HSQC NMR (400 MHz, 101 MHz, 298 K, DMSO-d_6) spectrum of intermediate **S5**.

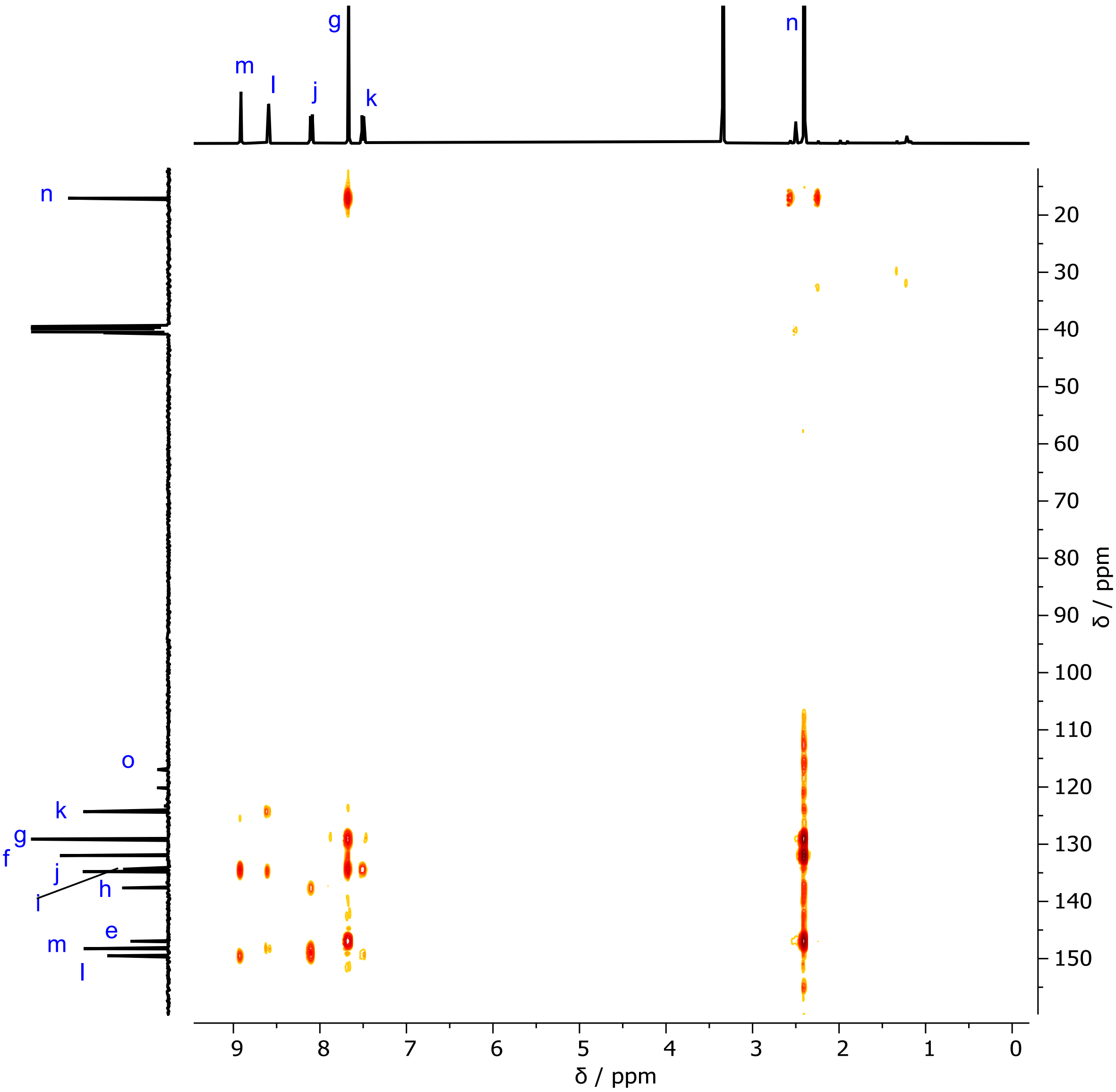

Figure S31. ^{1}H-^{13}C HMBC NMR (400 MHz, 101 MHz, 298 K, DMSO-d_6) spectrum of intermediate **S5**.

3.5 Synthesis and characterization of ligand 1

3.5.1 Synthesis of ligand 1

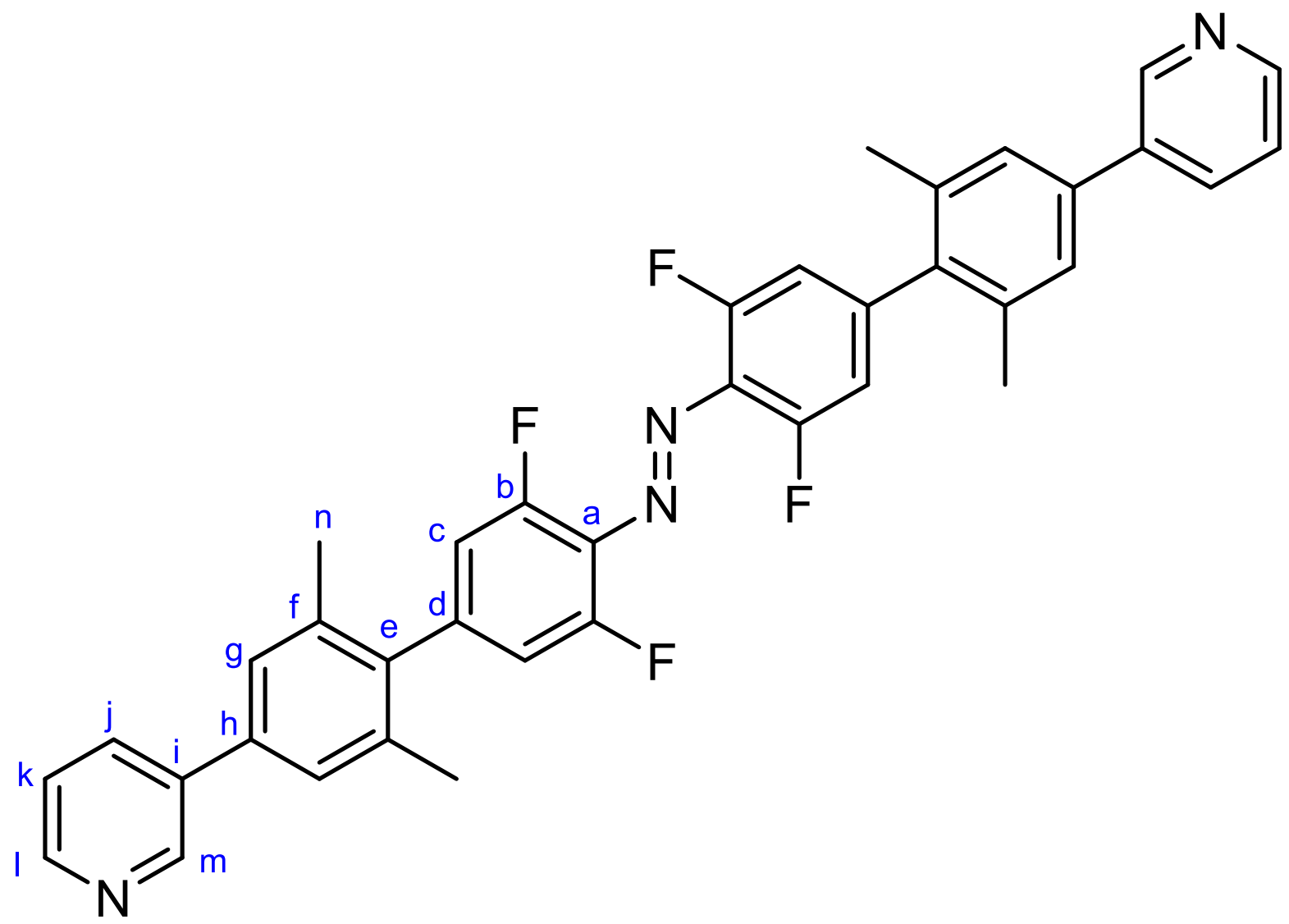

Intermediate **S2** (100 mg, 162 µmol, 1.00 equiv.) and intermediate **S5** (214 mg, 647 µmol, 4.00 equiv.) were mixed at room temperature with dioxane (3.0 mL). Then K_2CO_3 (55.9 mg, 404 µmol, 2.50 equiv.) in H_2O (0.6 mL) was added. The red mixture was degassed with argon for 20 minutes before Pd(dppf)Cl_2 (12 mg, 16 µmol, 0.1 equiv.) was added. The mixture was degassed for further 5 minutes and then placed in the microwave for 50 minutes at 130 °C. The reaction mixture was allowed to cool to room temperature, diluted with DCM (50 mL) and filtered over Celite. The organic phase was washed with water (2 × 50 mL) and brine (50 mL). The organic layer was dried over $MgSO_4$, and the solvent was removed *in vacuo*. The residue was purified twice by column chromatography (40 g, self-packed column) using EtOAc in hexane (10-100% gradient) first and then MeOH in DCM (0-10% gradient). The title compound was obtained as red crystals (45 mg, 73 µmol, 45%).

Ligand *E*-1:

^{1}H NMR (600 MHz, DMSO-d_6) δ 8.94 (d, *J* = 2.3 Hz, 2H, H^m), 8.59 (dd, *J* = 4.8, 1.6 Hz, 2H, H^l), 8.12 (ddd, *J* = 7.9, 2.4, 1.6 Hz, 2H, H^j), 7.56 (s, 4H, H^g), 7.51 (ddd, *J* = 7.9, 4.8, 0.8 Hz, 2H, H^k), 7.34 (dd, *J* = 9.8, 1.3 Hz, 4H, H^c), 2.18 (s, 12H, H^n).

^{19}F NMR (565 MHz, DMSO-d_6) δ -121.53 (d, *J* = 10.6 Hz, F^b).

^{13}C NMR (151 MHz, DMSO-d_6) δ 154.7 (dd, *J* = 260.0, 5.0 Hz, C^b), 148.6 (C^l), 147.7 (C^m), 145.5 (t, *J* = 10.5 Hz, C^d), 138.4 (C^e), 136.8 (C^h), 136.2 (C^f), 135.2 (C^i), 134.1 (C^j), 129.6 (t, *J* = 10.1 Hz, C^a), 126.1 (C^g), 123.9 (C^k), 114.2 (dd, *J* = 19.6, 3.5 Hz, C^c), 20.5 (C^n).

ESI-MS (m/z): 617.2307 $[M+H]^+$ (calculated for $C_{38}H_{29}F_4N_4^+$ 617.2323)

Ligand *Z*-1:

^{1}H NMR (600 MHz, DMSO) δ 8.90 (d, *J* = 2.4 Hz, 2H, H^m), 8.57 (dd, *J* = 4.8, 1.6 Hz, 2H, H^l), 8.08 (ddd, *J* = 7.9, 2.4, 1.6 Hz, 2H, H^j), 7.51 (s, 4H, H^g), 7.50 – 7.46 (m, 2H, H^k), 7.17 (d, *J* = 8.4 Hz, 4H, H^c), 2.02 (s, 12H, H^n).

^{19}F NMR (565 MHz, DMSO) δ -120.76 (d, *J* = 7.9 Hz, F^b)

3.5.2 1D and 2D NMR characterization of ligand *E*-1

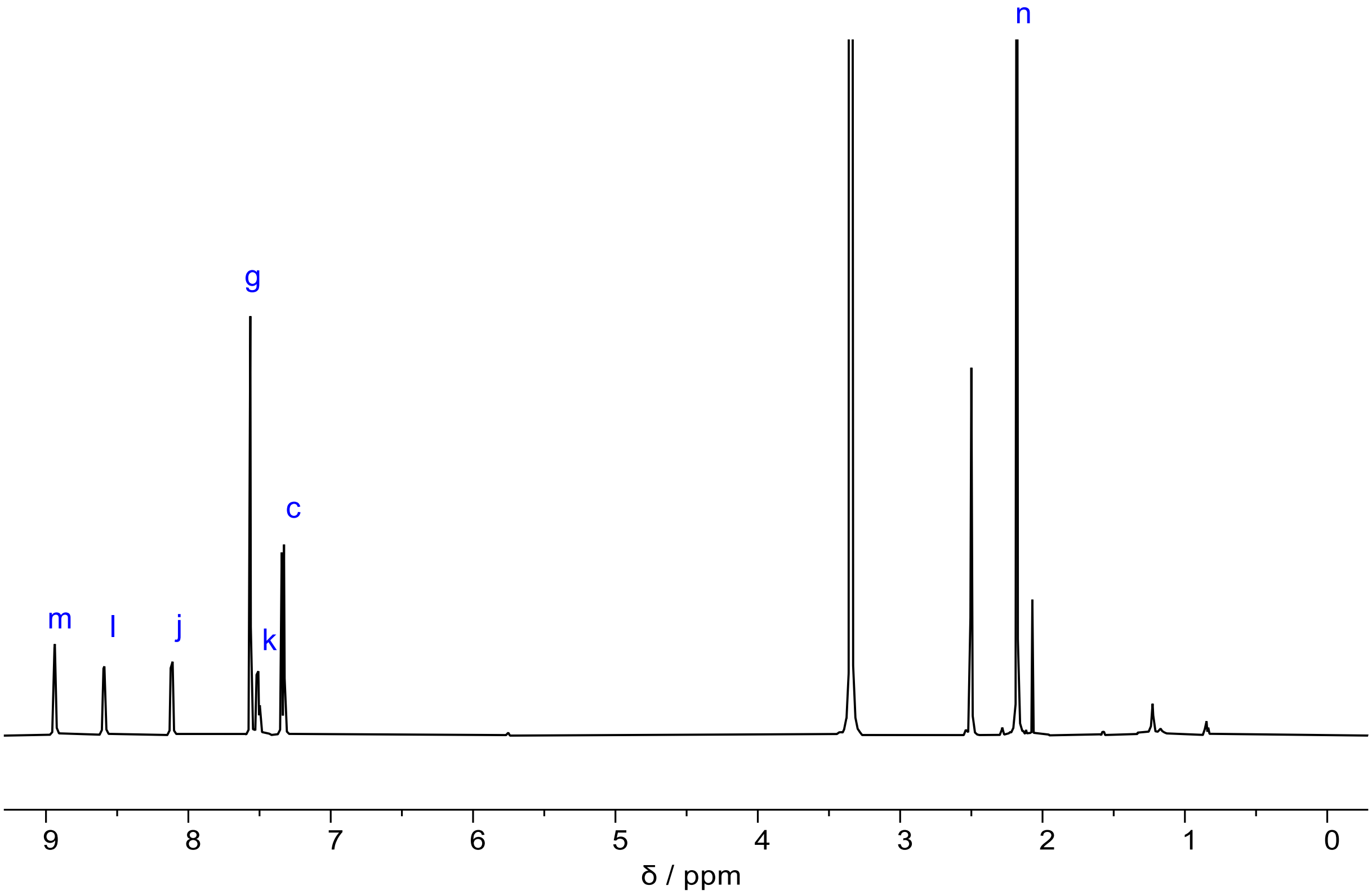

Figure S32. ^{1}H NMR (600 MHz, 298 K, DMSO-d_6) spectrum of ligand *E*-**1**.

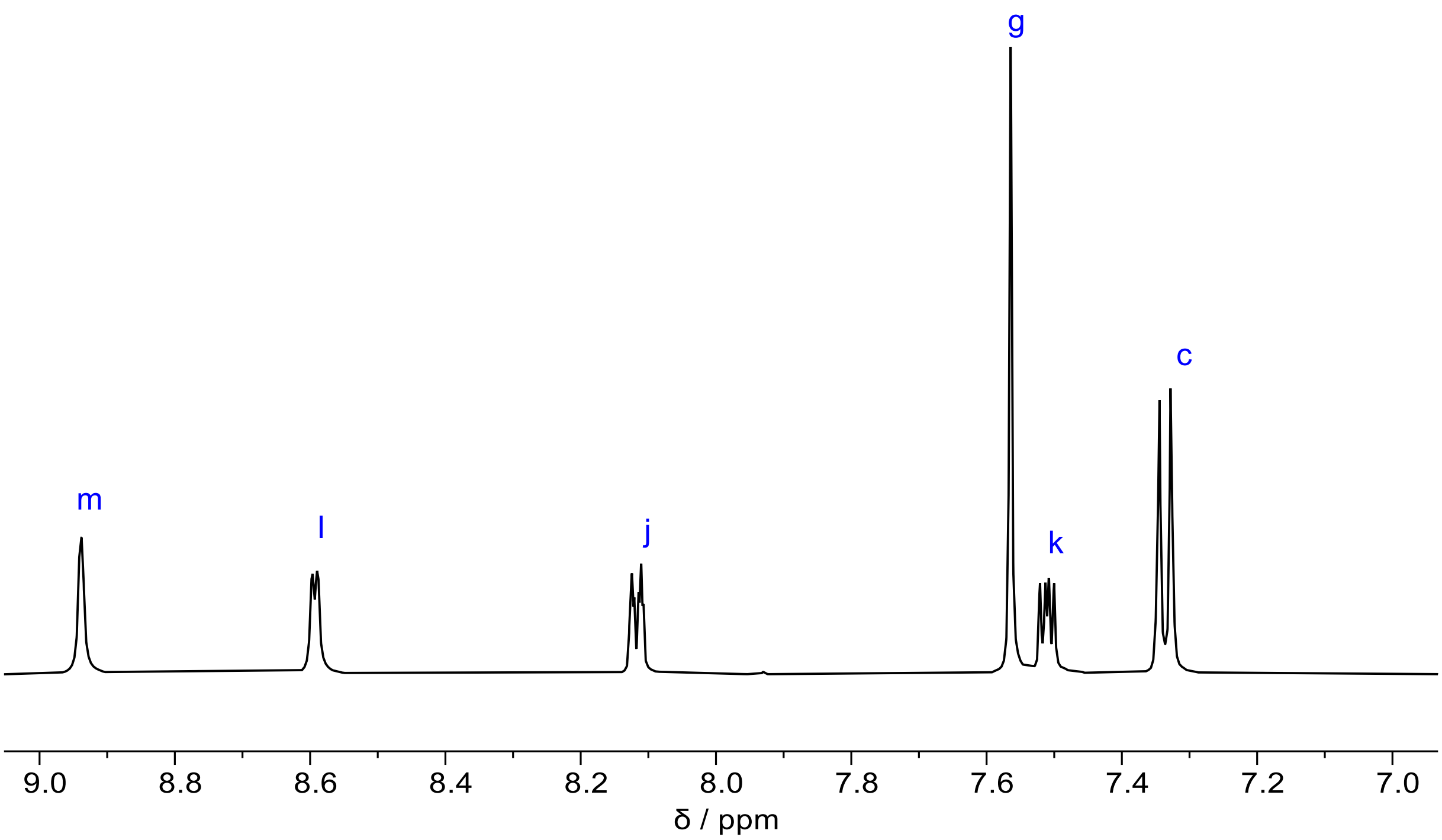

Figure S33. Partial ^{1}H NMR (600 MHz, 298 K, DMSO-d_6) spectrum of ligand *E*-**1**.

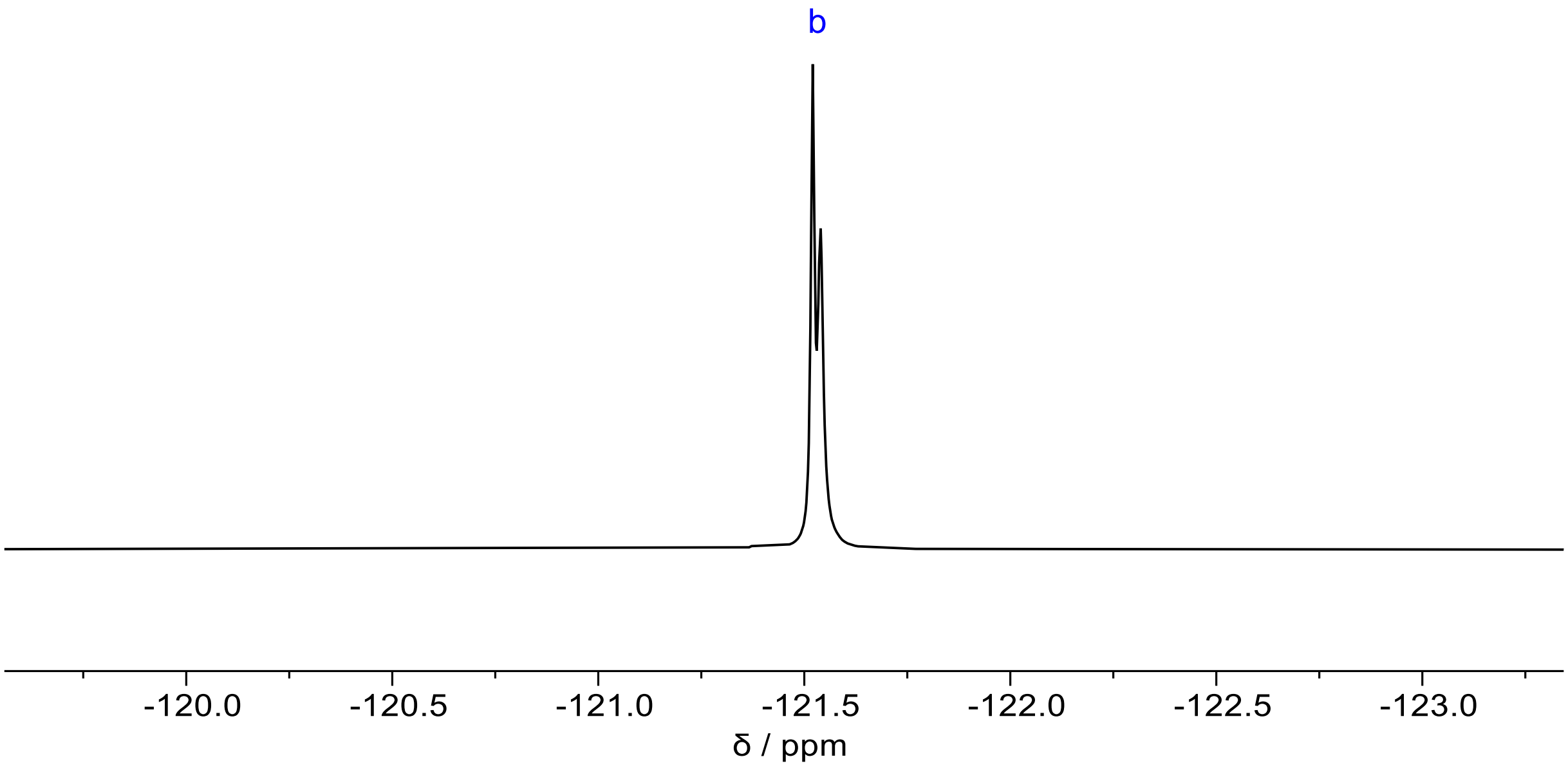

Figure S34. Partial [1]F NMR (565 MHz, 298 K, DMSO-*d*6) spectrum of ligand *E*-**1**.

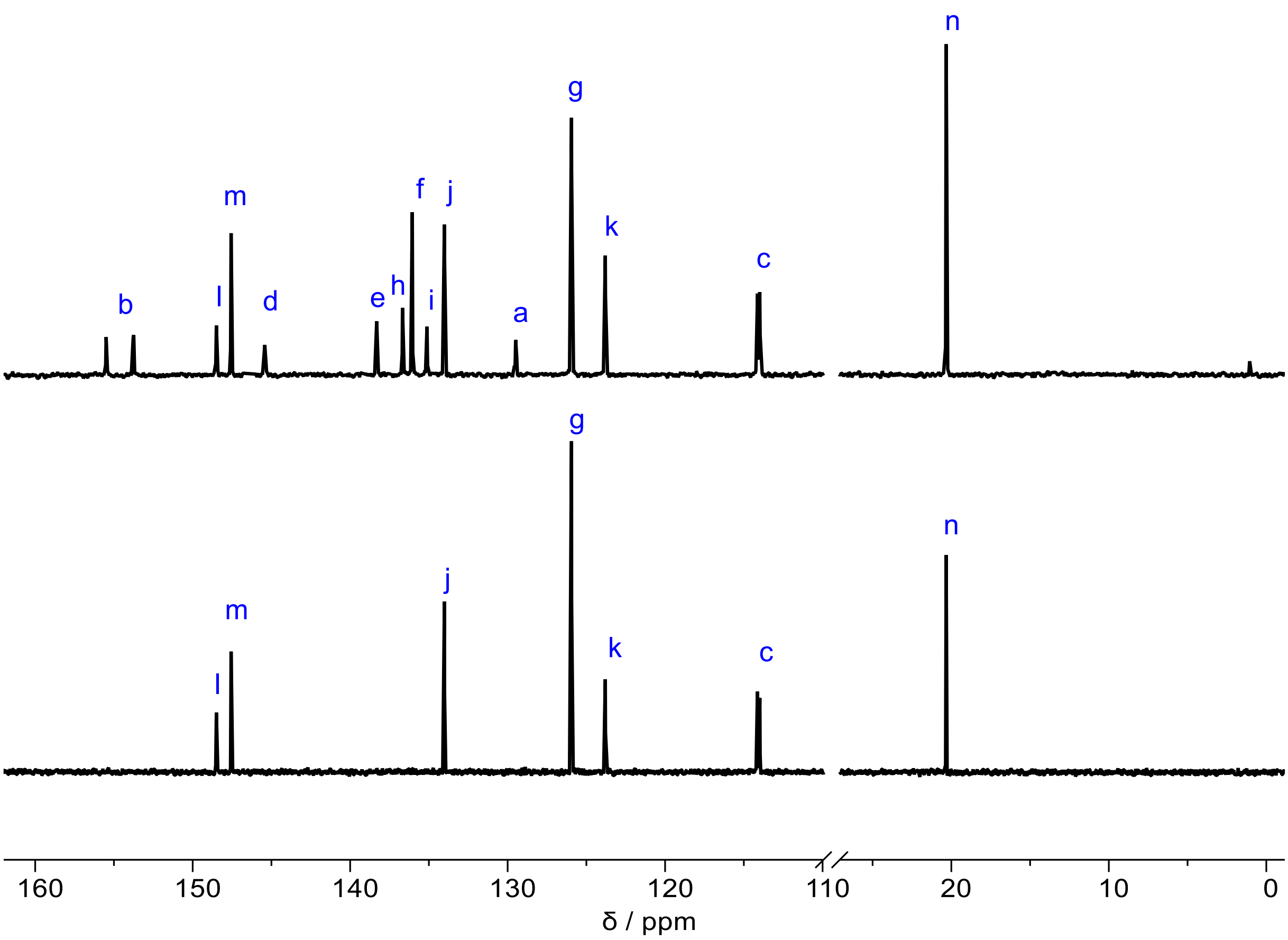

Figure S35. ^{13}C{^{1}H} and 135-DEPT NMR (151 MHz, 298 K, DMSO-*d*6) spectrum of ligand *E*-**1**.

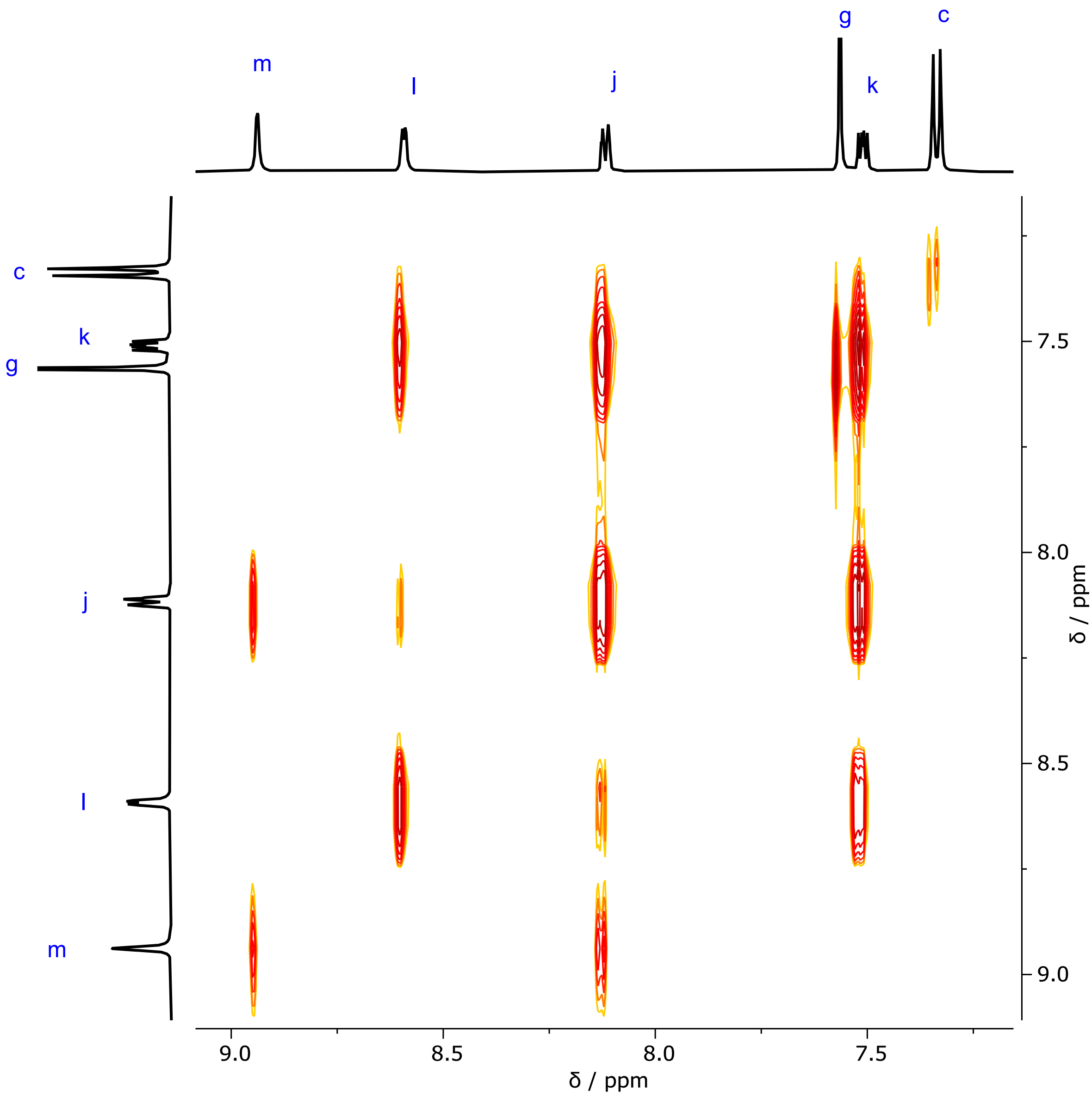

Figure S36. ^{1}H-^{1}H COSY NMR (600 MHz, 298 K, DMSO-d_6) spectrum of ligand *E*-**1**.

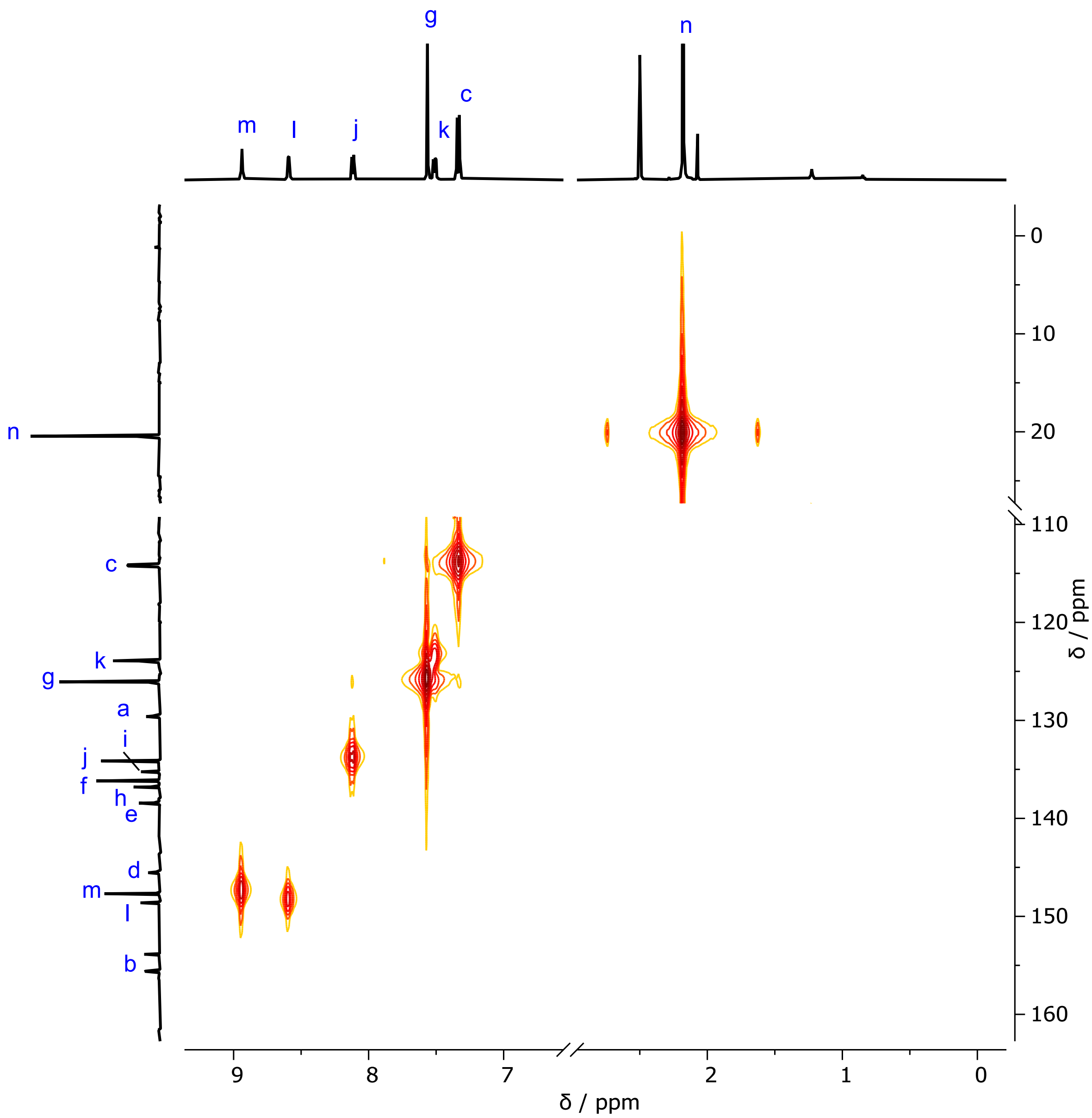

Figure S37. ^{1}H-^{13}C HSQC NMR (600 MHz, 151 MHz, 298 K, DMSO-d_6) spectrum of ligand *E*-**1**.

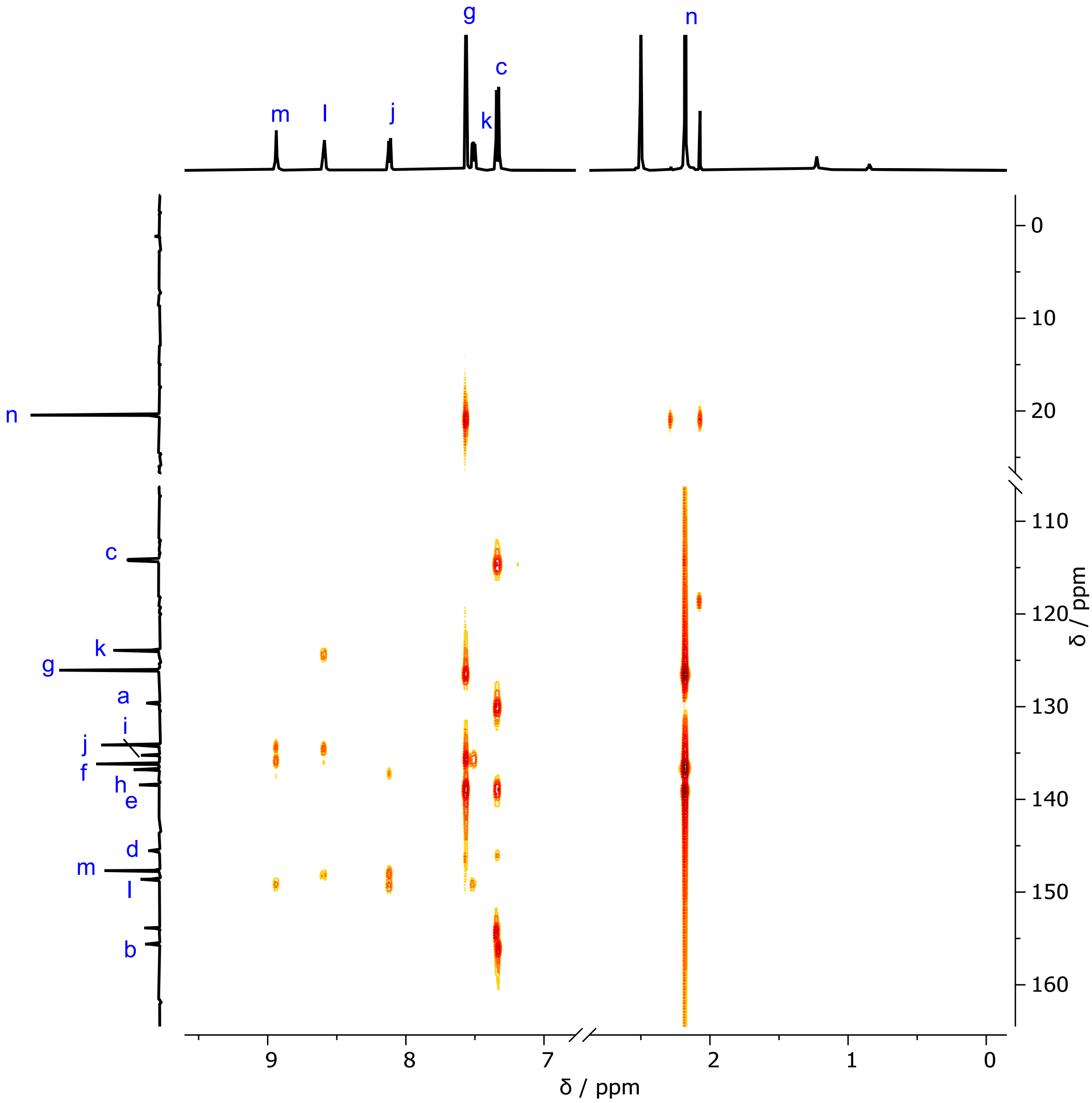

Figure S38. ^{1}H-^{13}C HMBC NMR (600 MHz, 151 MHz, 298 K, DMSO-d_6) spectrum of ligand *E*-**1**.

3.5.3 1D NMR characterization of a mixture of ligand *E*-1 and *Z*-1

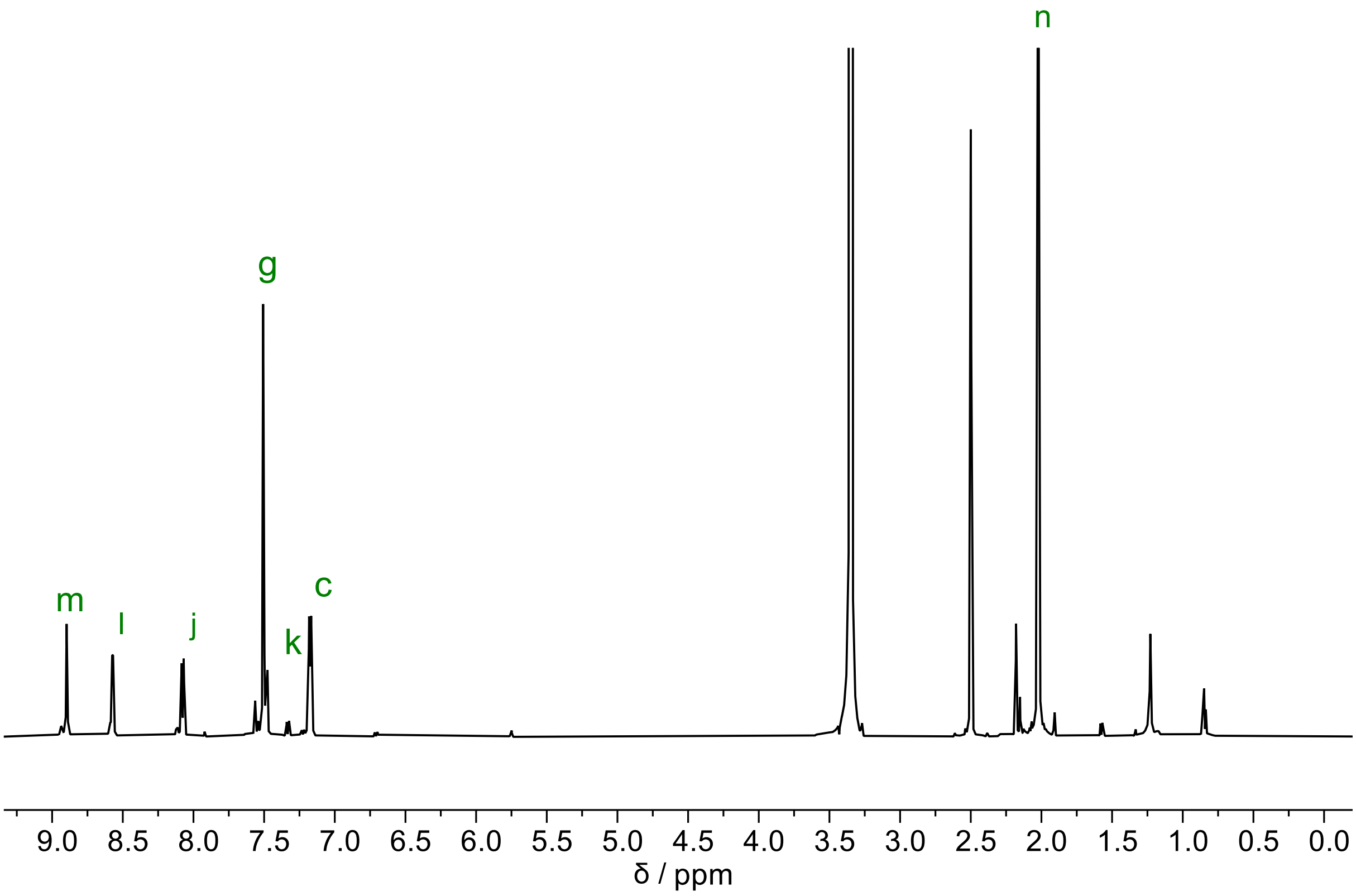

Figure S39. ^{1}H NMR (600 MHz, 298 K, DMSO-*d*$_6$) spectrum of a mixture of ligand *Z*-**1** and *E*-**1** obtained after irradiating a solution of *E*-**1** with 530 nm light for 30 min.

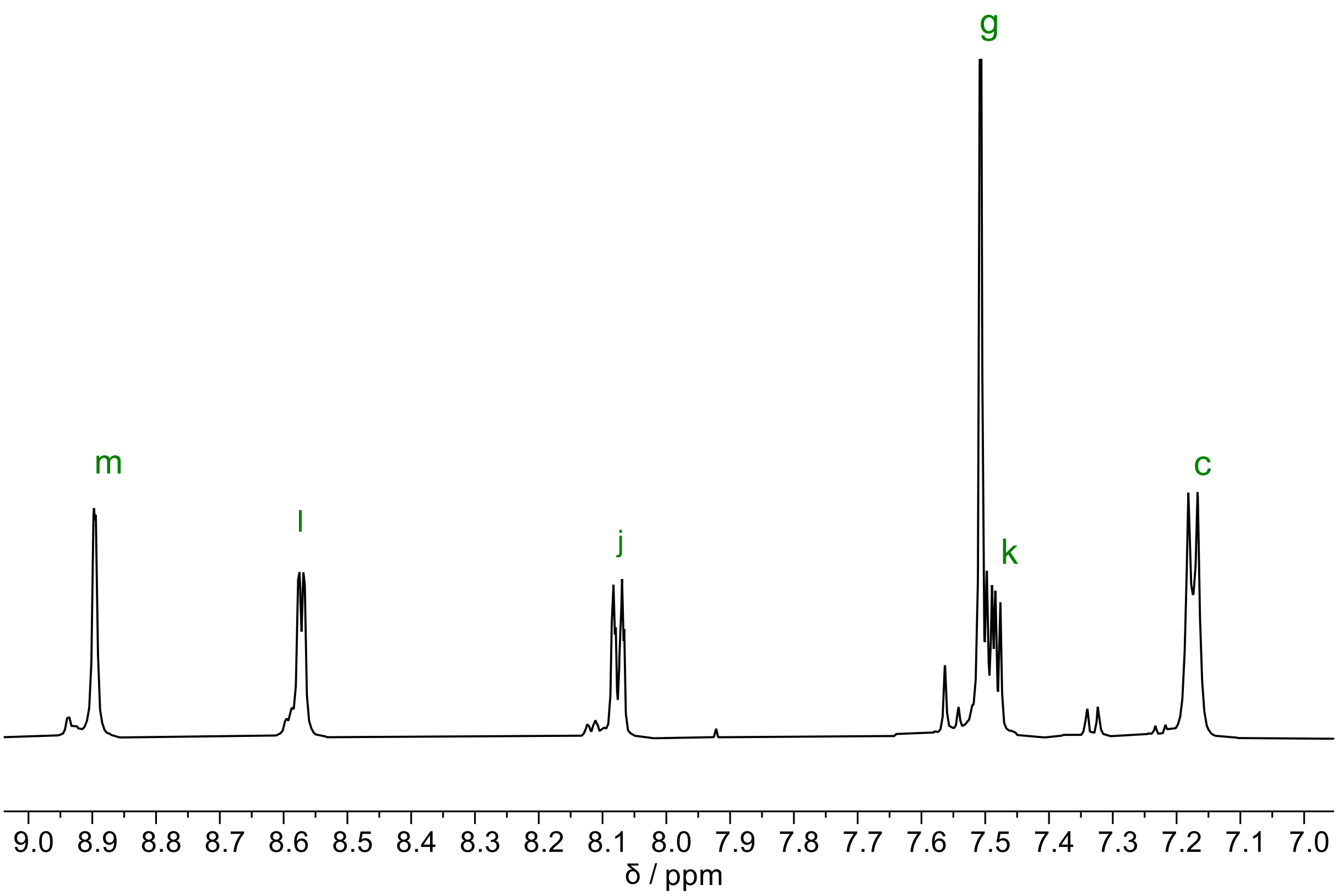

Figure S40. Partial ^{1}H NMR (600 MHz, 298 K, DMSO-*d*$_6$) spectrum of a mixture of ligand *Z*-**1** and *E*-**1** obtained after irradiating a solution of *E*-**1** with 530 nm light for 30 min.

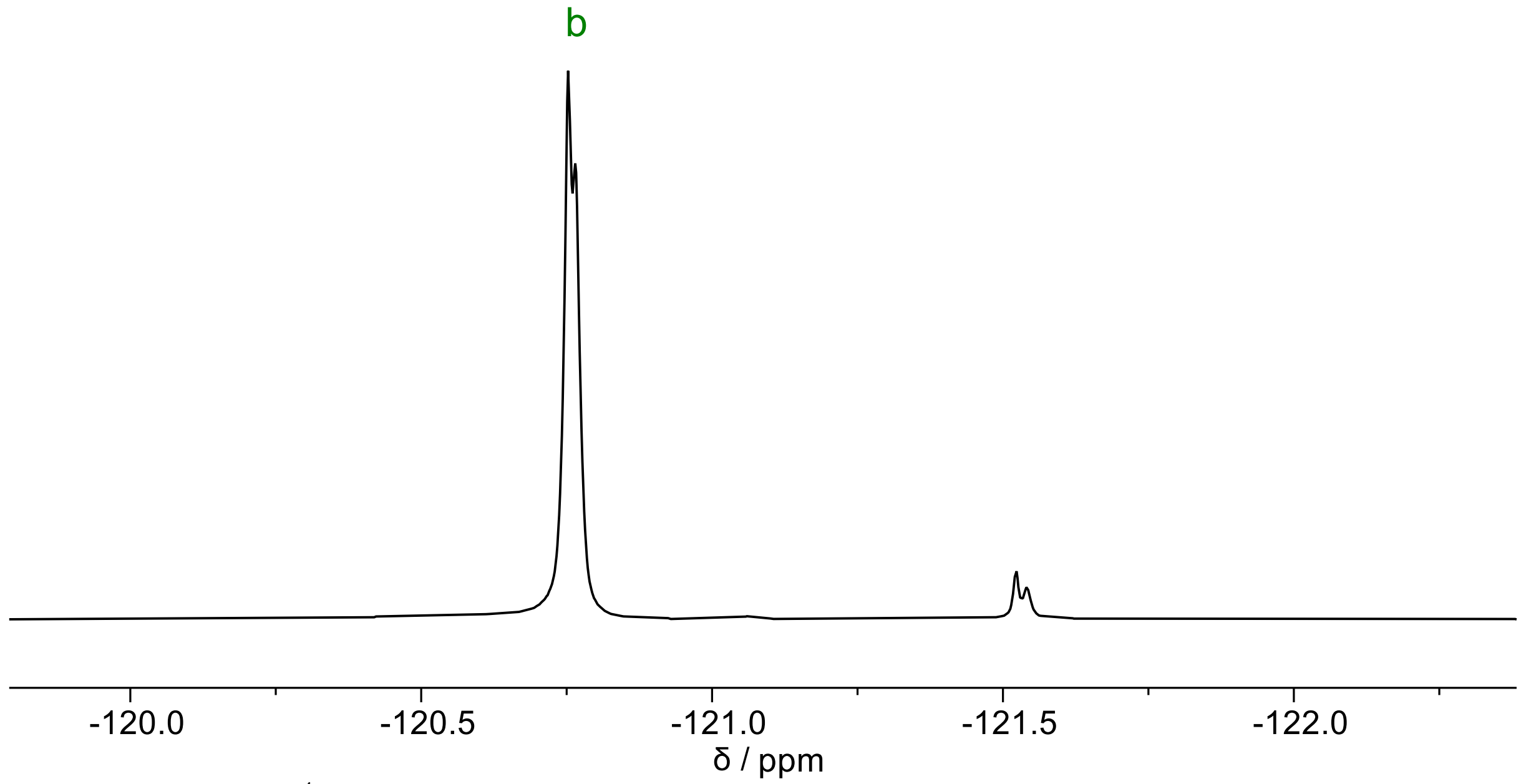

Figure S41. Partial [1]F NMR (565 MHz, 298 K, DMSO-d_6) spectrum of a mixture of ligand *Z*-**1** and *E*-**1** obtained after irradiating a solution of *E*-**1** with 530 nm light for 30 min..

4 Photoswitching properties of photoswitches 3, 4, 5 and ligand 1

4.1 Photoswitching properties of 3

4.1.1 Determining the PSS distributions of photoswitch 3 in DMSO-d_6 using ^{1}H and ^{19}F NMR spectroscopy

PSS distributions of photoswitch **3** were previously reported.[4]

4.1.1 UV-vis spectra of photoswitch 3 in DMSO after irradiation with different wavelengths

UV-vis spectra of photoswitch **3** in DMSO after irradiation with different wavelengths were previously reported.[4]

4.1.2 Determining the thermal half-life of photoswitch *Z*-3 in DMSO

The thermal stability of photoswitch **3** was previously reported by our group by fitting the change in absorbance at a single temperature using a mono-exponential curve and calculating the rate constant and thermal barrier. [4]

Detailed thermal stability measurements of photoswitch **3** at different temperatures were repeated here and then fitted using an Eyring plot.

UV-vis spectroscopy was used to determine the parameter for the thermal isomerization of *Z*-**3** to *E*-**3**. A sample of ligand **3** in DMSO (27 µM) was prepared and heated to 363 K in the UV-vis spectrophotometer. The sample was then irradiated at 363 K with 530 nm light for 10 minutes to enrich the sample with *Z*-**1** (PSS reached within 10 minutes). The light was turned off, and the absorbance at 358 nm was monitored every minute for 6 hours. The same experiment was repeated at 353 K and 343 K. The absorbance at 336 nm was plotted versus time, and the data were fit to a mono-exponential curve to determine the first-order rate constants of the thermal Z-**3** to *E*-**3** isomerization.

Model	Exponential		
Equation	y = y0 + A*exp(R0*x)		
Plot	363 K	353 K	343 K
y0	0.97027 ± 1.89736E-4	0.98587 ± 1.9266E-4	1.00251 ± 3.06414E-4
A	-0.5917 ± 0.00157	-0.59573 ± 8.13769E-4	-0.61094 ± 3.05947E-4
R0	-0.05395 ± 2.10003E-4	-0.02289 ± 5.29086E-5	-0.008 ± 1.26162E-5
Reduced Chi-Sqr	1.02904E-5	6.90671E-6	2.12262E-6
R-Square (COD)	0.99867	0.99957	0.99991
Adj. R-Square	0.99866	0.99957	0.99991

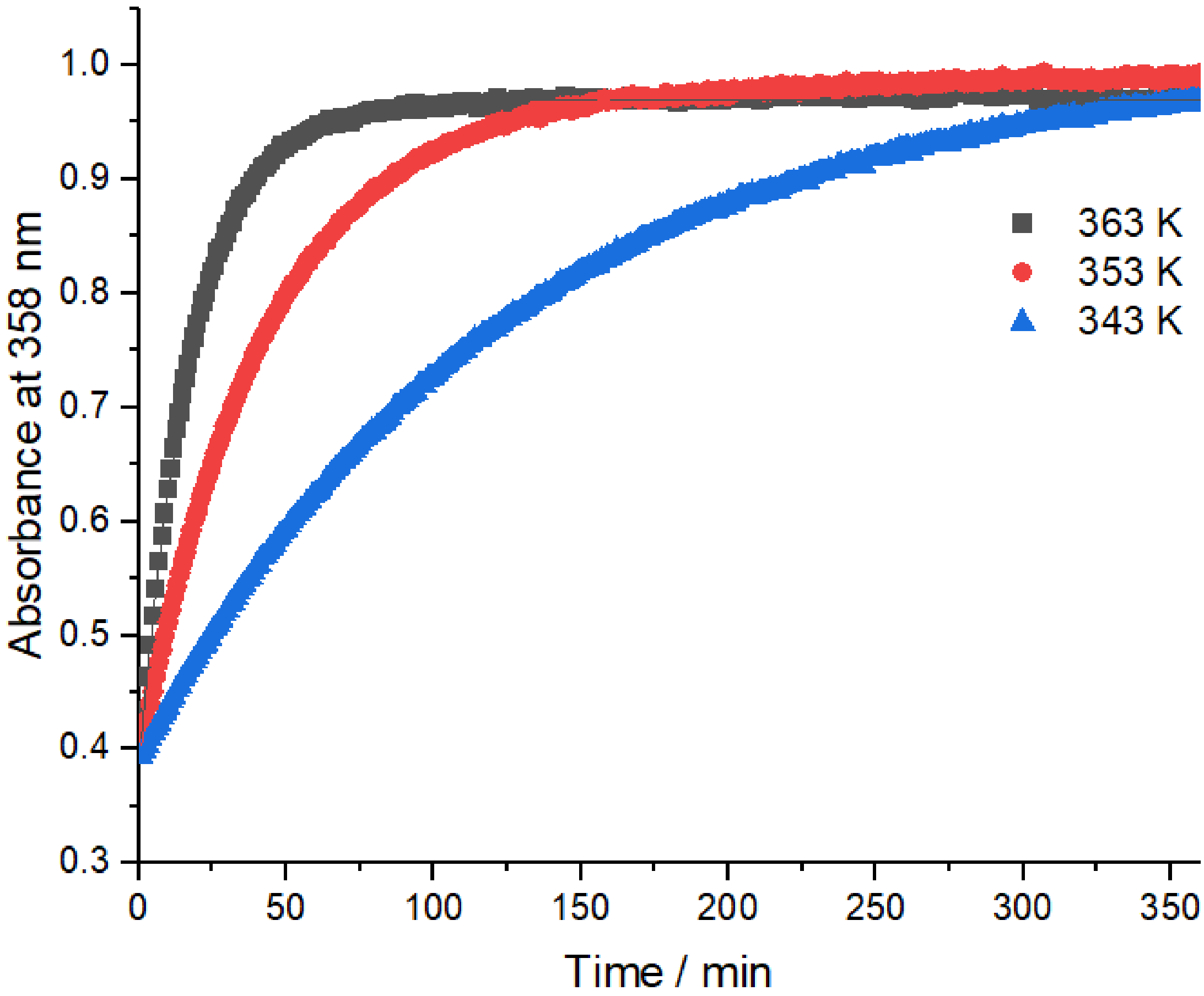

Figure S42. Thermal isomerization of *Z*-**3** to *E*-**3** in DMSO at 363, 353, and 343 K. Monitored over 6 hours by UV-vis spectroscopy.

Table S2. First-order rate constants for the thermal isomerization of *Z*-1 to *E*-1 at different temperatures.

**Temperature / K**	**363**	**353**	**343**
k/ min^{-1} × 10^{-2}	5.39 ± 0.02	2.29 ± 0.05	0.800 ± 0.01
k/ s^{-1} × 10^{-4}	8.99 ± 0.04	3.82 ± 0.01	1.33 ± 0.01
ln(k/T)	-12.91 ± 0.01	-13.74 ± 0.01	14.76 ± 0.01
1/T / K^{-1} × 10^{-3}	2.75 ± 0.01	2.84 ± 0.01	2.92 ± 0.01

The first-order rate constants of the thermal isomerization from *Z*-**3** to *E*-**3** are listed in Table S17. An Eyring plot (Figure S57) was constructed using ln(k/T) and 1/T. Based on the Eyring equation, the entropy of activation ($\Delta S^{\ddagger}$ = -40.4 J·mol^{-1}·K^{-1}) and the enthalpy of activation ($\Delta H^{\ddagger}$ = 95.9 kJ·mol^{-1}) were estimated.

Using $\Delta G^{\ddagger} = \Delta H^{\ddagger} - T\Delta S^{\ddagger}$ the Gibbs free energy of activation, $\Delta G^{\ddagger}$, was calculated for each temperature (363 K, 353 K and 343 K). We assumed that $\Delta H^{\ddagger}$ and $\Delta S^{\ddagger}$ remain constant across all temperatures, which is reasonable for intramolecular isomerization reactions, to extrapolate $\Delta G^{\ddagger}$ and the half-life for the thermal isomerization of photoswitch **3** at 298 K.

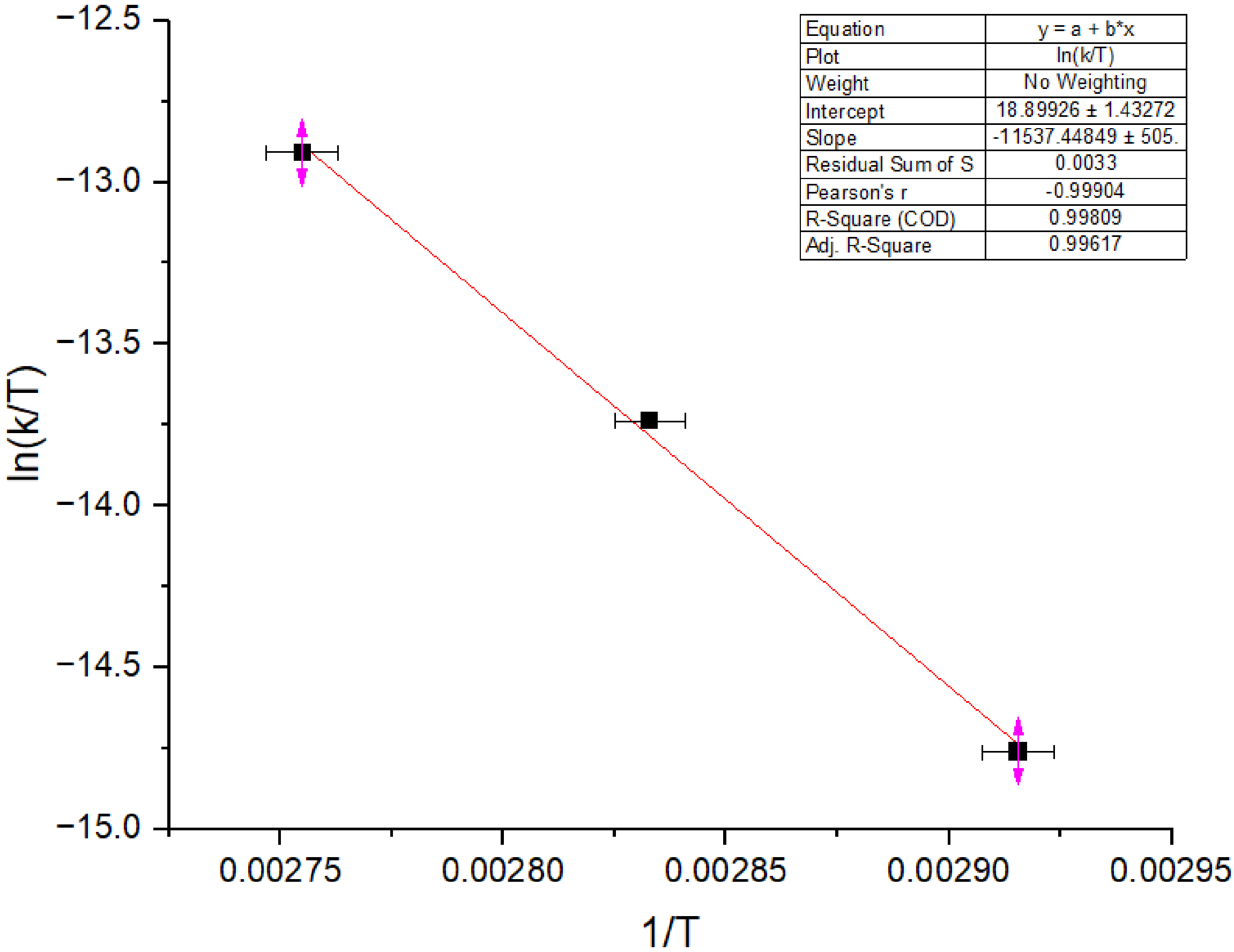

Figure S43. Eyring plot for the thermal isomerization of *Z*-**3** to *E*-**3** in DMSO. Rate constants were determined using UV-vis spectroscopy.

Table S3. $\Delta G^‡$ for thermal isomerisation of the *Z*-isomer of photoswitch **3** to the *E*-isomer at different temperatures in DMSO

**Temperature / K**	**363**	**353**	**343**
$\Delta G^‡$ / kJ·mol^{-1}	111 ± 4	110 ± 4	110 ± 4

Table S4. Thermodynamic parameters and half-life for the thermal isomerisation of the *Z*-isomer of photoswitch **3** to the *E*-isomer in DMSO at 298 K**.**

**$\Delta G^‡$ / kJ·mol^{-1}**	**$\Delta H^‡$ / kJ·mol^{-1}**	**$T\Delta S^‡$ / kJ·mol^{-1}**	**$t_{1/2}$ / days**
108 ± 4	96 ± 3	-12 ± 0.4	11

4.2 Photoswitching properties of 4

4.2.1 Determining the PSS distributions of photoswitch 4 in DMSO-d_6 using ^{1}H and ^{19}F NMR spectroscopy

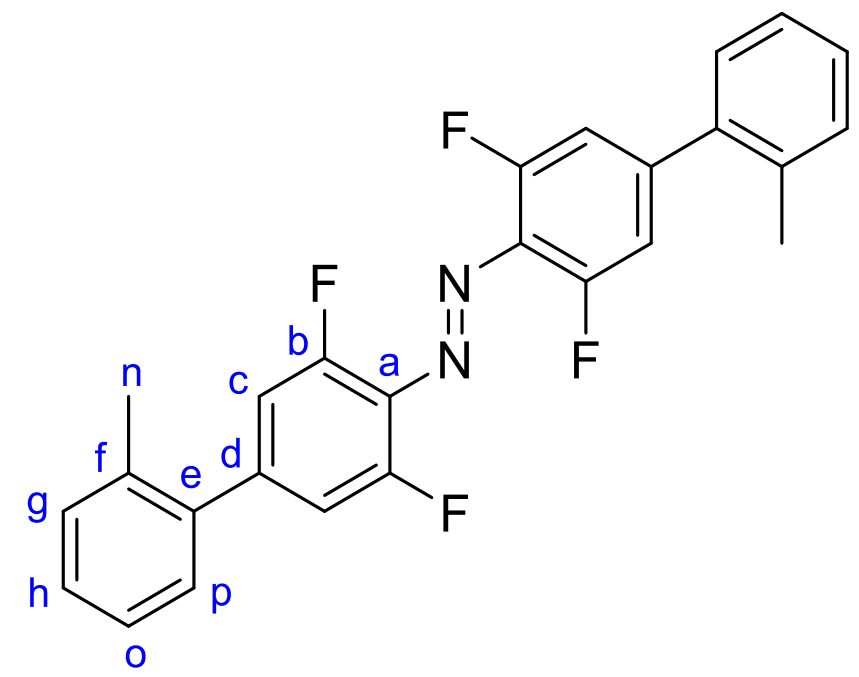

To determine the PSS distribution of photoswitch **4** in DMSO-d_6 ^{1}H and ^{19}F NMR spectroscopy was used.

An NMR sample of photoswitch **4** was prepared in DMSO-d_6 and heated with a heat gun (100 °C) for 5 minutes (Figure S44i) to convert all possible *Z*- to *E*-isomer. The sample was then irradiated with 530 nm light for 10 minutes (Figure S44iii), 20 minutes (Figure S44iiii), 30 minutes (Figure S44iiv), 40 minutes (Figure S44iv) and 50 minutes (Figure S44ivi). A photostationary state was reached after 30 minutes of irradiation. Next, the same sample was irradiated with 445 nm light for 10 minutes (Figure S45ii), 20 minutes (Figure S45iii), 30 minutes (Figure S45iv), 40 minutes (Figure S45v) and 50 minutes (Figure S45vi). The signal intensity only changed by 1% after the fourth irradiation period. Last, the sample was irradiated with 424 nm light for 10 minutes (Figure S46ii), 20 minutes (Figure S46iii), 30 minutes (Figure S46iv), 40 minutes (Figure S46v) and 50 minutes (Figure S46iv). The signal intensity again only changed by 1% after the fourth irradiation period.

The signal intensities of the H^n and F^b environments of photoswitch **4** were compared to calculate the PSS distribution of *E*-**4** and *Z*-**4** at the three different wavelengths (530 nm, 445 nm and 424 nm). These environments were selected, since they do not overlap with other signals in their *E*- or *Z*-form, allowing accurate determination of the PSS distribution. The signal intensities of H^n and F^b were measured after each irradiation period to calculate the percentages of the *E*-**4** and *Z*-**4**. The signal intensities and isomer percentages are presented in Table S5 and Table S6. The PSS distributions were determined to be PSS_{530} (12:88 *E*:*Z*), PSS_{445} (68:32 *E*:*Z*) and PSS_{424} (81:19 *E*:*Z*).

The PSS selectivity of photoswitch **4** (PSS_{530} 12:88 *E*:*Z*) improved compared to the unsubstituted photoswitch **3** (PSS_{530} 22:78 *E*:*Z*).[4]

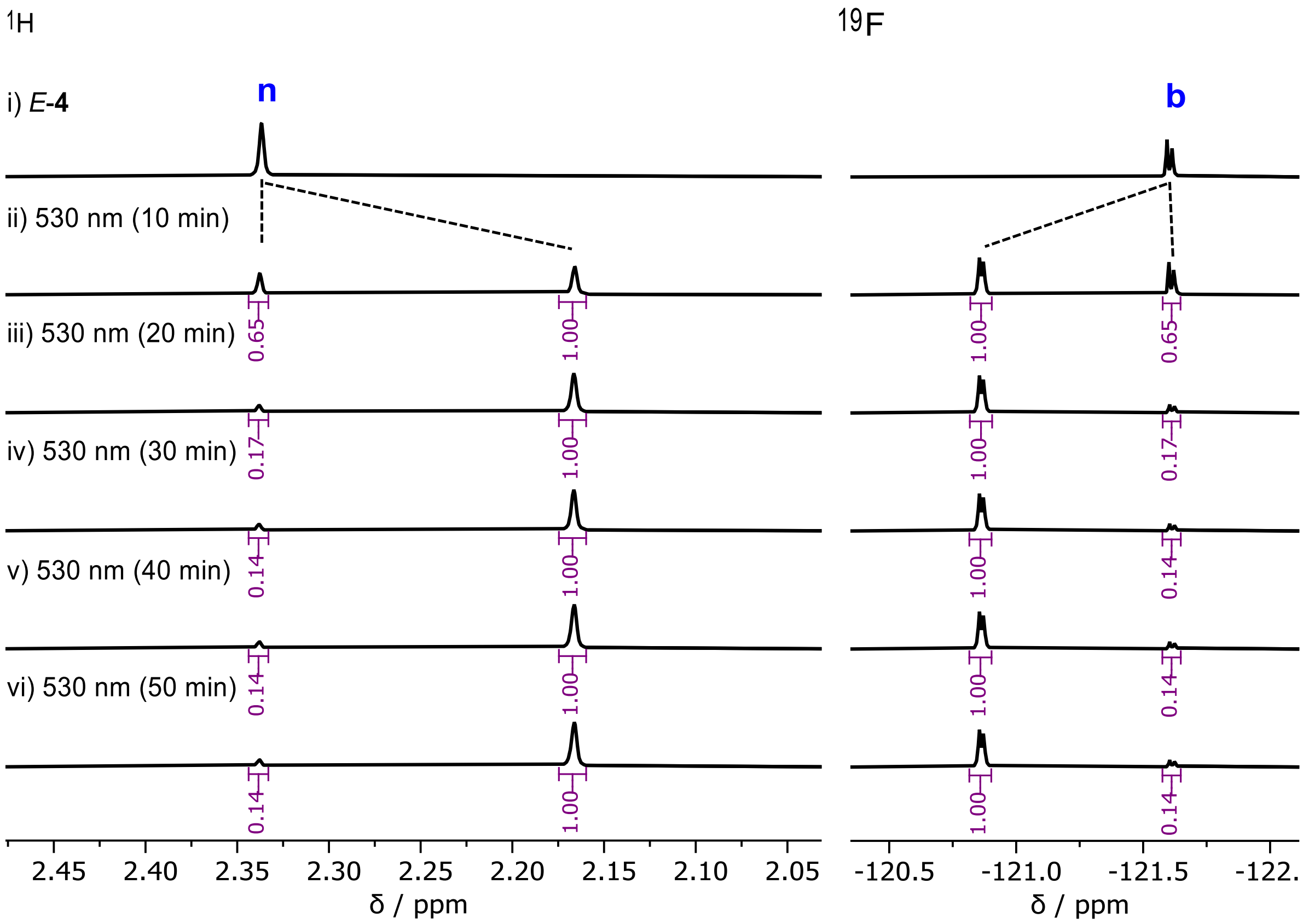

Figure S44. Partial ^{1}H NMR (600 MHz, 298K, DMSO-d_6) and ^{19}F NMR (565 MHz, 298K, DMSO-d_6) spectra of photoswitch **4** after i) thermal equilibration, ii) irradiation with 530 nm light for 10 minutes, iii) irradiation with 530 nm light for 20 minutes, iv) irradiation with 530 nm light for 30 minutes, v) irradiation with 530 nm light for 40 minutes, and vi) irradiation with 530 nm light for 50 minutes.

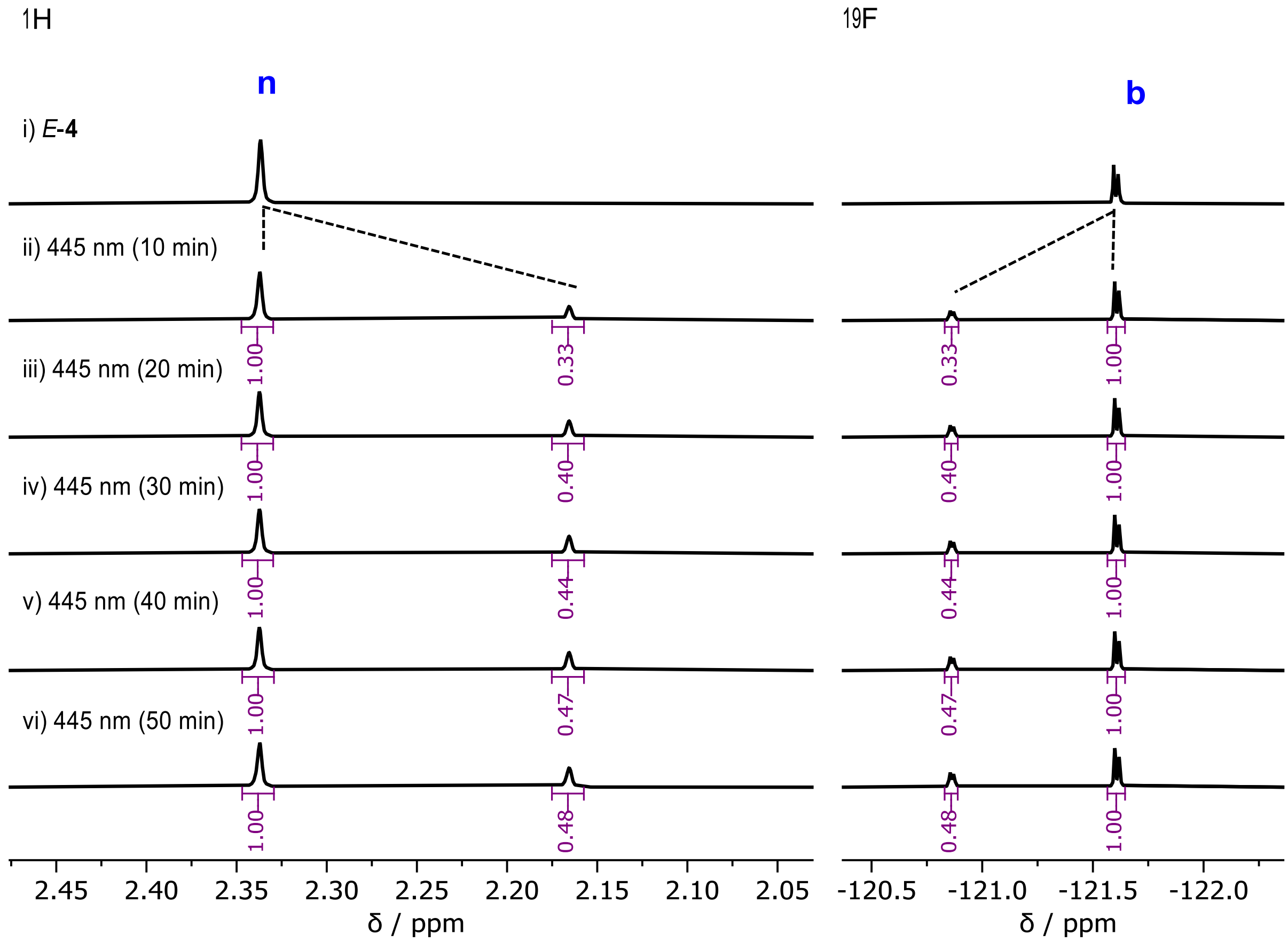

Figure S45. Partial ^{1}H NMR (600 MHz, 298K, DMSO-d_6) and ^{19}F NMR (565 MHz, 298K, DMSO-d_6) spectra of photoswitch **4** after i) thermal equilibration, ii) irradiation with 445 nm light for 10 minutes, iii) irradiation with 445 nm light for 20 minutes, iv) irradiation with 445 nm light for 30 minutes, v) irradiation with 445 nm light for 40 minutes, and vi) irradiation with 445 nm light for 50 minutes.

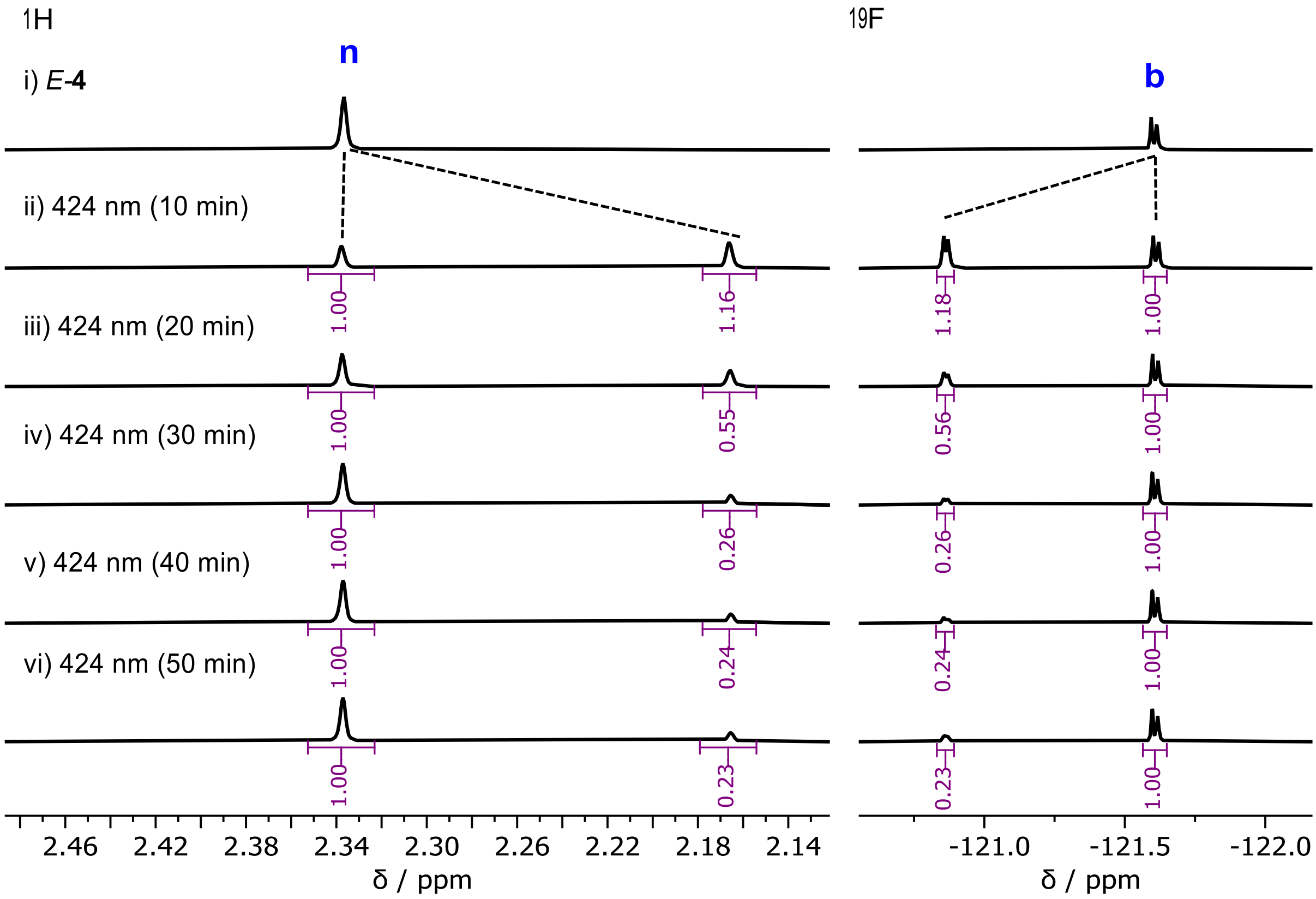

Figure S46. Partial ^{1}H NMR (600 MHz, 298K, DMSO-d_6) and ^{19}F NMR (565 MHz, 298K, DMSO-d_6) spectra of photoswitch **4** after i) thermal equilibration, ii) irradiation with 424 nm light for 10 minutes, iii) irradiation with 424 nm light for 20 minutes, iv) irradiation with 424 nm light for 30 minutes, v) irradiation with 424 nm light for 40 minutes, and vi) irradiation with 424 nm light for 50 minutes.

Table S5. ^{1}H NMR (600 MHz, 298K, DMSO-d_6) and ^{19}F (565 MHz, 298 K, DMSO-d_6) signal intensities and calculated isomer percentages for photoswitch **4** in DMSO-d_6 after irradiation with different wavelengths.

Environment H^n				
Irradiation	*E*-**4** (2.34 ppm)	*Z*-**4** (2.17 ppm)	%*E*-**4**	%*Z*-**4**
530 nm (10 min)	0.65	1.00	39.4	60.6
530 nm (20 min)	0.17	1.00	14.5	85.5
530 nm (30 min)	0.14	1.00	12.3	87.7
530 nm (40 min)	0.14	1.00	12.3	87.7
530 nm (50 min)	0.14	1.00	12.3	87.7
424 nm (10 min)	1.00	1.16	46.3	53.7
424 nm (20 min)	1.00	0.55	64.5	35.5
424 nm (30 min)	1.00	0.26	79.4	20.6
424 nm (40 min)	1.00	0.24	80.6	19.4
424 nm (50 min)	1.00	0.23	81.3	18.7
445 nm (10 min)	1.00	0.33	75.2	24.8
445 nm (20 min)	1.00	0.40	71.4	28.6
445 nm (30 min)	1.00	0.44	69.4	30.6
445 nm (40 min)	1.00	0.47	68.0	32.0
445 nm (50 min)	1.00	0.48	67.6	32.4
Environment F^b				
Irradiation	*E*-**4** (-121.60 ppm)	*Z*-**4** (-120.86 ppm)	%*E*-**4**	%*Z*-**4**
530 nm (10 min)	0.65	1.00	39.4	60.6
530 nm (20 min)	0.17	1.00	14.5	85.5
530 nm (30 min)	0.14	1.00	12.3	87.7
530 nm (40 min)	0.14	1.00	12.3	87.7
530 nm (50 min)	0.14	1.00	12.3	87.7
424 nm (10 min)	1.00	1.18	45.9	54.1
424 nm (20 min)	1.00	0.56	64.1	35.9
424 nm (30 min)	1.00	0.26	79.4	20.6
424 nm (40 min)	1.00	0.24	80.6	19.4
424 nm (50 min)	1.00	0.23	81.3	18.7
445 nm (10 min)	1.00	0.33	75.2	24.8
445 nm (20 min)	1.00	0.40	71.4	28.6
445 nm (30 min)	1.00	0.44	69.4	30.6
445 nm (40 min)	1.00	0.47	68.0	32.0
445 nm (50 min)	1.00	0.48	67.6	32.4

Table S6. Average PSS distribution of photoswitch **4** at different irradiation wavelengths.[a]

Irradiation wavelength	%*E*-**4**	%*Z*-**4**
530 nm	12	88
424 nm	81	19
445 nm	68	32

[a]PSS distributions determined from ^{1}H (600 MHz, 298 K, DMSO-d_6) and ^{19}F (565 MHz, 298 K, DMSO-d_6) NMR signal intensities for photoswitch **4** environments H^n and F^b.

4.2.2 UV-vis spectra of photoswitch 4 in DMSO after irradiation with different wavelengths

To measure the UV-vis spectra of photoswitch **4**, a sample of photoswitch **4** was prepared in DMSO (concentration = 25.0 µM). The sample was then placed in the UV-vis Instrument, where it was heated to 100 °C to convert all possible residual *Z*- to *E*-isomer. The sample of pure *E*-**4** was then cooled back 25 °C for the following measurements.

The UV-Vis spectrum of *E*-**4** was recorded before the sample was irradiated with 530 nm, 445 nm and 424 nm light. For each irradiation wavelength, the absorbance did not change after irradiation for 10 minutes indicating that the PSS had reached.

To calculate the UV-vis absorbance spectrum for the pure *Z*-**4** isomer, the UV-vis absorbance spectrum of *E*-**4** and the spectra at PSS_{530}, PSS_{445} and PSS_{424} were used in combination with the calculated PSS distributions determined from the ^{1}H and ^{19}F NMR spectra (see Section 4.2.1 for details). The UV-vis absorbance spectrum of *Z*-**4** was calculated using the following equation:

$$Abs_Z = \frac{Abs_{PSS} - (E \times Abs_E)}{Z}$$

Abs_Z = absorbance of the *Z*-isomer

Abs_{PSS} = absorbance at the relative PSS

E = relative abundance of the E-isomer

Abs_E = absorbance of the sample containing all *E*-isomer

Z = relative abundance of the Z-isomer

The absorbance of *Z*-**4** was calculated using the absorption spectra and relative abundance of the *E*-**4** and *Z*-**4** isomer at each PSS_{530}, PSS_{445}, and PSS_{424}. The three calculated spectra were averaged, and the standard deviation was calculated (Figure S47).

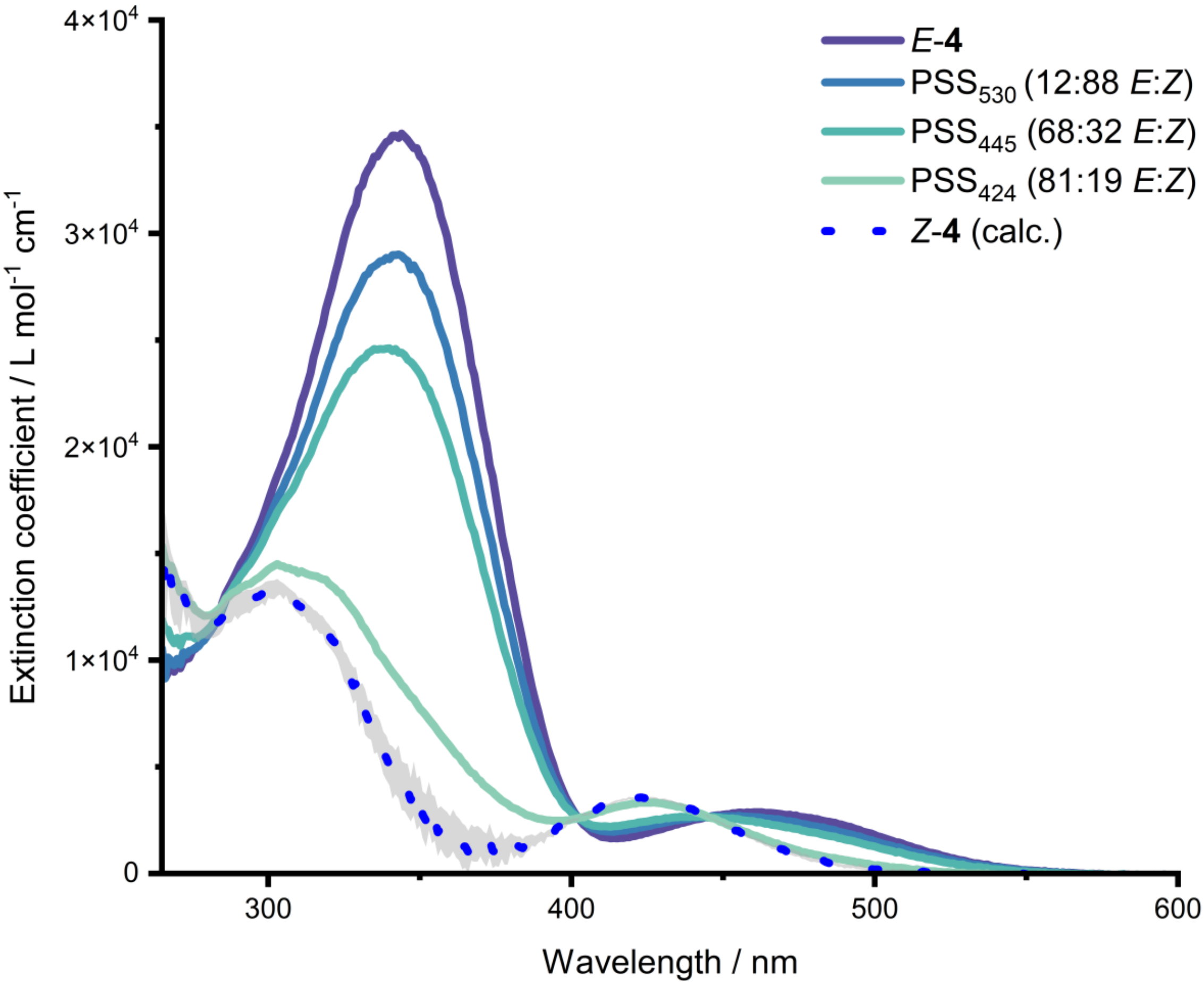

Figure S47. The UV-vis absorption spectra (DMSO-d_6, 298 K) of photoswitch **4** at PSS generated using LEDs with emission centred at various wavelengths. The spectrum for *Z*-**4** was calculated using the ^{1}H and ^{19}F NMR data together with absorption spectra solutions at PSS when irradiated with 530, 445 and 424 nm.

4.2.3 Determining the thermal half-life of photoswitch *Z*-4 in DMSO

UV-vis spectroscopy was used to determine the parameter for the thermal isomerization of *Z*-**4** to *E*-**4**. A sample of ligand **4** in DMSO (25 µM) was prepared and heated to 363 K in the UV-vis spectrophotometer. The sample was then irradiated at 363 K with 530 nm light for 10 minutes to enrich the sample with *Z*-**4** (PSS reached within 10 minutes). The light was turned off and the absorbance at 333 nm was monitored every minute for 6 hours. The same experiment was repeated at 353 K, 343 K and 333 K. The absorbance at 336 nm was plotted versus time, and the data were fit to a mono-exponential curve to determine the first-order rate constants of the thermal Z-**4** to *E*-**4** isomerization.

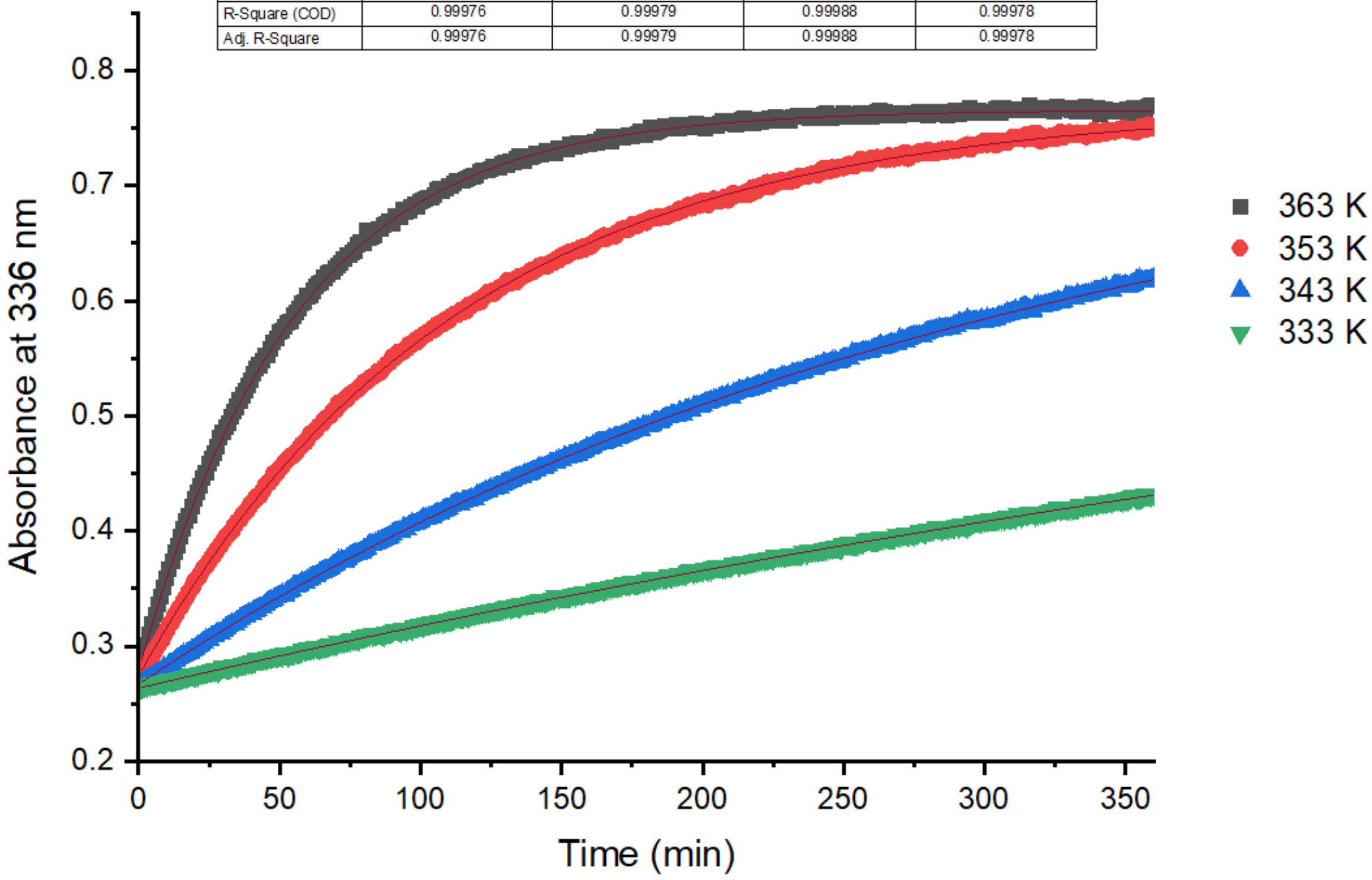

Model	Exponential			
Equation	y = y0 + A*exp(R0*x)			
Plot	363 K	353 K	343 K	333 K
y0	0.76575 ± 1.44625E-4	0.76974 ± 3.2625E-4	0.78059 ± 0.0012	0.74082 ± 0.00549
A	-0.48686 ± 4.74577E-4	-0.49565 ± 3.75953E-4	-0.51376 ± 0.00108	-0.47721 ± 0.00541
R0	-0.01808 ± 3.25687E-5	-0.0089 ± 2.01391E-5	-0.0032 ± 1.2749E-5	-0.0012 ± 1.70874E-5
Reduced Chi-Sqr	3.00309E-6	3.33511E-6	1.19381E-6	5.25154E-7
R-Square (COD)	0.99976	0.99979	0.99988	0.99978
Adj. R-Square	0.99976	0.99979	0.99988	0.99978

Figure S48. Thermal isomerization of *Z*-**4** to *E*-**4** in DMSO at 363, 353, 343 and 333 K. Monitored over 6 hours by UV-vis spectroscopy.

Table S7. First-order rate constants for the thermal isomerization of *Z*-**4** to *E*-**4** at different temperatures.

Temperature / K	363	353	343	333
k/ min^{-1} × 10^{-3}	18.1 ± 0.03	8.90 ± 0.02	3.2 ± 0.01	1.2 ± 0.02
k/ s^{-1} × 10^{-5}	30.13 ± 0.05	14.83 ± 0.03	5.33 ± 0.02	2.00 ± 0.03
ln(k/T)	-14.00 ± 0.03	-14.68 ± 0.03	-15.68 ± 0.07	-16.63 ± 0.10
1/T / K^{-1} × 10^{-3}	2.75 ± 0.01	2.84 ± 0.01	2.92 ± 0.01	3.00 ± 0.01

The first-order rate constants of the thermal back-isomerization from *Z*-**4** to *E*-**4** are listed in Table S17. An Eyring plot (Figure S57) was constructed using ln(k/T) and 1/T. Based on the Eyring equation, the entropy of activation ($\Delta S^\ddagger$ = -67.5 J·mol^{-1}·K^{-1}) and the enthalpy of activation ($\Delta H^\ddagger$ = 89.3 kJ·mol^{-1}) were estimated.

Using $\Delta G^\ddagger = \Delta H^\ddagger - T\Delta S^\ddagger$ the Gibbs free energy of activation, $\Delta G^\ddagger$, was calculated for each temperature (363 K, 353 K, 343 K and 333 K). We assumed that $\Delta H^\ddagger$ and $\Delta S^\ddagger$ remain constant across all temperatures, which is reasonable for intramolecular isomerization reactions, to extrapolate $\Delta G^\ddagger$ and the half-life for the thermal isomerization of photoswitch **4** at 298 K.

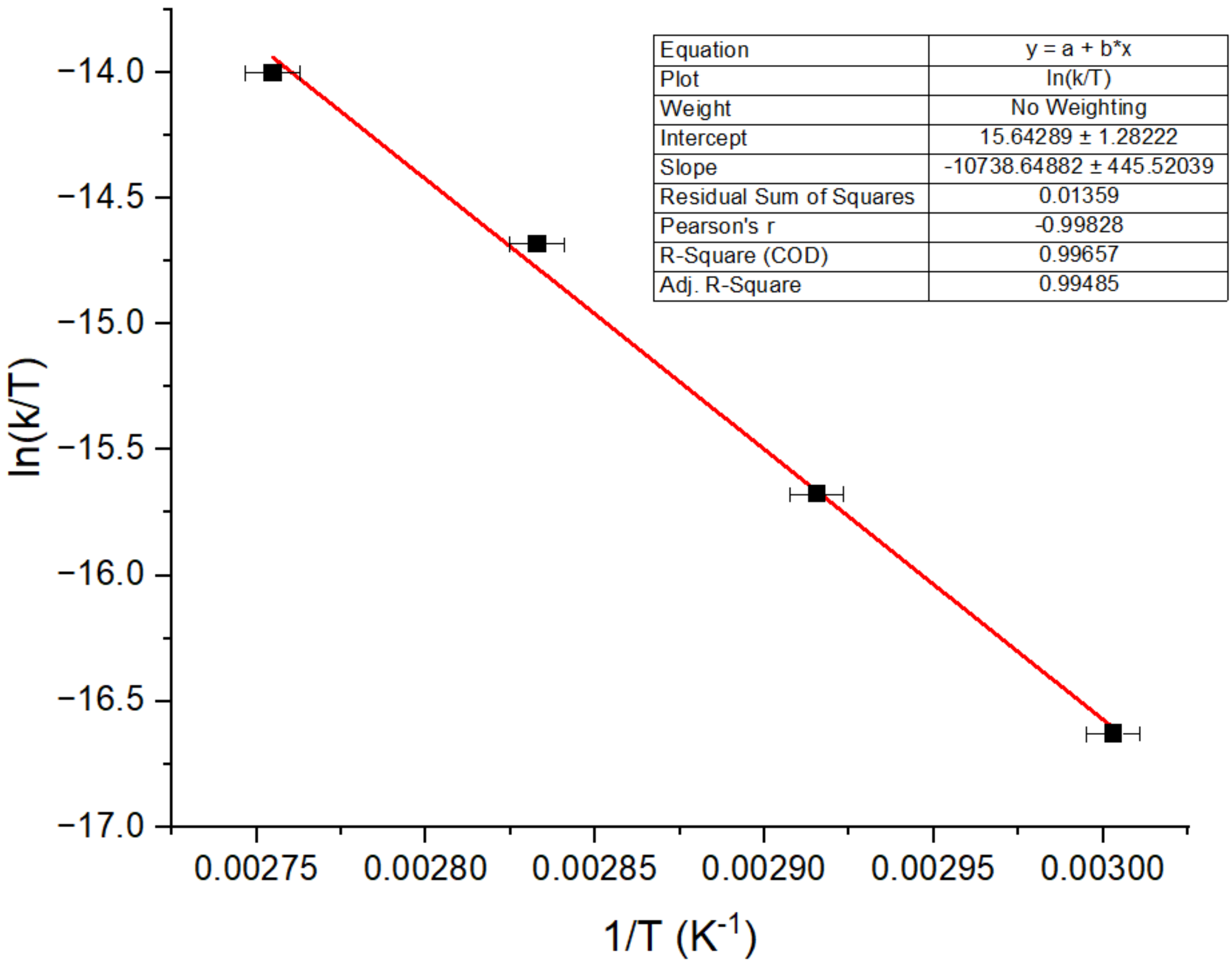

Figure S49. Eyring plot for the thermal isomerization of *Z*-**4** to *E*-**4** in DMSO. Rate constants were determined using UV-vis spectroscopy.

Table S8. $\Delta G^‡$ for thermal isomerisation of the *Z*-isomer of photoswitch **4** to the *E*-isomer at different temperatures in DMSO

**Temperature / K**	**363**	**353**	**343**	**333**
$\Delta G^‡$ / kJ·mol^{-1}	114 ± 4	113 ± 4	112 ± 4	112 ± 3

Table S9. Thermodynamic parameters and half-life for the thermal isomerisation of the *Z*-isomer of photoswitch **4** to the *E*-isomer in DMSO at 298 K**.**

**$\Delta G^‡$ / kJ·mol^{-1}**	**$\Delta H^‡$ / kJ·mol^{-1}**	**$T\Delta S^‡$ / kJ·mol^{-1}**	**$t_{1/2}$ / days**
109 ± 3	89 ± 3	-20 ± 0.4	19

4.3 Photoswitching properties of 5

4.3.1 Determining the PSS distributions of photoswitch 5 in DMSO-d_6 using ^{1}H and ^{19}F NMR spectroscopy

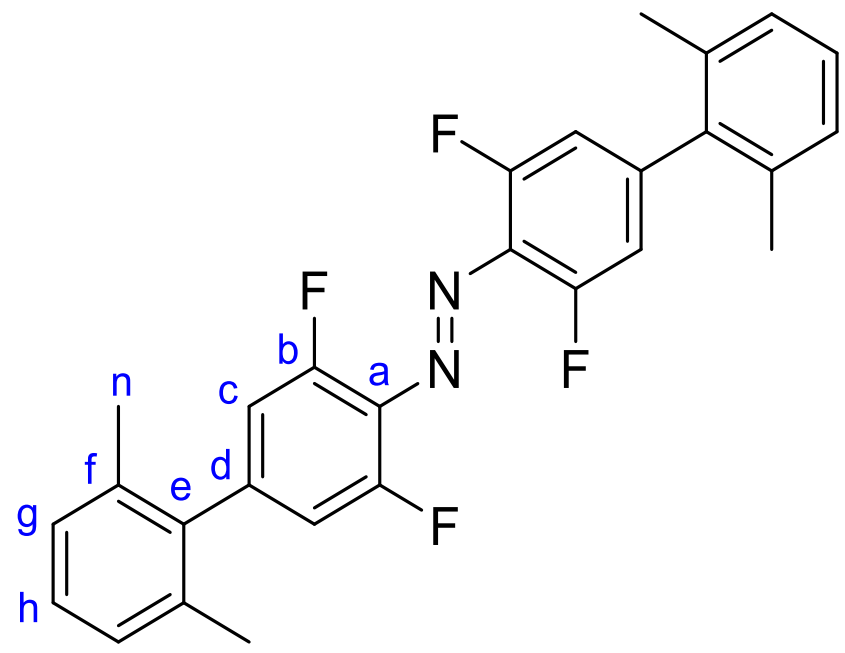

To determine the PSS distribution of photoswitch **5** in DMSO-d_6 ^{1}H and ^{19}F NMR spectroscopy was used.

An NMR sample of photoswitch **5** was prepared in DMSO-d_6 and heated with a heat gun (100 °C) for 5 minutes (Figure S50i) to convert all possible *Z*- to *E*- isomer. The sample was then irradiated with 530 nm light for 10 minutes (Figure S46ii) and 20 minutes (Figure S46iii). A photostationary state was reached after the first irradiation period. Next, the same sample was irradiated with 445 nm light for 10 minutes (Figure S46iv) and 20 minutes Figure S46v). The signal intensities in the NMR spectra did not change after the second irradiation period, indicating that a PSS was generated within 10 minutes. Last, the sample was irradiated with 424 nm light for 10 minutes (Figure S46vi) and 20 minutes (Figure S46vii), again generating a PSS after 10 minutes of irradiation.

The signal intensities of the H^{n} and F^{b} environments of photoswitch **5** were compared to calculate the PSS distribution of *E*-**5** and *Z*-**5** at the three different wavelengths (530 nm, 445 nm and 424 nm). These environments were selected, since they do not overlap with other signals in their *E*- or *Z*-form, allowing accurate determination of the PSS distribution. The signal intensities of H^{n} and F^{b} were measured after each irradiation period to calculate the percentages of the *E*-**5** and *Z*-**5**. The signal intensities and the isomer percentages are presented in Table S10 and Table S11. The PSS distribution was determined to be PSS_{530} (7:93 *E*:*Z*), PSS_{445} (57:43 *E*:*Z*) and PSS_{424} (79:21 *E*:*Z*).

The PSS selectivity of photoswitch **5** (PSS_{530} 7:93 *E*:*Z*) has improved compared to both, the mono-methylated photoswitch **4** (PSS_{530} 12:88 *E*:*Z,* see section 4.2.1) and the unsubstituted photoswitch **3** (PSS_{530} 22:78 *E*:*Z*).[4]

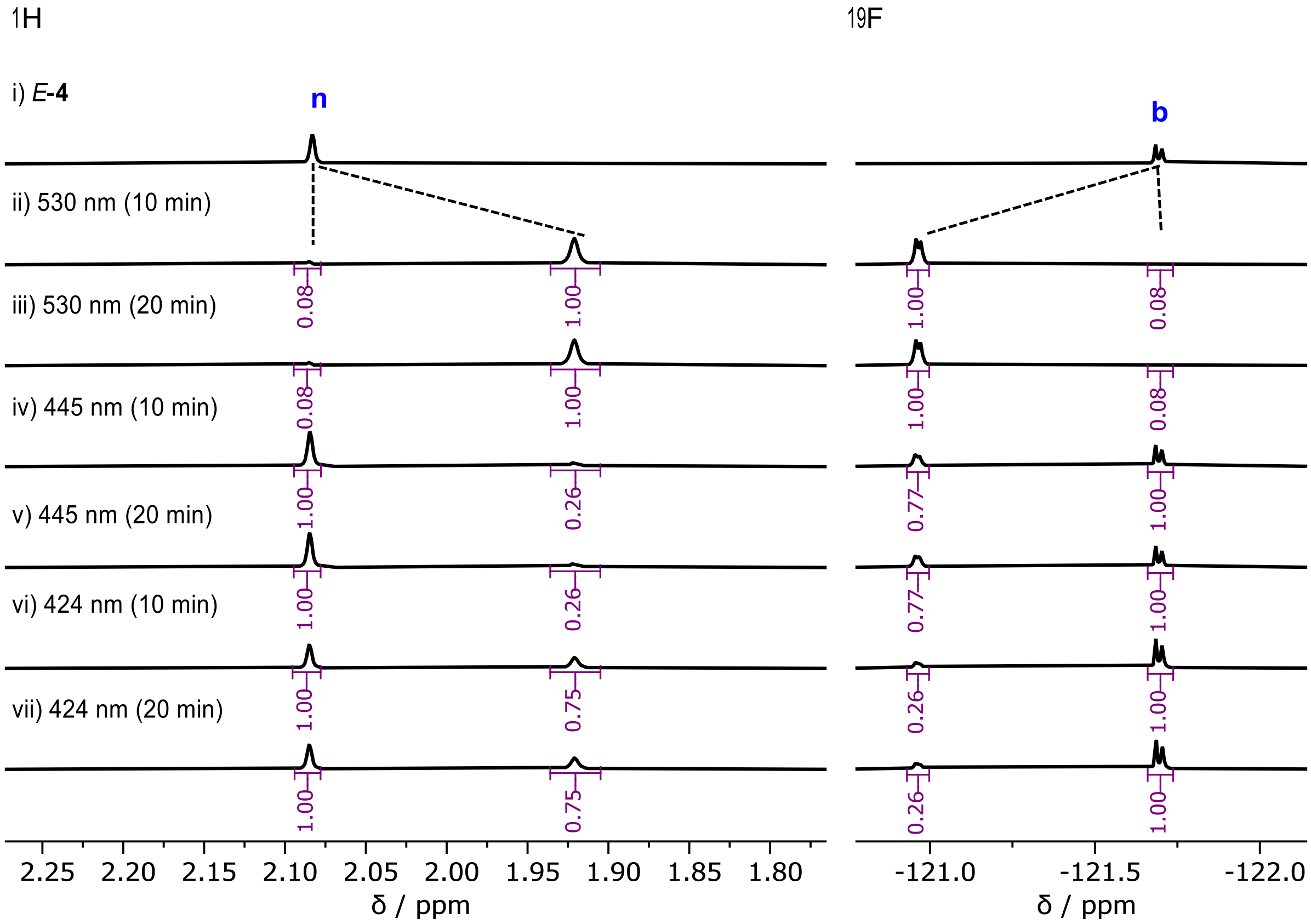

Figure S50. Partial ^{1}H NMR (600 MHz, 298K, DMSO-d_6) and ^{19}F NMR (565 MHz, 298K, DMSO-d_6) spectra of photoswitch **5** after i) thermal equilibration, ii) irradiation with 530 nm light for 10 minutes, iii) irradiation with 530 nm light for 20 minutes, iv) irradiation with 445 nm light for 10 minutes, v) irradiation with 445 nm light for 20 minutes, vi) irradiation with 424 nm light for 10 minutes and vii) irradiation with 424 nm light for 20 minutes.

Table S10. ^{1}H NMR (600 MHz, 298K, DMSO-d_6) and ^{19}F (575 MHz, 298 K, DMSO-d_6) signal intensities and calculated isomer percentages for photoswitch **5** in DMSO-d_6 after irradiation with different wavelengths.

Environment H^n				
Irradiation	*E*-**5** (2.09 ppm)	*Z*-**5** (1.92 ppm)	%*E*-**5**	%*Z*-**5**
530 nm (10 min)	0.08	1.00	7%	93%
530 nm (20 min)	0.08	1.00	7%	93%
424 nm (10 min)	1.00	0.26	79%	21%
424 nm (20 min)	1.00	0.26	79%	21%
445 nm (10 min)	1.00	0.75	57%	43%
445 nm (20 min)	1.00	0.75	57%	43%
Environment F^b				
Irradiation	*E*-**5** (-121.70 ppm)	*Z*-**5** (-120.96 ppm)	%*E*-**5**	%*Z*-**5**
530 nm (10 min)	0.08	1.00	7%	93%
530 nm (20 min)	0.08	1.00	7%	93%
424 nm (10 min)	1.00	0.26	79%	21%
424 nm (20 min)	1.00	0.26	79%	21%
445 nm (10 min)	1.00	0.77	56%	44%
445 nm (20 min)	1.00	0.77	56%	44%

Table S11. Average PSS distribution of photoswitch **5** at different irradiation wavelengths.

Irradiation wavelength	%*E*-**5**	%*Z*-**5**
530 nm	7%	93%
424 nm	79%	21%
445 nm	57%	43%

[a]PSS distributions determined from ^{1}H (600 MHz, 298 K, DMSO-d_6) and ^{19}F (565 MHz, 298 K, DMSO-d_6) NMR signal intensities for photoswitch **5** environments H^n and F^b.

4.3.2 UV-vis spectra of photoswitch 5 in DMSO after irradiation with different wavelengths

To measure the UV-vis spectra of photoswitch **5**, a sample of photoswitch **5** was prepared in DMSO (concentration = 25.0 μM). The sample was then placed in the UV-vis Instrument, where it was heated to 100 °C to convert all possible residual *Z*- to *E*-isomer. The sample of pure *E*-**5** was then cooled back 25 °C for the following measurements.

The UV-Vis spectrum of *E*-**5** was recorded before the sample was irradiated with 530 nm, 445 nm and 424 nm light. For each irradiation wavelength, the absorbance did not change after irradiation for 10 minutes, indicating that the PSS had reached.

To calculate the UV-vis absorbance spectrum for the pure *Z*-**5** isomer, the UV-vis absorbance spectrum of *E*-**5** and the spectra at PSS_{530}, PSS_{445} and PSS_{424} were used in combination with the calculated PSS distributions determined from the ^{1}H and ^{19}F NMR spectra (see section 4.3.1 for details). The UV-vis absorbance spectrum of *Z*-**5** was calculated using the following equation:

$$Abs_Z = \frac{Abs_{PSS} - (E \times Abs_E)}{Z}$$

Abs_Z = absorbance of the *Z*-isomer

Abs_{PSS} = absorbance at the relative PSS

E = relative abundance of the E-isomer

Abs_E = absorbance of the sample containing all *E*-isomer

Z = relative abundance of the Z-isomer

The absorbance of *Z*-**5** was calculated using the absorption spectra and relative abundance of the *E*-**5** and *Z*-**5** isomer at each PSS_{530}, PSS_{445}, and PSS_{424}. The three calculated spectra were averaged, and the standard deviation was calculated (Figure S51).

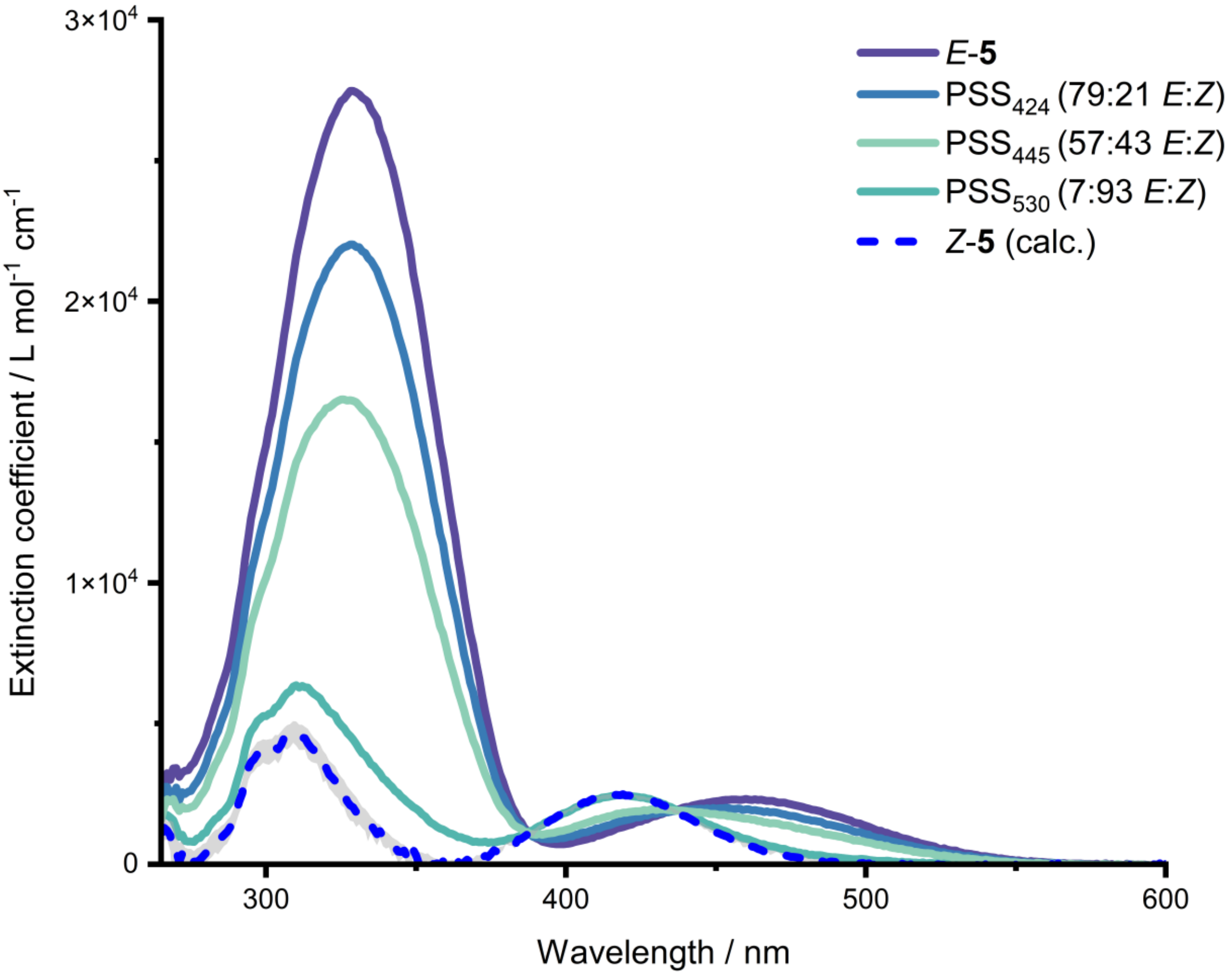

Figure S51. The UV-vis absorption spectra (DMSO-d_6, 298 K) of photoswitch **5** at PSS generated using LEDs with emission centred at various wavelengths. The spectrum for *Z*-**5** was calculated using the ^{1}H and ^{19}F NMR data together with absorption spectra solutions at PSS when irradiated with 530, 445 and 424 nm.

4.3.3 Determining the thermal half-life of photoswitch *Z*-5 in DMSO

UV-vis spectroscopy was used to determine the parameter for the thermal isomerization of *Z*-**5** to *E*-**5**. A sample of ligand **5** in DMSO (25 µM) was prepared and heated to 363 K in the UV-vis spectrophotometer. The sample was then irradiated at 363 K with 530 nm light for 10 minutes to enrich the sample with *Z*-**5** (PSS reached within 10 minutes). The light was turned off and the absorbance at 333 nm was monitored every minute for 6 hours. The same experiment was repeated at 363 K, 353 K and 343 K. The absorbance at 336 nm was plotted versus time, and the data were fit to a mono-exponential curve to determine the first-order rate constants of the thermal Z-**5** to *E*-**5** isomerization.

Model	Exponential		
Equation	y = y0 + A*exp(R0*x)		
Plot	363 K	353 K	343 K
y0	1.96128 ± 0.00338	1.93165 ± 0.00827	1.78364 ± 0.03625
A	-1.55579 ± 0.00279	-1.54382 ± 0.00799	-1.40356 ± 0.03609
R0	-0.00491 ± 2.31768E-5	-0.0018 ± 1.30949E-5	-6.34462E-4 ± 1.83383E-5
Reduced Chi-Sqr	4.71851E-5	5.76392E-6	1.76007E-6
R-Square (COD)	0.99964	0.99987	0.99974
Adj. R-Square	0.99964	0.99987	0.99974

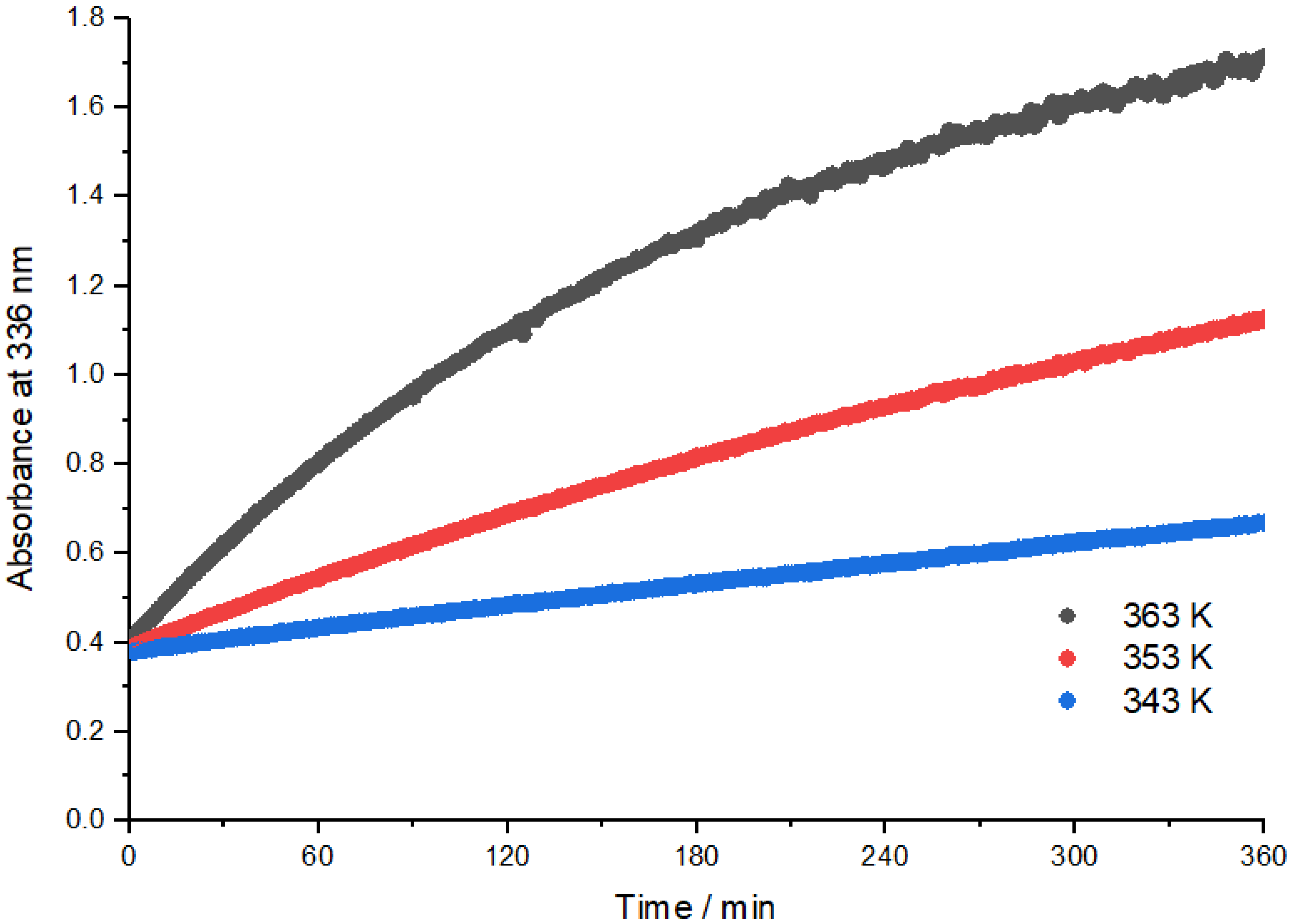

Figure S52. Thermal isomerization of *Z*-**5** to *E*-**5** in DMSO at 363, 353 and 343 K. Monitored over 6 hours by UV-vis spectroscopy.

Table S12. First-order rate constants for the thermal isomerization of *Z*-**5** to *E*-**5** at different temperatures.

**Temperature / K**	**363**	**353**	**343**
k/ $min^{-1} \times 10^{-3}$	4.74 ± 0.03	2.28 ± 0.04	0.777 ± 0.01
k/ $s^{-1} \times 10^{-5}$	7.90 ± 0.05	3.80 ± 0.07	1.29 ± 0.19
ln(k/T)	-15.34 ± 0.04	-16.04 ± 0.05	-17.09 ± 0.22
1/T / $K^{-1} \times 10^{-3}$	2.75 ± 0.01	2.84 ± 0.01	2.92 ± 0.01

The first-order rate constants of the thermal back-isomerization from *Z*-**5** to *E*-**5** are listed in Table S17. An Eyring plot (Figure S57) was constructed using ln(k/T) and 1/T. Based on the Eyring equation, the entropy of activation ($\Delta S^{\ddagger}$ = -39.7 $J \cdot mol^{-1} \cdot K^{-1}$) and the enthalpy of activation ($\Delta H^{\ddagger}$ = 103.5 $kJ \cdot mol^{-1}$) were estimated.

Using $\Delta G^{\ddagger} = \Delta H^{\ddagger} - T\Delta S^{\ddagger}$ the Gibbs free energy of activation, $\Delta G^{\ddagger}$, was calculated for each temperature (363 K, 353 K and 343 K). We assumed that $\Delta H^{\ddagger}$ and $\Delta S^{\ddagger}$ remain constant across all temperatures, which is reasonable for intramolecular isomerization reactions, to extrapolate $\Delta G^{\ddagger}$ and the half-life for the thermal isomerization of photoswitch **5** at 298 K.

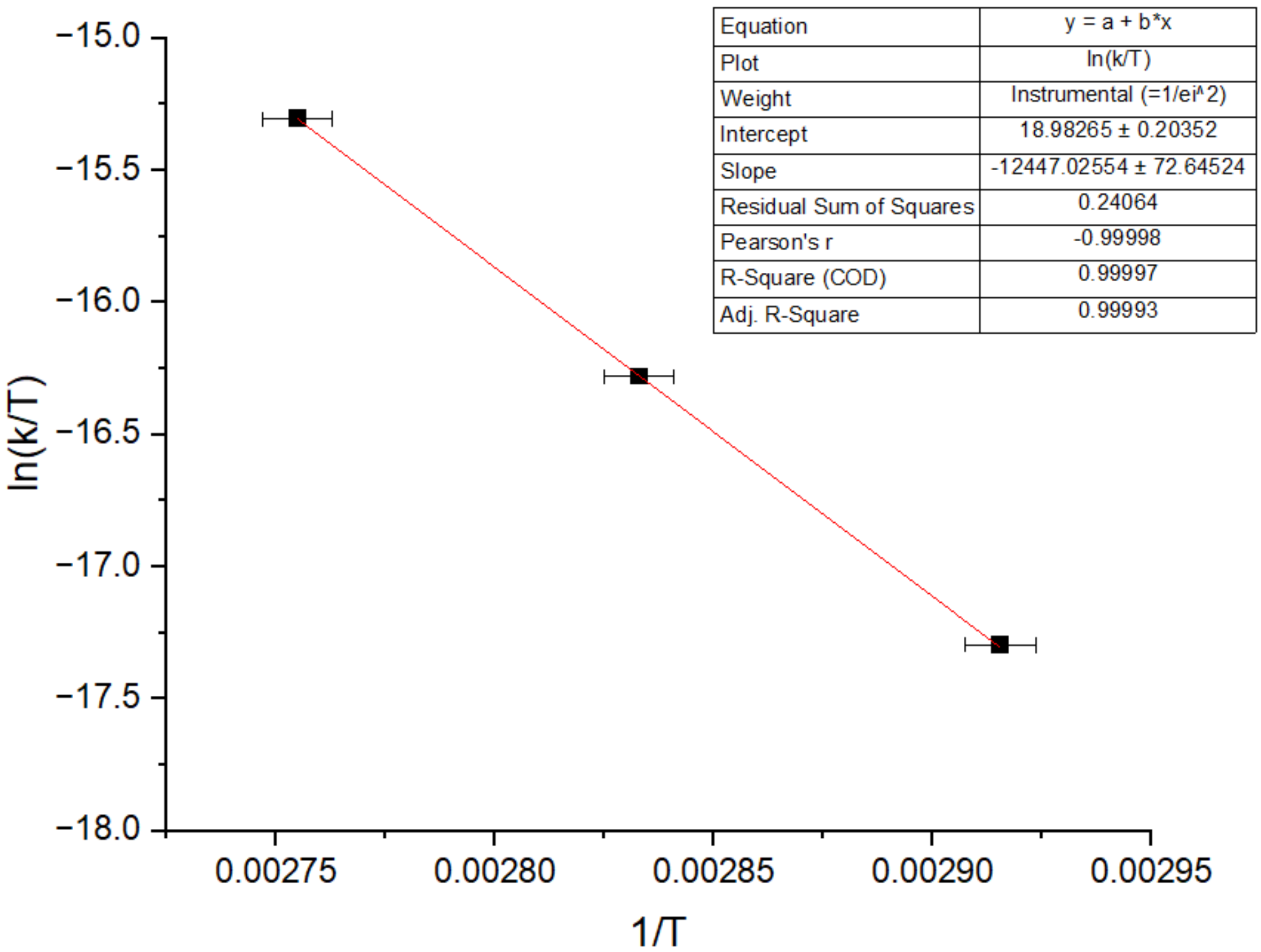

Figure S53. Eyring plot for the thermal isomerization of *Z*-**5** to *E*-**5** in DMSO. Rate constants were determined using UV-vis spectroscopy.

Table S13. $\Delta G^{\ddagger}$ for thermal isomerisation of the *Z*-isomer of photoswitch **5** to the *E*-isomer at different temperatures in DMSO

**Temperature / K**	**363**	**353**	**343**
$\Delta G^{\ddagger}$ / kJ·mol^{-1}	118 ± 1	118 ± 1	117 ± 1

Table S14. Thermodynamic parameters and half-life for the thermal isomerisation of the *Z*-isomer of photoswitch **5** to the *E*-isomer in DMSO at 298 K**.**

**$\Delta G^{\ddagger}$ / kJ·mol^{-1}**	**$\Delta H^{\ddagger}$ / kJ·mol^{-1}**	**$T\Delta S^{\ddagger}$ / kJ·mol^{-1}**	**$t_{1/2}$ / days**
116 ± 0.9	104 ± 0.5	-12 ± 0.4	209

4.4 Photoswitching properties of ligand 1

4.4.1 Determining the PSS distributions of ligand 1 in DMSO-d_6 using ^{1}H and ^{19}F NMR spectroscopy

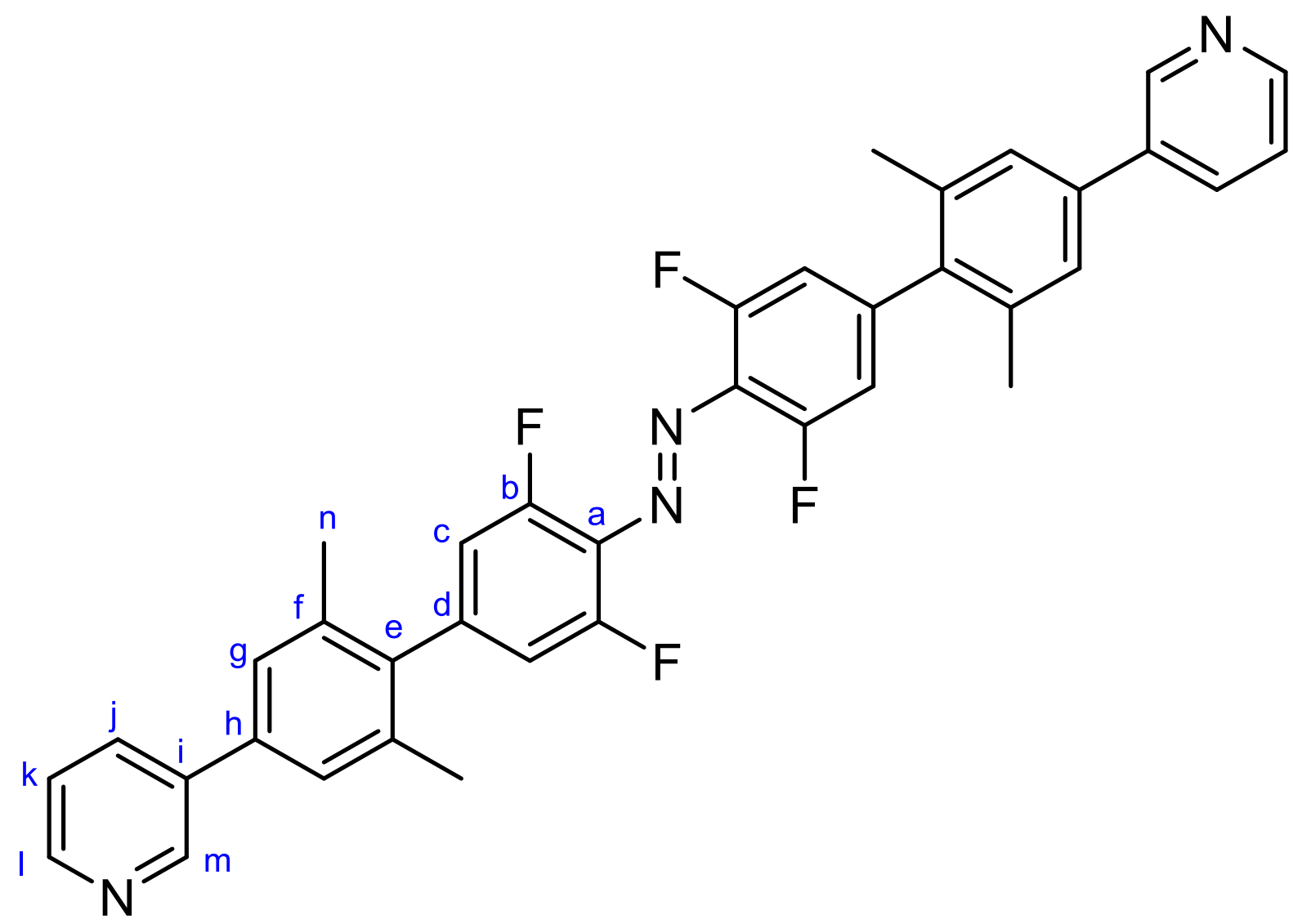

To determine the PSS distribution of ligand **1** in DMSO-d_6 ^{1}H and ^{19}F NMR spectroscopy was used.

An NMR sample of ligand **1** was prepared in DMSO-d_6 and heated with a heat gun (100 °C) for 5 minutes (Figure S54i) to convert all possible *Z*- to *E*- isomer. The sample was then irradiated with 530 nm light for 10 minutes (Figure S54ii), 20 minutes (Figure S54iii) and 30 minutes (Figure S54iv). A photostationary state was reached after the second irradiation period. Next, the same sample was irradiated with 445 nm light for 10 minutes (Figure S54v), 20 minutes (Figure S54vi) and 30 minutes (Figure S54vii). Last, the sample was irradiated with 424 nm light for 10 minutes (Figure S54viii), 20 minutes (Figure S54ix) and 30 minutes (Figure S54x), again generating a PSS after 20 minutes of irradiation.

The signal intensities of the H^c, H^n and F^b environments of photoswitch **1** were compared to calculate the PSS distribution of *E*-**1** and *Z*-**1** at the three different wavelengths (530 nm, 445 nm and 424 nm). These environments were selected, since they do not overlap with other signals in their *E*- or *Z*-form, allowing accurate determination of the PSS distribution. The signal intensities of H^c, H^n and F^b were measured after each irradiation period to calculate the percentages of the *E*-**1** and *Z*-**1**. The signal intensities and the isomer percentages are presented in Table S15 and Table S16. The PSS distribution was determined to be PSS_{530} (8:92 *E*:*Z*), PSS_{445} (57:43 *E*:*Z*) and PSS_{424} (79:21 *E*:*Z*).

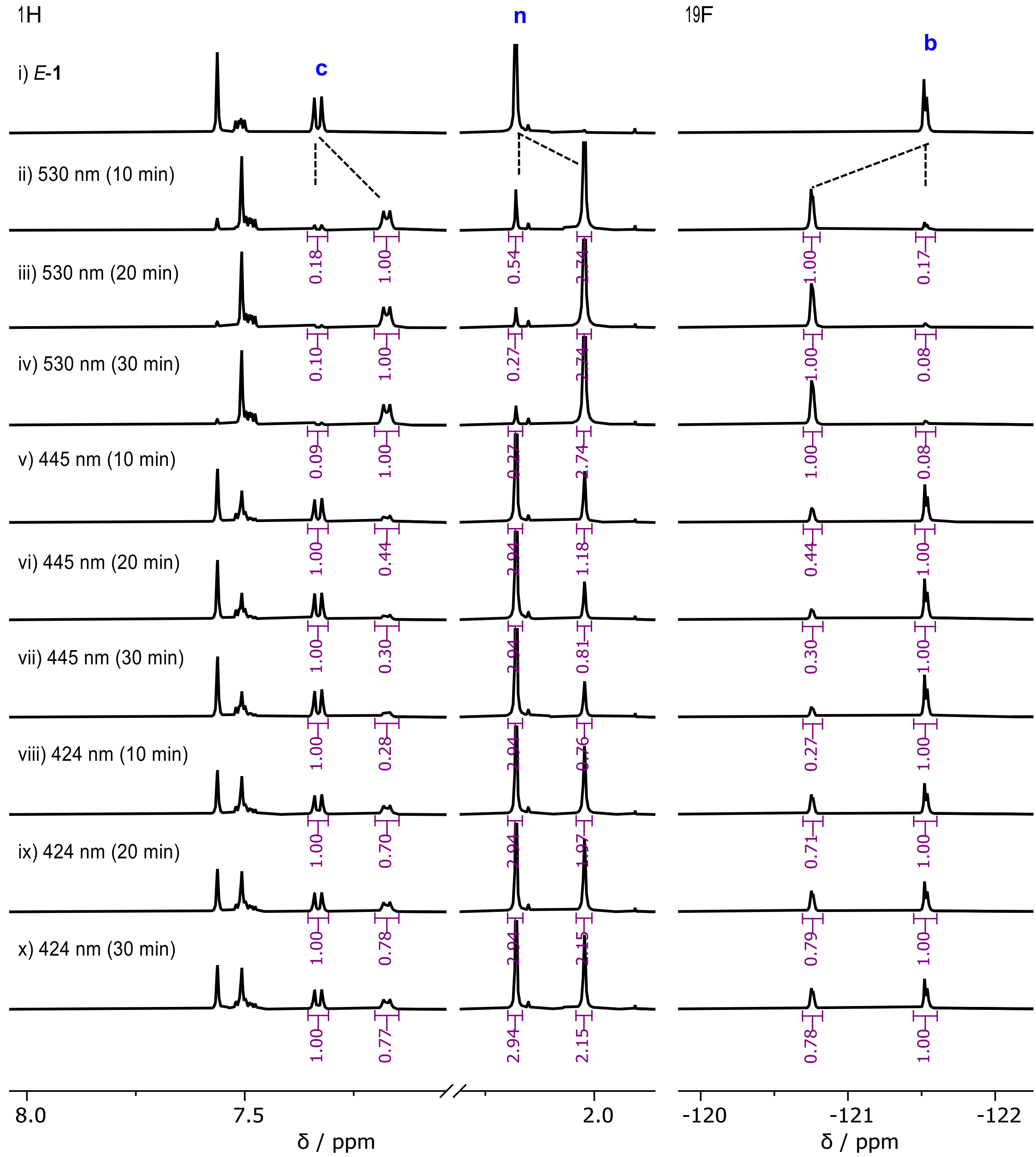

Figure S54. Partial ^{1}H and ^{19}F NMR (600 MHz, 565 MHz, 298K, DMSO-d_6) spectra of ligand **1** after i) thermal equilibration, ii) irradiation with 530 nm light for 10 minutes, iii) irradiation with 530 nm light for 20 minutes, iv) irradiation with 530 nm light for 30 minutes, v) irradiation with 445 nm light for 10 minutes, vi) irradiation with 445 nm light for 20 minutes, vii) irradiation with 445 nm light for 30 minutes, viii) irradiation with 424 nm light for 10 minutes, ix) irradiation with 424 nm light for 20 minutes and x) irradiation with 424 nm light for 30 minutes.

Table S15. ^{1}H NMR (600 MHz, 298K, DMSO-d_6) and ^{19}F (565 MHz, 298 K, DMSO-d_6) signal intensities and calculated isomer percentages for ligand **1** in DMSO-d_6 after irradiation with different wavelengths.

Environment H^n				
Irradiation	*E*-**1** (2.18 ppm)	*Z*-**1** (2.02 ppm)	%*E*-**1**	%*Z*-**1**
530 nm (10 min)	0.54	2.74	16.5	83.5
530 nm (20 min)	0.27	2.74	9.0	91.0
530 nm (30 min)	0.27	2.74	9.0	91.0
424 nm (10 min)	2.94	1.18	71.4	28.6
424 nm (20 min)	2.94	0.81	78.4	21.6
424 nm (30 min)	2.94	0.76	79.5	20.5
445 nm (10 min)	2.94	1.97	59.9	40.1
445 nm (20 min)	2.94	2.15	57.8	42.2
445 nm (30 min)	2.94	2.15	57.8	42.2
Environment H^c				
Irradiation	*E*-**1** (7.34 ppm)	*Z*-**1** (7.17 ppm)	%*E*-**1**	%*Z*-**1**
530 nm (10 min)	0.18	1.00	15.3	84.7
530 nm (20 min)	0.10	1.00	9.1	90.9
530 nm (30 min)	0.09	1.00	8.3	91.7
424 nm (10 min)	1.00	0.44	69.4	30.6
424 nm (20 min)	1.00	0.30	76.9	23.1
424 nm (30 min)	1.00	0.28	78.1	21.9
445 nm (10 min)	1.00	0.70	58.8	41.2
445 nm (20 min)	1.00	0.78	56.2	43.8
445 nm (30 min)	1.00	0.77	56.5	43.5
Environment F^b				
Irradiation	*E*-**1** (-121.53 ppm)	*Z*-**1** (-120.76 ppm)	%*E*-**1**	%*Z*-**1**
530 nm (10 min)	0.17	1.00	14.5	85.5
530 nm (20 min)	0.08	1.00	7.4	92.6
530 nm (30 min)	0.08	1.00	7.4	92.6
424 nm (10 min)	1.00	0.44	69.4	30.6
424 nm (20 min)	1.00	0.30	76.9	23.1
424 nm (30 min)	1.00	0.27	78.7	21.3
445 nm (10 min)	1.00	0.71	58.5	41.5
445 nm (20 min)	1.00	0.79	55.9	44.1
445 nm (30 min)	1.00	0.78	56.2	43.8

Table S16. Average PSS distribution of ligand **1** at different irradiation wavelengths.[a]

Irradiation wavelength	%*E*-**1**	%*Z*-**1**
530 nm	8	92
424 nm	79	21
445 nm	57	43

[a]PSS distributions determined from ^{1}H (600 MHz, 298 K, DMSO-d_6) and ^{19}F (565 MHz, 298 K, DMSO-d_6) NMR signal intensities for ligand **1** environments H^c, H^n and F^b.

4.4.2 UV-vis spectra of ligand 1 in DMSO after irradiation with different wavelengths

To measure the UV-vis spectra of ligand **1**, a sample of ligand **1** was prepared in DMSO (concentration = 25.0 µM). The sample was then placed in the UV-vis Instrument, where it was heated to 100 °C to convert all possible residual *Z*- to *E*-isomer. The sample of pure *E*-**1** was then cooled back 25 °C for the following measurements.

The UV-Vis spectrum of *E*-**1** was recorded before the sample was irradiated with 530 nm, 445 nm and 424 nm light. For each irradiation wavelength, the absorbance did not change after irradiation for 10 minutes indicating that the PSS had reached.

To calculate the UV-vis absorbance spectrum for the pure *Z*-**1** isomer, the UV-vis absorbance spectrum of *E*-**1** and the spectra at PSS_{530}, PSS_{445} and PSS_{424} were used in combination with the calculated PSS distributions determined from the ^{1}H and ^{19}F NMR spectra (see Section 4.4 for details). The UV-vis absorbance spectrum of *Z*-**1** was calculated using the following equation:

$$Abs_Z = \frac{Abs_{PSS} - (E \times Abs_E)}{Z}$$

Abs_Z = absorbance of the *Z*-isomer

Abs_{PSS} = absorbance at the relative PSS

E = relative abundance of the E-isomer

Abs_E = absorbance of the sample containing all *E*-isomer

Z = relative abundance of the Z-isomer

The absorbance of *Z*-**1** was calculated using the absorption spectra and relative abundance of the *E*-**1** and *Z*-**1** isomer at each PSS_{530}, PSS_{445}, and PSS_{424}. The three calculated spectra were averaged, and the standard deviation was calculated (Figure S55a).

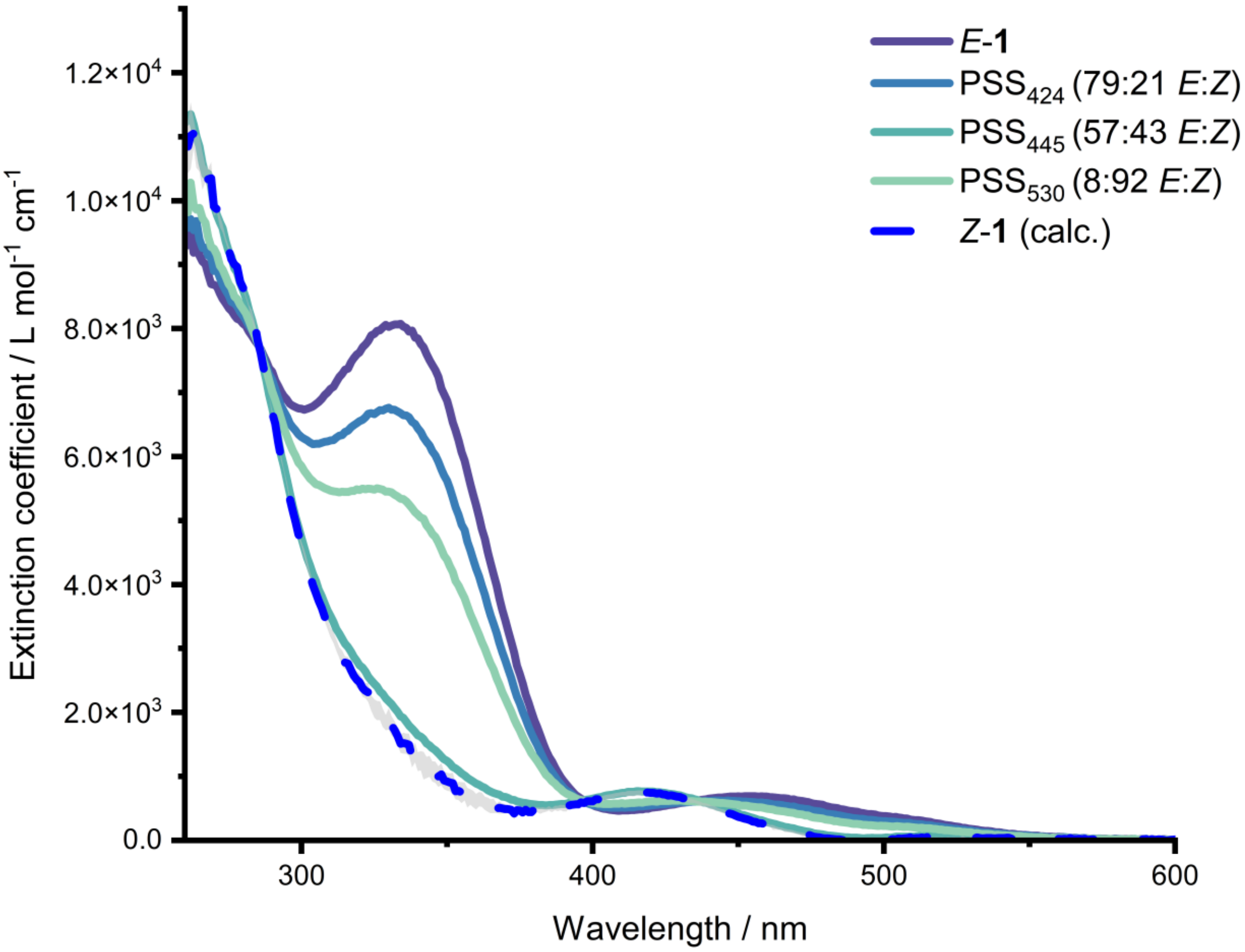

Figure S55. The UV-vis absorption spectra (DMSO-*d*6, 298 K) of ligand **1** at PSS generated using LEDs with emission centered at various wavelengths. The spectrum for *Z*-**1** was calculated using the ^{1}H and ^{19}F NMR data together with absorption spectra solutions at PSS when irradiated with 530, 445 and 424 nm.

4.4.3 Determining the thermal half-life of ligand *Z*-1 in DMSO

UV-vis spectroscopy was used to determine the parameter for the thermal isomerization of *Z*-**1** to *E*-**1**. A sample of ligand **1** in DMSO (5.2 µM) was prepared and heated to 363 K in the UV-vis spectrophotometer. The sample was then irradiated at 363 K with 530 nm light for 10 minutes to enrich the sample with *Z*-**1** (PSS reached within 10 minutes). The light was turned off and the absorbance at 333 nm was monitored every minute for 10 hours. The same experiment was repeated at 353 K and 343 K. The absorbance at 336 nm was plotted versus time, and the data were fit to a mono-exponential curve to determine the first-order rate constants of the thermal *Z*-**1** to *E*-**1** isomerization.

Model	Exponential		
Equation	y = y0 + A*exp(R0*x)		
Plot	363 K	353 K	343 K
y0	0.53071 ± 1.71932E-4	0.5282 ± 0.00111	0.45728 ± 0.00282
A	-0.38759 ± 2.25436E-4	-0.38376 ± 9.89408E-4	-0.31945 ± 0.00275
R0	-0.00582 ± 9.51693E-6	-0.00203 ± 1.02332E-5	-9.1514E-4 ± 1.05365E-5
Reduced Chi-Sqr	1.97471E-6	2.08323E-6	5.94602E-7
R-Square (COD)	0.9998	0.99965	0.99961
Adj. R-Square	0.9998	0.99965	0.99961

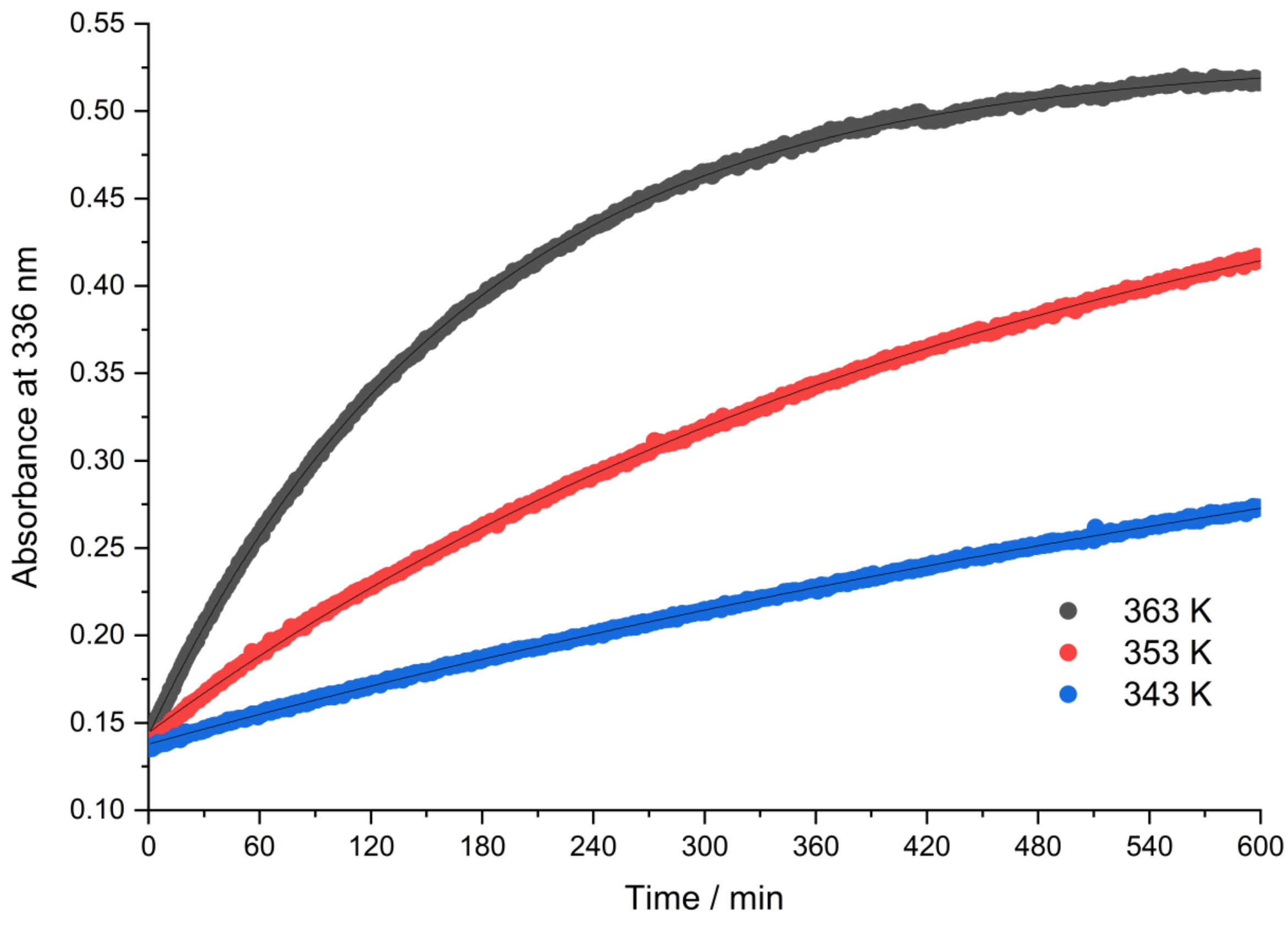

Figure S56. Thermal isomerization of *Z*-**1** to *E*-**1** in DMSO at 363, 353, and 343 K. Monitored over 10 hours by UV-vis spectroscopy.

Table S17. First-order rate constants for the thermal isomerization of *Z*-**1** to *E*-**1** at different temperatures.

**Temperature / K**	**363**	**353**	**343**
k/ $min^{-1} \times 10^{-3}$	5.82 ± 0.01	2.03 ± 0.01	0.915 ± 0.01
k/ $s^{-1} \times 10^{-5}$	9.70 ± 0.02	3.38 ± 0.02	1.53 ± 0.02
ln(k/T)	-15.14 ± 0.01	-16.16 ± 0.01	-16.93 ± 0.01
1/T / $K^{-1} \times 10^{-3}$	2.75 ± 0.01	2.84 ± 0.01	2.92 ± 0.01

The first-order rate constants of the thermal back-isomerization from *Z*-**1** to *E*-**1** are listed in Table S17. An Eyring plot (Figure S57) was constructed using ln(k/T) and 1/T. Based on the Eyring equation, the entropy of activation ($\Delta S^{\ddagger}$ = -62.6 $J{\cdot}mol^{-1}{\cdot}K^{-1}$) and the enthalpy of activation ($\Delta H^{\ddagger}$ = 94.7 $kJ{\cdot}mol^{-1}$) were estimated.

Using $\Delta G^{\ddagger} = \Delta H^{\ddagger} - T\Delta S^{\ddagger}$ the Gibbs free energy of activation, $\Delta G^{\ddagger}$, was calculated for each temperature (363 K, 353 K and 343 K). We assumed that $\Delta H^{\ddagger}$ and $\Delta S^{\ddagger}$ remain constant across all temperatures, which is reasonable for intramolecular isomerization reactions, to extrapolate $\Delta G^{\ddagger}$ and the half-life for the thermal isomerization of ligand **1** at 298 K.

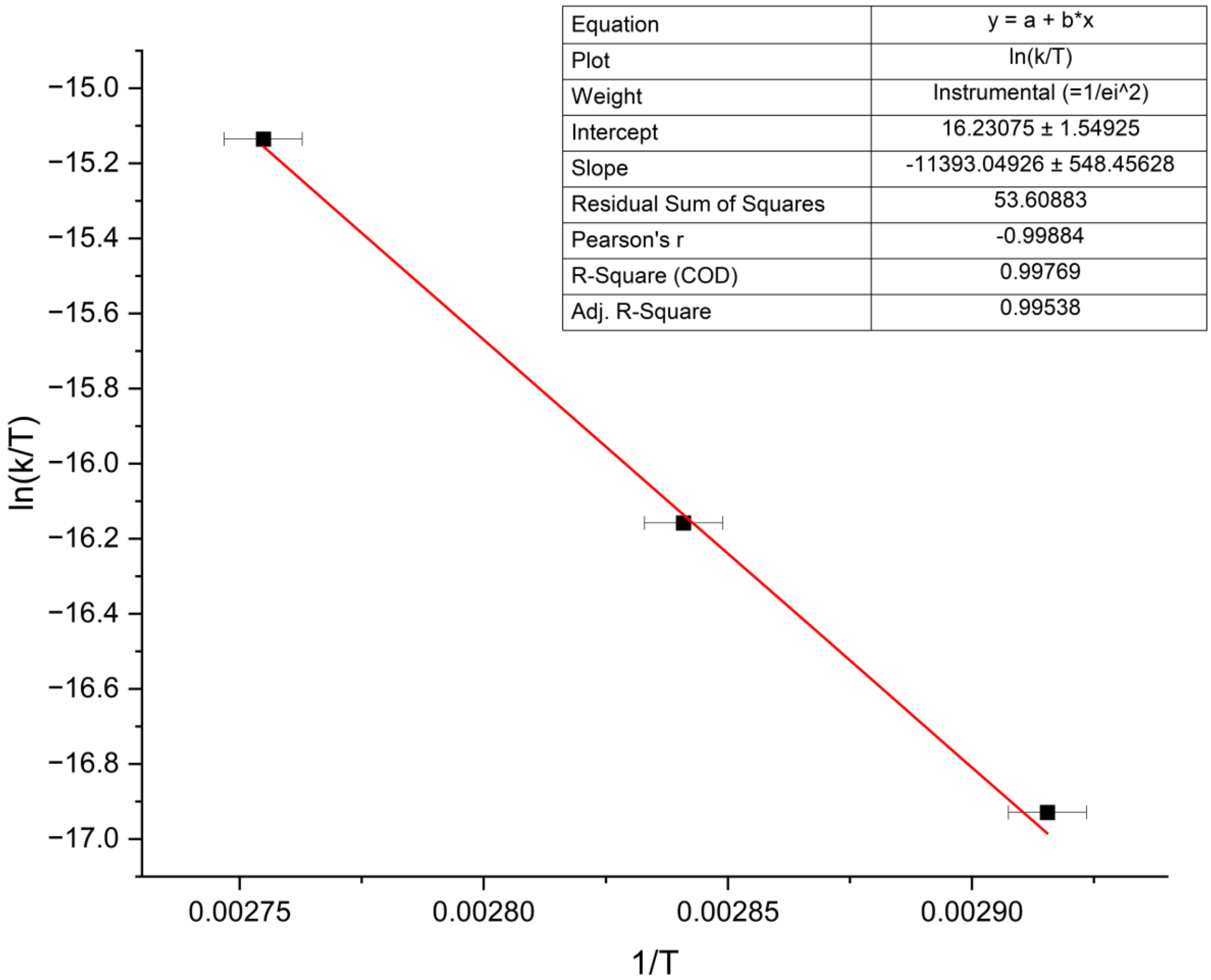

Figure S57. Eyring plot for the thermal isomerization of *Z*-**9** to *E*-**9** in DMSO. Rate constants were determined using UV-vis spectroscopy.

Table S18. ΔG‡ for thermal isomerisation of the *Z*-isomer of ligand **1** to the *E*-isomer at different temperatures in DMSO.

**Temperature / K**	**363**	**353**	**343**
ΔG‡ / kJ·mol^{-1}	117 ± 8	117 ± 7	116 ± 7

Table S19. Thermodynamic parameters and half-life for the thermal isomerisation of the *Z*-isomer of ligand **1** to the *E*-isomer in DMSO at 298 K**.**

**ΔG‡ / kJ·mol^{-1}**	**ΔH‡ / kJ·mol^{-1}**	**TΔS‡ / kJ·mol^{-1}**	**$t_{1/2}$ / days**
113 ± 7	95 ± 4	-19 ± 3	97

4.5 Summary photochromic properties of photoswitch 3, 4, 5 and ligand 1

Table S20. Summary of the photochromic properties of photoswitch **3**, **4**, **5** and ligand **1** in DMSO. The results were obtained using UV-Vis and ^{1}H and ^{19}F NMR spectroscopy.

**Photoswitch**	**PSS at 530 nm** ***%E:Z***	**PSS at 424 nm** ***%E:Z***	**$t_{½}$ at 25 °C**
Photoswitch **3**	22:78 [4]	81:19 [4]	11 days
Photoswitch **4**	12:88	81:19	19 days
Photoswitch **5**	7:93	79:21	209 days
Ligand **1**	8:92	79:21	97 days

5 Synthesis and characterization of homoleptic cages $[Pd_3(E\text{-}1)_6]^{6+}$ and $[Pd_4(E\text{-}1)_8]^{8+}$

5.1 Self assembly of $[Pd_3(E\text{-}1)_6](BAr_F)_6$ in MeCN-d_3

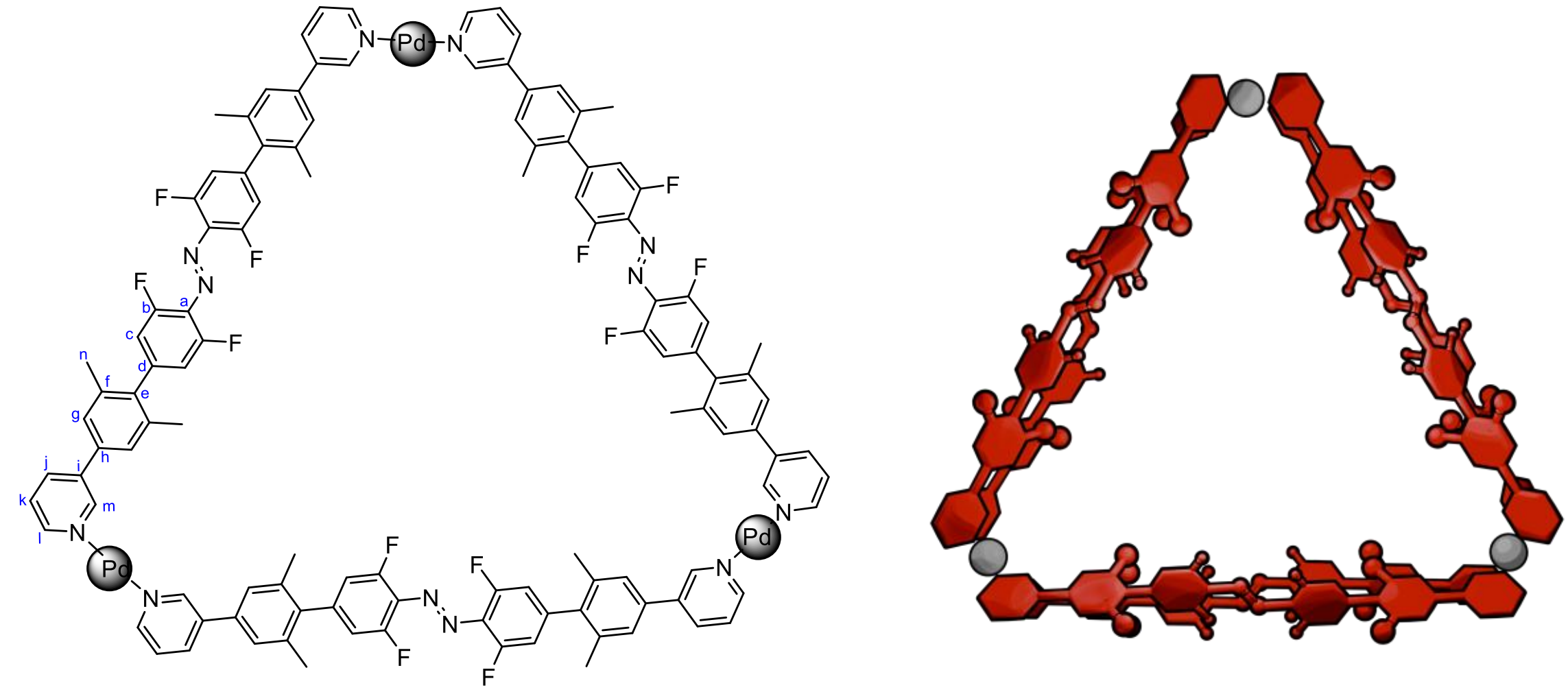

The self-assembly between $[Pd(Py^*)_4](BAr_F)_2$ and ligand **1** in MeCN-d_3 was characterized using ^1H and ^{19}F NMR spectroscopy as well as ESI-MS. To ensure only *E*-**1** was present, a solution of the photoswitchable ligand **1** (500 µL, 5.0 mM, 2.0 equiv., in MeCN-d_3) was heated in a sealed microwave tube at 120 °C for 3 hours (monitored by ^1H NMR spectroscopy). A solution of $[Pd(Py^*)_4](BAr_F)_2$ (50 µL, 29.7 mM, 1.0 equiv., in MeCN-d_3) was added to the ligand *E*-**1**, mixed well using a Vortex mixer and the mixture was left to equilibrate in a sand bath at 50 °C for 10 minutes. ^1H and ^{19}F NMR revealed the quantitative assembly of a single, highly symmetric species and free 3-chloropyridine (Py*). With the help of ESI-MS, this self-assembled species could be identified as a $[Pd_3(E\text{-}\mathbf{1})_6](BAr_F)_6$ cage. We propose that the structure of this $[Pd_3(E\text{-}\mathbf{1})_6](BAr_F)_6$ cage is a double-walled triangle, consistent with a previously reported structure formed from a similar ligand.[4]

^1H NMR (600 MHz, MeCN-d_3) δ 9.50 (d, J = 2.3 Hz, 12H, H^m), 9.11 (dd, J = 5.8, 1.3 Hz, 12H, H^l), 8.26 (dt, J = 8.0, 1.7 Hz, 12H, H^j), 7.71 (dd, J = 8.0, 5.8 Hz, $12H^k$), 7.39 (s, 24H, H^g), 7.01 (dd, J = 9.5, 1.2 Hz, 24H, H^c), 2.10 (s, 72H, H^n).

^{19}F NMR (565 MHz, MeCN-d_3) δ -122.56 (d, J = 10.7 Hz, F^b).

^{13}C NMR (151 MHz, CD_3CN) δ 156.4 (dd, J = 260.0, 4.5 Hz, C^b), 150.3 (C^h), 149.4 (C^l), 148.7 (C^m), 145.7 (t, J = 11.2 Hz, C^d), 141.1 (C^e), 139.7 (C^i), 138.2 (C^j), 131.2 (t, J = 10.1 Hz, C^a), 128.1 (C^k), 127.2 (C^f), 126.3 (C^g), 114.8 (d, J = 20.9 Hz, C^c), 20.9 (C^n).

5.1.1 1D and 2D NMR characterization of [Pd$_3$(*E*-1)$_6$](BAr$_F$)$_6$ in MeCN-*d*$_3$

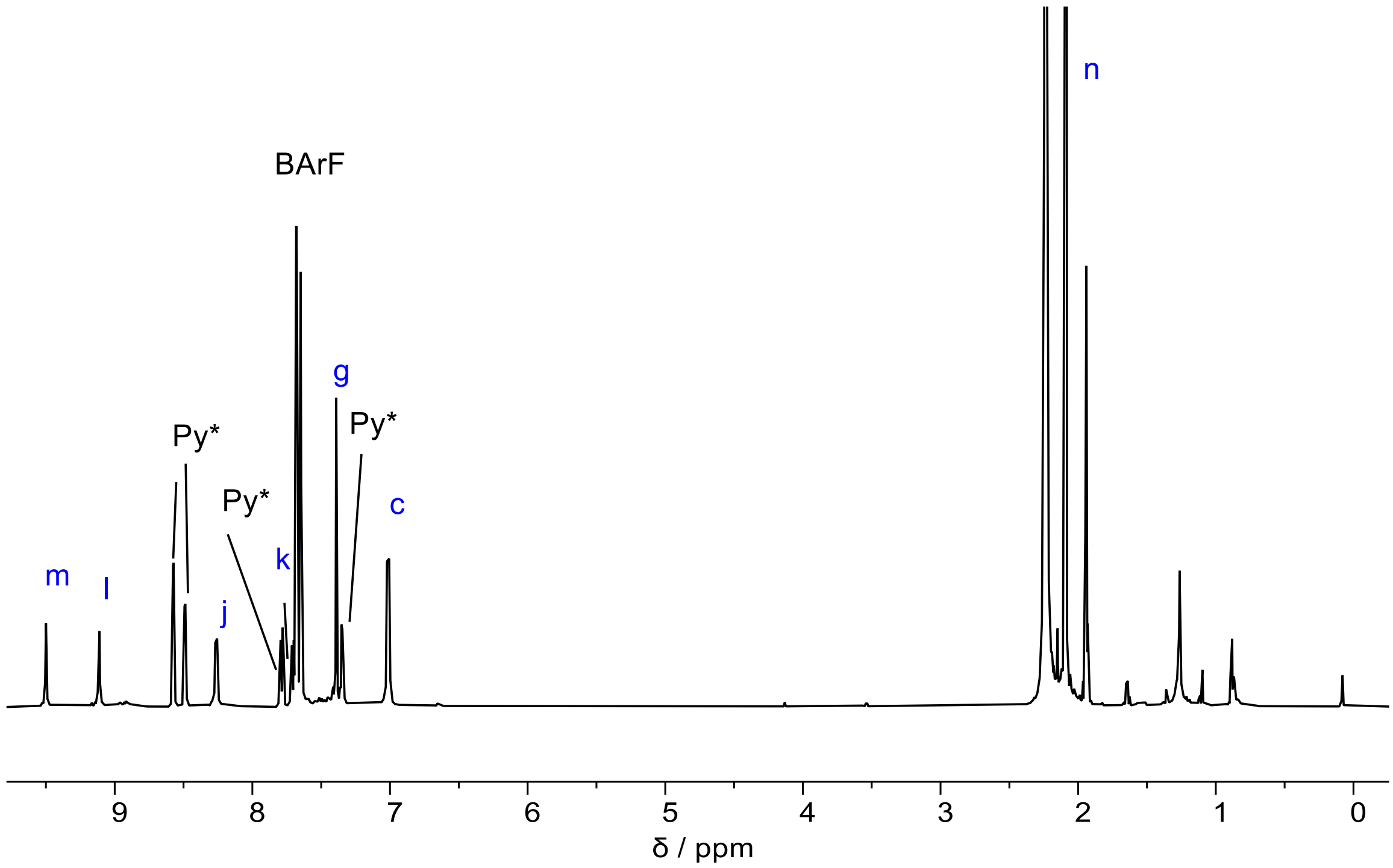

Figure S58. ^{1}H NMR (600 MHz, 298 K, MeCN-*d*$_3$) spectrum of [Pd$_3$(*E*-**1**)$_6$](BAr$_F$)$_6$ and free Py*.

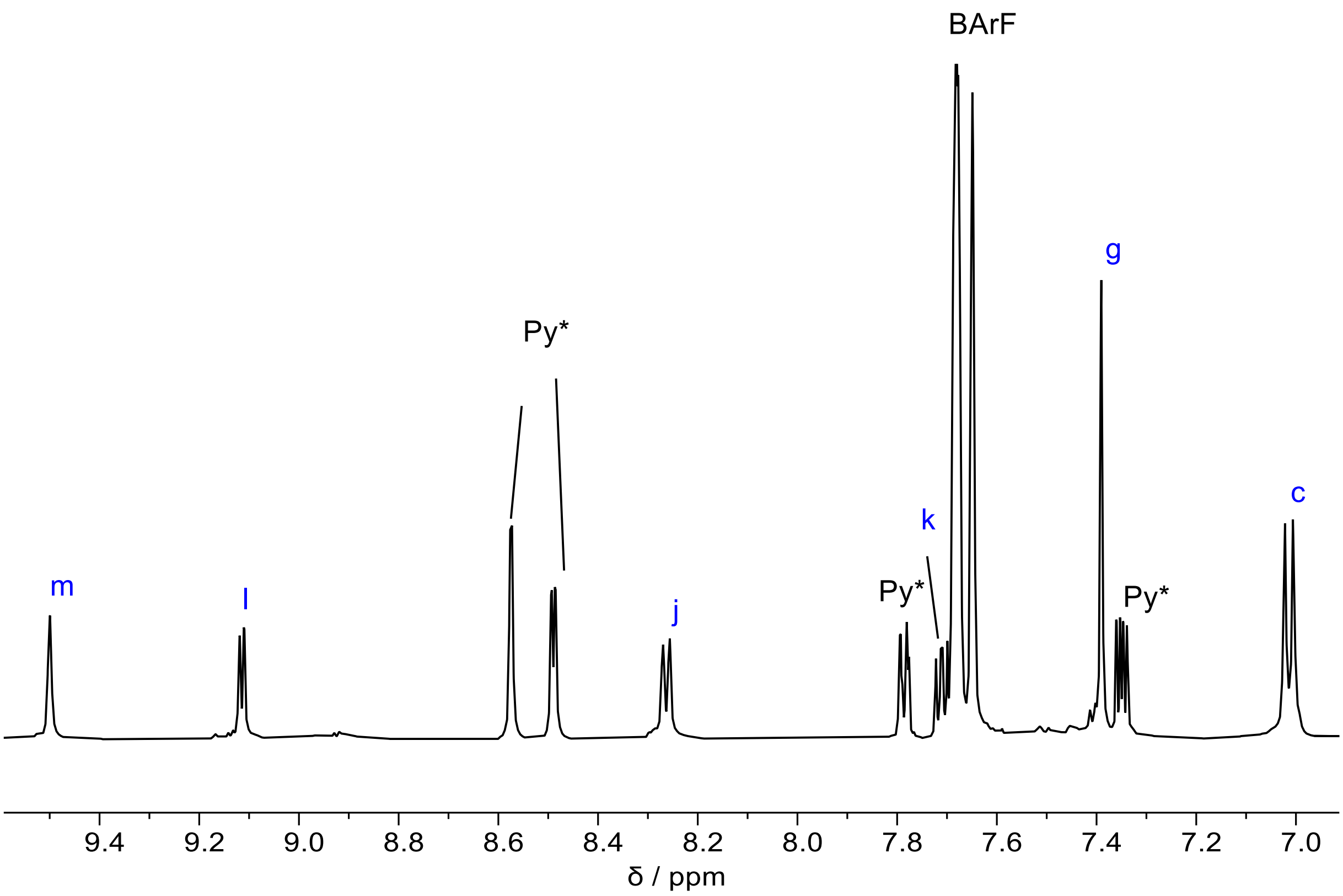

Figure S59. Partial ^{1}H NMR (600 MHz, 298 K, MeCN-*d*$_3$) spectrum of [Pd$_3$(*E*-**1**)$_6$](BAr$_F$)$_6$ and free Py*.

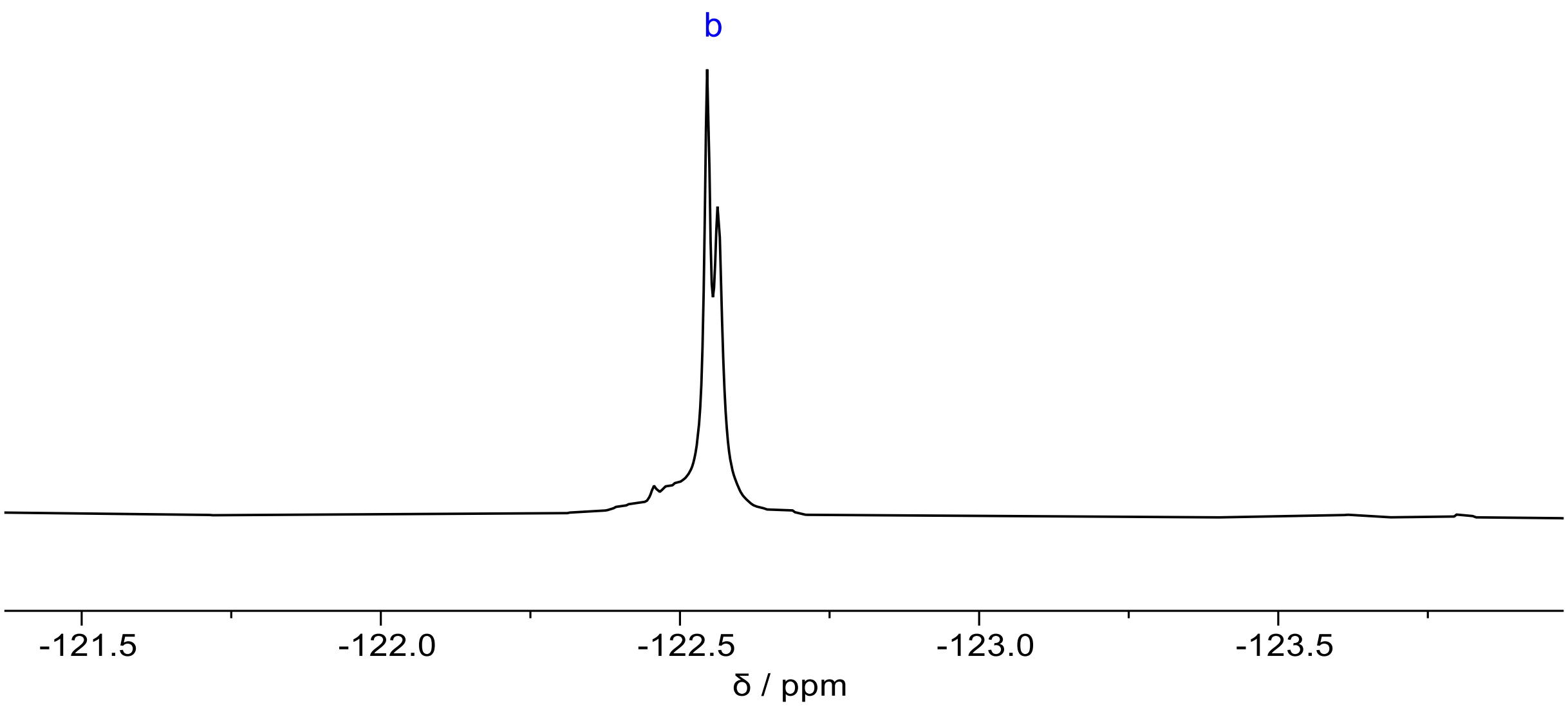

Figure S60. Partial ^{19}F NMR (565 MHz, 298 K, MeCN-d_3) spectrum of $[Pd_3(E\text{-}\mathbf{1})_6](BAr_F)_6$ and free Py*.

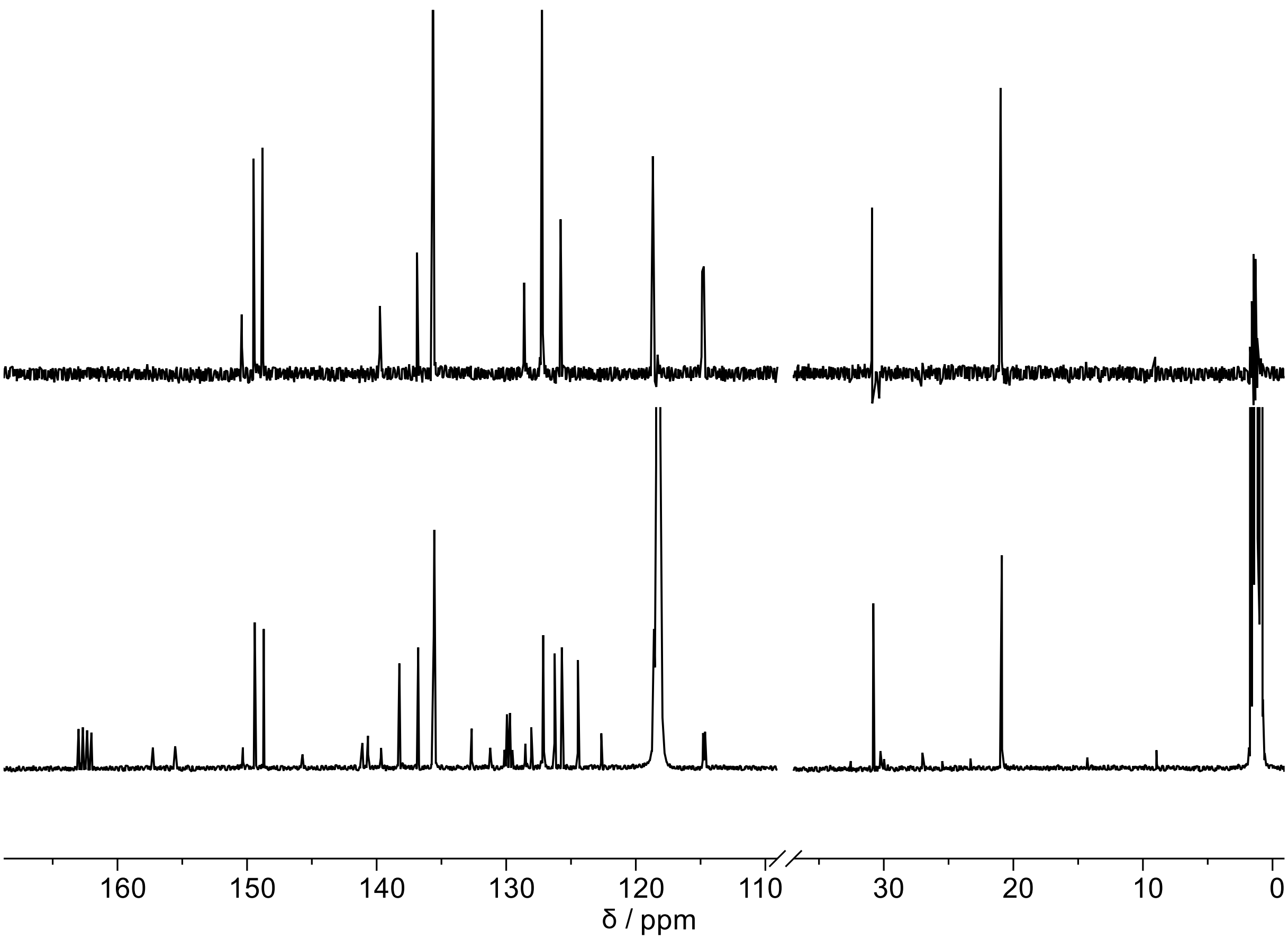

Figure S61. $^{13}C\{^1H\}$ and 135-DEPT NMR (151 MHz, 298 K, DMSO-d_6) spectrum of $[Pd_3(E\text{-}\mathbf{1})_6](BAr_F)_6$ and free Py*.

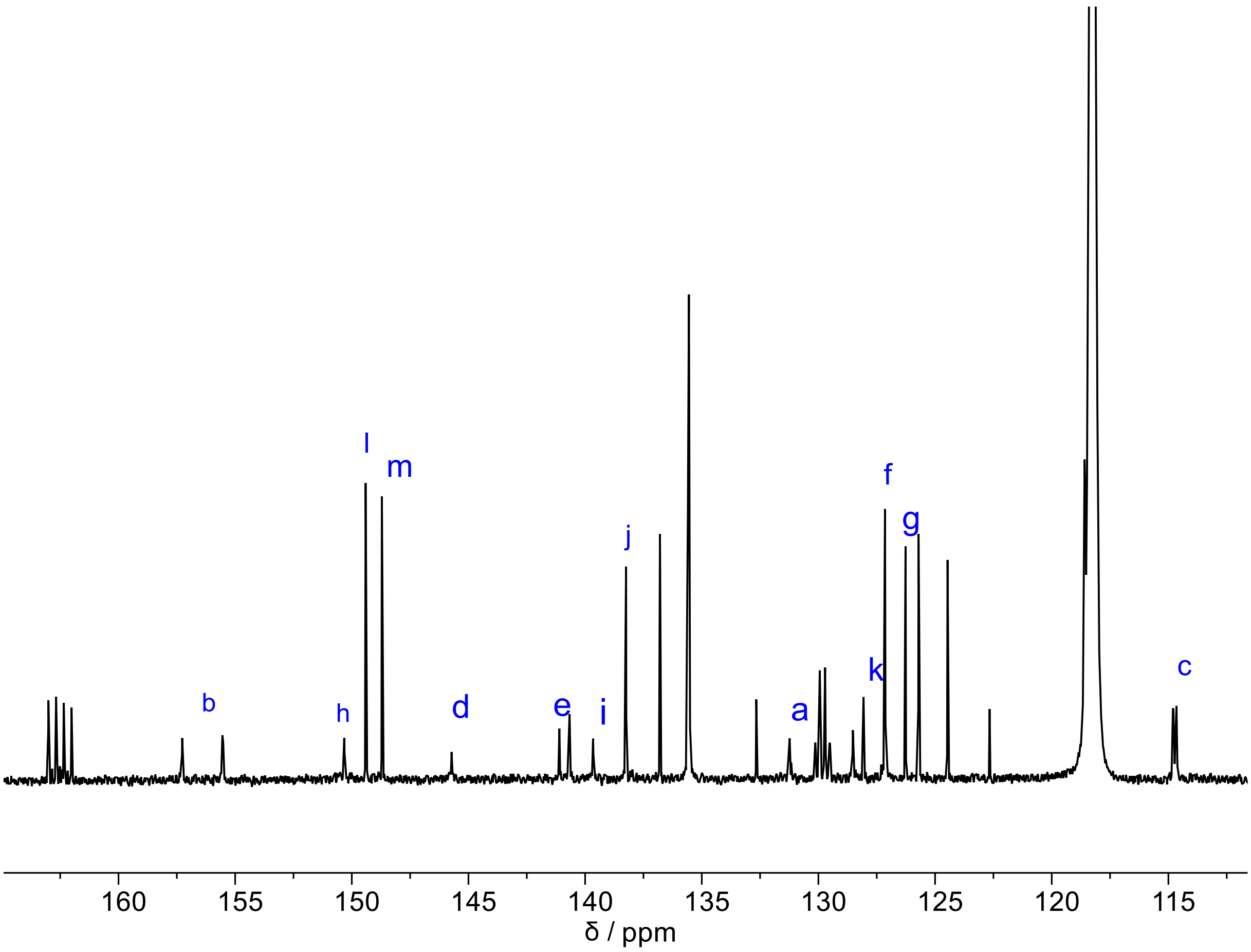

Figure S62. Partial ^{13}C{^{1}H} NMR (151 MHz, 298 K, MeCN-d_3) spectrum of $[Pd_3(E\text{-}1)_6](BAr_F)_6$ and free Py*. Unassigned peaks belong to BAr_F and free Py*.

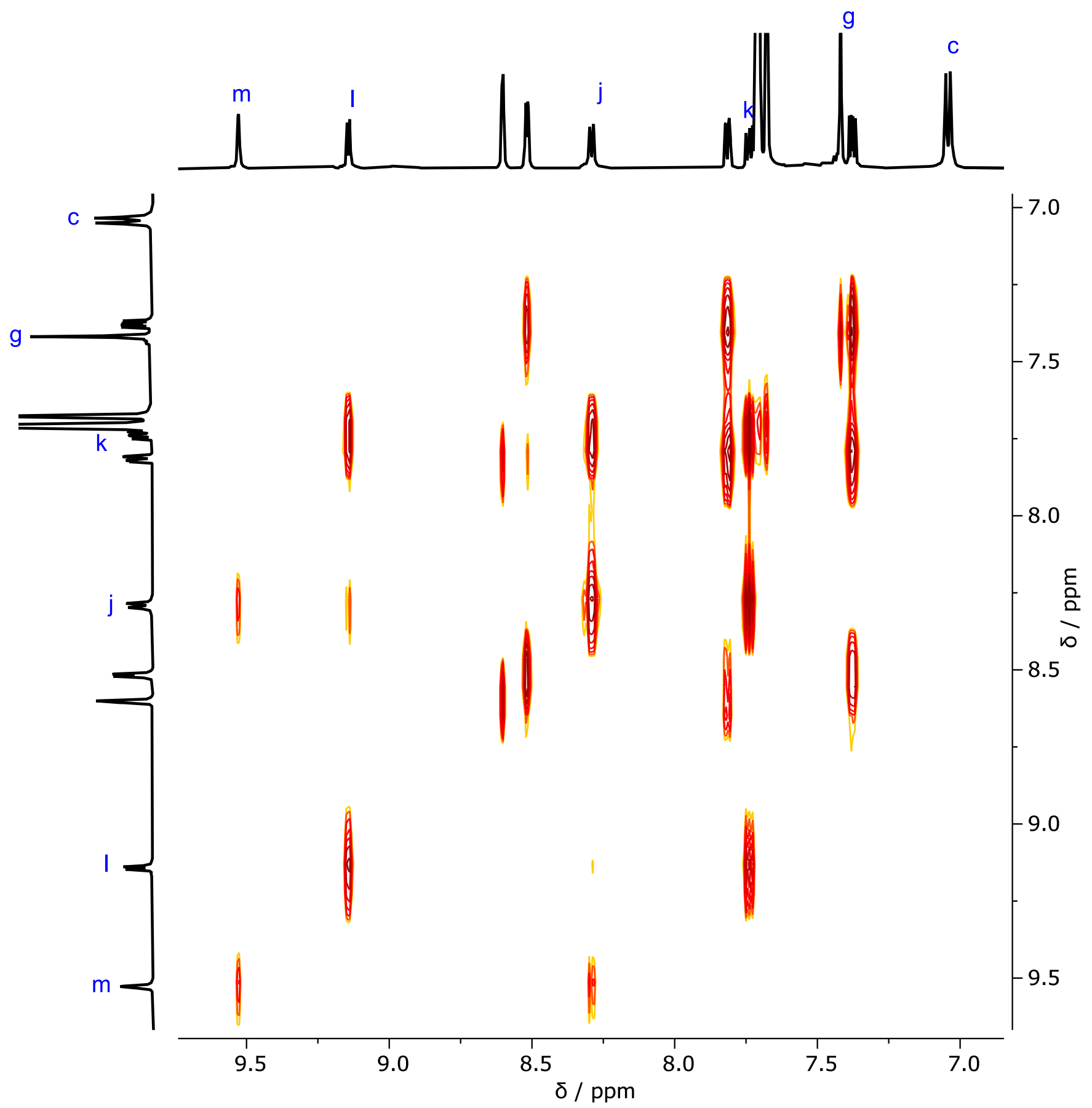

Figure S63. ^{1}H-^{1}H COSY NMR (600 MHz, 298 K, MeCN-d_3) spectrum of $[Pd_3(E\text{-}\mathbf{1})_6](BAr_F)_6$ and free Py*.

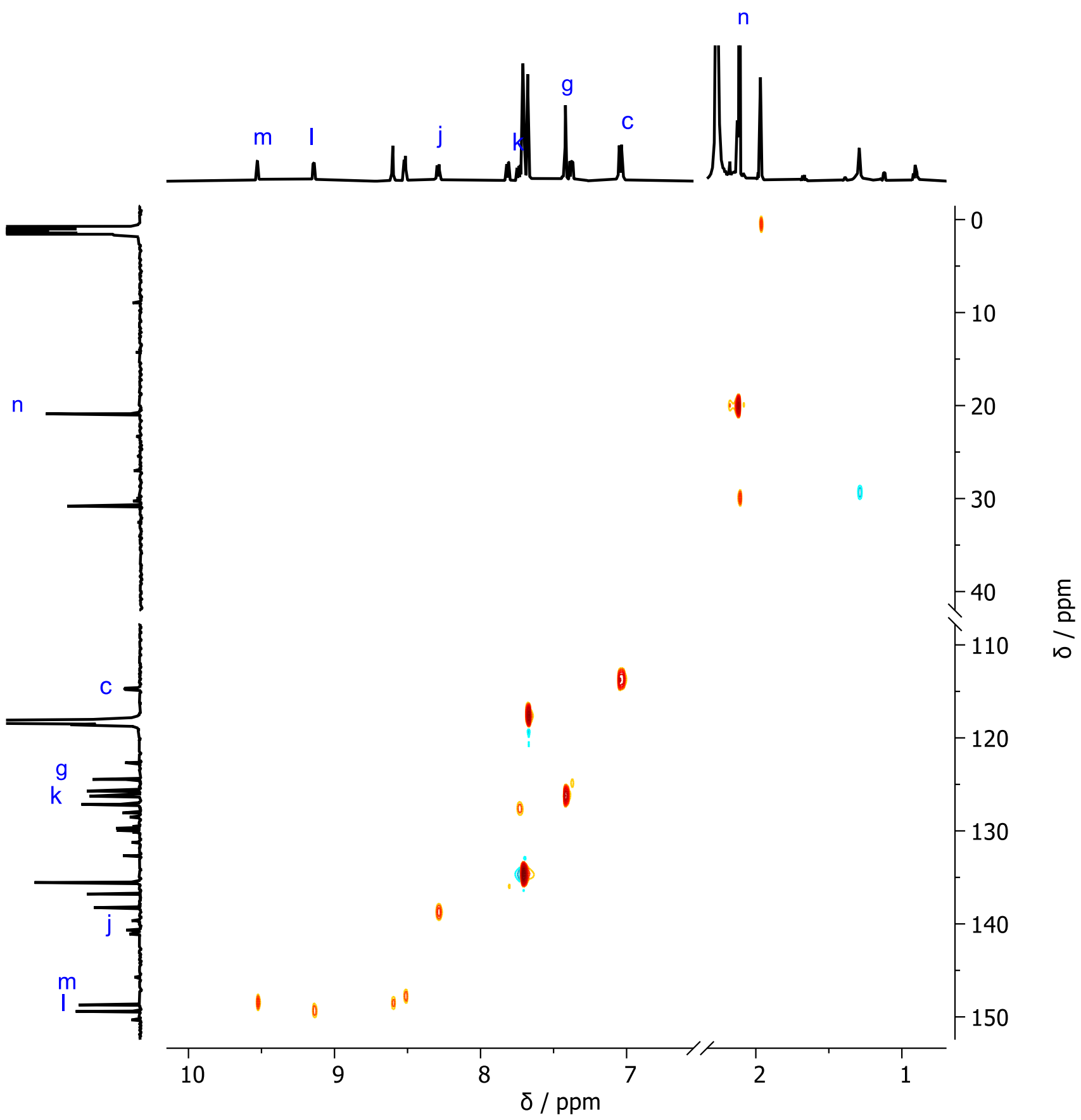

Figure S64. ^{1}H-^{13}C HSQC NMR (600 MHz, 151 MHz, 298 K, MeCN-d_3) spectrum of $[Pd_3(E\text{-}\mathbf{1})_6](BAr_F)_6$ and free Py*.

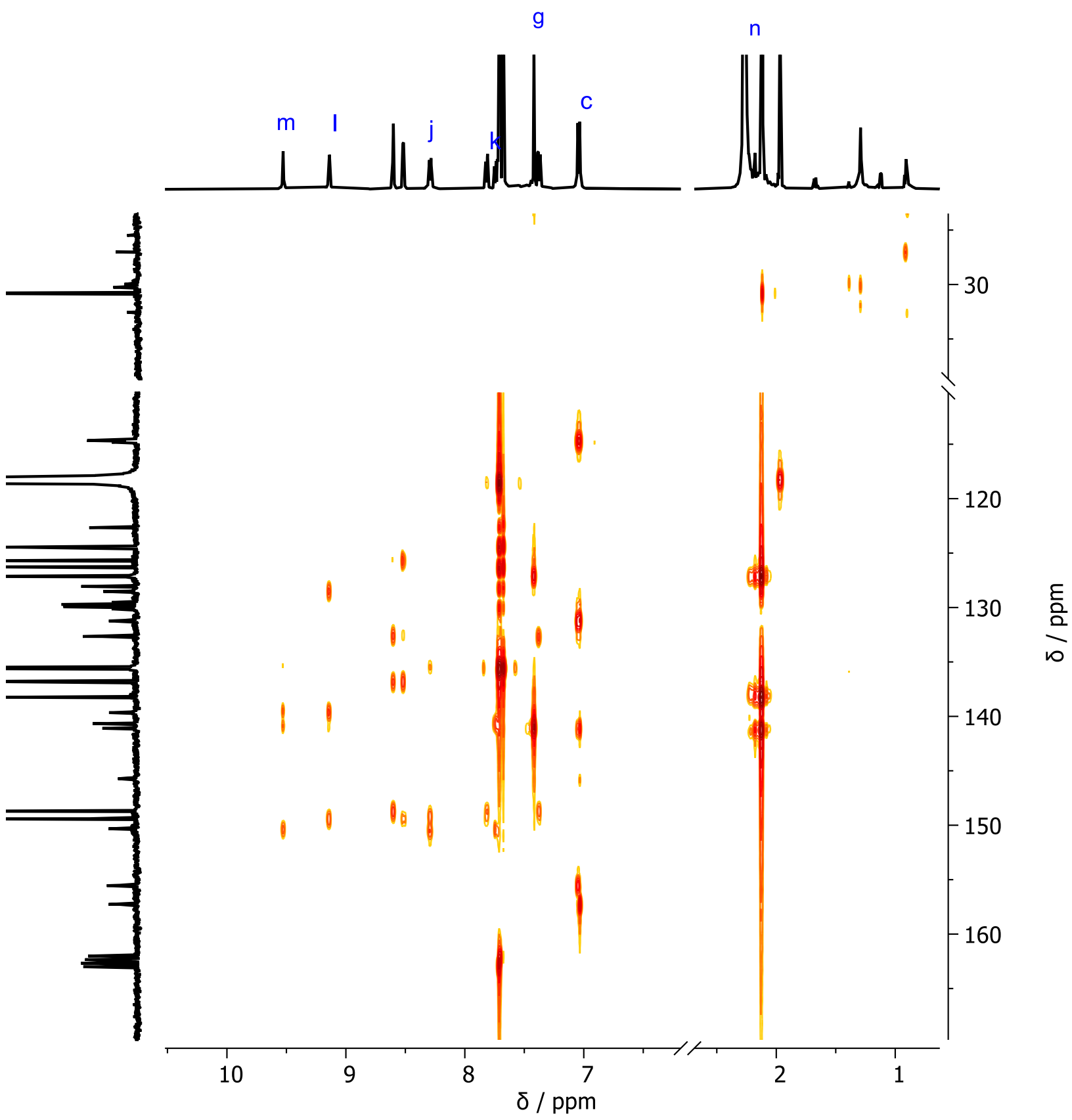

Figure S65. ^{1}H-^{13}C HMBC NMR (600 MHz, 151 MHz, 298 K, MeCN-d_3) spectrum of $[Pd_3(E\text{-}\mathbf{1})_6](BAr_F)_6$ and free Py*.

5.1.2 ESI-MS of $[Pd_3(E\text{-}\mathbf{1})_6](BAr_F)_6$ in MeCN-d_3

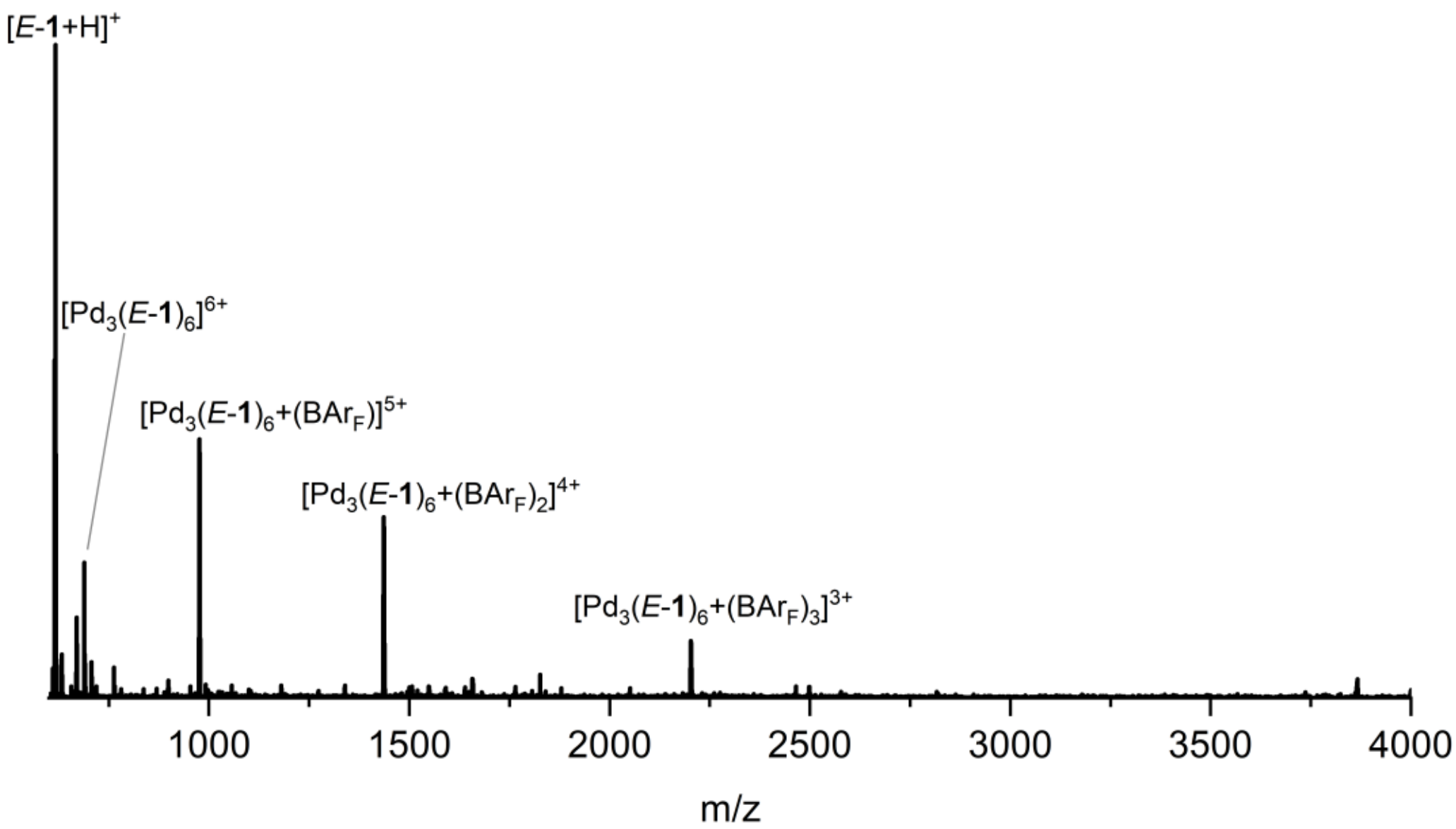

Figure S66. ESI-MS spectrum of $[Pd_3(E\text{-}\mathbf{1})_6](BAr_F)_6$ ([Pd] = 2.5 mM) in MeCN-d_3.

Table S 21. Zoom scans of select ESI-MS peaks, with simulated isotope patterns of $[Pd_3(E\text{-}\mathbf{1})_6](BAr_F)_6$ ([Pd] = 2.5 mM) in MeCN-d_3.

$[Pd_3(E\text{-}\mathbf{1})_6 + (BAr_F)_3]^{3+}$	$[Pd_3(E\text{-}\mathbf{1})_6 + (BAr_F)_2]^{4+}$
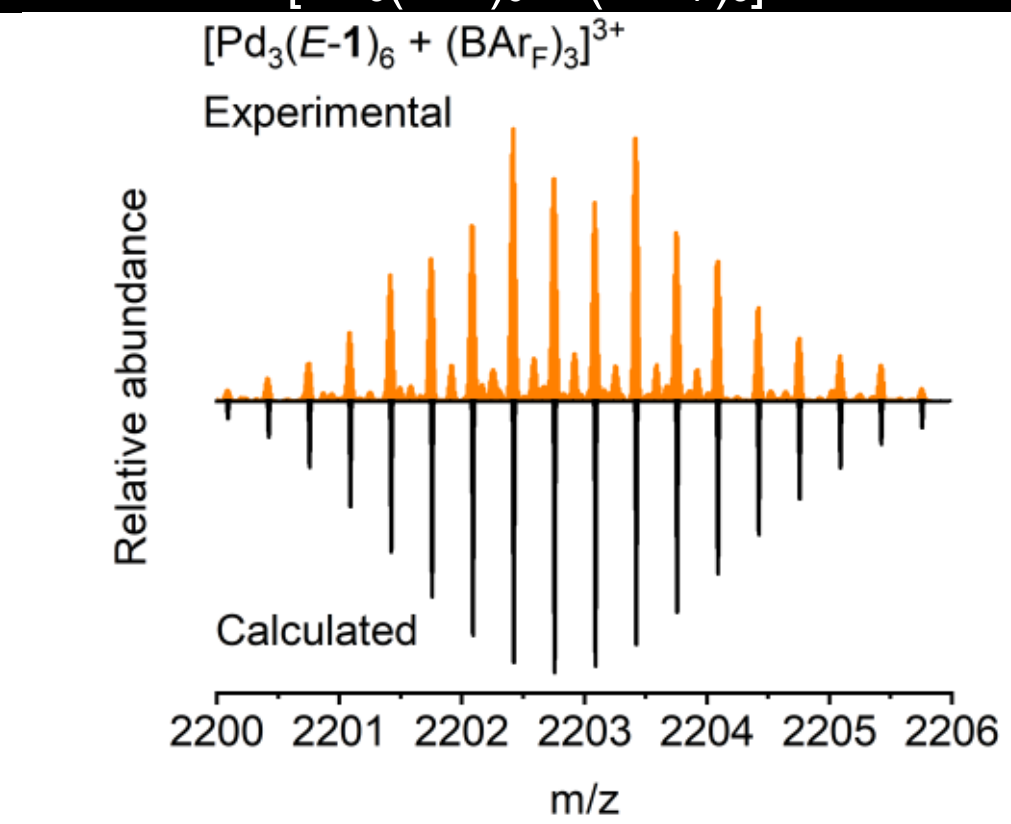	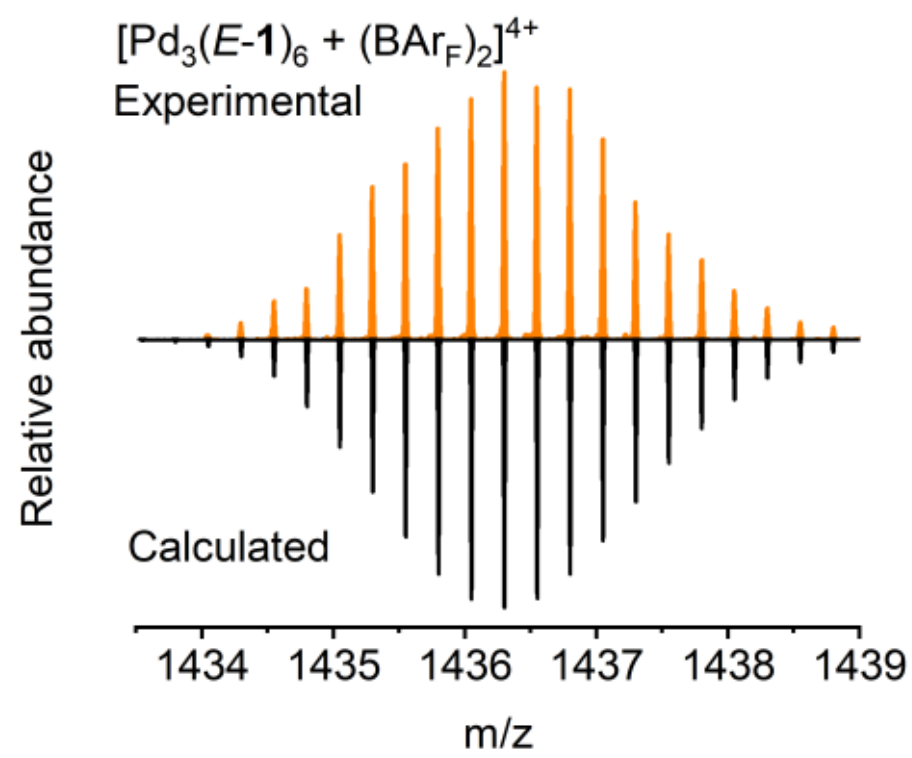
$[Pd_3(E\text{-}\mathbf{1})_6 + (BAr_F)]^{5+}$	$[Pd_3(E\text{-}\mathbf{1})_6]^{6+}$
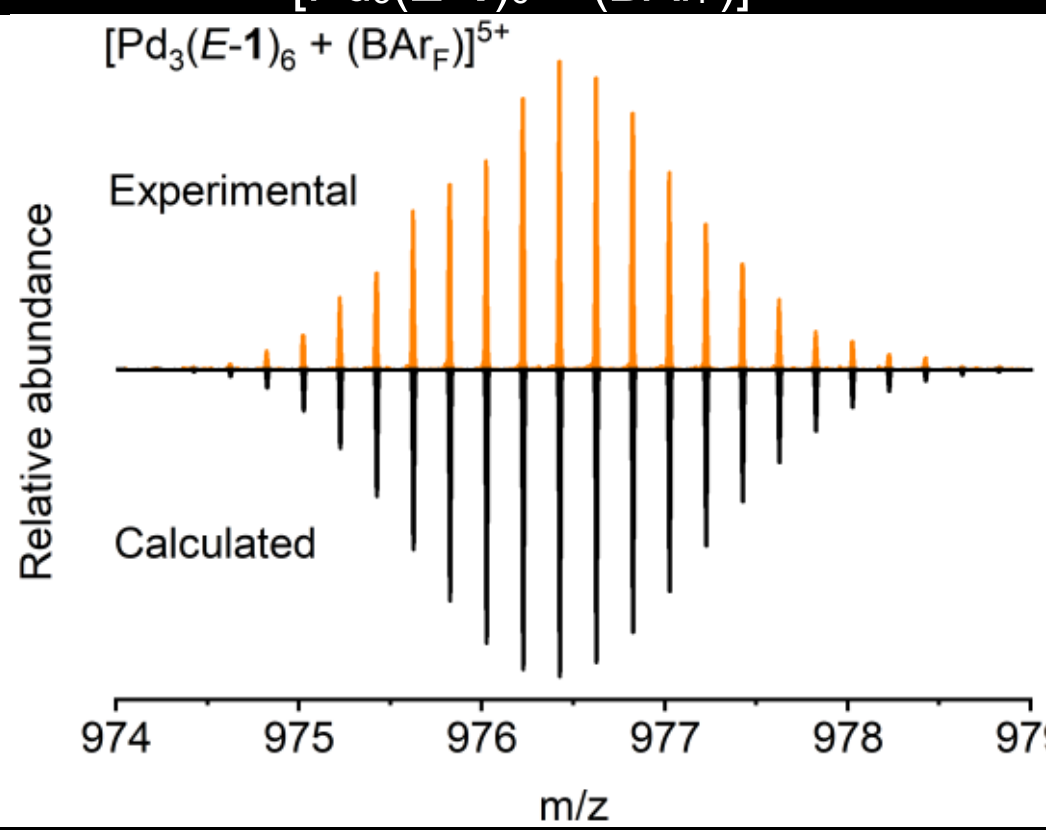	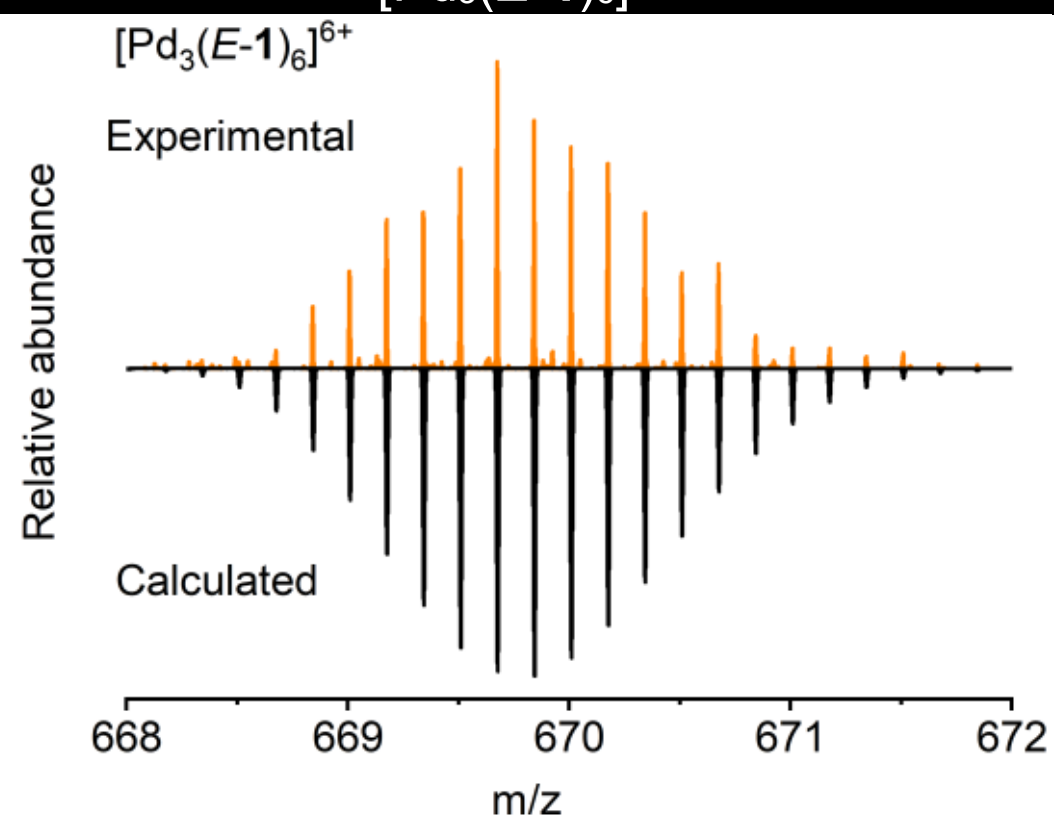

5.1.3 Self-assembly of $[Pd_3(E\text{-}1)_6](BF_4)_6$ in DMSO-d_6

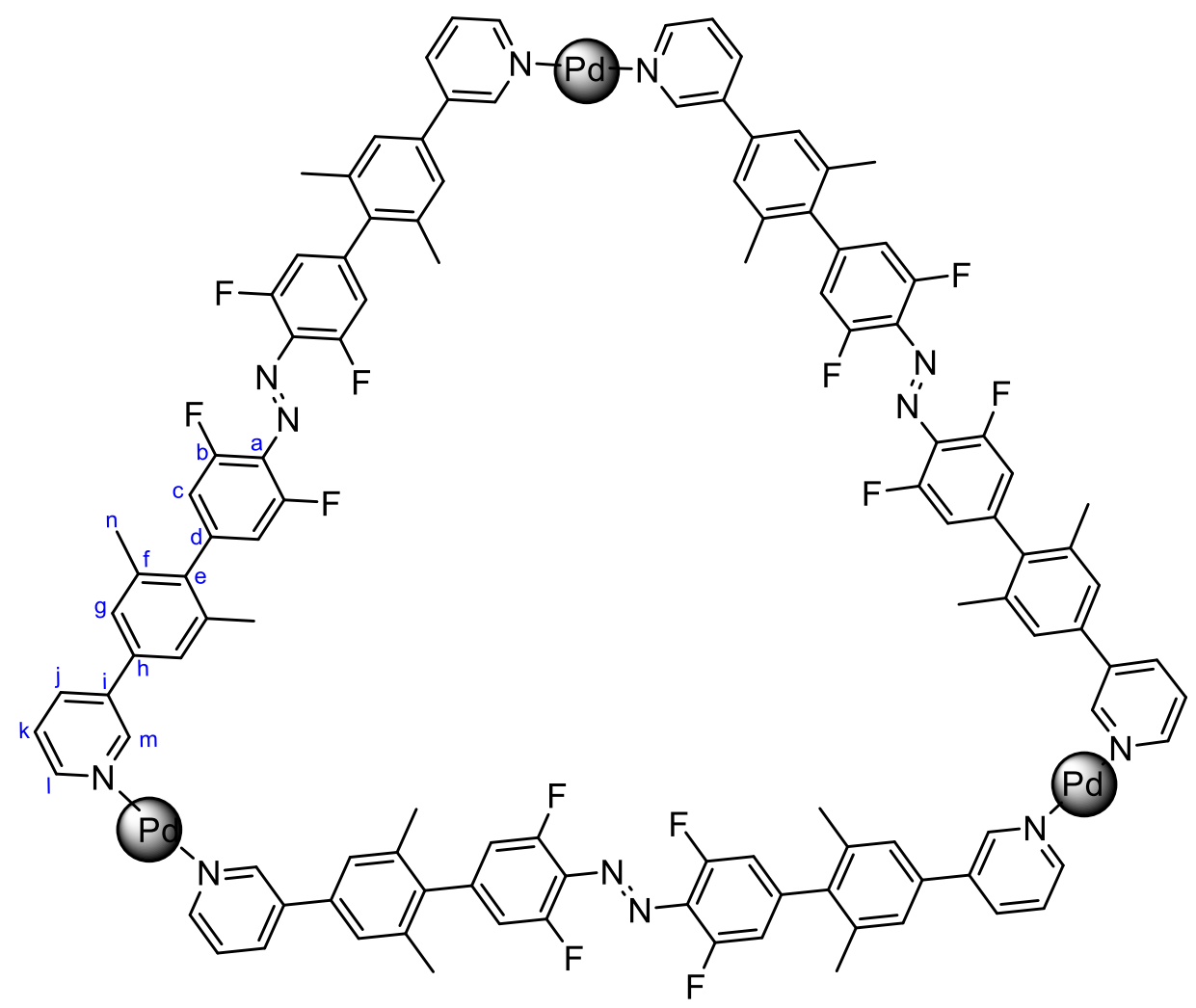

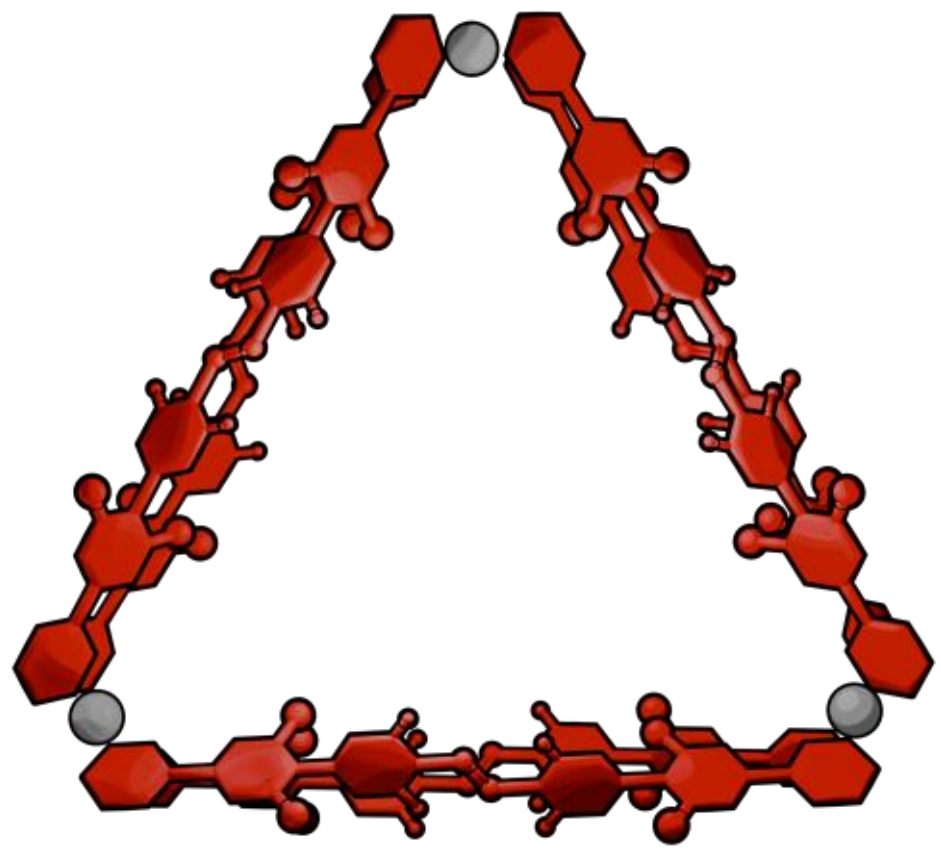

Self-assembly between $[Pd(MeCN)_4](BF_4)_2$ and ligand **1** in DMSO-d_6 led to the formation of the double-walled triangle $[Pd_3(E\text{-}\mathbf{1})_6](BF_4)_6$ cage, similar to the one reported in Section 5.1.

To ensure only *E*-**1** was present, a solution of the photoswitchable ligand **1** (500 µL, 4.3 mM, 2.0 equiv., in DMSO-d_6) was heated at 100 °C overnight (monitored by ^{1}H NMR spectroscopy). A solution of $[Pd(MeCN)_4](BF_4)_2$ (25 µL, 48.8 mM, 1.0 equiv., in DMSO-d_6) was added to the ligand *E*-**1**, mixed well using a Vortex mixer and the mixture was left to equilibrate in a sand bath at 50 °C for 10 minutes. ^{1}H and ^{19}F NMR revealed the quantitative assembly of a single, highly symmetric species. With the help of ESI-MS, this self-assembled species could be identified as a $[Pd_3(E\text{-}\mathbf{1})_6](BF_4)_6$ cage. We propose that the structure of this $[Pd_3(E\text{-}\mathbf{1})_6](BF_4)_6$ cage is a double-walled triangle, same as when ligand *E*-**1** is reacted with $[Pd(Py^*)_4](BAr_F)_2$ in MeCN-d_3

^{1}H NMR (600 MHz, DMSO-d_6) δ 9.82 (s, 12H, H^m), 9.43 (d, J = 5.9 Hz, 12H, H^l), 8.43 (d, J = 8.0 Hz, 23H, H^j), 7.90 (t, J = 6.9 Hz, 12H, H^k), 7.48 (s, 24H, H^g), 7.24 (d, J = 10.3 Hz, 24H, H^c), 2.09 (s, 72H, H^n).

^{19}F NMR (565 MHz, DMSO-d_6) δ -121.63 (F^b).

5.1.4 1D NMR characterization of $[Pd_3(E\text{-}1)_6](BF_4)_6$ in DMSO-d_6

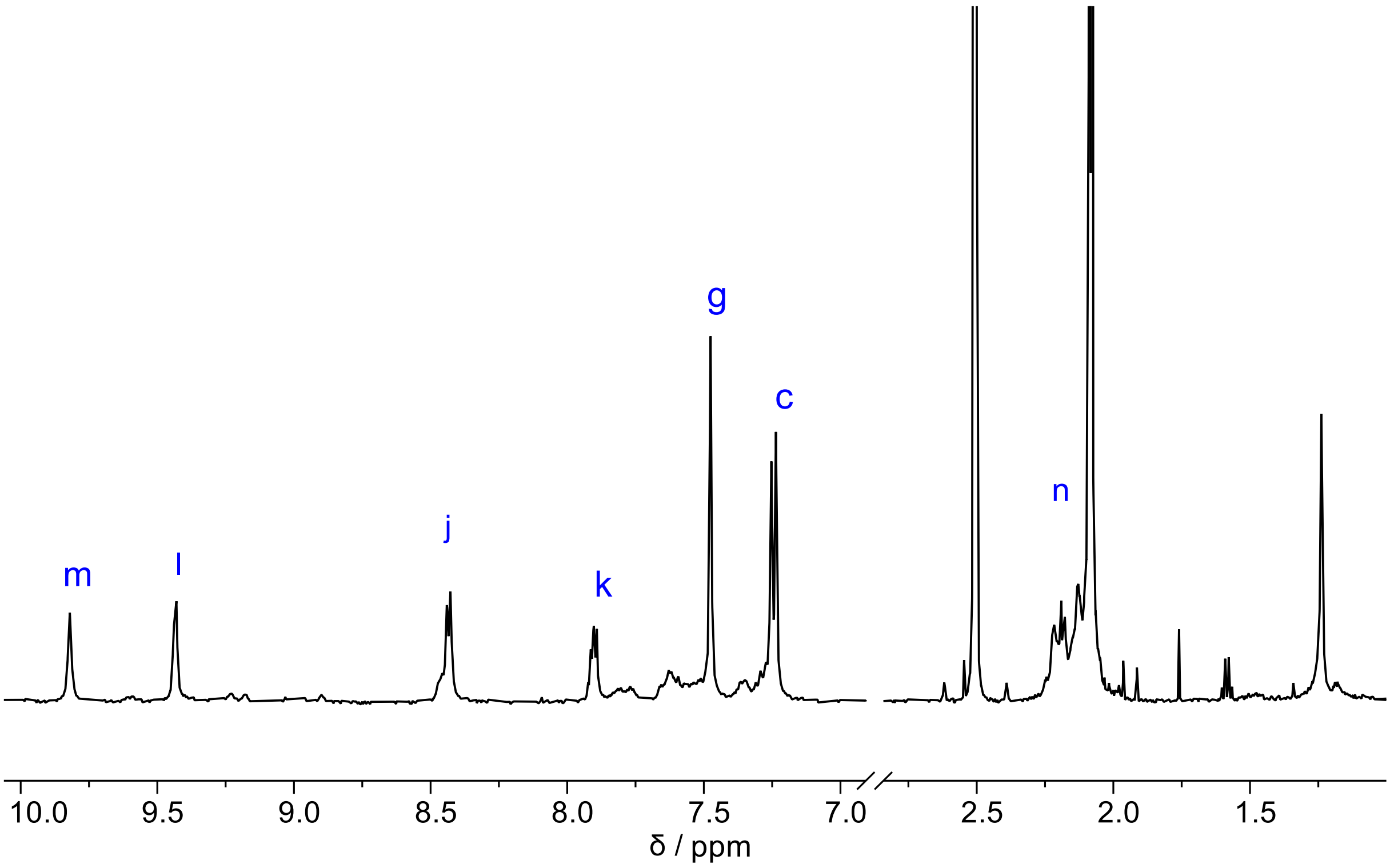

Figure S67. ^{1}H NMR (600 MHz, 298 K, DMSO-d_6) spectrum of $[Pd_3(E\text{-}\mathbf{1})_6](BF_4)_6$.

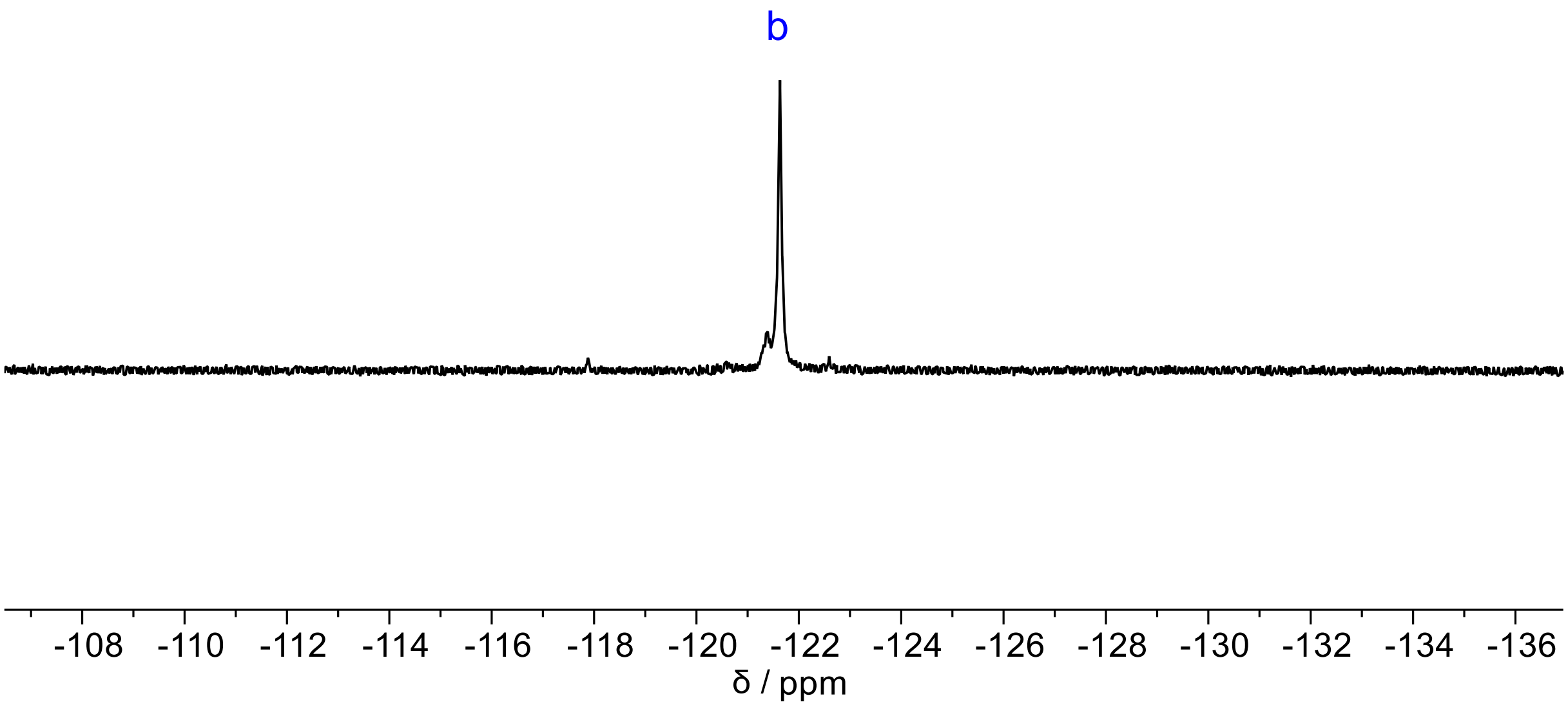

Figure S68. Partial ^{19}F NMR (565 MHz, 298 K, DMSO-d_6) spectrum of $[Pd_3(E\text{-}\mathbf{1})_6](BF_4)_6$.

5.1.5 ESI-MS of $[Pd_3(E\text{-}\mathbf{1})_6](BF_4)_6$ in DMSO

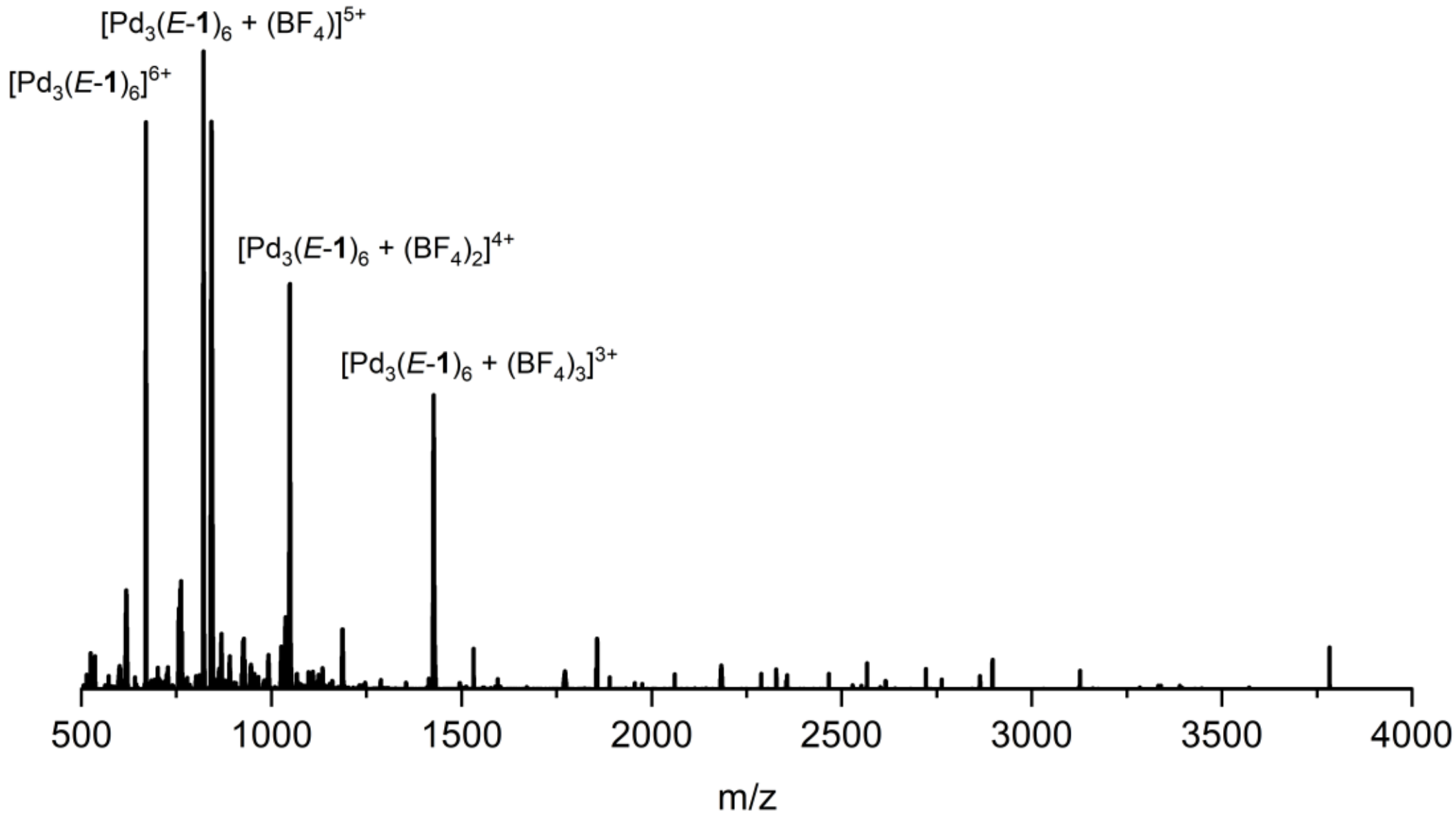

Figure S69. ESI-MS spectrum of $[Pd_3(E\text{-}\mathbf{1})_6](BF_4)_6$ ([Pd] = 2.2 mM) in DMSO-d_6.

Table S 22. Zoom scans of select ESI-MS peaks, with simulated isotope patterns of $[Pd_3(E\text{-}\mathbf{1})_6](BF_4)_6$ ([Pd] = 2.2 mM) in DMSO-d_6.

$[Pd_3(E\text{-}\mathbf{1})_6 + (BF_4)_3]^{3+}$	$[Pd_3(E\text{-}\mathbf{1})_6 + (BF_4)_2]^{4+}$
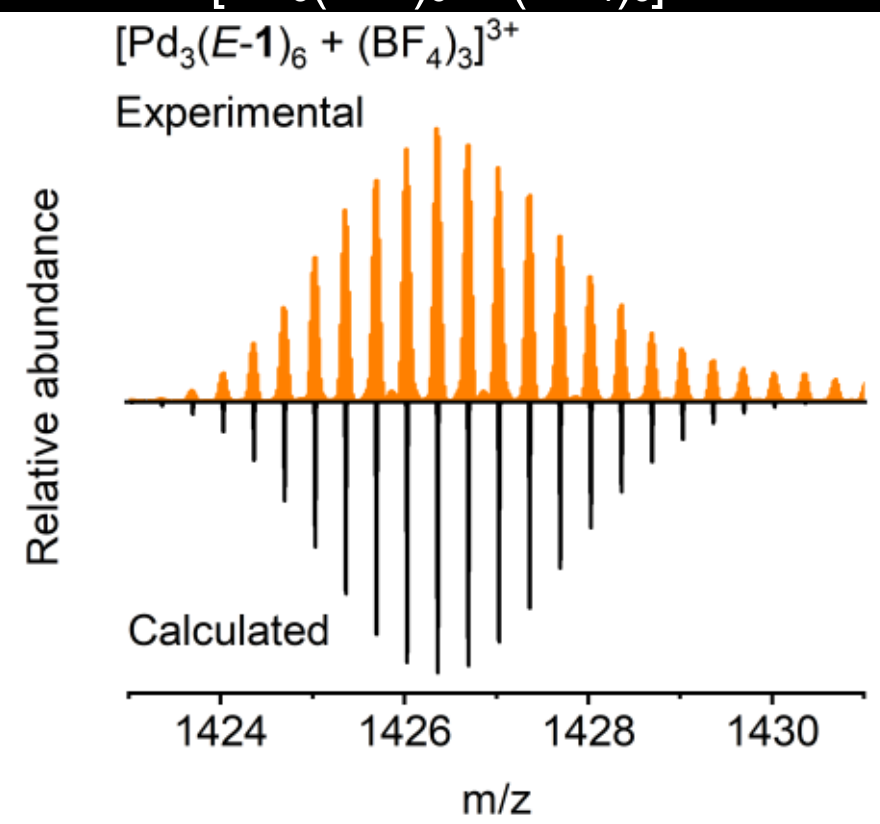	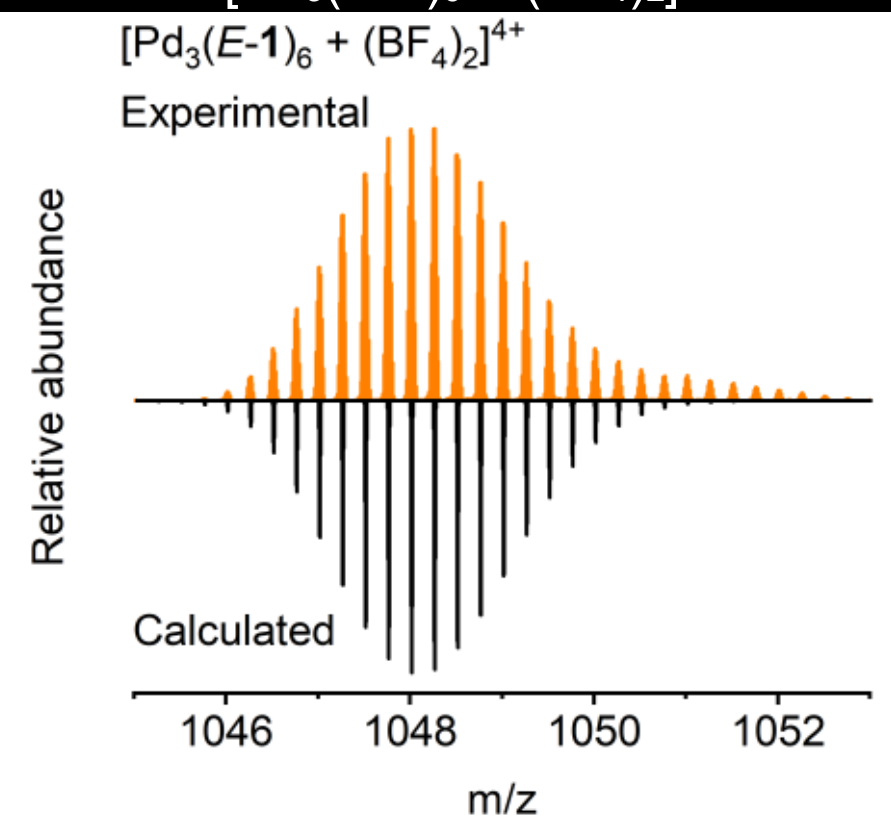
$[Pd_3(E\text{-}\mathbf{1})_6 + (BF_4)]^{5+}$	$[Pd_3(E\text{-}\mathbf{1})_6]^{6+}$
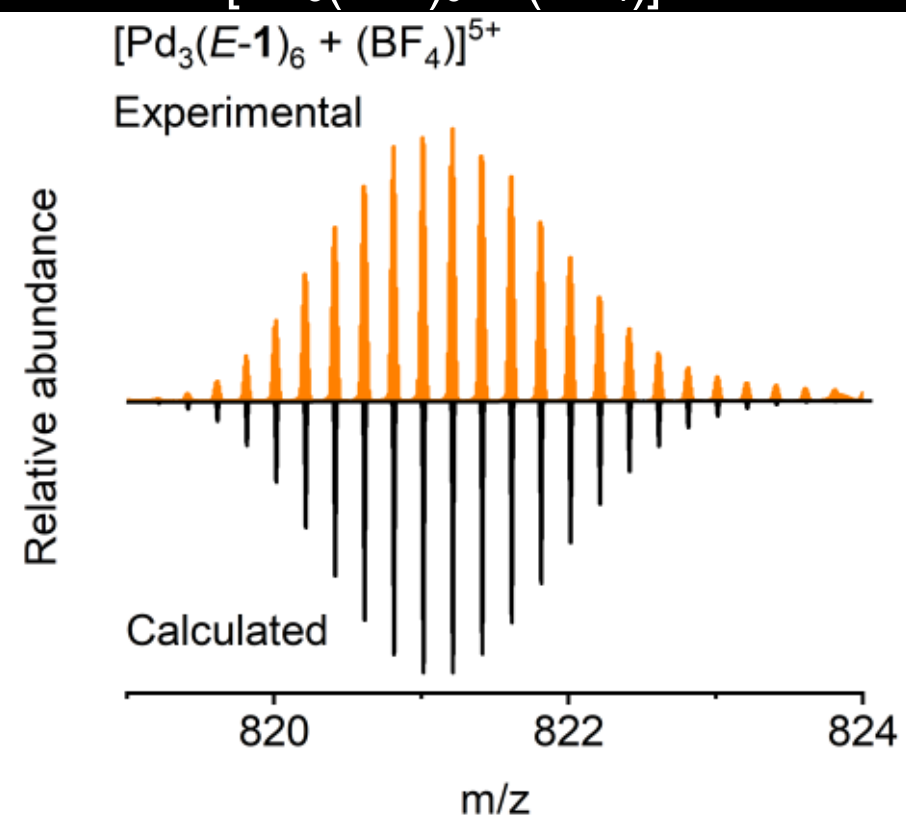	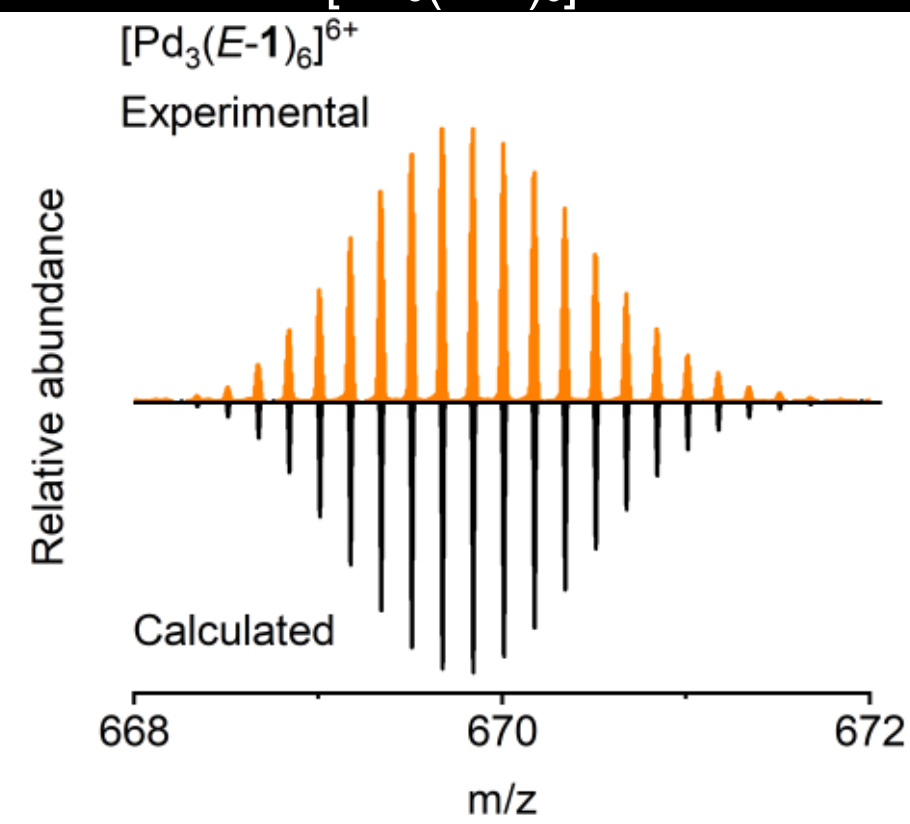

5.2 Self-assembly of $[Pd_3(E\text{-}1)_6](BF_4)_6$ and $[Pd_4(E\text{-}1)_8](BF_4)_8$ in MeCN-d_3

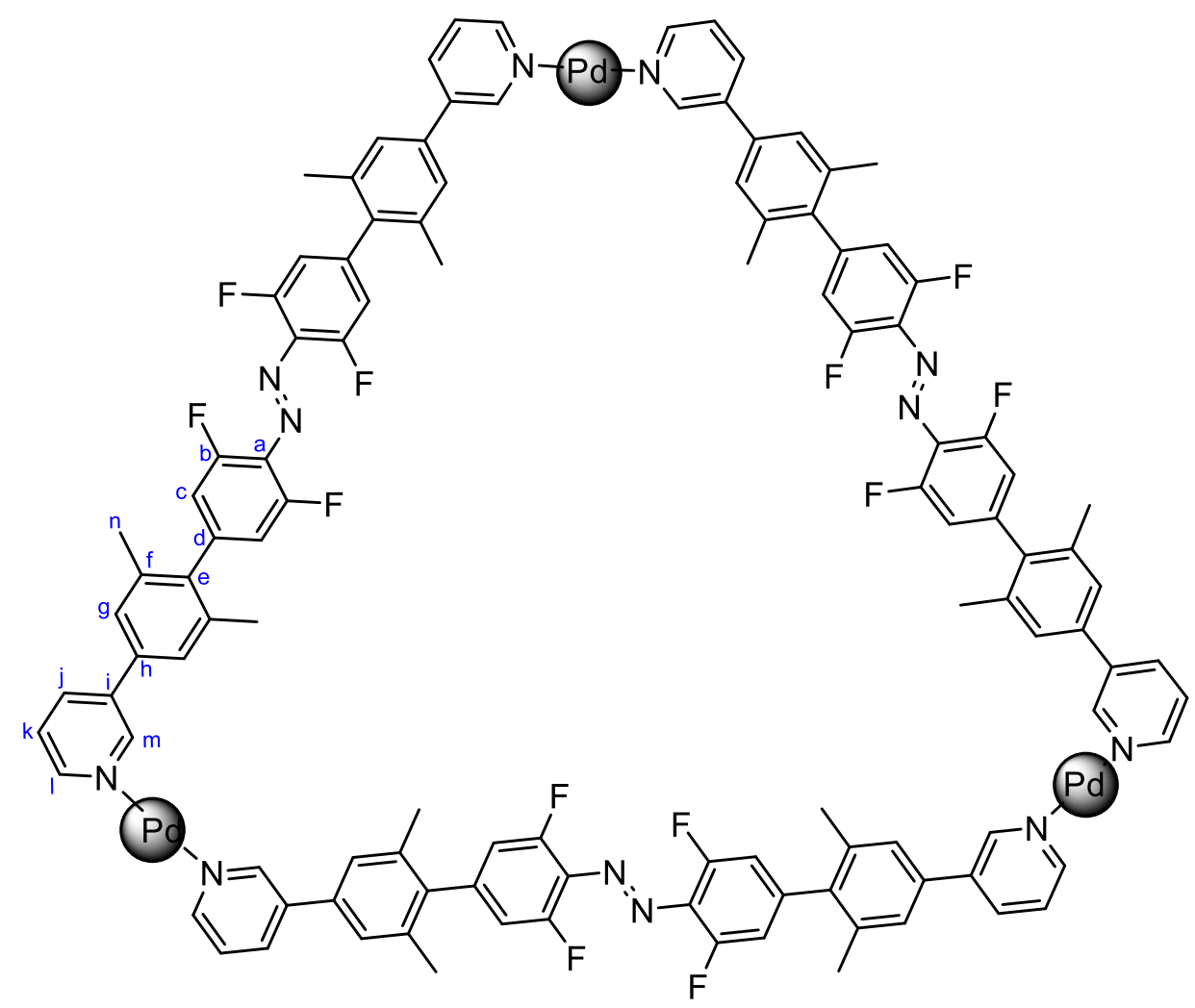
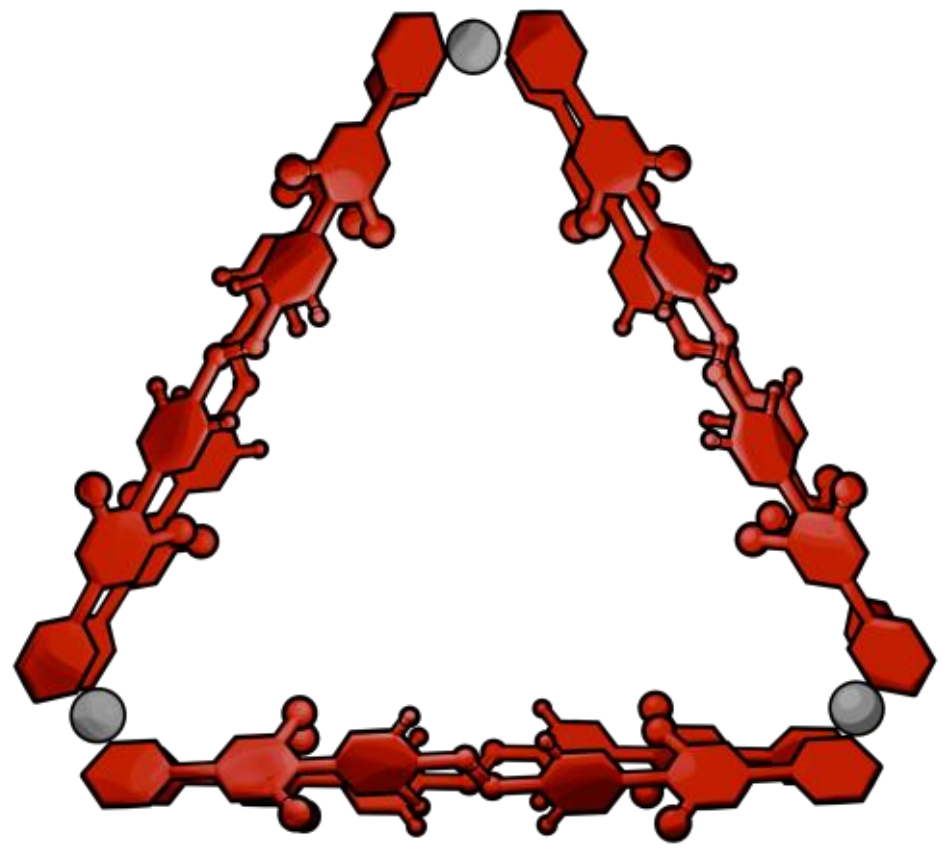

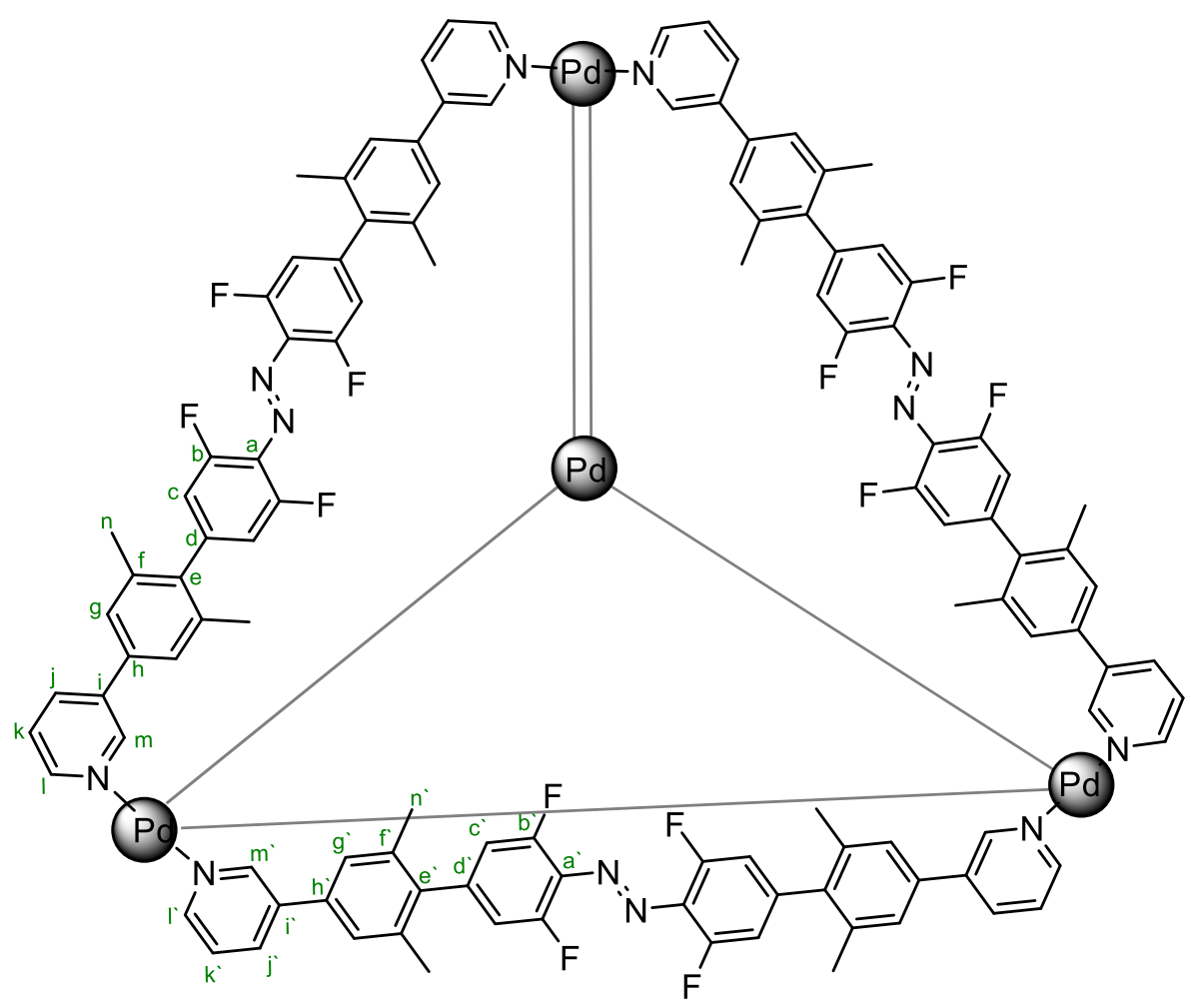
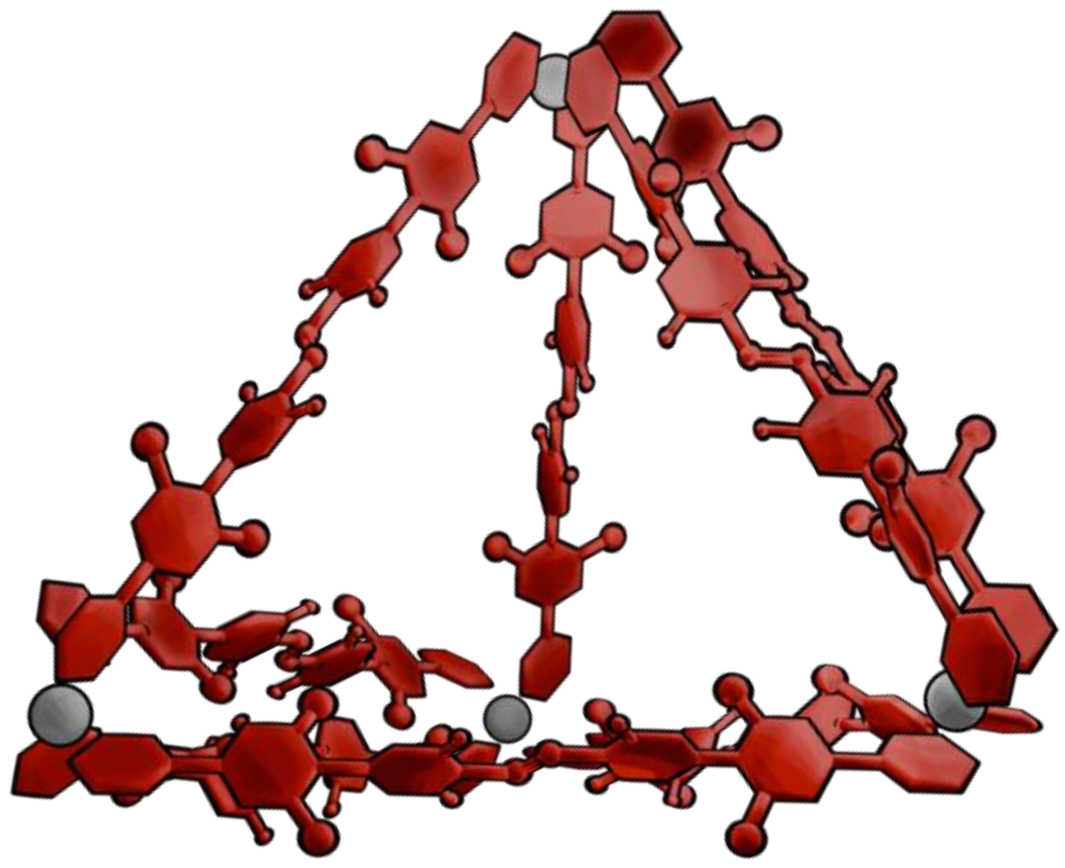

Self-assembly between $[Pd(MeCN)_4](BF_4)_2$ and ligand **1** in MeCN-d_3 led to the formation of a mixture of a double-walled triangle $[Pd_3(E\text{-}\mathbf{1})_6](BF_4)_6$ and a distorted tetrahedron $[Pd_4(E\text{-}\mathbf{1})_8](BF_4)_8$, similar to the one previously reported in our group.[4]

To ensure only *E*-**1** was present, a solution of the photoswitchable ligand **1** (500 µL, 4.7 mM, 2.0 equiv., in MeCN-d_3) was heated in a sealed microwave tube at 120 °C for 3 hours (monitored by ^{1}H NMR spectroscopy). A solution of $[Pd(MeCN)_4](BF_4)_2$ (25 µL, 50.3 mM, 1.0 equiv., in MeCN-d_3) was added to the ligand *E*-**1**, mixed well using a Vortex mixer and the mixture was left to equilibrate in a sand bath at 50 °C for 10 minutes. ^{1}H and ^{19}F NMR revealed the quantitative assembly of two sets of peaks, which we assign to i) a single, highly symmetric species and ii) a species with two different ligand environments. With the help of ESI-MS, this self-assembled species could be identified as a $[Pd_3(E\text{-}\mathbf{1})_6](BF_4)_6$ and a $[Pd_4(E\text{-}\mathbf{1})_8](BF_4)_8$ cage. We propose that the structure of this $[Pd_3(E\text{-}\mathbf{1})_6](BF_4)_6$ cage is again a double-walled triangle, same as when ligand *E*-**1** is reacted with $[Pd(Py^*)_4](BAr_F)_2$ in MeCN-d_3 or with $[Pd(MeCN)_4](BF_4)_2$ in DMSO-d_6. For the structure of the $[Pd_4(E\text{-}\mathbf{1})_8](BF_4)_8$ cage we propose a distorted tetrahedron, consistent with a previously reported structure formed from a similar ligand.[4] Selective 1D ROESY NMR of the mixture of $[Pd_3(E\text{-}\mathbf{1})_6](BF_4)_6$ and $[Pd_4(E\text{-}\mathbf{1})_8](BF_4)_8$ confirmed through-space interactions of m and m\` and l and l\` in $[Pd_4(E\text{-}\mathbf{1})_8](BF_4)_8$, supporting the presence of two different ligand environments in the distorted tetrahedron (Figure S75).

[Pd$_4$(*E*-1)$_8$](BF$_4$)$_8$:

^{1}H NMR (600 MHz, MeCN-d_3) δ 9.83 (d, J = 2.2 Hz, 8H, H^{m}), 9.58 (d, J = 2.1 Hz, 8H, $H^{m`}$), 9.20 (dd, $J$ = 5.8, 1.4 Hz, 8H, $H^{l}$), 8.98 (dd, $J$ = 5.8, 1.3 Hz, 8H, $H^{l`}$), 8.32 (dt, J = 8.4, 1.6 Hz, 8H, H^{j}), 8.29 – 8.27 (m, 8H, $H^{j`}$), 7.78 (dd, $J$ = 8.2, 5.7 Hz, 8H, $H^{k}$), 7.63 (d, $J$ = 5.6 Hz, 8H, $H^{k`}$), 7.62 (s, 16H, H^{g}), 7.59 (s, 16H $H^{g`}$), 7.10 – 7.07 (m, 32H, $H^{c+c`}$), 2.19 (br. s, 48H, $H^{n+n`}$).

^{19}F and ^{13}C NMR: Due to overlapping peaks and low concentration of the cages the individual peaks cannot unambiguously be assigned to either [Pd$_3$(*E*-**1**)$_6$](BF$_4$)$_6$ or [Pd$_4$(*E*-**1**)$_8$](BF$_4$)$_8$.

[Pd$_3$(*E*-1)$_6$](BF$_4$)$_6$:

^{1}H NMR (600 MHz, MeCN-d_3) δ 9.77 (d, J = 2.2 Hz, 8H, H^{m}), 9.18 (dd, J = 5.8, 1.3 Hz, 8H, H^{l}), 8.31 – 8.29 (m, 8H, H^{j}), 7.72 (dd, J = 8.0, 5.7 Hz, 8H, H^{k}), 7.56 (s, 16H, H^{g}), 7.07 (d, J = 3.0 Hz, 16H, H^{c}), 2.19 (br. s, 48H, H^{n}).

^{19}F and ^{13}C NMR: Due to overlapping peaks and low concentration of the cages the individual peaks cannot unambiguously be assigned to either [Pd$_3$(*E*-**1**)$_6$](BF$_4$)$_6$ or [Pd$_4$(*E*-**1**)$_8$](BF$_4$)$_8$.

5.2.1 1D and 2D NMR characterization of [Pd$_3$(*E*-1)$_6$](BF$_4$)$_6$ and [Pd$_4$(*E*-1)$_8$](BF$_4$)$_8$ in MeCN-d_3

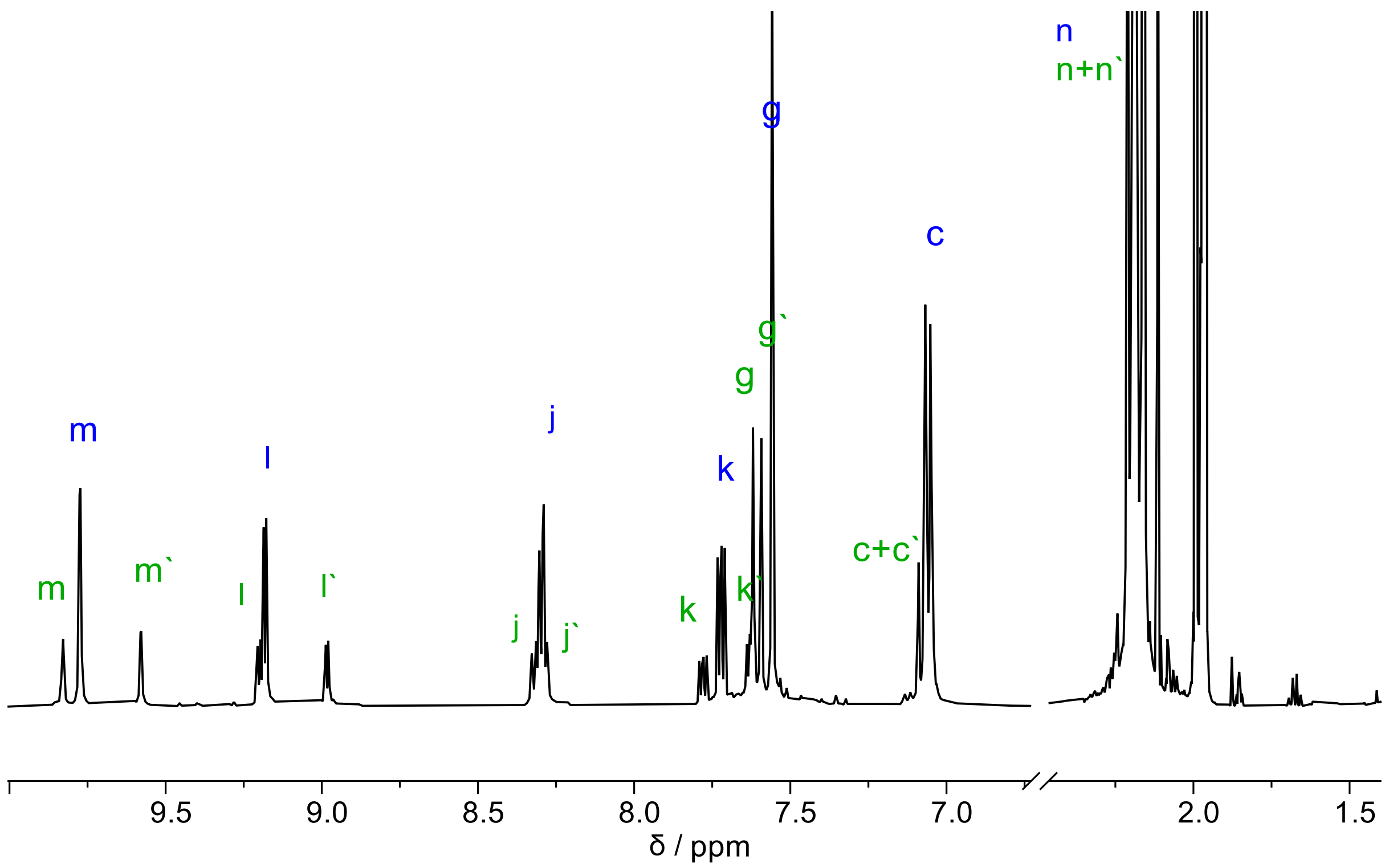

Figure S70. ^{1}H NMR (600 MHz, 298 K, MeCN-d_3) spectrum of the mixture of [Pd$_3$(*E*-**1**)$_6$](BF$_4$)$_6$ and [Pd$_4$(*E*-**1**)$_8$](BF$_4$)$_8$.

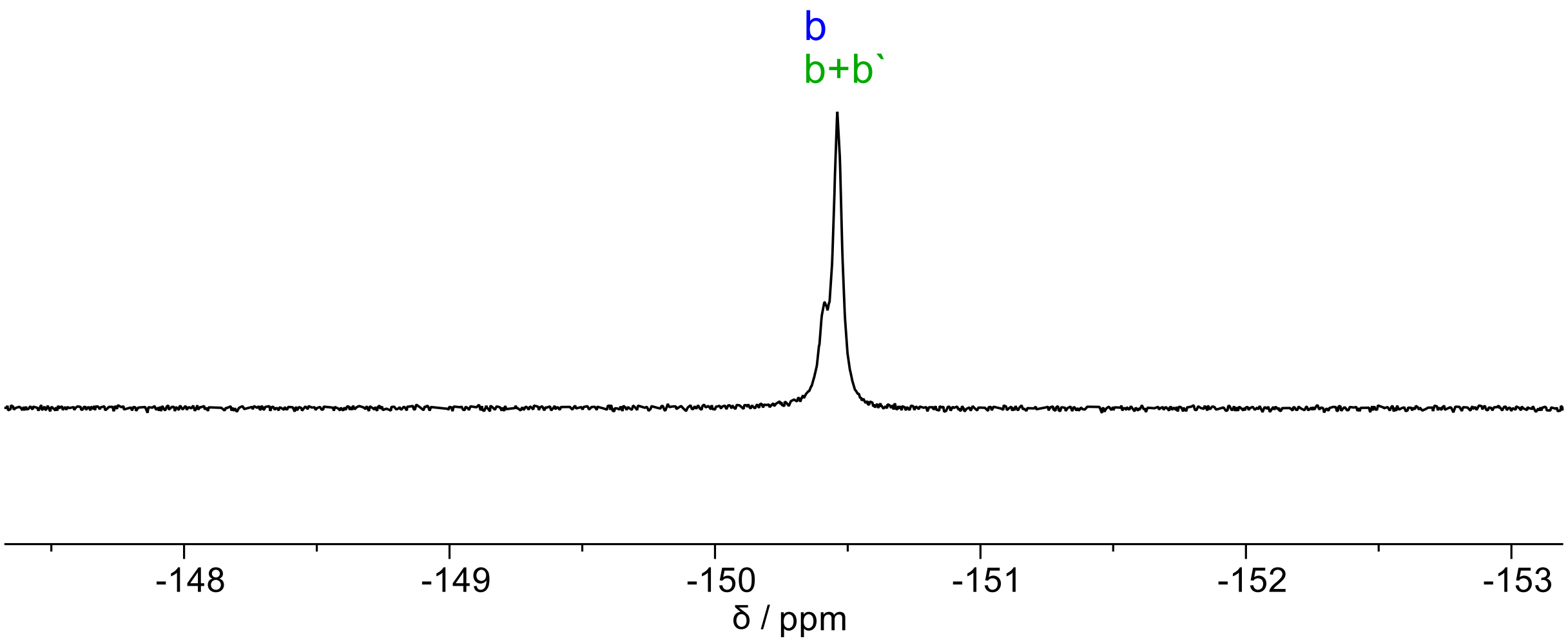

Figure S71. Partial ^{19}F NMR (565 MHz, 298 K, MeCN-d_3) spectrum of $[Pd_3(E\text{-}\mathbf{1})_6](BF_4)_6$ and $[Pd_4(E\text{-}\mathbf{1})_8](BF_4)_8$.

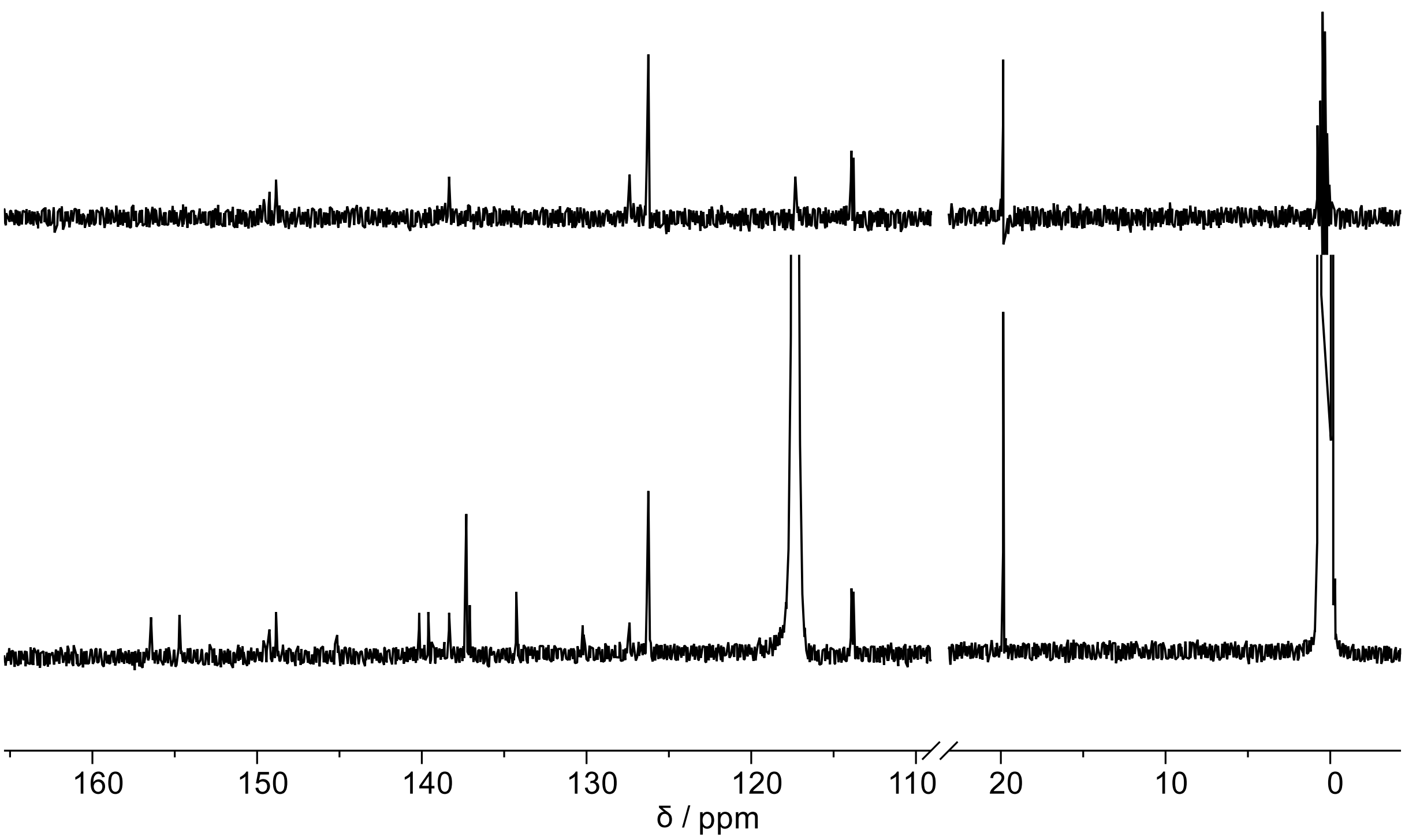

Figure S72. ^{13}C{^{1}H} and 135-DEPT NMR (151 MHz, 298 K, DMSO-d_6) spectrum of $[Pd_3(E\text{-}\mathbf{1})_6](BF_4)_6$ and $[Pd_4(E\text{-}\mathbf{1})_8](BF_4)_8$.

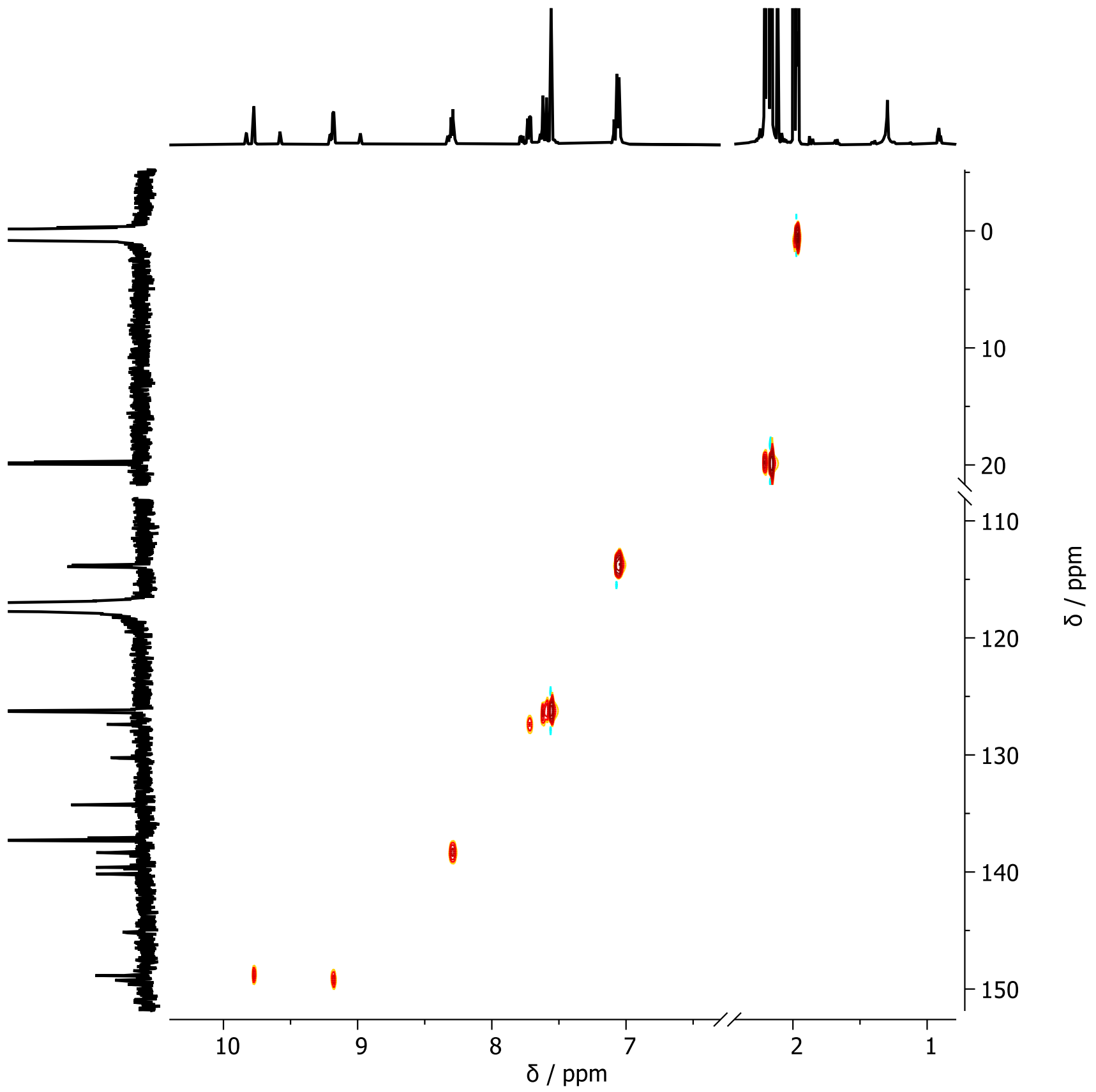

Figure S73. ^{1}H-^{13}C HSQC NMR (600 MHz, 151 MHz, 298 K, MeCN-d_3) spectrum of $[Pd_3(E\text{-}\mathbf{1})_6](BF_4)_6$ and $[Pd_4(E\text{-}\mathbf{1})_8](BF_4)_8$.

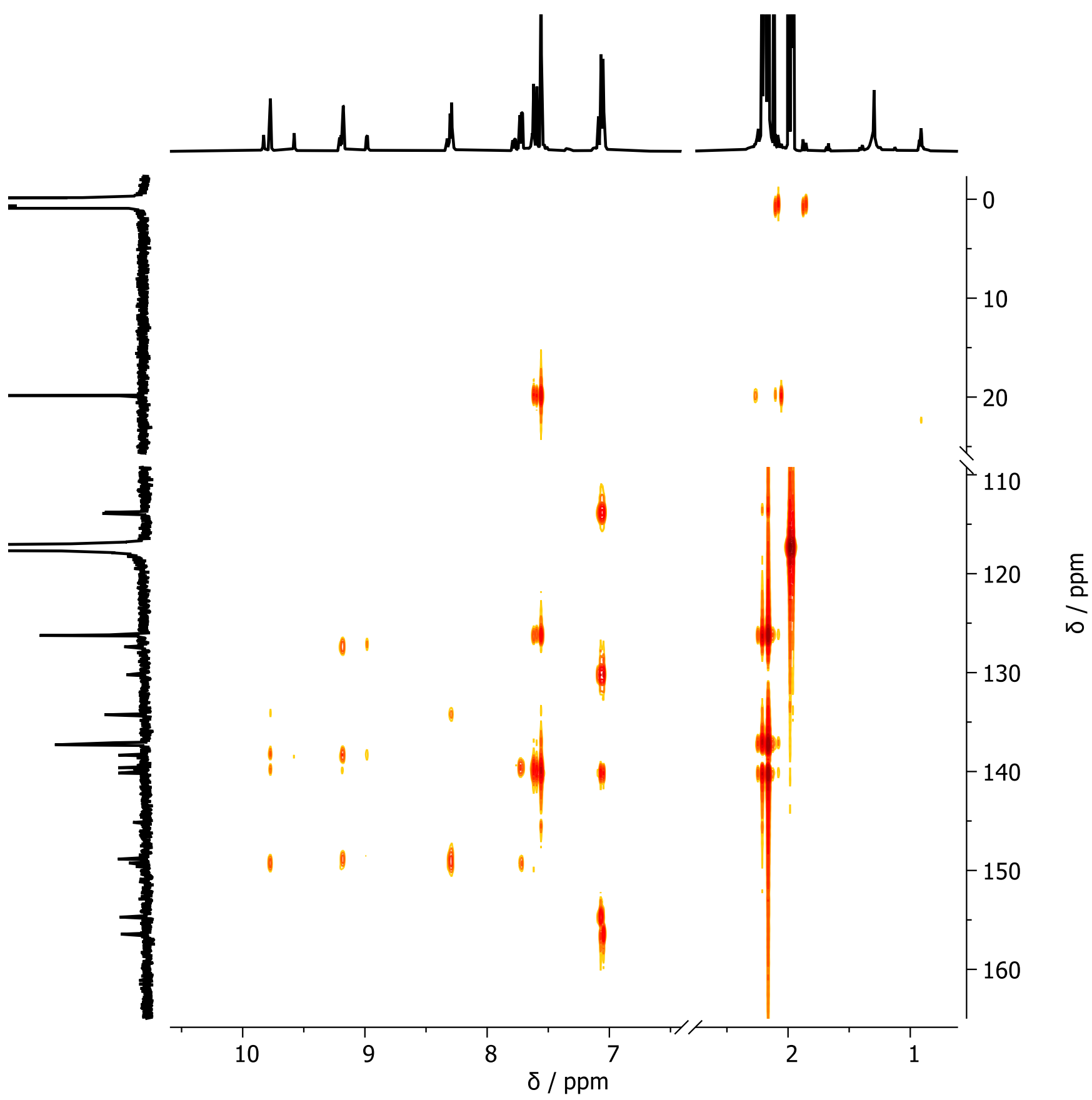

Figure S74. ^{1}H-^{13}C HMBC NMR (600 MHz, 151 MHz, 298 K, MeCN-d_3) spectrum of $[Pd_3(E\text{-}\mathbf{1})_6](BF_4)_6$ and $[Pd_4(E\text{-}\mathbf{1})_8](BF_4)_8$.

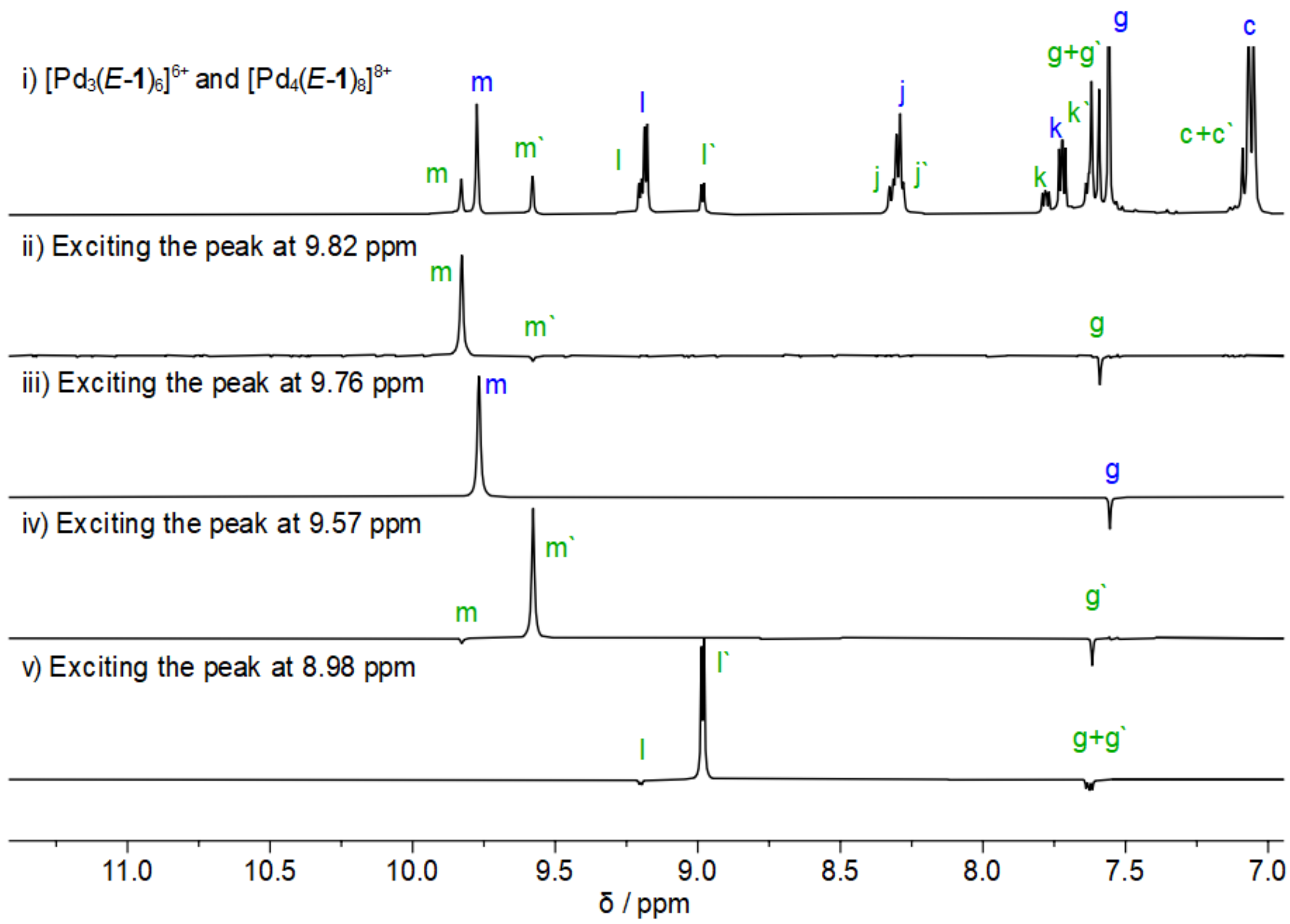

Figure S75. ^{1}H NMR (600 MHz, 151 MHz, 298 K, MeCN-*d*$_3$) spectrum of i) [Pd$_3$(*E*-**1**)$_6$](BF$_4$)$_6$ and [Pd$_4$(*E*-**1**)$_8$](BF$_4$)$_8$, and 1D ROESY NMR of the same sample with selective excitation of the peaks at ii) 9.82 ppm, iii) 9.76 ppm, iv) 9.57 ppm, and v) 8.98 ppm.

5.2.2 ESI-MS of the mixture of $[Pd_3(E\text{-}\mathbf{1})_6](BF_4)_6$ and $[Pd_4(E\text{-}\mathbf{1})_8](BF_4)_8$ in MeCN-d_3

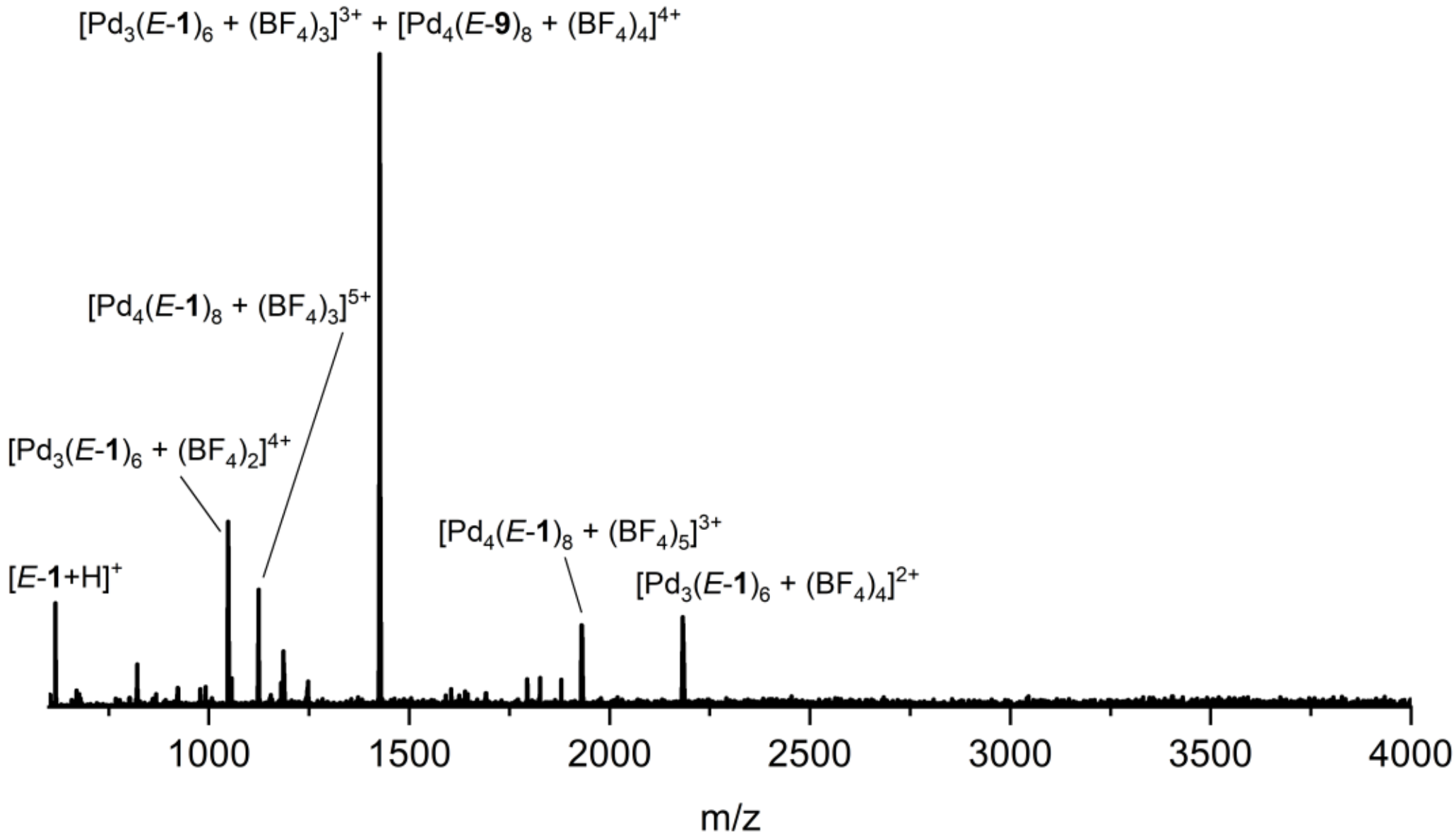

Figure S76. ESI-MS spectrum of the mixture of $[Pd_3(E\text{-}\mathbf{1})_6](BF_4)_6$ and $[Pd_4(E\text{-}\mathbf{1})_8](BF_4)_8$ ([Pd] = 2.3 mM) in MeCN-d_3.

Table S 23. Zoom scans of select ESI-MS peaks, with simulated isotope patterns of $[Pd_3(E\text{-}\mathbf{1})_6](BF_4)_6$ ([Pd] = 2.3 mM) in MeCN-d_3.

$[Pd_3(E\text{-}\mathbf{1})_6 + (BF_4)_4]^{2+}$

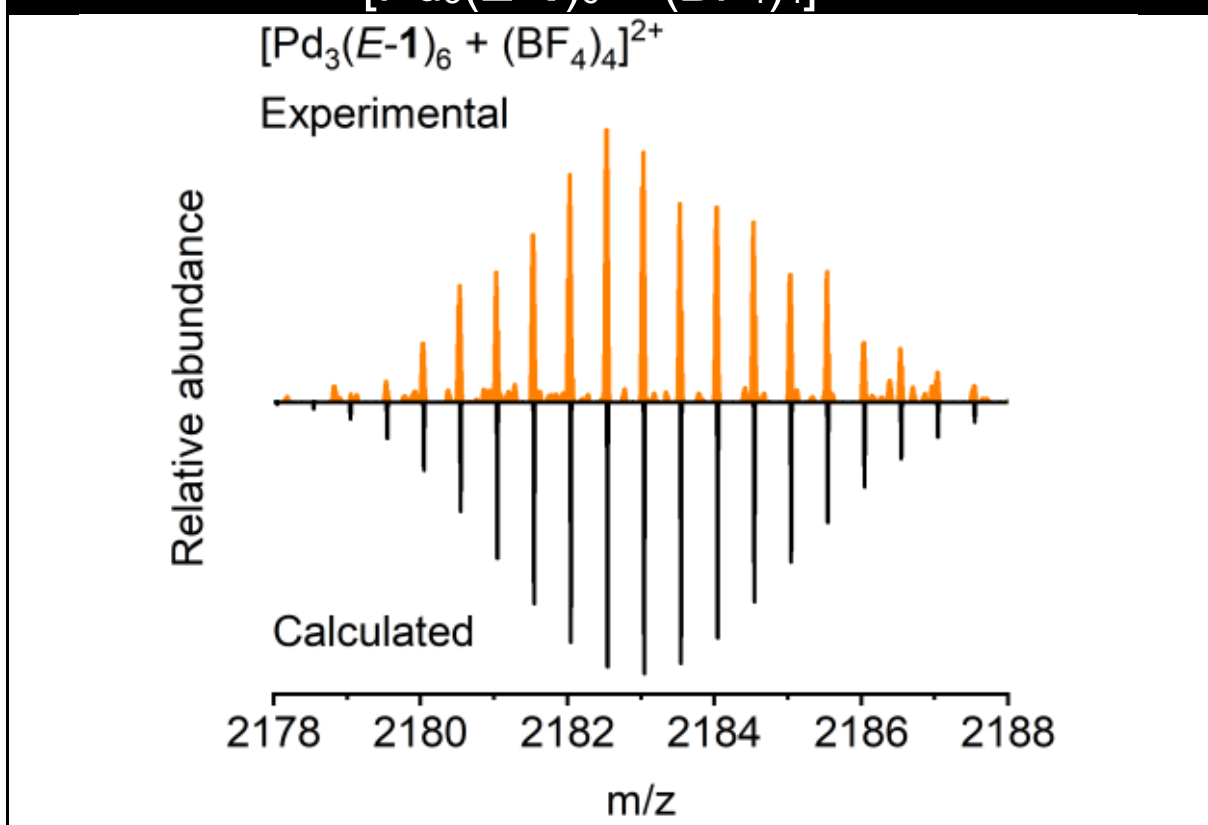

$[Pd_3(E\text{-}\mathbf{1})_6 + (BF_4)_3]^{3+}$

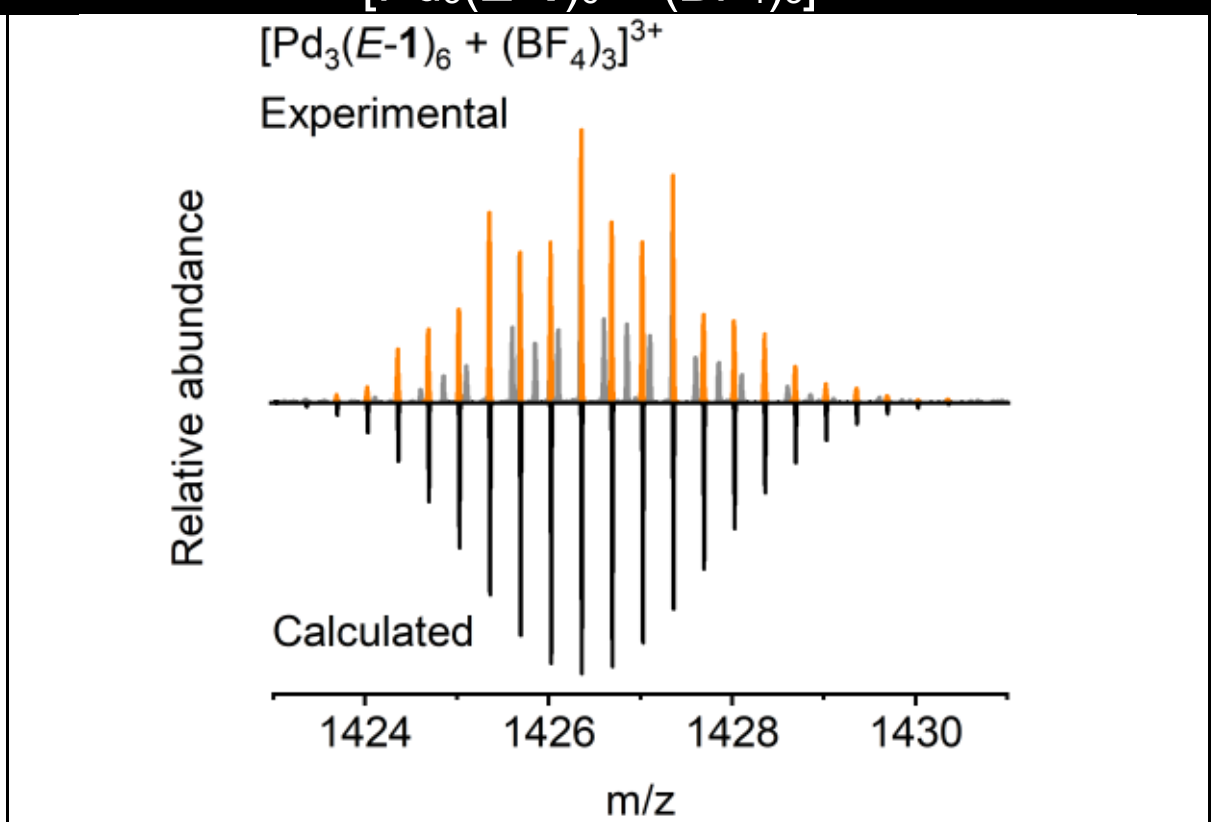

$[Pd_3(E\text{-}\mathbf{1})_6 + (BF_4)_2]^{4+}$

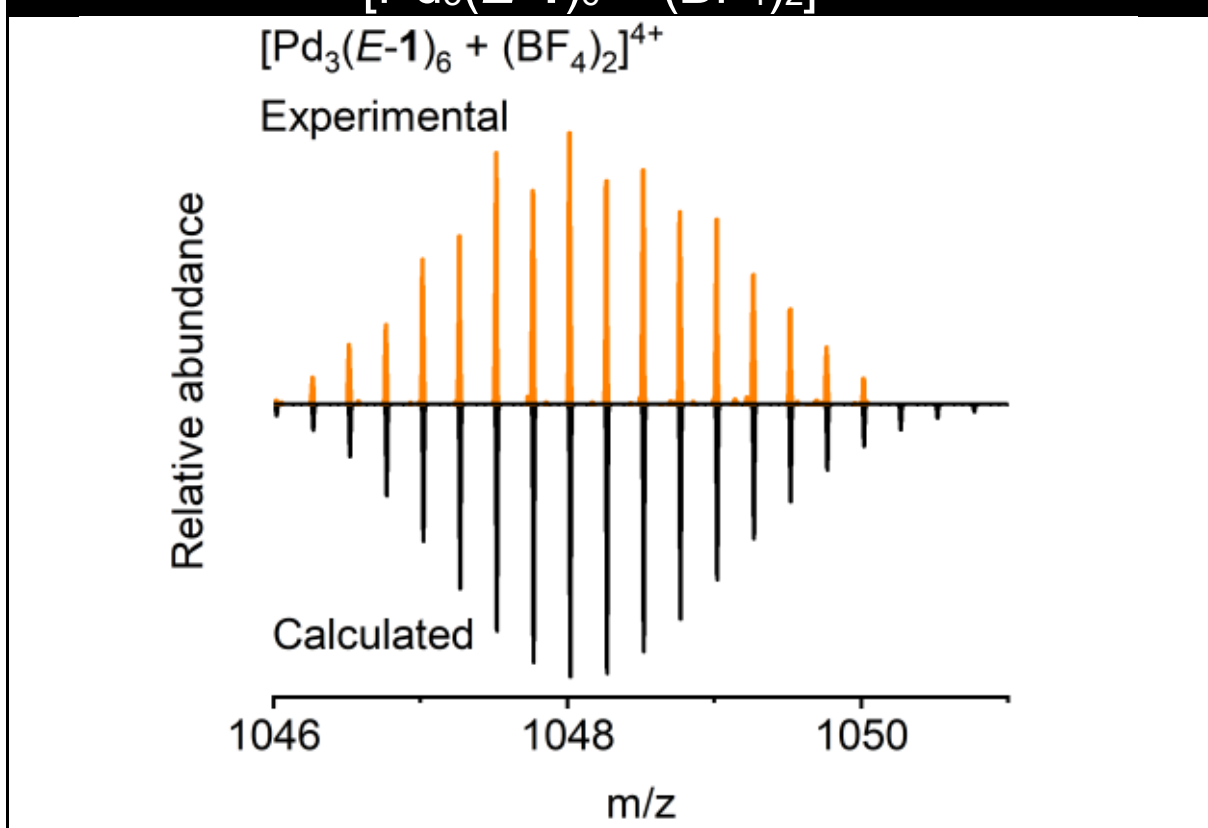

$[Pd_3(E\text{-}\mathbf{1})_6 + (BF_4)]^{5+}$

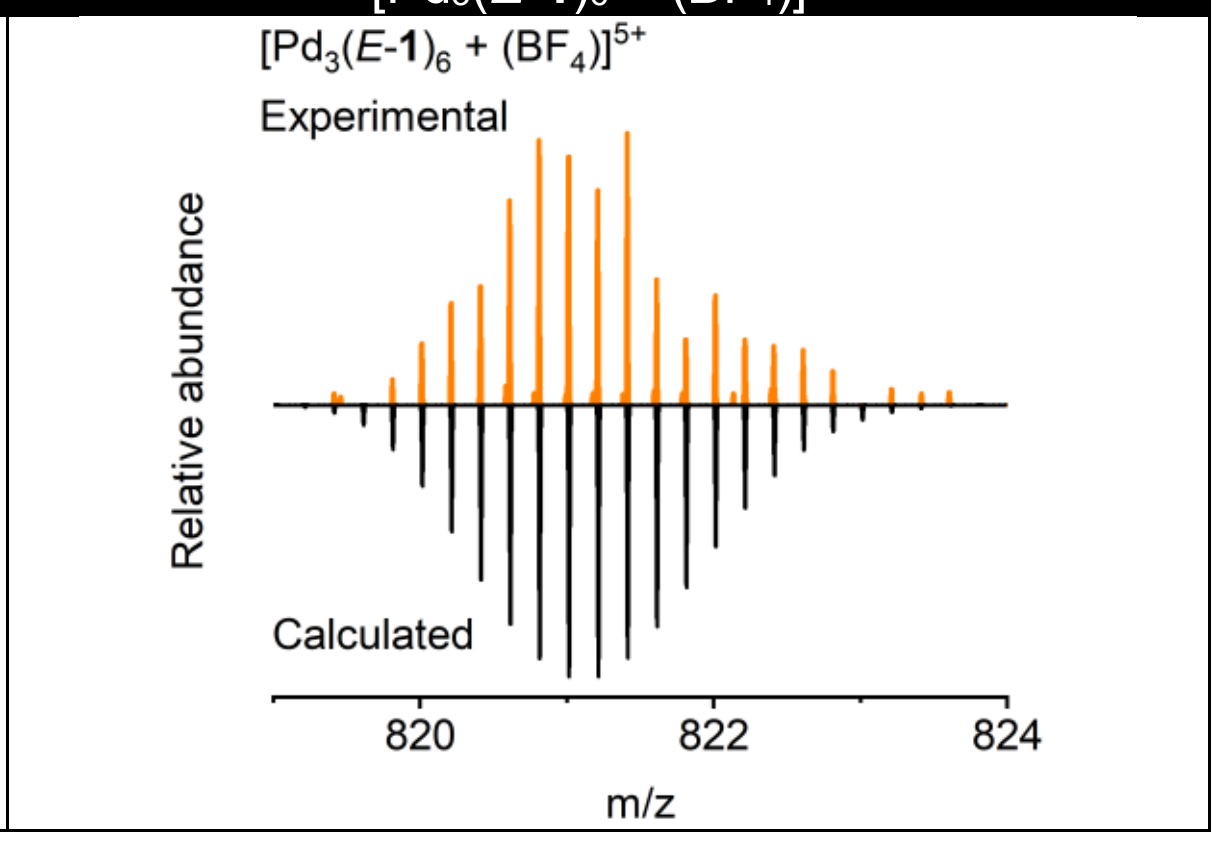

Table S 24. Zoom scans of select ESI-MS peaks, with simulated isotope patterns of $[Pd_4(E\text{-}\mathbf{1})_8](BF_4)_8$ ([Pd] = 2.3 mM) in MeCN-d_3.

$[Pd_4(E\text{-}\mathbf{1})_8 + (BF_4)_5]^{3+}$

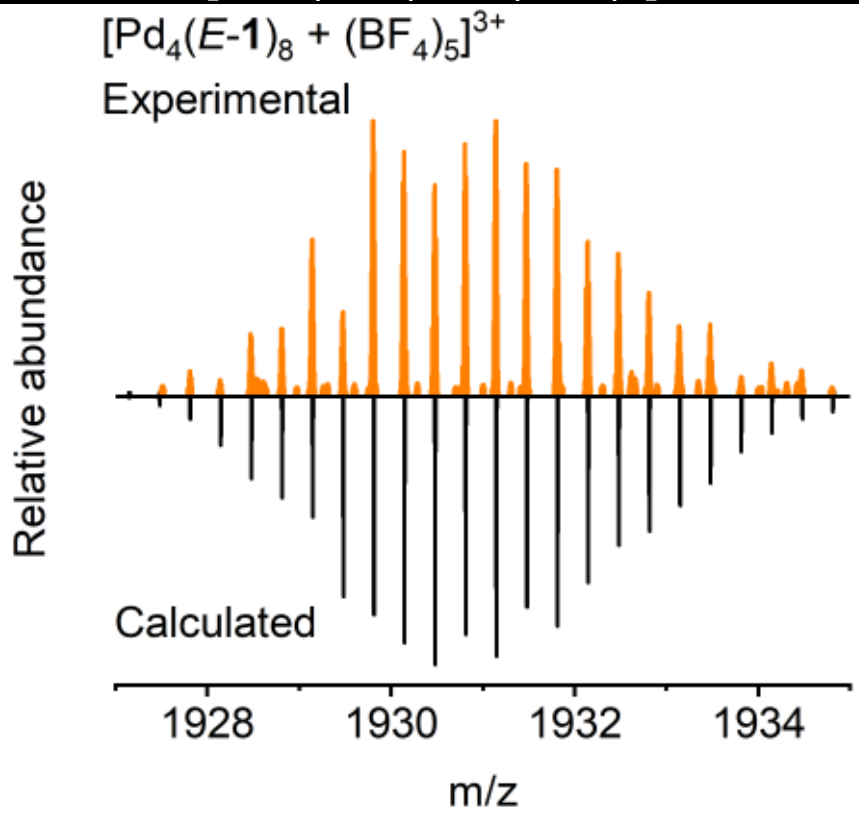

$[Pd_4(E\text{-}\mathbf{1})_8 + (BF_4)_4]^{4+}$

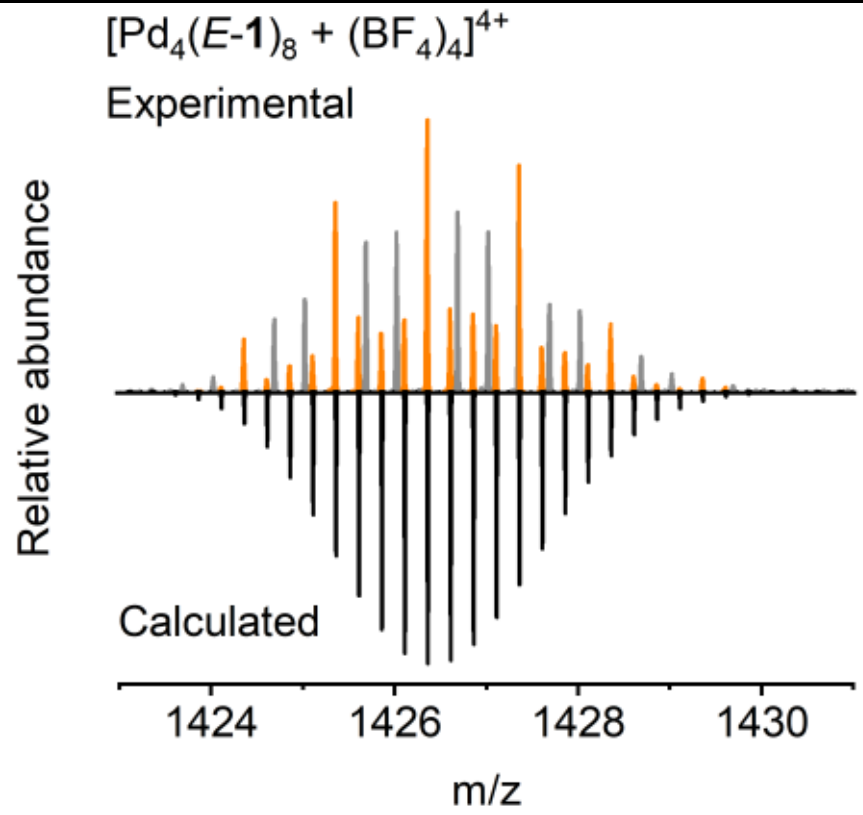

$[Pd_4(E\text{-}\mathbf{1})_8 + (BF_4)_3]^{5+}$

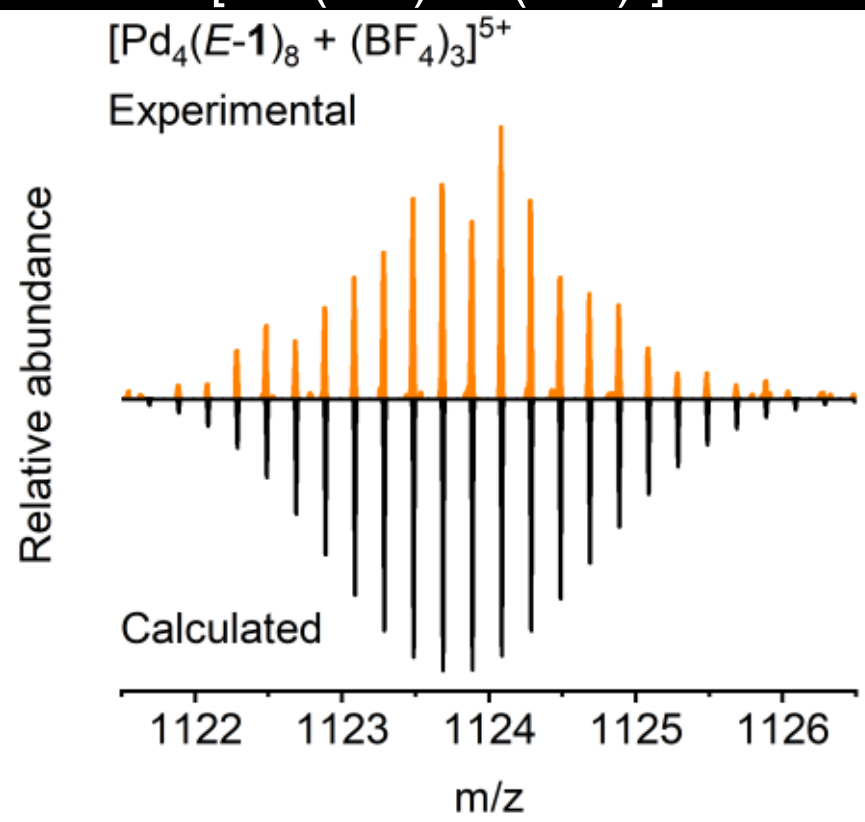

$[Pd_4(E\text{-}\mathbf{1})_8 + (BF_4)_2]^{6+}$

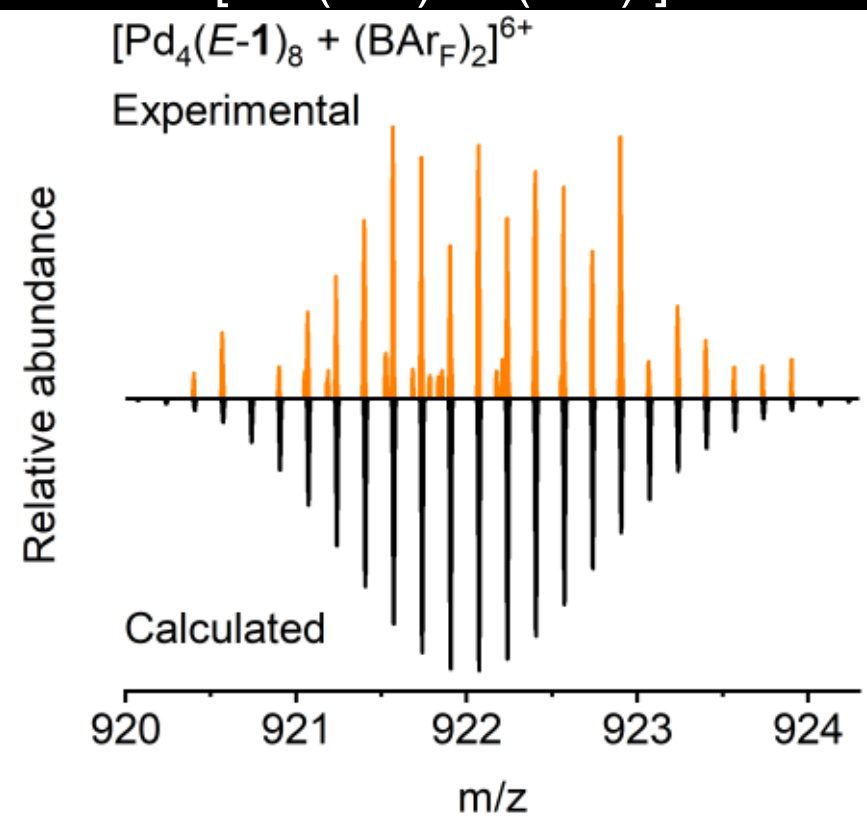

5.3 Interconversion of $[Pd_3(E\text{-}\mathbf{1})_6](BF_4)_6$ and $[Pd_4(E\text{-}\mathbf{1})_8](BF_4)_8$ into $[Pd_3(E\text{-}\mathbf{1})_6](BAr_F)_6$ and vice versa by counter anion exchange

After showing in section 5.1 and 5.2 that the homoleptic self-assemblies of ligand *E*-**1** in combination with palladium(II) are highly dependent on the counter anion, we investigated the ability of interconverting the mixture of $[Pd_3(E\text{-}\mathbf{1})_6](BF_4)_6$ and $[Pd_4(E\text{-}\mathbf{1})_8](BF_4)_8$ into $[Pd_3(E\text{-}\mathbf{1})_6](BAr_F)_6$ and vice versa by exchanging the respective counter anion.

5.3.1 Interconverting $[Pd_3(E\text{-}\mathbf{1})_6](BAr_F)_6$ into $[Pd_3(E\text{-}\mathbf{1})_6](BF_4)_6$ and $[Pd_4(E\text{-}\mathbf{1})_8](BF_4)_8$ by adding $NaBF_4$

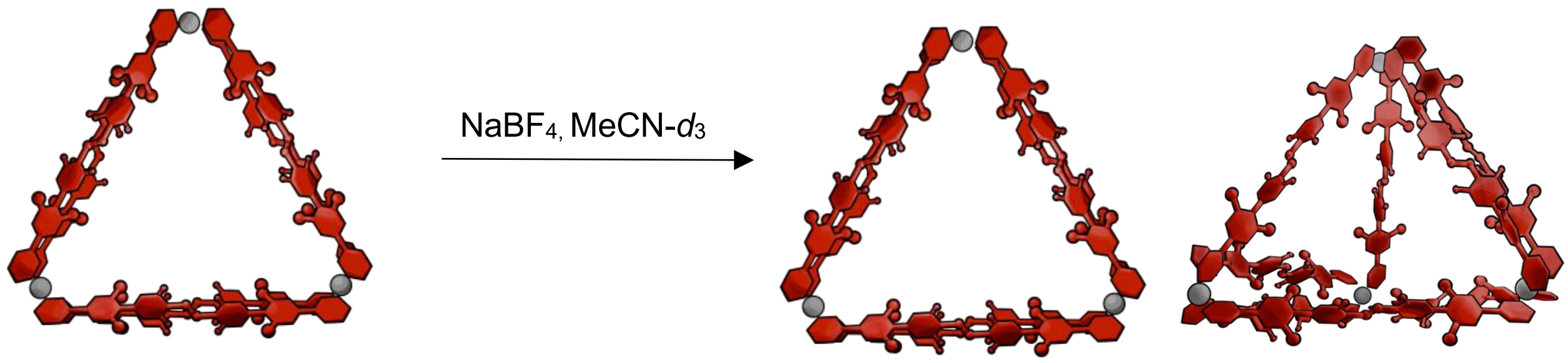

A sample of $[Pd_3(E\text{-}\mathbf{1})_6](BAr_F)_6$ was prepared in MeCN-d_3 ([Pd] = 2.3 mM, 500 µL, 1.0 equiv.) according to the procedure described in section 5.1. Furthermore, a stock solution of $NaBF_4$ (32.2 mM in MeCN-d_3) was prepared.

The $NaBF_4$ stock solution was added stepwise (0.1 equiv. / 0.2 equiv. / 0.5 equiv. / 1.0 equiv. / 2.0 equiv. / 3.0 equiv. / 5.0 equiv. / 10.0 equiv.) to the $[Pd_3(E\text{-}\mathbf{1})_6](BAr_F)_6$ and after each $NaBF_4$ addition, the sample was allowed to equilibrate at 50 °C for 30 minutes, before ^{1}H and ^{19}F NMR spectra were recorded (Figure S77 and Figure S78).

Already upon the addition of 0.1 equiv. $NaBF_4$, a downfield shift of H^m in the ^{1}H NMR spectrum was observed, which increased progressively with further additions of $NaBF_4$. After adding 10.0 equiv. of $NaBF_4$ H^m has shifted downfield by total 0.26 ppm. After adding 0.2 equiv. of NaBF4 we could observe two new peaks to the left and to the right of H^m, corresponding to H^m and $H^{m`}$ of the newly formed distorted tetrahedron $[Pd_4(E\text{-}\mathbf{1})_8](BF_4)_8$. The intensity of these peaks increased as well with further additions of $NaBF_4$. Similar peak shifts were observed in the ^{19}F NMR spectra. The BF_4 peak moved from -148.8 ppm (0.1 equiv. $NaBF_4$) to -152.0 ppm (10.0 equiv. $NaBF_4$). There is more than one BF_4 peak visible which indicates multiple binding events occurring, presumably in- and outside the now two cages present. The ^{1}H NMR titration data were fitted to a 1:1 fast-exchanging binding model using a nonlinear least-square algorithm built in the BindFit website.[7-8] Using this fitting, the association constant of BF_4^- was calculated to be K_a = 1453 M^{-1}.

Comparing the NMR spectrum of the $[Pd_3(E\text{-}\mathbf{1})_6](BAr_F)_6$ sample upon addition of the 10.0 equiv. of $NaBF_4$ (Figure S79ii) with an NMR spectrum of a mixture of $[Pd_3(E\text{-}\mathbf{1})_6](BF_4)_6$ and $[Pd_4(E\text{-}\mathbf{1})_8](BF_4)_8$ (Figure S79iii) reveals that these two NMR spectra are identical. By titrating $NaBF_4$ into the $[Pd_3(E\text{-}\mathbf{1})_6](BAr_F)_6$ we were able to exchange the counter anion and to interconvert the $[Pd_3(E\text{-}\mathbf{1})_6](BAr_F)_6$ cage into a mixture of $[Pd_3(E\text{-}\mathbf{1})_6](BF_4)_6$ and $[Pd_4(E\text{-}\mathbf{1})_8](BF_4)_8$.

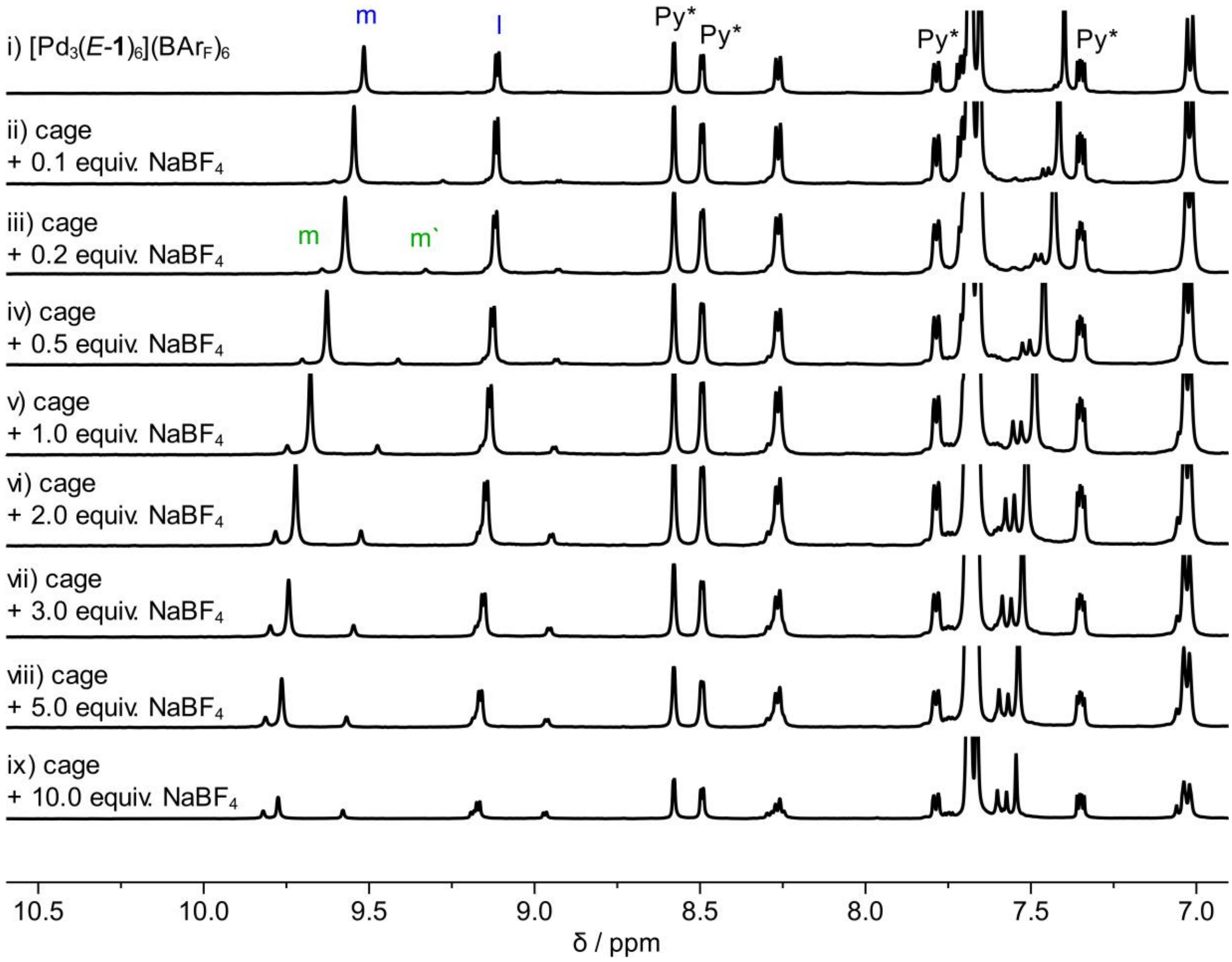

Figure S77. Partial ^{1}H NMR spectra (600 MHz, 298 K, MeCN-d_3) of i) a mixture of $[Pd_3(E\text{-}\mathbf{1})_6](BAr_F)_6$ ([Pd] = 2.3 mM, 500 µL, 1.0 equiv.), and after the addition of ii) 0.1 equiv. $NaBF_4$ (4 µL stock solution), iii) 0.2 equiv. $NaBF_4$ (8 µL stock solution), iv) 0.5 equiv. $NaBF_4$ (20 µL stock solution), v) 1.0 equiv. $NaBF_4$ (40 µL stock solution), vi) 2.0 equiv. $NaBF_4$ (80 µL stock solution), vii) 3.0 equiv. $NaBF_4$ (120 µL stock solution), viii) 5.0 equiv. $NaBF_4$ (200 µL stock solution), and ix) 10.0 equiv. $NaBF_4$ (400 µL stock solution).

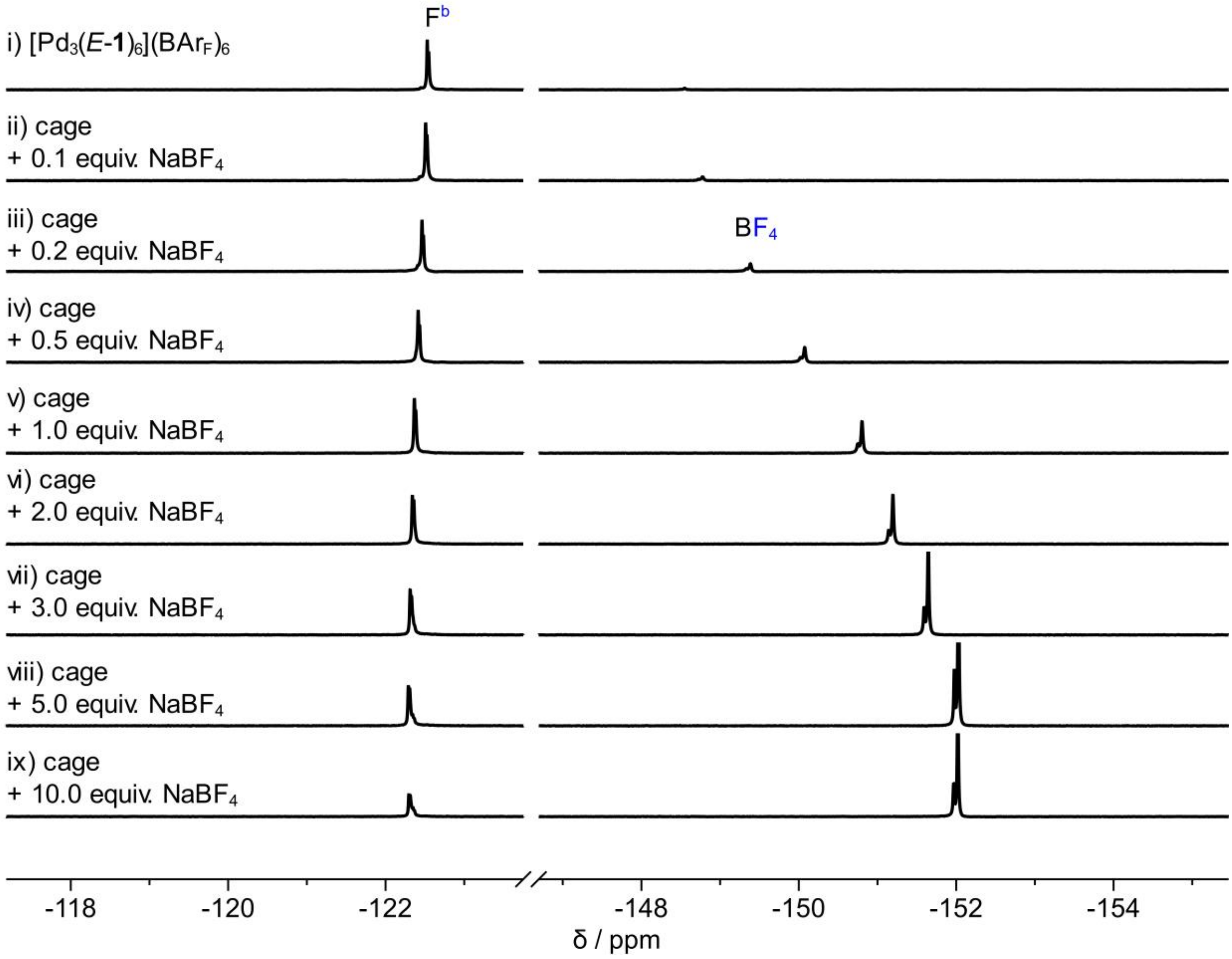

Figure S78. Partial ^{19}F NMR spectra (565 MHz, 298 K, MeCN-d_3) of i) a mixture of $[Pd_3(E\text{-}\mathbf{1})_6](BAr_F)_6$ ([Pd] = 2.3 mM, 500 µL, 1.0 equiv.), and after the addition of ii) 0.1 equiv. $NaBF_4$ (4 µL stock solution), iii) 0.2 equiv. $NaBF_4$ (8 µL stock solution), iv) 0.5 equiv. $NaBF_4$ (20 µL stock solution), v) 1.0 equiv. $NaBF_4$ (40 µL stock solution), vi) 2.0 equiv. $NaBF_4$ (80 µL stock solution), vii) 3.0 equiv. $NaBF_4$ (120 µL stock solution), viii) 5.0 equiv. $NaBF_4$ (200 µL stock solution), and ix) 10.0 equiv. $NaBF_4$ (400 µL stock solution).

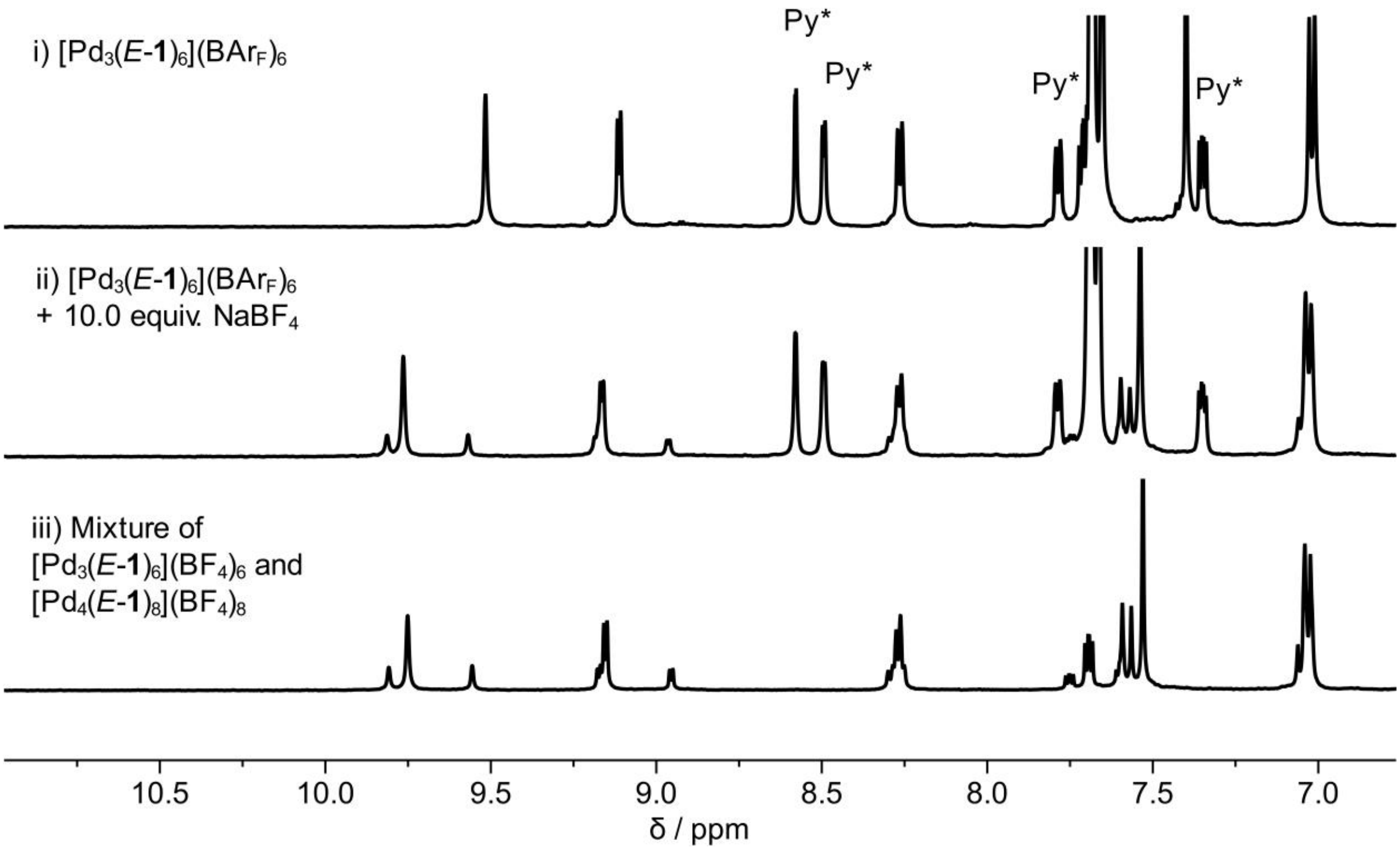

Figure S79. Partial ^{1}H NMR spectra (600 MHz, 298 K, MeCN-d_3) of i) $[Pd_3(E\text{-}\mathbf{1})_6](BAr_F)_6$ ([Pd] = 2.3 mM, 500 µL, 1.0 equiv.), ii) the previous sample after the addition of 10.0 equiv. $NaBF_4$ (400 µL stock solution), and iii) a mixture of $[Pd_3(E\text{-}\mathbf{1})_6](BF_4)_6$ and $[Pd_4(E\text{-}\mathbf{1})_8](BF_4)_8$ for comparison.

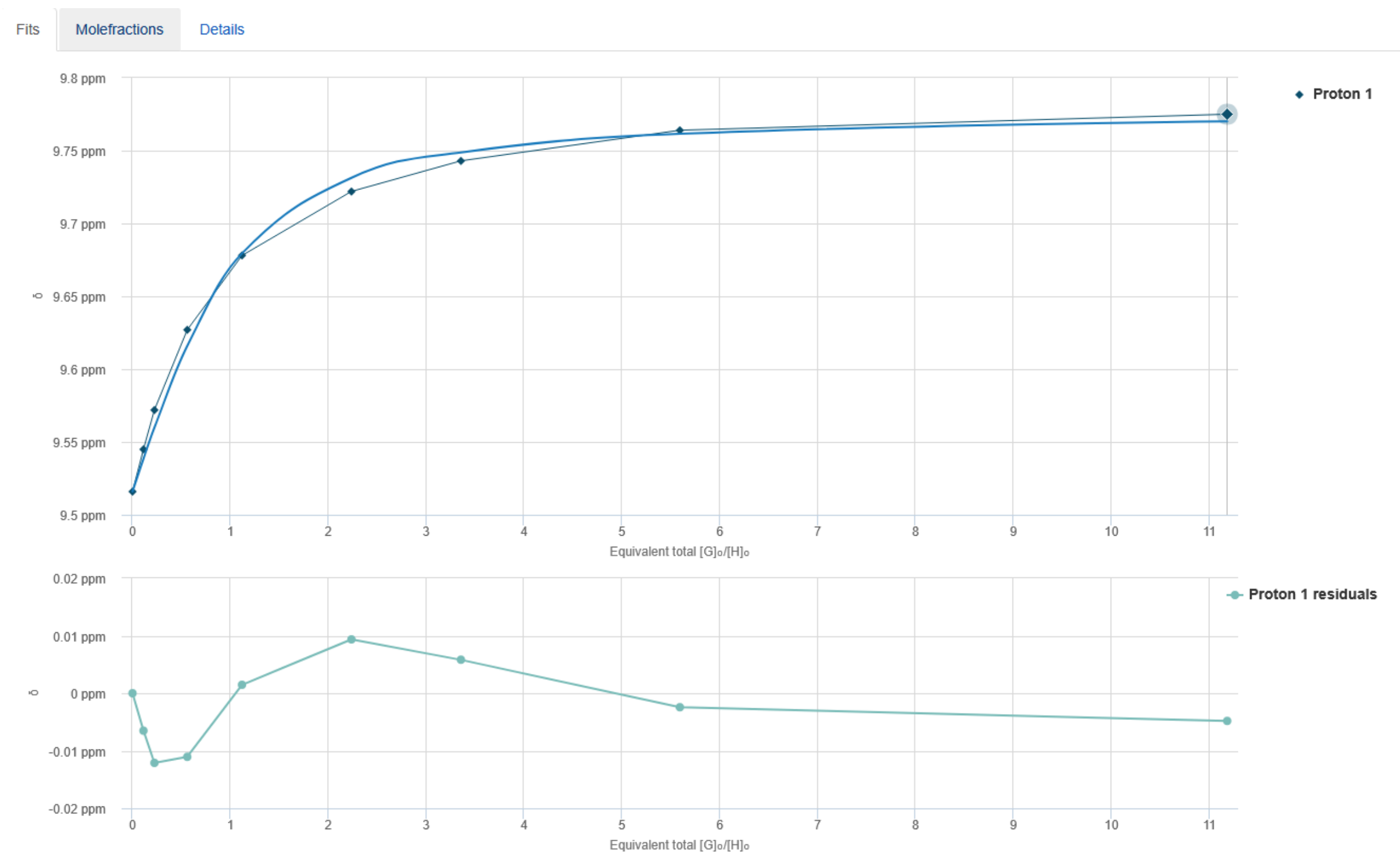

Figure S80. Association constant determination using BindFit[7-8] for ^{1}H NMR data in Figure S77 in equivalent total $[G]_0/[H]_0$ vs. ppm. For fitting, see http://app.supramolecular.org/bindfit/view/18bc5f6d-192b-43f1-b2be-03a1972df0d0

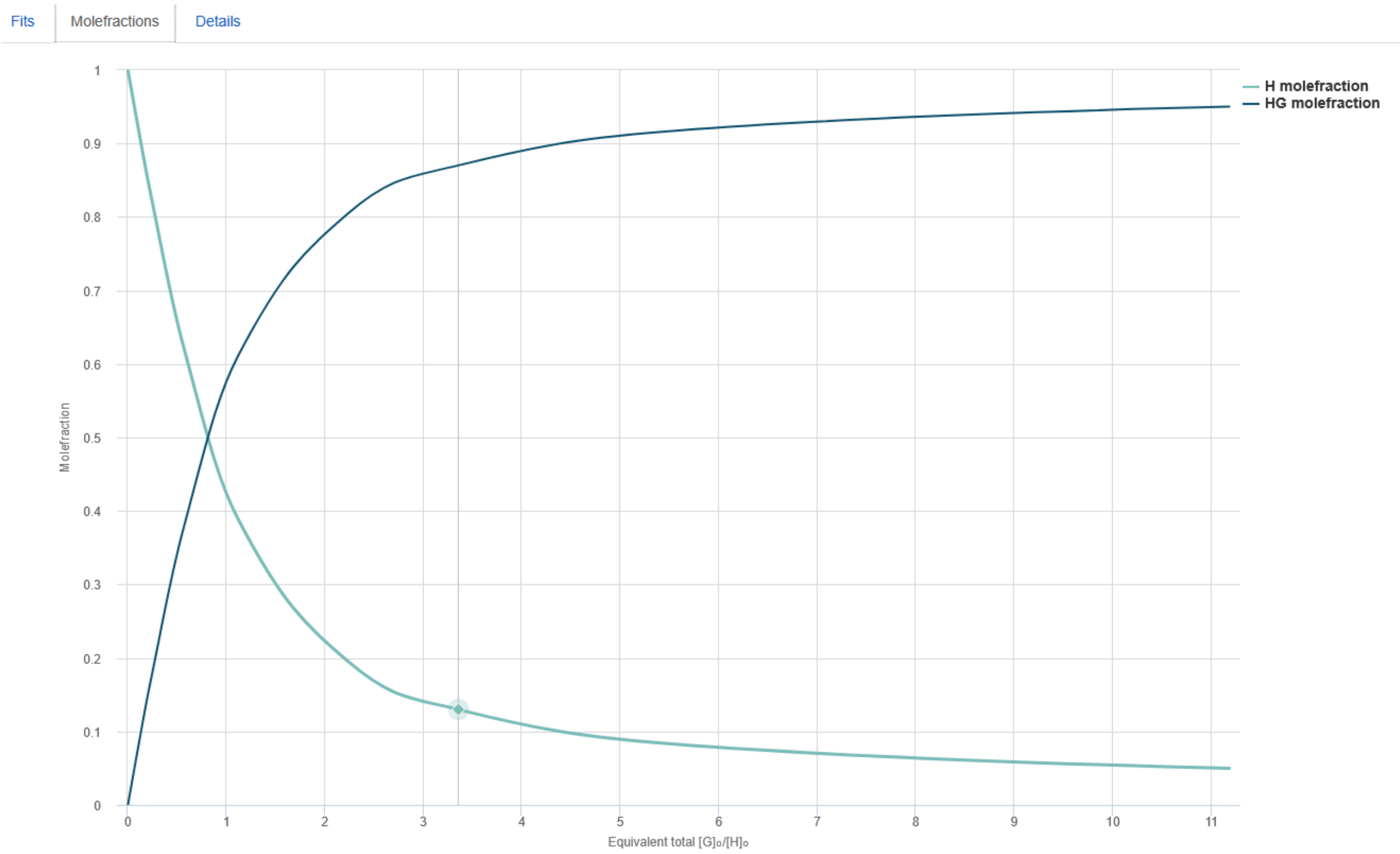

Figure S81. Association constant determination using BindFit[7-8] for ^{1}H NMR data in Figure S77 in equivalent total $[G]_0/[H]_0$ vs. molefraction. For fitting, see http://app.supramolecular.org/bindfit/view/18bc5f6d-192b-43f1-b2be-03a1972df0d0

5.3.2 Interconverting $[Pd_3(E\text{-}1)_6](BF_4)_6$ and $[Pd_4(E\text{-}1)_8](BF_4)_8$ into $[Pd_3(E\text{-}1)_6](BAr_F)_6$ by adding $NaBAr_F$

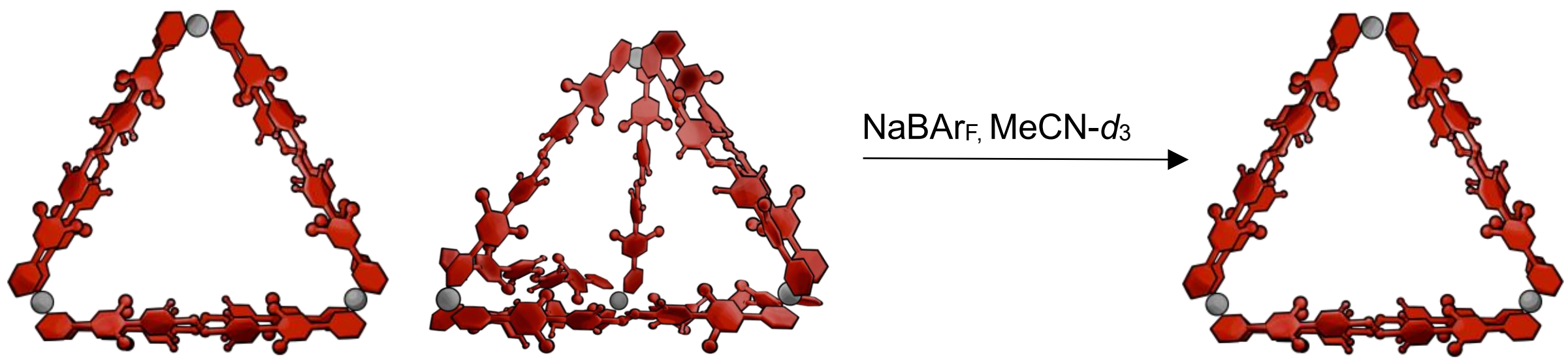

A sample of the cage mixture of $[Pd_3(E\text{-}\mathbf{1})_6](BF_4)_6$ and $[Pd_4(E\text{-}\mathbf{1})_8](BF_4)_8$ was prepared in MeCN-d_3 ([Pd] = 2.3 mM, 500 µL, 1.0 equiv.) according to the procedure described in section 5.2. Furthermore, a stock solution of $NaBAr_F$ (25.7 mM in MeCN-d_3) was prepared.

The $NaBAr_F$ stock solution was added stepwise (0.1 equiv. / 0.2 equiv. / 0.5 equiv. / 1.0 equiv. / 2.0 equiv. / 3.0 equiv. / 5.0 equiv. / 10.0 equiv.) to the cage mixture and after each addition, the sample was allowed to equilibrate at 50 °C for 30 minutes, before ^{1}H and ^{19}F NMR spectra were recorded.

The addition of $NaBAr_F$ to the cage mixture of $[Pd_3(E\text{-}\mathbf{1})_6](BF_4)_6$ and $[Pd_4(E\text{-}\mathbf{1})_8](BF_4)_8$ caused an upfield shift of all H^m, H^m and H^m in the ^{1}H NMR spectrum, which increased progressively with further additions of $NaBAr_F$. After adding 10.0 equiv. of $NaBAr_F$ H^m has shifted upfield by total -0.12 ppm. Similar peak shifts were observed in the ^{19}F NMR spectra. The BF_4 peak moved from -150.3 ppm (0.1 equiv. $NaBAr_F$) to -151.0 ppm (10.0 equiv. $NaBF_4$). The peak shifts after titrating $NaBAr_F$ to a cage mixture of $[Pd_3(E\text{-}\mathbf{1})_6](BF_4)_6$ and $[Pd_4(E\text{-}\mathbf{1})_8](BF_4)_8$ were less distinct than titrating $NaBF_4$ into $[Pd_3(E\text{-}\mathbf{1})_6](BAr_F)_6$. This suggests weaker binding of the BAr_F^- counter anion. This hypothesis was confirmed by fitting the ^{1}H NMR titration data to a 1:1 fast-exchanging binding model using a nonlinear least-square algorithm built in the BindFit website.[7-8] Using this fitting, the association constant of BAr_F^- was calculated to be K_a = 24 M⁻¹.

Comparing the NMR spectrum of the mixture of $[Pd_3(E\text{-}\mathbf{1})_6](BF_4)_6$ and $[Pd_4(E\text{-}\mathbf{1})_8](BF_4)_8$sample upon addition of the 10.0 equiv. of $NaBAr_F$ (Figure S84ii) with an NMR spectrum of $[Pd_3(E\text{-}\mathbf{1})_6](BAr_F)_6$ (Figure S84iii) reveals that these two NMR spectra are similar but not yet identical. Since BAr_F^- is binding much weaker to the self-assembled cages than BF_4^- higher amounts of BAr_F^- are needed to fully interconvert the different species into each other.

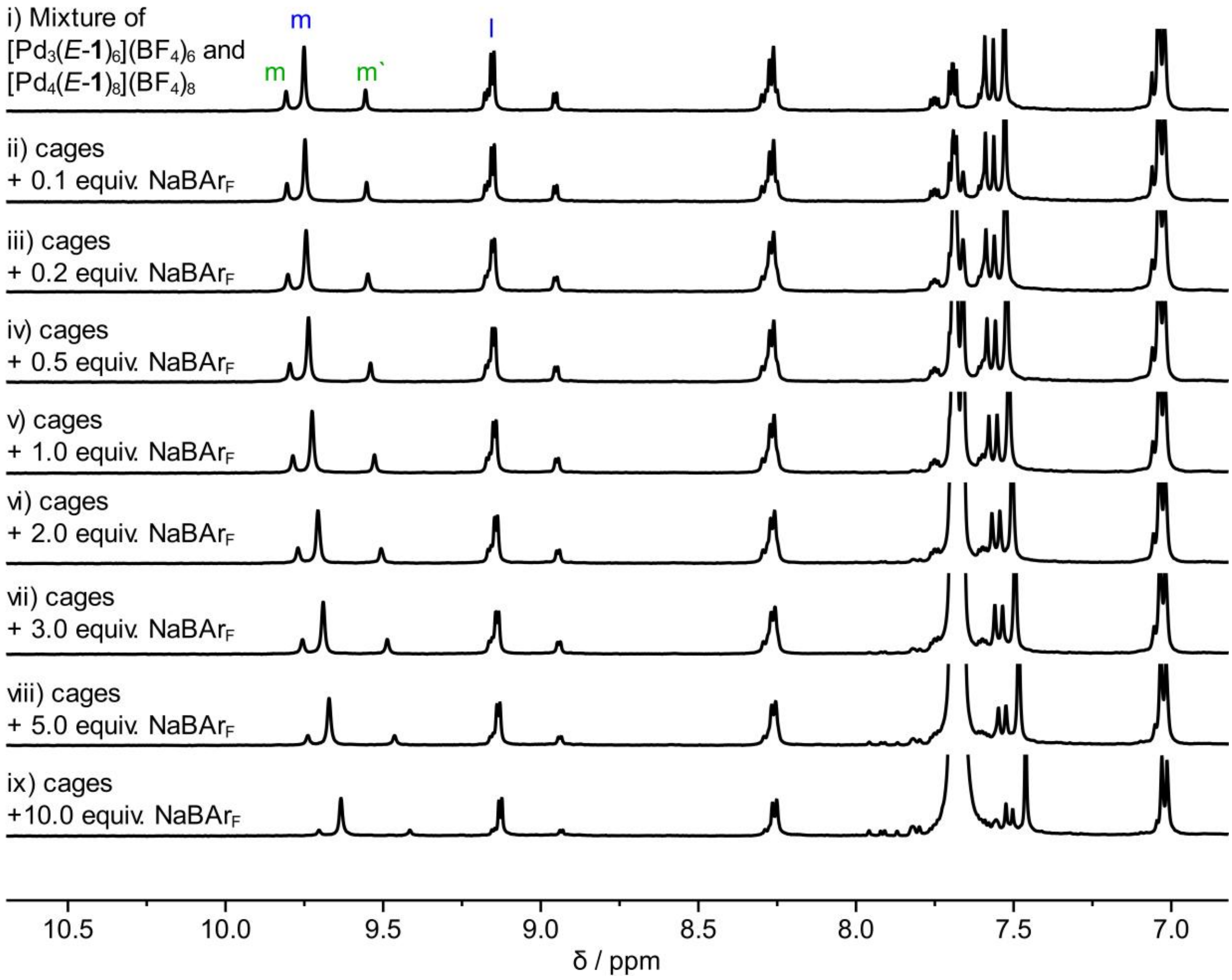

Figure S82. Partial ^{1}H NMR spectra (600 MHz, 298 K, MeCN-d_3) of i) a mixture of $[Pd_3(E\text{-}\mathbf{1})_6](BF_4)_6$ and $[Pd_4(E\text{-}\mathbf{1})_8](BF_4)_8$ ([Pd] = 2.3 mM, 500 µL, 1.0 equiv.), and after the addition of ii) 0.1 equiv. $NaBAr_F$ (5 µL stock solution), iii) 0.2 equiv. $NaBAr_F$ (10 µL stock solution), iv) 0.5 equiv. $NaBAr_F$ (25 µL stock solution), v) 1.0 equiv. $NaBAr_F$ (50 µL stock solution), vi) 2.0 equiv. $NaBAr_F$ (100 µL stock solution), vii) 3.0 equiv. $NaBAr_F$ (150 µL stock solution), viii) 5.0 equiv. $NaBAr_F$ (250 µL stock solution), and ix) 10.0 equiv. $NaBAr_F$ (500 µL stock solution).

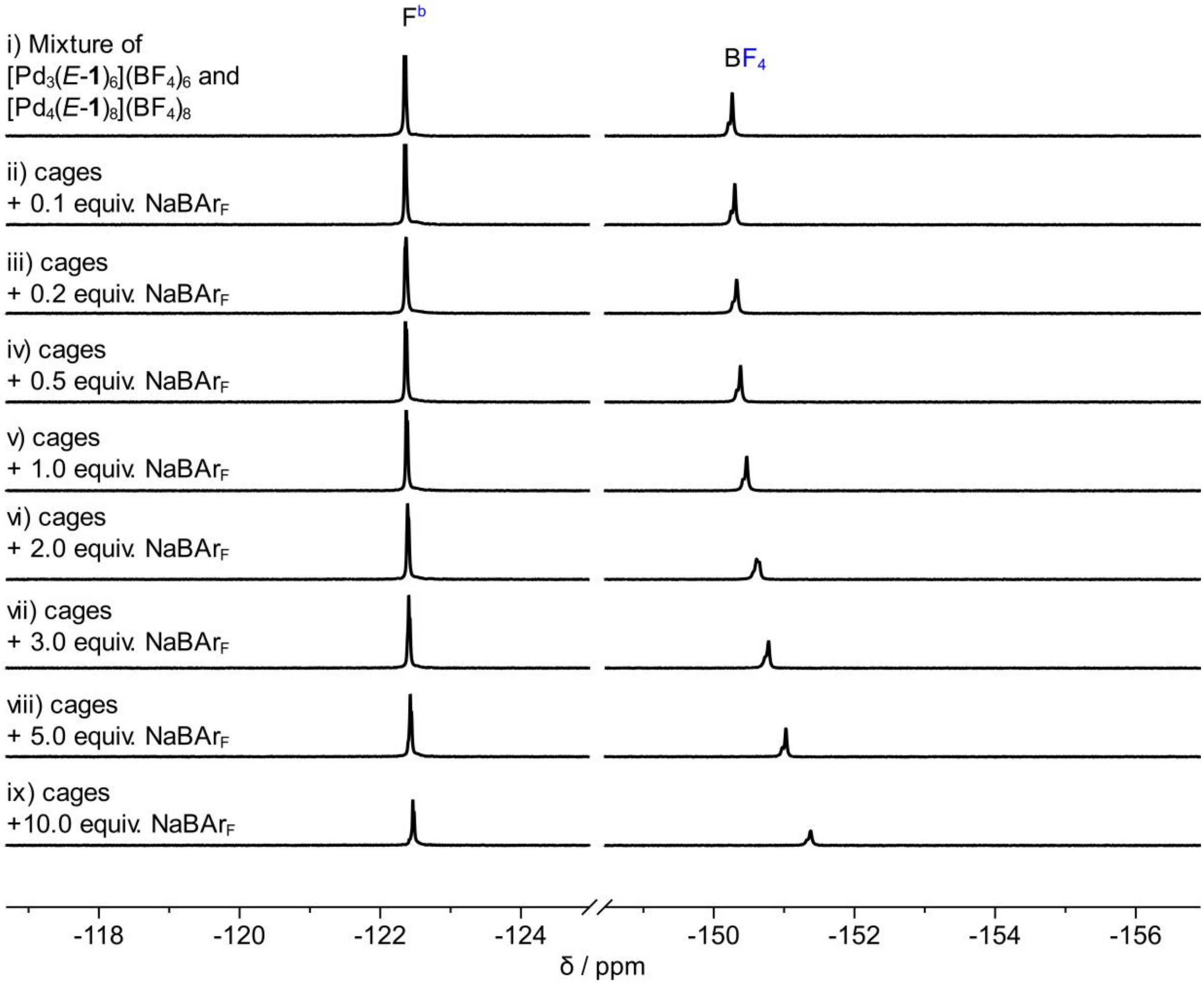

Figure S83. Partial ^{19}F NMR spectra (565 MHz, 298 K, MeCN-d_3) of i) a mixture of $[Pd_3(E\text{-}\mathbf{1})_6](BF_4)_6$ and $[Pd_4(E\text{-}\mathbf{1})_8](BF_4)_8$ ([Pd] = 2.3 mM, 500 µL, 1.0 equiv.), and after the addition of ii) 0.1 equiv. $NaBAr_F$ (5 µL stock solution), iii) 0.2 equiv. $NaBAr_F$ (10 µL stock solution), iv) 0.5 equiv. $NaBAr_F$ (25 µL stock solution), v) 1.0 equiv. $NaBAr_F$ (50 µL stock solution), vi) 2.0 equiv. $NaBAr_F$ (100 µL stock solution), vii) 3.0 equiv. $NaBAr_F$ (150 µL stock solution), viii) 5.0 equiv. $NaBAr_F$ (250 µL stock solution), and ix) 10.0 equiv. $NaBAr_F$ (500 µL stock solution).

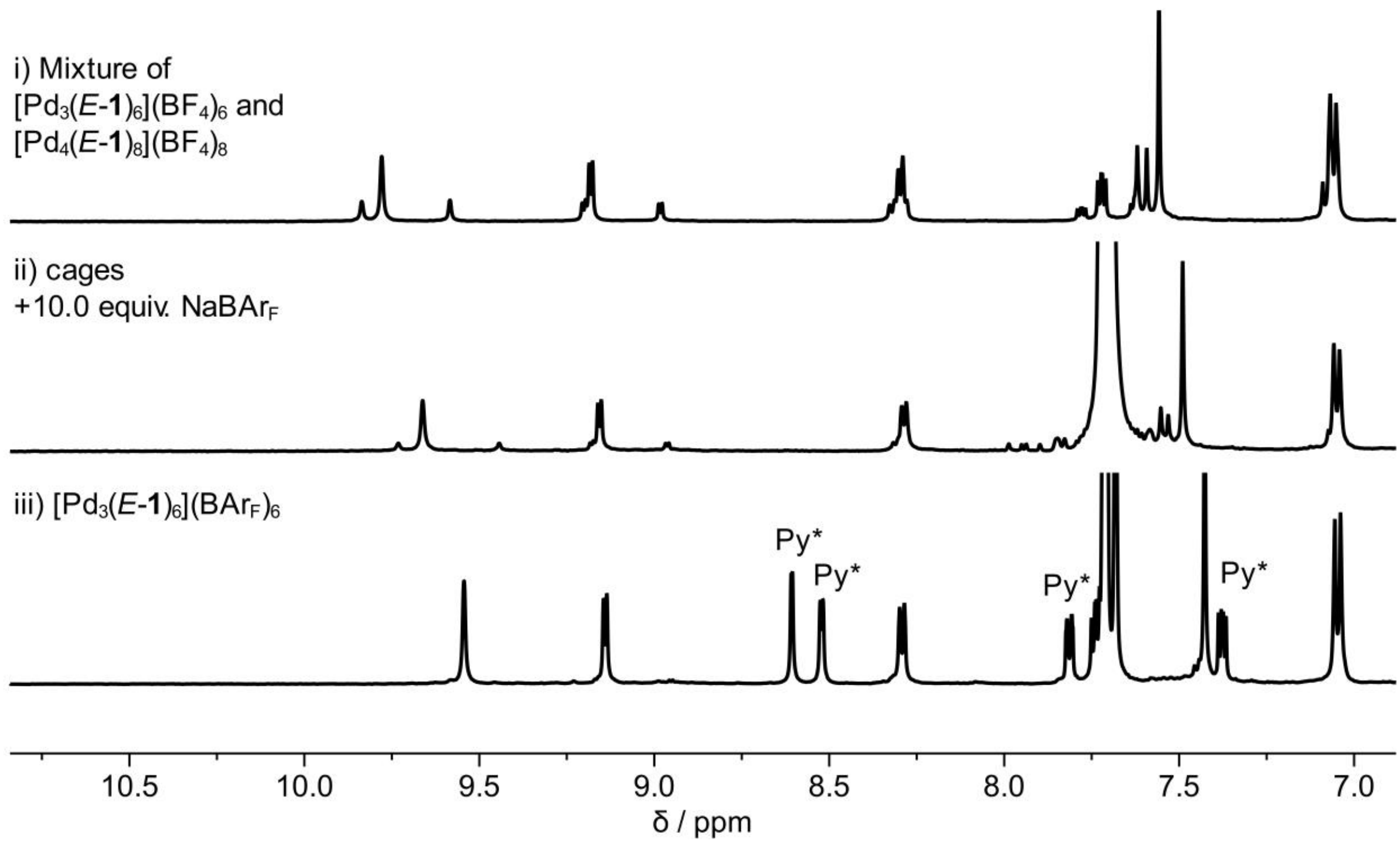

Figure S84. Partial ^{1}H NMR spectra (600 MHz, 298 K, MeCN-d_3) of i) a mixture of $[Pd_3(E\text{-}\mathbf{1})_6](BF_4)_6$ and $[Pd_4(E\text{-}\mathbf{1})_8](BF_4)_8$ ([Pd] = 2.3 mM, 500 µL, 1.0 equiv.), ii) the previous sample after the addition of 10.0 equiv. $NaBAr_F$ (500 µL stock solution), and iii) $[Pd_3(E\text{-}\mathbf{1})_6](BAr_F)_6$ for comparison.

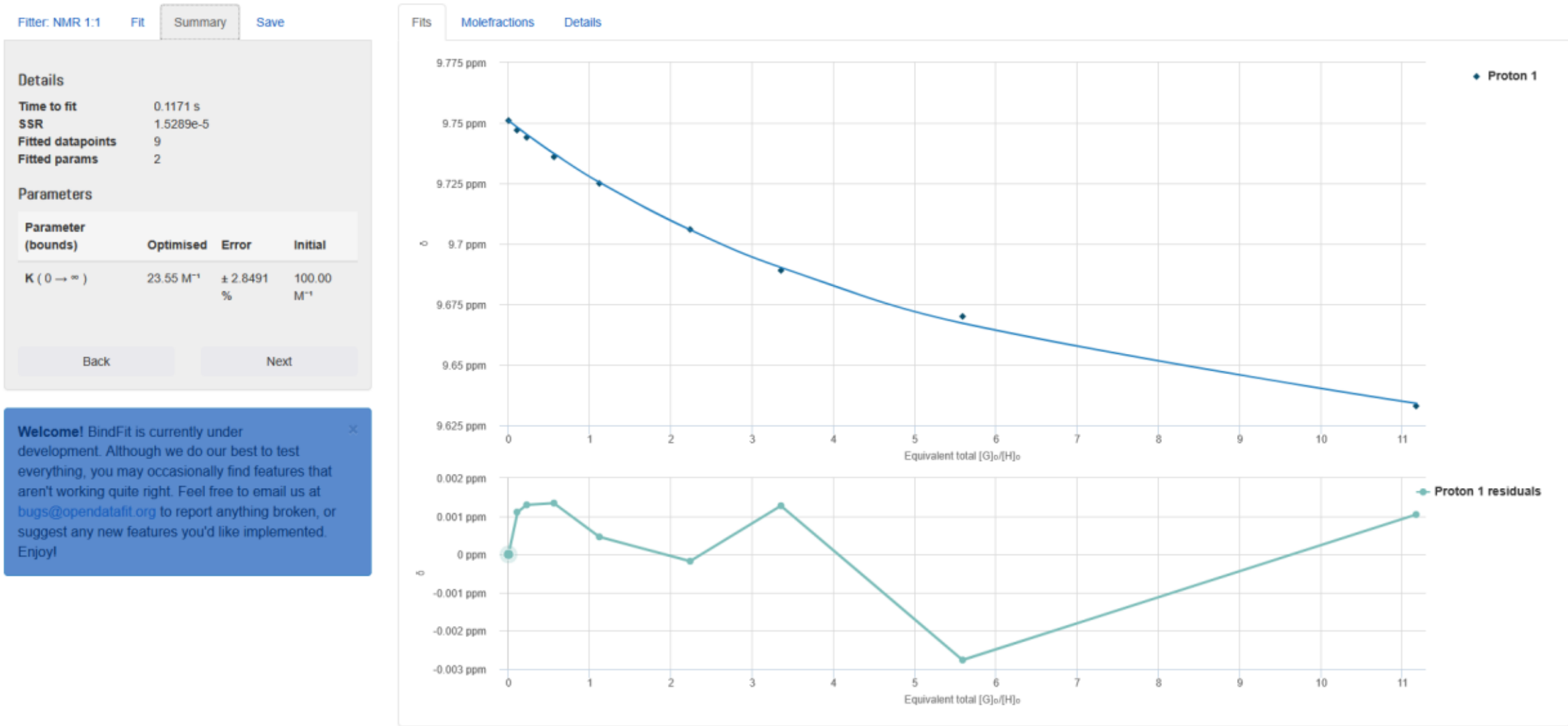

Figure S85. Association constant determination using BindFit[7-8] for ^{1}H NMR data in Figure S82 in equivalent total $[G]_0/[H]_0$ vs. ppm. For fitting, see http://app.supramolecular.org/bindfit/view/a0375879-c525-47e8-9be4-dcfed942d51d.

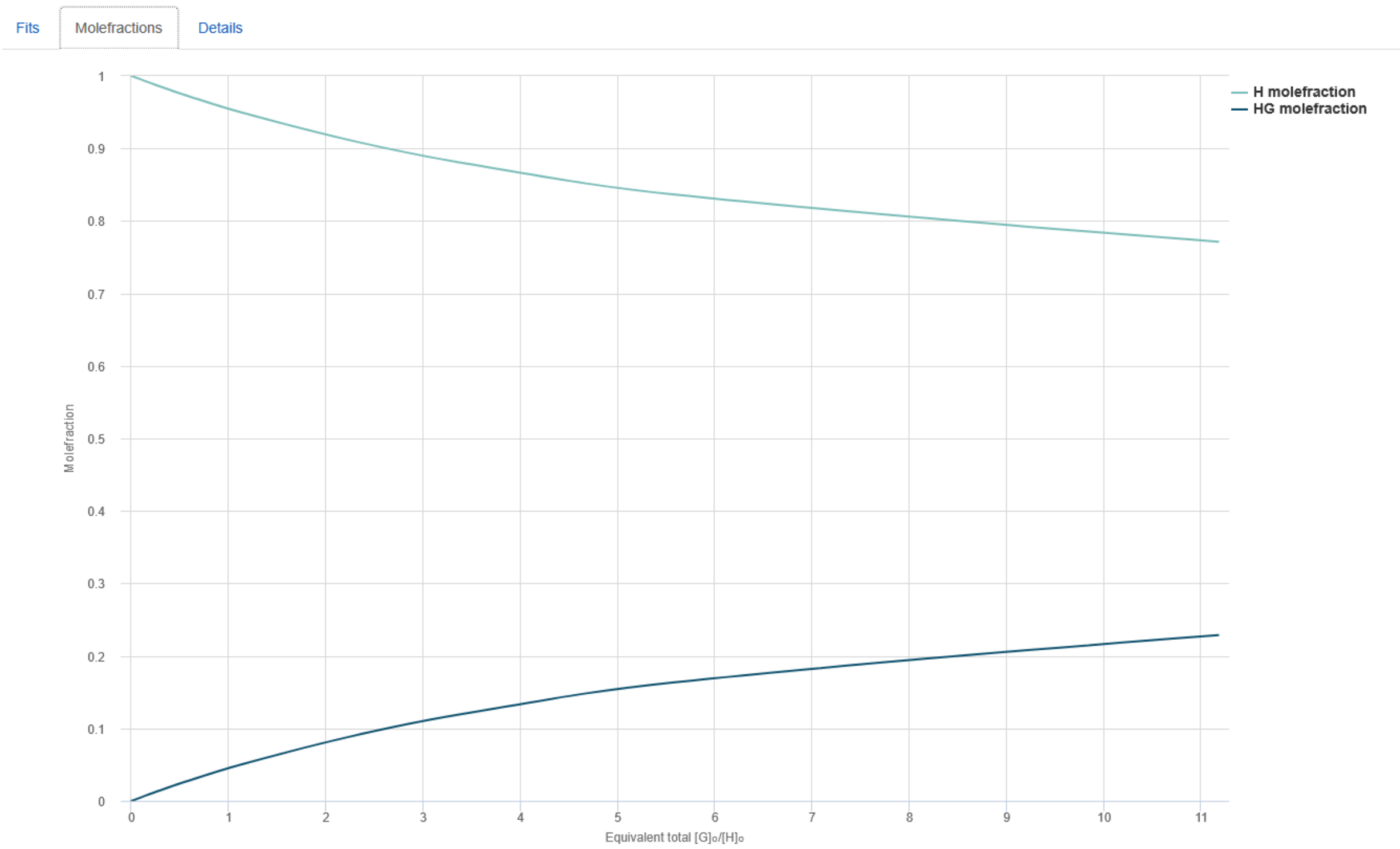

Figure S86. Association constant determination using BindFit[7-8] for NMR data in Figure Figure S82 in equivalent total $[G]_0/[H]_0$ vs. molefraction. For fitting, see http://app.supramolecular.org/bindfit/view/a0375879-c525-47e8-9be4-dcfed942d51d.

5.4 Photoswitching of homoleptic $[Pd_3(E\text{-}1)_6]^{6+}$ and $[Pd_4(E\text{-}1)_8]^{8+}$ species

5.4.1 Photoswitching of $[Pd_3(E\text{-}1)_6](BAr_F)_6$ in MeCN-d_3

The sample of $[Pd_3(E\text{-}\mathbf{1})_6](BAr_F)_6$ prepared in section 5.1 (Figure S87i and Figure S87i) was irradiated with 530 nm light for 10 minutes before ^1H and ^{19}F NMR spectra were recorded (Figure S87ii and Figure S87ii). This resulted in the quantitative disassembly of the double-walled $[Pd_3(E\text{-}\mathbf{1})_6](BAr_F)_6$ species and the formation of a new species with both, broad and sharp peaks present in the ^1H and ^{19}F NMR spectra. This suggests the formation of ill-defined or polymeric products which do not significantly resolve over the NMR timescale. There was no major product peak found in the ESI-MS, supporting the hypothesis that non-distinct products are formed once ligand **1** is in its *Z*-isomeric form.

By irradiating this mixture of ill-defined or polymeric products with 424 nm light for 10 minutes, the original $[Pd_3(E\text{-}\mathbf{1})_6](BAr_F)_6$ species could be restored (Figure S87iii and Figure S87iii).

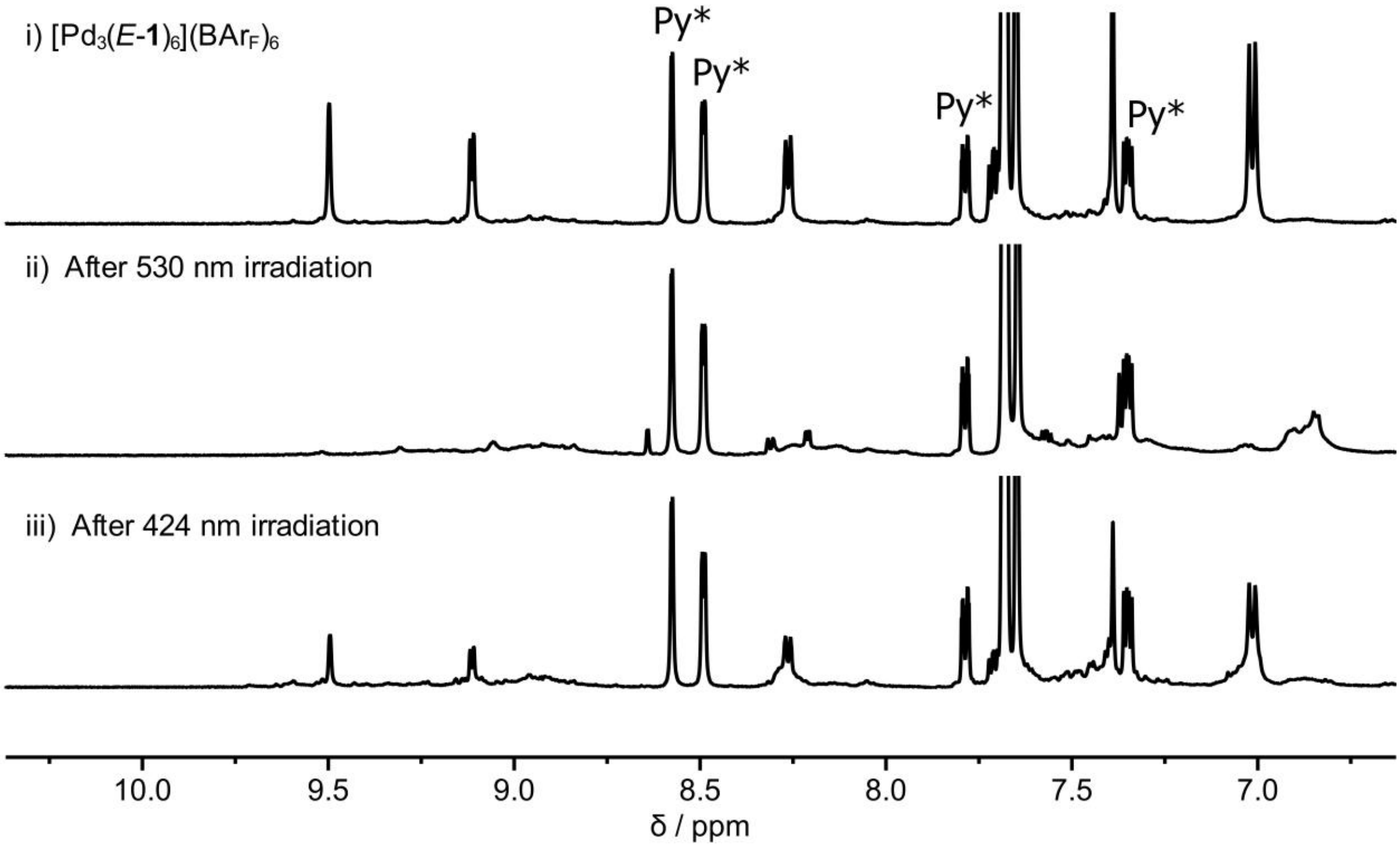

Figure S87. Partial ^1H NMR spectra (600 MHz, 298 K, MeCN-d_3) of i) $[Pd_3(E\text{-}\mathbf{1})_6](BAr_F)_6$, ii) the previous sample after irradiation with 530 nm light for 10 minutes, and iii) the previous sample after irradiation with 424 nm light for 10 minutes.

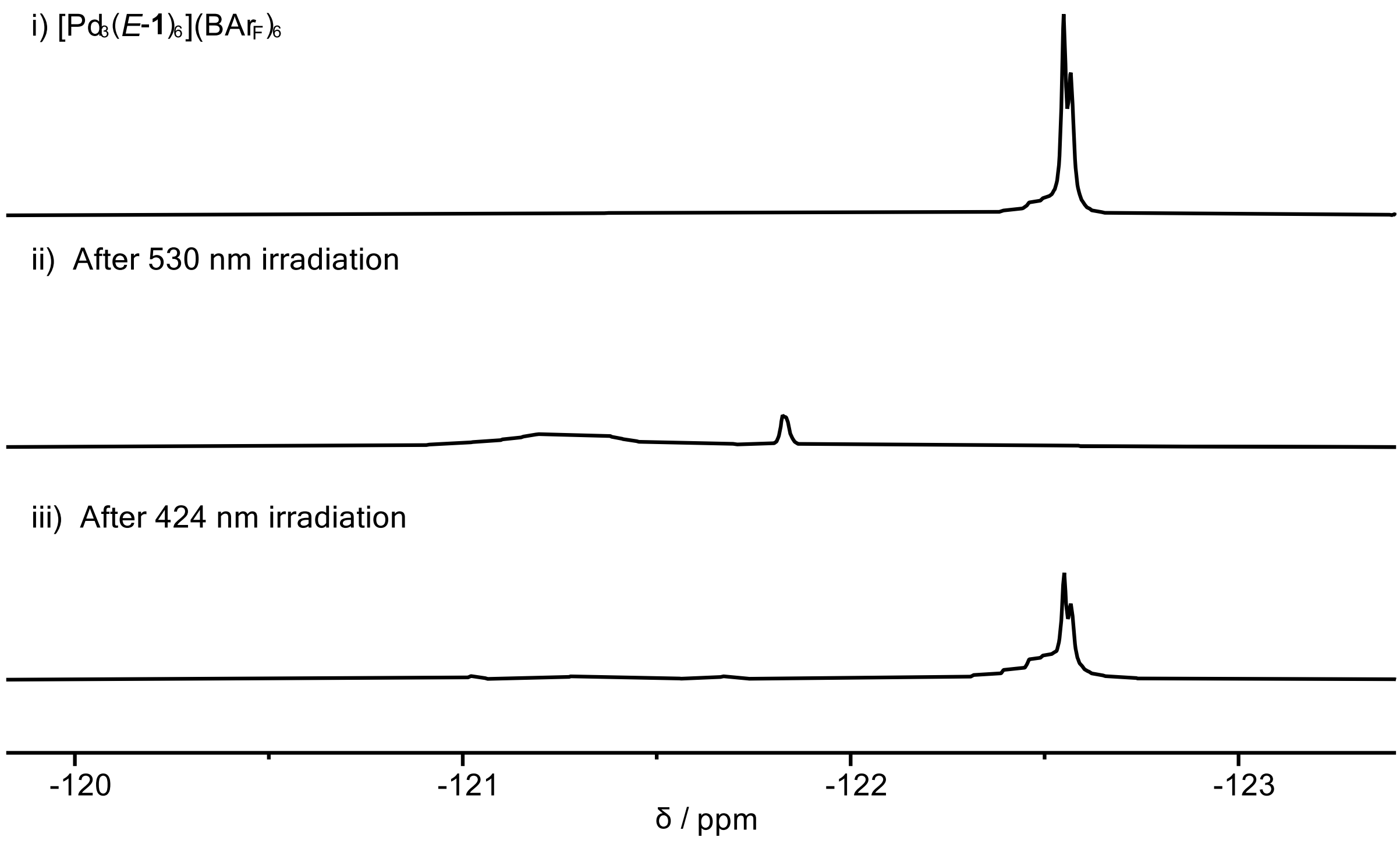

Figure S88. ^{19}F NMR spectra (565 MHz, 298 K, MeCN-d_3) of i) [Pd$_3$(*E*-**1**)$_6$](BAr$_F$)$_6$, ii) the previous sample after irradiation with 530 nm light for 10 minutes, and iii) the previous sample after irradiation with 424 nm light for 10 minutes.

5.4.2 Photoswitching of [Pd$_3$(*E*-1)$_6$](BF$_4$)$_6$ in DMSO-d_6

The sample of [Pd$_3$(*E*-**1**)$_6$](BF$_4$)$_6$ prepared in section 5.1.3 (Figure S89i and Figure S90i) was irradiated with 530 nm light for 10 minutes before ^{1}H and ^{19}F NMR spectra were recorded (Figure S89ii and Figure S90ii). This resulted in the almost quantitative disassembly of the double-walled [Pd$_3$(*E*-**1**)$_6$](BF$_4$)$_6$ species and the formation of a new species with broad peaks in the ^{1}H and ^{19}F NMR spectra. This suggests the formation of ill-defined or polymeric products which do not significantly resolve over the NMR timescale. There was no major product peak found in the ESI-MS (Figure S91), supporting the hypothesis that non-distinct products are formed once ligand **1** is in its *Z*-isomeric form.

By irradiating this mixture of ill-defined or polymeric products with 424 nm light for 10 minutes, the original [Pd$_3$(*E*-**1**)$_6$](BF$_4$)$_6$ species could be restored (Figure S89iii and Figure S90iii).

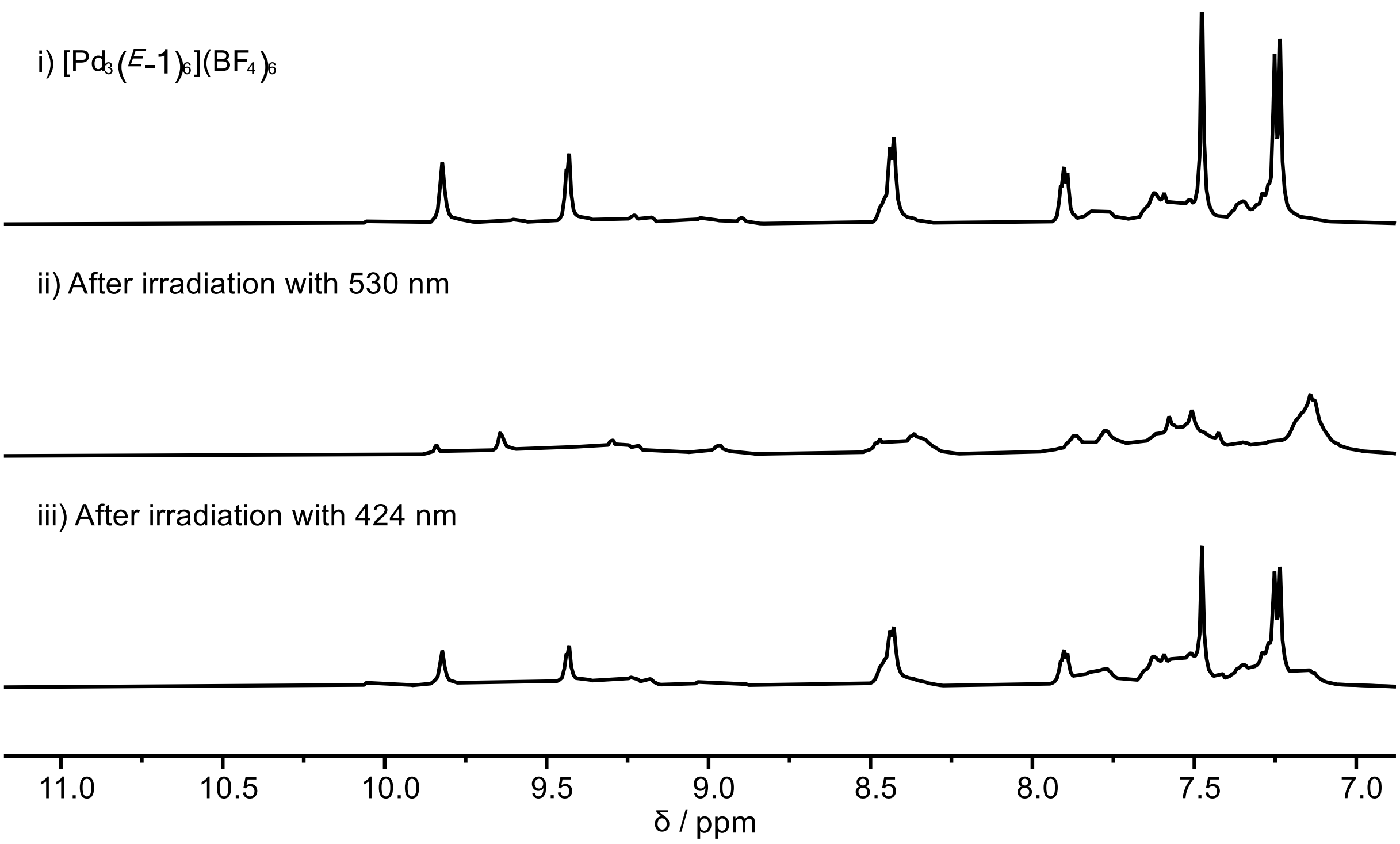

Figure S89. Partial ^{1}H NMR spectra (600 MHz, 298 K, DMSO-d_6) of i) $[Pd_3(E\text{-}\mathbf{1})_6](BF_4)_6$, ii) the previous sample after irradiation with 530 nm light for 10 minutes, and iii) the previous sample after irradiation with 424 nm light for 10 minutes.

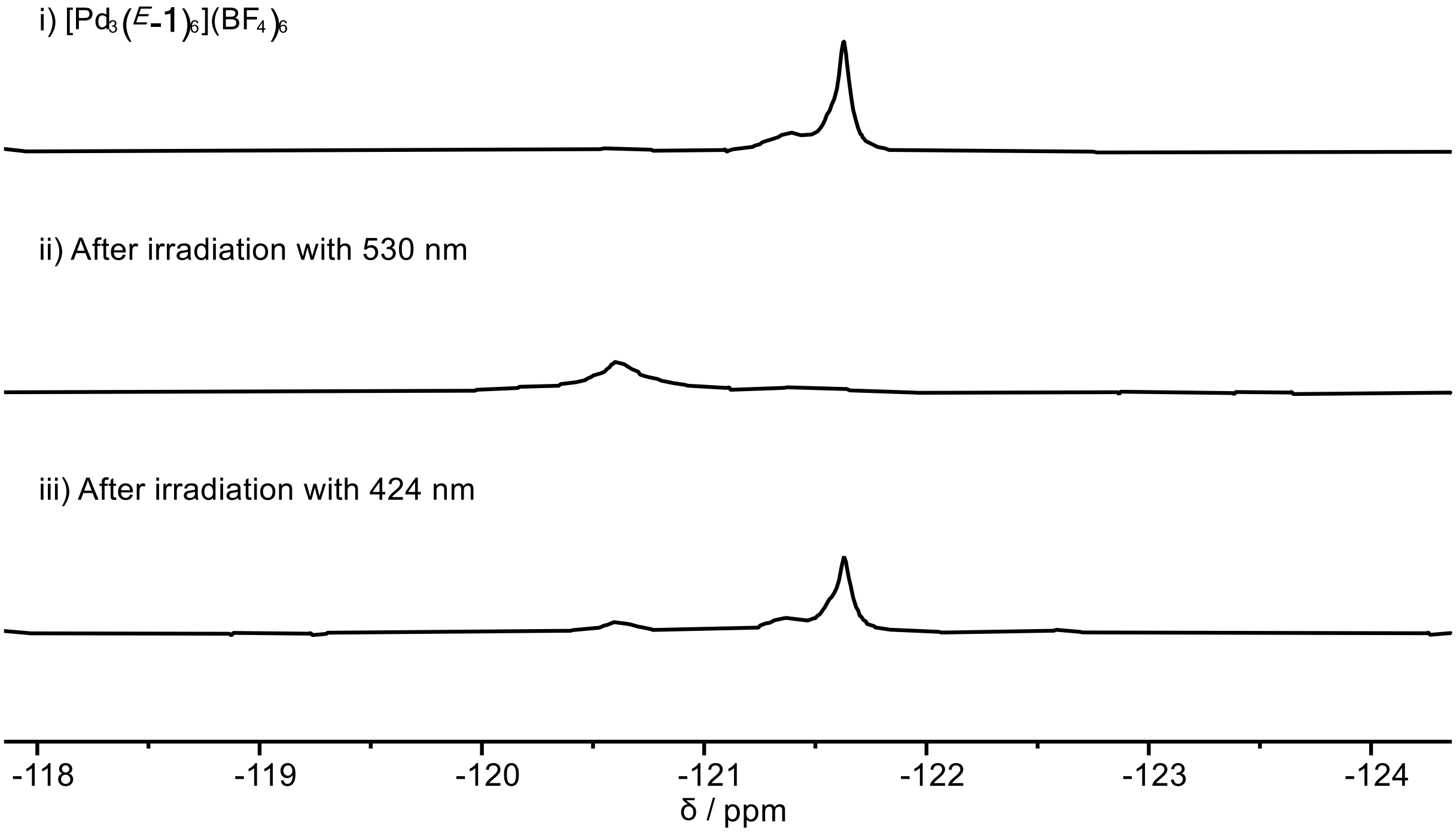

Figure S90. ^{19}F NMR spectra (565 MHz, 298 K, DMSO-d_6) of i) $[Pd_3(E\text{-}\mathbf{1})_6](BF_4)_6$, ii) the previous sample after irradiation with 530 nm light for 10 minutes, and iii) the previous sample after irradiation with 424 nm light for 10 minutes.

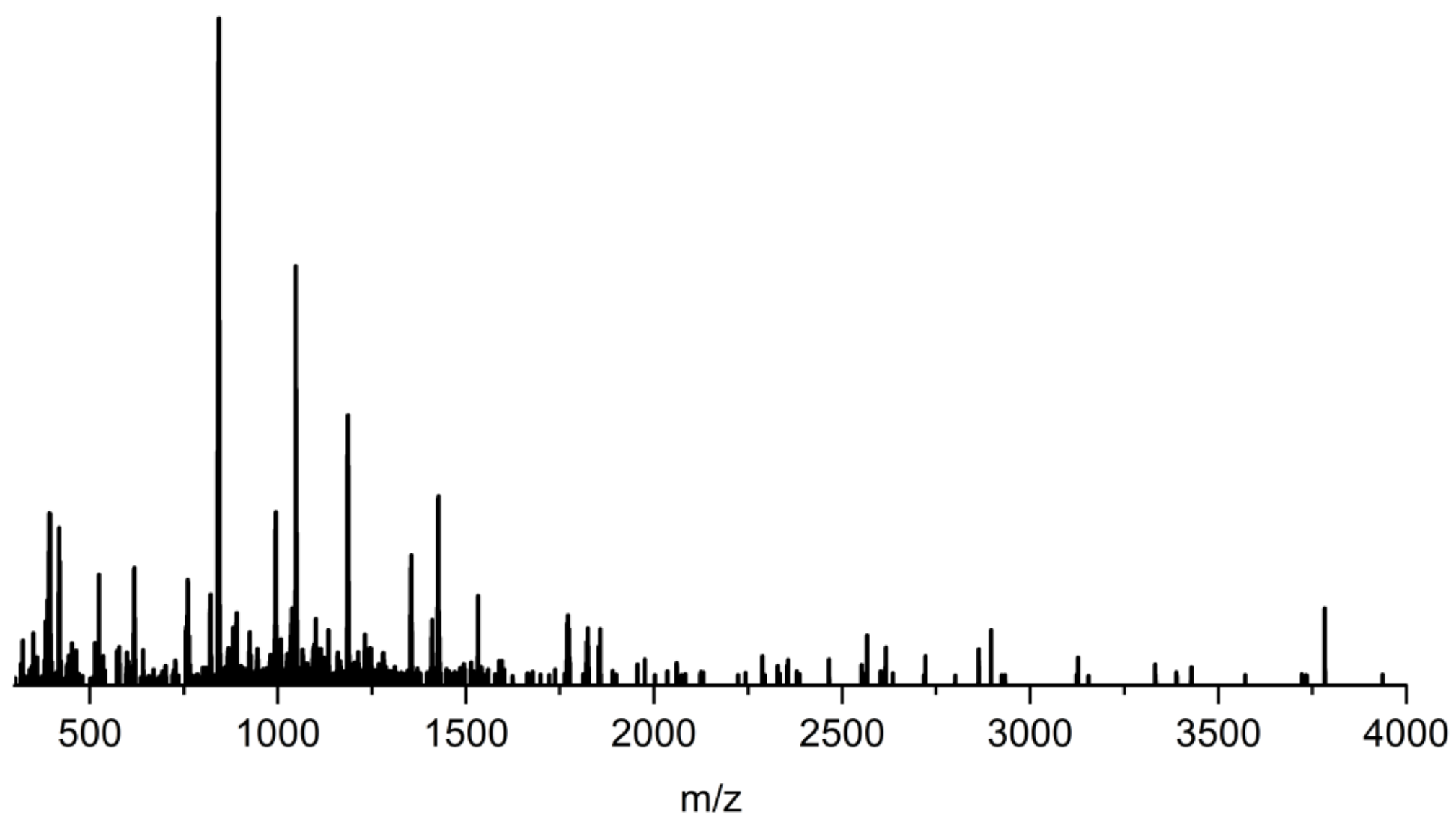

Figure S91. ESI-MS spectrum of $[Pd_3(E\text{-}\mathbf{1})_6](BF_4)_6$ ([Pd] = 2.0 mM) in DMSO-d_2 after irradiation with 530 nm light for 10 minutes.

5.4.3 Photoswitching of the mixture of [Pd_3(*E*-1)$_6$](BF_4)$_6$ and [Pd_4(*E*-1)$_8$](BF_4)$_8$ in MeCN-*d*$_3$

The sample mixture of [Pd_3(*E*-**1**)$_6$](BF_4)$_6$ and [Pd_4(*E*-**1**)$_8$](BF_4)$_8$ prepared in section 5.2 (Figure S92i and Figure S93i) was irradiated with 530 nm light for 10 minutes before ^{1}H and ^{19}F NMR spectra were recorded (Figure S92ii and Figure S93ii). This resulted in the quantitative disassembly of the [Pd_3(*E*-**1**)$_6$](BF_4)$_6$ and [Pd_4(*E*-**1**)$_8$](BF_4)$_8$ species and the formation of a new species with very broad peaks in the ^{1}H and ^{19}F NMR spectra. This suggests the formation of ill-defined, polymeric or large products which do not significantly resolve over the NMR timescale.

By irradiating this mixture of ill-defined or polymeric products with 424 nm light for total 40 minutes, the original [Pd_3(*E*-**1**)$_6$](BF_4)$_6$ and [Pd_4(*E*-**1**)$_8$](BF_4)$_8$ mixture could not be restored, suggesting that the formed *Z*-isomeric products are kinetically trapped (Figure S92iii and Figure S93iii). Even after using longer irradiation times (total four hours) and heating the sample to 50 degrees overnight, the system could not be driven back towards the initial [Pd_3(*E*-**1**)$_6$](BF_4)$_6$ and [Pd_4(*E*-**1**)$_8$](BF_4)$_8$ mixture.

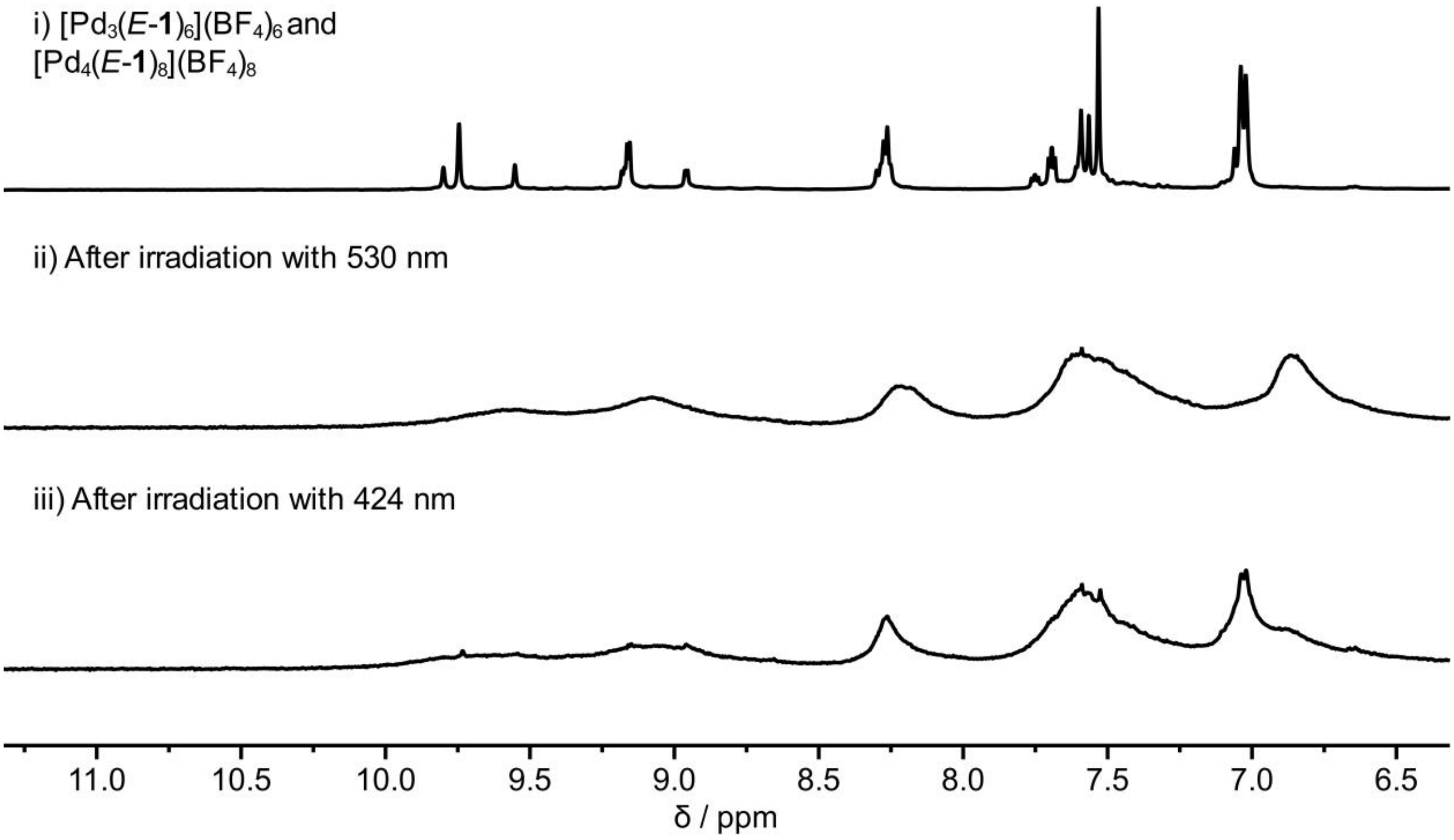

Figure S92. Partial ^{1}H NMR spectra (600 MHz, 298 K, MeCN-*d*$_{a3}$) of i) a mixture of [Pd_3(*E*-**1**)$_6$](BF_4)$_6$ and [Pd_4(*E*-**1**)$_8$](BF_4)$_8$, ii) the previous sample after irradiation with 530 nm light for 10 minutes, and iii) the previous sample after irradiation with 424 nm light for 40 minutes.

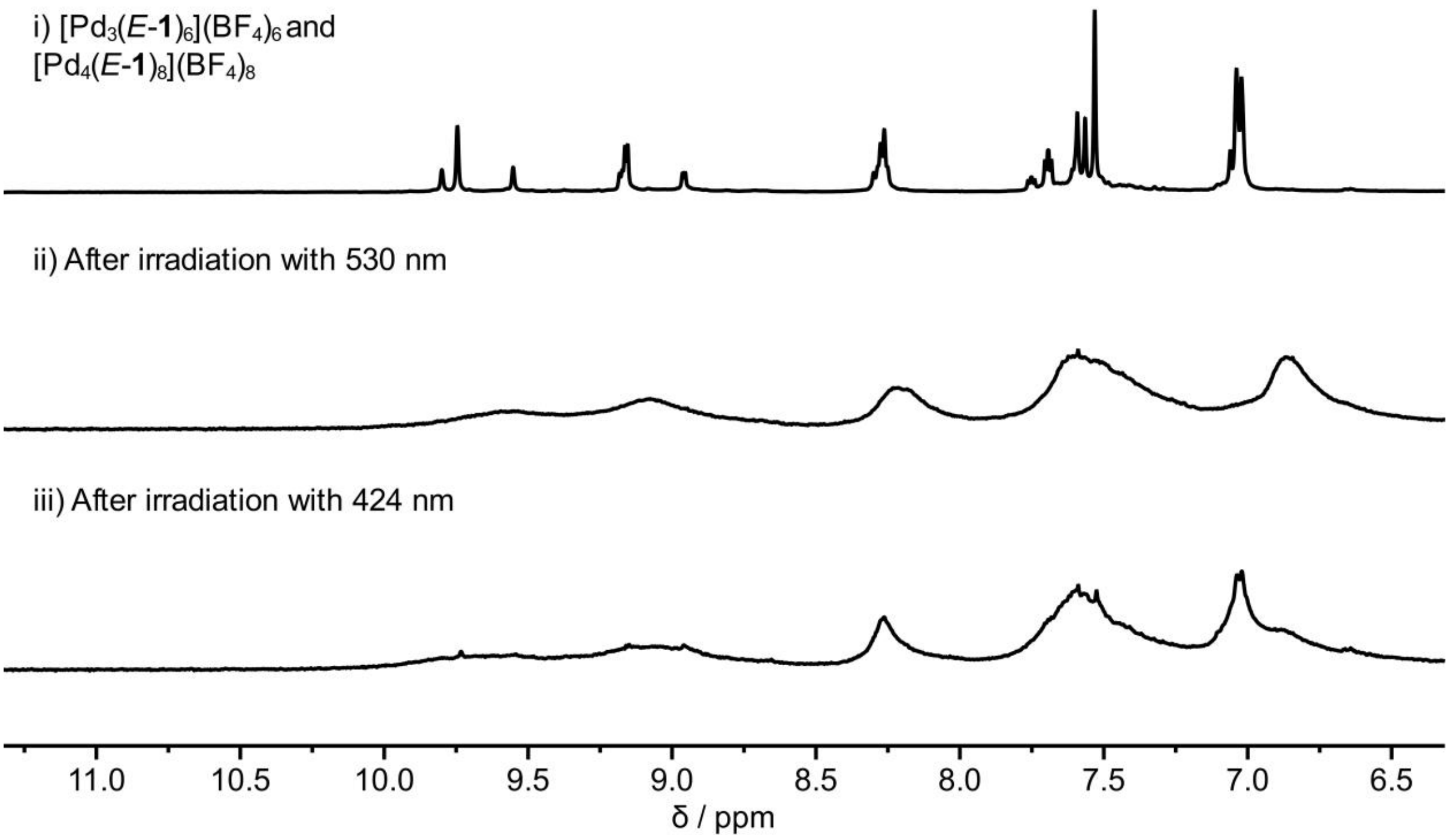

Figure S93. ^{19}F NMR spectra (565 MHz, 298 K, MeCN-d_{a3}) of i) a mixture of $[Pd_3(E\text{-}\mathbf{1})_6](BF_4)_6$ and $[Pd_4(E\text{-}\mathbf{1})_8](BF_4)_8$, ii) the previous sample after irradiation with 530 nm light for 10 minutes, and iii) the previous sample after irradiation with 424 nm light for 40 minutes.

6 Synthesis and characterization of heteroleptic metastable $[Pd_2(Z\text{-}\mathbf{1})_2(\mathbf{2})_2](BAr_F)_4$ in MeCN-d_3

6.1 Synthesis of heteroleptic metastable $[Pd_2(Z\text{-}\mathbf{1})_2(\mathbf{2})_2](BAr_F)_4$ in MeCN-d_3

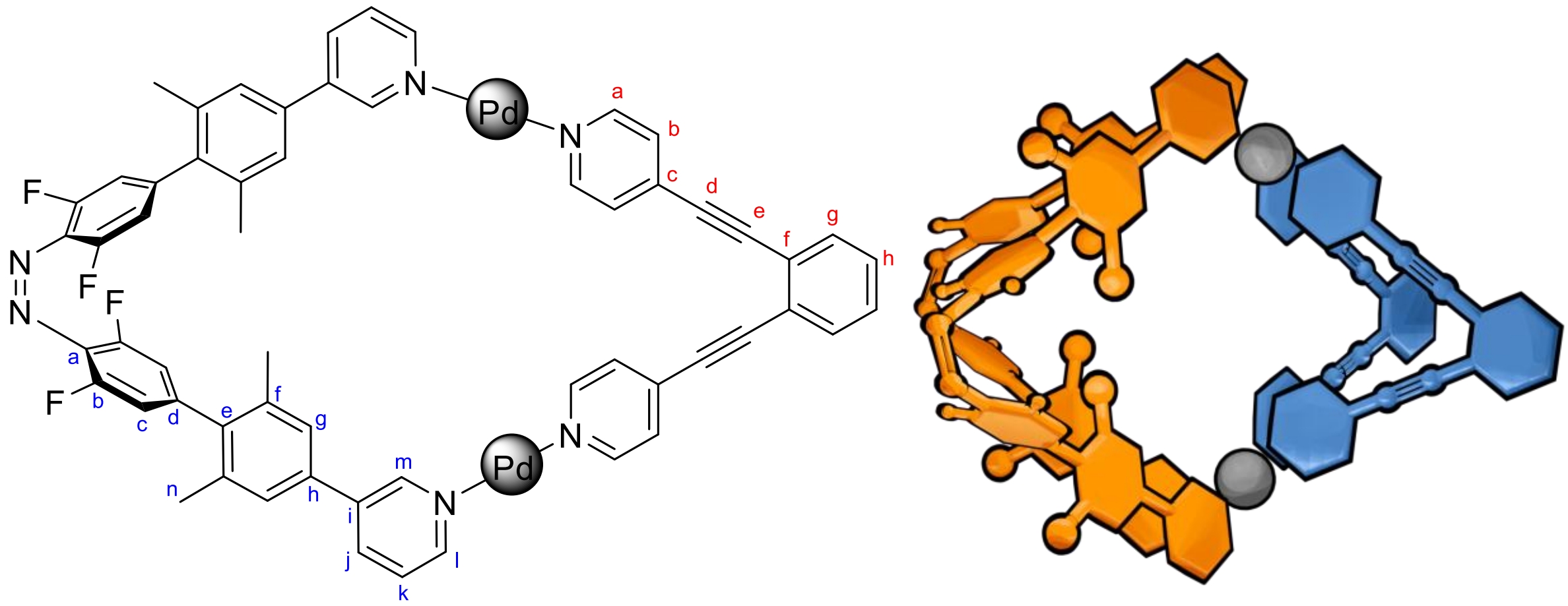

The ability of ligand *Z*-**1** to form a metastable heteroleptic cage with ligand **2** was investigated using ^{1}H and ^{19}F NMR spectroscopy as well as ESI-MS.

To ensure only *E*-**1** was present, a solution of the photoswitchable ligand **1** (250 µL, 4.0 mM, 2.0 equiv., in MeCN-d_3) was heated in a sealed microwave tube at 120 °C for 3 hours (monitored by ^{1}H NMR spectroscopy, Figure S94i). The ligand *E*-**1** solution was then mixed with a solution of ligand **2** (250 µL, 4.0 mM, 2.0 equiv., in MeCN-d_3) to form a 1:1 mixture of the ligands *E*-**1** and **2** (Figure S94iii, final ligand concentration = 2.0 mM each). $[Pd(Py^*)_4](BArF)_2$ stock solution (40 µL, 29.7 mM, 1.0 equiv.) was added to the 1:1 mixture of the ligands. The sample was mixed well using a Vortex mixer and was then left in the sand bath (50 °C) to equilibrate for 2 hours. ^{1}H and ^{19}F NMR revealed a mixture of different self-assembled species and with the help of ESI-MS we could identify the homoleptic $[Pd_3(E\text{-}\mathbf{1})_6](BArF)_6$, $[Pd_3(\mathbf{2})_6](BArF)_6$ and heteroleptic $[Pd_3(E\text{-}\mathbf{1})_4(\mathbf{2})_2](BArF)_6$ and $[Pd_4(E\text{-}\mathbf{1})_4(\mathbf{2})_4](BArF)_8$ cages (Figure S94iv). Once this cage mixture is irradiated with 530 nm light for 30 minutes, a single, highly symmetric species is found in the ^{1}H and ^{19}F NMR (Figure S94v). Together with ESI-MS data this species could be identified as the heteroleptic metastable $[Pd_2(Z\text{-}\mathbf{1})_2(\mathbf{2})_2](BArF)_4$ cage. We propose that the structure of this $[Pd_2(Z\text{-}\mathbf{1})_2(\mathbf{2})_2](BArF)_4$ cage is lantern-shaped, consistent with a previously reported structure formed from a similar ligand combination.[2]

Comparing the ^{1}H NMR spectrum of $[Pd_2(Z\text{-}\mathbf{1})_2(\mathbf{2})_2](BArF)_4$ to the ^{1}H NMR spectra of free ligands *E*-**1**, *Z*-**1** and **2** and homoleptic $[Pd_3(E\text{-}\mathbf{1})_6](BArF)_6$ and $[Pd_3(\mathbf{2})_6](BArF)_6$ cages confirms the quantitative assembly of a single heteroleptic species. A cross peak between H^m and H^a in the ^{1}H-^{1}H ROESY NMR confirmed proximity between the two ligands, consistent with the formation of a heteroleptic cage.

Heteroleptic $[Pd_2(Z\text{-}\mathbf{1})_2(\mathbf{2})_2](BArF)_4$ can also be assembled by mixing equivual amounts of homoleptic $[Pd_3(E\text{-}\mathbf{1})_6](BArF)_6$ ([Pd] = 2.0 mM, 250 µL, 1.0 equivuiv., in MeCN-d_3) and $[Pd_3(\mathbf{2})_6](BArF)_6$ ([Pd] = 2.0 mM, 250 µL, 1.0 equiv., in MeCN-d_3) followed by irradiation with 530 nm light for 10 minutes.

Blue assignments correspond to signals arising from ligand *Z*-**1** and red assignments to ligand **2**.

^{1}H NMR (600 MHz, MeCN-d_3) δ 9.46 (d, *J* = 2.1 Hz, 4H, H^{m}), 9.06 (dd, *J* = 5.7, 1.3 Hz, 4H, $H^{l)}$, 8.96 (dd, *J* = 6.7, 1.4 Hz, 8H, H^{a}), 8.28 (ddd, *J* = 8.1, 2.1, 1.3 Hz, 4H, H^{j}), 7.76 (dd, *J* = 6.7, 1.4 Hz, 8H, H^{b}), 7.74 – 7.69 (m, 8H, H^{k+g}), 7.56 (dd, *J* = 6.0, 3.2 Hz, 4H, H^{h}), 7.46 (s, 8H, H^{g}), 6.92 (d, *J* = 8.7 Hz, 8H, H^{c}), 2.02 (br s, 24H, H^{n}).

^{19}F NMR (565 MHz, MeCN-d_3) δ -121.58 (d, *J* = 230.0 Hz, F^{b}).

^{13}C NMR (151 MHz, MeCN-d_3) δ 153.6 (d, *J* = 247.1 Hz, C^{b}), 151.8 (C^{a}), 150.1 (C^{l}), 150.0 (C^{m}), 140.5 (C^{h}), 139.3 (C^{j}), 136.3 (C^{c}), 134.3 (C^{f}), 131.9 (C^{h}), 130.3 (C^{b}), 127.1 (br. C^{g}), 124.6 (C^{g}), 114.0 (d, *J* = 18.6 Hz, C^{c}), 97.6 (C^{e}), 89.6 (C^{d}), 20.7 (br. C^{n}).

Quaternary C^{a}, C^{d}, C^{e}, C^{f}, C^{i} of the ligand *Z*-**1** could not unambiguously be assigned due to weak carbon signals and no observable cross peak in the HMBC. C^{k} could not be assigned due to overlap with the broad BAr_F peak in the HSQC. Broad carbon peaks were observed for C^{g} and C^{n}, presumably due to slow exchange.

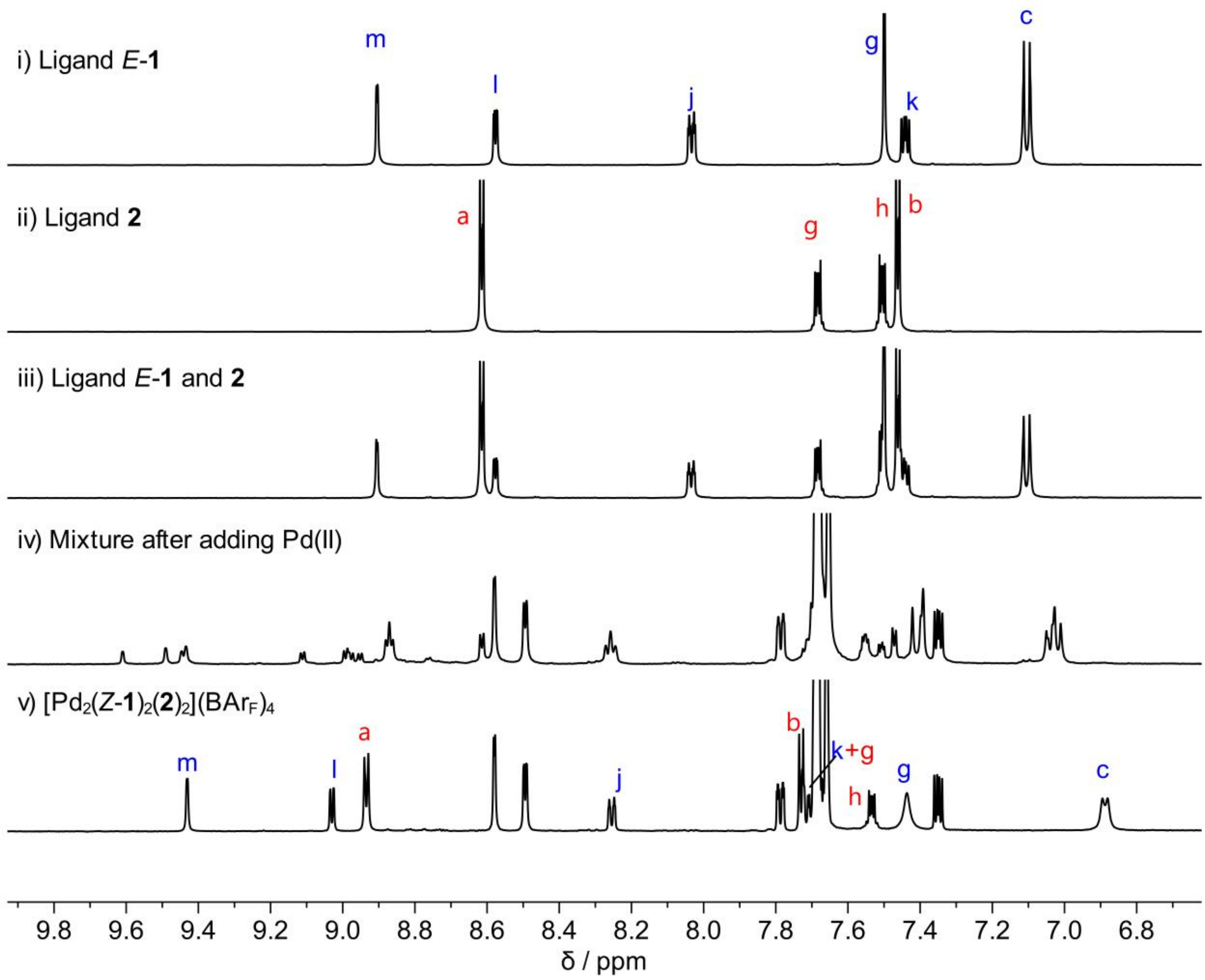

Figure S94. ^{1}H NMR (600 MHz, 298 K, MeCN-d_3) spectrum of i) ligand *E*-**1**, ii) ligand **2**, iii) a 1:1 mixture of ligand *E*-**1** and ligand **2**, iv) the mixture after the addition of $[Pd(Py^*)_4](BArF)_2$, and v) after irradiation of the previous sample with 530 nm light affording $[Pd_2(Z\text{-}\mathbf{1})_2(\mathbf{2})_2](BAr_F)_4$ and free Py*.

6.2 1D and 2D NMR characterization of [Pd_2(*Z*-1)$_2$(2)$_2$](BAr$_F$)$_4$

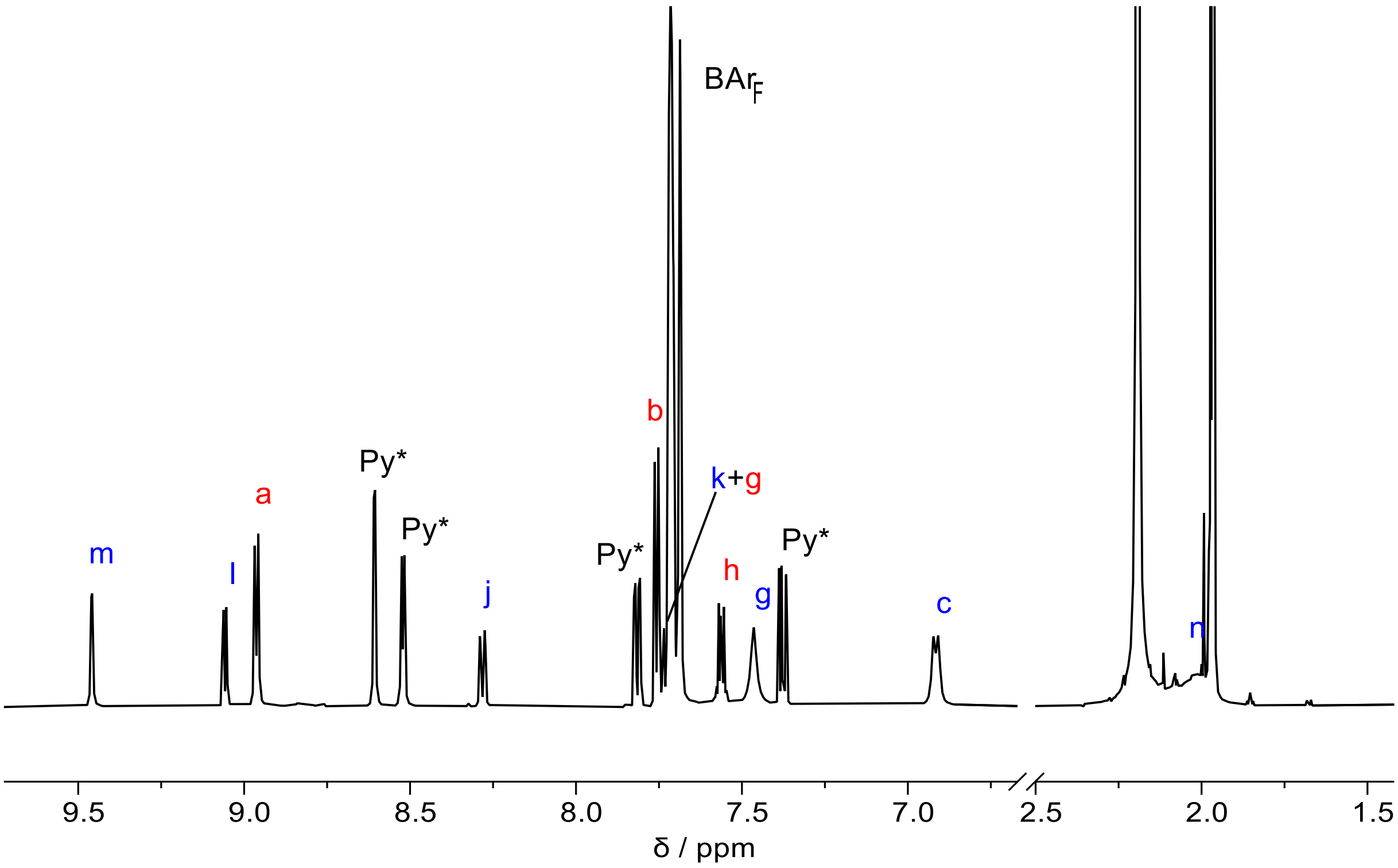

Figure S95. ^{1}H NMR (600 MHz, 298 K, MeCN-d_3) spectrum of [Pd_2(*Z*-**1**)$_2$(**2**)$_2$](BAr$_F$)$_4$ and free Py*.

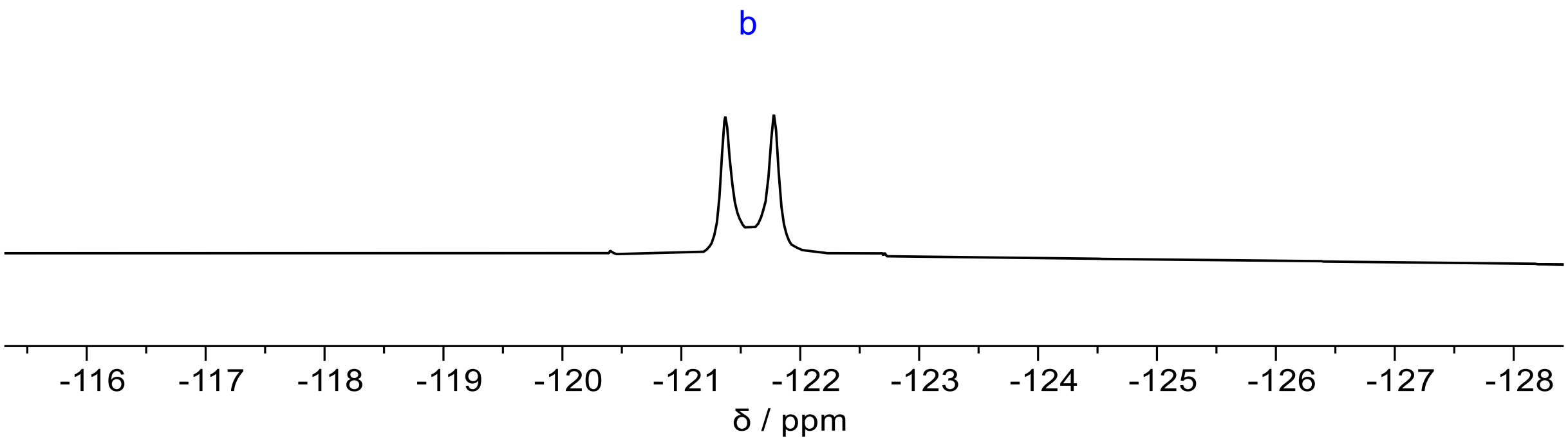

Figure S96. ^{19}F NMR (565 MHz, 298 K, MeCN-d_3) spectrum of [Pd_2(*Z*-**1**)$_2$(**2**)$_2$](BAr$_F$)$_4$ and free Py*.

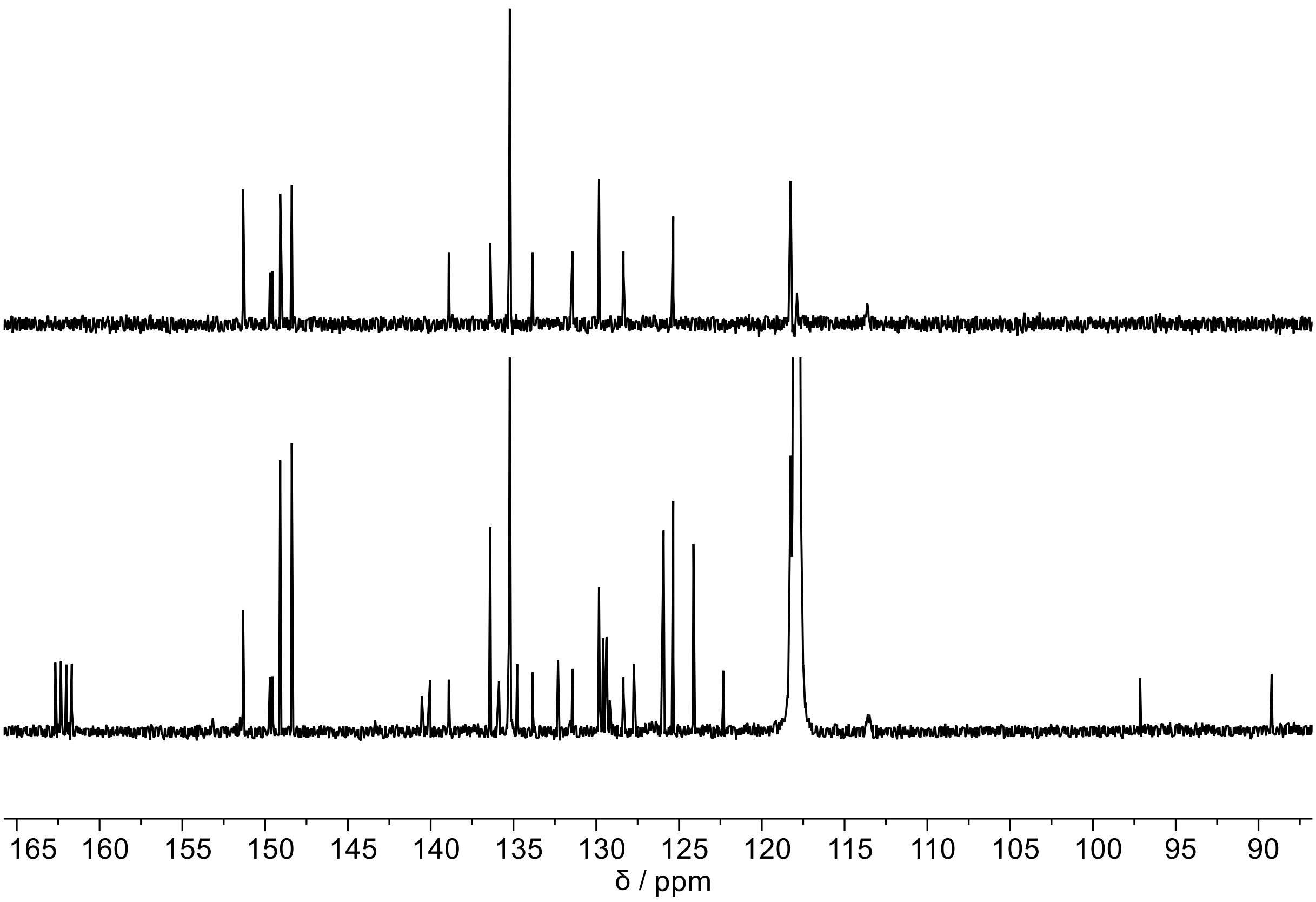

Figure S97 $^{13}C\{^1H\}$ and 135-DEPT NMR (151 MHz, 298 K, MeCN-*d*$_3$) spectrum of [Pd$_2$(*Z*-**1**)$_2$(**2**)$_2$](BAr$_F$)$_4$ and free Py*.

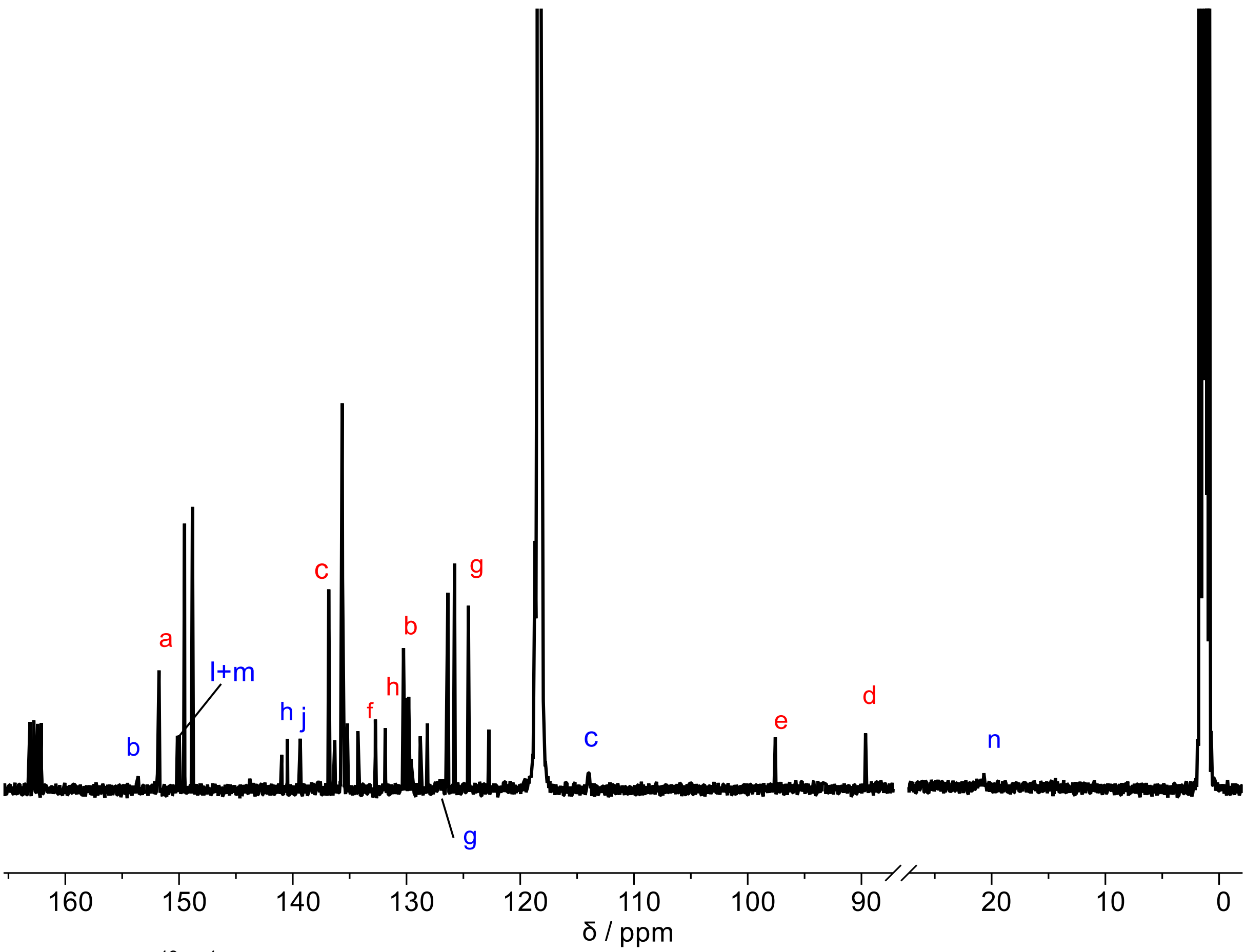

Figure S98. $^{13}C\{^1H\}$ NMR (151 MHz, 298 K, MeCN-*d*$_3$) spectrum of [Pd$_2$(*Z*-**1**)$_2$(**2**)$_2$](BAr$_F$)$_4$ and free Py*. Unassigned peaks belong to BAr$_F$ and free Py*.

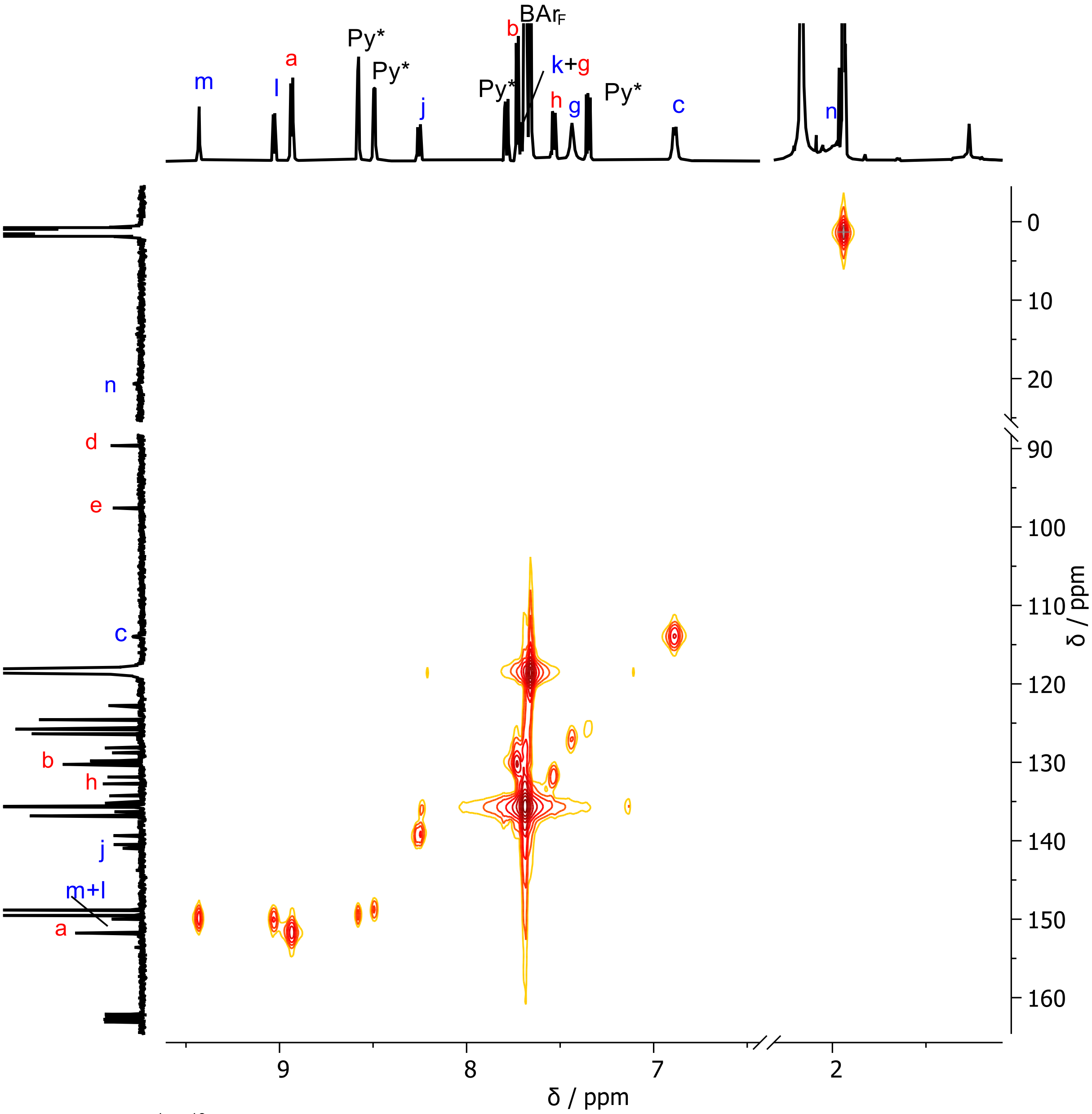

Figure S99. ^{1}H-^{13}C HSQC NMR (600 MHz, 151 MHz, 298 K, MeCN-d_3) spectrum of $[Pd_2(Z\text{-}\mathbf{1})_2(\mathbf{2})_2](BAr_F)_4$ and free Py*.

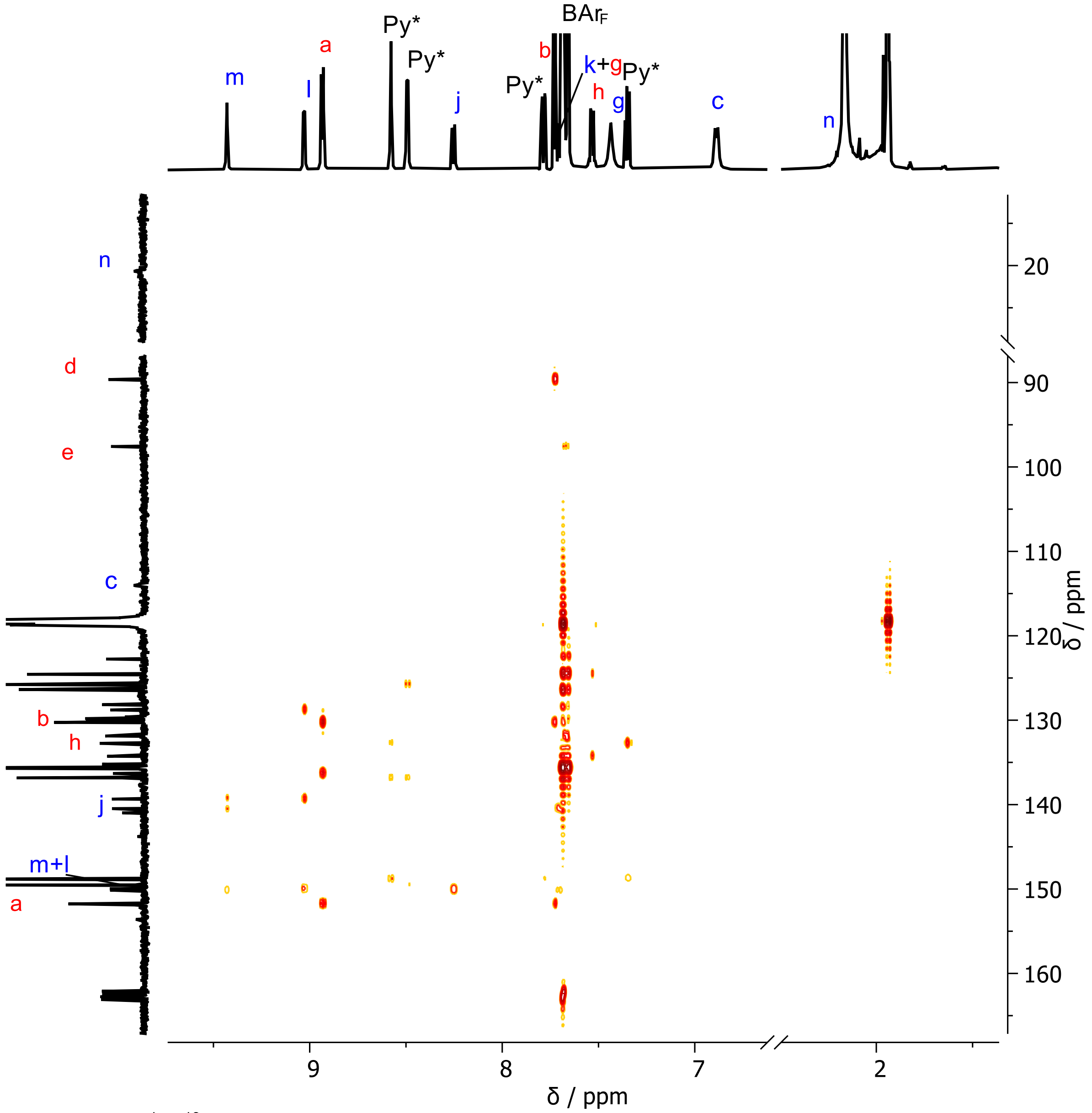

Figure S100. ^{1}H-^{13}C HMBC NMR (600 MHz, 151 MHz, 298 K, MeCN-d_3) spectrum of of [Pd_2(Z-**1**)$_2$(**2**)$_2$](BAr_F)$_4$ and free Py*.

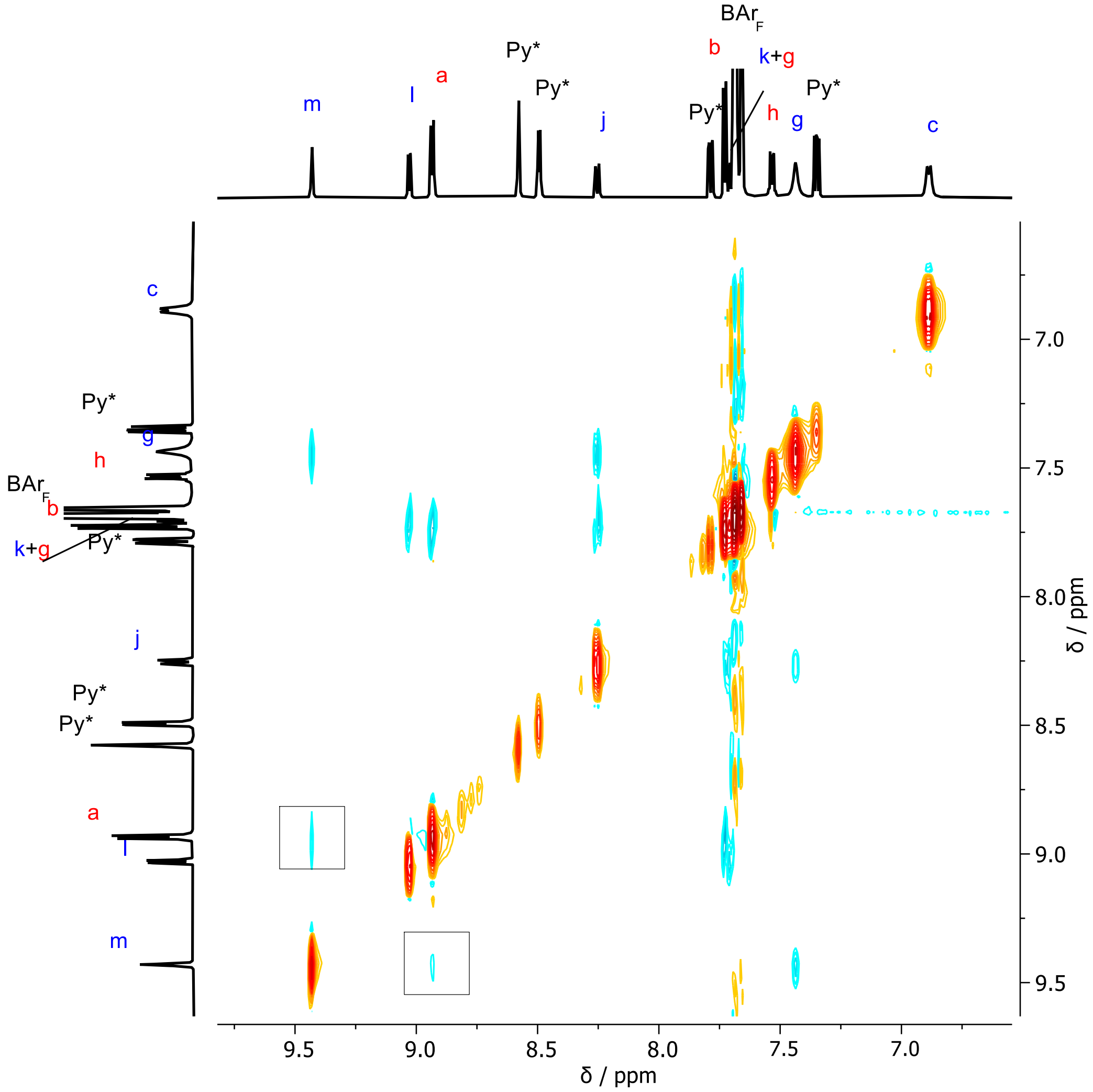

Figure S101. ^1H-^1H ROESY NMR (600 MHz, 298 K, MeCN-d_3) spectrum of $[Pd_2(Z\text{-}\mathbf{1})_2(\mathbf{2})_2](BAr_F)_4$ and free Py* showing the through space coupling between H^m and H^a.

6.3 ESI-MS of $[Pd_2(Z\text{-}\mathbf{1})_2(\mathbf{2})_2](BAr_F)_4$ in MeCN-d_3

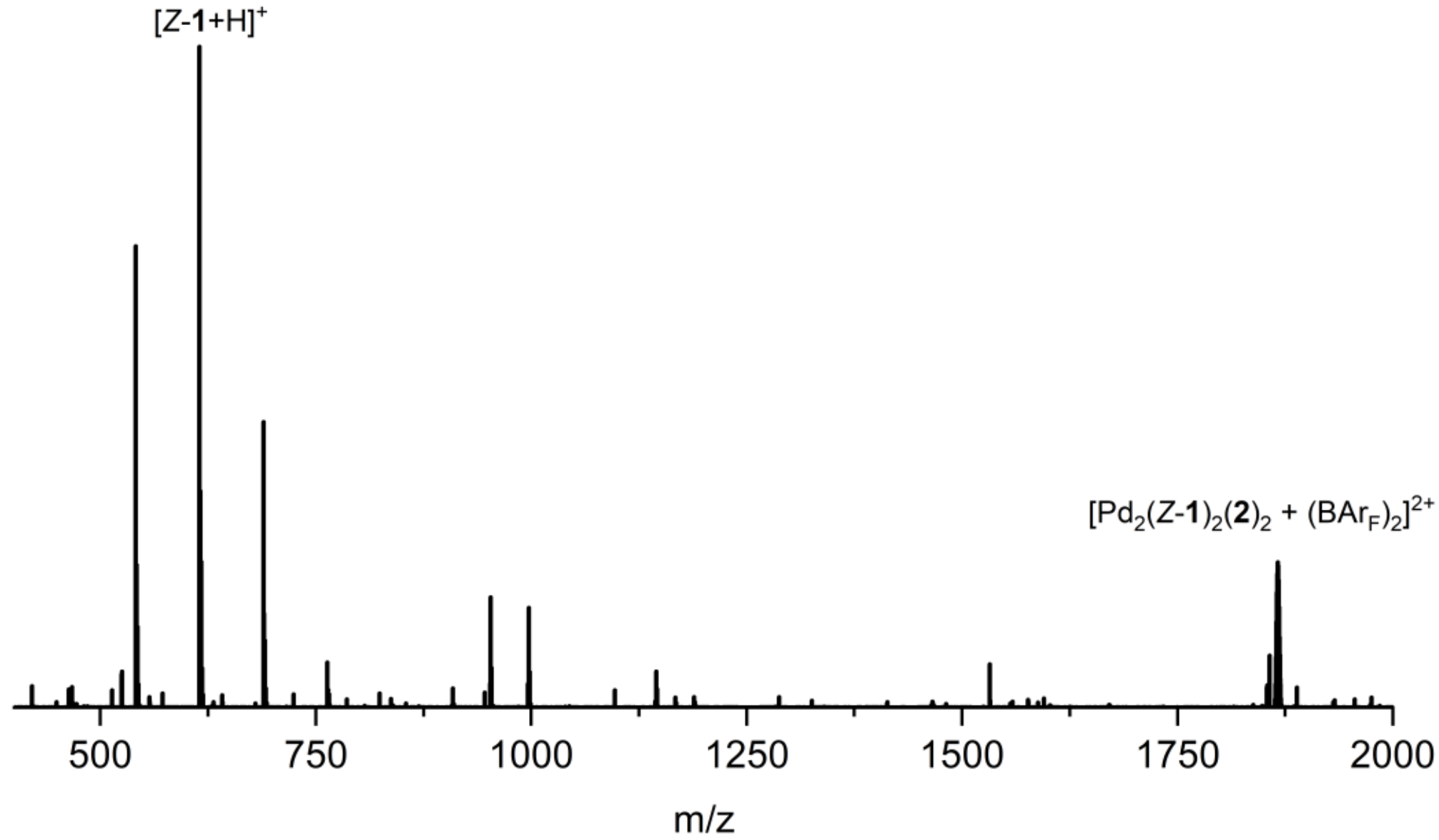

Figure S102. ESI-MS spectrum of $[Pd_2(Z\text{-}\mathbf{1})_2(\mathbf{2})_2](BAr_F)_4$ ([Pd] = 2.0 mM) in MeCN-d_3.

Table S 25. Zoom scans of select ESI-MS peaks, with simulated isotope patterns of $[Pd_2(Z\text{-}\mathbf{1})_2(\mathbf{2})_2](BAr_F)_4$ ([Pd] = 2.0 mM) in MeCN-d_3.

$[Pd_2(Z\text{-}\mathbf{1})_2(\mathbf{2})_2 + (BAr_F)_2]^{2+}$
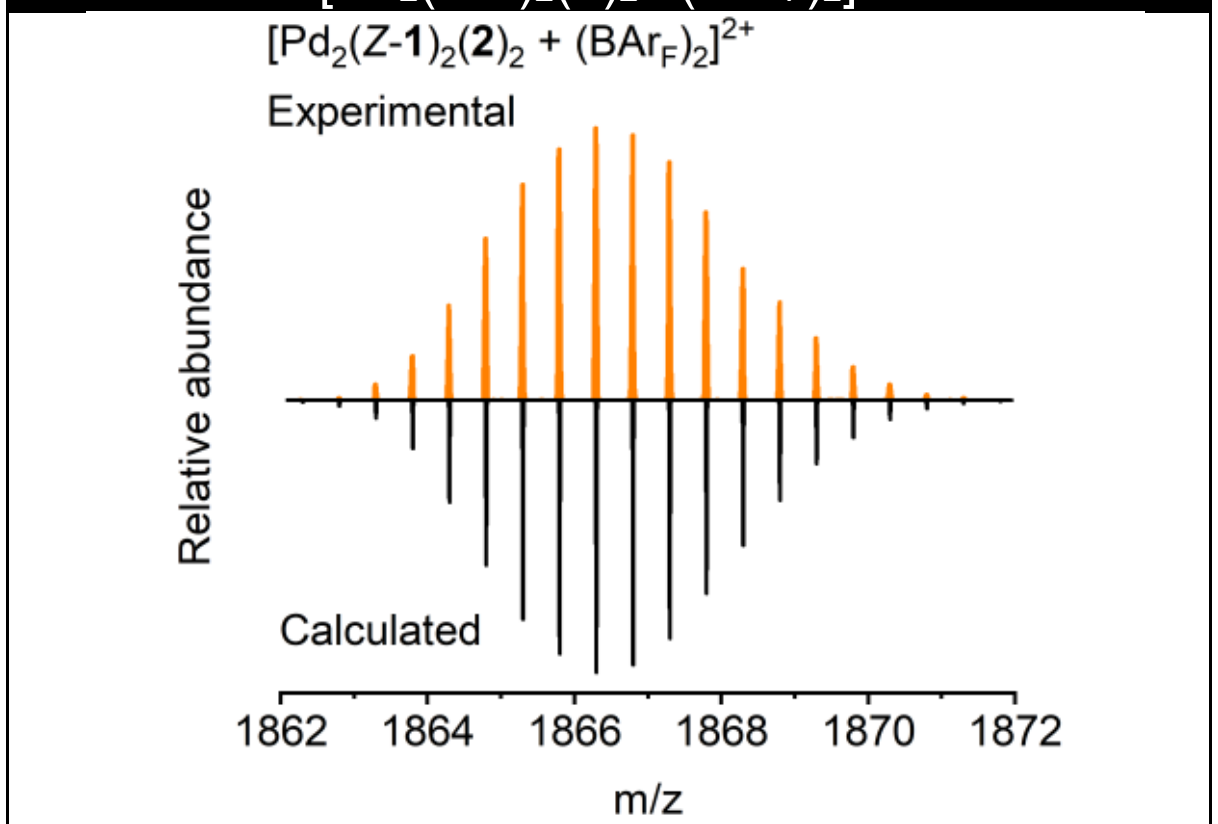

6.4 ESI-MS of the mixture of $[Pd_3(E\text{-}\mathbf{1})_4(\mathbf{2})_2](BAr_F)_6$ and $[Pd_4(E\text{-}\mathbf{1})_4(\mathbf{2})_4](BAr_F)_8$ in MeCN-d_3

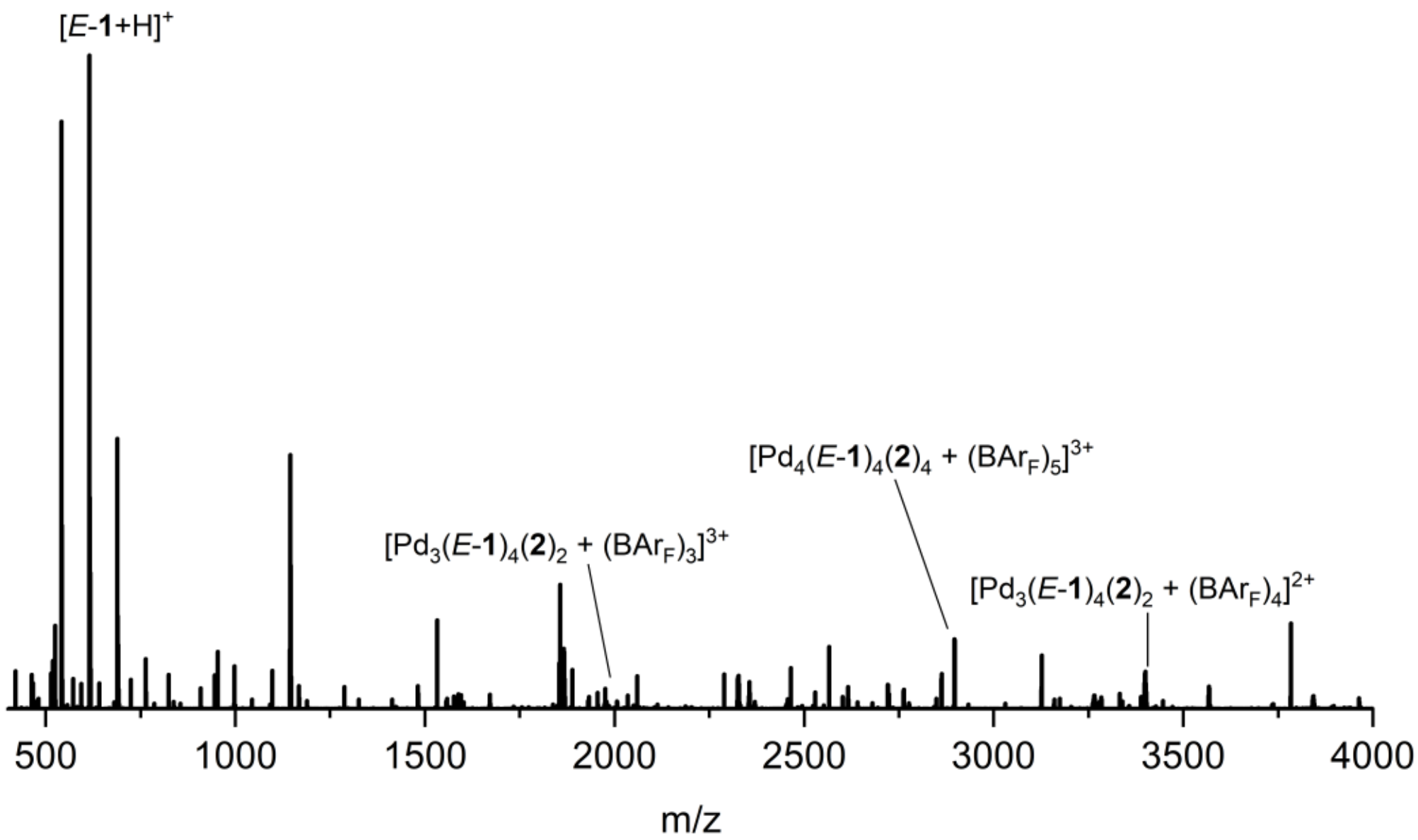

Figure S103. ESI-MS spectrum of the mixture of $[Pd_3(E\text{-}\mathbf{1})_4(\mathbf{2})_2](BAr_F)_6$ and $[Pd_4(E\text{-}\mathbf{1})_4(\mathbf{2})_4](BAr_F)_8$ ([Pd] = 2.0 mM) in MeCN-d_3.

Table S26. Zoom scans of select ESI-MS peaks, with simulated isotope patterns of $[Pd_3(E\text{-}\mathbf{1})_4(\mathbf{2})_2](BAr_F)_6$ and $[Pd_4(E\text{-}\mathbf{1})_4(\mathbf{2})_4](BAr_F)_8$([Pd] = 2.0 mM) in MeCN-$d_3$.

$[Pd_3(E\text{-}\mathbf{1})_4(\mathbf{2})_2 + (BAr_F)_4]^{2+}$	$[Pd_3(E\text{-}\mathbf{1})_4(\mathbf{2})_2 + (BAr_F)_3]^{3+}$
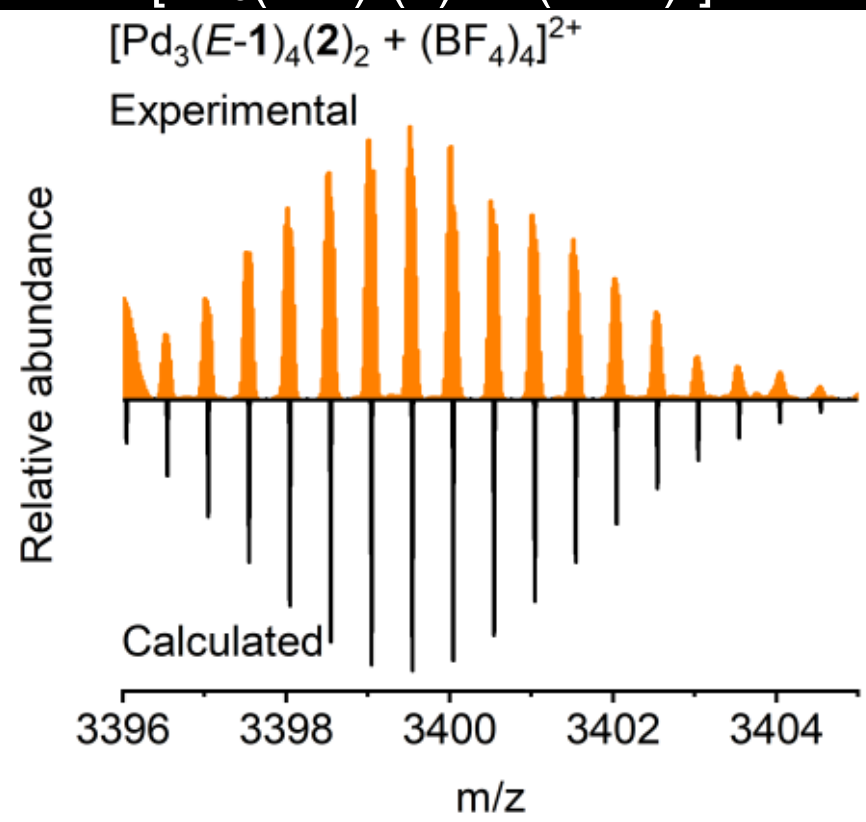	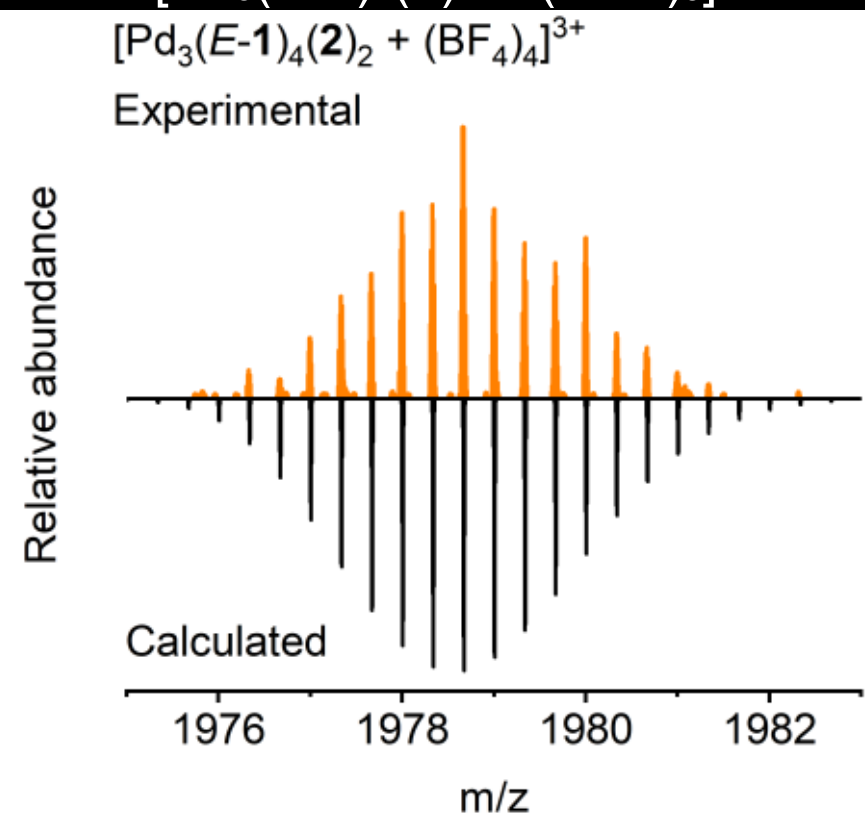
$[Pd_4(E\text{-}\mathbf{1})_4(\mathbf{2})_4 + (BAr_F)_5]^{3+}$	
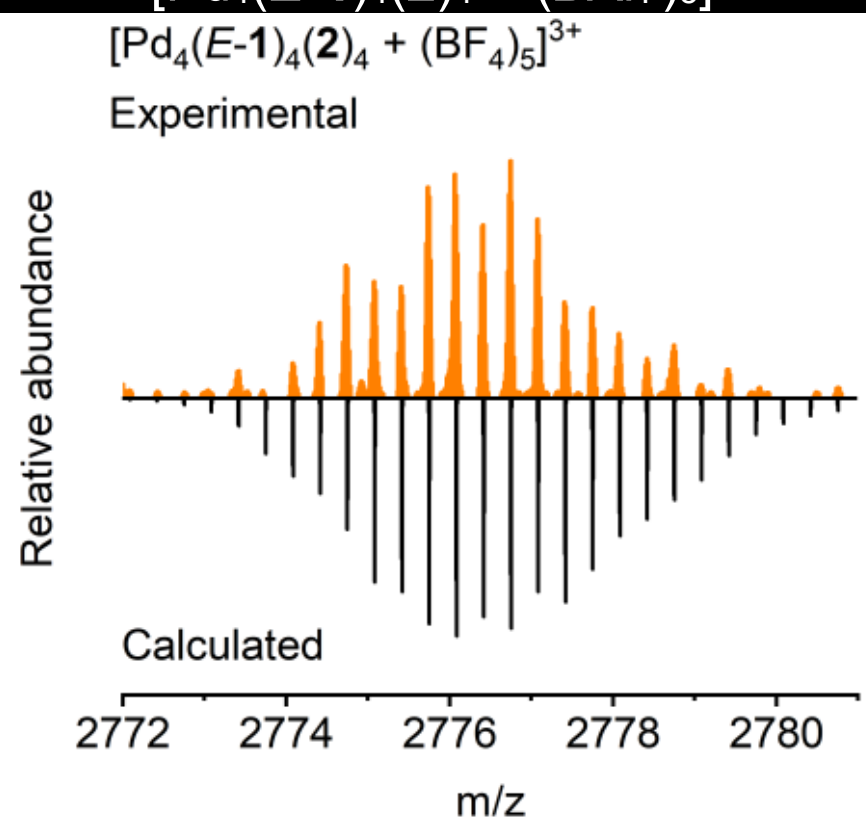	

6.5 Photoswitching and thermal stability of heteroleptic $[Pd_2(Z\text{-}\mathbf{1})_2(\mathbf{2})_2](BAr_F)_4$

6.5.1 Irradiating $[Pd_2(Z\text{-}\mathbf{1})_2(\mathbf{2})_2](BAr_F)_4$ with 530 nm and 424 nm light

A sample of the cages mixture $[Pd_3(E\text{-}\mathbf{1})_6](BArF)_6$, $[Pd_3(\mathbf{2})_6](BArF)_6$, $[Pd_3(E\text{-}\mathbf{1})_4(\mathbf{2})_2](BArF)_6$ and $[Pd_4(E\text{-}\mathbf{1})_4(\mathbf{2})_4](BArF)_8$ prepared in section 6.1 (Figure S104i and Figure S105i) was irradiated with 530 nm light for 10 minutes before ^1H and ^{19}F NMR spectra were recorded (Figure S104ii and Figure S105ii). This resulted in the quantitative disassembly of the different self-assembled species present and the formation of a new, highly symmetric species, which could be identified as $[Pd_2(Z\text{-}\mathbf{1})_2(\mathbf{2})_2](BAr_F)_4$.

Irradiation of $[Pd_2(Z\text{-}\mathbf{1})_2(\mathbf{2})_2](BAr_F)_4$ with 424 nm light led to the disassembly of the metastable lantern-shaped cage and the partial reformation of the cages mixture $[Pd_3(E\text{-}\mathbf{1})_6](BArF)_6$, $[Pd_3(\mathbf{2})_6](BArF)_6$, $[Pd_3(E\text{-}\mathbf{1})_4(\mathbf{2})_2](BArF)_6$ and $[Pd_4(E\text{-}\mathbf{1})_4(\mathbf{2})_4](BArF)_8$ (Figure S104iii and Figure S105iii).

By irradiating this mixture with 530 nm light for 10 minutes, the $[Pd_2(Z\text{-}\mathbf{1})_2(\mathbf{2})_2](BAr_F)_4$ species could be restored (Figure S104iv and Figure S105iv). This demonstrates that $[Pd_2(Z\text{-}\mathbf{1})_2(\mathbf{2})_2](BAr_F)_4$ can be reversibly and quantitatively assembled and disassembled using visible light.

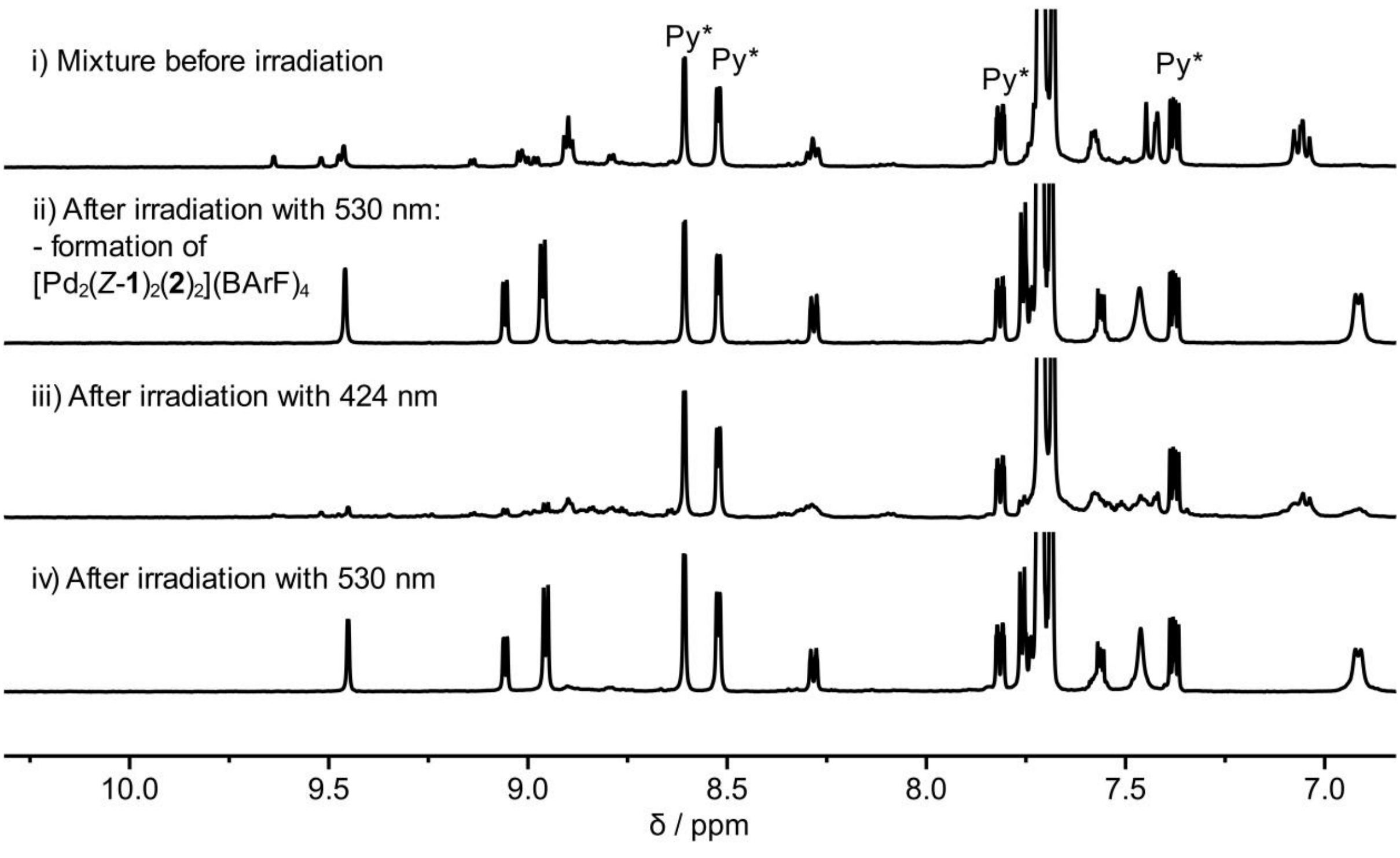

Figure S104. Partial ^1H NMR spectra (600 MHz, 298 K, MeCN-d_3) of i) a mixture of the cages $[Pd_3(E\text{-}\mathbf{1})_6](BArF)_6$, $[Pd_3(\mathbf{2})_6](BArF)_6$, $[Pd_3(E\text{-}\mathbf{1})_4(\mathbf{2})_2](BArF)_6$ and $[Pd_4(E\text{-}\mathbf{1})_4(\mathbf{2})_4](BArF)_8$ before irradiation, ii) the previous sample after irradiation with 530 nm light for 10 minutes, forming the metastable heteroleptic cage $[Pd_2(Z\text{-}\mathbf{1})_2(\mathbf{2})_2](BAr_F)_4$, iii) the previous sample after irradiation with 424 nm light for 10 minutes, and iv) the previous sample after irradiation with 530 nm light for 10 minutes, reassembling metastable $[Pd_2(Z\text{-}\mathbf{1})_2(\mathbf{2})_2](BAr_F)_4$.

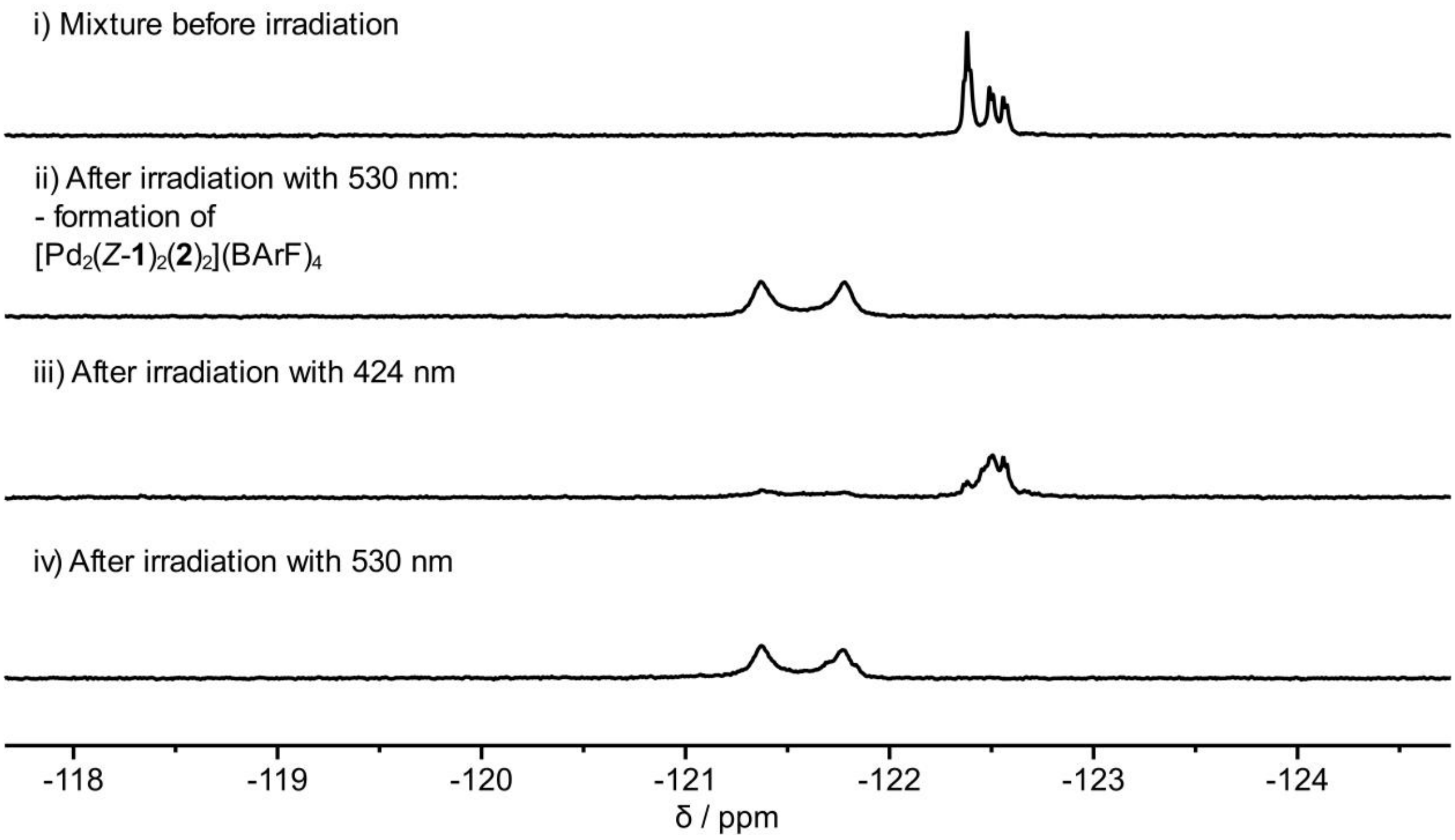

Figure S105. ^{19}F NMR spectra (565 MHz, 298 K, MeCN-*d*$_3$) of i) a mixture of the cages $[Pd_3(E\text{-}\mathbf{1})_6](BArF)_6$, $[Pd_3(\mathbf{2})_6](BArF)_6$, $[Pd_3(E\text{-}\mathbf{1})_4(\mathbf{2})_2](BArF)_6$ and $[Pd_4(E\text{-}\mathbf{1})_4(\mathbf{2})_4](BArF)_8$ before irradiation, ii) the previous sample after irradiation with 530 nm light for 10 minutes, forming the metastable heteroleptic cage $[Pd_2(Z\text{-}\mathbf{1})_2(\mathbf{2})_2](BAr_F)_4$, iii) the previous sample after irradiation with 424 nm light for 10 minutes, and iv) the previous sample after irradiation with 530 nm light for 10 minutes, reassembling metastable $[Pd_2(Z\text{-}\mathbf{1})_2(\mathbf{2})_2](BAr_F)_4$.

6.5.2 Determining the PSS distribution of $[Pd_2(Z\text{-}1)_2(2)_2](BAr_F)_4$ in MeCN-d_3 after irradiation with 530 nm light

A sample of $[Pd_2(Z\text{-}\mathbf{1})_2(\mathbf{2})_2](BAr_F)_4$ in MeCN-d_3 was prepared according to the procedure described in section 6.1 ([Pd] = 2.0 mM). Next, we wanted to determine the PSS distribution of $[Pd_2(Z\text{-}\mathbf{1})_2(\mathbf{2})_2](BAr_F)_4$ after the cage had been formed under 530 nm light irradiation for 10 minutes.

To determine how much *Z*-**1** and possibly *E*-**1** ligand is present in the cage sample, 4-dimethylaminopyridine (DMAP, 164 mM, 128 µL in MeCN-d_3, 21.0 µmol, 20.0 equiv.) was added to the sample. This afforded a solution containing free ligand **1**, free ligand **2**, free DMAP and $[Pd(DMAP)_4](BF_4)_2$. Analysis of the resulting ^1H and ^{19}F NMR spectra showed, that only *Z*-**1** and no *E*-**1** isomer is present in the mixture. This indicates, that once the cage mixture $[Pd_3(E\text{-}\mathbf{1})_6](BArF)_6$, $[Pd_3(\mathbf{2})_6](BArF)_6$, $[Pd_3(E\text{-}\mathbf{1})_4(\mathbf{2})_2](BArF)_6$ and $[Pd_4(E\text{-}\mathbf{1})_4(\mathbf{2})_4](BArF)_8$ is irradiated with 530 nm light and $[Pd_2(Z\text{-}\mathbf{1})_2(\mathbf{2})_2](BAr_F)_4$ is formed, a PSS containing 100% *Z*-**1** and 0% *E*-**1** is generated.

This PSS is higher than the PSS of the free ligand **1**, where irradiation with 530 nm light generated a PSS containing 92% *Z*-**1** and 8% *E*-**1**. This indicates that coordination to palladium(II) improves the selectivity towards the metastable isomer. Similar behavior was observed in our previously published example.[9] We suggest that the shape-complementarity of ligands *Z*-**1** and **2** also contributes to the selectivity towards the metastable isomer.

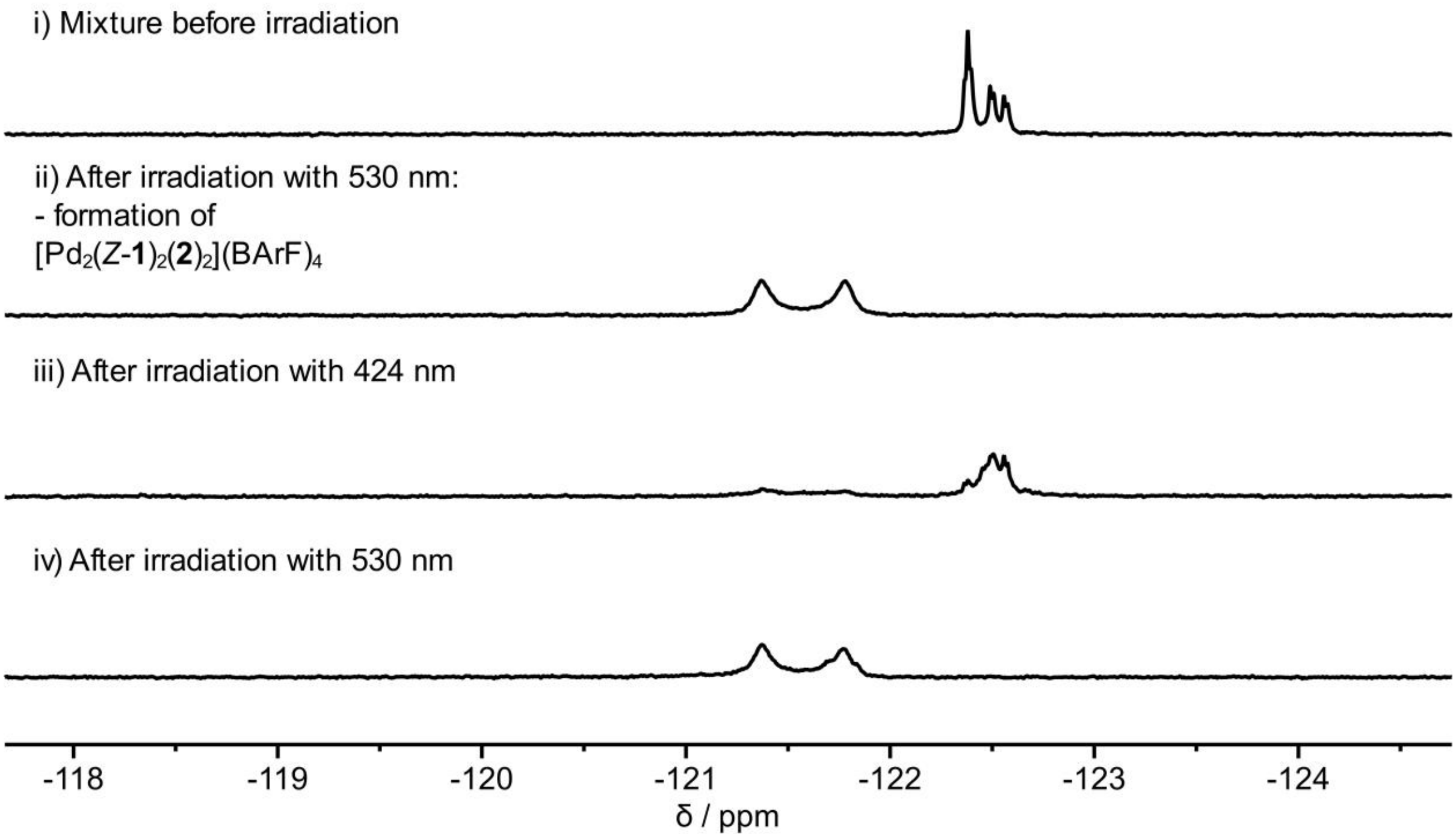

Figure S106. Partial ^1H NMR spectra (600 MHz, 298 K, MeCN-d_3) of i) $[Pd_2(Z\text{-}\mathbf{1})_2(\mathbf{2})_2](BAr_F)_4$ ([Pd] = 2.0 mM), and ii) the previous sample after the addition of DMAP (20 equiv.).

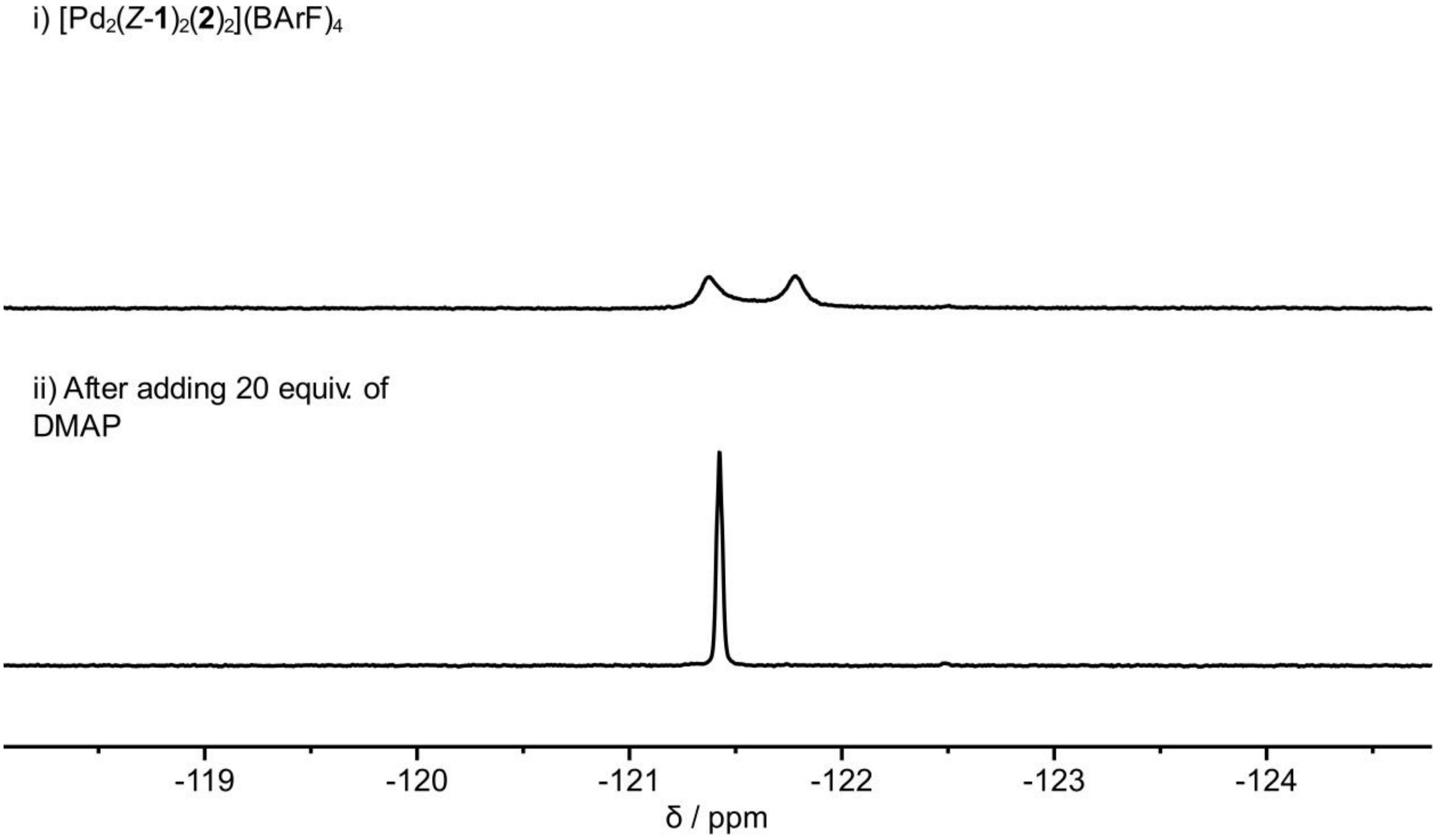

Figure S107. ^{19}F NMR spectra (565 MHz, 298 K, MeCN-d_3) of i) [Pd_2(*Z*-**1**)$_2$(**2**)$_2$](BAr_F)$_4$ ([Pd] = 2.0 mM), and ii) the previous sample after the addition of DMAP (20 equiv.).

6.5.3 Thermal half-life of $[Pd_2(Z\text{-}\mathbf{1})_2(\mathbf{2})_2](BAr_F)_4$ in DMSO-d_6

The thermal half-life of $[Pd_2(Z\text{-}\mathbf{1})_2(\mathbf{2})_2](BAr_F)_4$ was monitored at different temperatures. For this, a sample of $[Pd_2(Z\text{-}\mathbf{1})_2(\mathbf{2})_2](BAr_F)_4$ was prepared by mixing ligand *E*-**1** (250 µL, 4.0 mM, 2.0 equiv., in MeCN-d_3) with ligand **2** (250 µL, 4.0 mM, 2.0 equiv., in MeCN-d_3) and then adding $[Pd(Py^*)_4](BArF)_2$ stock solution (40 µL, 29.7 mM, 1.0 equiv.). Next, the orange sample was irradiated with 530 nm light for 10 minutes to afford a sample of $[Pd_2(Z\text{-}\mathbf{1})_2(\mathbf{2})_2](BAr_F)_4$ ([Pd] = 2.0 mM).

The thermal half-life was first measured in MeCN-d_3, but no thermal back-isomerization of the metastable cage was observed even after heating the sample at 60 degrees for 16 hours. Since MeCN-d_3 could not be heated higher, we decided to exchange the solvent to DMSO-d_6.

For this reason, the $[Pd_2(Z\text{-}\mathbf{1})_2(\mathbf{2})_2](BAr_F)_4$ in MeCN-d_3 solution was transferred to a 4 mL vial. The sample was heated to 50 °C under a flow of argon to remove all the solvent. The residue was then redissolved in DMSO-d_6 (500 µL).

The $[Pd_2(Z\text{-}\mathbf{1})_2(\mathbf{2})_2](BAr_F)_4$ in DMSO-d_6 sample was then heated to 343 K and the thermal back-isomerization was monitored using ^{1}H NMR spectroscopy over 10 hours, recording an ^{1}H NMR spectrum every 5 minutes. The same was repeated for 353, 363 and 373 K. The concentration of $[Pd_2(Z\text{-}\mathbf{1})_2(\mathbf{2})_2](BAr_F)_4$ was measured by integrating the signal at 6.95 ppm and subsequent normalisation. It was observed that 343, 353 and 363 K have little to no effect on $[Pd_2(Z\text{-}\mathbf{1})_2(\mathbf{2})_2](BAr_F)_4$ over 10 hours, already indicating a high thermal stability of the self-assembled species. After heating the sample to 373 K we could see a significant decrease in the signal intensity of $[Pd_2(Z\text{-}\mathbf{1})_2(\mathbf{2})_2](BAr_F)_4$ at 6.95 ppm. Only the kinetic data after heating to 373 K could be fit to a mono-exponential. The rate constant was determined to be 3.3×10^{-5} s^{-1}, corresponding to a thermal half-life of ~20 years at room temperature and an apparent thermal barrier of 124.1 kJ mol^{-1}.

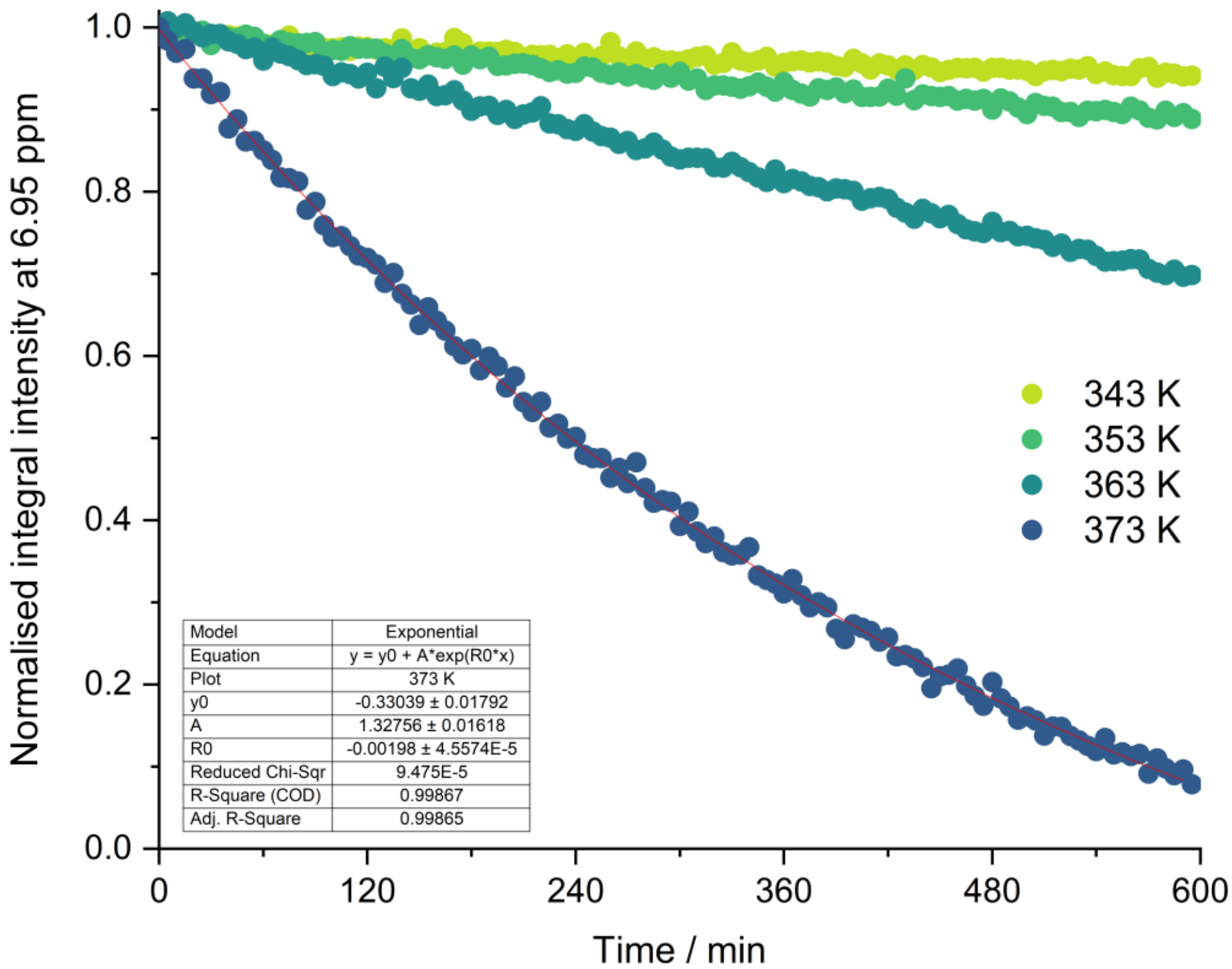

Figure S108. ^{1}H NMR (600 MHz, 298 K, DMSO-d_6) signal intensity of the peak at 6.95 ppm of the thermal back-isomerization of $[Pd_2(Z\text{-}\mathbf{1})_2(\mathbf{2})_2](BAr_F)_4$ at 343, 353, 363 K and 373 K.

7 Integrative self-sorting of photoswitchable cages

7.1 General

We have previously reported[2] a thermodynamically stable heteroleptic cage formed from ligand *E*-**6** and **2**. The heteroleptic cage $[Pd_2(E\text{-}\mathbf{6})_2(\mathbf{2})_2](BAr_F)_4$ forms only when ligand **6** is in the *E*-isomeric form, since only the *E*-form is shape complementary to ligand **2** (Figure S109). Upon irradiation with 530 nm light, ligand *E*-**6** isomerizes to *Z*-**6**, and is now no longer shape complementary to ligand **2**. In consequence, the heteroleptic cage $[Pd_2(E\text{-}\mathbf{6})_2(\mathbf{2})_2](BAr_F)_4$ gets disassembled, and homoleptic $[Pd(Z\text{-}\mathbf{6})_2](BAr_F)_2$, $[Pd_3(\mathbf{2})_6](BAr_F)_6$ and other ill-defined structures are formed. In summary, heteroleptic $[Pd_2(E\text{-}\mathbf{6})_2(\mathbf{2})_2](BAr_F)_4$ only exists in the dark and gets disassembled after irradiation with 530 nm light.

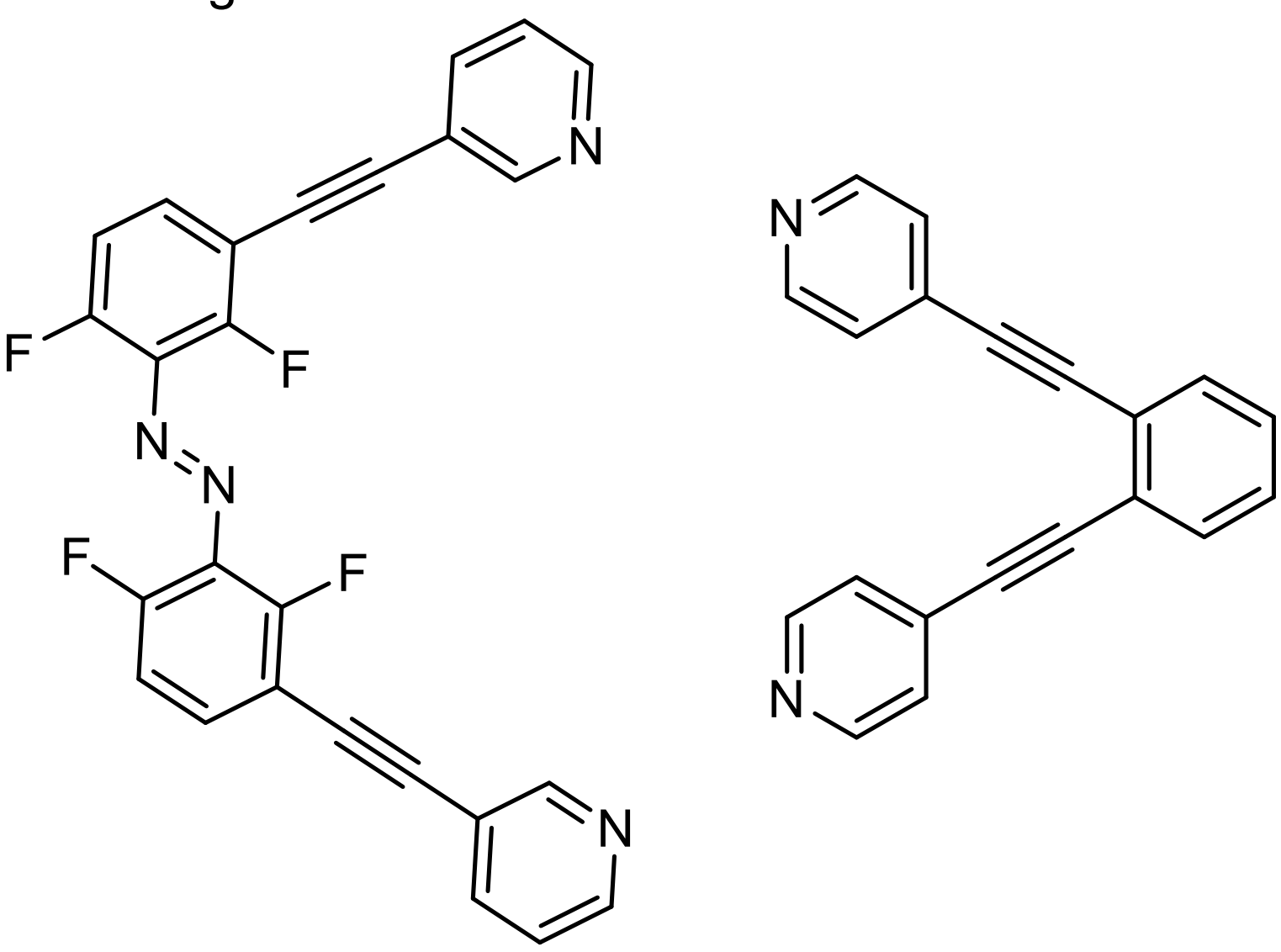

Ligand *E*-**6** Ligand **2**

Figure S109. Structure of ligand *E*-**6**, which is shape complementary to ligand **2**.

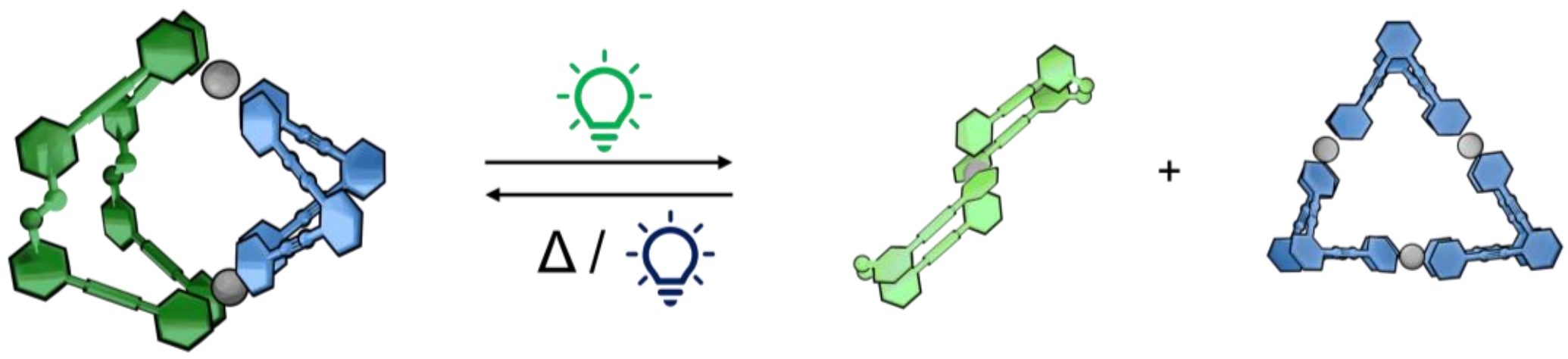

$[Pd_2(E\text{-}\mathbf{6})_2(\mathbf{2})_2]^{4+}$ $[Pd(Z\text{-}\mathbf{6})_2]^{2+}$ $[Pd_3(\mathbf{2})_6]^{6+}$

Figure S110. Self-assembly of ligand *E*-**6** and **2** with Pd(II). The heteroleptic cage $[Pd_2(E\text{-}\mathbf{6})_2(\mathbf{2})_2](BAr_F)_4$ switches to $[Pd(Z\text{-}\mathbf{6})_2](BAr_F)_2$ and $[Pd_3(\mathbf{2})_6](BAr_F)_6$ upon irradiation with 530 nm light.

With the metastable cage $[Pd_2(Z\text{-}\mathbf{1})_2(\mathbf{2})_2](BAr_F)_4$ in hand, which only exists after irradiation with 530 nm light, we wanted to investigate, whether we can mix ligands *E*-**1,** *E*-**6** and **2** to achieve self-sorting before and after light irradiation.

In the dark, out of all the known possible cages (homoleptic $[Pd_3(E\text{-}\mathbf{1})_6](BAr_F)_6$, $[Pd_2(E\text{-}\mathbf{6})_4](BAr_F)_4$, $[Pd_3(\mathbf{2})_6](BAr_F)_6$, heteroleptic $[Pd_3(E\text{-}\mathbf{6})_4(\mathbf{2})_2](BAr_F)_6$, $[Pd_4(E\text{-}\mathbf{6})_4(\mathbf{2})_4](BAr_F)_8$, $[Pd_2(E\text{-}\mathbf{6})_2(\mathbf{2})_2](BAr_F)_4$ and possibly mixed *E*-**1** and *E*-**6** structures), we propose that heteroleptic $[Pd_2(E\text{-}\mathbf{6})_2(\mathbf{2})_2](BAr_F)_4$ is favored due to the shape complementarity of ligands *E*-**6** and **2**. This would leave *E*-**1** to form homoleptic $[Pd_3(E\text{-}\mathbf{1})_6](BAr_F)_6$ (Figure S111). Upon irradiation with 530 nm light, both *E*-**1** and *E*-**6** will isomerize to *Z*-**1** and *Z*-**6**. Now ligand *Z*-**1** is shape complementary to ligand **2**, which is why we propose

that heteroleptic cage $[Pd_2(Z\text{-}\mathbf{1})_2(\mathbf{2})_2](BAr_F)_4$ together with homoleptic $[Pd(Z\text{-}\mathbf{6})_2](BAr_F)_2$ should be the preferred structures after light irradiation (Figure S111).

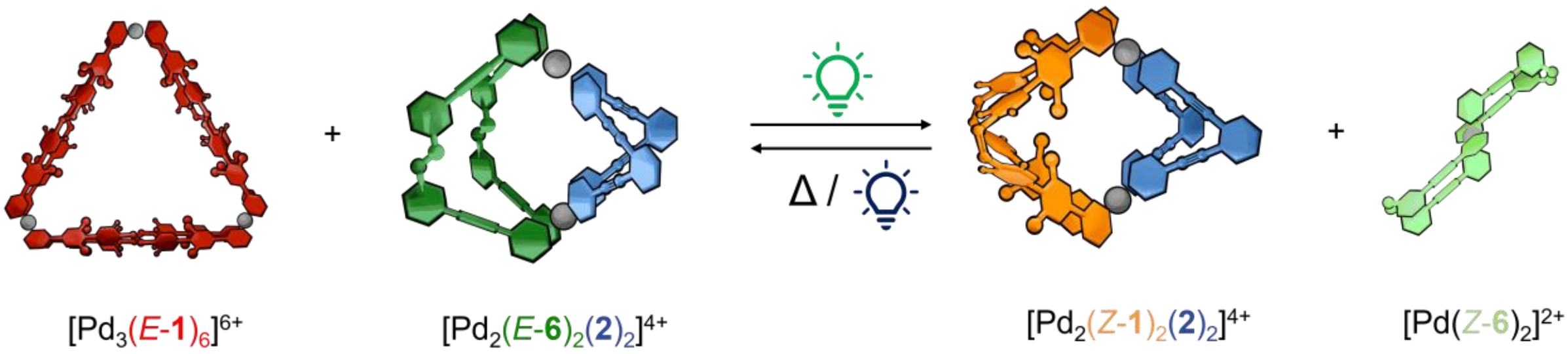

Figure S111. Proposed self-sorting of ligands **1**, **6** and **2** in the dark and after irradiation with 530 nm light.

7.2 Self-sorting of ligands *E*-1, *E*-6 and 2

To prepare an isomerically pure sample of ligand *E*-**1**, ligand **1** (7.9 mg, 12.9 µmol, mixture of *E*-**1** and *Z*-**1**) was dissolved in MeCN-d_3 (3.0 mL) in a sealed microwave vial ([ligand **1**] = 4.3 mM). The mixture was then heated to 130 °C for 3 hours to ensure all possible *Z*-**1** isomer back-isomerized to the *E*-**1** isomer.

The same procedure was repeated for ligand **6**: To prepare an isomerically pure sample of ligand *E*-**6**, ligand **6** (5.9 mg, 13.0 µmol, mixture of *E*-**6** and *Z*-**6**) was dissolved in MeCN-d_3 (3.0 mL) in a sealed microwave vial ([ligand **6**] = 4.3 mM). The mixture was then heated to 130 °C for 3 hours to ensure all possible *Z*-**6** isomer back-isomerized to the *E*-**6** isomer.

Ligand *E*-**1** solution (250 µL, 1.1 µmol, 4.3 mM, 1.0 equiv.) was mixed with ligand *E*-**6** solution (250 µL, 1.1 µmol, 4.3 mM, 1.0 equiv.) and ligand **2** solution (125 µL, 1.1 µmol, 9.3 mM, 1.0 equiv.). The 1:1:1 stoichiometry of *E*-**1,** *E*-**6** and **2** was verified by ^{1}H NMR analysis (Figure S112i). Then $[Pd(Py^*)_4](BAr_F)_2$ stock solution (56 µL, 1.7 µmol, 29.7 mM, 1.5 equiv.) was added to the ligand mixture. The sample was left to equilibrate in a sand bath at 50 °C for one hour. ^{1}H NMR spectroscopic analysis revealed the clean formation of heteroleptic [Pd$_2$(*E*-**6**)$_2$(**2**)$_2$](BAr$_F$)$_4$ and double-walled triangle [Pd$_3$(*E*-**1**)$_6$](BAr$_F$)$_6$ together with free Py* (Figure S112ii). We could observe self-sorting of ligands *E*-**1,** *E*-**6** and **2** combined with Pd(II) due to shape complementarity of ligands *E*-**6** and **2**.

The NMR sample was then irradiated with 530 nm light for 30 minutes. Immediate ^{1}H NMR analysis showed switching of ligands *E*-**1** and *E*-**6** to *Z*-**1** and *Z*-**6** and rearrangement of the system to form several new species. However, equilibration of the same sample in a sand bath at 50 °C overnight showed the complete rearrangement of the system and almost quantitative assembly (Figure S112iii) of the proposed [Pd$_2$(*Z*-**1**)$_2$(**2**)$_2$](BAr$_F$)$_4$ together with homoleptic [Pd(*Z*-**6**)$_2$](BAr$_F$)$_2$, and some residual [Pd$_2$(*E*-**6**)$_2$(**2**)$_2$](BAr$_F$)$_4$ due to incomplete switching of ligand *E*-**6** (PSS$_{530}$ 12:88 *E*:*Z*).[2]

The NMR sample was then irradiated with 424 nm light for 30 minutes. Immediate ^{1}H NMR analysis showed switching of ligands *Z*-**1** and *Z*-**6** to *E*-**1** and *E*-**6** and rearrangement of the system to form several new species. Equilibration of the same sample in a sand bath at 50 °C for four hours showed the rearrangement of the system and reassembly (Figure S112iv) of heteroleptic [Pd$_2$(*Z*-**1**)$_2$(**2**)$_2$](BAr$_F$)$_4$ and homoleptic [Pd$_3$(*E*-**1**)$_6$](BAr$_F$) together with some residual [Pd$_2$(*E*-**6**)$_2$(**2**)$_2$](BAr$_F$)$_4$ due to incomplete switching of ligand *E*-**1** and some other minor additional species.

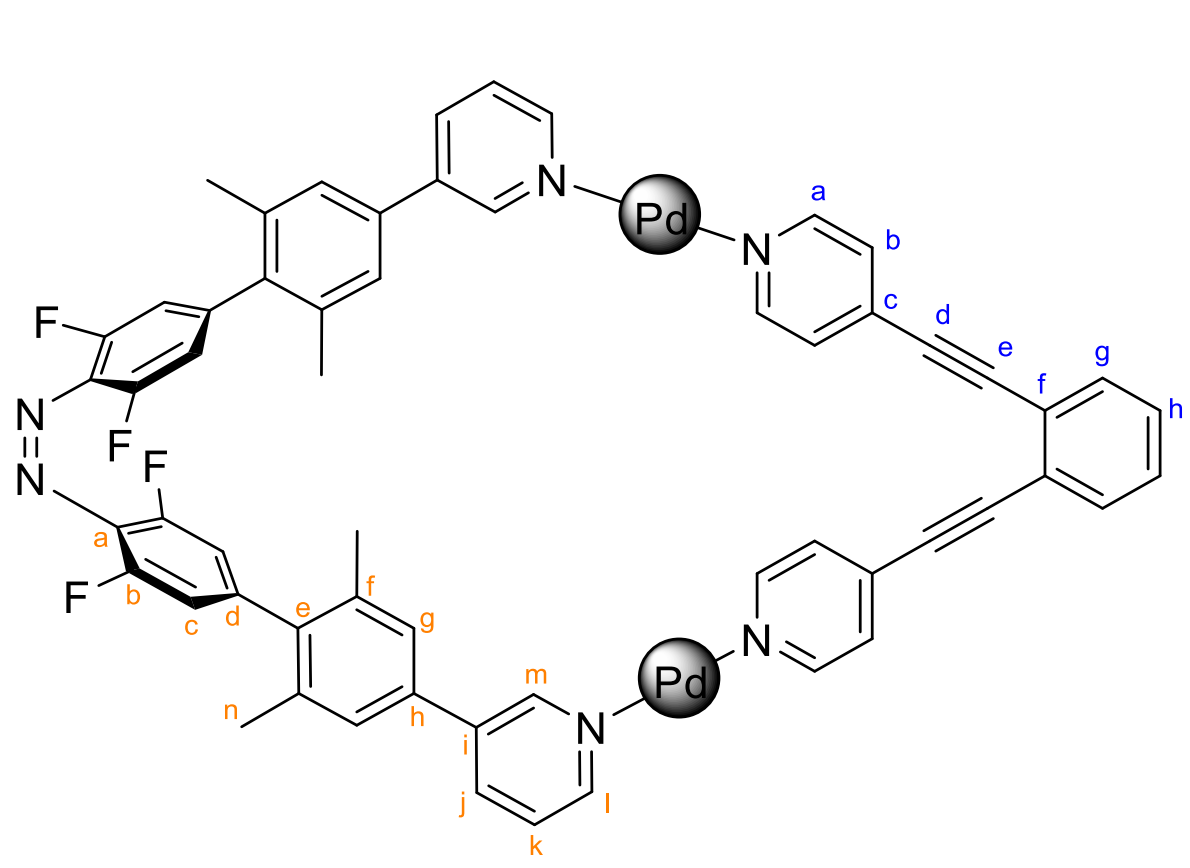

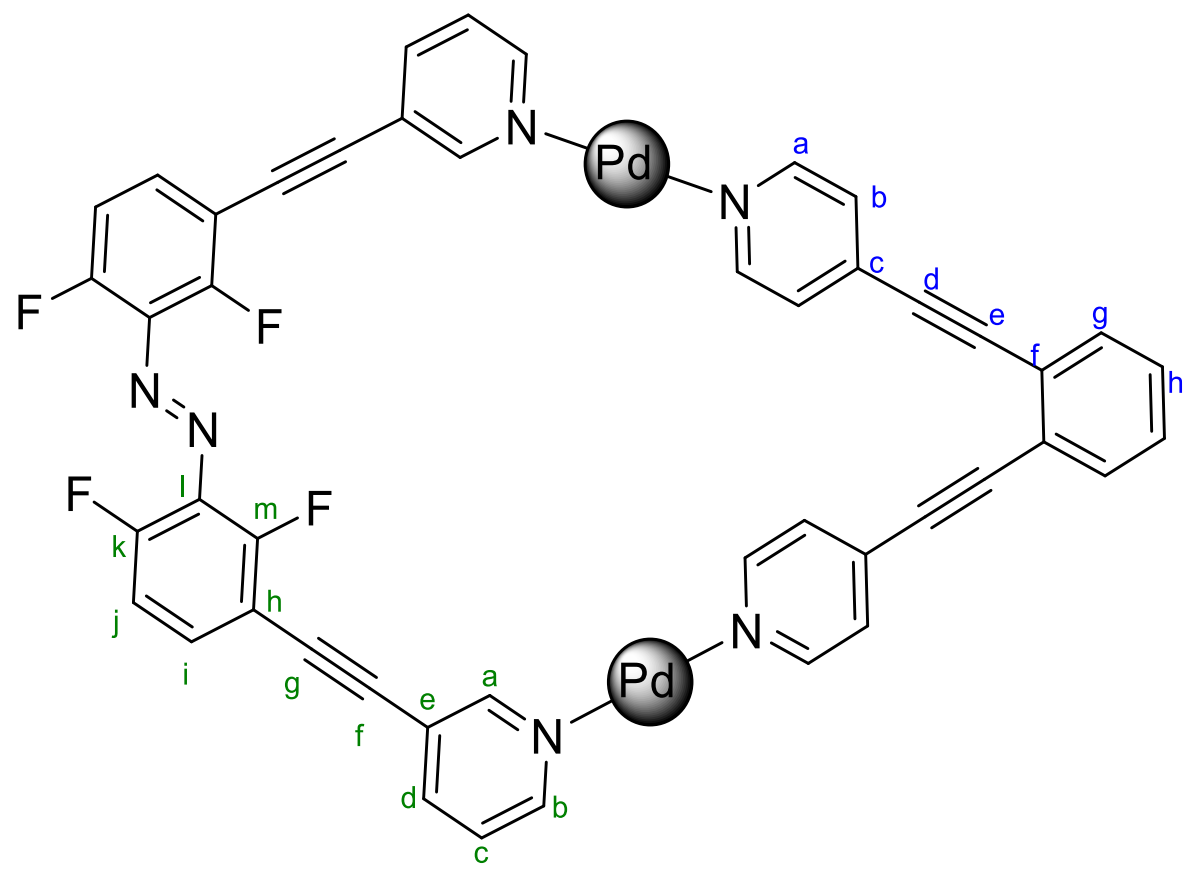

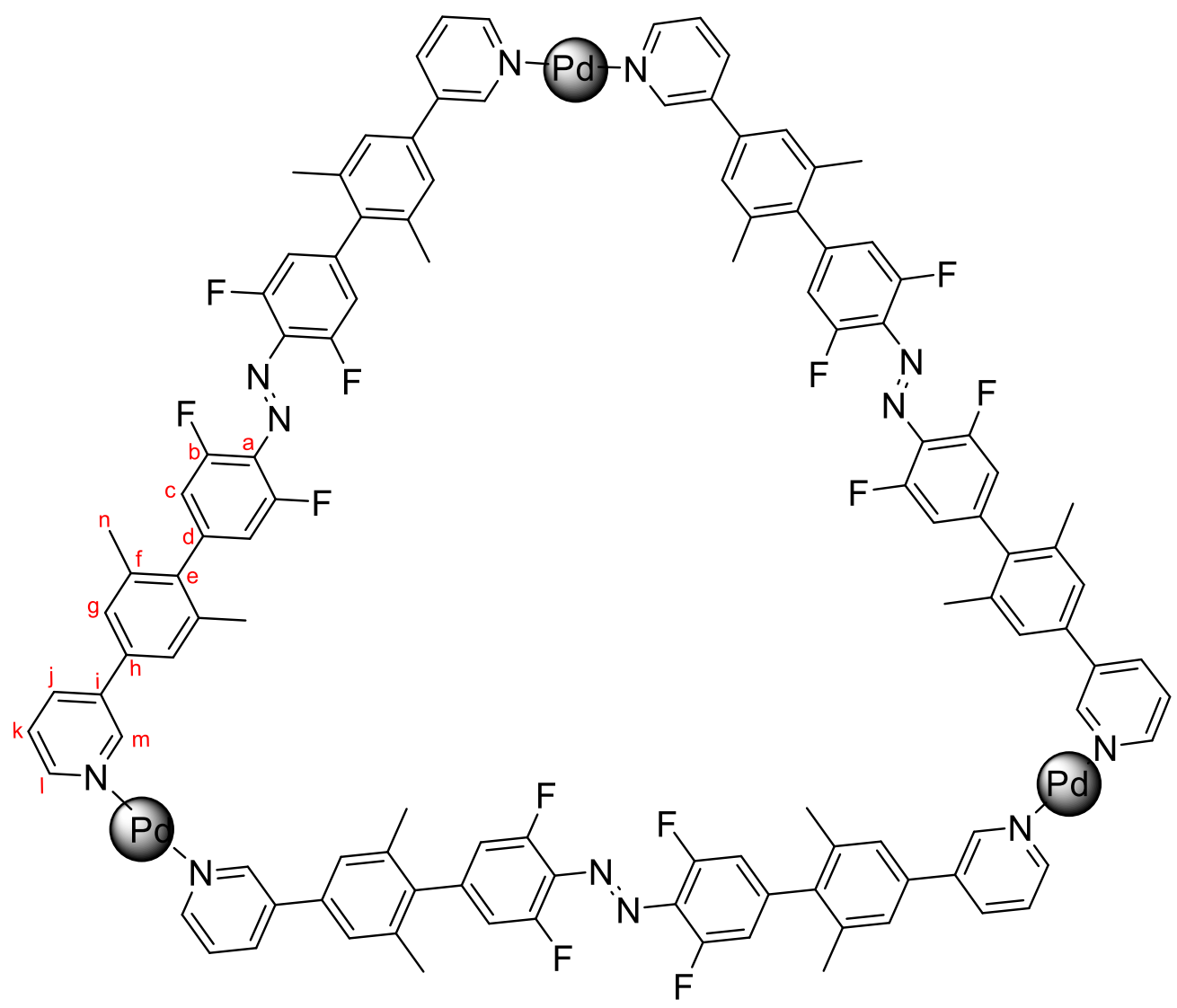

Red assignments correspond to signals arising from ligand *E*-**1**, green assignments to ligand *E*-**6** and blue assignments correspond to ligand **2**. After irradiation, orange assignments correspond to ligand *Z*-**1**, light green assignments to ligand *Z*-**6** and blue assignments correspond to ligand **2.**

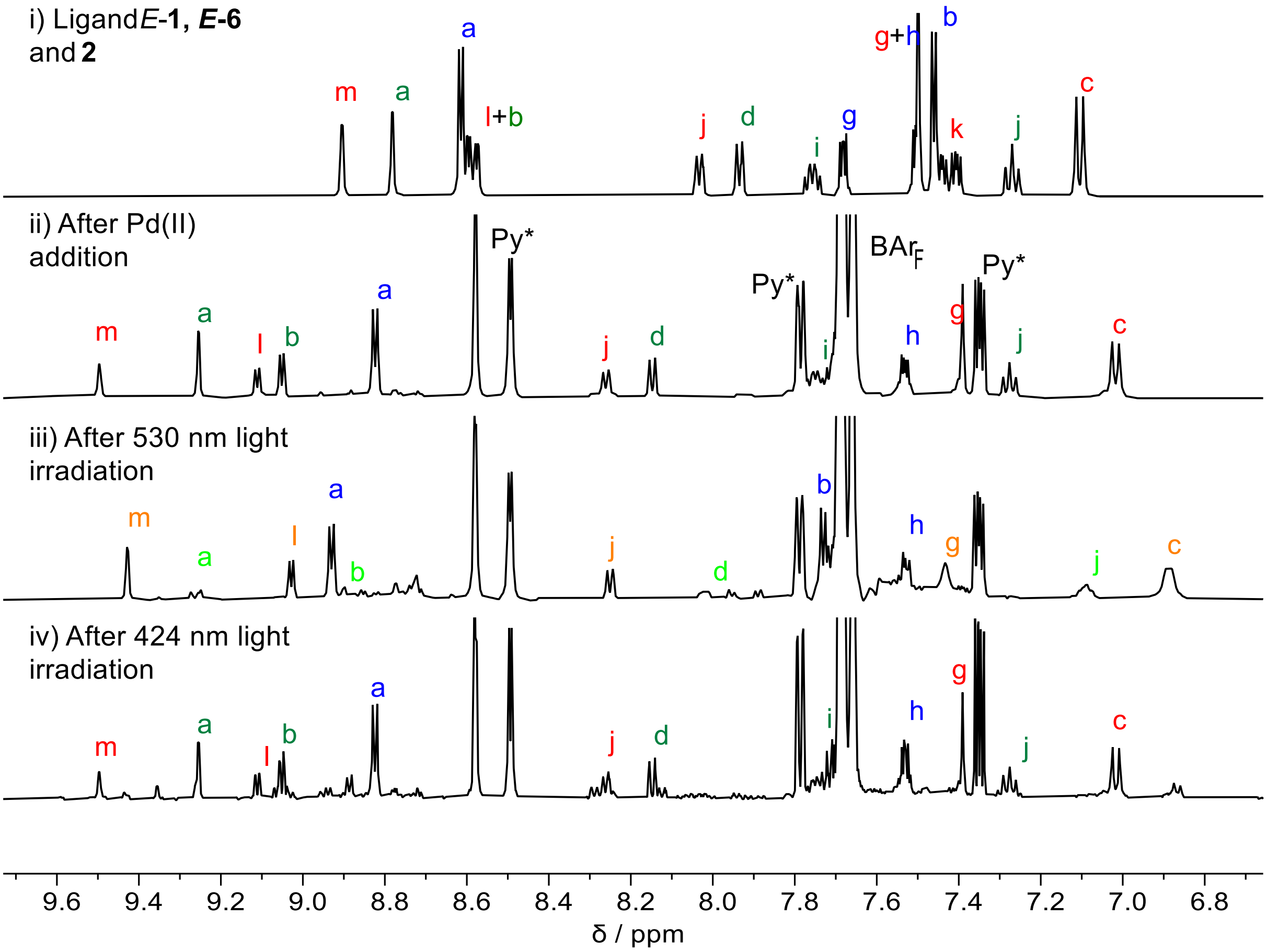

Figure S112. Partial ^{1}H NMR spectra (600 MHz, 298 K, MeCN-d_3) of i) a 1:1:1 mixture of ligands *E*-**1**, *E*-**6** and **2**, ii) after the addition of $[Pd(Py^*)_4](BArF)_2$ stock solution and equilibration at 50 °C for one hour, iii) the previous sample after irradiation with 530 nm light for 30 minutes and after equilibration at 50 °C overnight, and iv) the previous sample after irradiation with 424 nm light for 30 minutes and after equilibration at 50 °C for 4 hours.

7.3 Competition experiments

Since both ligands *E*-**6** and *Z*-**1** can form heteroleptic cages with ligand **2**, we wanted to investigate whether there is any preference for one ligand over the other to pair with ligand **2**.

7.3.1 Adding ligand *E*-6 to $[Pd_2(Z\text{-}1)_2(2)_2](BAr_F)_4$

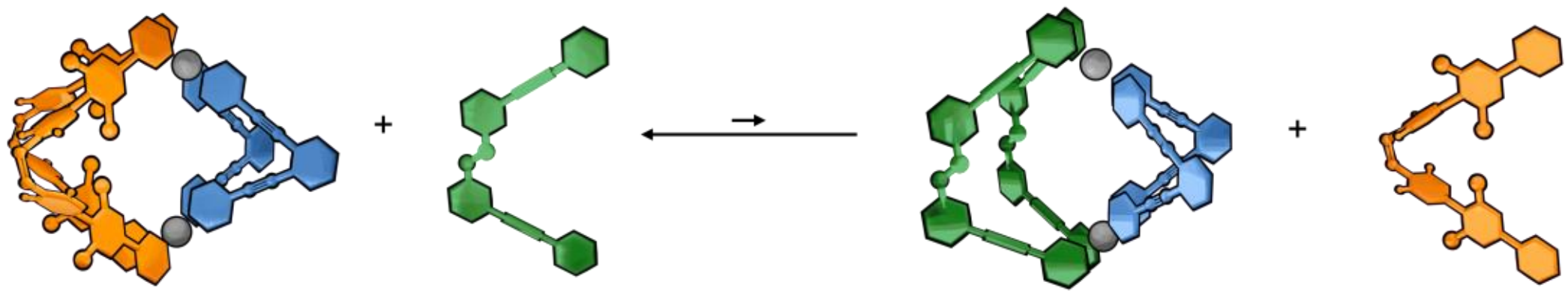

For this reason, we prepared a sample of $[Pd_2(Z\text{-}\mathbf{1})_2(\mathbf{2})_2](BAr_F)_4$ in MeCN-d_3 ([Pd] = 2.1 mM, for preparation details see section 6.1). The metastable cage $[Pd_2(Z\text{-}\mathbf{1})_2(\mathbf{2})_2](BAr_F)_4$ in MeCN-d_3 was then transferred to a 4 mL vial. The sample was heated to 50 °C under a flow of argon to remove all the solvent. The residue was then redissolved in MeCN-d_3 (300 µL) to afford a sample of $[Pd_2(Z\text{-}\mathbf{1})_2(\mathbf{2})_2](BAr_F)_4$ ([Pd] = 3.5 mM, 300 µL, 1.0 equiv., MeCN-d_3) for catalysis (Figure S113i, a more concentrated sample was prepared in order to keep the final concentrations constant). Then a solution of ligand *E*-**6** (250 µL, 1.1 µmol, 4.3 mM, 1.0 equiv.) was added (Figure S113ii). The solution was mixed well and the rearrangement of the self-assembled species was monitored using ^{1}H NMR spectroscopy over 10 hours (recording a spectrum every 5 minutes).

After ten hours we could observe the formation of $[Pd_2(E\text{-}\mathbf{6})_2(\mathbf{2})_2](BAr_F)_4$ and what appeared to be a trileptic cage $[Pd_2(Z\text{-}\mathbf{1})(E\text{-}\mathbf{6})(\mathbf{2})_2](BAr_F)_4$ but metastable $[Pd_2(Z\text{-}\mathbf{1})_2(\mathbf{2})_2](BAr_F)_4$ and free ligand *E*-**6** remained the major species present in the mixture (Figure S113iii). Comparison of ligand *E*-**6** integral right in the beginning and after 10 hours revealed that 92% of ligand *E*-**6** is still present as a free ligand in solution (Figure S114). We first proposed that this result is due to slow ligand exchange, but repetition of the same experiment at 50 degrees led to the same result. It appears that metastable cage $[Pd_2(Z\text{-}\mathbf{1})_2(\mathbf{2})_2](BAr_F)_4$ is the preferred species. To confirm this result, we carried out the same experiment by adding ligand *Z*-**1** to a sample of $[Pd_2(E\text{-}\mathbf{6})_2(\mathbf{2})_2](BAr_F)_4$ (see next section 7.3.2).

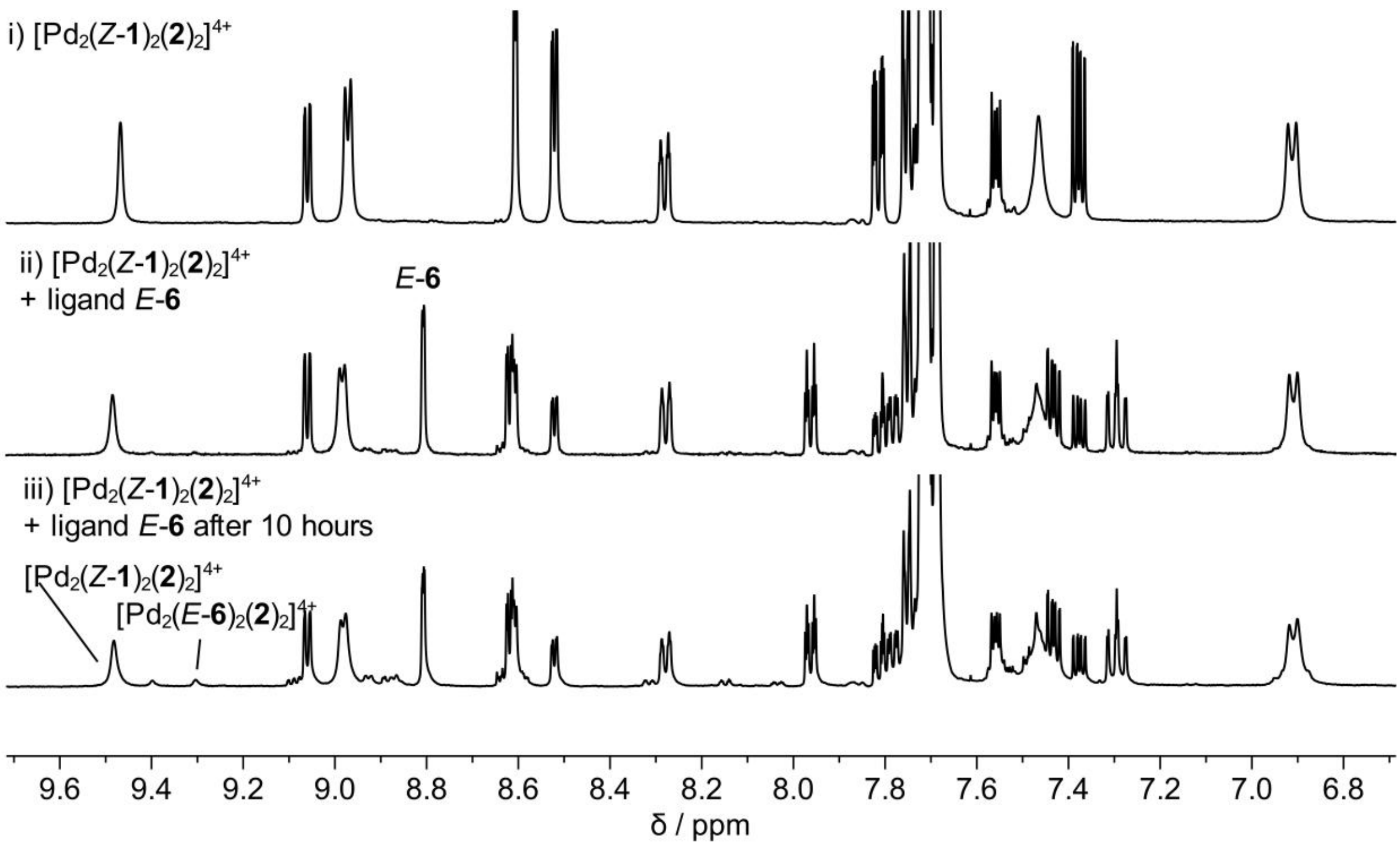

Figure S113. Partial ^{1}H NMR spectra (600 MHz, 298 K, MeCN-d_3) of i) $[Pd_2(Z\text{-}\mathbf{1})_2(\mathbf{2})_2](BAr_F)_4$ ii) the same sample immediately after the addition of ligand *E*-**6** stock solution, and iii) the previous sample after 10 hours at room temperature.

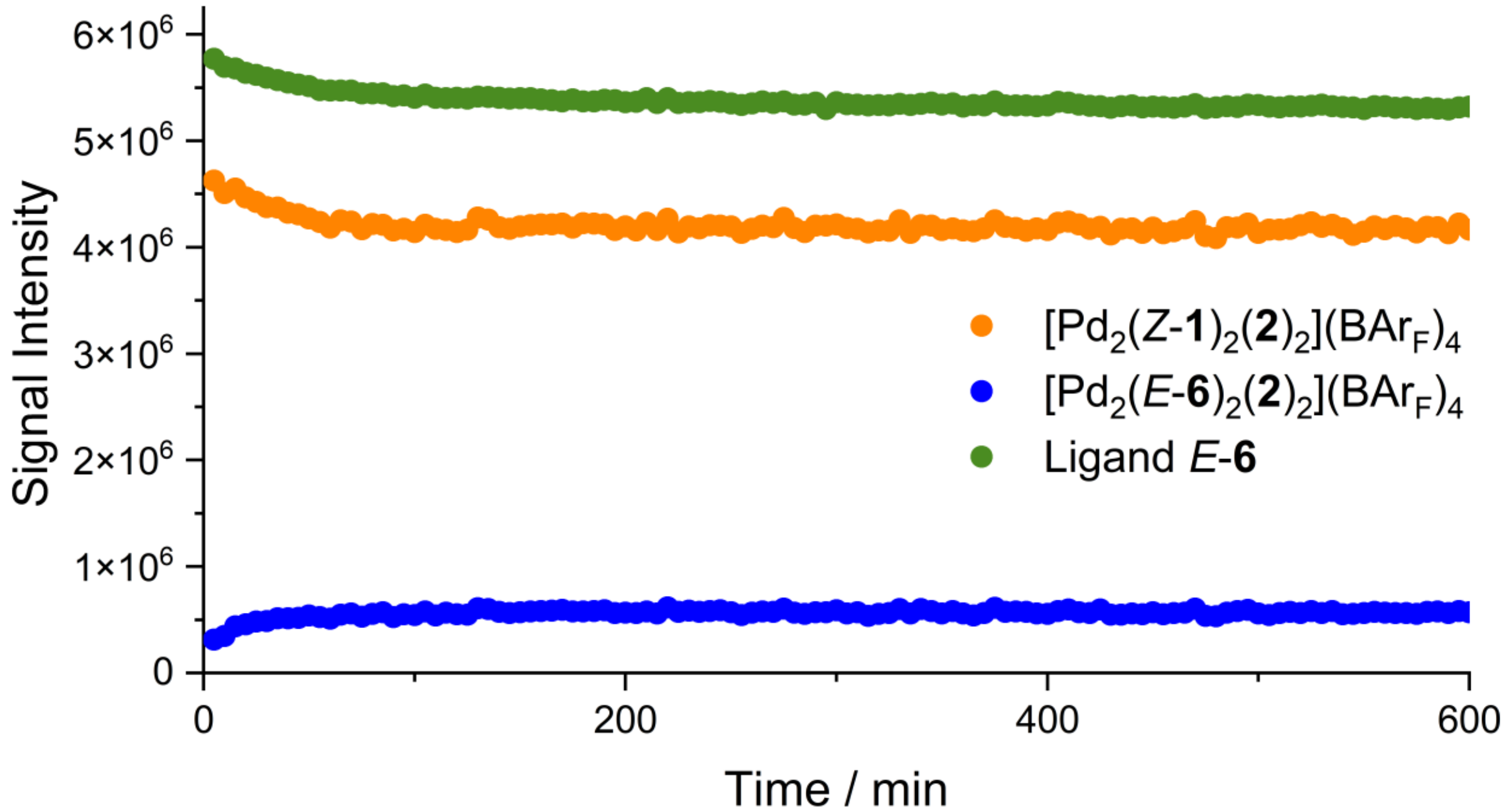

Figure S114. Monitoring the signal intensities of $[Pd_2(Z\text{-}\mathbf{1})_2(\mathbf{2})_2](BAr_F)_4$ (signal measured at 9.48 ppm), $[Pd_2(E\text{-}\mathbf{6})_2(\mathbf{2})_2](BAr_F)_4$ (signal measured at 9.30 ppm) and ligand *E*-**6** (signal measured at 8.80 ppm) over 10 hours.

7.3.2 Adding ligand *Z*-1 to [Pd_2(*E*-6)$_2$(2)$_2$](BAr$_F$)$_4$

We first prepared a sample of [Pd_2(*E*-**6**)$_2$(**2**)$_2$](BAr$_F$)$_4$ in MeCN-d_3 ([Pd] = 2.1 mM): To prepare an isomerically pure sample of ligand *E*-**6**, ligand **6** (5.9 mg, 13.0 µmol, mixture of *E*-**6** and *Z*-**6**) was dissolved in MeCN-d_3 (3.0 mL) in a sealed microwave vial ([ligand **6**] = 4.3 mM). The mixture was then heated to 130 °C for 3 hours to ensure all possible *Z*-**6** isomer back-isomerized to the *E*-**6** isomer. Ligand *E*-**6** solution (250 µL, 1.1 µmol, 4.3 mM, 1.0 equiv.) was then mixed with a solution of non-switchable ligand **2** (250 µL, 1.1 µmol. 4.3 mM, 1.0 equiv.). [Pd(Py*)$_4$](BAr$_F$)$_2$ stock solution (36 µL, 1.1 µmol, 29.7 mM, 1.0 equiv.) was added to the mixture of ligands *E*-**6** and **2** to afford [Pd_2(*E*-**6**)$_2$(**2**)$_2$](BAr$_F$)$_4$ ([Pd] = 2.1 mM) and free Py*.

[Pd_2(*E*-**6**)$_2$(**2**)$_2$](BAr$_F$)$_4$ in MeCN-d_3 was then transferred to a 4 mL vial. The sample was heated to 50 °C under a flow of argon to remove all the solvent. The residue was then redissolved in MeCN-d_3 (300 µL) to afford a sample of [Pd_2(*E*-**6**)$_2$(**2**)$_2$](BAr$_F$)$_4$ ([Pd] = 3.5 mM, 300 µL, 1.0 equiv., MeCN-d_3) for catalysis (Figure S 115i, a more concentrated sample was prepared in order to keep the final concentrations constant). Then a solution of ligand *Z*-**1** (250 µL, 1.1 µmol, 4.3 mM, 1.0 equiv., ligand *Z*-**1** was obtained by irradiating a solution of *E*-**1** with 530 nm light for 10 minutes) was added (Figure S 115ii). The solution was mixed well and the rearrangement of the self-assembled species was monitored using ^{1}H NMR spectroscopy over 10 hours (recording a spectrum every 5 minutes).

Immediately after the addition of ligand *Z*-**1** a ^{1}H NMR was recorded and the fast rearrangement of the system to disassemble [Pd_2(*E*-**6**)$_2$(**2**)$_2$](BAr$_F$)$_4$ and to form [Pd_2(*Z*-**1**)$_2$(**2**)$_2$](BAr$_F$)$_4$ was observed. The rearrangement of the system was still monitored for 10 hours. After ten hours we could observe that [Pd_2(*Z*-**1**)$_2$(**2**)$_2$](BAr$_F$)$_4$ and free ligand *E*-**6** were the major species and minor amounts of [Pd_2(*E*-**6**)$_2$(**2**)$_2$](BAr$_F$)$_4$ and trileptic cage [Pd_2(*Z*-**1**)(*E*-**6**)(**2**)$_2$](BAr$_F$)$_4$ were observed (Figure S 115iii). The distribution of the different cages and free ligand *E*-**6** (~90%) is identical to the experiment performed in the section before (7.3.1). This indicates that metastable cage [Pd_2(*Z*-**1**)$_2$(**2**)$_2$](BAr$_F$)$_4$ is preferred when all three ligands *Z*-**1**, *E*-**6** and 2 are present in a mixture with Pd(II).

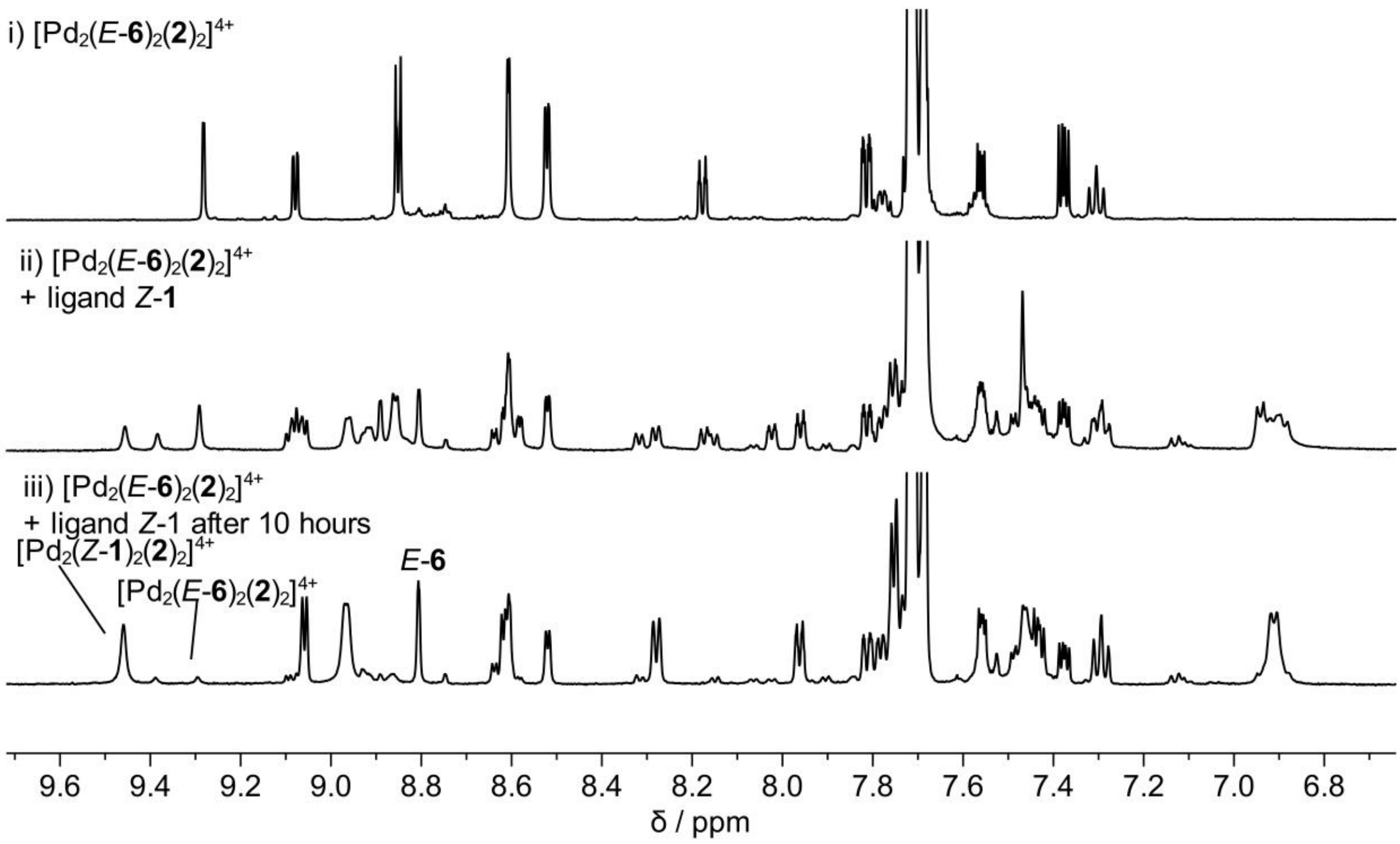

Figure S 115. Partial ^{1}H NMR spectra (600 MHz, 298 K, MeCN-d_3) of i) [Pd_2(*E*-**6**)$_2$(**2**)$_2$](BAr$_F$)$_4$ ii) the same sample immediately after the addition of ligand *Z*-**1** stock solution, and iii) the previous sample after 10 hours at room temperature.

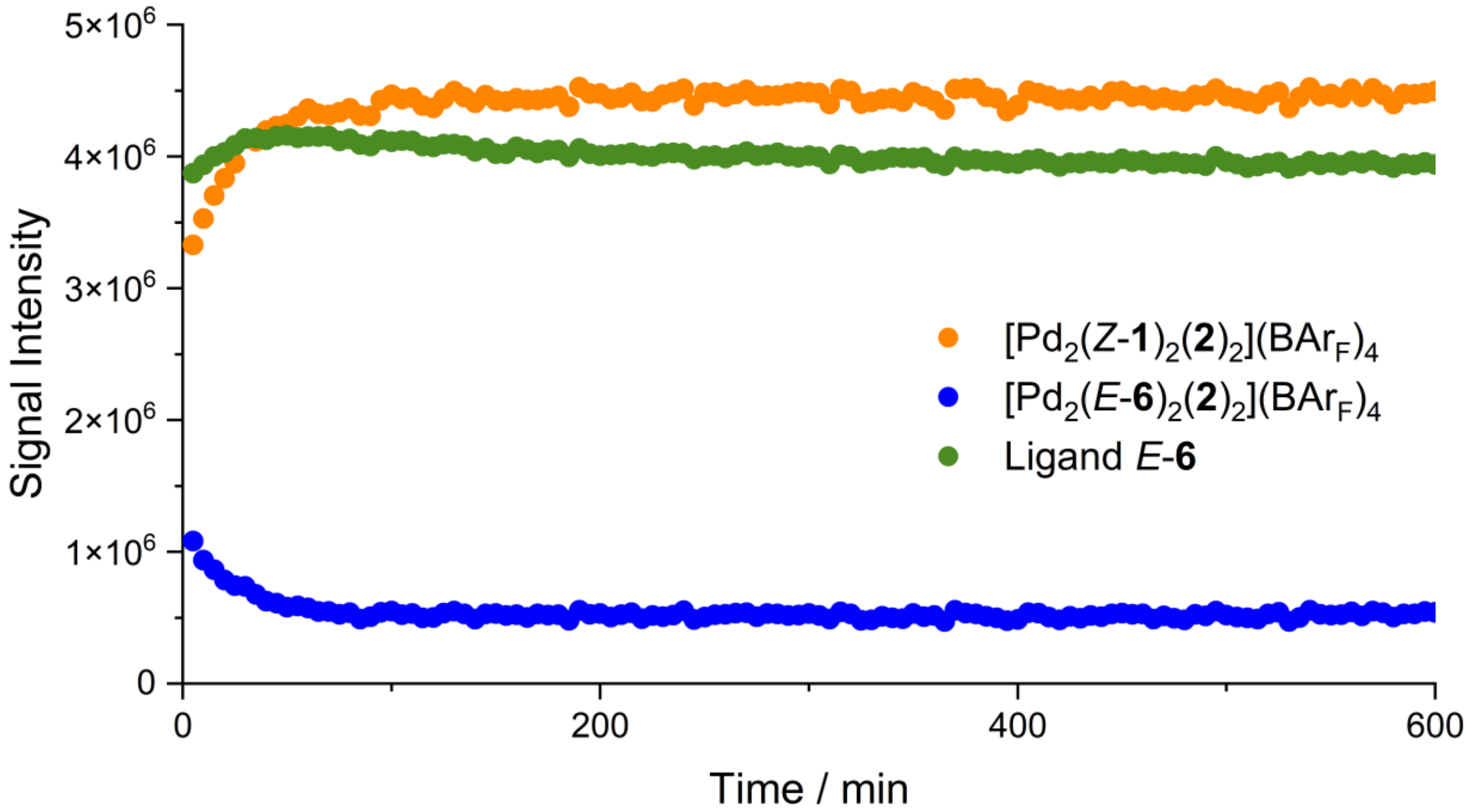

Figure S116. Monitoring the signal intensities of $[Pd_2(Z\text{-}\mathbf{1})_2(\mathbf{2})_2](BAr_F)_4$ (signal measured at 9.48 ppm), $[Pd_2(E\text{-}\mathbf{6})_2(\mathbf{2})_2](BAr_F)_4$ (signal measured at 9.30 ppm) and ligand *E*-**6** (signal measured at 8.80 ppm) over 10 hours.

8 Catalysis experiments - general

8.1 Preparation of self-assembled species for catalysis

8.2 Preparation of a $[Pd_3(E\text{-}1)_6](BAr_F)_6$ sample

$[Pd_3(E\text{-}1)_6](BAr_F)_6$ sample in MeCN-d_3

To prepare an isomerically pure sample of ligand *E*-**1**, ligand **1** (7.9 mg, 12.9 µmol, mixture of *E*-**1** and *Z*-**1**) was dissolved in MeCN-d_3 (3.0 mL) in a sealed microwave vial ([ligand **1**] = 4.3 mM). The mixture was then heated to 130 °C for 3 hours to ensure all possible *Z*-**1** isomer back-isomerized to the *E*-**1** isomer. $[Pd(Py^*)_4](BAr_F)_2$ stock solution (36 µL, 1.1 µmol, 29.7 mM, 1.0 equiv.) was added to 500 µL of this ligand *E*-**1** solution (500 µL, 2.2 µmol, 4.3 mM, 2.0 equiv.). The sample was left to equilibrate in a sand bath at 50 °C for one hour to afford a sample of the double-walled triangle cage $[Pd_3(E\text{-}\mathbf{1})_6](BAr_F)_6$ ([Pd] = 2.1 mM) and free Py*.

For catalysis that required MeCN-d_3:DCM-d_2 1:9 as a solvent mixture:

The double-walled triangle cage $[Pd_3(E\text{-}\mathbf{1})_6](BAr_F)_6$ in MeCN-d_3 was transferred to a 4 mL vial. The sample was heated to 50 °C under a flow of argon to remove all the solvent. The residue was then redissolved in MeCN-d_3 (50 µL) and DCM-d_2 (450 µL) to afford a mixture of $[Pd_3(E\text{-}\mathbf{1})_6](BAr_F)_6$ and $[Pd_4(E\text{-}\mathbf{1})_8](BAr_F)_8$ ([Pd] = 2.1 mM, 500 µL, 1.0 equiv., MeCN-d_3:DCM-d_2 1:9) for catalysis. Note that in the chosen solvent, MeCN-d_3:DCM-d_2 1:9 there is a mixture of $[Pd_3(E\text{-}\mathbf{1})_6](BAr_F)_6$ and $[Pd_4(E\text{-}\mathbf{1})_8](BAr_F)_8$ present while there is only $[Pd_3(E\text{-}\mathbf{1})_6](BAr_F)_6$ present in pure MeCN-d_3.

For catalysis that required $MeNO_2$-d_3:DCM-d_2 1:8 as a solvent mixture:

The double-walled triangle cage $[Pd_3(E\text{-}\mathbf{1})_6](BAr_F)_6$ in MeCN-d_3 was transferred to a 4 mL vial. The sample was heated to 50 °C under a flow of argon to remove all the solvent. The residue was then redissolved in $MeNO_2$-d_3 (70 µL) and DCM-d_2 (500 µL) to afford a mixture of $[Pd_3(E\text{-}\mathbf{1})_6](BAr_F)_6$ and $[Pd_4(E\text{-}\mathbf{1})_8](BAr_F)_8$ ([Pd] = 1.8 mM, 570 µL, 1.0 equiv., $MeNO_2$-d_3:DCM-d_2 1:8) for catalysis. Note that in the chosen solvent, $MeNO_2$-d_3:DCM-d_2 1:8 there is a mixture of $[Pd_3(E\text{-}\mathbf{1})_6](BAr_F)_6$ and $[Pd_4(E\text{-}\mathbf{1})_8](BAr_F)_8$ present while there is only $[Pd_3(E\text{-}\mathbf{1})_6](BAr_F)_6$ present in pure MeCN-d_3.

8.3 Preparation of a $[Pd_3(\mathbf{2})_6](BAr_F)_6$ sample

Ligand **2** (2.0 mg, 7.1 μmol) was dissolved in MeCN-d_3 (1.6 mL, [ligand **2**] = 4.4 mM). $[Pd(Py^*)_4](BAr_F)_2$ stock solution (37 μL, 1.1 μmol, 29.7 mM, 1.0 equiv.) was added to 500 μL of this ligand **2** solution (500 μL, 2.2 μmol, 4.4 mM, 2.0 equiv.). The sample was left to equilibrate in a sand bath at 50 °C for one hour to afford a sample of the double-walled triangle cage $[Pd_3(\mathbf{2})_6](BAr_F)_6$ ([Pd] = 2.1 mM) and free Py*.

For catalysis that required MeCN-*d*$_3$:DCM-*d*$_2$ 1:9 as a solvent mixture:

The double-walled triangle cage $[Pd_3(\mathbf{2})_6](BAr_F)_6$ solution in MeCN-d_3 was then transferred to a 4 mL vial. The sample was heated to 50 °C under a flow of argon to remove all the solvent. The residue was then redissolved in MeCN-d_3 (50 μL) and DCM-d_2 (450 μL) to afford $[Pd_3(\mathbf{2})_6](BAr_F)_6$ ([Pd] = 2.1 mM, 500 μL, 1.0 equiv., MeCN-d_3:DCM-d_2 1:9) for catalysis.

For catalysis that required MeNO$_2$-*d*$_3$:DCM-*d*$_2$ 1:8 as a solvent mixture:

The double-walled triangle cage $[Pd_3(\mathbf{2})_6](BAr_F)_6$ solution in MeCN-d_3 was then transferred to a 4 mL vial. The sample was heated to 50 °C under a flow of argon to remove all the solvent. The residue was then redissolved in $MeNO_2$-d_3 (70 μL) and DCM-d_2 (500 μL) to afford $[Pd_3(\mathbf{2})_6](BAr_F)_6$ ([Pd] = 1.8 mM, 570 μL, 1.0 equiv., $MeNO_2$-d_3:DCM-d_2 1:8) for catalysis.

8.4 Preparation of a mixture of $[Pd_3(E\text{-}\mathbf{1})_6](BAr_F)_6$, $[Pd_3(\mathbf{2})_6](BAr_F)_6$, $[Pd_3(E\text{-}\mathbf{1})_4(\mathbf{2})_2](BAr_F)_6$ and $[Pd_4(E\text{-}\mathbf{1})_4(\mathbf{2})_4](BAr_F)_8$ sample

To prepare an isomerically pure sample of ligand *E*-**1**, ligand **1** (7.9 mg, 12.9 μmol, mixture of *E*-**1** and *Z*-**1**) was dissolved in MeCN-d_3 (3.0 mL) in a sealed microwave vial ([ligand **1**] = 4.3 mM). The mixture was then heated to 130 °C for 3 hours to ensure all possible *Z*-**1** isomer back-isomerized to the *E*-**1** isomer.

Ligand *E*-**1** solution (250 μL, 1.1 μmol, 4.3 mM, 1.0 equiv.) was then mixed with a solution of non-switchable ligand **2** (250 μL, 1.1 μmol. 4.3 mM, 1.0 equiv.). $[Pd(Py^*)_4](BAr_F)_2$ stock solution (36 μL, 1.1 μmol, 29.7 mM, 1.0 equiv.) was added to the mixture of ligands *E*-**1** and **2**. The sample was left to equilibrate in a sand bath at 50 °C for two hours to afford a mixture of $[Pd_3(E\text{-}\mathbf{1})_6](BAr_F)_6$, $[Pd_3(\mathbf{2})_6](BAr_F)_6$, $[Pd_3(E\text{-}\mathbf{1})_4(\mathbf{2})_2](BAr_F)_6$ and $[Pd_4(E\text{-}\mathbf{1})_4(\mathbf{2})_4](BAr_F)_8$ (total [Pd] = 2.1 mM) and free Py*.

For catalysis that required MeCN-*d*$_3$:DCM-*d*$_2$ 1:9 as a solvent mixture:

The cage mixture in MeCN-d_3 was then transferred to a 4 mL vial. The sample was heated to 50 °C under a flow of argon to remove all the solvent. The residue was then redissolved in MeCN-d_3 (50 μL) and DCM-d_2 (450 μL) to afford a mixture of $[Pd_3(E\text{-}\mathbf{1})_6](BAr_F)_6$, $[Pd_3(\mathbf{2})_6](BAr_F)_6$, $[Pd_3(E\text{-}\mathbf{1})_4(\mathbf{2})_2](BAr_F)_6$ and $[Pd_4(E\text{-}\mathbf{1})_4(\mathbf{2})_4](BAr_F)_8$ ([Pd] = 2.1 mM, 500 μL, 1.0 equiv., MeCN-d_3:DCM-d_2 1:9) for catalysis.

For catalysis that required MeNO$_2$-*d*$_3$:DCM-*d*$_2$ 1:8 as a solvent mixture:

The cage mixture in MeCN-d_3 was then transferred to a 4 mL vial. The sample was heated to 50 °C under a flow of argon to remove all the solvent. The residue was then redissolved in $MeNO_2$-d_3 (70 μL) and DCM-d_2 (500 μL) to afford a mixture of $[Pd_3(E\text{-}\mathbf{1})_6](BAr_F)_6$, $[Pd_3(\mathbf{2})_6](BAr_F)_6$, $[Pd_3(E\text{-}\mathbf{1})_4(\mathbf{2})_2](BAr_F)_6$ and $[Pd_4(E\text{-}\mathbf{1})_4(\mathbf{2})_4](BAr_F)_8$ ([Pd] = 1.8 mM, 570 μL, 1.0 equiv., $MeNO_2$-d_3:DCM-d_2 1:8) for catalysis.

For switchable catalysis that required MeNO$_2$-*d*$_3$:MeCN-*d*$_3$:DCM-*d*$_2$ 1.4:1:9 as a solvent mixture:

The cage mixture in MeCN-d_3 was then transferred to a 4 mL vial. The sample was heated to 50 °C under a flow of argon to remove all the solvent. The residue was then redissolved in $MeNO_2$-d_3 (70 μL), MeCN-d_3 (50 μL) and DCM-d_2 (450 μL) to afford a mixture of $[Pd_3(E\text{-}\mathbf{1})_6](BAr_F)_6$, $[Pd_3(\mathbf{2})_6](BAr_F)_6$, $[Pd_3(E\text{-}\mathbf{1})_4(\mathbf{2})_2](BAr_F)_6$ and [Pd$_4$(*E*-

1)$_4$(**2**)$_4$](BAr$_F$)$_8$ ([Pd] = 1.8 mM, 570 µL, 1.0 equiv., MeNO$_2$-*d*$_3$: MeCN-*d*$_3$:DCM-*d*$_2$ 1.4:1:9) for catalysis.

8.5 Preparation of a [Pd$_2$(*Z*-1)$_2$(2)$_2$](BAr$_F$)$_4$ sample

To prepare an isomerically pure sample of ligand *E*-**1**, ligand **1** (7.9 mg, 12.9 µmol, mixture of *E*-**1** and *Z*-**1**) was dissolved in MeCN-*d*$_3$ (3.0 mL) in a sealed microwave vial ([ligand **1**] = 4.3 mM). The mixture was then heated to 130 °C for 3 hours to ensure all possible *Z*-**1** isomer back-isomerized to the *E*-**1** isomer.

Ligand *E*-**1** solution (250 µL, 1.1 µmol, 4.3 mM, 1.0 equiv.) was then mixed with a solution of non-switchable ligand **2** (250 µL, 1.1 µmol. 4.3 mM, 1.0 equiv.). [Pd(Py*)$_4$](BAr$_F$)$_2$ stock solution (36 µL, 1.1 µmol, 29.7 mM, 1.0 equiv.) was added to the mixture of ligands *E*-**1** and **2.** The sample was then irradiated with 530 nm light for 15 minutes to afford [Pd$_2$(*Z*-**1**)$_2$(**2**)$_2$](BAr$_F$)$_4$ ([Pd] = 2.1 mM) and free Py*.

For catalysis that required MeCN-*d*$_3$:DCM-*d*$_2$ 1:9 as a solvent mixture:

The metastable cage [Pd$_2$(*Z*-**1**)$_2$(**2**)$_2$](BAr$_F$)$_4$ in MeCN-*d*$_3$ was then transferred to a 4 mL vial. The sample was heated to 50 °C under a flow of argon to remove all the solvent. The residue was then redissolved in MeCN-*d*$_3$ (50 µL) and DCM-*d*$_2$ (450 µL) to afford a sample of [Pd$_2$(*Z*-**1**)$_2$(**2**)$_2$](BAr$_F$)$_4$ ([Pd] = 2.1 mM, 500 µL, 1.0 equiv., MeCN-*d*$_3$:DCM-*d*$_2$ 1:9) for catalysis.

For catalysis that required MeNO$_2$-*d*$_3$:DCM-*d*$_2$ 1:8 as a solvent mixture:

The metastable cage [Pd$_2$(*Z*-**1**)$_2$(**2**)$_2$](BAr$_F$)$_4$ in MeCN-*d*$_3$ was then transferred to a 4 mL vial. The sample was heated to 50 °C under a flow of argon to remove all the solvent. The residue was then redissolved in MeNO$_2$-*d*$_3$ (70 µL) and DCM-*d*$_2$ (500 µL) to afford a sample of [Pd$_2$(*Z*-**1**)$_2$(**2**)$_2$](BAr$_F$)$_4$ ([Pd] = 1.8 mM, 570 µL, 1.0 equiv., MeNO$_2$-*d*$_3$:DCM-*d*$_2$ 1:8) for catalysis.

9 Catalysis: Michael addition between benzoyl nitromethane and methyl vinyl ketone catalysed by different self-assembled species

9.1 Preparation of stock solutions used for Michael addition catalysis samples

The following stock solutions were prepared:

Preparation of $[Pd(Py^*)_4](BAr_F)_2$ stock solution

$[Pd(Py^*)_4](BAr_F)_2$ (33.9 mg, 14.8 μmol) was dissolved in MeCN-d_3 (500 μL) to afford a stock solution of $[Pd(Py^*)_4](BAr_F)_2$ ([Pd] = 29.7 mM).

Preparation of methyl vinyl ketone stock solution

Methyl vinyl ketone (10 μL, 120 μmol) was dissolved in DCM-d_2 (190 μL) to afford a stock solution of methyl vinyl ketone (600 mM).

Preparation of benzoyl nitromethane stock solution

Benzoyl nitromethane (41.6 mg, 252 μmol) was dissolved in DCM-d_2 (500 μL) to afford a stock solution of benzoyl nitromethane (504 mM).

Preparation of 18-crown-6 stock solution

18-Crown-6 (30.5 mg, 115 μmol) was dissolved in DCM-d_2 (400 μL) to afford a stock solution of 18-crown-6 (289 mM).

9.2 Monitoring the Michael addition reaction between benzoyl nitromethane and methyl vinyl ketone using ^{1}H NMR spectroscopy

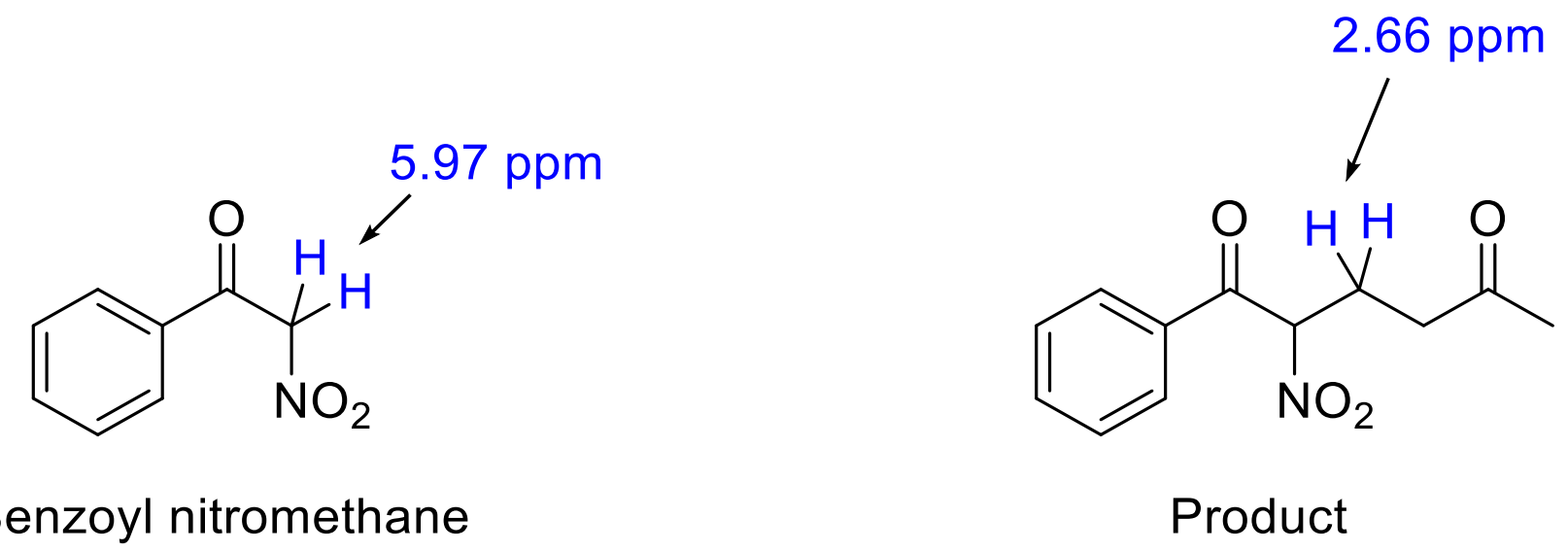

To calculate the conversion of the Michael addition, the intensity of the signal at 5.97 ppm corresponding to the benzoyl nitromethane starting material and the intensity of the signal at 2.66 ppm of the Michael addition product were measured over time.

The mol% of benzoyl nitromethane and the Michael addition product relative to each other were determined by dividing all signal intensities by the sum of the signal intensities for benzoyl nitromethane and the product at the start of the reaction (i.e. t = 0):

$$mol\% = \left(\frac{SI}{NBA_0 + P_0}\right)$$

Mol% = mol% of either benzoyl nitromethane or the Michael addition product

SI = signal intensity of either benzoyl nitromethane or the Michael addition product

NBA_0 = signal intensity of benzoyl nitromethane at t = 0

P_0 = signal intensity of the Michael addition product at t = 0

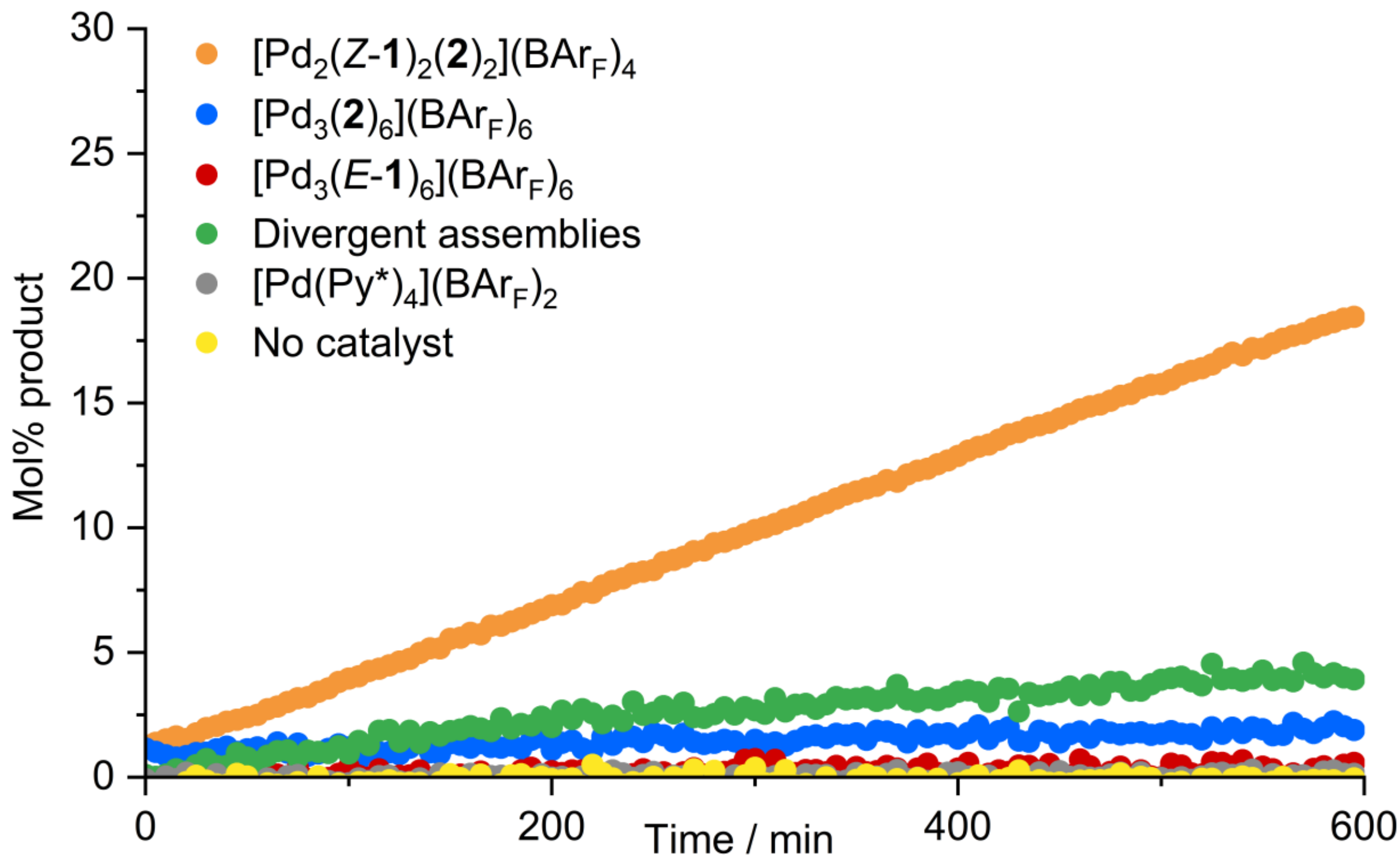

Figure S117. ^{1}H NMR (600 MHz, 298 K, DCM-d_2:MeCN-d_3 (11:1)) signal intensities of Michael addition product following the reaction between benzoyl nitromethane (17.5 mM, 9.8 mM) and methyl vinyl ketone (31.3 mM, 17.5 equiv.) in the presence of 18-crown-6 (12.5 mM, 7.0 equiv.) and different self-assembled species ([Pd] = 1.8 mM, 1.0 equiv.).

9.3 Using $[Pd_2(Z\text{-}1)_2(2)_2](BAr_F)_4$ to catalyze the Michael addition reaction between benzoyl nitromethane and methyl vinyl ketone

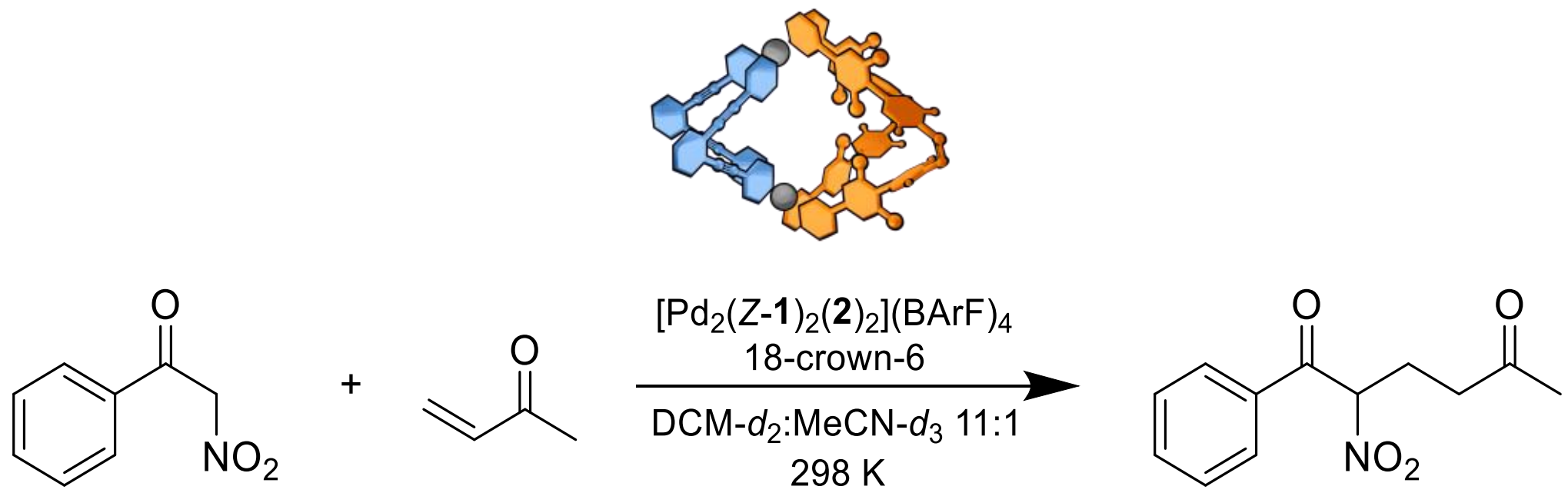

A solution of $[Pd_2(Z\text{-}\mathbf{1})_2(\mathbf{2})_2](BAr_F)_4$ ([Pd] = 2.1 mM, 500 µL, 1.0 µmol, 1.0 equiv., MeCN-d_3:DCM-d_2 1:9, preparation see section 8.5) was placed in an NMR tube. Then stock solutions of methyl vinyl ketone (30 µL, 18 µmol, 600 mM, in DCM-d_2) and benzoyl nitromethane (20 µL, 10 µmol, 504 mM, in DCM-d_2) were added. The Michael addition reaction was initiated by the addition of 18-crown-6 stock solution (25 µL, 7.2 µmol, 289 mM, in DCM-d_2). This afforded a sample containing $[Pd_2(Z\text{-}\mathbf{1})_2(\mathbf{2})_2](BAr_F)_4$ ([Pd] = 1.8 mM, 1.0 equiv.), methyl vinyl ketone (31.3 mM, 17.5 equiv.), benzoyl nitromethane (17.5 mM, 9.8 equiv.) and 18-crown-6 (12.5 mM, 7.0 equiv.) in MeCN-d_3:DCM-d_2 1:11.

The mol% of benzoyl nitromethane and the product were plot against time (see Supporting Information section S9.2 for details) to determine whether the metastable cage $[Pd_2(Z\text{-}\mathbf{1})_2(\mathbf{2})_2](BAr_F)_4$ can catalyse the Michael addition reaction between benzoyl nitromethane and methyl vinyl ketone. 18% product formation was observed after 10 hours (Figure S118) similar to previous results. [2]

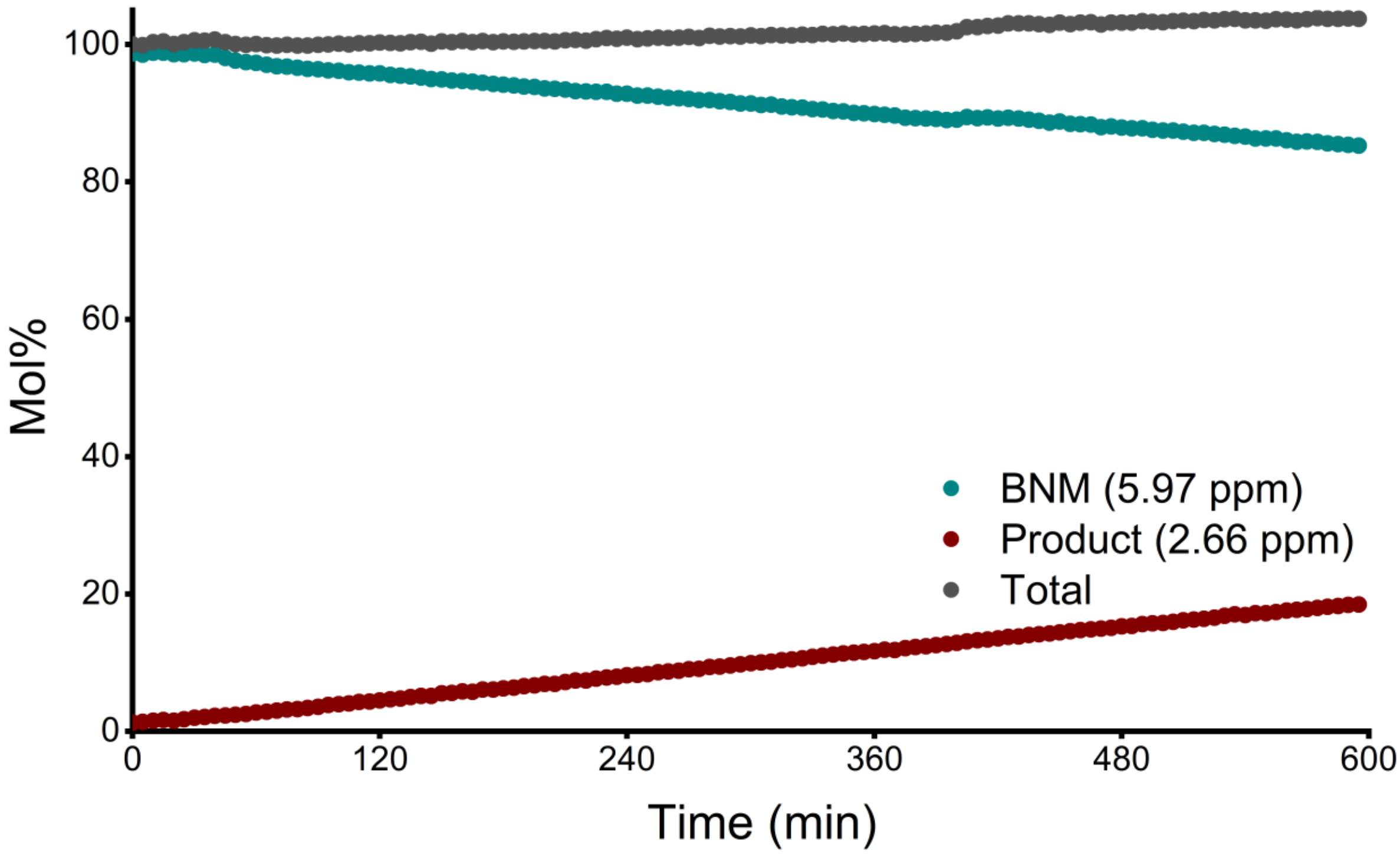

Figure S118. Monitoring catalysis within heteroleptic cage $[Pd_2(Z\text{-}\mathbf{1})_2(\mathbf{2})_2](BAr_F)_4$. ^{1}H NMR (600 MHz, 298 K, DCM-d_2:MeCN-d_3 (11:1)) signal intensities of benzoyl nitromethane and Michael addition product following the reaction between benzoyl nitromethane (17.5 mM, 9.8 equiv.) and methyl vinyl ketone (31.3 mM, 17.5 equiv.) in the presence of 18-crown-6 (12.5 mM, 7.0 equiv.) and $[Pd_2(Z\text{-}\mathbf{1})_2(\mathbf{2})_2](BAr_F)_4$ ([Pd] = 1.8 mM, 1.0 equiv.). The integral of the signal at 5.97 ppm was used to monitor the abundance of benzoyl nitromethane and the integral of the signal at 2.66 ppm was used to monitor the abundance of the Michael addition product. Relaxation delay (Bruker setting D1) was set to 5 seconds.

9.4 Using $[Pd_3(\mathbf{2})_6](BAr_F)_6$ to catalyze the Michael addition reaction between benzoyl nitromethane and methyl vinyl ketone

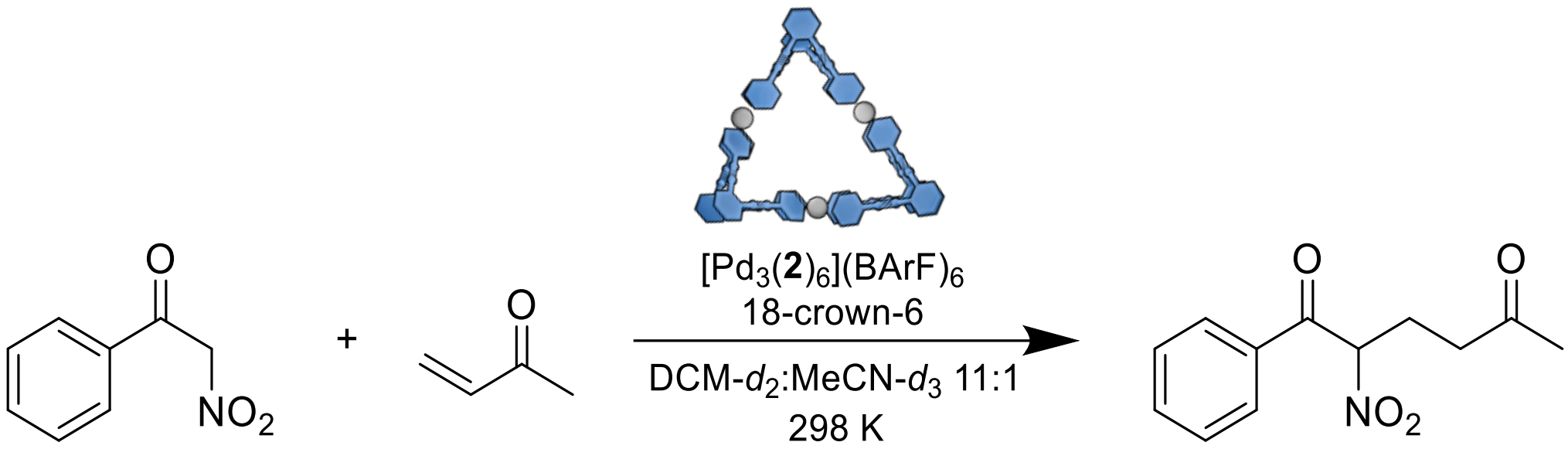

A solution of $[Pd_3(\mathbf{2})_6](BAr_F)_6$ ([Pd] = 2.1 mM, 500 µL, 1.0 µmol, 1.0 equiv., MeCN-d_3:DCM-d_2 1:9, preparation see section 8.3) was placed in an NMR tube. Then stock solutions of methyl vinyl ketone (30 µL, 18 µmol, 600 mM, in DCM-d_2) and benzoyl nitromethane (20 µL, 10 µmol, 504 mM, in DCM-d_2) were added. The Michael addition reaction was initiated by the addition of 18-crown-6 stock solution (25 µL, 7.2 µmol, 289 mM, in DCM-d_2). This afforded a sample containing $[Pd_3(\mathbf{2})_6](BAr_F)_6$ ([Pd] = 1.8 mM, 1.0 equiv.), methyl vinyl ketone (31.3 mM, 17.5 equiv), benzoyl nitromethane (17.5 mM, 9.8 equiv.) and 18-crown-6 (12.5 mM, 7.0 equiv.) in MeCN-d_3:DCM-d_2 1:11.

The mol% of benzoyl nitromethane and the product were plot against time (see Supporting Information section S9.2 for details) to determine whether the homoleptic cage $[Pd_3(\mathbf{2})_6](BAr_F)_6$ can catalyse the Michael addition reaction between benzoyl nitromethane and methyl vinyl ketone. Only ~2% product formation was observed after 10 hours (Figure S119), similar to previous results. [2]

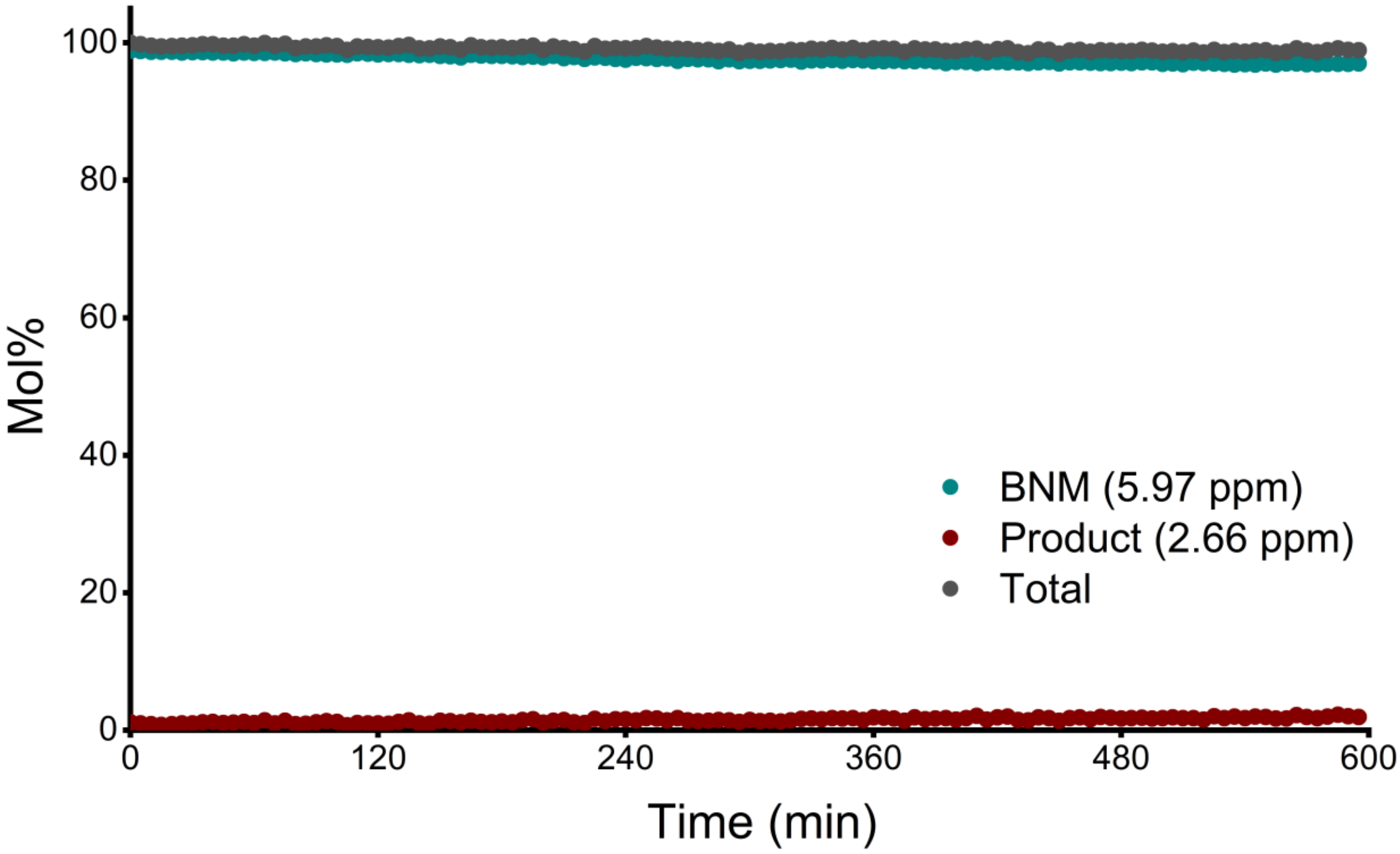

Figure S119. Monitoring catalysis within homoleptic cage $[Pd_3(\mathbf{2})_6](BAr_F)_6$. ^{1}H NMR (600 MHz, 298 K, DCM-d_2:MeCN-d_3 (11:1)) signal intensities of benzoyl nitromethane and Michael addition product following the reaction between benzoyl nitromethane (17.5 mM, 9.8 equiv.) and methyl vinyl ketone (31.3 mM, 17.5 equiv.) in the presence of 18-crown-6 (12.5 mM, 7.0 equiv.) and $[Pd_3(\mathbf{2})_6](BAr_F)_6$ ([Pd] = 1.8 mM, 1.0 equiv.). The integral of the signal at 5.97 ppm was used to monitor the abundance of benzoyl nitromethane and the integral of the signal at 2.66 ppm was used to monitor the abundance of the Michael addition product. Relaxation delay (Bruker setting D1) was set to 5 seconds.

9.5 Using a mixture of [Pd_3(*E*-1)$_6$](BAr$_F$)$_6$, [Pd_3(2)$_6$](BAr$_F$)$_6$, [Pd_3(*E*-1)$_4$(2)$_2$](BAr$_F$)$_6$ and [Pd_4(*E*-1)$_4$(2)$_4$](BAr$_F$)$_8$ to catalyze the Michael addition reaction between benzoyl nitromethane and methyl vinyl ketone

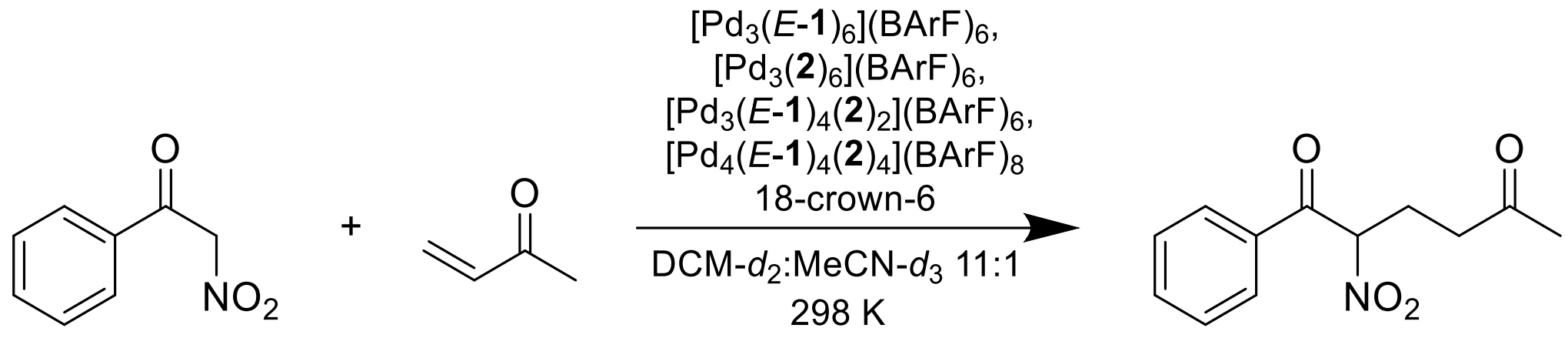

A solution of [Pd_3(*E*-**1**)$_6$](BAr$_F$)$_6$, [Pd_3(**2**)$_6$](BAr$_F$)$_6$, [Pd_3(*E*-**1**)$_4$(**2**)$_2$](BAr$_F$)$_6$ and [Pd_4(*E*-**1**)$_4$(**2**)$_4$](BAr$_F$)$_8$ (total [Pd] = 2.1 mM, 500 µL, 1.0 µmol, 1.0 equiv., MeCN-d_3:DCM-d_2 1:9, preparation see section 8.2) was placed in an NMR tube. Then stock solutions of methyl vinyl ketone (30 µL, 18 µmol, 600 mM, in DCM-d_2) and benzoyl nitromethane (20 µL, 10 µmol, 504 mM, in DCM-d_2) were added. The Michael addition reaction was initiated by the addition of 18-crown-6 stock solution (25 µL, 7.2 µmol, 289 mM, in DCM-d_2). This afforded a sample containing [Pd_3(*E*-**1**)$_6$](BAr$_F$)$_6$, [Pd_3(**2**)$_6$](BAr$_F$)$_6$, [Pd_3(*E*-**1**)$_4$(**2**)$_2$](BAr$_F$)$_6$ and [Pd_4(*E*-**1**)$_4$(**2**)$_4$](BAr$_F$)$_8$ (total [Pd] = 1.8 mM, 1.0 equiv.), methyl vinyl ketone (31.3 mM, 17.5 equiv), benzoyl nitromethane (17.5 mM, 9.8 equiv.) and 18-crown-6 (12.5 mM, 7.0 equiv.) in MeCN-d_3:DCM-d_2 1:11.

The mol% of benzoyl nitromethane and the product were plot against time (see Supporting Information section S9.2 for details) to determine whether the mixture of homo- / heterolelptic cages with ligand *E*-**1** can catalyse the Michael addition reaction between benzoyl nitromethane and methyl vinyl ketone. After 10 hours, 4% product was observed (Figure S120).

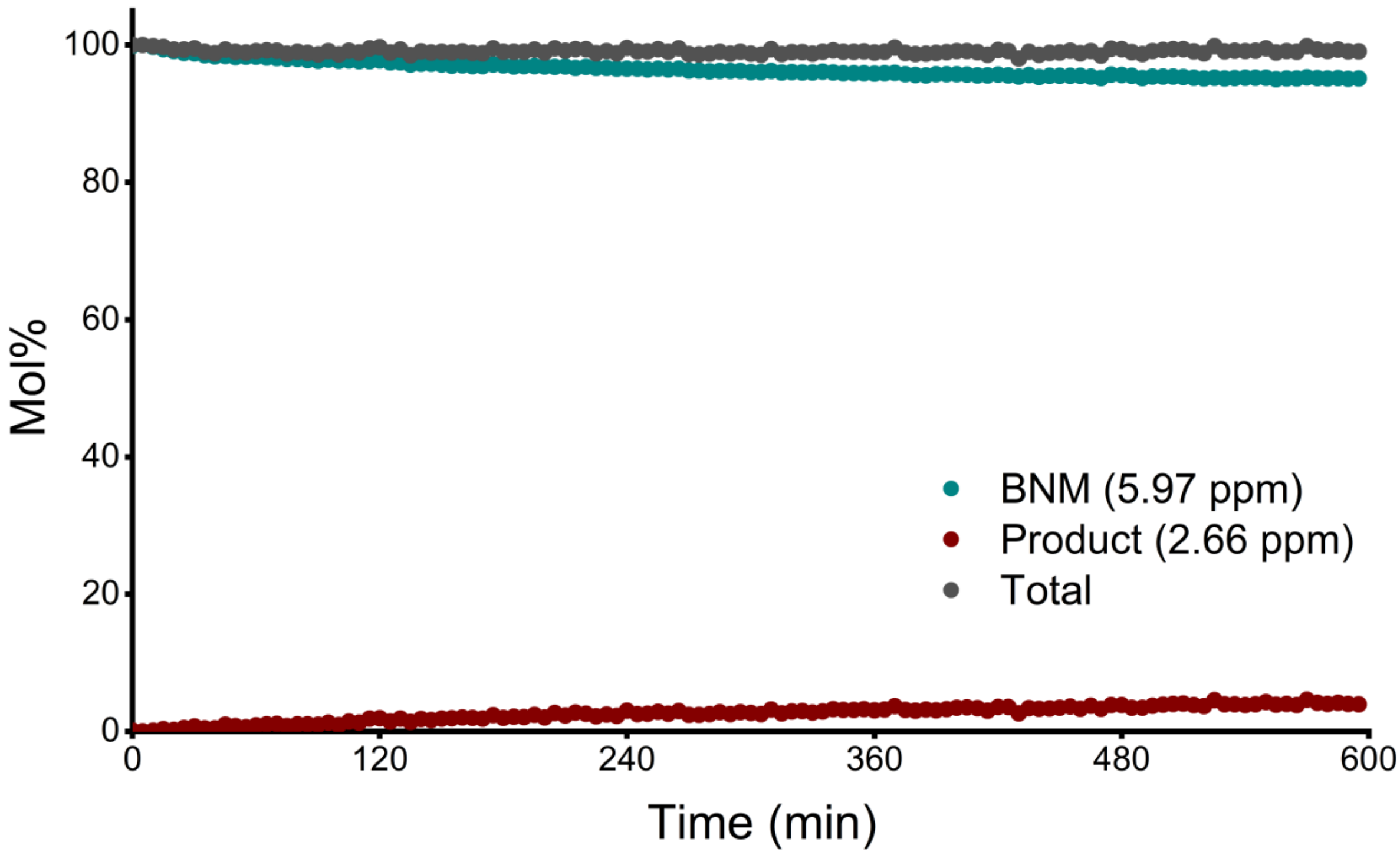

Figure S120. Monitoring catalysis within a mixture of [Pd_3(*E*-**1**)$_6$](BAr$_F$)$_6$, [Pd_3(**2**)$_6$](BAr$_F$)$_6$, [Pd_3(*E*-**1**)$_4$(**2**)$_2$](BAr$_F$)$_6$ and [Pd_4(*E*-**1**)$_4$(**2**)$_4$](BAr$_F$)$_8$. ^{1}H NMR (600 MHz, 298 K, DCM-d_2:MeCN-d_3 (11:1)) signal intensities of benzoyl nitromethane and Michael addition product following the reaction between benzoyl nitromethane (17.5 mM, 9.8 equiv.) and methyl vinyl ketone (31.3 mM, 17.5 equiv.) in the presence of 18-crown-6 (12.5 mM, 7.0 equiv.) and a mixture of [Pd_3(*E*-**1**)$_6$](BAr$_F$)$_6$, [Pd_3(**2**)$_6$](BAr$_F$)$_6$, [Pd_3(*E*-**1**)$_4$(**2**)$_2$](BAr$_F$)$_6$ and [Pd_4(*E*-**1**)$_4$(**2**)$_4$](BAr$_F$)$_8$ ([Pd] = 1.8 mM, 1.0 equiv.). The integral of the signal at 5.97 ppm was used to monitor the abundance of benzoyl nitromethane and the integral of the signal at 2.66 ppm was used to monitor the abundance of the Michael addition product. Relaxation delay (Bruker setting D1) was set to 5 seconds.

9.6 Using $[Pd_3(E\text{-}1)_6](BArF)_6$ / $[Pd_4(E\text{-}1)_8](BArF)_8$ to catalyze the Michael addition reaction between benzoyl nitromethane and methyl vinyl ketone

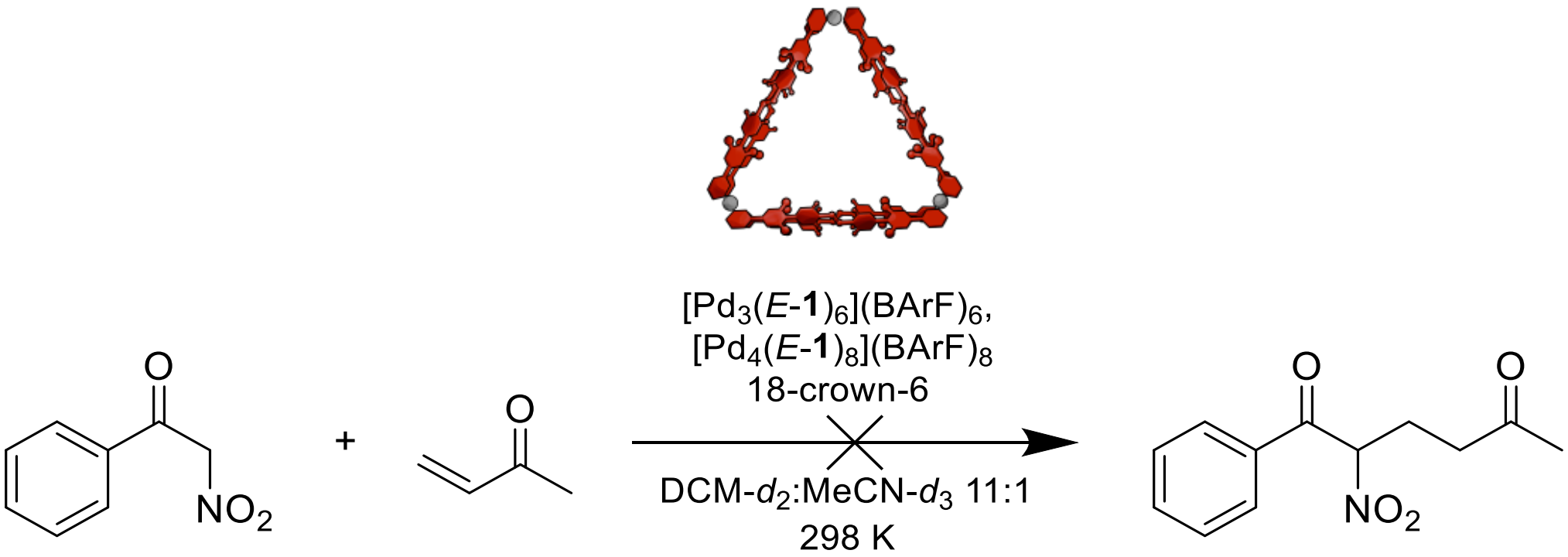

A solution of $[Pd_3(E\text{-}\mathbf{1})_6](BAr_F)_6$ / $[Pd_4(E\text{-}\mathbf{1})_8](BAr_F)_8$ (total [Pd] = 2.1 mM, 500 µL, 1.0 µmol, 1.0 equiv., MeCN-d_3:DCM-d_2 1:9, preparation see section 8.2) was placed in an NMR tube. Then stock solutions of methyl vinyl ketone (30 µL, 18 µmol, 600 mM, in DCM-d_2) and benzoyl nitromethane (20 µL, 10 µmol, 504 mM, in DCM-d_2) were added. The Michael addition reaction was initiated by the addition of 18-crown-6 stock solution (25 µL, 7.2 µmol, 289 mM, in DCM-d_2). This afforded a sample containing $[Pd_3(E\text{-}\mathbf{1})_6](BAr_F)_6$ / $[Pd_4(E\text{-}\mathbf{1})_8](BAr_F)_8$ (total [Pd] = 1.8 mM, 1.0 equiv.), methyl vinyl ketone (31.3 mM, 17.5 equiv), benzoyl nitromethane (17.5 mM, 9.8 equiv.) and 18-crown-6 (12.5 mM, 7.0 equiv.) in MeCN-d_3:DCM-d_2 1:11.

The mol% of benzoyl nitromethane and the product were plot against time (see Supporting Information section S9.2 for details) to determine whether the homoleptic cages $[Pd_3(E\text{-}\mathbf{1})_6](BAr_F)_6$ / $[Pd_4(E\text{-}\mathbf{1})_8](BAr_F)_8$ can catalyse the Michael addition reaction between benzoyl nitromethane and methyl vinyl ketone. No product formation was observed after 10 hours (Figure S121).

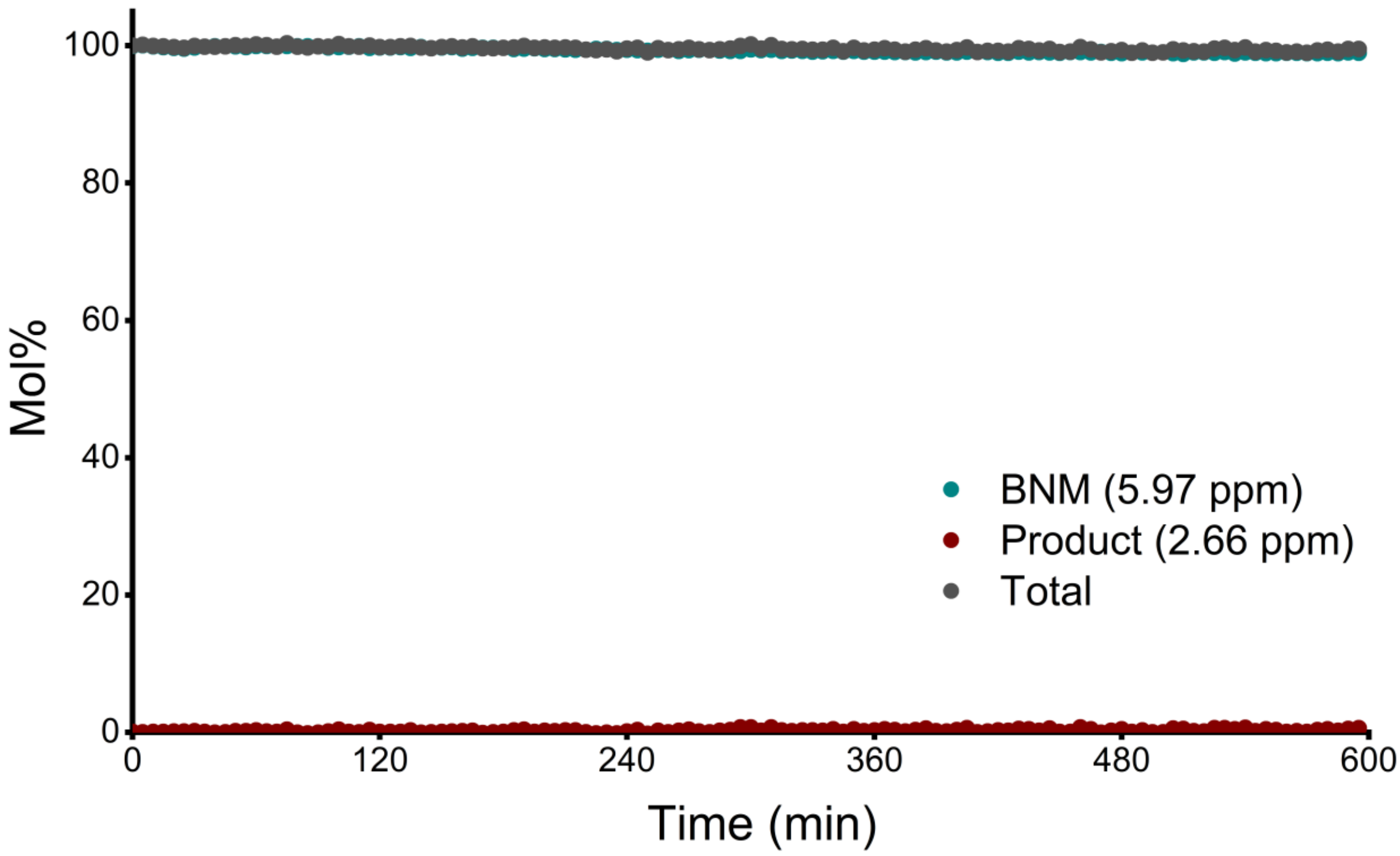

Figure S121. Monitoring catalysis within homoleptic cages $[Pd_3(E\text{-}\mathbf{1})_6](BAr_F)_6$ and $[Pd_4(E\text{-}\mathbf{1})_8](BAr_F)_8$. ^{1}H NMR (600 MHz, 298 K, DCM-d_2:MeCN-d_3 (11:1)) signal intensities of benzoyl nitromethane and Michael addition product following the reaction between benzoyl nitromethane (17.5 mM, 9.8 equiv.) and methyl vinyl ketone (31.3 mM, 17.5 equiv.) in the presence of 18-crown-6 (12.5 mM, 7.0 equiv.) and $[Pd_3(E\text{-}\mathbf{1})_6](BAr_F)_6$ and $[Pd_4(E\text{-}\mathbf{1})_8](BAr_F)_8$ ([Pd] = 1.8 mM, 1.0 equiv.). The integral of the signal at 5.97 ppm was used to monitor the abundance of benzoyl nitromethane and the integral of the signal at 2.66 ppm was used to monitor the abundance of the Michael addition product. Relaxation delay (Bruker setting D1) was set to 5 seconds.

9.7 Monitoring the Michael addition reaction between benzoyl nitromethane and methyl vinyl ketone using $[Pd(Py^*)_4](BAr_F)_2$

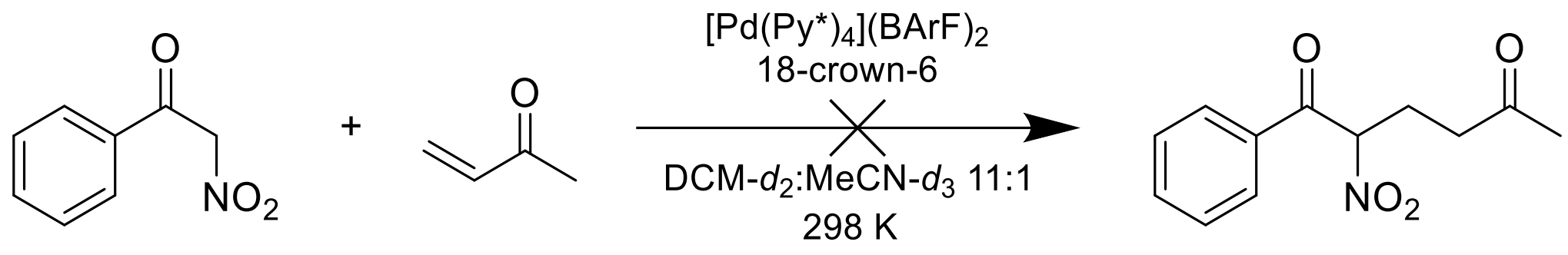

A solution of $[Pd(Py^*)_4](BAr_F)_2$ ([Pd] = 29.7 mM, 36 μL, 1.1 μmol, 1.0 equiv., MeCN-d_3) was placed in an NMR tube. DCM-d_2 (450 μL) and MeCN-d_3 (14 μL) were added. Then stock solutions of methyl vinyl ketone (30 μL, 18 μmol, 600 mM, in DCM-d_2) and benzoyl nitromethane (20 μL, 10 μmol, 504 mM, in DCM-d_2) were added. The 18-crown-6 stock solution (25 μL, 7.2 μmol, 289 mM, in DCM-d_2) was added last. This afforded a sample containing $[Pd(Py^*)_4](BAr_F)_2$ ([Pd] = 1.9 mM, 1.0 equiv.), methyl vinyl ketone (31.3 mM, 17.5 equiv), benzoyl nitromethane (17.5 mM, 9.8 equiv.) and 18-crown-6 (12.5 mM, 7.0 equiv.) in MeCN-d_3:DCM-d_2 1:11.

The mol% of benzoyl nitromethane and the product were plot against time (see Supporting Information section S9.2 for details) to determine whether $[Pd(Py^*)_4](BAr_F)_2$ can catalyse the Michael addition reaction between benzoyl nitromethane and methyl vinyl ketone. No product formation was observed after 10 hours, demonstrating that palladium(**II**) was not responsible for the catalysis observed in the sample containing $[Pd_2(Z\text{-}\mathbf{1})_2(\mathbf{2})_2](BAr_F)_4$ (Figure S122).

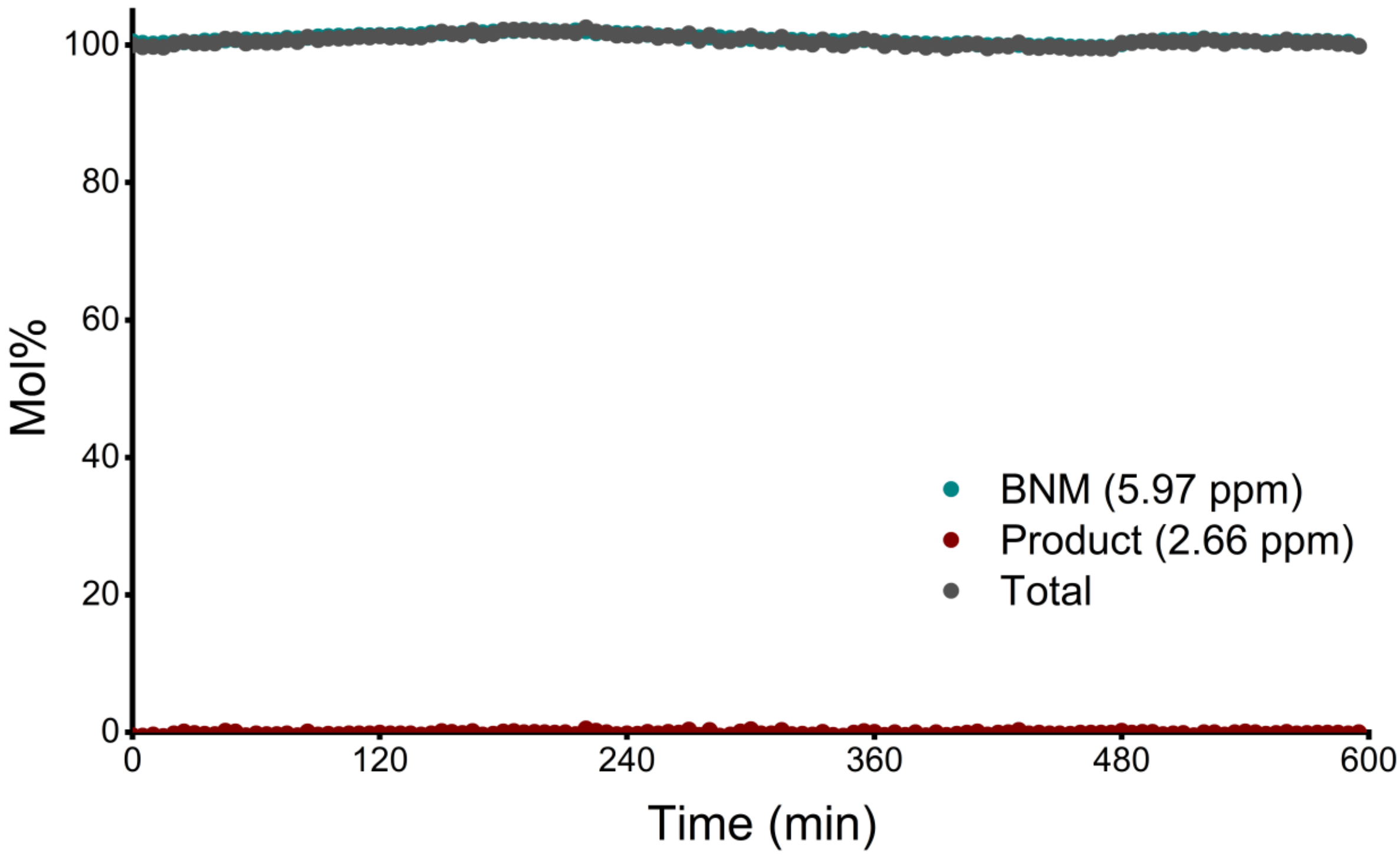

Figure S122. Monitoring catalysis using $[Pd(Py^*)_4](BAr_F)_2$. ^1H NMR (600 MHz, 298 K, DCM-d_2:MeCN-d_3 (11:1)) signal intensities of benzoyl nitromethane and Michael addition product following the reaction between benzoyl nitromethane (17.5 mM, 9.8 equiv.) and methyl vinyl ketone (31.3 mM, 17.5 equiv.) in the presence of 18-crown-6 (12.5 mM, 7.0 equiv.) and $[Pd(Py^*)_4](BAr_F)_2$ ([Pd] = 1.8 mM, 1.0 equiv.). The integral of the signal at 5.97 ppm was used to monitor the abundance of benzoyl nitromethane and the integral of the signal at 2.66 ppm was used to monitor the abundance of the Michael addition product. Relaxation delay (Bruker setting D1) was set to 5 seconds.

9.8 Monitoring the Michael addition reaction between benzoyl nitromethane and methyl vinyl ketone using no catalyst

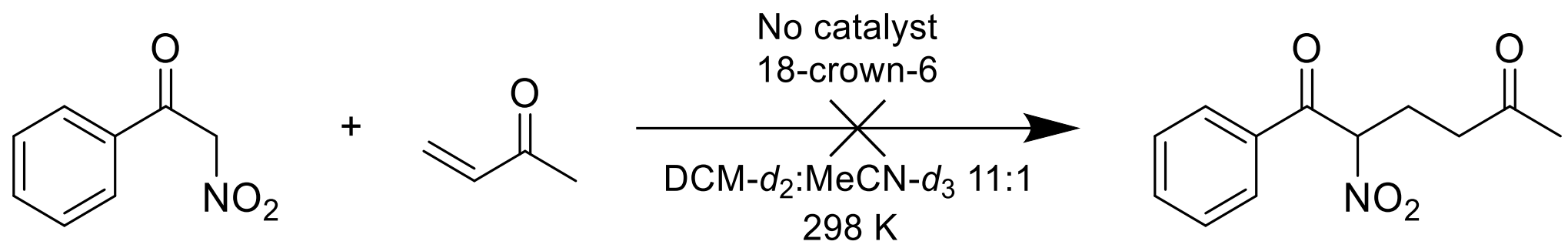

An NMR tube was charged with DCM-d_2 (450 μL) and MeCN-d_3 (50 μL). Then stock solutions of methyl vinyl ketone (30 μL, 18 μmol, 600 mM, in DCM-d_2) and benzoyl nitromethane (20 μL, 10 μmol, 504 mM, in DCM-d_2) were added. The 18-crown-6 stock solution (25 μL, 7.2 μmol, 289 mM, in DCM-d_2) was added last. This afforded a sample containing methyl vinyl ketone (31.3 mM, 17.5 equiv), benzoyl nitromethane (17.5 mM, 9.8 equiv.) and 18-crown-6 (12.5 mM, 7.0 equiv.) in MeCN-d_3:DCM-d_2 1:11.

The mol% of benzoyl nitromethane and the product were plot against time (see Supporting Information section S9.2 for details) to determine whether $[Pd(Py^*)_4](BAr_F)_2$ can catalyse the Michael addition reaction between benzoyl nitromethane and methyl vinyl ketone. No product formation was observed after 10 hours, demonstrating that there is no background reaction between benzoyl nitromethane and methyl vinyl ketone (Figure S123).

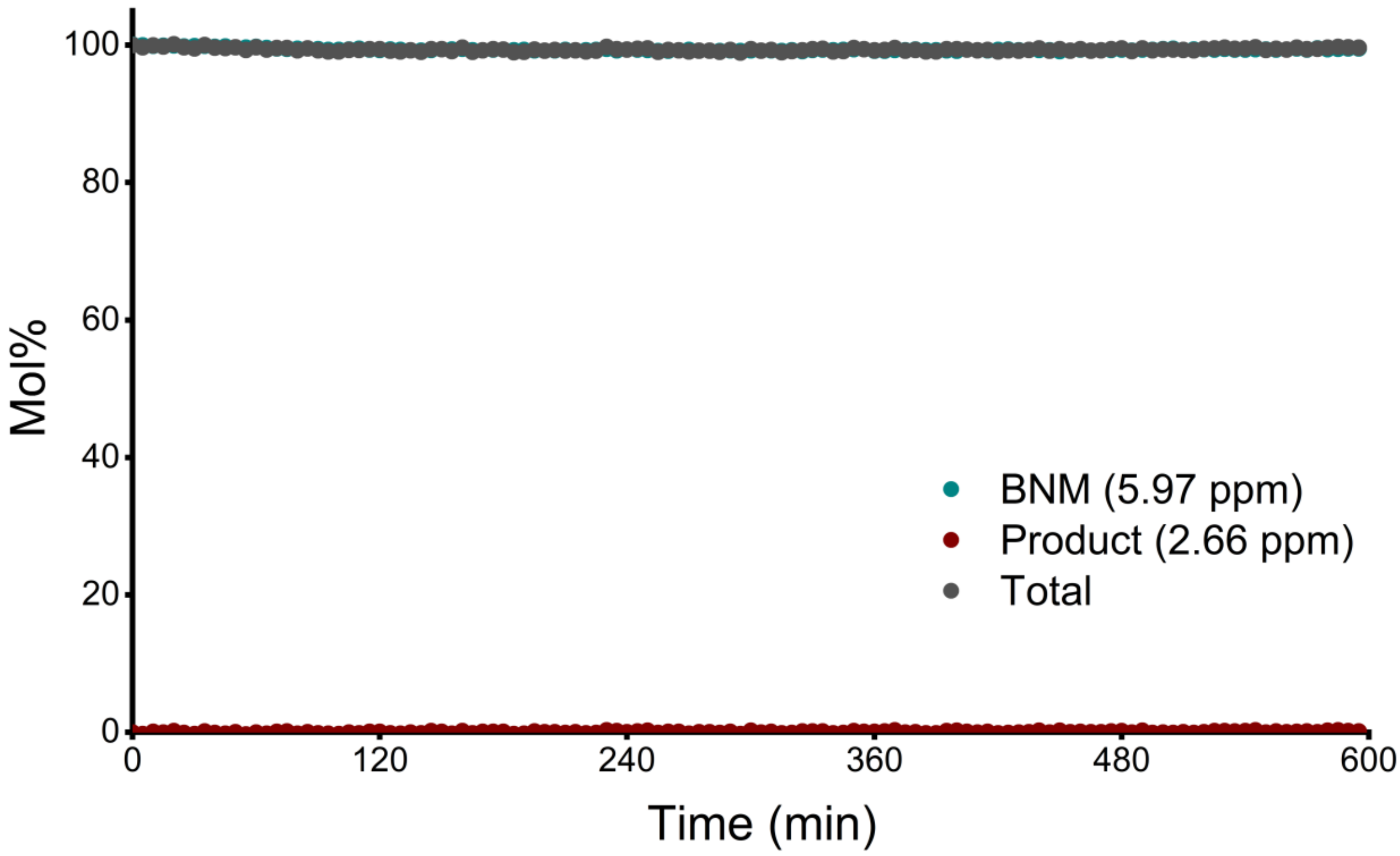

Figure S123. Monitoring catalysis using no catalyst. ^{1}H NMR (600 MHz, 298 K, DCM-d_2:MeCN-d_3 (11:1)) signal intensities of benzoyl nitromethane and Michael addition product following the reaction between benzoyl nitromethane (17.5 mM, 9.8 equiv.) and methyl vinyl ketone (31.3 mM, 17.5 equiv.) in the presence of 18-crown-6 (12.5 mM, 7.0 equiv.). The integral of the signal at 5.97 ppm was used to monitor the abundance of benzoyl nitromethane and the integral of the signal at 2.66 ppm was used to monitor the abundance of the product Michael addition product. Relaxation delay (Bruker setting D1) was set to 5 seconds.

10 Catalysis: Nitroaldol reaction between nitromethane and 4-nitrobenzaldehyde

10.1 Preparation of stock solutions used for the nitroaldol reaction

Preparation of 4-nitrobenzaldehyde stock solution

4-Nitrobenzaldehyde (46.1 mg, 305 µmol) was dissolved in DCM-d_2 (300 µL) to afford a stock solution of 4-nitrobenzaldehyde (1017 mM).

Preparation of 2,6-di-*tert*-butylpyridine stock solution

2,6-Di-*tert*-butylpyridine (10 µL, 44.5 µmol) was dissolved in DCM-d_2 (170 µL) to afford a stock solution of 2,6-di-*tert*-butylpyridine (247 mM).

10.2 Monitoring the nitroaldol reaction between 4-nitrobenzaldehyde and nitromethane in self-assembled species using ^{1}H NMR spectroscopy

10.2.1 General procedure for the nitroaldol reaction between 4-nitrobenzaldehyde and nitromethane

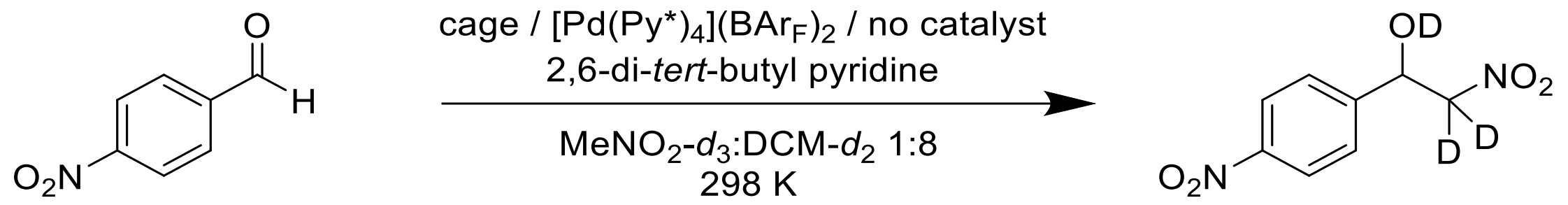

The substrates were used as received, and stock solutions were prepared according to section 10.1. All reactions were monitored over 15 hours using ^{1}H NMR spectroscopy, measuring a spectrum every 5 minutes. The relaxation delay (Bruker setting D1) was set to 5 seconds.

For cage catalyzed reactions: An NMR tube was charged with a solution of the cage/cage mixtures ([Pd] = 1.8 mM, 570 µL, 1.0 µmol, 1.0 equiv., $MeNO_2$-d_3:DCM-d_2 1:8, preparation see section 8). 4-Nitrobenzaldehyde stock solution (60 µL, 61 µmol, 1017 mM, in DCM-d_2) was added. 2,6-Di-*tert*-butylpyridine stock solution (6 µL, 1.5 µmol, 247 mM, in DCM-d_2) was added. This afforded a catalysis sample containing the cage ([Pd] = 1.6 mM, 1.0 equiv.), 4-nitrobenzaldehyde (96 mM, 59 equiv.) and 2,6-di-*tert*-butylpyridine (2.3 mM, 1.4 equiv.) in $MeNO_2$-d_3:DCM-d_2 (636 µL, 1:8).

For reactions with $[Pd(Py^*)_4](BAr_F)_2$: An NMR tube was charged with DCM-d_2 (466 µL) and $MeNO_2$-d_3 (70 µL) and a stock solution of $[Pd(Py^*)_4](BAr_F)_2$ (34 µL, 32 mM, in DCM-d_2). Then 4-nitrobenzaldehyde stock solution (60 µL, 61 µmol, 1017 mM, in DCM-d_2) was added. 2,6-Di-*tert*-butylpyridine stock solution (6 µL, 1.5 µmol, 247 mM, in DCM-d_2) was added last. This afforded a sample containing $[Pd(Py^*)_4](BAr_F)_2$ ([Pd] = 1.6 mM, 1.0 equiv.), 4-nitrobenzaldehyde (96 mM, 59 equiv.) and 2,6-di-*tert*-butylpyridine (2.3 mM, 1.4 equiv.) in $MeNO_2$-d_3:DCM-d_2 (636 µL, 1:8).

For uncatalyzed reactions: An NMR tube was charged with DCM-d_2 (500 µL) and $MeNO_2$-d_3 (70 µL). Then 4-nitrobenzaldehyde stock solution (60 µL, 61 µmol, 1017 mM, in DCM-d_2) was added. 2,6-Di-*tert*-butylpyridine stock solution (6 µL, 1.5 µmol, 247 mM, in DCM-d_2) was added last. This afforded a sample containing 4-nitrobenzaldehyde (96 mM, 59 equiv.) and 2,6-di-*tert*-butylpyridine (2.3 mM, 1.4 equiv.) in $MeNO_2$-d_3:DCM-d_2 (636 µL, 1:8).

10.2.2 Calculating the conversion of the nitroaldol reaction between 4-nitrobenzaldehyde and nitromethane

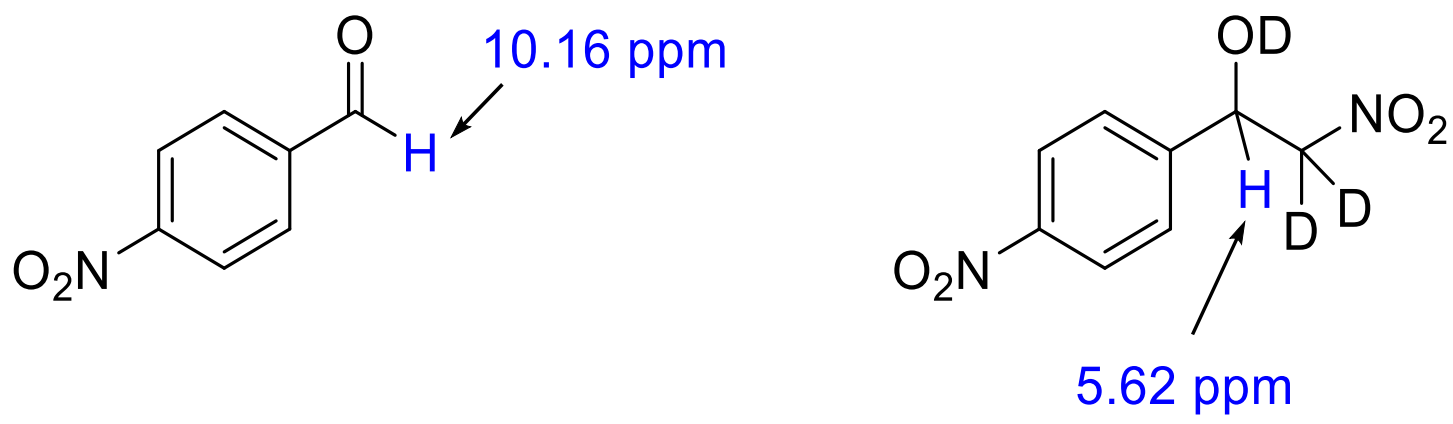

4-Nitrobenzaldehyde Product

To calculate the conversion of the nitroaldol reaction, the intensity of the signal at 10.16 ppm corresponding to the aldehyde proton of the 4-nitrobenzaldehyde starting material and the intensity of the signal at 5.62 ppm of the nitroaldol product were measured over time.

The mol% of 4-nitrobenzaldehyde and the nitroaldol product relative to each other were determined by dividing all signal intensities by the sum of the signal intensities for 4-nitrobenzaldehyde and the product at the start of the reaction (i.e. t = 0):

$$mol\% = \left(\frac{SI}{NBA_0 + P_0}\right)$$

Mol% = mol% of either 4-nitrobenzaldehyde or the nitroaldol product

SI = signal intensity of either 4-nitrobenzaldehyde or the nitroaldol product

NBA_0 = signal intensity of 4-nitrobenzaldehyde at t = 0

P_0 = signal intensity of the nitroaldol product at t = 0

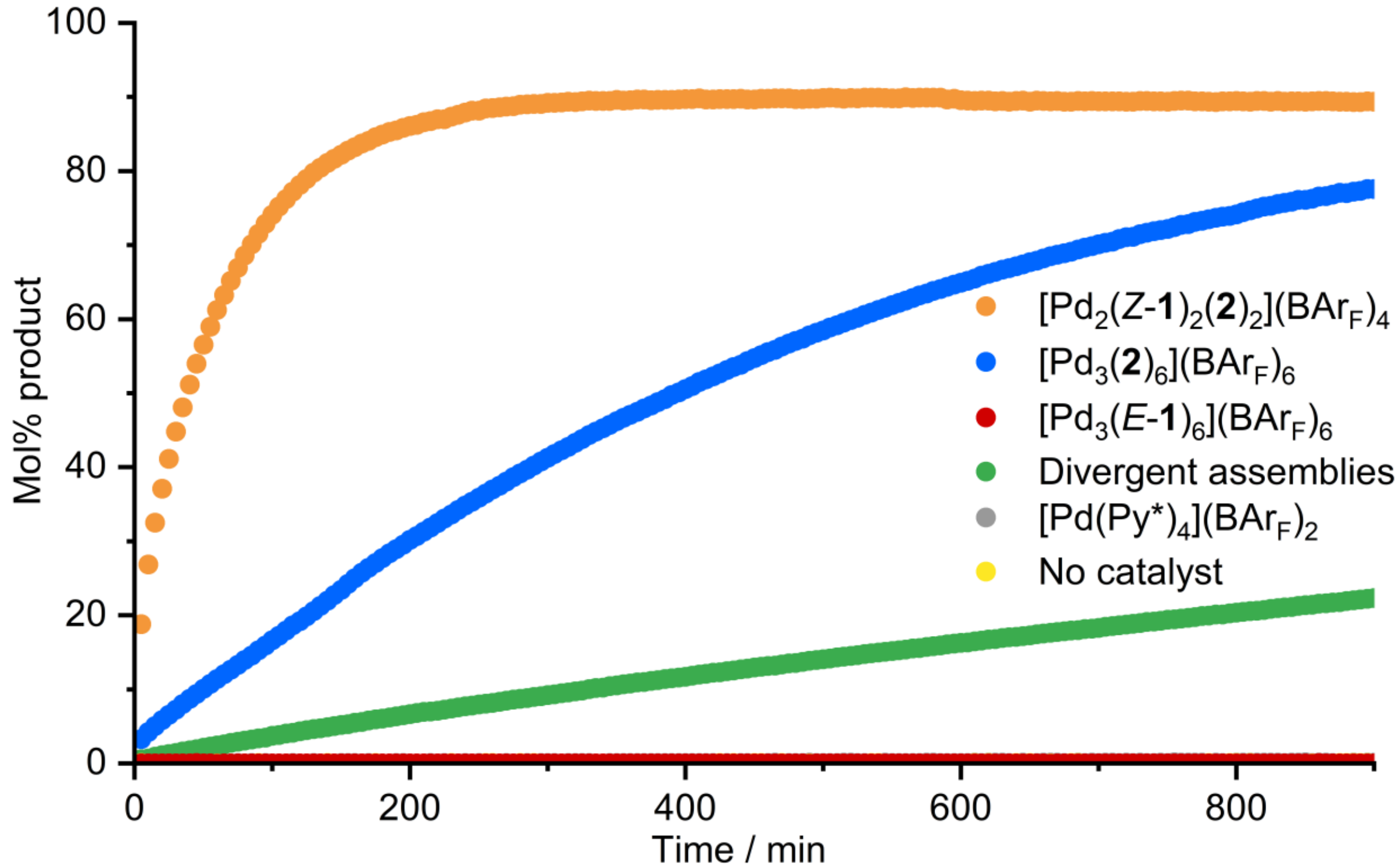

Figure S124. ^{1}H NMR (500 MHz, 298 K, $MeNO_2$-d_3:DCM-d_2 1:8) signal intensities of nitroaldol product following the reaction between 4-nitrobenzaldehyde (96 mM, 59 equiv.) and nitromethane-d_3 (used as a solvent $MeNO_2$-d_3:DCM-d_2 1:8) in the presence of 2,6-di-*tert*-butylpyridine (2.3 mM, 1.4 equiv.) and different self-assembled species as catalysts ([Pd] = 1.6 mM, 1.0 equiv.).

Equation	y = a + b*x					
Plot	[Pd2(Z-1)2(2)2](BArF	[Pd3(2)6](BArF)6	Divergent assemblie	[Pd(Py*)4](BArF)2	No catalyst	[Pd3(E-1))6](BArF)
Weight	No Weighting					
Intercept	19.12599 ± 1.53315	2.74834 ± 0.0800	0.38235 ± 0.01641	-0.02146 ± 0.01445	0.02154 ± 0.00966	-0.01228 ± 0.01107
Slope	0.7992 ± 0.04942	0.1487 ± 0.00258	0.03474 ± 5.29029E-	1.03989E-4 ± 4.65801E	-6.36871E-4 ± 3.11481E	5.41492E-4 ± 3.5691E-
Residual Sum of Squar	40.29524	0.10994	0.00462	0.00358	0.0016	0.0021
Pearson's r	0.98505	0.9988	0.99907	0.07869	-0.58585	0.47269
R-Square (COD)	0.97032	0.9976	0.99815	0.00619	0.34322	0.22344
Adj. R-Square	0.96661	0.99729	0.99792	-0.11803	0.26112	0.12637

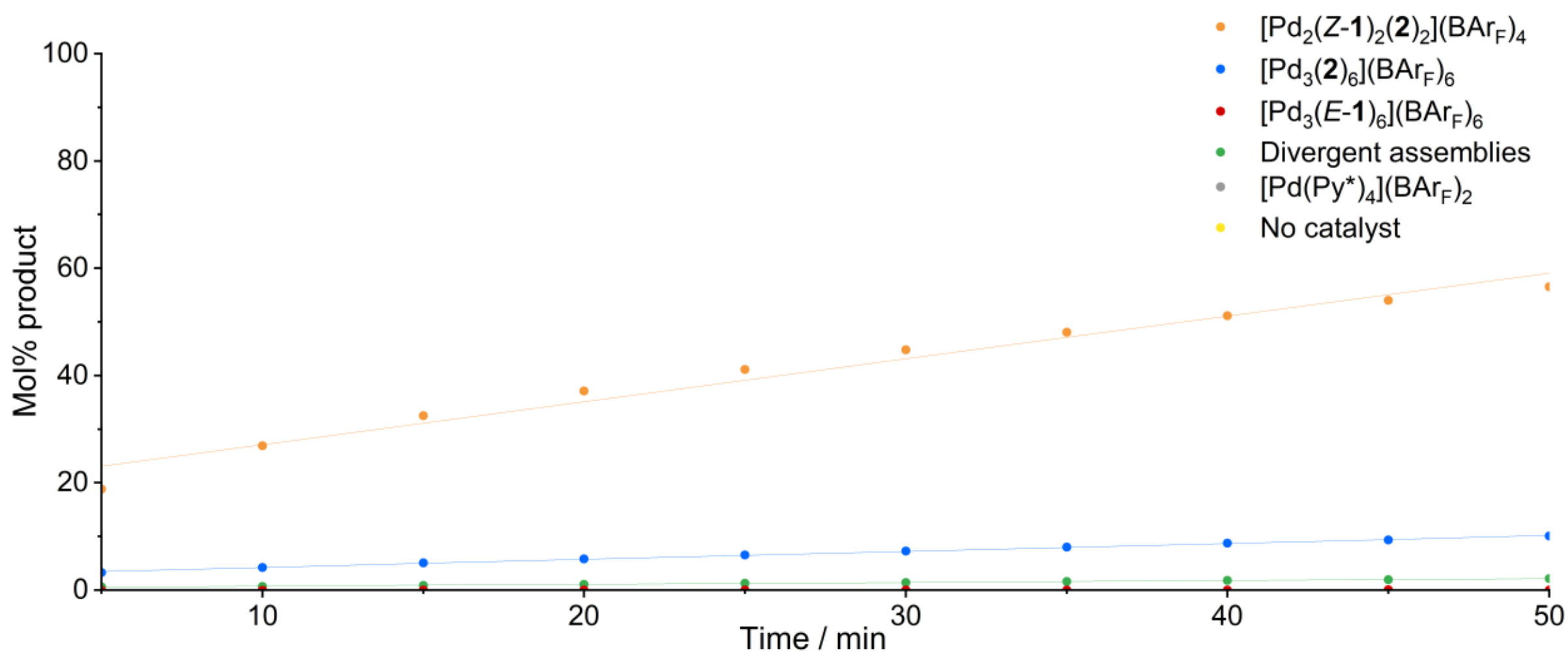

Figure S125. ^{1}H NMR (500 MHz, 298 K, $MeNO_2$-*d*3:DCM-*d*2 1:8) signal intensities of nitroaldol product following the first 50 minutes of the reaction between 4-nitrobenzaldehyde (96 mM, 59 equiv.) and nitromethane-*d*3 (used as a solvent $MeNO_2$-*d*3:DCM-*d*2 1:8) in the presence of 2,6-di-*tert*-butylpyridine (2.3 mM, 1.4 equiv.) and different self-assembled species as catalysts ([Pd] = 1.6 mM, 1.0 equiv.). Curves were fit linear.

The reaction rates of the nitroaldol reaction within the different self-assembled species were determined in the first 50 minutes of the reaction, as within this time period the rates resembled to be linear. The mol% product of each of the self-assembled species were fit to a linear curve to yield the reaction rate as the slope given in Mol% min^{-1}. To determine the reaction rate in Mol s^{-1}, the rates in Mol% min^{-1} were divided by 60 to give Mol% s^{-1} and then multiplied by the initial concentration of 4-nitrobenzaldehyde (96 mM).

Table S 27. Calculation of the initial rate constants of the nitroaldol reaction within the different self-assembled species within the first 50 minutes of the reaction between 4-nitrobenzaldehyde (96 mM, 59 equiv.) and nitromethane-*d*3 (used as a solvent $MeNO_2$-*d*3:DCM-*d*2 1:8) in the presence of 2,6-di-*tert*-butylpyridine (2.3 mM, 1.4 equiv.) and different self-assembled species as catalysts ([Pd] = 1.6 mM, 1.0 equiv.).

**Reaction rate**	**[Pd2(*Z*-1)2(2)2] (BArF)4**	**[Pd3(2)6] (BArF)6**	**Divergent assemblies**	**No catalyst**	**[Pd(Py*)4] (BArF)2**	**[Pd3(*E*-1)6] (BArF)6**
Mol% min^{-1}	0.799 ± 0.05	0.149 ± 0.003	0.0347± 0.001	1.04 ± 4 × 10^{-4}	-6.37 ± 3 × 10^{-4}	5.41 ± 3 × 10^{-4}
Mol% s^{-1}	1.33 ± 0.08 × 10^{-2}	2.48 ± 0.01 × 10^{-3}	5.79 ± 0.08 × 10^{-4}	1.73 ± 7 × 10^{-6}	-1.06 ± 3 × 10^{-6}	9.02 ± 6 × 10^{-6}
Mol s^{-1}	1.27 ± 0.1 × 10^{-3}	2.38 ± 0.04 × 10^{-4}	5.56 ± 0.08 × 10^{-5}	1.66 ± 7 × 10^{-7}	-1.02 ± 0.5 × 10^{-6}	8.66 ± 6 × 10^{-6}

10.3 Using $[Pd_2(Z\text{-}\mathbf{1})_2(\mathbf{2})_2](BAr_F)_4$ to catalyze the nitroaldol reaction between 4-nitrobenzaldehyde and nitromethane

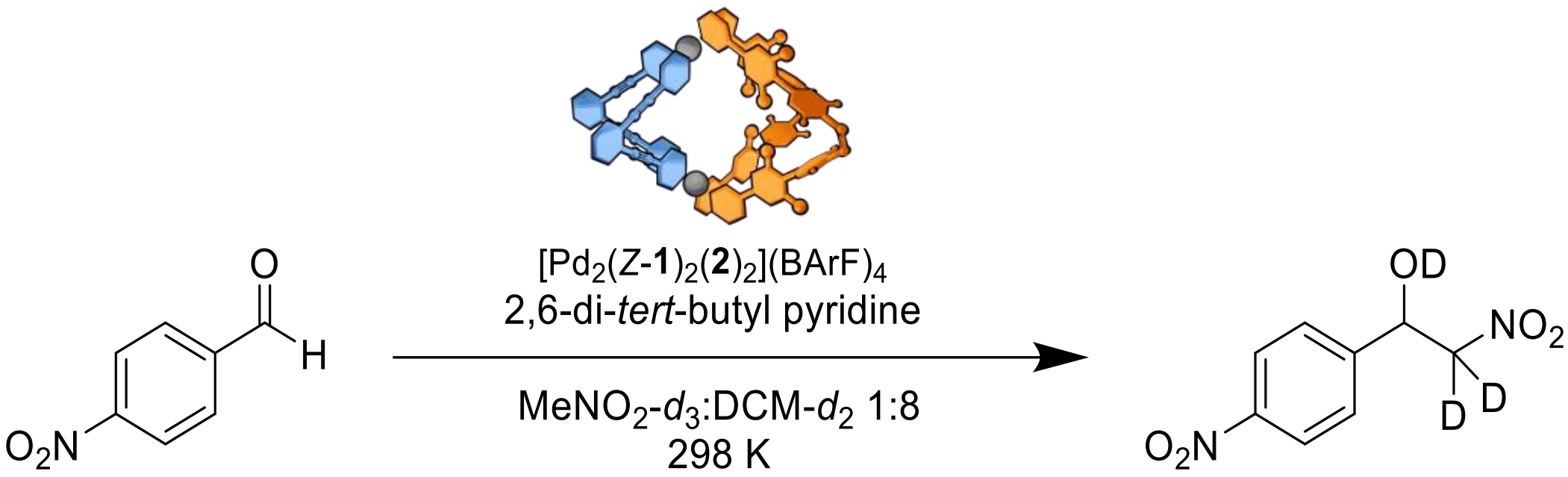

An NMR tube was charged with a solution of $[Pd_2(Z\text{-}\mathbf{1})_2(\mathbf{2})_2](BAr_F)_4$ ([Pd] = 1.8 mM, 570 µL, 1.0 µmol, 1.0 equiv., $MeNO_2$-d_3:DCM-d_2 1:8, preparation see section 8.5). Then 4-nitrobenzaldehyde stock solution (60 µL, 61 µmol, 1017 mM, in DCM-d_2) was added. 2,6-Di-*tert*-butylpyridine stock solution (6 µL, 1.5 µmol, 247 mM, in DCM-d_2) was added last. This afforded a catalysis sample containing the cage ([Pd] = 1.6 mM, 1.0 equiv.), 4-nitrobenzaldehyde (96 mM, 59 equiv.) and 2,6-di-*tert*-butylpyridine (2.3 mM, 1.4 equiv.) in $MeNO_2$-d_3:DCM-d_2 (636 µL, 1:8).

The mol% of 4-nitrobenzaldehyde and the product were plot against time (see Supporting Information section 10.2.2 for details) to determine whether the metastable cage $[Pd_2(Z\text{-}\mathbf{1})_2(\mathbf{2})_2](BAr_F)_4$ can catalyse the nitroaldol reaction between 4-nitrobenzaldehyde and nitromethane. A total of 90% product formation was observed after 15 hours, while a plateau has reached already after ~4 hours (Figure S126).

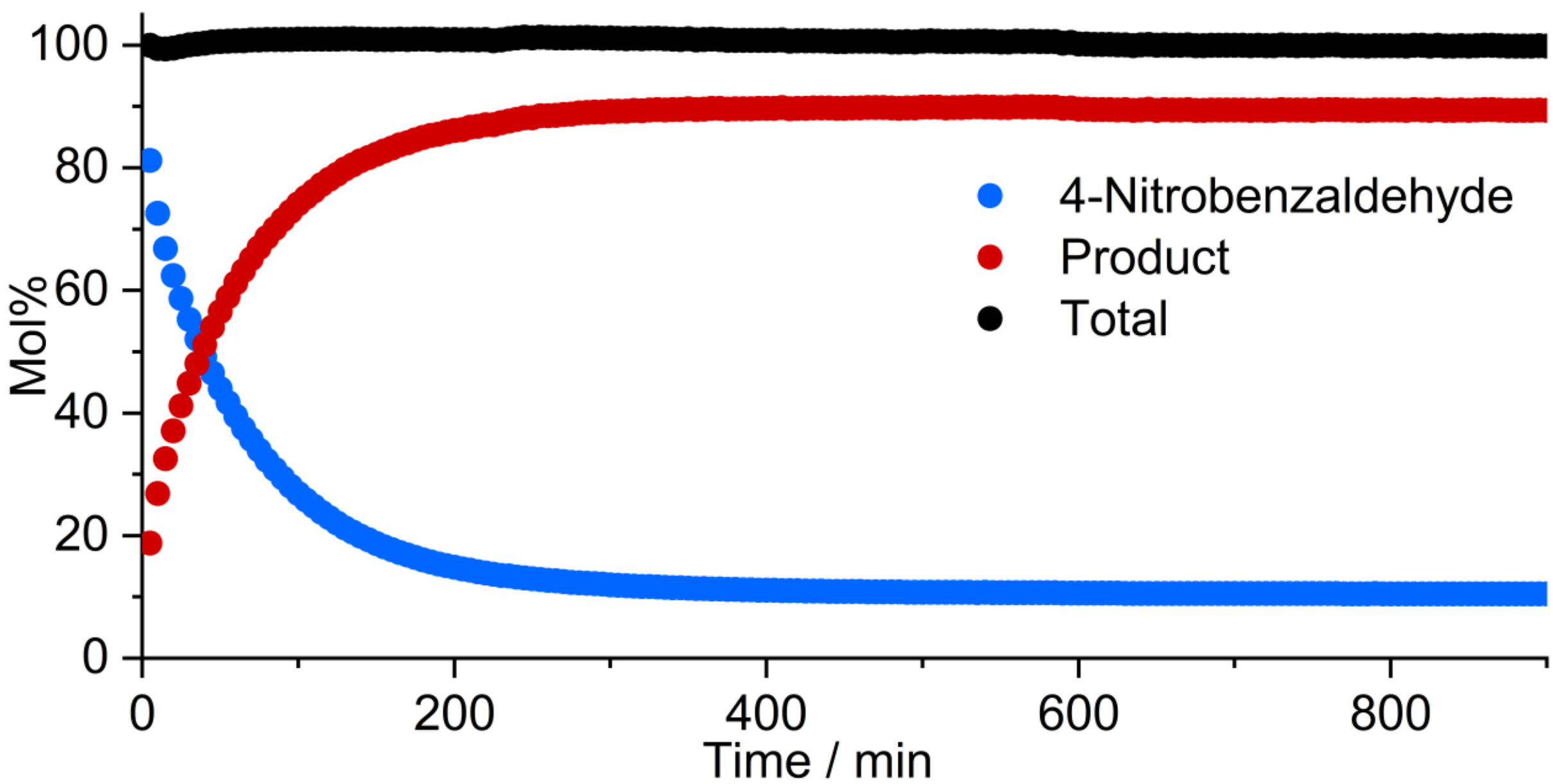

Figure S126. Monitoring nitroaldol catalysis within heteroleptic cage $[Pd_2(Z\text{-}\mathbf{1})_2(\mathbf{2})_2](BAr_F)_4$. ^1H NMR (500 MHz, 298 K, $MeNO_2$-d_3:DCM-d_2 1:8) signal intensities of 4-nitrobenzaldehyde and nitroaldol product following the reaction between 4-nitrobenzaldehyde (96 mM, 59 equiv.) and nitromethane-d_3 (used as a solvent $MeNO_2$-d_3:DCM-d_2 1:8) in the presence of 2,6-di-*tert*-butylpyridine (2.3 mM, 1.4 equiv.) and $[Pd_2(Z\text{-}\mathbf{1})_2(\mathbf{2})_2](BAr_F)_4$ ([Pd] = 1.6 mM, 1.0 equiv.). The integral of the signal at 10.16 ppm was used to monitor the abundance of 4-nitrobenzaldehyde and the integral of the signal at 5.62 ppm was used to monitor the abundance of the nitroaldol product. Relaxation delay (Bruker setting D1) was set to 5 seconds.

10.4 Using $[Pd_3(2)_6](BAr_F)_6$ to catalyze the nitroaldol reaction between 4-nitrobenzaldehyde and nitromethane

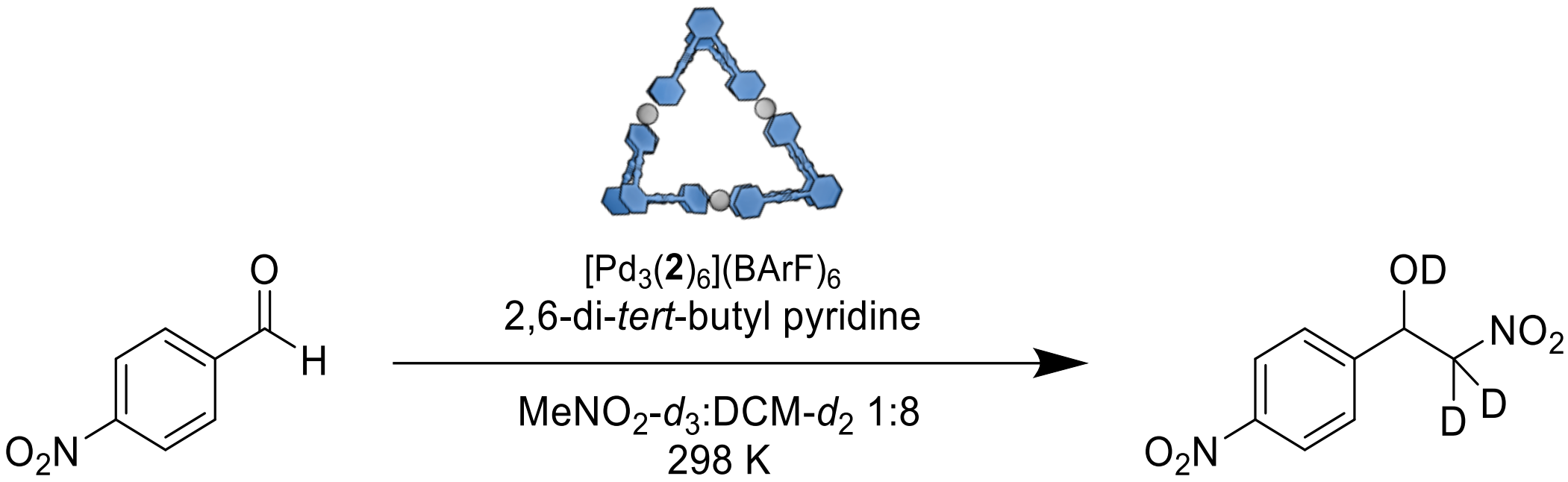

An NMR tube was charged with a solution of $[Pd_3(\mathbf{2})_6](BAr_F)_6$ ([Pd] = 1.8 mM, 570 µL, 1.0 µmol, 1.0 equiv., $MeNO_2$-d_3:DCM-d_2 1:8, preparation see section 8.3). Then 4-nitrobenzaldehyde stock solution (60 µL, 61 µmol, 1017 mM, in DCM-d_2) was added. 2,6-Di-*tert*-butylpyridine stock solution (6 µL, 1.5 µmol, 247 mM, in DCM-d_2) was added last. This afforded a catalysis sample containing the cage ([Pd] = 1.6 mM, 1.0 equiv.), 4-nitrobenzaldehyde (96 mM, 59 equiv.) and 2,6-di-*tert*-butylpyridine (2.3 mM, 1.4 equiv.) in $MeNO_2$-d_3:DCM-d_2 (636 µL, 1:8).

The mol% of 4-nitrobenzaldehyde and the product were plot against time (see Supporting Information section 10.2.2 for details) to determine whether the homoleptic cage $[Pd_3(\mathbf{2})_6](BAr_F)_6$ can catalyse the nitroaldol reaction between 4-nitrobenzaldehyde and nitromethane. A total of 78% product formation was observed after 15 hours (Figure S127).

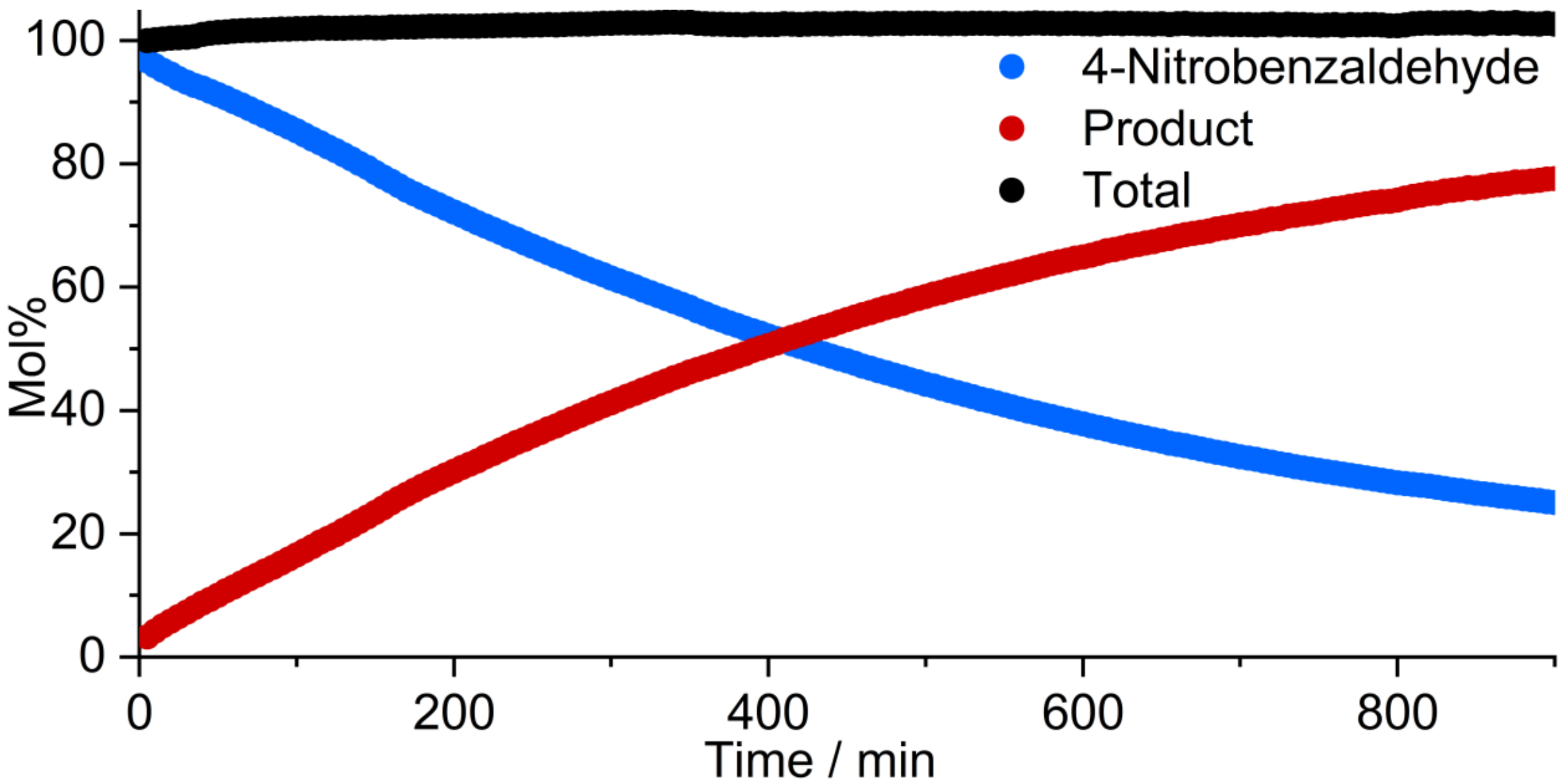

Figure S127. Monitoring nitroaldol catalysis within homoleptic cage $[Pd_3(\mathbf{2})_6](BAr_F)_6$. ^1H NMR (500 MHz, 298 K, $MeNO_2$-d_3:DCM-d_2 1:8) signal intensities of 4-nitrobenzaldehyde and nitroaldol product following the reaction between 4-nitrobenzaldehyde (96 mM, 59 equiv.) and nitromethane-d_3 (used as a solvent $MeNO_2$-d_3:DCM-d_2 1:8) in the presence of 2,6-di-*tert*-butylpyridine (2.3 mM, 1.4 equiv.) and $[Pd_3(\mathbf{2})_6](BAr_F)_6$ ([Pd] = 1.6 mM, 1.0 equiv.). The integral of the signal at 10.16 ppm was used to monitor the abundance of 4-nitrobenzaldehyde and the integral of the signal at 5.62 ppm was used to monitor the abundance of the nitroaldol product. Relaxation delay (Bruker setting D1) was set to 5 seconds.

10.5 Using $[Pd_3(E\text{-}\mathbf{1})_6](BAr_F)_6$ and $[Pd_4(E\text{-}\mathbf{1})_8](BAr_F)_8$ to catalyze the nitroaldol reaction between 4-nitrobenzaldehyde and nitromethane

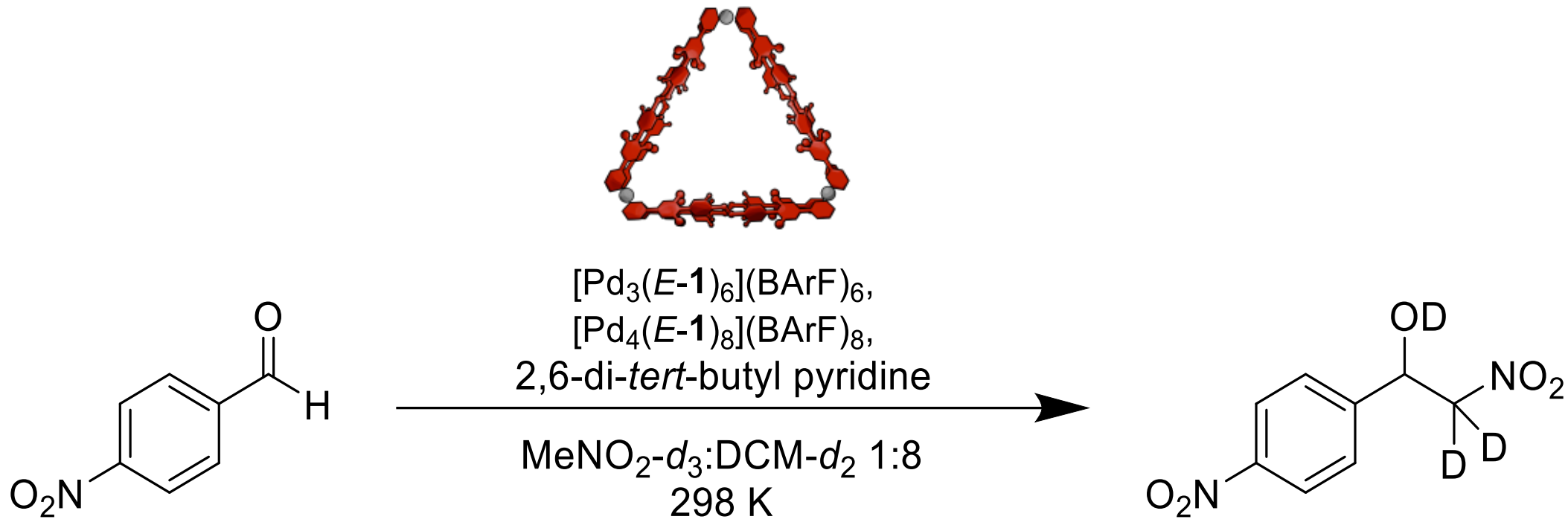

An NMR tube was charged with a solution of $[Pd_3(E\text{-}\mathbf{1})_6](BAr_F)_6$ / $[Pd_4(E\text{-}\mathbf{1})_8](BAr_F)_8$ (total [Pd] = 1.8 mM, 570 µL, 1.0 µmol, 1.0 equiv., $MeNO_2$-d_3:DCM-d_2 1:8, preparation see section 8.2). Then 4-nitrobenzaldehyde stock solution (60 µL, 61 µmol, 1017 mM, in DCM-d_2) was added. 2,6-Di-*tert*-butylpyridine stock solution (6 µL, 1.5 µmol, 247 mM, in DCM-d_2) was added last. This afforded a catalysis sample containing the cages ([Pd] = 1.6 mM, 1.0 equiv.), 4-nitrobenzaldehyde (96 mM, 59 equiv.) and 2,6-di-*tert*-butylpyridine (2.3 mM, 1.4 equiv.) in $MeNO_2$-d_3:DCM-d_2 (636 µL, 1:8).

The mol% of 4-nitrobenzaldehyde and the product were plot against time (see Supporting Information section 10.2.2 for details) to determine whether the homoleptic cages $[Pd_3(E\text{-}\mathbf{1})_6](BAr_F)_6$ / $[Pd_4(E\text{-}\mathbf{1})_8](BAr_F)_8$ can catalyse the nitroaldol reaction between 4-nitrobenzaldehyde and nitromethane. No product formation was observed after 15 hours (Figure S128).

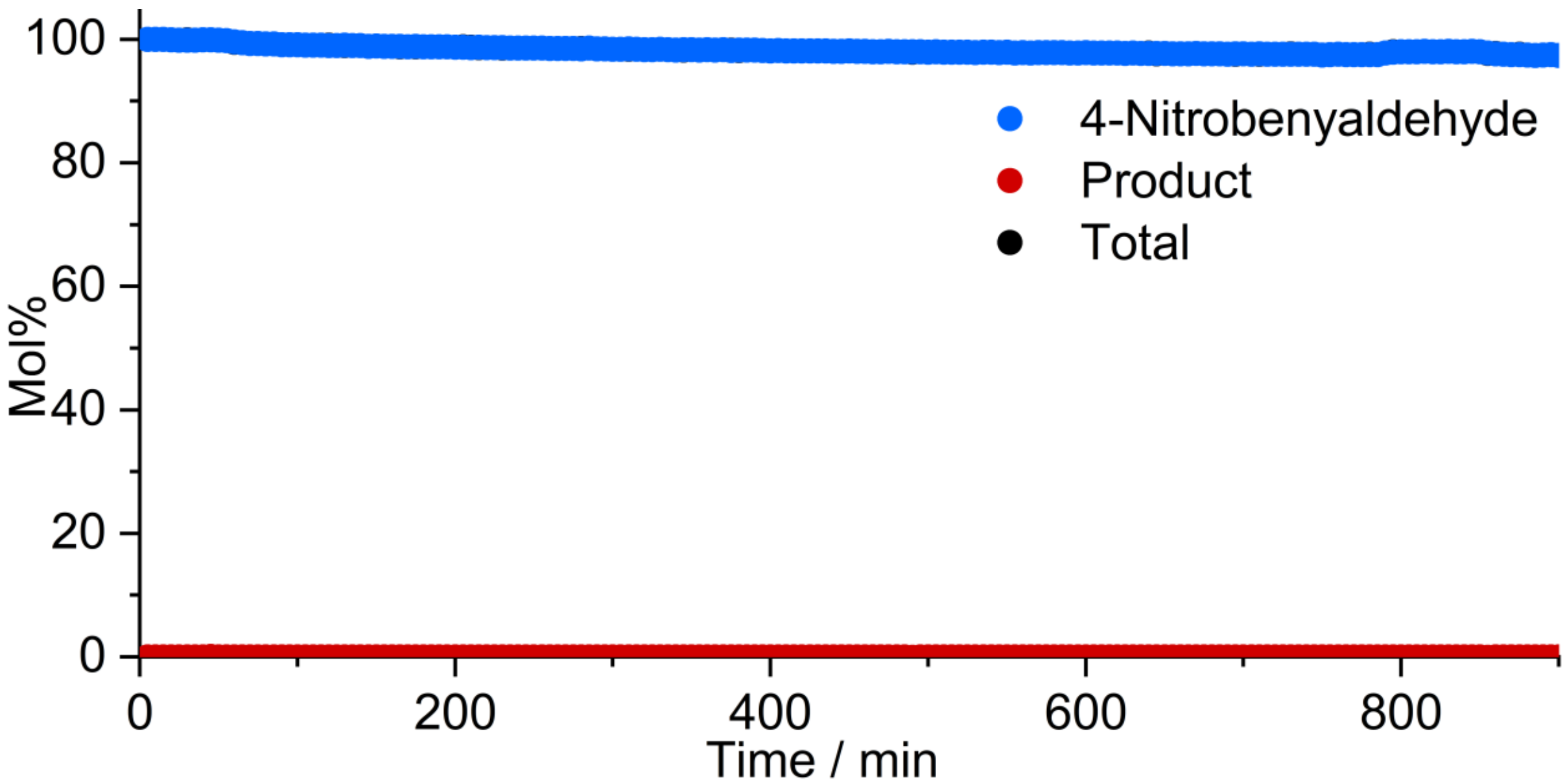

Figure S128. Monitoring nitroaldol catalysis within homoleptic cages $[Pd_3(E\text{-}\mathbf{1})_6](BAr_F)_6$ and $[Pd_4(E\text{-}\mathbf{1})_8](BAr_F)_8$. ^1H NMR (500 MHz, 298 K, $MeNO_2$-d_3:DCM-d_2 1:8) signal intensities of 4-nitrobenzaldehyde and nitroaldol product following the reaction between 4-nitrobenzaldehyde (96 mM, 59 equiv.) and nitromethane-d_3 (used as a solvent $MeNO_2$-d_3:DCM-d_2 1:8) in the presence of 2,6-di-*tert*-butylpyridine (2.3 mM, 1.4 equiv.) and $[Pd_3(E\text{-}\mathbf{1})_6](BAr_F)_6$ / $[Pd_4(E\text{-}\mathbf{1})_8](BAr_F)_8$ (total [Pd] = 1.6 mM, 1.0 equiv.). The integral of the signal at 10.16 ppm was used to monitor the abundance of 4-nitrobenzaldehyde and the integral of the signal at 5.62 ppm was used to monitor the abundance of the nitroaldol product. Relaxation delay (Bruker setting D1) was set to 5 seconds.

10.6 Using a mixture of $[Pd_3(E\text{-}\mathbf{1})_6](BAr_F)_6$, $[Pd_3(\mathbf{2})_6](BAr_F)_6$, $[Pd_3(E\text{-}\mathbf{1})_4(\mathbf{2})_2](BAr_F)_6$ and $[Pd_4(E\text{-}\mathbf{1})_4(\mathbf{2})_4](BAr_F)_8$ to catalyze the nitroaldol reaction between 4-nitrobenzaldehyde and nitromethane

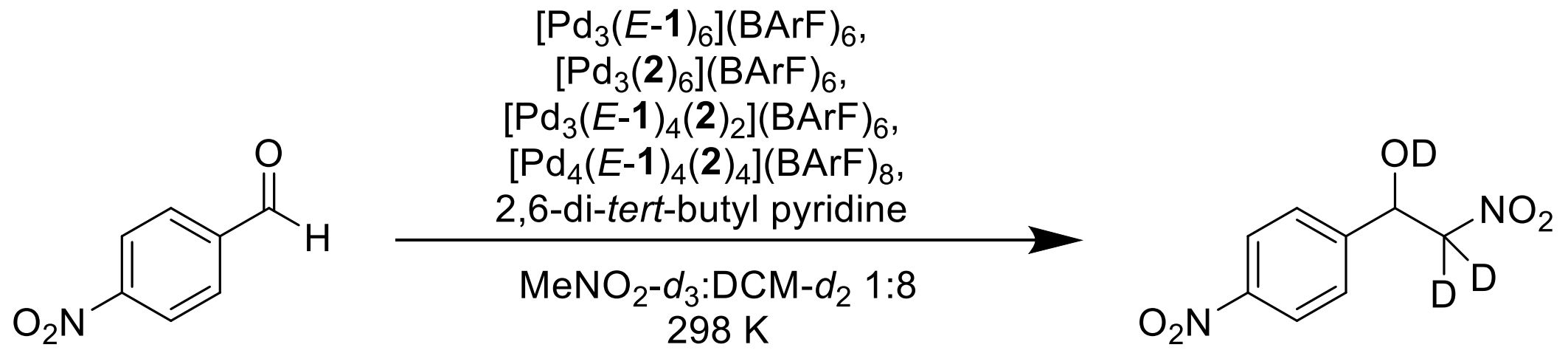

An NMR tube was charged with a solution of $[Pd_3(E\text{-}\mathbf{1})_6](BAr_F)_6$, $[Pd_3(\mathbf{2})_6](BAr_F)_6$, $[Pd_3(E\text{-}\mathbf{1})_4(\mathbf{2})_2](BAr_F)_6$ and $[Pd_4(E\text{-}\mathbf{1})_4(\mathbf{2})_4](BAr_F)_8$ (total [Pd] = 1.8 mM, 570 µL, 1.0 µmol, 1.0 equiv., $MeNO_2$-d_3:DCM-d_2 1:8, preparation see section 8.4). Then 4-nitrobenzaldehyde stock solution (60 µL, 61 µmol, 1017 mM, in DCM-d_2) was added. 2,6-Di-*tert*-butylpyridine stock solution (6 µL, 1.5 µmol, 247 mM, in DCM-d_2) was added last. This afforded a catalysis sample containing the cages ([Pd] = 1.6 mM, 1.0 equiv.), 4-nitrobenzaldehyde (96 mM, 59 equiv.) and 2,6-di-*tert*-butylpyridine (2.3 mM, 1.4 equiv.) in $MeNO_2$-d_3:DCM-d_2 (636 µL, 1:8).

The mol% of 4-nitrobenzaldehyde and the product were plot against time (see Supporting Information section 10.2.2 for details) to determine whether the divergent assemblies can catalyse the nitroaldol reaction between 4-nitrobenzaldehyde and nitromethane. A total of 22% product formation was observed after 15 hours (Figure S129).

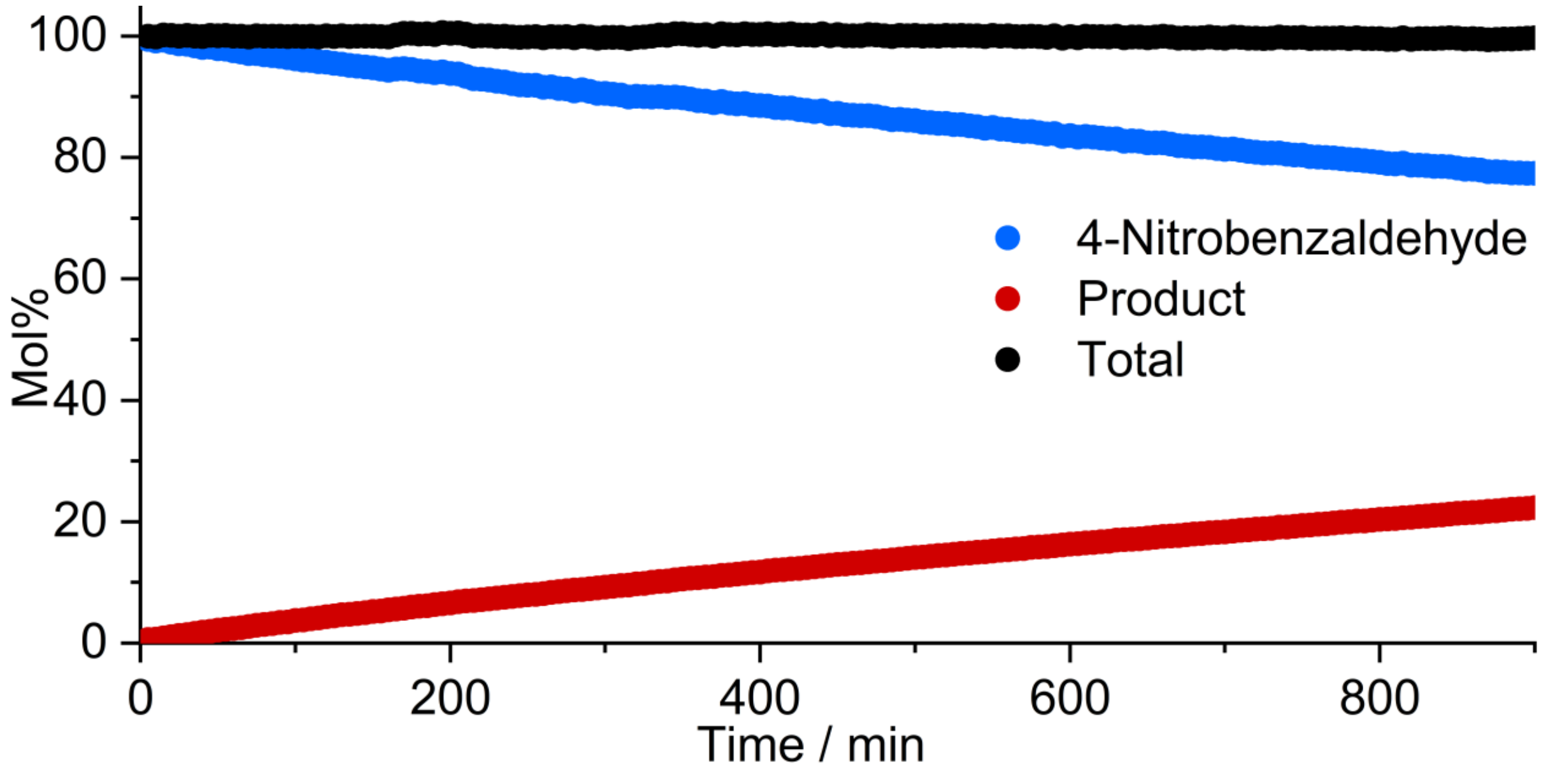

Figure S129. Monitoring nitroaldol catalysis within the divergent assemblies $[Pd_3(E\text{-}\mathbf{1})_6](BAr_F)_6$, $[Pd_3(\mathbf{2})_6](BAr_F)_6$, $[Pd_3(E\text{-}\mathbf{1})_4(\mathbf{2})_2](BAr_F)_6$ and $[Pd_4(E\text{-}\mathbf{1})_4(\mathbf{2})_4](BAr_F)_8$. ^1H NMR (500 MHz, 298 K, $MeNO_2$-d_3:DCM-d_2 1:8) signal intensities of 4-nitrobenzaldehyde and nitroaldol product following the reaction between 4-nitrobenzaldehyde (96 mM, 59 equiv.) and nitromethane-d_3 (used as a solvent $MeNO_2$-d_3:DCM-d_2 1:8) in the presence of 2,6-di-*tert*-butylpyridine (2.3 mM, 1.4 equiv.) and the cages ([Pd] = 1.6 mM, 1.0 equiv.). The integral of the signal at 10.16 ppm was used to monitor the abundance of 4-nitrobenzaldehyde and the integral of the signal at 5.62 ppm was used to monitor the abundance of the nitroaldol product. Relaxation delay (Bruker setting D1) was set to 5 seconds.

10.7 Monitoring the nitroaldol reaction between 4-nitrobenzaldehyde and nitromethane using $[Pd(Py^*)_4](BAr_F)_2$

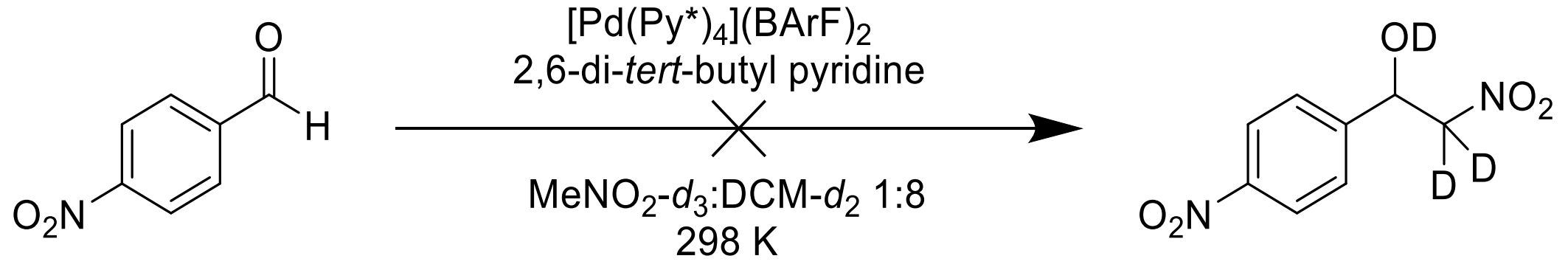

An NMR tube was charged with DCM-d_2 (466 µL) and $MeNO_2$-d_3 (70 µL) and a stock solution of $[Pd(Py^*)_4](BAr_F)_2$ (34 µL, 32 mM, in DCM-d_2). Then 4-nitrobenzaldehyde stock solution (60 µL, 61 µmol, 1017 mM, in DCM-d_2) was added. 2,6-Di-*tert*-butylpyridine stock solution (6 µL, 1.5 µmol, 247 mM, in DCM-d_2) was added last. This afforded a sample containing $[Pd(Py^*)_4](BAr_F)_2$ ([Pd] = 1.6 mM, 1.0 equiv.), 4-nitrobenzaldehyde (96 mM, 59 equiv.) and 2,6-di-*tert*-butylpyridine (2.3 mM, 1.4 equiv.) in $MeNO_2$-d_3:DCM-d_2 (636 µL, 1:8).

The mol% of 4-nitrobenzaldehyde and the product were plot against time (see Supporting Information section 10.2.2 for details) to determine whether $[Pd(Py^*)_4](BAr_F)_2$ can catalyse the nitroaldol reaction between 4-nitrobenzaldehyde and nitromethane. No product formation was observed after 15 hours (Figure S130) showing that the reaction is not palladium(II) catalyzed.

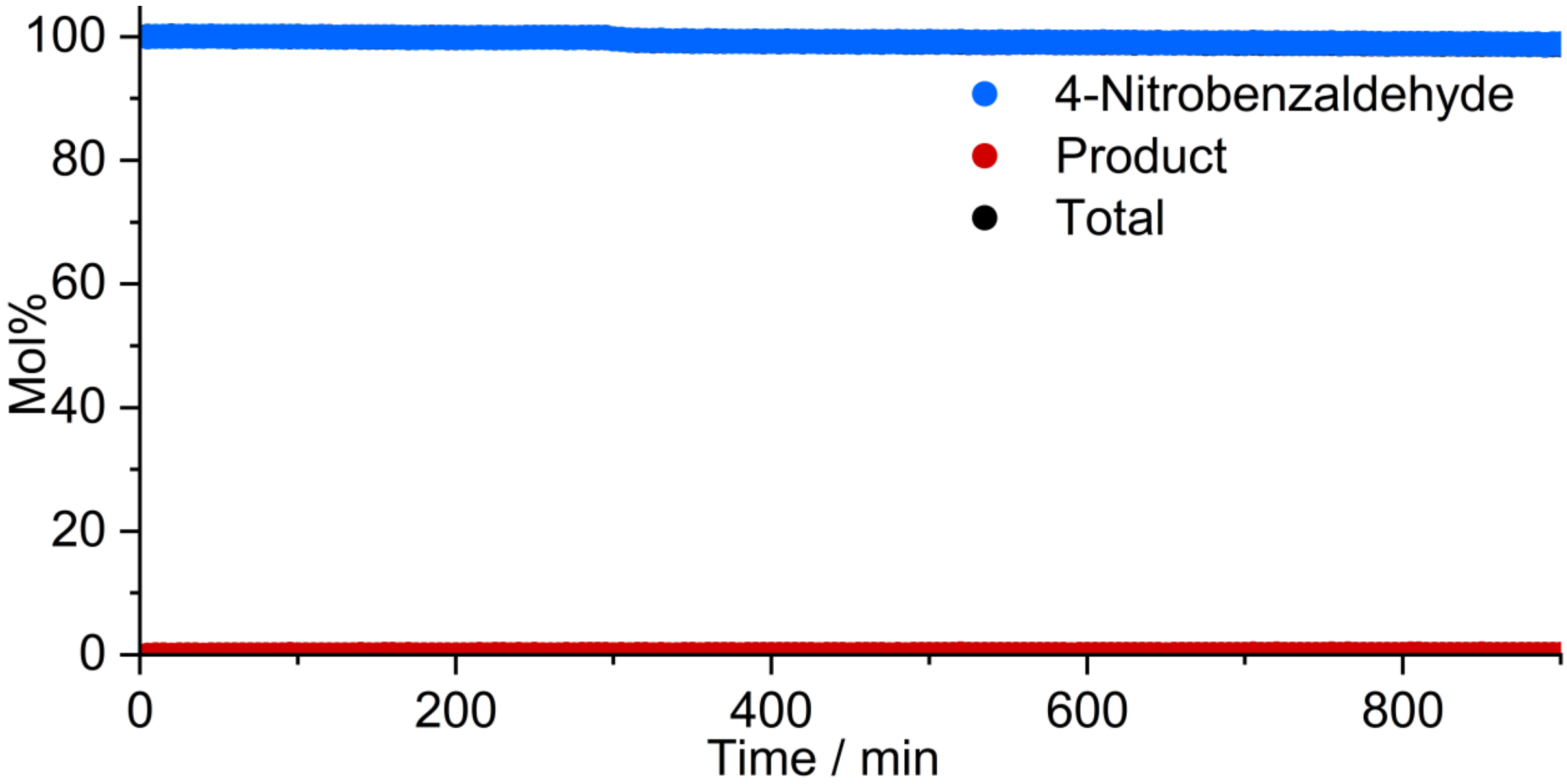

Figure S130. Monitoring nitroaldol catalysis using $[Pd(Py^*)_4](BAr_F)_2$ as a catalyst. ^1H NMR (500 MHz, 298 K, $MeNO_2$-d_3:DCM-d_2 1:8) signal intensities of 4-nitrobenzaldehyde and nitroaldol product following the reaction between 4-nitrobenzaldehyde (96 mM, 59 equiv.) and nitromethane-d_3 (used as a solvent $MeNO_2$-d_3:DCM-d_2 1:8) in the presence of 2,6-di-*tert*-butylpyridine (2.3 mM, 1.4 equiv.) and $[Pd(Py^*)_4](BAr_F)_2$ ([Pd] = 1.6 mM, 1.0 equiv.). The integral of the signal at 10.16 ppm was used to monitor the abundance of 4-nitrobenzaldehyde and the integral of the signal at 5.62 ppm was used to monitor the abundance of the nitroaldol product. Relaxation delay (Bruker setting D1) was set to 5 seconds.

10.8 Monitoring the nitroaldol reaction between 4-nitrobenzaldehyde and nitromethane using no catalyst

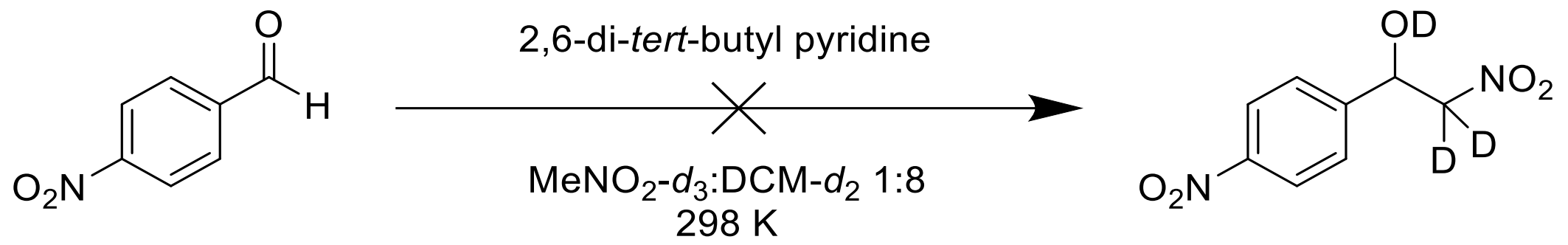

An NMR tube was charged with DCM-d_2 (500 µL) and $MeNO_2$-d_3 (70 µL). Then 4-nitrobenzaldehyde stock solution (60 µL, 61 µmol, 1017 mM, in DCM-d_2) was added. 2,6-Di-*tert*-butylpyridine stock solution (6 µL, 1.5 µmol, 247 mM, in DCM-d_2) was added last. This afforded a sample containing 4-nitrobenzaldehyde (96 mM, 59 equiv.) and 2,6-di-*tert*-butylpyridine (2.3 mM, 1.4 equiv.) in $MeNO_2$-d_3:DCM-d_2 (636 µL, 1:8).

The mol% of 4-nitrobenzaldehyde and the product were plot against time (see Supporting Information section 10.2.2 for details) to determine whether thee is a background nitroaldol reaction between 4-nitrobenzaldehyde and nitromethane. No product formation was observed after 15 hours (Figure S131) showing that there is no background reaction.

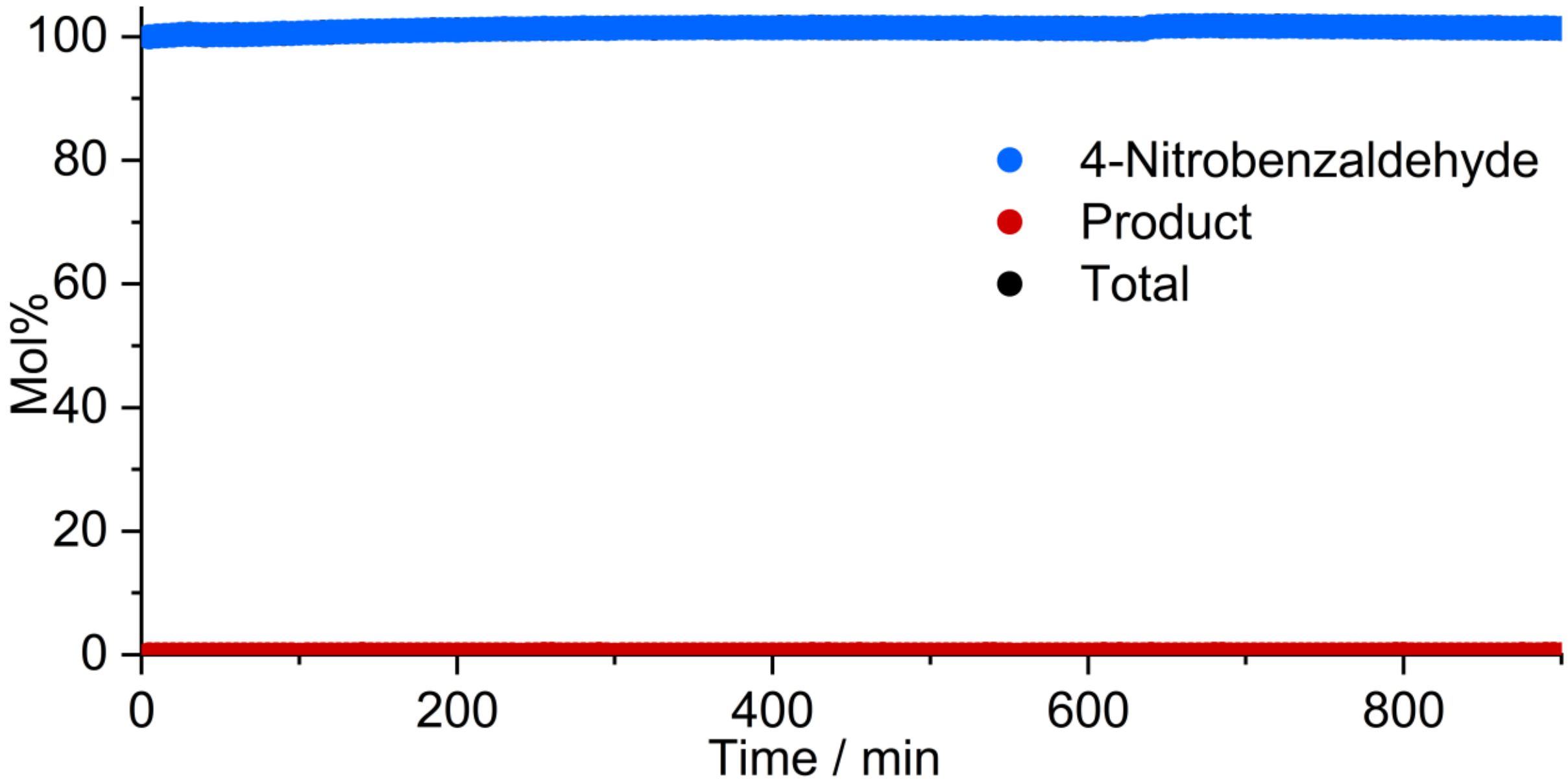

Figure S131. Monitoring nitroaldol catalysis using no catalyst. ^{1}H NMR (500 MHz, 298 K, $MeNO_2$-d_3:DCM-d_2 1:8) signal intensities of 4-nitrobenzaldehyde and nitroaldol product following the reaction between 4-nitrobenzaldehyde (96 mM, 59 equiv.) and nitromethane-d_3 (used as a solvent $MeNO_2$-d_3:DCM-d_2 1:8) in the presence of 2,6-di-*tert*-butylpyridine (2.3 mM, 1.4 equiv.). The integral of the signal at 10.16 ppm was used to monitor the abundance of 4-nitrobenzaldehyde and the integral of the signal at 5.62 ppm was used to monitor the abundance of the nitroaldol product. Relaxation delay (Bruker setting D1) was set to 5 seconds.

10.9 Competitive binding: Preventing the nitroaldol reaction by adding triflate to the metastable cage

Tetrabutylammonium trifluoromethanesulfonate (TBAOTf) is known to be a strong competitive binder within the cavity of lantern shaped $[Pd_2(\mathbf{L})_4]^{4+}$ cages. Adding TBAOTf to the metastable cage should hence occupy the cage cavity, making it unavailable for further guest binding and preventing the reaction from occurring. It further indicates, that the catalysis takes place inside the cage cavity rather than on the outside.

For this reason, an NMR sample of $[Pd_2(Z\text{-}\mathbf{1})_2(\mathbf{2})_2](BAr_F)_4$ ([Pd] = 1.8 mM, 570 µL, 1.0 µmol, 1.0 equiv., $MeNO_2$-d_3:DCM-d_2 1:8, preparation see section 8.5) was prepared. Then a stock solution of TBAOTf (13 µL, 78 mM, 1.0 equiv. in DCM-d_2) was added. Finally 4-nitrobenzaldehyde stock solution (60 µL, 61 µmol, 1017 mM, in DCM-d_2) and 2,6-di-*tert*-butylpyridine stock solution (6 µL, 1.5 µmol, 247 mM, in DCM-d_2) were added. The reaction was monitored over 15 hours using ^{1}H NMR spectroscopy, measuring a spectrum every 5 minutes. The relaxation delay (Bruker setting D1) was set to 5 seconds.

No product formation was observed over the period of 15 hours (Figure S132), proving that the nitroaldol reaction requires access to an unbound cage cavity.

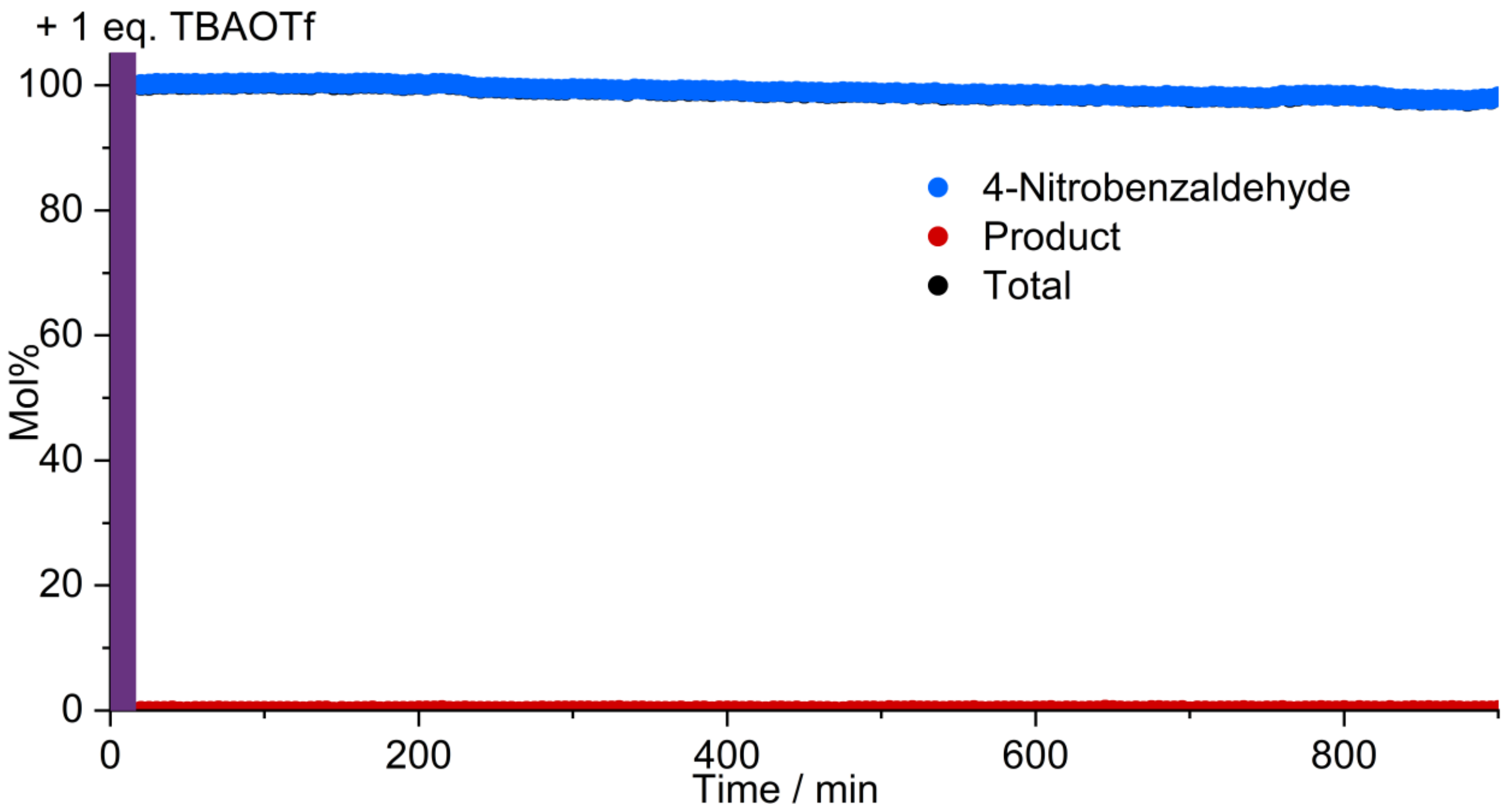

Figure S132. Monitoring catalysis within heteroleptic cage $[Pd_2(Z\text{-}\mathbf{1})_2(\mathbf{2})_2](BAr_F)_4$ after the addition of 1.0 equiv. of TBAOTf. ^{1}H NMR (500 MHz, 298 K, $MeNO_2$-d_3:DCM-d_2 1:8) signal intensities of 4-nitrobenzaldehyde and nitroaldol product following the reaction between 4-nitrobenzaldehyde (96 mM, 59 equiv.) and nitromethane-d_3 (used as a solvent $MeNO_2$-d_3:DCM-d_2 1:8) in the presence of 2,6-di-*tert*-butylpyridine (2.3 mM, 1.4 equiv.) and $[Pd_2(Z\text{-}\mathbf{1})_2(\mathbf{2})_2](BAr_F)_4$ ([Pd] = 1.6 mM, 1.0 equiv.). The integral of the signal at 10.16 ppm was used to monitor the abundance of 4-nitrobenzaldehyde and the integral of the signal at 5.62 ppm was used to monitor the abundance of the nitroaldol product. Relaxation delay (Bruker setting D1) was set to 5 seconds.

10.10 Binding of triflate (TBAOTf) by $[Pd_2(Z\text{-}\mathbf{1})_2(\mathbf{2})_2](BAr_F)_4$ in:$MeNO_2$-d_3:DCM-d_2 8:1

Tetrabutylammonium trifluoromethanesulfonate (TBAOTf, 12.2 mg, 31.1 µmol) was dissolved in DCM-d_2 (400 µL) to afford a stock solution of TBAOTf (78 mM).

A sample of $[Pd_2(Z\text{-}\mathbf{1})_2(\mathbf{2})_2](BAr_F)_4$ ([Pd] = 1.8 mM, 570 µL, 1.0 µmol, 1.0 equiv.) was prepared in $MeNO_2$-d_3:DCM-d_2 1:8 according to the procedure described in section 8.5.

TBAOTf stock solution was added stepwise (0.4 equiv. / 0.8 equiv. / 1.0 equiv. / 1.5 equiv. / 2.0 equiv. / 4.0 equiv. and 6.0 equiv.) to $[Pd_2(Z\text{-}\mathbf{1})_2(\mathbf{2})_2](BAr_F)_4$ and a ^1H and ^{19}F NMR spectrum was obtained after each addition (Figure S133 and Figure S134).

The addition of TBAOTf to the metastable cage $[Pd_2(Z\text{-}\mathbf{1})_2(\mathbf{2})_2](BAr_F)_4$ caused a downfield shift of H^m, H^l and H^a in the ^1H NMR spectrum, which increased progressively with further additions of TBAOTf. After adding a total of 6.0 equiv. of TBAOTf H^m, H^l and H^a have shifted downfield by total 0.255 ppm, 0.632 ppm, and 0.704 ppm, respectively. We could observe that H^m only shifted until the addition of 1.0 equiv. of TBAOTf, while H^l and H^a kept shifting with the addition of further TBAOTf. We suggest that the first equivalent of TBAOTf gets bound within the cage cavity, causing the shift of H^m. Once the cage is saturated with TBAOTf, a second binding event on the outside of the cage takes place, causing H^l and H^a to further shift downfield, while the shift of H^m remains unchanged.

Fitting the ^1H NMR titration data of H^m, H^l and H^a to a 1:1 fast-exchanging binding model using a nonlinear least-square algorithm built in the BindFit website[7-8] remained challenging, since multiple binding events are happening.

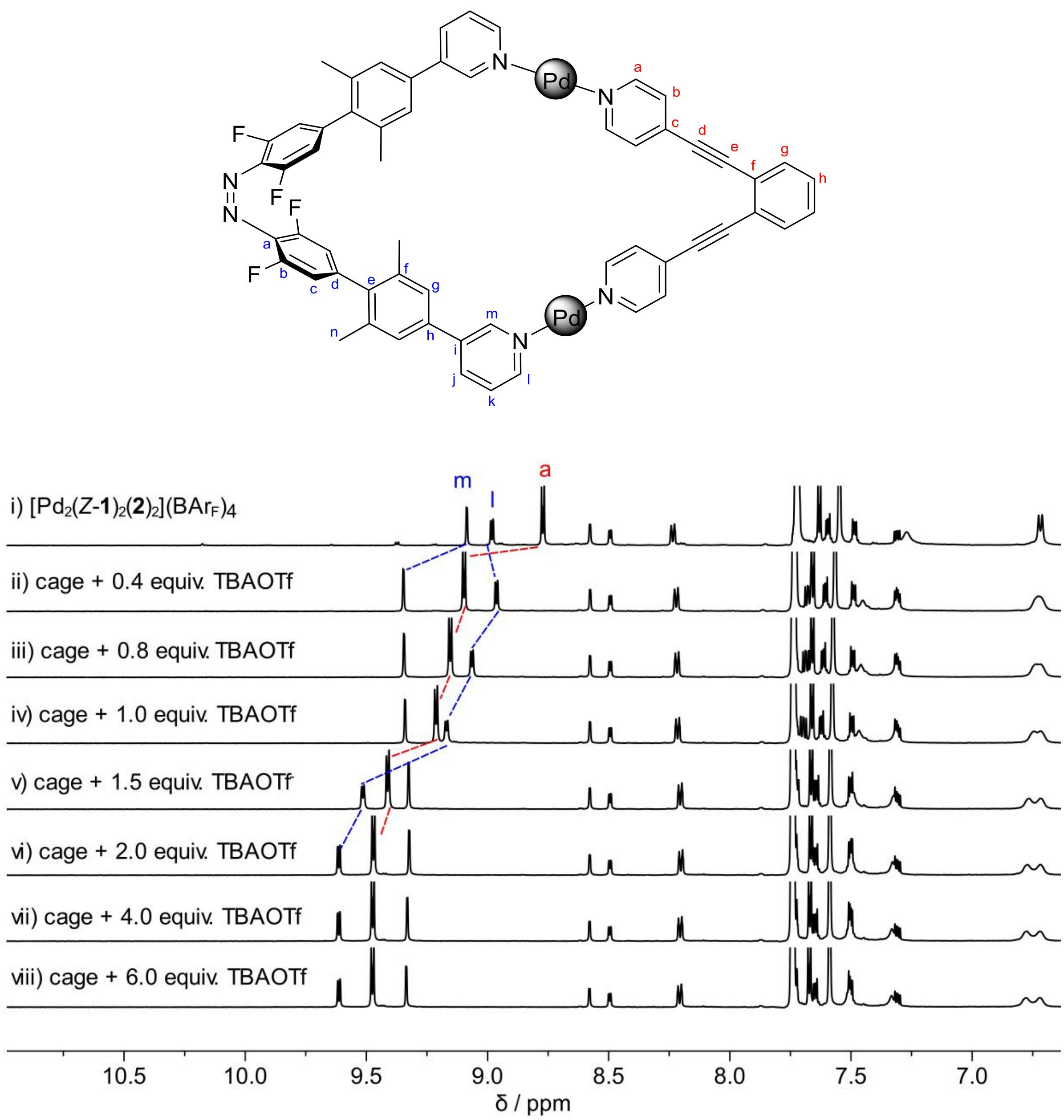

Figure S133. Partial ^{1}H NMR spectra (600 MHz, 298 K, MeNO$_2$-d_3:DCM-d_2 1:8) of i) the metastable [Pd$_2$(*Z*-**1**)$_2$(**2**)$_2$](BAr$_F$)$_4$ ([Pd] = 1.8 mM, 570 µL, 1.0 equiv., MeNO$_2$-d_3:DCM-d_2 1:8), and after the addition of ii) 0.4 equiv. TBAOTf (5 µL stock solution), iii) 0.8 equiv. TBAOTf (10 µL stock solution), iv) 1.0 equiv. TBAOTf (13 µL stock solution), v) 1.5 equiv. TBAOTf (20 µL stock solution), vi) 2.0 equiv. TBAOTf (27 µL stock solution), vii) 4.0 equiv. TBAOTf (54 µL stock solution), and viii) 6.0 equiv. TBAOTf (80 µL stock solution).

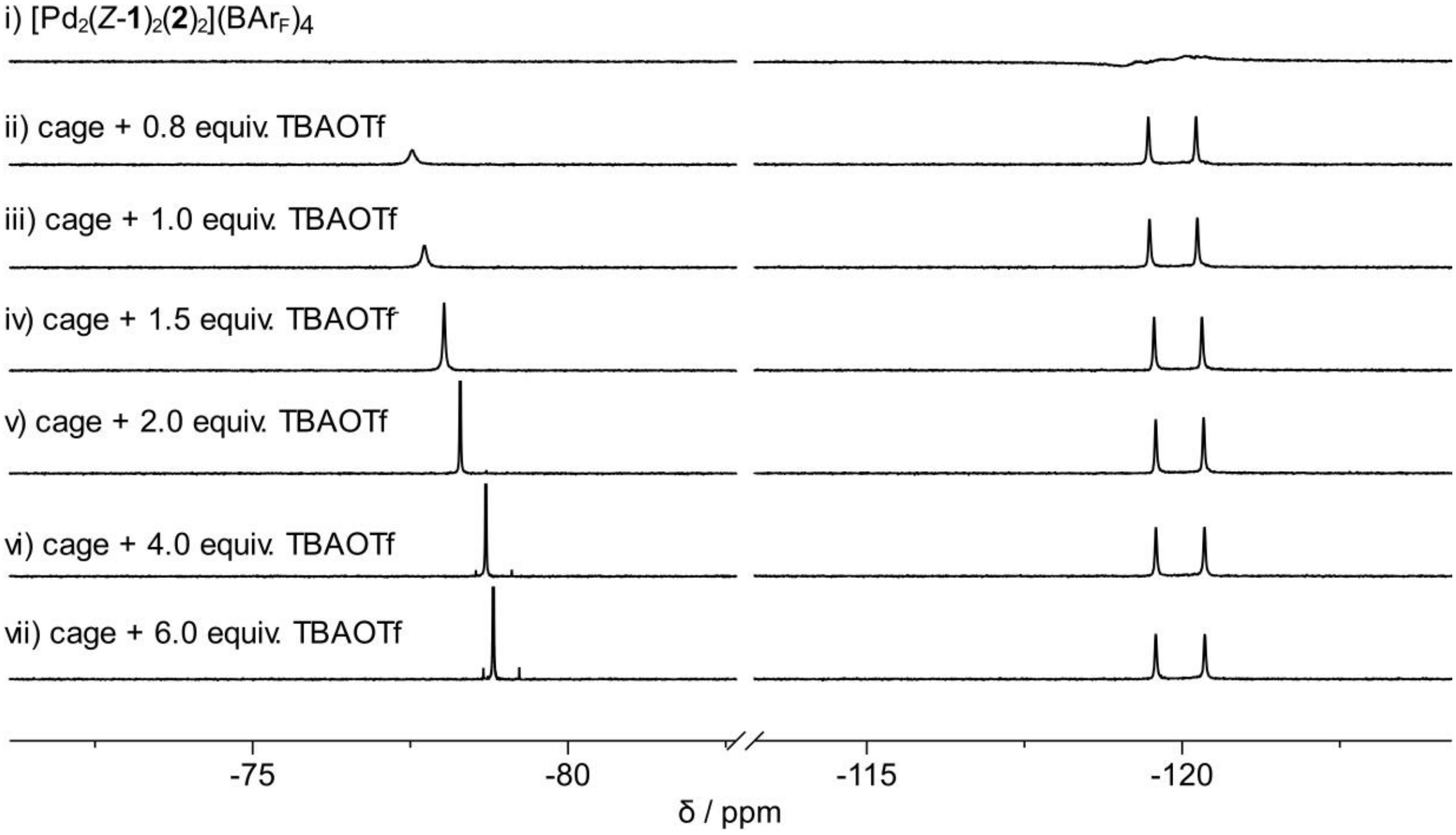

Figure S134. Partial ^{19}F NMR spectra (565 MHz, 298 K, MeCN-d_3) of i) the metastable $[Pd_2(Z\text{-}\mathbf{1})_2(\mathbf{2})_2](BAr_F)_4$ ([Pd] = 2.1 mM, 500 µL, 1.0 equiv., MeCN-d_3), and after the addition of ii) 0.8 equiv. TBAOTf (10 µL stock solution), iii) 1.0 equiv. TBAOTf (13 µL stock solution), iv) 1.5 equiv. TBAOTf (20 µL stock solution), v) 2.0 equiv. TBAOTf (27 µL stock solution), vi) 4.0 equiv. TBAOTf (54 µL stock solution), and vii) 6.0 equiv. TBAOTf (80 µL stock solution).

10.11 Manipulating the nitroaldol catalysis rates using visible light

10.11.1 Switching nitroaldol catalysis in DCM-d_2:MeNO$_2$-d_3 8:1

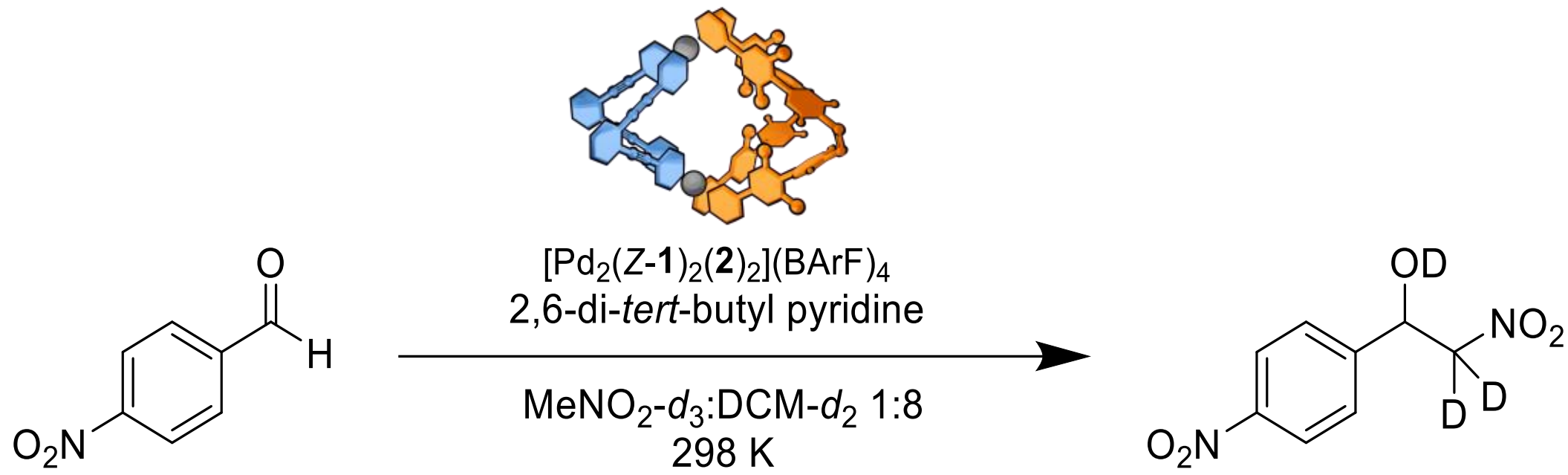

An NMR tube was charged with a solution of [Pd$_2$(*Z*-**1**)$_2$(**2**)$_2$](BAr$_F$)$_4$ ([Pd] = 1.8 mM, 570 µL, 1.0 µmol, 1.0 equiv., MeNO$_2$-d_3:DCM-d_2 1:8, preparation see section 8.5). Then 4-nitrobenzaldehyde stock solution (60 µL, 61 µmol, 1017 mM, in DCM-d_2) was added. 2,6-Di-*tert*-butylpyridine stock solution (6 µL, 1.5 µmol, 247 mM, in DCM-d_2) was added last. This afforded a catalysis sample containing the cage ([Pd] = 1.6 mM, 1.0 equiv.), 4-nitrobenzaldehyde (96 mM, 59 equiv.) and 2,6-di-*tert*-butylpyridine (2.3 mM, 1.4 equiv.) in MeNO$_2$-d_3:DCM-d_2 (636 µL, 1:8).

The catalysis sample was monitored using ^{1}H NMR spectroscopy, with a spectrum collected every 5 min. After 1 hour the sample was removed from the NMR instrument and irradiated with an LED centred at 424 nm for 10 min. The sample was immediately placed back in the NMR instrument to avoid exposure to ambient light and was monitored for an additional 1 hour. The sample was then removed from the NMR instrument again, irradiated with 530 nm light for 10 min, immediately placed back in the NMR instrument and monitored for an additional 15 hours. The generation of the nitroaldol product was determined by measuring the signal intensity for the 4-nitrobenzaldehyde signal at 10.16 ppm ppm and the nitroaldol product signal at 5.62 ppm (for details see section 10.2.2). The signal intensities were plot against time to estimate how the rate of product formation was affected upon irradiation with 530 nm and 424 nm light (Figure S135).

We could observe a rapid stop of the nitroaldol catalysis after irradiating the catalysis sample with 424 nm light. Irradiation with 530 nm light reformed the metastable [Pd$_2$(*Z*-**1**)$_2$(**2**)$_2$](BAr$_F$)$_4$ cage and catalysis resumed immediately. However, we observed that the rate of reaction after the irradiation with 530 nm light was not as fast as in the beginning. We propose that this is due to both, incomplete reassembly of the cage in the used solvent (MeNO$_2$-d_3:DCM-d_2 1:8) and consumption of the substrates.

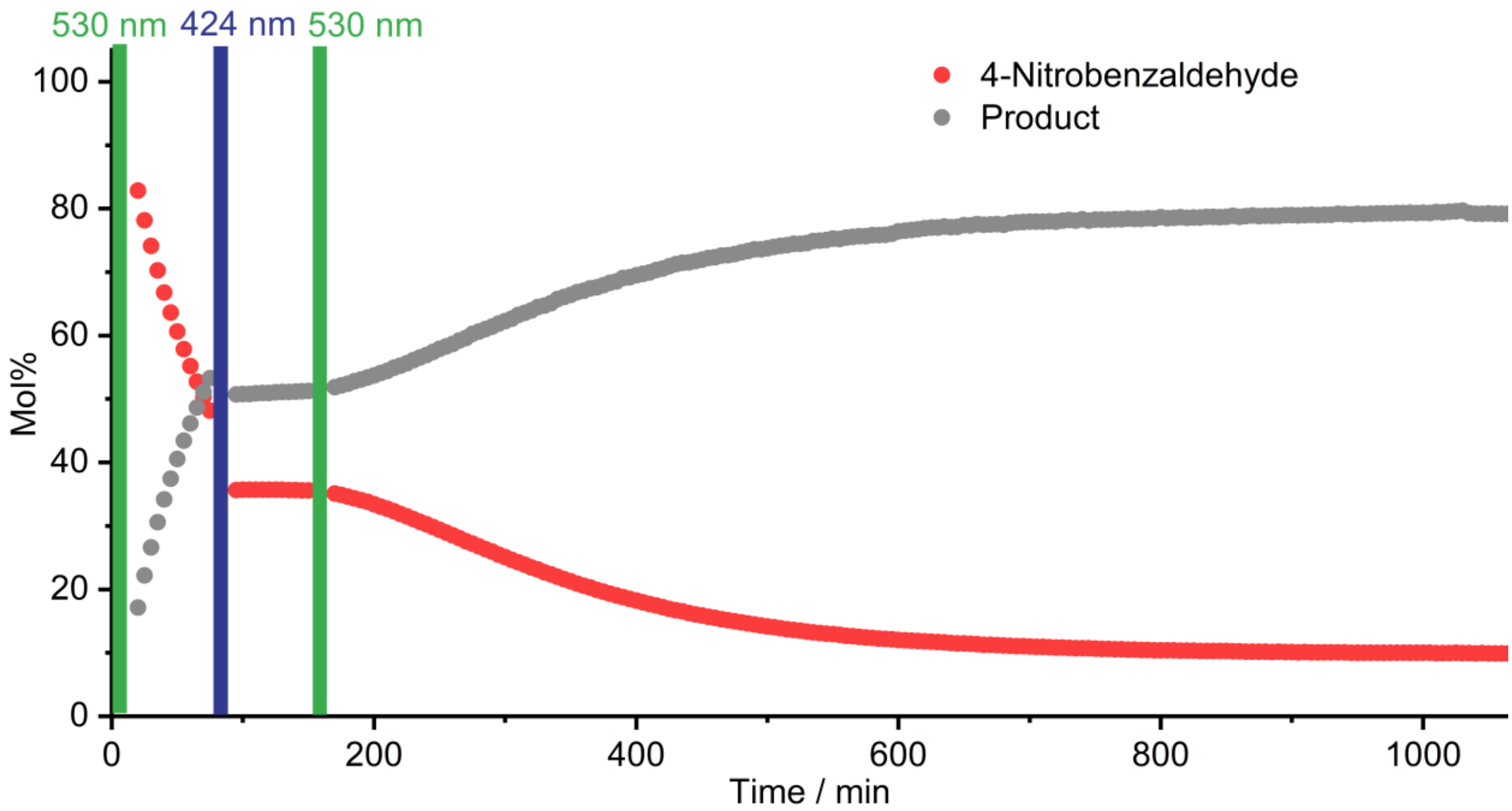

Figure S135. Monitoring switchable nitroaldol catalysis within heteroleptic cage $[Pd_2(Z\text{-}\mathbf{1})_2(\mathbf{2})_2](BAr_F)_4$. ^1H NMR (500 MHz, 298 K, $MeNO_2\text{-}d_3$:$DCM\text{-}d_2$ 1:8) signal intensities of 4-nitrobenzaldehyde and nitroaldol product following the reaction between 4-nitrobenzaldehyde (96 mM, 59 equiv.) and nitromethane-d_3 (used as a solvent $MeNO_2\text{-}d_3$:$DCM\text{-}d_2$ 1:8) in the presence of 2,6-di-*tert*-butylpyridine (2.3 mM, 1.4 equiv.) and $[Pd_2(Z\text{-}\mathbf{1})_2(\mathbf{2})_2](BAr_F)_4$ ([Pd] = 1.6 mM, 1.0 equiv.). The integral of the signal at 10.16 ppm was used to monitor the abundance of 4-nitrobenzaldehyde and the integral of the signal at 5.62 ppm was used to monitor the abundance of the nitroaldol product. Relaxation delay (Bruker setting D1) was set to 5 seconds. The green vertical line indicates when the sample was irradiated with 530 nm light. The violet vertical line indicates when the sample was irradiated with 424 nm light.

10.11.2 Switching nitroaldol catalysis in DCM-d_2:MeCN-d_3:MeNO2-d_3 9:1:1.4

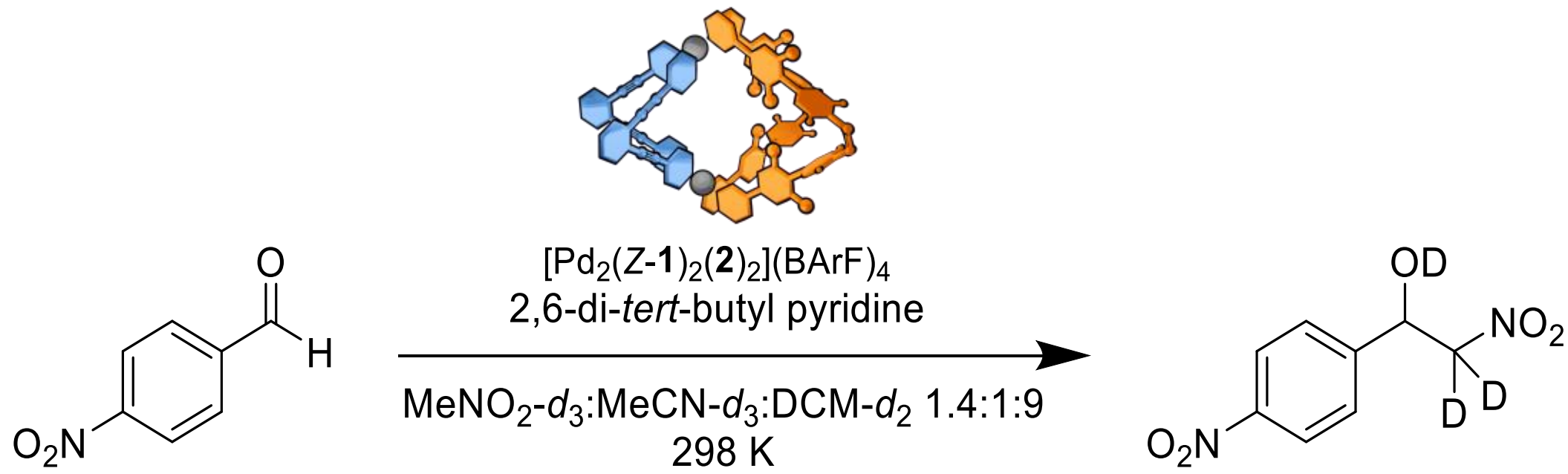

An NMR tube was charged with a mixture of [Pd_3(*E*-**1**)$_6$](BAr_F)$_6$, [Pd_3(**2**)$_6$](BAr_F)$_6$, [Pd_3(*E*-**1**)$_4$(**2**)$_2$](BAr_F)$_6$ and [Pd_4(*E*-**1**)$_4$(**2**)$_4$](BAr_F)$_8$ ([Pd] = 1.8 mM, 570 µL, 1.0 µmol, 1.0 equiv., $MeNO_2$-d_3:MeCN-d_3:DCM-d_2 1.4:1:9, preparation see section 8.4). The catalysis sample was then irradiated with 424 nm light to ensure that the same mixture of species is present like in the following irradiation cycles. Then 4-nitrobenzaldehyde stock solution (60 µL, 61 µmol, 1017 mM, in DCM-d_2) was added. 2,6-Di-*tert*-butylpyridine stock solution (6 µL, 1.5 µmol, 247 mM, in DCM-d_2) was added last. This afforded a catalysis sample containing the cage ([Pd] = 1.6 mM, 1.0 equiv.), 4-nitrobenzaldehyde (96 mM, 59 equiv.) and 2,6-di-*tert*-butylpyridine (2.3 mM, 1.4 equiv.) in $MeNO_2$-d_3:MeCN-d_3:DCM-d_2 (636 µL, 1.4:1:9).

The catalysis sample was monitored using ^{1}H NMR spectroscopy, with a spectrum collected every 5 min. After 2 hours the sample was removed from the NMR instrument and irradiated with an LED centred at 530 nm for 10 min. The sample was immediately placed back in the NMR instrument to avoid exposure to ambient light and was monitored for an additional 2 hours. The sample was then removed from the NMR instrument again, irradiated with 424 nm light for 10 min, immediately placed back in the NMR instrument. This irradiation cycle and ^{1}H NMR catalysis measurements were repeated 3 more times every two hours. After the final irradiation with 530 nm light for 10 min, the catalysis was monitored for additional 10 hours. The generation of the nitroaldol product was determined by measuring the signal intensity for the 4-nitrobenzaldehyde signal at 10.16 ppm ppm and the nitroaldol product signal at 5.62 ppm (for details see section 10.2.2). The signal intensities were plot against time to estimate how the rate of product formation was affected upon irradiation with 530 nm and 424 nm light (Figure S136).

After the first irradiation period with 424 nm light we could observer slow catalysis. We propose that the slow catalysis in this solvent mixture is caused by remaining [Pd_2(*Z*-**1**)$_2$(**2**)$_2$](BAr_F)$_4$ from incomplete switching and from [Pd_3(**2**)$_6$](BAr_F)$_6$. After irradiation with 530 nm light, [Pd_2(*Z*-**1**)$_2$(**2**)$_2$](BAr_F)$_4$ reassembled quantitatively and rapid catalysis was observed. This change in rate of catalysis could be observed over the next four switching cycles showing no fatigue.

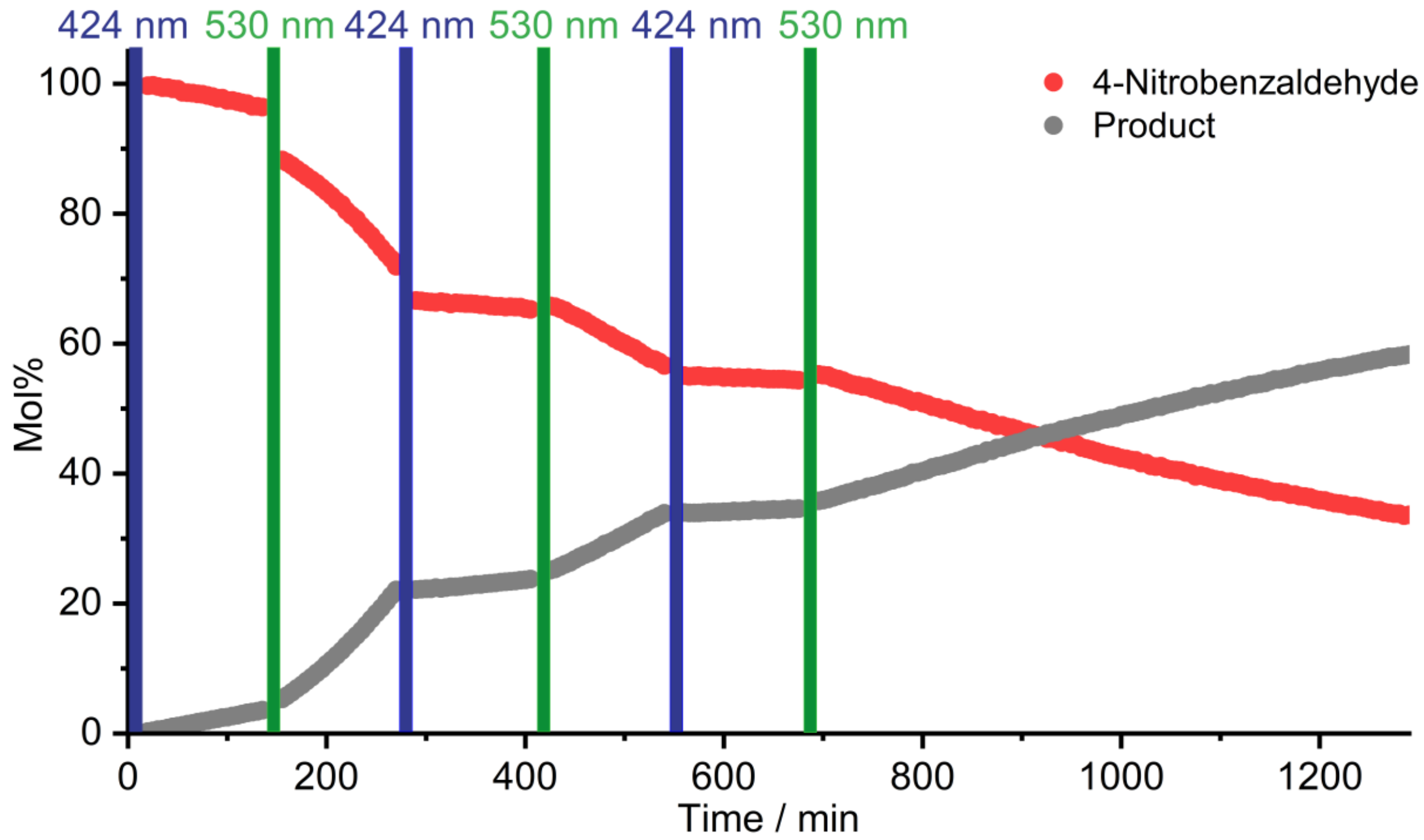

Figure S136. Monitoring switchable nitroaldol catalysis within heteroleptic cage [Pd_2(Z-**1**)$_2$(**2**)$_2$](BAr_F)$_4$. ^{1}H NMR (500 MHz, 298 K, $MeNO_2$-d_3:MeCN-d_3:DCM-d_2 1.4:1:9) signal intensities of 4-nitrobenzaldehyde and nitroaldol product following the reaction between 4-nitrobenzaldehyde (96 mM, 59 equiv.) and nitromethane-d_3 (used as a solvent $MeNO_2$-d_3:DCM-d_2 1:8) in the presence of 2,6-di-*tert*-butylpyridine (2.3 mM, 1.4 equiv.) and [Pd_2(Z-**1**)$_2$(**2**)$_2$](BAr_F)$_4$ ([Pd] = 1.6 mM, 1.0 equiv.). The integral of the signal at 10.16 ppm was used to monitor the abundance of 4-nitrobenzaldehyde and the integral of the signal at 5.62 ppm was used to monitor the abundance of the nitroaldol product. Relaxation delay (Bruker setting D1) was set to 5 seconds. The green vertical line indicates when the sample was irradiated with 530 nm light. The violet vertical line indicates when the sample was irradiated with 424 nm light.

We could observe efficient switching between the different catalytically active species. However, after the first switching cycle a rapid drop of the 4-nitrobenzaldehyde signal intensity was observed. We propose that this mismatch between decrease of starting materials signal and the increase of the product signal is due to different relaxation times of the chosen proton signals on the NMR timescale. The experiment was therefore repeated with a longer relaxation delay of 25 seconds (Bruker setting D1) which led to a better correlation between the signal intensity of the starting material 4-nitrobenzaldehyde and the product (Figure S137)

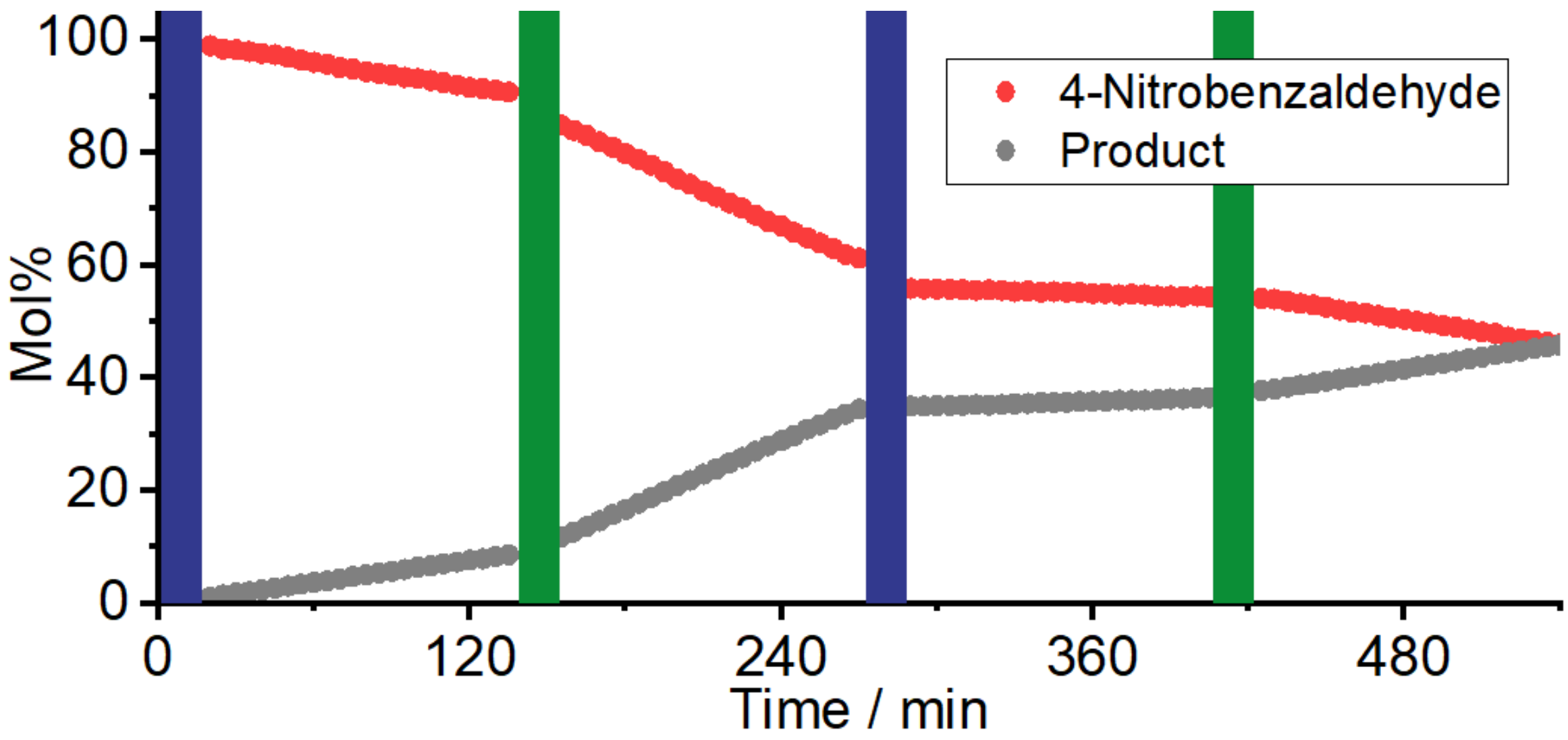

Figure S137. Monitoring switchable nitroaldol catalysis within heteroleptic cage $[Pd_2(Z\text{-}\mathbf{1})_2(\mathbf{2})_2](BAr_F)_4$. ^{1}H NMR (500 MHz, 298 K, $MeNO_2$-d_3:MeCN-d_3:DCM-d_2 1.4:1:9) signal intensities of 4-nitrobenzaldehyde and nitroaldol product following the reaction between 4-nitrobenzaldehyde (96 mM, 59 equiv.) and nitromethane-d_3 (used as a solvent $MeNO_2$-d_3:DCM-d_2 1:8) in the presence of 2,6-di-*tert*-butylpyridine (2.3 mM, 1.4 equiv.) and $[Pd_2(Z\text{-}\mathbf{1})_2(\mathbf{2})_2](BAr_F)_4$ ([Pd] = 1.6 mM, 1.0 equiv.). The integral of the signal at 10.16 ppm was used to monitor the abundance of 4-nitrobenzaldehyde and the integral of the signal at 5.62 ppm was used to monitor the abundance of the nitroaldol product. Relaxation delay (Bruker setting D1) was set to 25 seconds. The green vertical line indicates when the sample was irradiated with 530 nm light. The violet vertical line indicates when the sample was irradiated with 424 nm light.

11 Diffusion NMR measurements

11.1 Gradient calibration

The gradient strength used for diffusion NMR experiments is dependent on the geometry of the sample, among other things, and needs to be calibrated using the known diffusion coefficient of the residual protons in D_2O at 298 K (1.93×10^{-9} m^2 s^{-1}).[10] The water calibration was performed before data collection and the nominal *b* values were corrected by the resulting calibration factor of 1.09605.

The Bruker Avance III 400 MHz instrument with a Prodigy CryoProbe used the diff5 command with the Bruker diffSte pulsed-gradient stimulated echo (PGSTE) pulse sequence, with gradient length δ and echo delay Δ as specified in each experiment, including a spoiler gradient (followed by a 1.5 s recovery) to destroy magnetization remaining from the gradient pulse. Sine-shaped gradient pulses were used, typically ranging from 2-53.5 G cm^{-1}.

Using the standard Stejskal–Tanner equation:[11]

$$I = I_0 \exp\left[-D\gamma^2 g^2 \delta^2 \left(\Delta - \frac{1}{3}\delta\right)\right] = I_0 \exp[-Db]$$

where I_0 is the NMR signal intensity in the absence of the applied gradient, γ is the gyromagnetic ratio of the nucleus, g and δ describe the amplitude and duration of the magnetic field gradient pulse, respectively, and the delay Δ defines the timescale over which diffusion is measured.

Estimates of D and I_0 are determined by nonlinear regression of Stejskal–Tanner equation onto the signal integral data.

Samples of the homoleptic switchable cage $[Pd_3(E\text{-}\mathbf{1})_6](BAr_F)_6$, homoleptic switchable cage mixture $[Pd_3(E\text{-}\mathbf{1})_6](BF_4)_6$ and $[Pd_4(E\text{-}\mathbf{1})_8](BF_4)_8$, homoleptic non-switchable cage $[Pd_3(\mathbf{2})_6](BArF)_6$ and heteroleptic metastable cage $[Pd_2(E\text{-}\mathbf{1})_2(\mathbf{2})_2](BAr_F)_4$, all with the same concentration and volume (2.5 mM, 500 μL) in MeCN-d_3 were prepared.

11.2 Diffusion measurements for $[Pd_3(E\text{-}\mathbf{1})_6](BAr_F)_6$

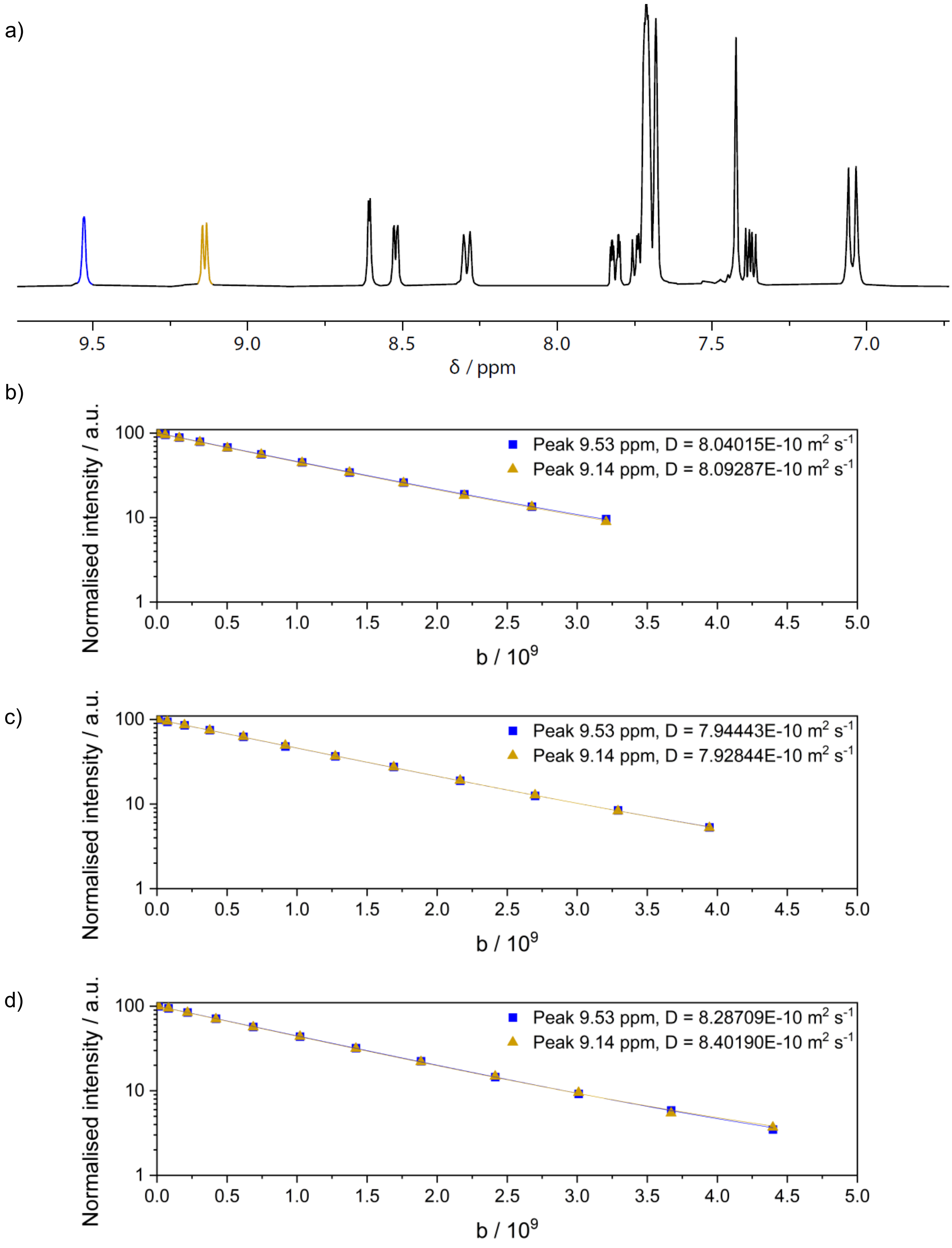

Figure S138. a) ^{1}H NMR spectrum (400 MHz, 298 K,MeCN-d_3) of $[Pd_3(E\text{-}\mathbf{1})_6](BAr_F)_6$, b) Fitting of integrals from ^{1}H NMR diffusion experiment (ste, δ = 1.8 ms, Δ = 45.0 ms), c) Fitting of integrals from ^{1}H NMR diffusion experiment (ste, δ = 2.0 ms, Δ = 45.0 ms), and d) Fitting of integrals from ^{1}H NMR diffusion experiment (ste, δ = 2.0 ms, Δ = 50.0 ms). Data were fit to a monoexponential using corrected gradient values ($G_{corrected} = G_{app} \times 1.09605$).

11.3 Diffusion measurements of the mixture of [Pd_3(*E*-1)$_6$](BF_4)$_6$ and [Pd_4(*E*-1)$_8$](BF_4)$_8$

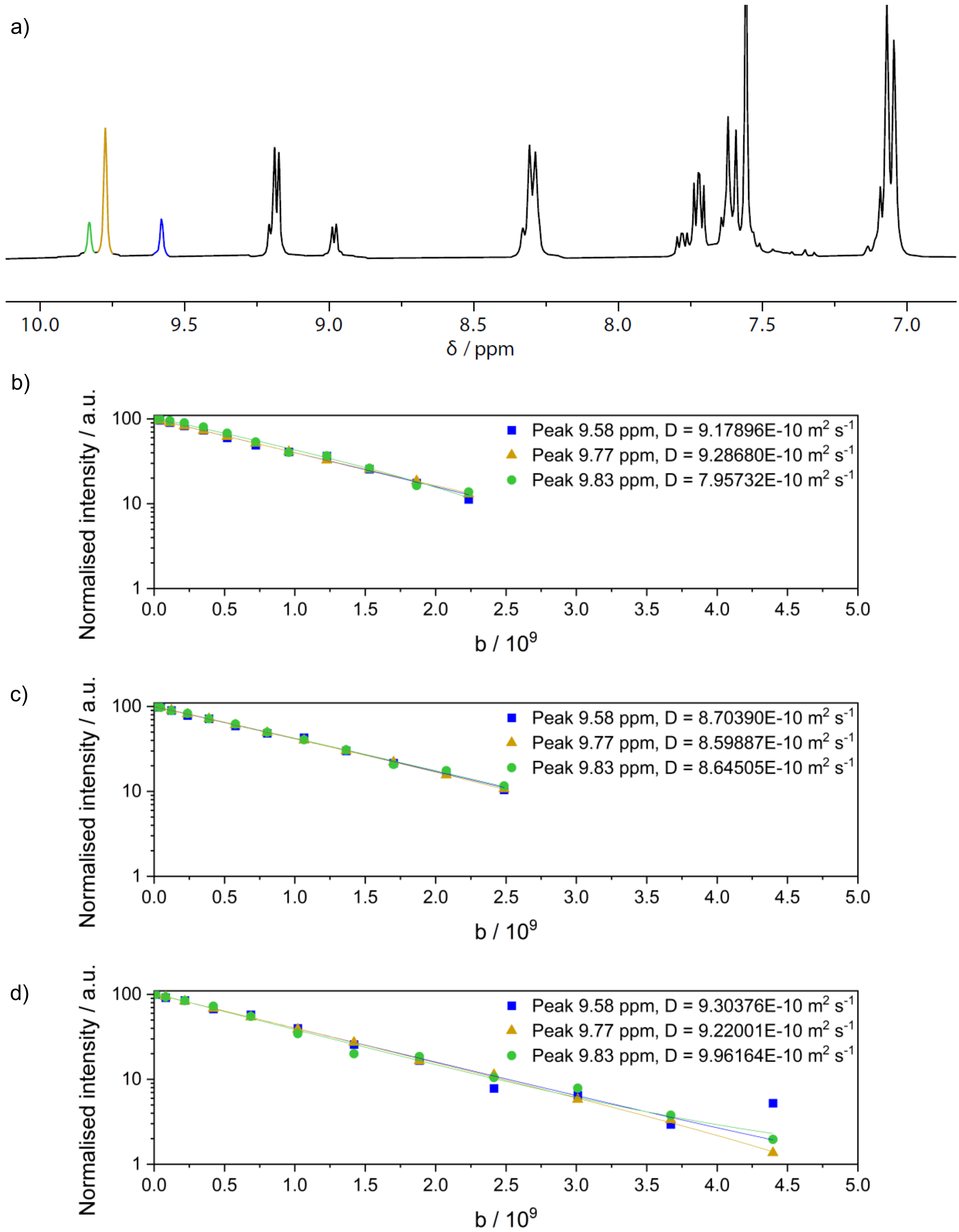

Figure S139. a) ^{1}H NMR spectrum (400 MHz, 298 K, MeCN-*d*$_3$) of the mixture of [Pd_3(*E*-**1**)$_6$](BF_4)$_6$ and [Pd_4(*E*-**1**)$_8$](BF_4)$_8$, b) Fitting of integrals from ^{1}H NMR diffusion experiment (ste, δ = 1.5 ms, Δ = 45.0 ms), c) Fitting of integrals from ^{1}H NMR diffusion experiment (ste, δ = 1.0 ms, Δ = 50.0 ms), and d) Fitting of integrals from ^{1}H NMR diffusion experiment (ste, δ = 2.0 ms, Δ = 55.0 ms). Data were fit to a monoexponential using corrected gradient values ($G_{corrected} = G_{app} \times 1.09605$).

11.4 Diffusion measurements for $[Pd_2(Z\text{-}1)_2(2)_2](BAr_F)_4$

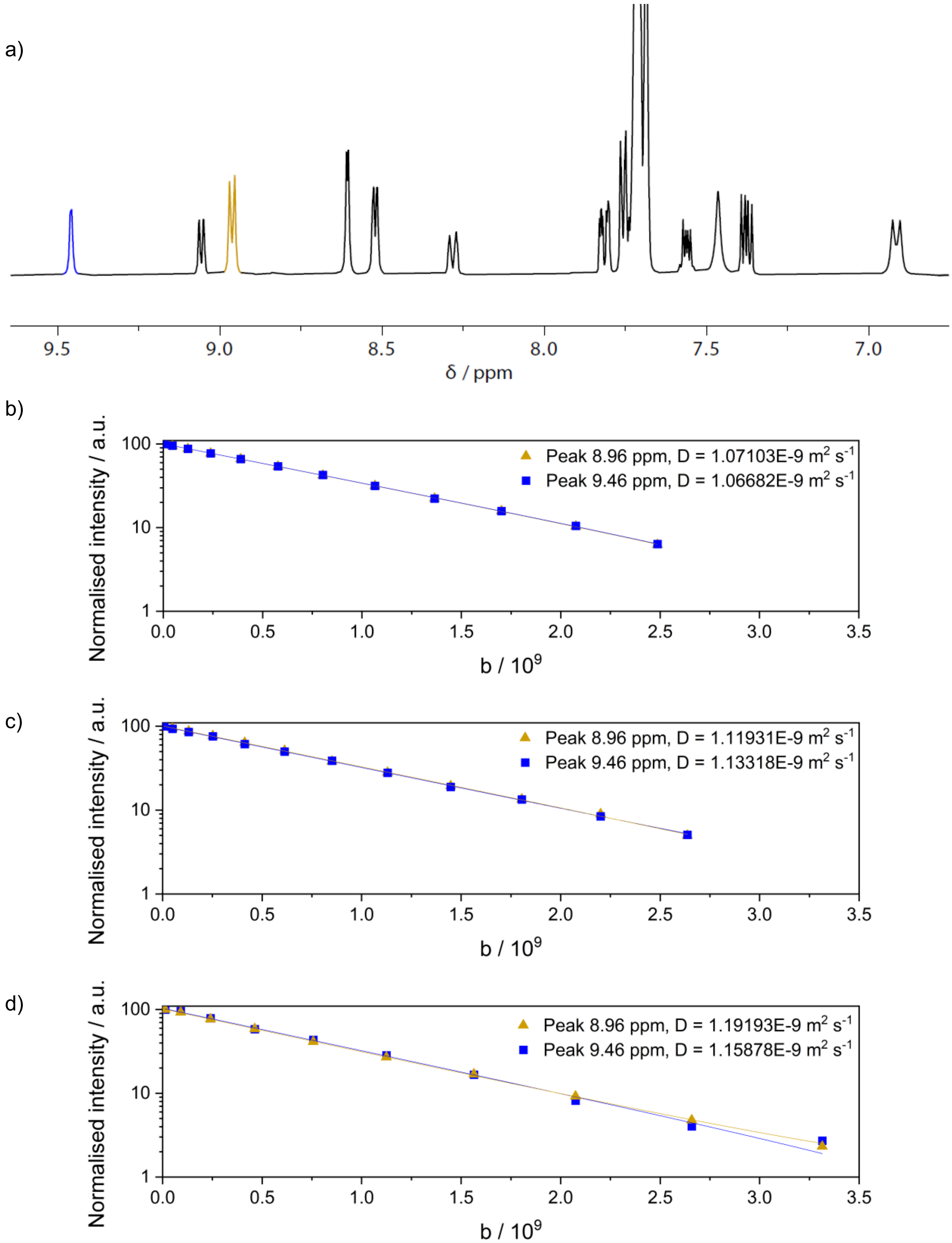

Figure S140. a) ^{1}H NMR spectrum (400 MHz, 298 K, MeCN-d_3) of $[Pd_2(Z\text{-}\mathbf{1})_2(\mathbf{2})_2](BAr_F)_4$, b) Fitting of integrals from ^{1}H NMR diffusion experiment (ste, δ = 1.5 ms, Δ = 50.0 ms), c) Fitting of integrals from ^{1}H NMR diffusion experiment (ste, δ = 1.5 ms, Δ = 53.0 ms), and d) Fitting of integrals from ^{1}H NMR diffusion experiment (ste, δ = 2.0 ms, Δ = 55.0 ms). Data were fit to a monoexponential using corrected gradient values (*G*corrected = *Gapp* × 1.09605).

11.5 Diffusion measurements for $[Pd_3(2)_6](BAr_F)_6$

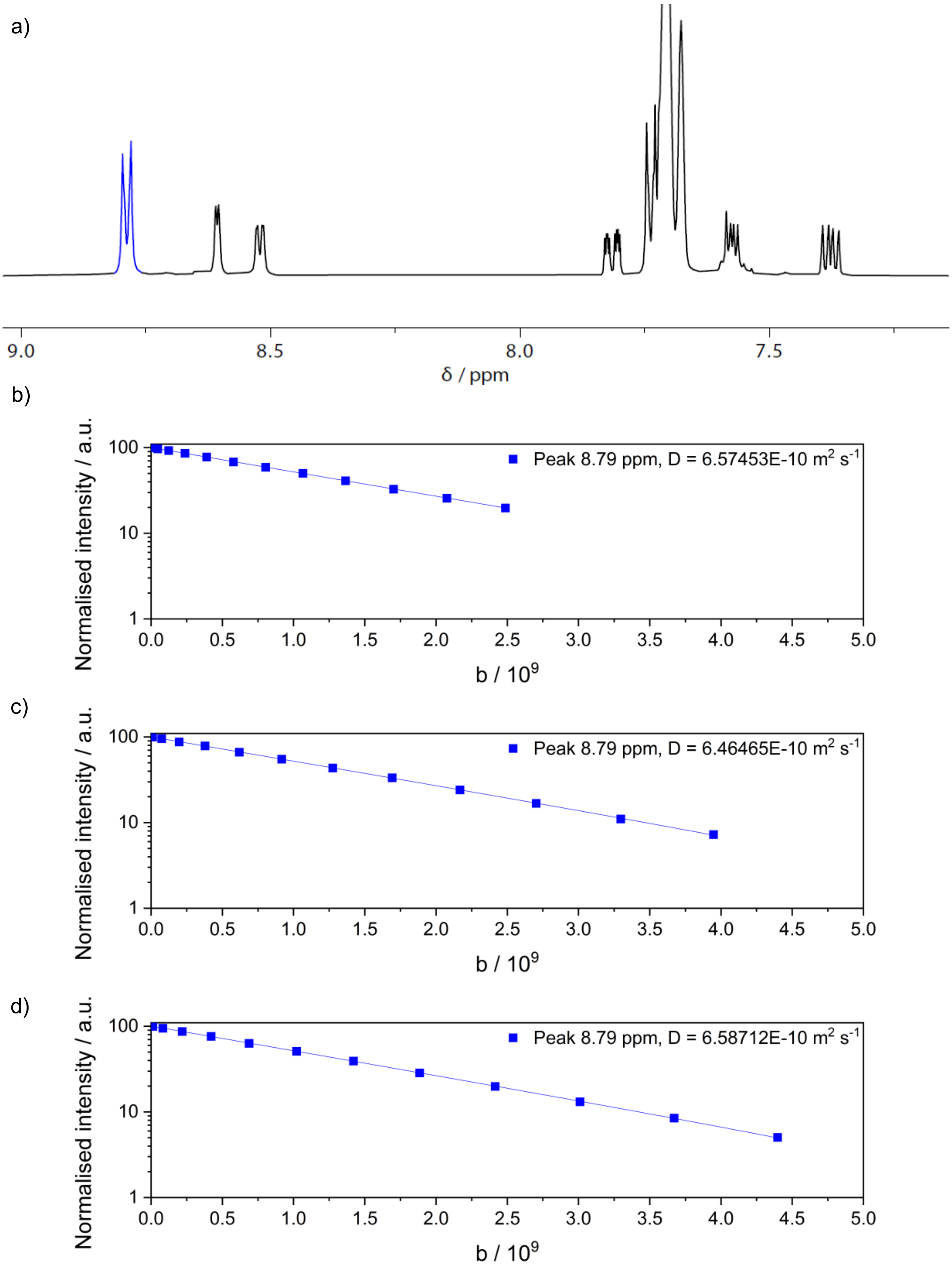

Figure S141. a) ^{1}H NMR spectrum (400 MHz, 298 K, MeCN-d_3) of $[Pd_3(\mathbf{2})_6](BAr_F)_6$, b) Fitting of integrals from ^{1}H NMR diffusion experiment (ste, δ = 1.5 ms, Δ = 40.0 ms), c) Fitting of integrals from ^{1}H NMR diffusion experiment (ste, δ = 2.0 ms, Δ = 45.0 ms), and d) Fitting of integrals from ^{1}H NMR diffusion experiment (ste, δ = 2.0 ms, Δ = 50.0 ms). Data were fit to a monoexponential using corrected gradient values ($G_{corrected} = G_{app} \times 1.09605$).

11.6 Summary Diffusion measurements

Table S28. Summary of ^{1}H diffusion NMR (400 MHz, 298 K, MeCN-d_3) experiments for $[Pd_3(E\text{-}\mathbf{1})_6](BAr_F)_6$ at 298 K.

δ, Δ	$[Pd_3(E\text{-}1)_6](BAr_F)_6$	
	D / 10^{-10} m^2 s^{-1} For the peak at 1.53 ppm	D / 10^{-10} m^2 s^{-1} For the peak at 1.14 ppm
1.8 ms, 45.0 ms	8.04	8.09
2.0 ms, 45.0 ms	7.94	7.93
2.0 ms, 50.0 ms	8.29	8.40
**D / 10^{-10} m^2 s^{-1}**	**8.12 ± 0.11**	
**Hydrodynamic radii / Å**	**7.88 ± 0.11**	

Table S29. Summary of ^{1}H diffusion NMR (400 MHz, 298 K, MeCN-d_3) experiments for the mixture of $[Pd_3(E\text{-}\mathbf{1})_6](BF_4)_6$ and $[Pd_4(E\text{-}\mathbf{1})_8](BF_4)_8$ at 298 K.

δ, Δ	$[Pd_4(E\text{-}1)_8](BF_4)_8$	$[Pd_4(E\text{-}1)_8](BF_4)_8$	$[Pd_3(E\text{-}1)_6](BF_4)_6$
	D / 10^{-10} m^2 s^{-1} For the peak at 9.83 ppm	D / 10^{-10} m^2 s^{-1} For the peak at 9.58 ppm	D / 10^{-10} m^2 s^{-1} For the peak at 9.77 ppm
1.0 ms, 50.0 ms	8.65	8.70	8.60
1.5 ms, 45.0 ms	7.96	9.18	9.29
2.0 ms, 55.0 ms	9.96	9.30	9.22
**D / 10^{-10} m^2 s^{-1}**	**8.96 ± 0.68**		**9.04 ± 0.38**
**Hydrodynamic radii / Å**	**7.14 ± 0.59**		**7.08 ± 0.31**

Table S30. Summary of ^{1}H diffusion NMR (400 MHz, 298 K, MeCN-d_3) experiments for $[Pd_2(Z\text{-}\mathbf{1})_2(\mathbf{2})_2](BAr_F)_4$ at 298 K.

δ, Δ	$[Pd_2(Z\text{-}1)_2(2)_2](BAr_F)_4$	
	D / 10^{-9} m^2 s^{-1} For the peak at 8.96 ppm	D / 10^{-9} m^2 s^{-1} For the peak at 9.46 ppm
1.5 ms, 55.0 ms	1.07	1.07
1.5 ms, 53.0 ms	1.13	1.12
2.0 ms, 55.0 ms	1.16	1.19
**D / 10^{-9} m^2 s^{-1}**	**1.12 ± 0.05**	
**Hydrodynamic radii / Å**	**5.72 ± 0.26**	

Table S31. Summary of ^{1}H diffusion NMR (400 MHz, 298 K, MeCN-d_3) experiments for $[Pd_3(\mathbf{2})_6](BAr_F)_6$ at 298 K.

**δ, Δ**	**$[Pd_3(2)_6](BAr_F)_6$**<br>**D / 10^{-10} m^2 s^{-1}**<br>**For the peak at 8.79 ppm**
1.5 ms, 40.0 ms	6.57
2.0 ms, 45.0 ms	6.46
2.0 ms, 50.0 ms	6.59
**D / 10^{-10} m^2 s^{-1}**	**6.74 ± 0.07**
**Hydrodynamic radii / Å**	**9.49 ± 0.11**

12 Spartan models of $[Pd_3(E\text{-}1)_6]^{6+}$, $[Pd_4(E\text{-}1)_8]^{8+}$, $[Pd_3(E\text{-}1)_4(2)_2]^{6+}$, $[Pd_4(E\text{-}1)_4(2)_4]^{8+}$ and $[Pd_2(Z\text{-}1)_2(2)_2]^{4+}$

We used Spartan Student v9, Version 9.0.3 to create plausible models for the observed cages $[Pd_3(E\text{-}\mathbf{1})_6]^{6+}$, $[Pd_4(E\text{-}\mathbf{1})_8]^{8+}$, $[Pd_3(E\text{-}\mathbf{1})_4(\mathbf{2})_2]^{6+}$, $[Pd_4(E\text{-}\mathbf{1})_4(\mathbf{2})_4]^{8+}$ and $[Pd_2(Z\text{-}\mathbf{1})_2(\mathbf{2})_2]^{4+}$ using simple molecular mechanics only. The *cis*-N-Pd-N bond angles were all restricted to 90 degrees and *trans*-N-Pd-N are restricted to 180 degrees as these angles are rarely deviate from ideal. The torsion angles of the C-N=N-C on each side of the azo bonds were restricted in cages where ligand **1** is present in its *E*-form to enforce co-planarity of the rings on each side, similar to that observed in the X-ray structure of *E*-**1**.

All cage models show a perpendicular arrangement of the *ortho*-methylated arene spacer with respect to the azobenzene unit, in accordance with the observations in the X-ray structures of both, *E*-**1** and *Z*-**1**.

The models of the double-walled triangle $[Pd_3(E\text{-}\mathbf{1})_6]^{6+}$ and the distorted tetrahedron $[Pd_4(E\text{-}\mathbf{1})_8]^{8+}$ are similar to the models of a related ligand.[4] The model of heteroleptic $[Pd_4(E\text{-}\mathbf{1})_4(\mathbf{2})_4]^{8+}$ shows a twisted helicate structure, similar to that of related homoleptic cages.[2, 9] The simple molecular mechanics model of the heteroleptic metastable cage $[Pd_2(Z\text{-}\mathbf{1})_2(\mathbf{2})_2]^{4+}$ has a structure similar to that of the X-ray structure of $[Pd_2(Z\text{-}\mathbf{1})_2(\mathbf{2})_2]^{4+}$, suggesting these simple models could be reasonable approximations for the structures of these cages.

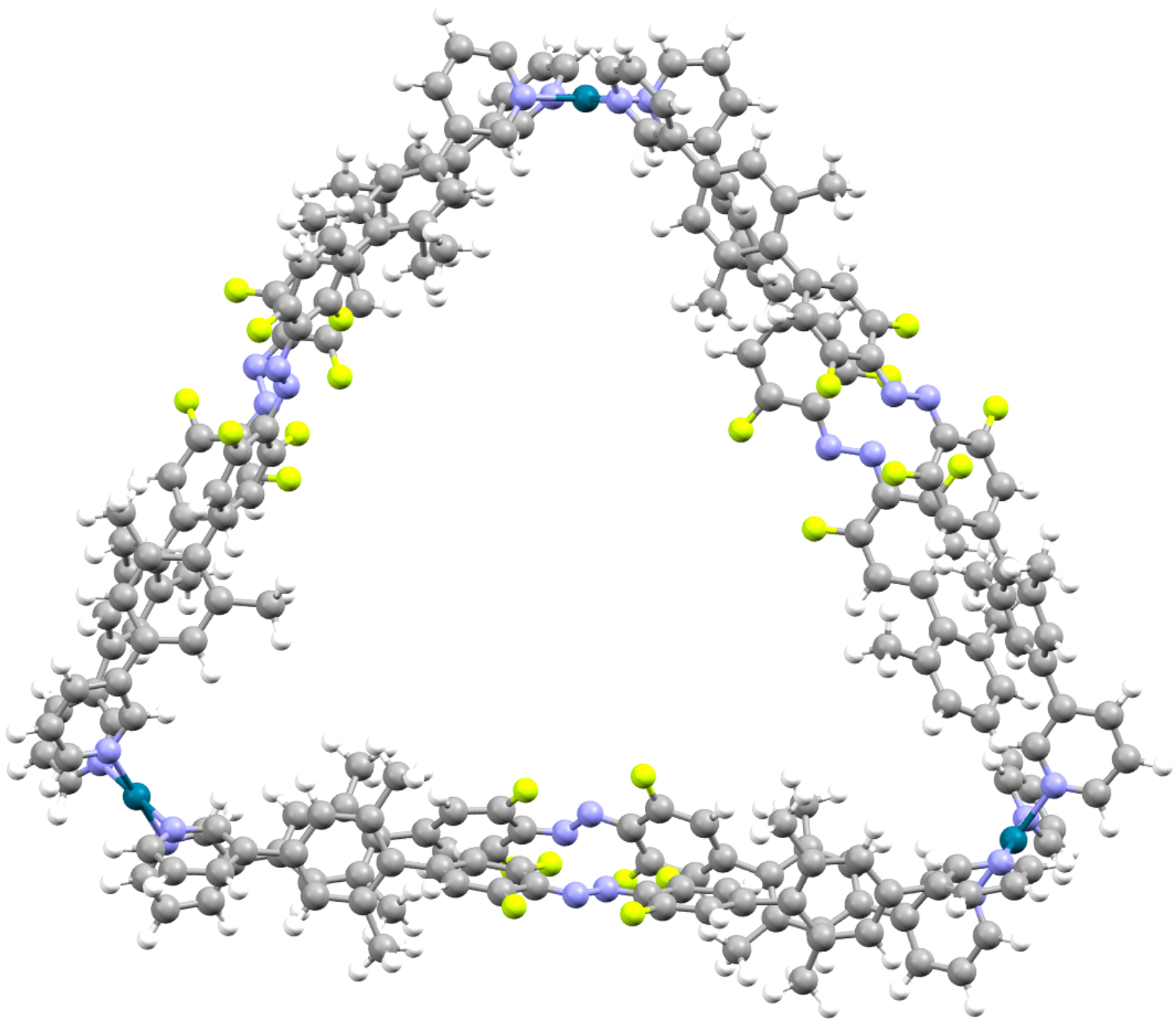

Figure S142. Simple molecular mechanics model of homoleptic $[Pd_3(E\text{-}\mathbf{1})_6]^{6+}$.

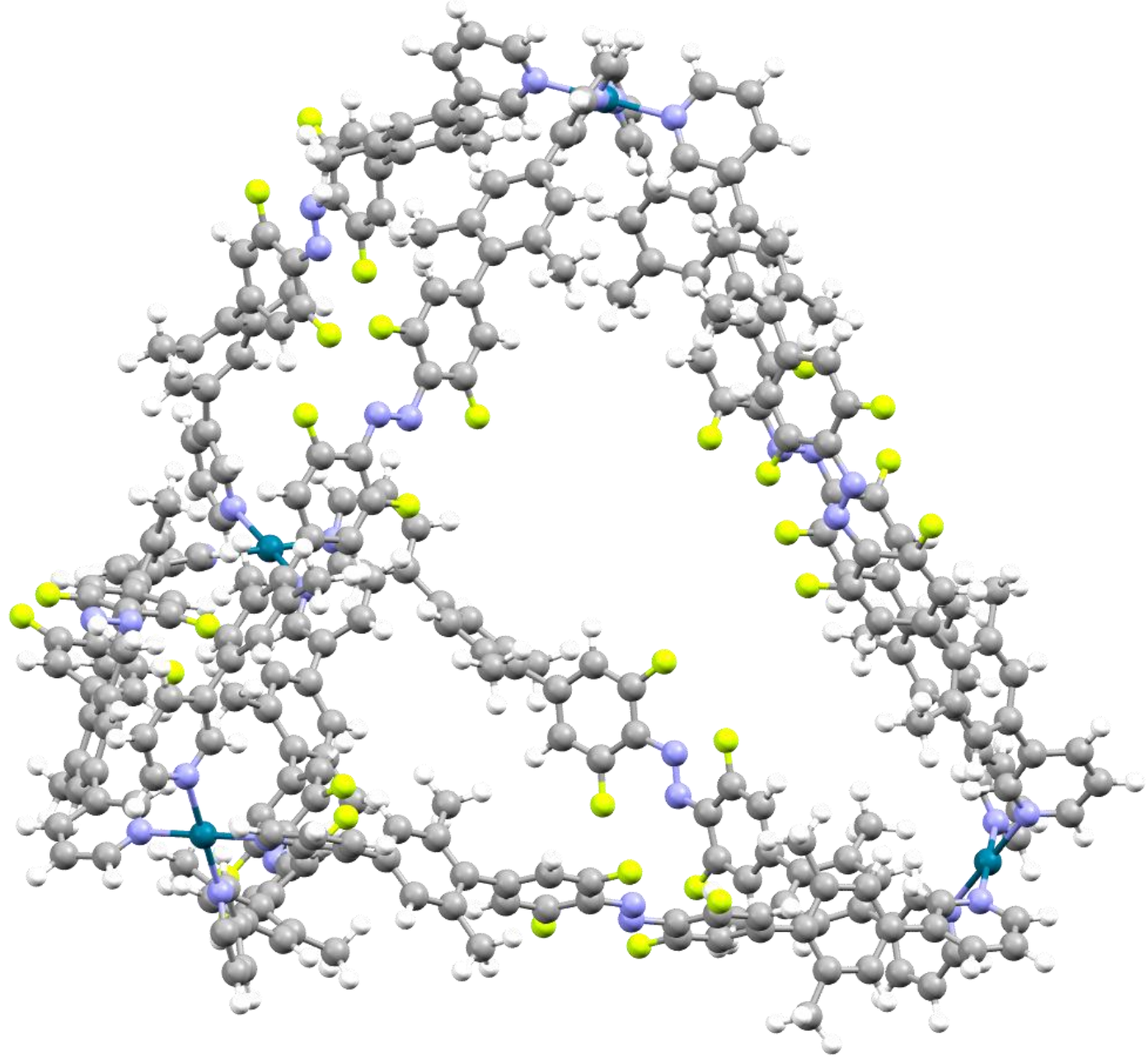

Figure S143. Simple molecular mechanics model of homoleptic $[Pd_4(E\text{-}\mathbf{1})_8]^{8+}$.

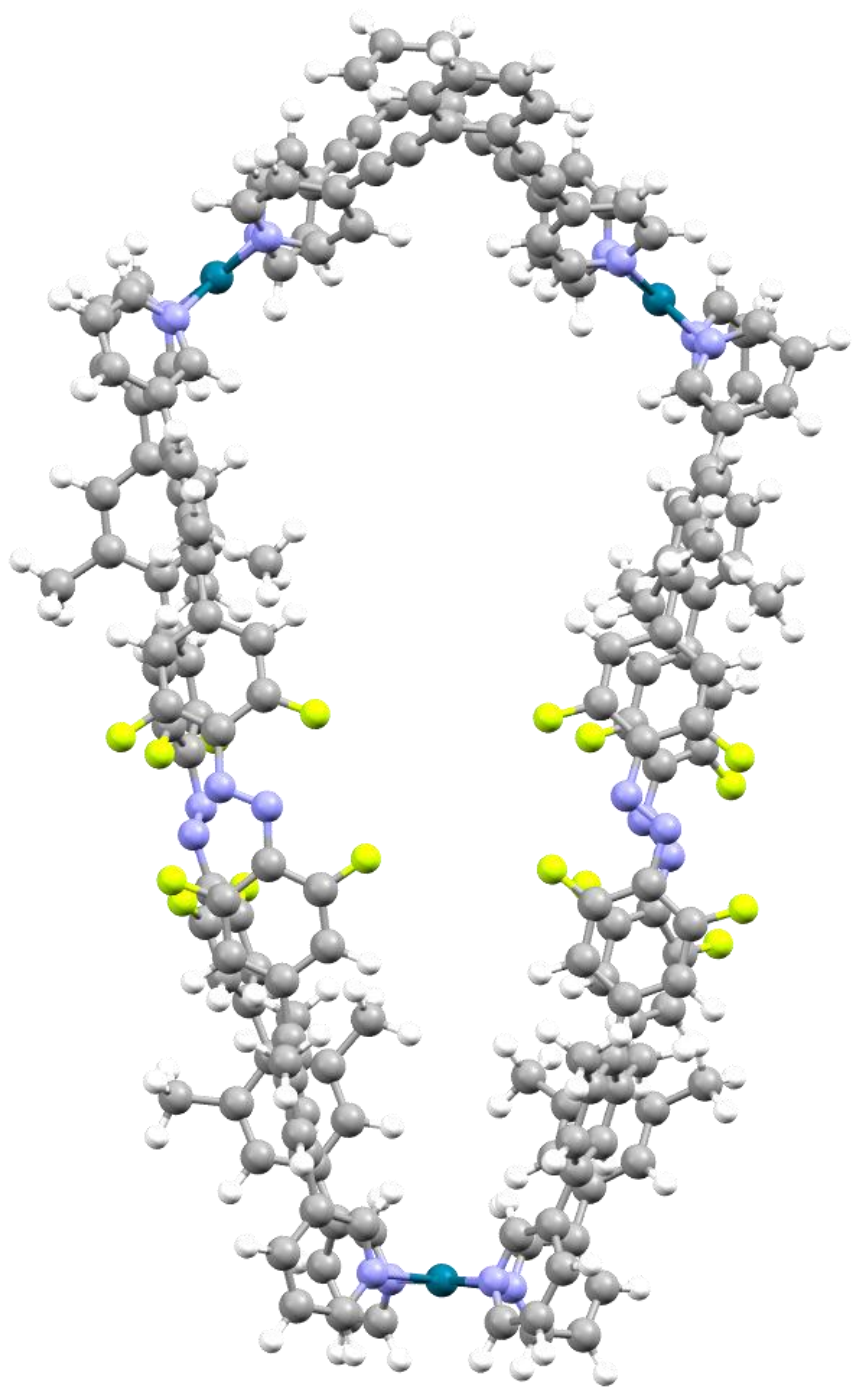

Figure S144. Simple molecular mechanics model of heteroleptic $[Pd_3(E\text{-}\mathbf{1})_4(\mathbf{2})_2]^{6+}$.

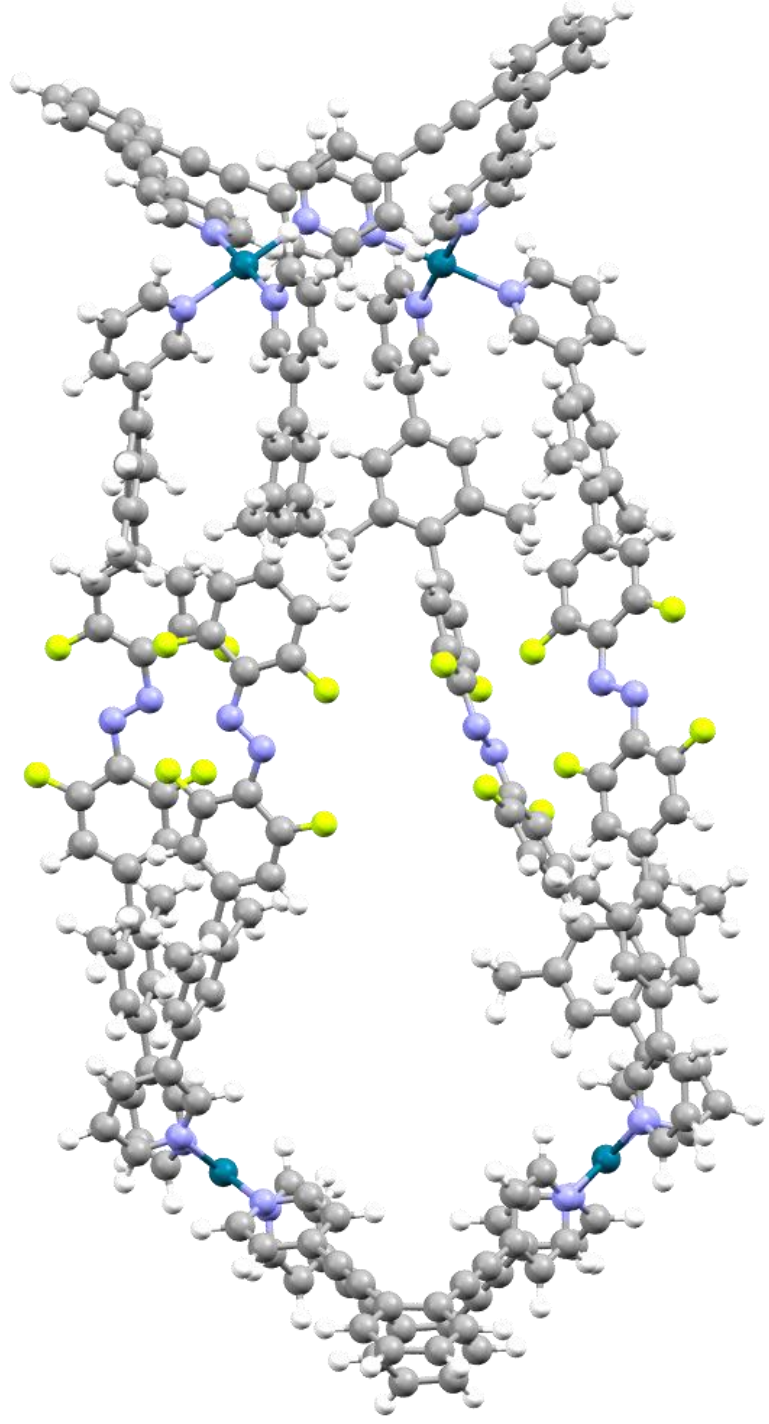

Figure S145. Simple molecular mechanics model of heteroleptic [Pd_4(*E*-**1**)$_4$(**2**)$_4$]$^{8+}$.

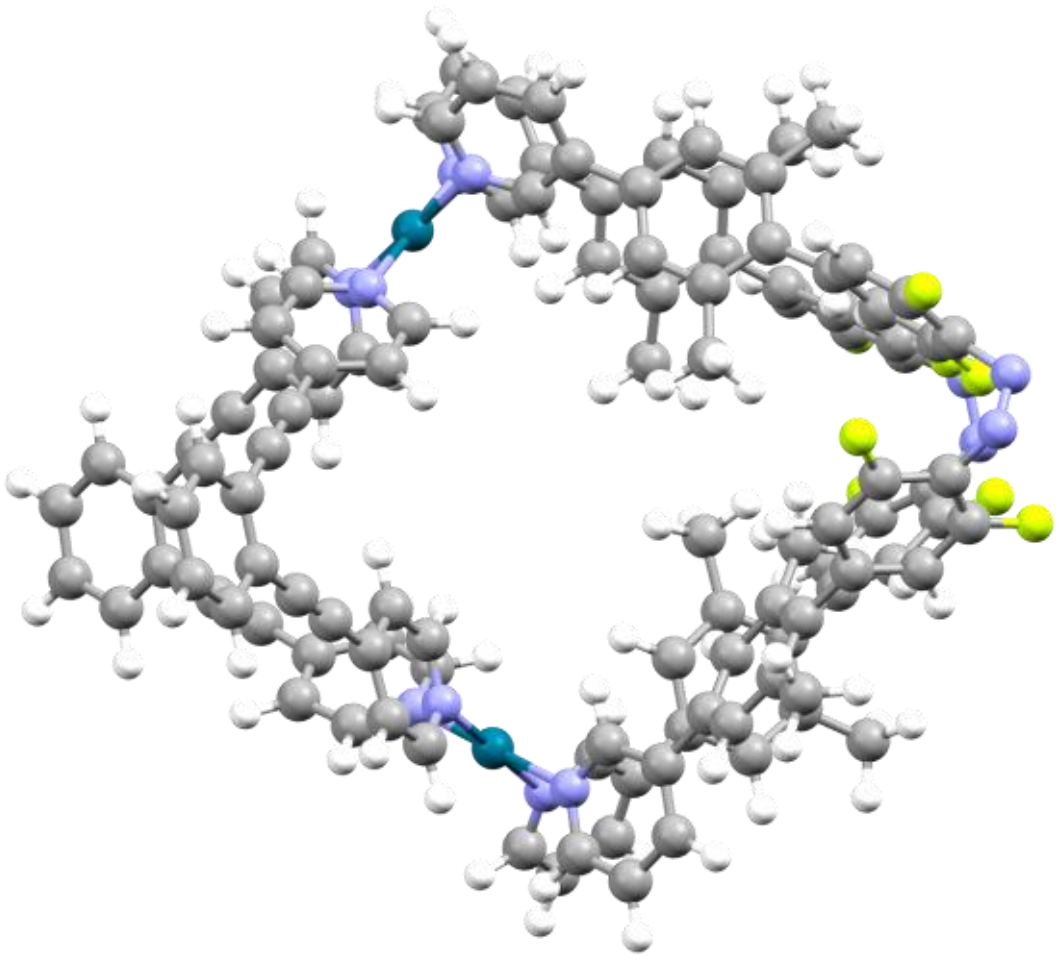

Figure S146. Simple molecular mechanics model of heteroleptic [Pd_2(*Z*-**1**)$_2$(**2**)$_2$]$^{4+}$.

13 Single crystal X-ray structures

13.1 Single crystal X-ray structure of S2 (CCDC: 2552325)

Crystals suitable for X-ray crystallography were grown by the slow evaporation of a solution of **S2** in acetonitrile. A plate-like red single crystal with dimensions of 0.25 × 0.18 × 0.20 mm was selected under a polarizing microscope (Leica M165Z), and was mounted on a MicroMount (MiTeGen, USA), consisting of a thin polymer tip with a wicking aperture. The X-ray diffraction measurements were carried out on a Bruker D8 Quest Single Crystal diffractometer with a Photon II detector at 120 K by using CuKα (λ = 1.54178 Å). The single crystal was coated with NVH immersion oil and then quickly transferred to the cold nitrogen stream using the Oxford Cryostream 800 series attachment and was mounted on the goniometer using a cryo loop for intensity measurement. The solutions were obtained by intrinsic phasing using SHELXT[12] followed by successive refinements using full-matrix least-squares method against F^2 using SHELXL[13] as implemented within OLEX2-1.5[14]. All non-hydrogen atoms were refined with anisotropic thermal parameters, with hydrogen atoms being added geometrically and refined using riding thermal parameters. The crystal structure of **S2** was refined as a two-component twin.

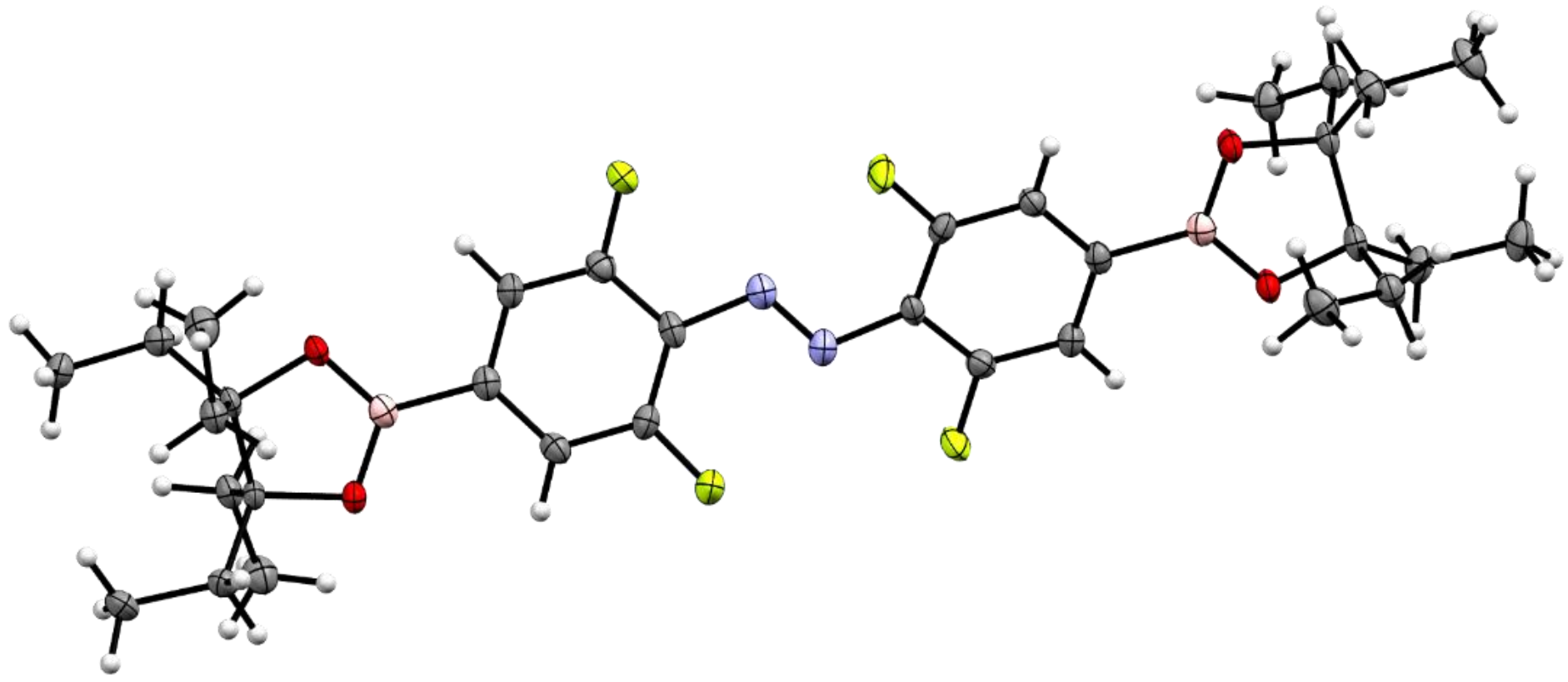

Figure S147. ORTEP type representation of intermediate **S2**. Ellipsoids drawn at 50% probability.

Table S32. Crystal data and structure refinement for **S2**.

Crystallographic details	**S2**
Empirical formula	$C_{32}H_{44}B_2F_4N_2O_4$
Formula weight	618.31
Temperature/K	120.00
Crystal system	triclinic
Space group	P-1
a/Å	10.0827(11)
b/Å	10.9205(12)
c/Å	15.7801(18)
α/°	105.754(4)
β/°	103.131(4)
γ/°	101.072(4)
Volume/Å^3	1567.7(3)
Z	2
ρ_{calc}g/cm^3	1.310
μ/mm^{-1}	0.835
F(000)	656.0
Crystal size/mm^3	0.25 × 0.18 × 0.02
Radiation	CuKα (λ = 1.54178)
2Θ range for data collection/°	6.092 to 145.474
Index ranges	-12 ≤ h ≤ 12, -13 ≤ k ≤ 13, -19 ≤ l ≤ 19
Reflections collected	44794
Independent reflections	44794 [R_{int} = ?, R_{sigma} = 0.0599]
Data/restraints/parameters	44794/0/406
Goodness-of-fit on F^2	1.066
Final R indexes [I>=2σ (I)]	R_1 = 0.0582, wR_2 = 0.1636
Final R indexes [all data]	R_1 = 0.0622, wR_2 = 0.1664
Largest diff. peak/hole / e Å^{-3}	0.73/-0.31

13.2 Single crystal X-ray structure of photoswitch 3 (CCDC: 2552326)

Crystals suitable for X-ray crystallography were grown by the slow evaporation of a solution of *E*-**3** in acetonitrile. A plate-like orange single crystal with dimensions of 0.32 × 0.20 × 0.20 mm was selected under a polarizing microscope (Leica M165Z), and was mounted on a MicroMount (MiTeGen, USA), consisting of a thin polymer tip with a wicking aperture. The X-ray diffraction measurements were carried out on a Bruker D8 Quest Single Crystal diffractometer with a Photon II detector at 120 K by using MoKα (λ = 0.71073 Å). The single crystal was coated with NVH immersion oil and then quickly transferred to the cold nitrogen stream using the Oxford Cryostream 800 series attachment and was mounted on the goniometer using a cryo loop for intensity measurement. The solutions were obtained by intrinsic phasing using SHELXT[12] followed by successive refinements using full-matrix least-squares method against F^2 using SHELXL[13] as implemented within OLEX2-1.5[14]. All non-hydrogen atoms were refined with anisotropic thermal parameters, with hydrogen atoms being added geometrically and refined using riding thermal parameters.

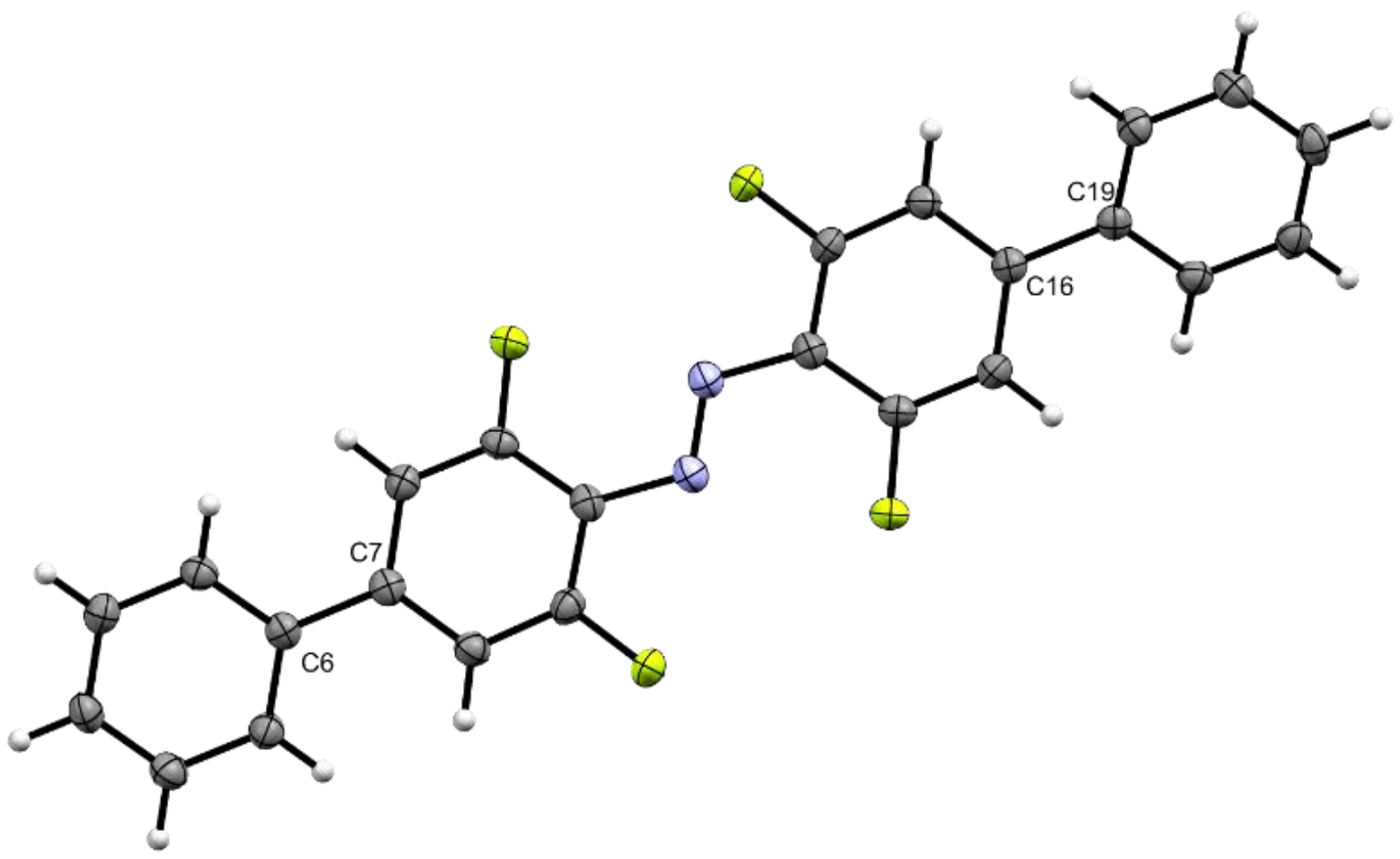

Figure S148. ORTEP type representation of photoswitch *E*-**3**. Ellipsoids drawn at 50% probability.

Table S33. Crystal data and structure refinement for *E*-**3**.

Crystallographic details	*E*-**3**
Empirical formula	$C_{24}H_{14}F_4N_2$
Formula weight	406.37
Temperature/K	120.00
Crystal system	monoclinic
Space group	C2/c
a/Å	14.718(3)
b/Å	10.487(2)
c/Å	23.765(5)
α/°	90
β/°	98.046(6)
γ/°	90
Volume/Å^3	3632.0(12)
Z	8
ρ_{calc}g/cm^3	1.486
μ/mm^{-1}	0.992
F(000)	1664.0
Crystal size/mm^3	0.32 × 0.2 × 0.2
Radiation	CuKα (λ = 1.54178)
2Θ range for data collection/°	10.392 to 145.428
Index ranges	-18 ≤ h ≤ 18, -12 ≤ k ≤ 12, -27 ≤ l ≤ 29
Reflections collected	50951
Independent reflections	3596 [R_{int} = 0.0385, R_{sigma} = 0.0181]
Data/restraints/parameters	3596/0/271
Goodness-of-fit on F^2	1.054
Final R indexes [I>=2σ (I)]	R_1 = 0.0323, wR_2 = 0.0884
Final R indexes [all data]	R_1 = 0.0327, wR_2 = 0.0888
Largest diff. peak/hole / e Å^{-3}	0.23/-0.19

Table S34. Angles between the planes of pairs of connected rings in photoswitch *E*-**3.**

Pairs of connected rings	Angle/°
Ring containing C6 and C7	31.1
Ring containing C16 and C19	28.1

13.3 Single crystal X-ray structure of photoswitch 4 (CCDC: 2552327)

Crystals suitable for X-ray crystallography were grown by the slow evaporation of a solution of *E*-**4** in acetonitrile and dichloromethane (~5:1). A plate-like orange single crystal with dimensions of 0.02 × 0.02 × 0.005 mm was selected under a polarizing microscope (Leica M165Z), and was mounted on a MicroMount (MiTeGen, USA), consisting of a thin polymer tip with a wicking aperture. The X-ray diffraction measurements were carried out on a Bruker D8 Quest Single Crystal diffractometer with a Photon II detector at 120 K by using MoKα (λ = 0.71073 Å). The single crystal was coated with NVH immersion oil and then quickly transferred to the cold nitrogen stream using the Oxford Cryostream 800 series attachment and was mounted on the goniometer using a cryo loop for intensity measurement. The solutions were obtained by intrinsic phasing using SHELXT[12] followed by successive refinements using full-matrix least-squares method against F^2 using SHELXL[13] as implemented within OLEX2-1.5[14].All non-hydrogen atoms were refined with anisotropic thermal parameters, with hydrogen atoms being added geometrically and refined using riding thermal parameters.

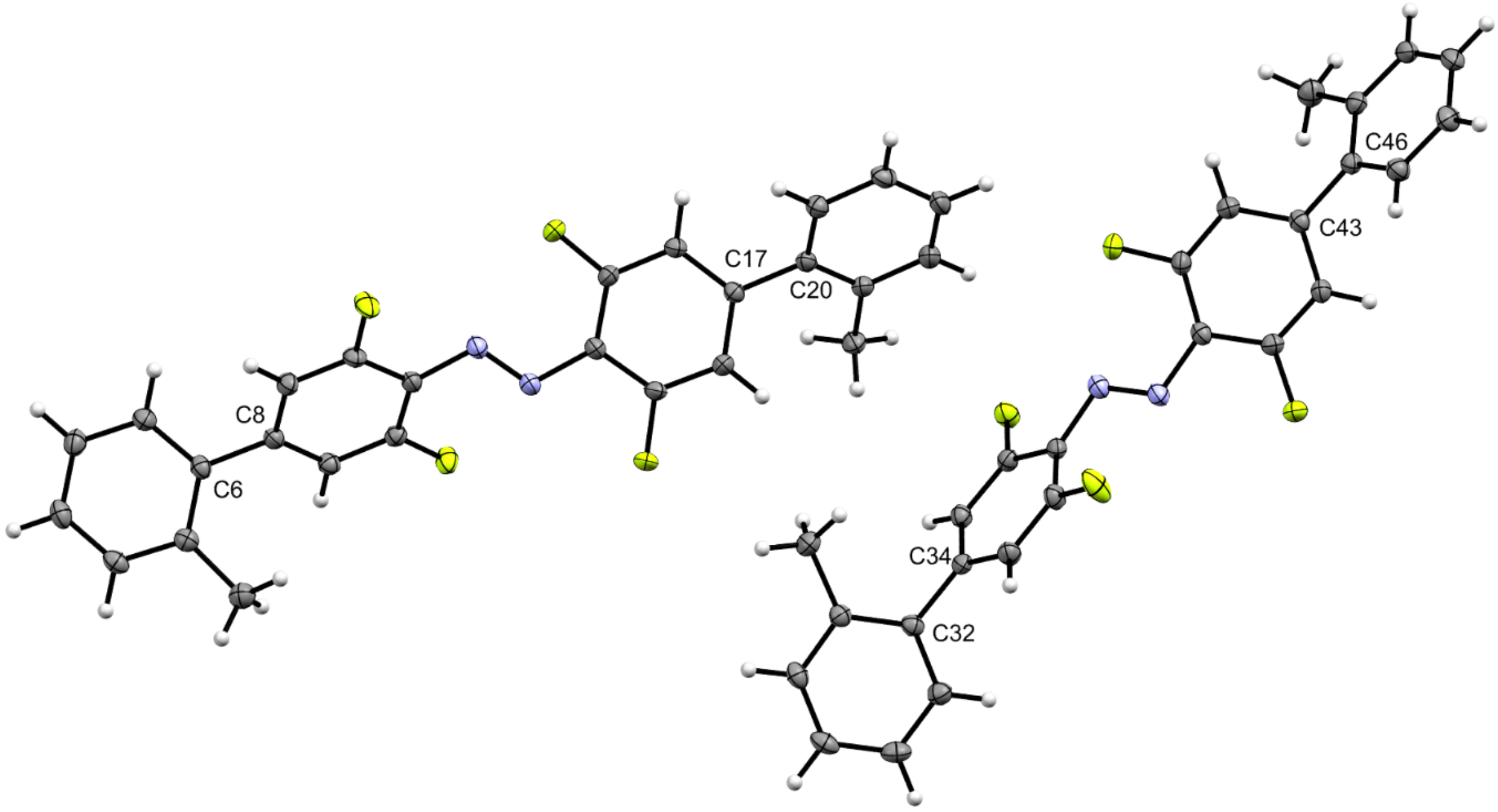

Figure S149. ORTEP type representation of the asymmetric unit of photoswitch *E*-**4**. Ellipsoids drawn at 50% probability.

Table S35. Crystal data and structure refinement for *E*-**4**.

Crystallographic details	*E*-**4**
Empirical formula	$C_{26}H_{18}F_4N_2$
Formula weight	434.42
Temperature/K	120.00
Crystal system	triclinic
Space group	P-1
a/Å	7.4808(3)
b/Å	13.6991(6)
c/Å	19.5939(9)
α/°	91.588(2)
β/°	90.426(2)
γ/°	93.512(2)
Volume/Å^3	2003.36(15)
Z	4
ρ_{calc}g/cm^3	1.440
μ/mm^{-1}	0.111
F(000)	896.0
Crystal size/mm^3	0.02 × 0.02 × 0.005
Radiation	MoKα (λ = 0.71073)
2Θ range for data collection/°	4.16 to 55.156
Index ranges	-9 ≤ h ≤ 9, -17 ≤ k ≤ 17, -25 ≤ l ≤ 25
Reflections collected	109543
Independent reflections	9267 [R_{int} = 0.0563, R_{sigma} = 0.0258]
Data/restraints/parameters	9267/0/581
Goodness-of-fit on F^2	1.044
Final R indexes [I>=2σ (I)]	R_1 = 0.0401, wR_2 = 0.1059
Final R indexes [all data]	R_1 = 0.0441, wR_2 = 0.1094
Largest diff. peak/hole / e Å^{-3}	0.48/-0.36

Table S36. Angles between the planes of pairs of connected rings in photoswitch *E*-**4.**

Pairs of connected rings	Angle/°
Ring containing C6 and C8	49.4
Ring containing C17 and C20	48.2
Ring containing C32 and C34	71.0
Ring containing C43 and C46	43.0

13.4 Single crystal X-ray structure of photoswitch 5 (CCDC: 2552328)

Crystals suitable for X-ray crystallography were grown by the slow evaporation of a solution of *E*-**5** in acetonitrile and dichloromethane (5:1). A block-like single crystal with dimensions of 0.58 × 0.13 × 0.13 mm was selected under a polarizing microscope (Leica M165Z), and was mounted on a MicroMount (MiTeGen, USA), consisting of a thin polymer tip with a wicking aperture. The X-ray diffraction measurements were carried out on a Bruker D8 Quest Single Crystal diffractometer with a Photon II detector at 120 K by using µS 3.0 Microfocus Source with Cu-Kα radiation (λ = 1.54178 Å). The single crystal was coated with NVH immersion oil and then quickly transferred to the cold nitrogen stream using the Oxford Cryostream 800 series attachment, and it was mounted on the goniometer using a cryo loop for intensity measurement. The solutions were obtained by intrinsic phasing using SHELXT[12] followed by successive refinements using full-matrix least-squares method against F^2 using SHELXL[13] as implemented within OLEX2-1.5[14]. All non-hydrogen atoms were refined with anisotropic thermal parameters, with hydrogen atoms being added geometrically and refined using riding thermal parameters.

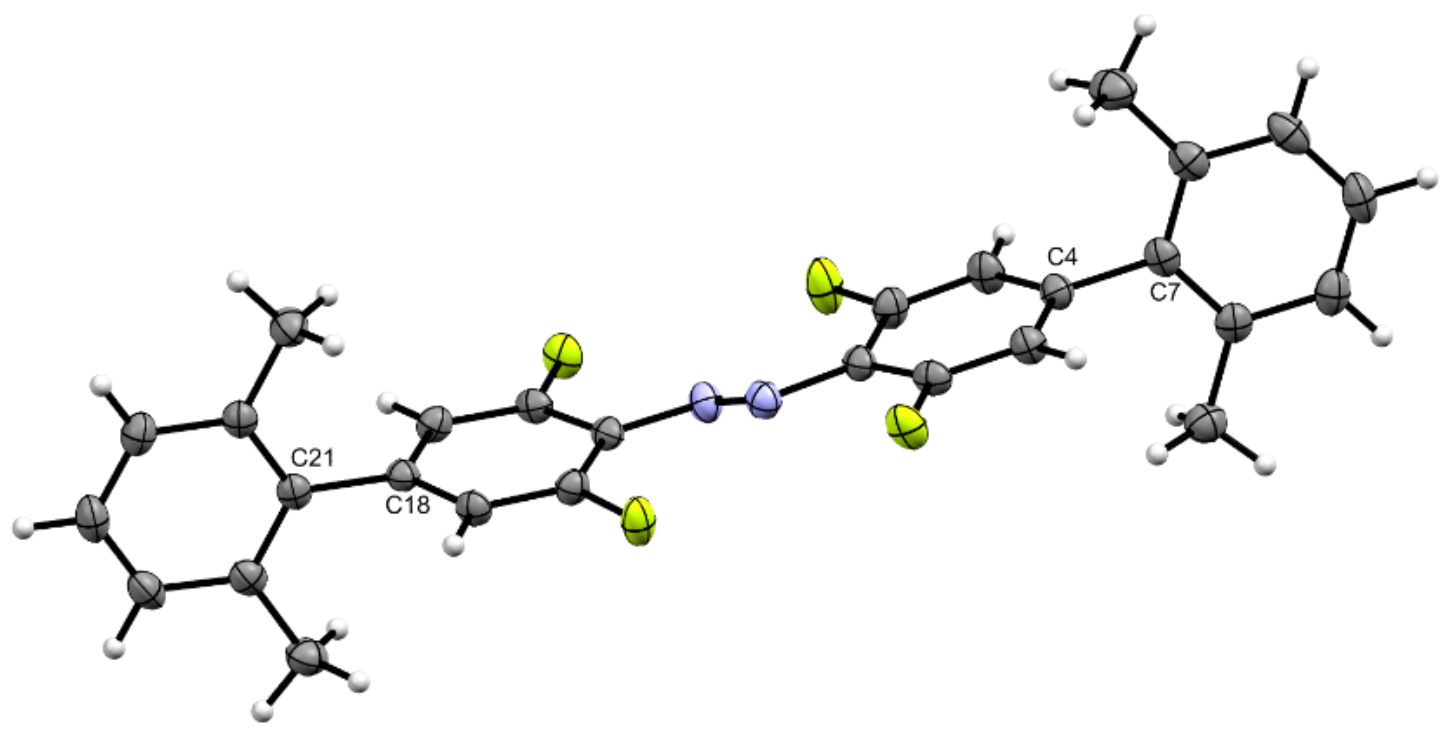

Figure S150. ORTEP type representation of photoswitch *E*-**5**. Ellipsoids drawn at 50% probability.

Table S37. Crystal data and structure refinement for *E*-**5**.

Crystallographic details	*E*-**5**
Empirical formula	$C_{28}H_{22}F_4N_2$
Formula weight	462.47
Temperature/K	120
Crystal system	orthorhombic
Space group	Pbca
a/Å	14.2474(8)
b/Å	15.9098(8)
c/Å	19.9670(11)
α/°	90
β/°	90
γ/°	90
Volume/Å^3	4526.0(4)
Z	8
ρ_{calc}g/cm^3	1.357
μ/mm^{-1}	0.861
F(000)	1920.0
Crystal size/mm^3	0.58 × 0.13 × 0.13
Radiation	CuKα (λ = 1.54178)
2Θ range for data collection/°	8.858 to 137.782
Index ranges	-17 ≤ h ≤ 17, -19 ≤ k ≤ 19, -24 ≤ l ≤ 24
Reflections collected	126537
Independent reflections	4176 [R_{int} = 0.0579, R_{sigma} = 0.0175]
Data/restraints/parameters	4176/0/311
Goodness-of-fit on F^2	1.040
Final R indexes [I>=2σ (I)]	R_1 = 0.0376, wR_2 = 0.1007
Final R indexes [all data]	R_1 = 0.0403, wR_2 = 0.1033
Largest diff. peak/hole / e Å^{-3}	0.26/-0.24

Table S38. Angles between the planes of pairs of connected rings in photoswitch *E*-**5.**

Pairs of connected rings	Angle/°
Ring containing C4 and C7	84.8
Ring containing C18 and C21	89.5

13.5 Single crystal X-ray structure of ligand *E*-1 (CCDC: 2552329)

Crystals suitable for X-ray crystallography were grown by the slow evaporation of a solution of *E*-**1** in acetonitrile and dichloromethane (5:1). A block-like single crystal with dimensions of 0.15 × 0.12 × 0.02 mm was selected under a polarizing microscope (Leica M165Z), and was mounted on a MicroMount (MiTeGen, USA), consisting of a thin polymer tip with a wicking aperture. The X-ray diffraction measurements were carried out on a Bruker D8 Quest Single Crystal diffractometer with a Photon II detector at 120 K by using µS 3.0 Microfocus Source with Cu-Kα radiation (λ = 1.54178 Å). The single crystal was coated with NVH immersion oil and then quickly transferred to the cold nitrogen stream using the Oxford Cryostream 800 series attachment, and it was mounted on the goniometer using a cryo loop for intensity measurement. The solutions were obtained by intrinsic phasing using SHELXT[12] followed by successive refinements using full-matrix least-squares method against F^2 using SHELXL[13] as implemented within OLEX2-1.5[14]. All non-hydrogen atoms were refined with anisotropic thermal parameters, with hydrogen atoms being added geometrically and refined using riding thermal parameters. The ring containing C14 and the azobond was disordered and was modelled over two positions with 0.5 occupancies each. Geometrical restraints and restraints for their thermal parameter had to be applied. The crystal structure of *E*-**1** was refined as a two-component twin.

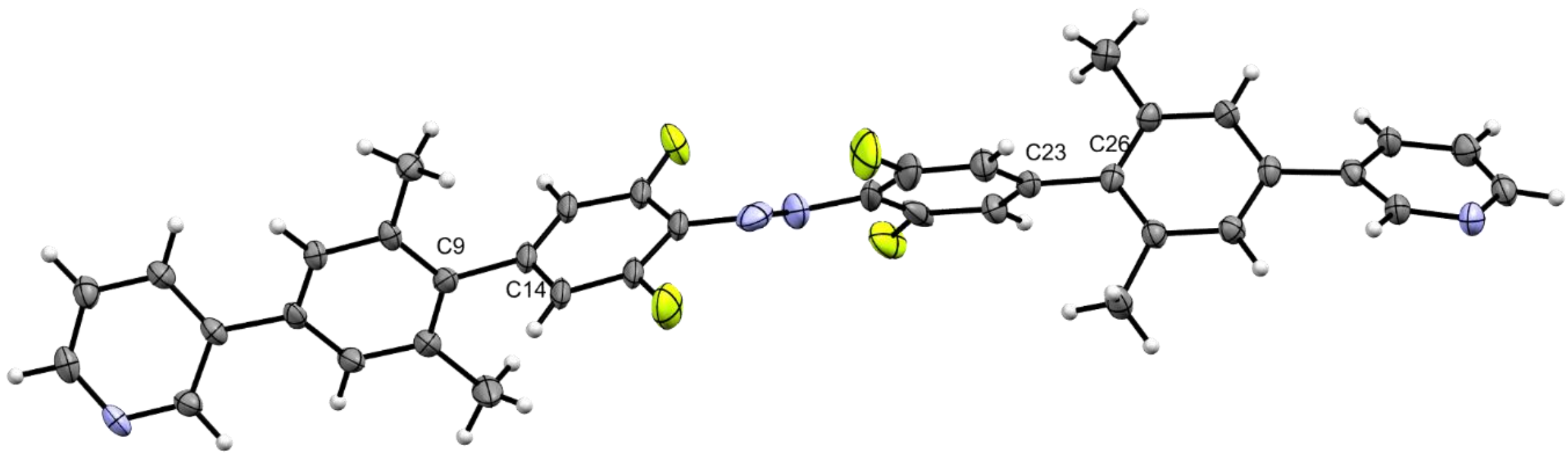

Figure S151. ORTEP type representation of ligand *E*-**1**. Ellipsoids drawn at 50% probability.

Table S39. Crystal data and structure refinement for *E*-**1**.

Crystallographic details	*E*-**1**
Empirical formula	$C_{38}H_{28}F_4N_4$
Formula weight	616.64
Temperature/K	120.00
Crystal system	monoclinic
Space group	$P2_1/c$
a/Å	21.8583(17)
b/Å	11.2278(9)
c/Å	12.2601(9)
α/°	90
β/°	96.893(5)
γ/°	90
Volume/Å^3	2987.1(4)
Z	4
ρ_{calc}g/cm^3	1.371
μ/mm^{-1}	0.819
F(000)	1280.0
Crystal size/mm^3	0.15 × 0.12 × 0.02
Radiation	CuKα (λ = 1.54178)
2Θ range for data collection/°	4.072 to 137.602
Index ranges	-26 ≤ h ≤ 26, -13 ≤ k ≤ 13, -14 ≤ l ≤ 14
Reflections collected	81580
Independent reflections	81580 [R_{int} = ?, R_{sigma} = 0.1222]
Data/restraints/parameters	81580/6/424
Goodness-of-fit on F^2	1.078
Final R indexes [I>=2σ (I)]	R_1 = 0.1115, wR_2 = 0.2773
Final R indexes [all data]	R_1 = 0.1528, wR_2 = 0.2973
Largest diff. peak/hole / e Å^{-3}	0.54/-0.57

Table S40. Angles between the planes of pairs of connected rings in ligand *E*-**1.**

Pairs of connected rings	Angle/°
Ring containing C9 and C14	87.1
Ring containing C23 and C26	78.9

13.6 Single crystal X-ray structure of ligand *Z*-1 (CCDC: 2552330)

Crystals suitable for X-ray crystallography were grown by the slow evaporation of a solution of *Z*-**1** in acetonitrile and dichloromethane (5:1). A block-like single crystal with dimensions of 0.20 × 0.15 × 0.02 mm was selected under a polarizing microscope (Leica M165Z), and was mounted on a MicroMount (MiTeGen, USA), consisting of a thin polymer tip with a wicking aperture. The X-ray diffraction measurements were carried out on a Bruker D8 Quest Single Crystal diffractometer with a Photon II detector at 120 K by using μS 3.0 Microfocus Source with Cu-Kα radiation (λ = 1.54178 Å). The single crystal was coated with NVH immersion oil and then quickly transferred to the cold nitrogen stream using the Oxford Cryostream 800 series attachment, and it was mounted on the goniometer using a cryo loop for intensity measurement. The solutions were obtained by intrinsic phasing using SHELXT[12] followed by successive refinements using full-matrix least-squares method against F^2 using SHELXL[13] as implemented within OLEX2-1.5[14]. All non-hydrogen atoms were refined with anisotropic thermal parameters, with hydrogen atoms being added geometrically and refined using riding thermal parameters.

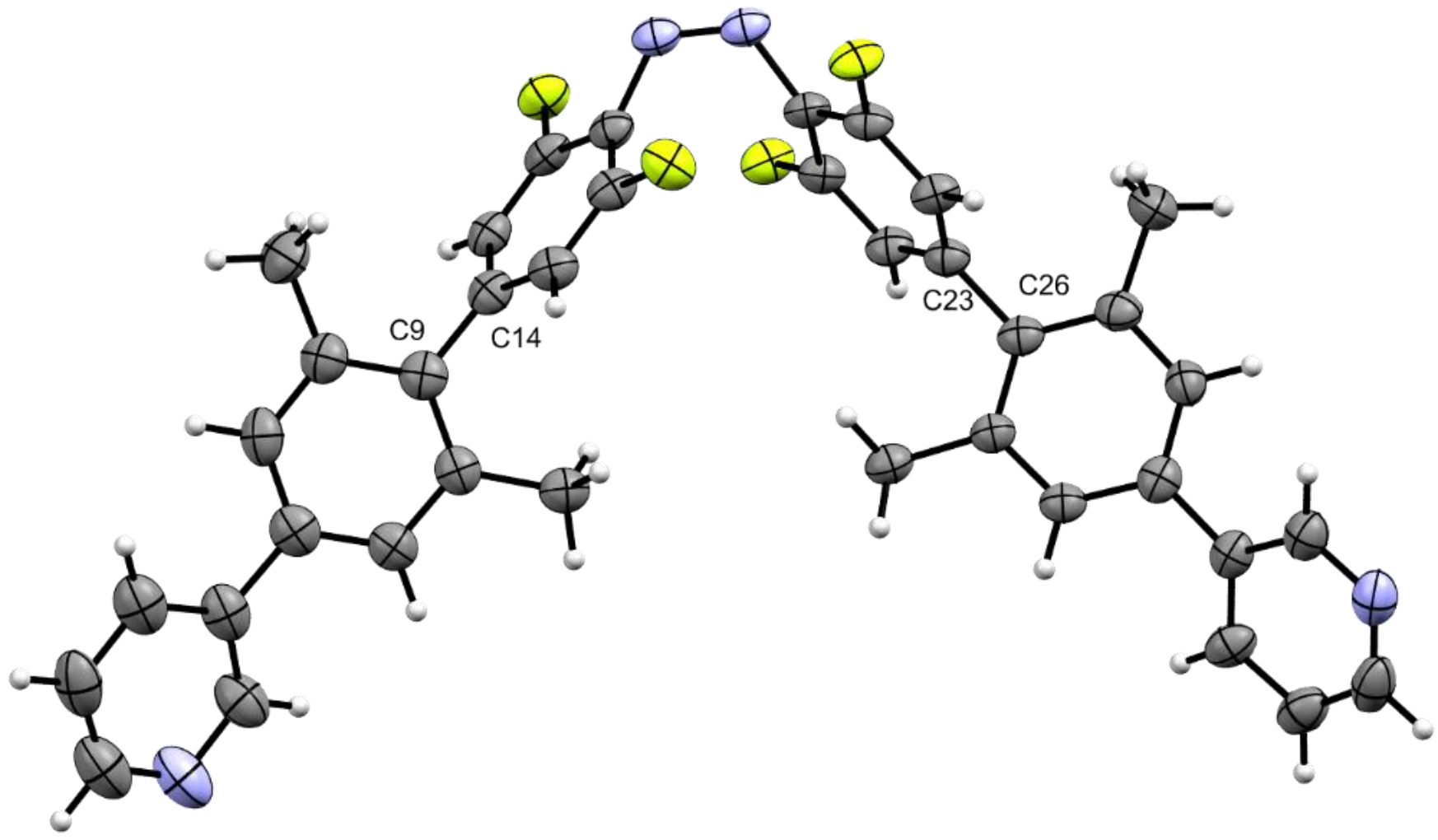

Figure S152. ORTEP type representation of ligand *Z*-**1**. Ellipsoids drawn at 50% probability.

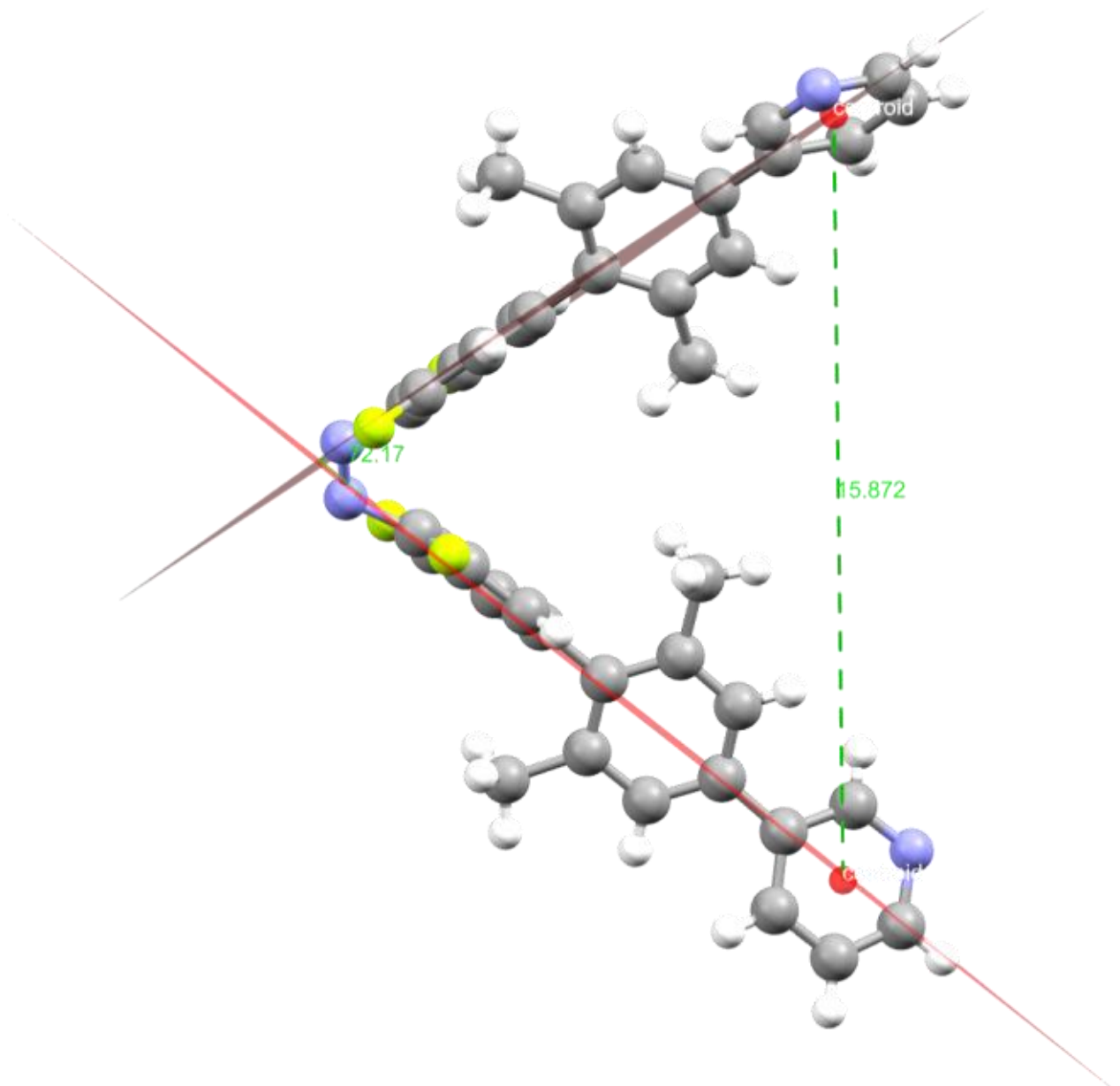

Figure S153. Pyridine centroid-centroid distance and angle between two planes in *Z*-**1**.

Table S41. Crystal data and structure refinement for *Z*-**1**.

Crystallographic details	***Z*-1**
Empirical formula	$C_{38}H_{28}F_4N_4$
Formula weight	616.64
Temperature/K	120.00
Crystal system	monoclinic
Space group	C2/c
a/Å	39.263(3)
b/Å	10.0850(10)
c/Å	16.9072(10)
α/°	90
β/°	95.584(7)
γ/°	90
Volume/Å^3	6662.8(10)
Z	8
ρ_{calc}g/cm^3	1.229
μ/mm^{-1}	0.735
F(000)	2560.0
Crystal size/mm^3	0.2 × 0.15 × 0.02
Radiation	CuKα (λ = 1.54178)
2Θ range for data collection/°	4.522 to 126.414
Index ranges	-44 ≤ h ≤ 45, -11 ≤ k ≤ 11, -19 ≤ l ≤ 19
Reflections collected	74134
Independent reflections	5365 [R_{int} = 0.0855, R_{sigma} = 0.0314]
Data/restraints/parameters	5365/0/419
Goodness-of-fit on F^2	1.029
Final R indexes [I>=2σ (I)]	R_1 = 0.0593, wR_2 = 0.1419
Final R indexes [all data]	R_1 = 0.0841, wR_2 = 0.1640
Largest diff. peak/hole / e Å^{-3}	0.24/-0.25

Table S42. Angles between the planes of pairs of connected rings in ligand *Z*-**1.**

Pairs of connected rings	Angle/°
Ring containing C9 and C14	63.0
Ring containing C23 and C26	71.6

13.7 Single crystal X-ray structure of $[Pd_2(Z\text{-}1)_2(2)_2](BF_4)_4$ (CCDC: 2580400)

Yellow plate-like single crystals of $[Pd_2(Z\text{-}\mathbf{1})_2(\mathbf{2})_2](BF_4)_4$ were grown in a 5 mm NMR tube by layering a diisopropyl ether solution over an acetonitrile solution of $[Pd_2(Z\text{-}\mathbf{1})_2(\mathbf{2})_2](BF_4)_4$ and keeping the sample at 5 °C. A single crystal with dimensions of 0.20 × 0.08 × 0.03 mm was selected under the polarizing microscope using the cold mounting setup, and was then mounted on a MicroMount, consisting of a thin polymer tip with a wicking aperture. X-ray diffraction data were collected at 100 K on the MX3[15] Macromolecular Crystallography beamline at the Australian Synchrotron at a wavelength of 0.65255 Å. Cell refinement and data reduction with XDS[16], CCTBX[17] and CCP4[18] interfaced through FastDP[19]. The solutions were obtained by intrinsic phasing using SHELXT[12] followed by successive refinements using full-matrix least-squares method against F^2 using SHELXL[13] as implemented within OLEX2-1.5[14]. All non-hydrogen atoms were refined with anisotropic thermal parameters, with hydrogen atoms being added geometrically and refined using riding thermal parameters. Due to the software set up at Australian Synchrotron, absorption correction and transmission factors are not reported.

The crystals were small and suffered from rapid solvent loss upon removal from the mother liquor. Despite cold mounting the sample and the use of synchrotron radiation we could not unambiguously model the four BF_4^- counter anions and the acetonitrile lattice solvent. A solvent mask in Olex2 was calculated and 571 electrons were found in a volume of 2133 Å^3 in 1 void per unit cell. This is consistent with the presence of 4 BF_4^- counter anions and 6 CH_3CN solvent molecules per asymmetric unit which account for 592 electrons per unit cell. The four *ortho*-methylated aromatic rings in the two ligands **1** were disordered over two positions with equal occupancy. The ortho-fluoroazobenzene unit in one of the two ligands **1** was disordered over two positions and was modelled in two parts equal occupancy. Differences in ADPs of the carbon atoms in some phenyl rings were treated using AFIX 66 and EADP constraints.

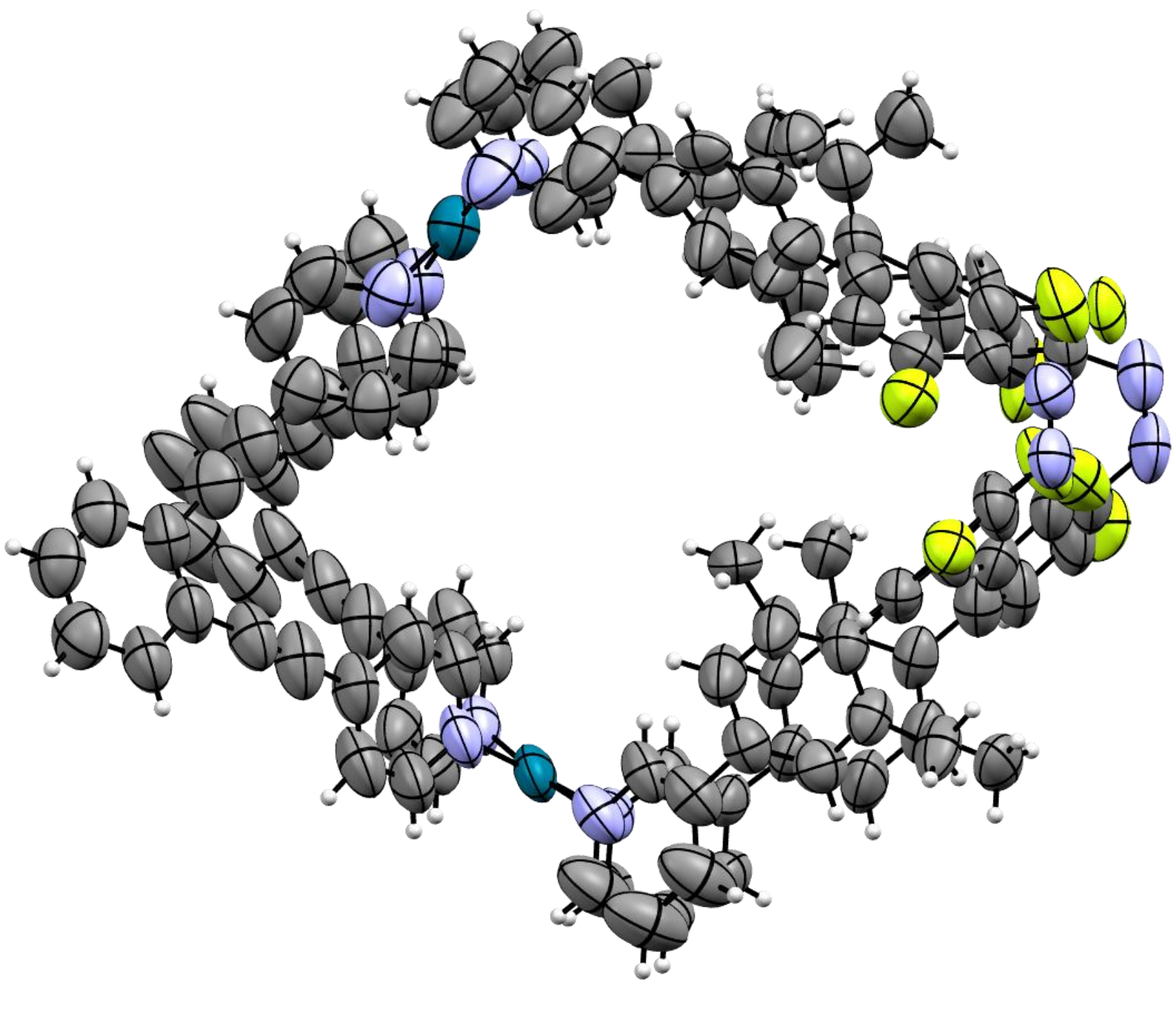

Figure S 154. ORTEP type representation of part 1 in $[Pd_2(Z\text{-}\mathbf{1})_2(\mathbf{2})_2](BF_4)_4\cdot 6CH_3CN$. The disordered part 2 was omitted for clarity. Ellipsoids drawn at 50% probability.

Table S43. Crystal data and structure refinement for $[Pd_2(Z\text{-}\mathbf{1})_2(\mathbf{2})_2](BF_4)_4{\cdot}6CH_3CN$.

Crystallographic details	$[Pd_2(Z\text{-}\mathbf{1})_2(\mathbf{2})_2](BF_4)_4{\cdot}6CH_3CN$
Empirical formula	$C_{128}H_{98}B_4F_{24}N_{18}Pd_2$
Formula weight	2600.28
Temperature/K	100(2)
Crystal system	triclinic
Space group	P-1
a/Å	14.8670(3)
b/Å	15.3375(3)
c/Å	29.7823(6)
α/°	89.834(2)
β/°	78.270(2)
γ/°	72.544(2)
Volume/Å^3	6330.7(2)
Z	2
ρ_{calc}g/cm^3	1.364
μ/mm^{-1}	0.302
F(000)	2640.0
Crystal size/mm^3	0.2 × 0.08 × 0.03
Radiation	Synchrotron (λ = 0.65255)
2Θ range for data collection/°	2.56 to 44.592
Index ranges	-17 ≤ h ≤ 17, -17 ≤ k ≤ 17, -34 ≤ l ≤ 34
Reflections collected	70755
Independent reflections	19811 [R_{int} = 0.0527, R_{sigma} = 0.0673]
Data/restraints/parameters	19811/3324/1359
Goodness-of-fit on F^2	1.105
Final R indexes [I>=2σ (I)]	R_1 = 0.1047, wR_2 = 0.3136
Final R indexes [all data]	R_1 = 0.1442, wR_2 = 0.3387
Largest diff. peak/hole / e Å^{-3}	0.95/-1.13

14 References

[1] T. Tateishi, S. Takahashi, A. Okazawa, V. Marti-Centelles, J. Wang, T. Kojima, P. J. Lusby, H. Sato, S. Hiraoka, "Navigated Self-Assembly of a Pd(2)L(4) Cage by Modulation of an Energy Landscape under Kinetic Control", *J. Am. Chem. Soc.* **2019**, *141*, 19669-19676.

[2] R. G. DiNardi, S. Rasheed, S. S. Capomolla, M. H. Chak, I. A. Middleton, L. K. Macreadie, J. P. Violi, W. A. Donald, P. J. Lusby, J. E. Beves, "Photoswitchable Catalysis by a Self-Assembled Molecular Cage", *J. Am. Chem. Soc.* **2024**, *146*, 21196-21202.

[3] K. Suzuki, M. Kawano, M. Fujita, "Solvato-Controlled Assembly of Pd3L6 and Pd4L8 Coordination "Boxes"", *Angew. Chem. Int. Ed.* **2007**, *46*, 2819-2822.

[4] A. D. W. Kennedy, R. G. DiNardi, L. L. Fillbrook, W. A. Donald, J. E. Beves, "Visible-Light Switching of Metallosupramolecular Assemblies**", *Chem.– Eur. J.* **2022**, *28*, e202104461.

[5] N. Oka, T. Yamada, H. Sajiki, S. Akai, T. Ikawa, "Aryl Boronic Esters Are Stable on Silica Gel and Reactive under Suzuki-Miyaura Coupling Conditions", *Org. Lett.* **2022**, *24*, 3510-3514.

[6] E. Zysman-Colman, K. Arias, J. S. Siegel, "Synthesis of arylbromides from arenes and N-bromosuccinimide (NBS) in acetonitrile — A convenient method for aromatic bromination", *Can. J. Chem.* **2009**, *87*, 440-447.

[7] P. Thordarson, https://supramolecular.org

[8] P. Thordarson, "Determining association constants from titration experiments in supramolecular chemistry", *Chem. Soc. Rev.* **2011**, *40*, 1305-1323.

[9] R. G. DiNardi, A. O. Douglas, R. Tian, J. R. Price, M. Tajik, W. A. Donald, J. E. Beves, "Visible-Light-Responsive Self-Assembled Complexes: Improved Photoswitching Properties by Metal Ion Coordination", *Angew. Chem. Int. Ed.* **2022**, *61*, e202205701.

[10] W. S. Price, *NMR Studies of Translational Motion: Principles and Applications*, Cambridge University Press, Cambridge, **2009**.

[11] E. O. Stejskal, J. E. Tanner, "Spin Diffusion Measurements: Spin Echoes in the Presence of a Time-Dependent Field Gradient", *J. Chem. Phys.* **1965**, *42*, 288-292.

[12] G. M. Sheldrick, "A short history of SHELX", *Acta Crystallogr A* **2008**, *64*, 112-122.

[13] G. M. Sheldrick, "Crystal structure refinement with SHELXL", *Acta Crystallogr. C Struct. Chem.* **2015**, *71*, 3-8.

[14] O. V. Dolomanov, L. J. Bourhis, R. J. Gildea, J. A. K. Howard, H. Puschmann, "OLEX2: a complete structure solution, refinement and analysis program", *J. Appl. Crystallogr.* **2009**, *42*, 339-341.

[15] T. M. Mcphillips, S. E. Mcphillips, H.-J. Chiu, C. A. E., A. M. Deacon, P. J. Ellis, E. Garman, A. Gonzalez, N. K. Sauter, R. P. Phizackerley, S. M. Soltisa, P. Kuhn, "Blu-Ice and the DistributedControlSystem: software for data acquisition and instrument control at macromolecular crystallography beamlines", *J. Synchrotron Rad.* **2002**, *9*, 401-406.

[16] W. Kabsch, "Xds", *Acta Crystallogr. D Biol. Crystallogr.* **2010**, *66*, 125-132.

[17] R. W. Grosse-Kunstleve, N. K. Sauter, N. W. Moriarty, P. D. Adams*, "The Computational Crystallography Toolbox: crystallographic algorithms in a reusable software framework", *J. Appl. Crystallogr.* **2002**, *35*, 126-136.

[18] M. D. Winn, C. C. Ballard, K. D. Cowtan, E. J. Dodson, P. Emsley, P. R. Evans, R. M. Keegan, E. B. Krissinel, A. G. Leslie, A. McCoy, S. J. McNicholas, G. N. Murshudov, N. S. Pannu, E. A. Potterton, H. R. Powell, R. J. Read, A. Vagin, K. S. Wilson, "Overview of the CCP4 suite and current developments", *Acta Crystallogr. D Biol. Crystallogr.* **2011**, *67*, 235-242.

[19] G. Winter, K. E. McAuley, "Automated data collection for macromolecular crystallography", *Methods* **2011**, *55*, 81-93.
